# Discovery of Potential SARS-CoV-2 Papain-like Protease Natural Inhibitors Employing a Multi-Phase In Silico Approach

**DOI:** 10.3390/life12091407

**Published:** 2022-09-09

**Authors:** Eslam B. Elkaeed, Ahmed M. Metwaly, Mohamed S. Alesawy, Abdulrahman M. Saleh, Aisha A. Alsfouk, Ibrahim H. Eissa

**Affiliations:** 1 Department of Pharmaceutical Sciences, College of Pharmacy, AlMaarefa University, Riyadh 13713, Saudi Arabia; 2 Pharmacognosy and Medicinal Plants Department, Faculty of Pharmacy (Boys), Al-Azhar University, Cairo 11884, Egypt; 3 Biopharmaceutical Products Research Department, Genetic Engineering and Biotechnology Research Institute, City of Scientific Research and Technological Applications (SRTA-City), Alexandria 21934, Egypt; 4 Pharmaceutical Medicinal Chemistry & Drug Design Department, Faculty of Pharmacy (Boys), Al-Azhar University, Cairo 11884, Egypt; 5 Department of Pharmaceutical Sciences, College of Pharmacy, Princess Nourah bint Abdulrahman University, P.O. Box 84428, Riyadh 11671, Saudi Arabia

**Keywords:** papain-like protease, SARS-CoV-2, natural products, structural similarity, molecular docking, ADMET, DFT

## Abstract

As an extension of our research against COVID-19, a multiphase *in silico* approach was applied in the selection of the three most common inhibitors (Glycyrrhizoflavone (**76**), Arctigenin (**94**)**,** and Thiangazole (**298**)) against papain-like protease, PLpro (PDB ID: 4OW0), among 310 metabolites of natural origin. All compounds of the exam set were reported as antivirals. The structural similarity between the examined compound set and **S88**, the co-crystallized ligand of PLpro, was examined through structural similarity and fingerprint studies. The two experiments pointed to Brevicollin (**28**), Cryptopleurine (**41**), Columbamine (**46**), Palmatine (**47**), Glycyrrhizoflavone (**76**), Licochalcone A (**87**), Arctigenin (**94**), Termilignan (**98**), Anolignan B (**99**), 4,5-dihydroxy-6″-deoxybromotopsentin (**192**), Dercitin (**193**), Tryptanthrin (**200**), 6-Cyano-5-methoxy-12-methylindolo [2, 3A] carbazole (**211**), Thiangazole (**298**), and Phenoxan (**300**). The binding ability against PLpro was screened through molecular docking, disclosing the favorable binding modes of six metabolites. ADMET studies expected molecules **28**, **76**, **94**, **200**, and **298** as the most favorable metabolites. Then, molecules **76**, **94**, and **298** were chosen through *in silico* toxicity studies. Finally, DFT studies were carried out on glycyrrhizoflavone (**76**) and indicated a high level of similarity in the molecular orbital analysis. The obtained data can be used in further *in vitro* and *in vivo* studies to examine and confirm the inhibitory effect of the filtered metabolites against PLpro and SARS-CoV-2.

## 1. Introduction

As of 26 July 2022, the WHO stated the confirmation of the incidence of 57,223,945 COVID-19 infections and 6,390,401 deaths [1]. Accordingly, a constant search in the field of drug discovery should be sustained to discover a cure.

Cheminformatics (computational- *in silico*) labels the connection between informatics and chemistry [2]. This approach applies the software in the field of chemistry [3] and has been used effectively to predict a cure against COVID-19 [4,5,6]. The chemoinformatic approach was also employed efficiently in drug discovery [7], drug molecular design [8,9], computational chemistry [10,11], toxicity prediction [12], ADMET assessment [13], and DFT calculation [14].

Human interest in the use of natural products has been back-traced for thousands of years [15,16]. The power of natural products as antiviral medicines has been confirmed in several scientific reports [17,18,19,20].

PLpro is a crucial protein in the coronavirus that has an essential role in the processing mechanism of viral polyproteins. This step results in the generation of an efficient replicase complex [21]. PLpro has another essential role against human immunity through post-translational modifications on human proteins [22].

Against COVID-19, we employed *in silico* methods to disclose the potential inhibition of several types of natural compounds. For example, four isoflavonoids [23] and three alkaloids [24] were proposed to exert promising anti-SARS-CoV-2 activities. We designed and applied *in silico* experiments to recommend the most fitting inhibitor against certain essential enzymes of SARS-CoV-2 such as SARS-CoV-2 nsp10 [25], SARS-CoV-2 PLpro [26], SARS-CoV-2 nsp16-nsp10 2′-*o*-Methyltransferase Complex [27], SARS-CoV-2 M^Pro^ [28,29], and SARS-CoV-2 RdRp [30].

**Figure 1:**
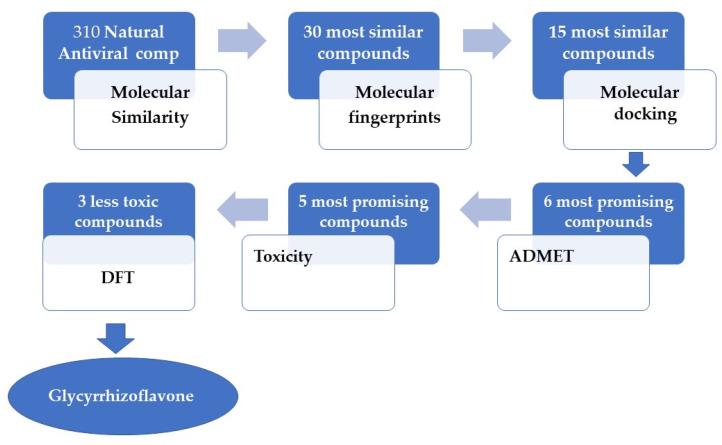
In silico protocol to select the most promising candidate against PLpro.

In the current study, we report the use of several computational filtration methods on 310 metabolites of natural origin that belong to diverse chemical classes and are reported as antivirals (Figure S1 and Table S1). Our experiments revealed the most expected inhibitors of human coronavirus PLpro among them. We depended on the reported similarities between the PLpro of SARS-CoV-1 and SARS-CoV-2 (Figure 1).

## 2. Results and Discussion

### 2.1. Molecular Similarity

It is worth mentioning that **S88** was used as a positive control (lead molecule) in this work as **S88** is the co-crystallized ligand of our target protein and has a reported binding mode. Additionally, currently, there are no FDA-approved drugs for the treatment of coronavirus targeting PLpro. Accordingly, it was found that **S88** may serve as a good candidate to check the similarity of our compounds against it.

The following descriptors (H-bond donor (HBA) [31], H-bond acceptor (HBD) [32], partition coefficient (ALog p) [33], molecular weight (M. Wt) [34], rotatable bonds [35], rings, and aromatic rings [36] besides molecular fractional polar surface area (MFPSA) [37]) were examined between the 310 metabolites (Figure S1, Supplementary data) and **S88** using Discovery Studio software (Vélizy-Villacoublay, France). The degree of likeness was calculated through the computation of minimum distances. The minimum distances were computed based on the variations in the aforementioned parameters and represent the computed quantitative difference in the structure between **S88** and the examined compounds and are inversely proportional to the similarity degree.

**Figure 2:**
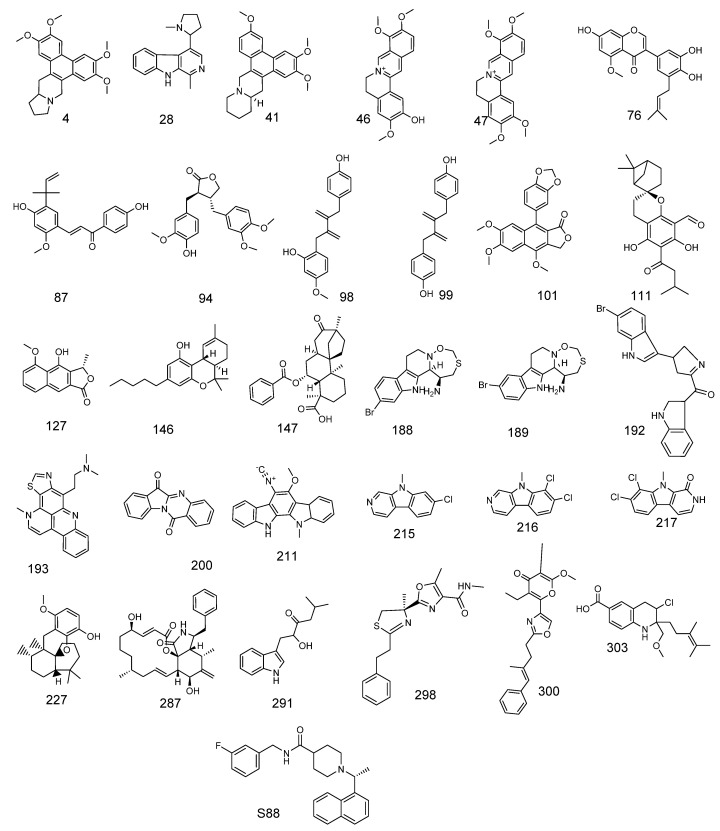
Thirty molecules with good molecular similarity with **S88**.

**Figure 3:**
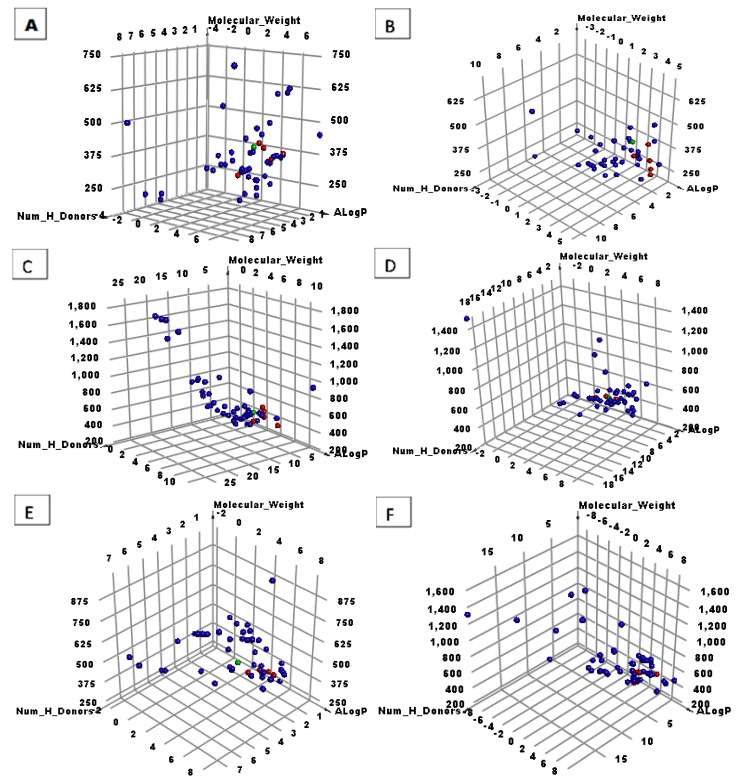
The similarity outputs of the tested compounds and **S88**. Green balls = **S88**, red balls = similar molecules, blue balls = not similar molecules. (**A**) First 50 molecules, (**B**) second 50 molecules, (**C**) third 50 molecules, (**D**) fourth 50 molecules, (**E**) fifth 50 molecules, and (**F**) last 60 molecules.

The 310 molecules were spit into five equal groups of 50 molecules each, and one (last group) that contained 60 molecules. The study determined the 30 most suitable metabolites (Figure 2 and Figure 3, and Table 1).

### 2.2. Filter Using Fingerprints

Various computational methods that describe the similarities between different molecules have gained more interest in drug discovery [38]. One of the most helpful techniques in this approach is fingerprints [39]. The fingerprint study includes binary strings that compute the existence or absence of vital sub-structural fragments to calculate the structural similarity between molecules. This technique is currently utilized in virtual screening and detection of similarities between hit compounds and the lead one. The main difference between the fingerprints and molecular similarity studies is that the first individually calculates the presence and or absence of certain descriptors in **S88** and the examined compounds, while molecular similarity calculates the degree of similarity between them as a whole structure.

The fingerprints technique was carried out using Discovery Studio software and examined the following parameters: HBA, HBD [40], charge [41], hybridization [42], positive and negative ionizable [43], halogen, aromatic, or none of them besides the ALogP category of atoms. All the mentioned parameters were converted to pits by the computer. Then, the computer calculated the bits in both **S88** and the target compounds (SA), in the target compounds only (SB), or **S88** only (SA). The identification of the most similar (that have the most identical molecular fingerprints) compounds to **S88** is important to pick compounds with a higher degree of similarities. The most similar compounds are expected to exert greater protein binding and activity.

The study (Table 2) favored **28**, **41**, **46**, **47**, **76**, **87**, **94**, **98**, **99**, **192**, **193**, **200**, **211**, **298**, and **300** due to their similarity with **S88**.

### 2.3. Docking Studies

**Figure 4:**
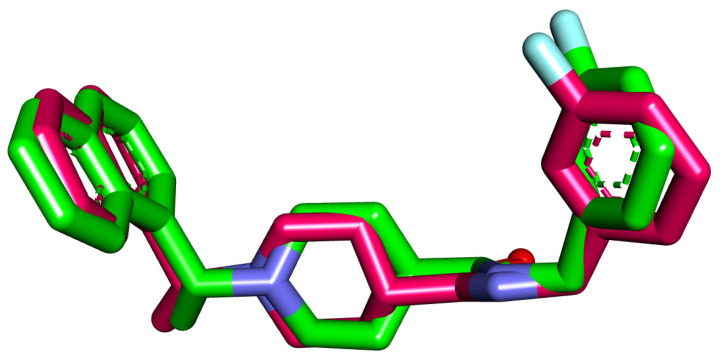
Superimposition of the co-crystallized pose (magenta) and the re-docking pose (turquoise) of the same ligand (**S88**) in the active site of the PLpro enzyme.

The docking analysis of **28**, **41**, **46**, **47**, **76**, **87**, **94**, **98**, **99**, **192**, **193**, **200**, **211**, **298**, and **300** was carried out against the coronavirus PLpro enzyme’s binding site (PDB ID: 4OW0). The crystallized ligand (**S88**) was used as a reference compound. For each compound, 30 run poses were carried out. The applied procedure of molecular docking was verified through the there-docking of **S88** against the PLpro active site for another time. The small value of the RMSD (0.98 Å) between the two poses indicated the applicability of the applied protocol (Figure 4).

Differentiation between the tested compounds for their binding affinity was dependent on certain factors. (i) The first factor is the correct binding mode of a tested compound. The compound that exerted a binding mode very close to **S88** was expected to have a good affinity against PLpro. The correct binding modes were determined according to the nature of the interactions (hydrogen or hydrophobic bonds) with the specific amino acid residues in the active pocket of PLpro. This factor is critical as a compound with the correct binding mode is expected to have a higher affinity than a compound with high binding energy having an incorrect binding mode. Therefore, the incorrect binding mode, resulting in incorrect affinity predictions, decreases the compound’s rate of virtual screening [44,45]. (ii) Gibbs free energy (ΔG binding) indicates the stability of the obtained conformation between the tested compound and PLpro (Table 3). According to the thermodynamic balance law, the value of ΔG is inversely proportional to the stability of the examined molecule and indicates that binding with PLpro will occur spontaneously. In other words, the increase in the negative free energy of a compound (reactant) will increase the reaction spontaneously and result in more stable conformations [46,47].

The molecular docking energy for compounds **76**, **94**, and **98** exhibited final values of −51.63, −50.82, and 52.21 kcal/mol, respectively. These values of free energies are the highest score indicating the spontaneity of the interactions and the stability of these compounds in the active site. Moreover, compounds **76**, **94**, and **98** have correct binding modes as these compounds formed many HBs with the crucial amino acid residues in the active sites. On the other hand, compounds **193** (ΔG = −44.02), **200** (ΔG = −41.31), and **298** (ΔG = −48.46) showed less free energies than some of the other tested compounds but had correct binding modes. For this reason, such compounds were selected for further investigation.

**Figure 5:**
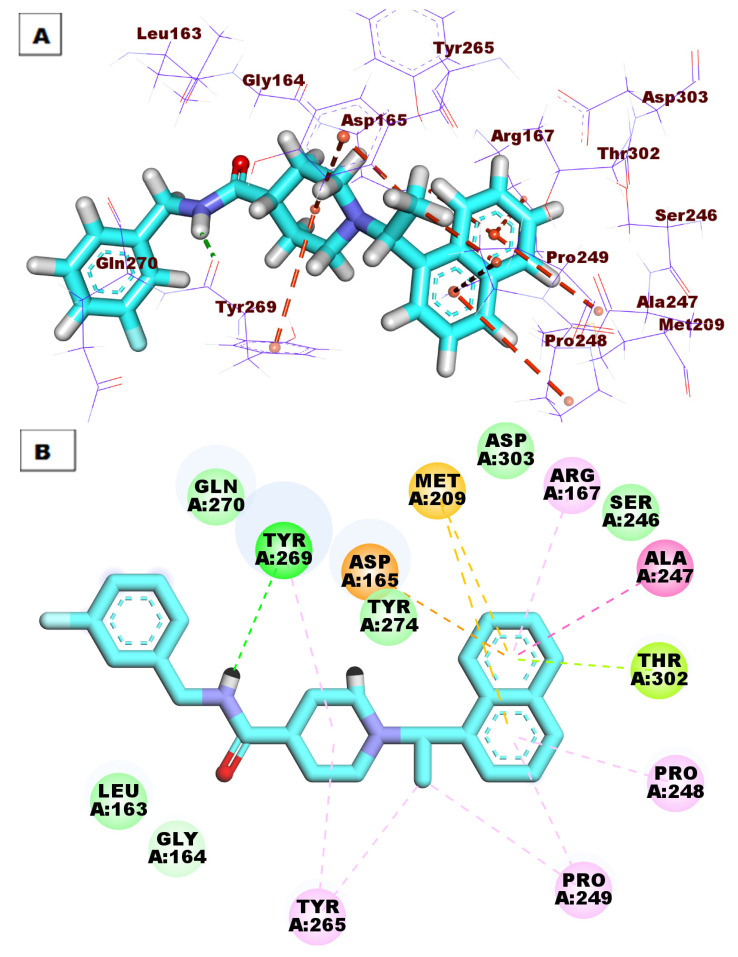

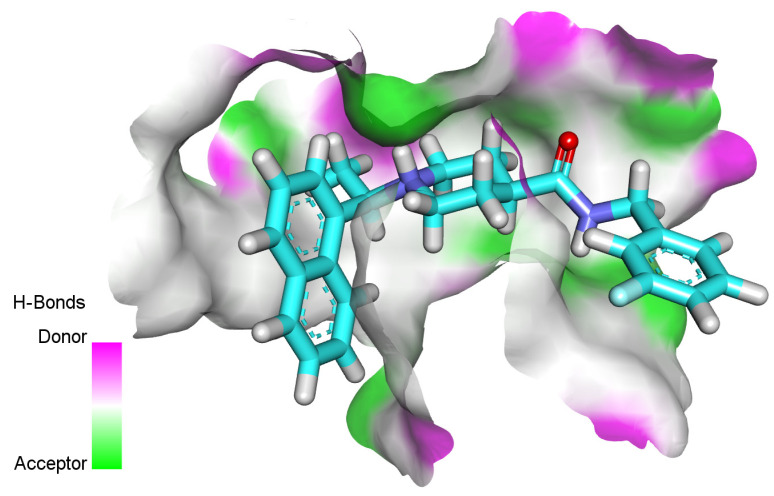
(**A**) Three-dimensional and (**B**) two-dimensional binding modes of **S88** in the active site of PLpro. As shown in Figure 6, compound **76** expressed a ΔG of −51.63 into the PLpro active site. Compound **76** made four HBs with Tyr265, Thr302, Tyr274, and Gln270. Moreover, the aromatic systems were included in many HIs with Asp165, Pro249, Tyr265, Gly164, Leu163, and Tyr269.

The proposed binding mode of **S88** expressed a ΔG of −59.13 kcal/mol. **S88** made one HB between its amide moiety and Tyr269. Additionally, the naphthyl moiety made eight hydrophobic interactions (HI) withAsp165, Met209, Arg167, Ala247, Thr302, Pro248, and Pro249. The ethyl bridge was included in two hydrophobic interactions with Pro249 and Tyr265. The piperidine moiety formed two hydrophobic bonds with Tyr265 and Tyr269. (Figure 5).

**Figure 7:**
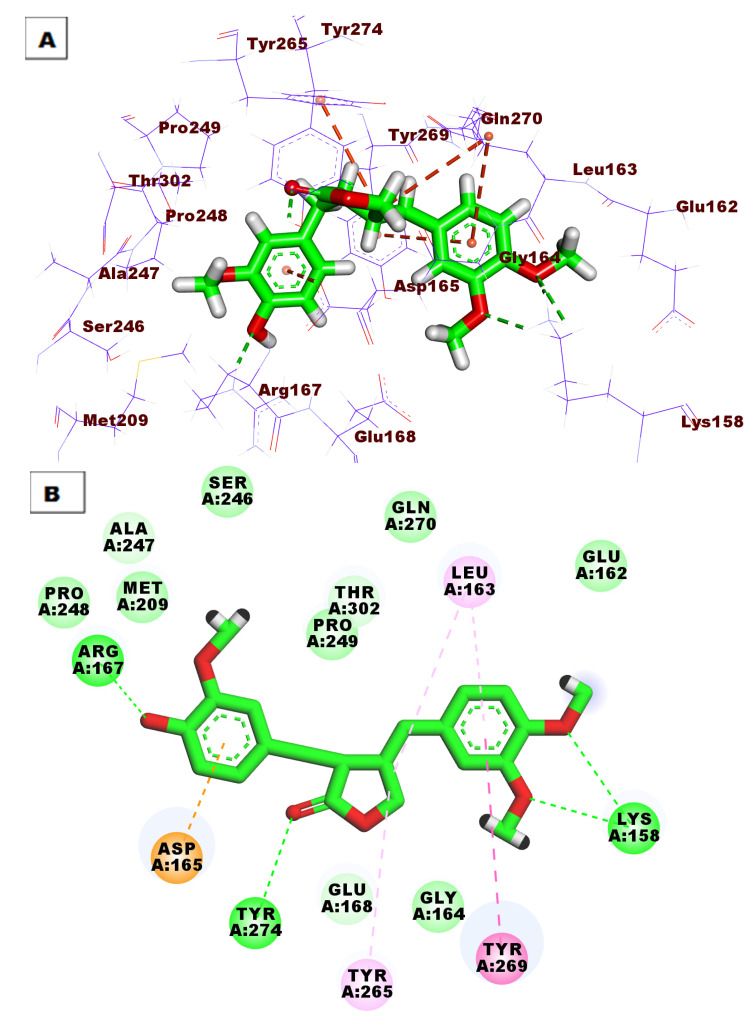

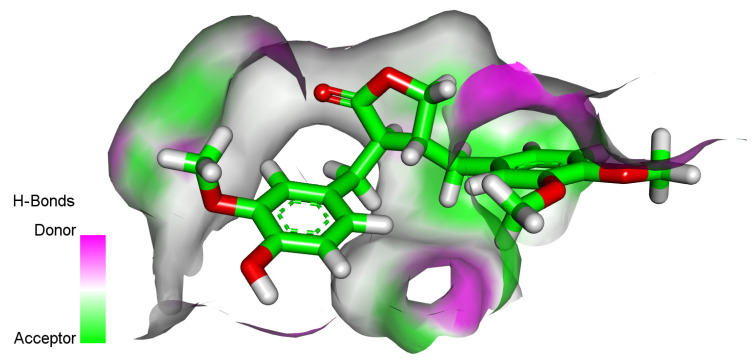
(**A**) Three-dimensional and (**B**) two-dimensional binding modes of compound **94** in the PLpro active site.

Compound **94** showed good binding energy (ΔG = −50.82) against the PLpro active site. It formed four HBs with Lys158, Tyr274, and Arg167. Additionally, the phenyl rings were involved in five HIs with Leu163, Tyr269, Tyr265, and Asp165 (Figure 7).

**Figure 8:**
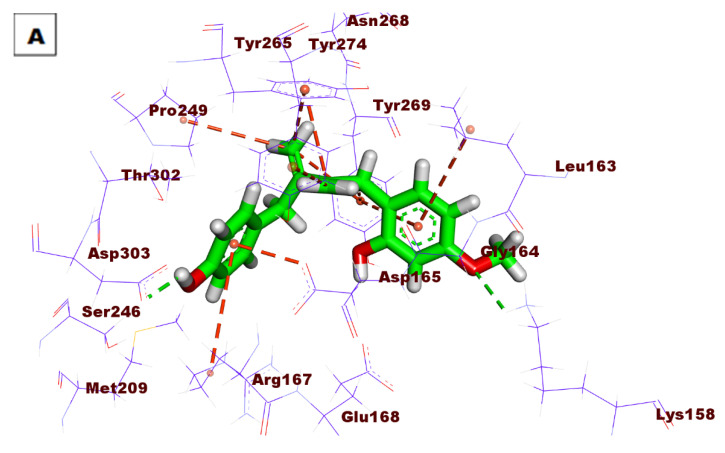

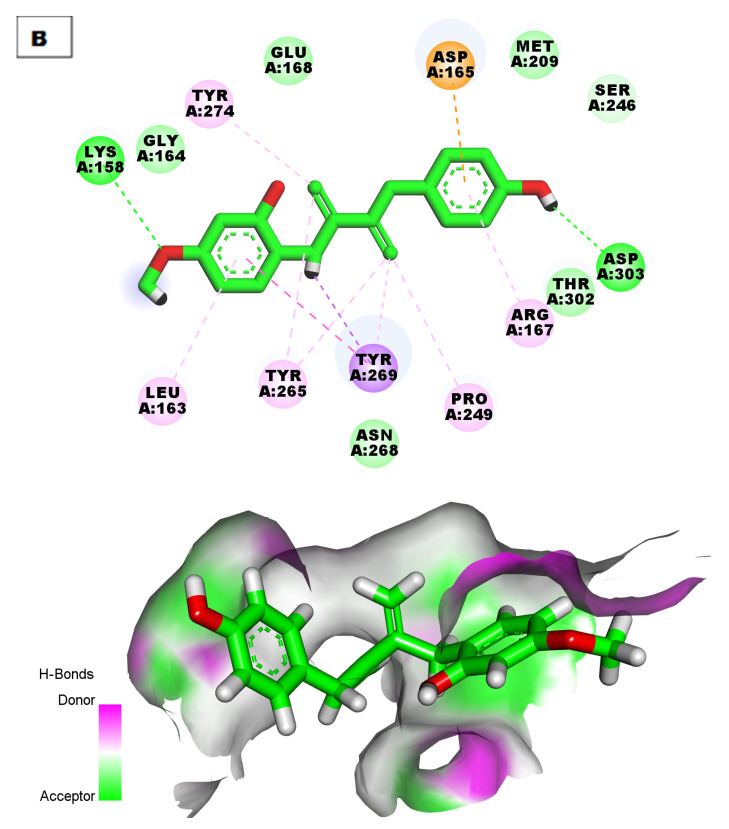
(**A**) Three-dimensional and (**B**) two-dimensional binding modes of compound **98** in the PLpro active site.

Compound **98** revealed good fitting with a docking score of −52.21 kcal/mol. The OH group formed one HB with Asp303, and the methoxy group formed another HB with Lys158. Many HIs were observed between the tested compound and Asp165, Arg167, Pro249, Tyr269, Tyr265, Leu163, and Tyr274 (Figure 8).

**Figure 9:**
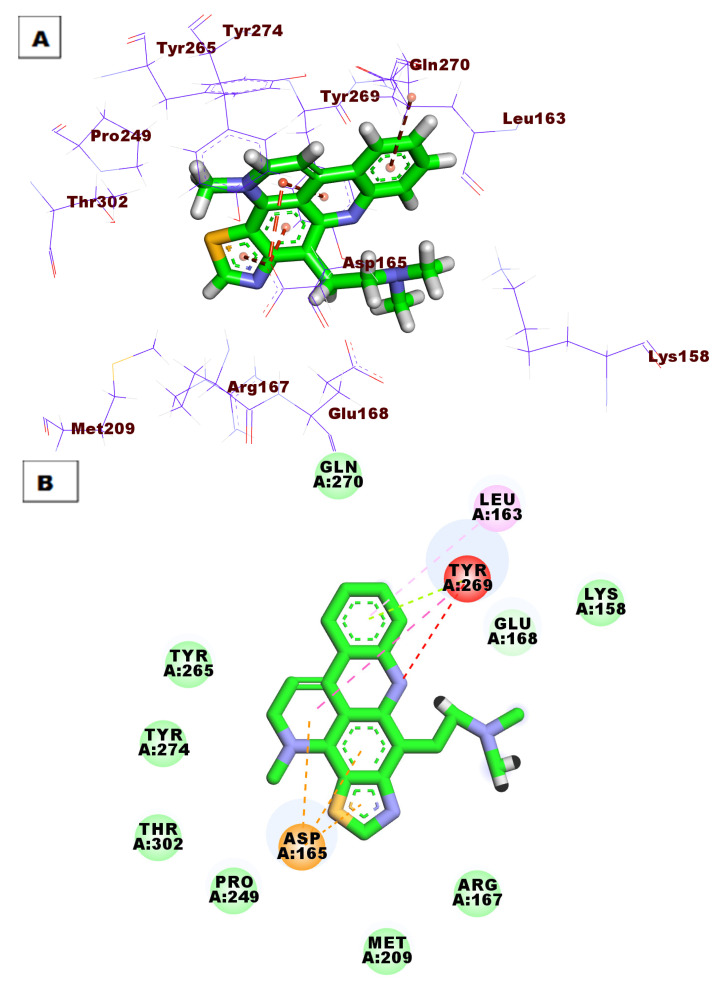

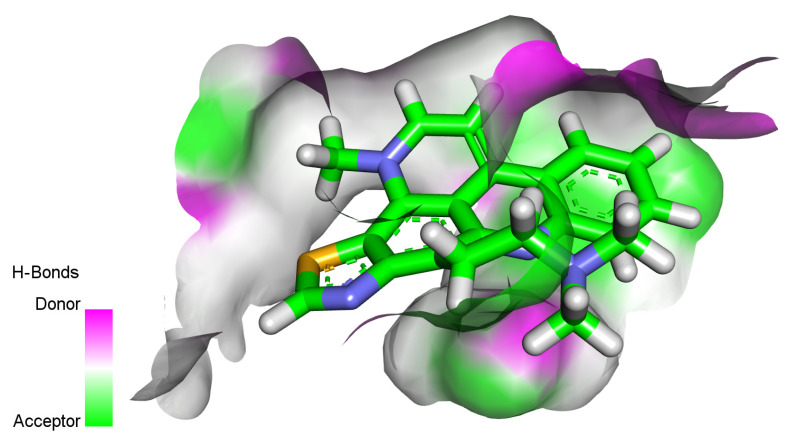
(**A**) Three-dimensional and (**B**) two-dimensional binding modes of compound **193** in the PLpro active site.

**Figure 10:**
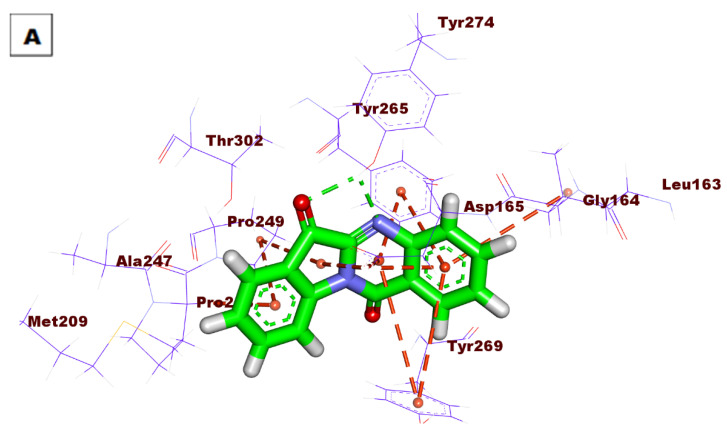

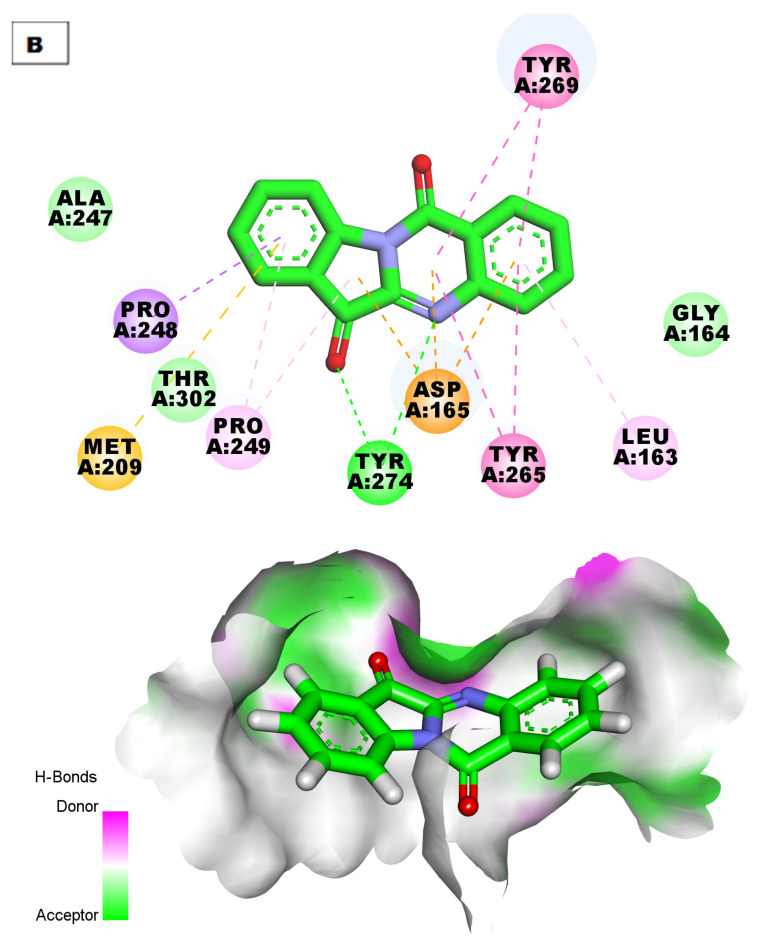
(**A**) Three-dimensional and (**B**) two-dimensional binding modes of compound **200** in the PLpro active site.

The top docking poses of compounds **193** and **200** (affinity values of −44.02 and −41.31 kcal/mol), respectively, were investigated. Compound **193** demonstrated eight HIs with Leu163, Tyr269, and Asp165 (Figure 9). The compound demonstrated two HBs with Tyr274. In addition, it formed 12 HIs, as shown in Figure 10.

**Figure 11:**
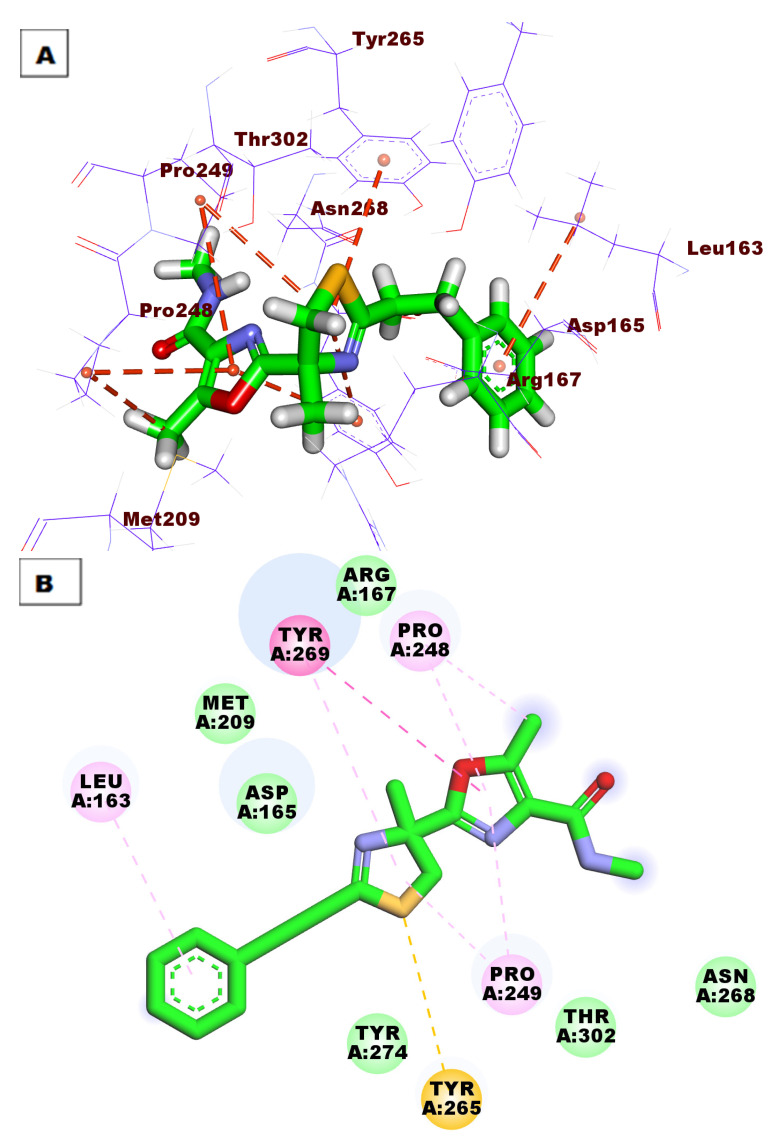

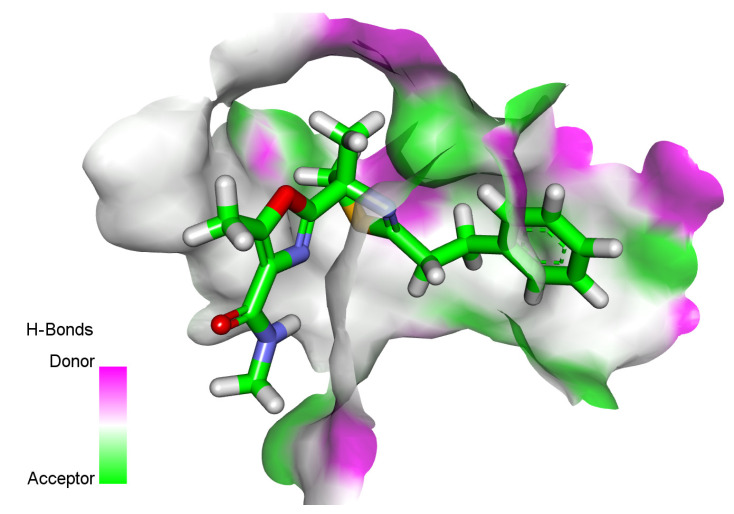
(**A**) Three-dimensional and (**B**) two-dimensional binding modes of compound **298** in the PLpro active site.

Compound **298** showed a binding mode against the PLpro active site with a binding affinity of −48.46 kcal/mol. It was incorporated in eight HIs with Pro248, Tyr265, Leu163, Tyr269, and Pro249 (Figure 11).

### 2.4. ADMET

**Figure 12:**
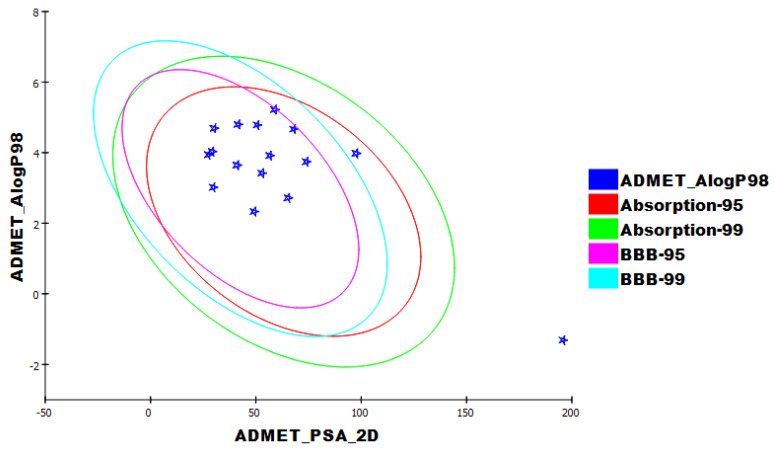
The expected ADMET characters.

ADMET studies were achieved using Discovery Studio 4.0, with remdesivir as a reference. The following descriptors were examined. (i) The ability to penetrate the blood–brain barrier [48] (BBB), intestinal absorption [49] (HIA), aqueous solubility [50] (S), CYP2D6 binding [51], hepatotoxicity, and plasma protein binding [52] (PPB). The calculated properties are listed in (Table 4). All compounds showed high levels of BBB penetration except molecules **28**, **76**, **94**, **200**, and **298**, which displayed medium to very low BBB levels. All the tested molecules showed good absorption characteristics comparable to remdesivir, which exhibited a very low level of absorption. Moreover, the solubility of the tested molecules was projected to be between low and good levels except for molecule **211,** which showed a very low level. All molecules in addition to remdesivir were calculated to be inhibitors against CYP2D6 except molecules **28**, **87**, **94**, **98**, **99**, **192**, **200**, **298** and **300**. All the tested molecules were expected to have unfavorable hepatotoxic effects except molecules **28**, **41**, and **192**, which were predicted to be non-toxic. All tested molecules and remdesivir were expected to bind to the plasma protein with a percentage of >90%, except molecule **46**, which demonstrated plasma protein binding <90%. (Figure 12).

### 2.5. Toxicity Studies

Toxicity predictions were made using Discovery Studio 4.0 software, which was based on validated and assembled models for the following parameters: the FDA rat carcinogenicity test [53,54], carcinogenic potentiality TD_50_ [55], maximum tolerated dose (MTD) in rats [56,57], oral LD_50_ in rats [58], chronic LOAEL in rats [59,60], ocular [61], and skin irritancies [61,62].

In silico testing revealed that the majority of molecules had expected low levels of toxicity (Table 5).

All compounds were expected to be non-carcinogens except molecules **28, 99**, **193**, **200**, **211**, and **300**, which were predicted to be carcinogens in the FDA rat carcinogenicity model.

Molecules **41**, **46**, **47**, **192**, and **211** showed TD_50_ values within range of (0.16 to 0.730 mg·kg^−1^/day), which were less than remdesivir (1.012 mg·kg^−1^/day), while molecules **28**, **76**, **87**, **94**, **98**, **99**, **193**, **200**, **298**, and **300** showed TD_50_ values within the range of (1.58 to 69.07 mg·kg^−1^/day), which were higher than remdesivir.

All molecules revealed an MTD within the range of 0.012 to 0.113 g·kg^−1^, less than remdesivir (0.235 g·kg^−1^), except molecules **99** and **192,** which demonstrated MTD of 0.240 and 1.099 g·kg^−1^, respectively, which are higher than remdesivir.

All molecules showed oral LD_50_ values higher than remdesivir (0.309 mg·kg^−1^/day) except compounds 41, 211, and 298, which exhibited oral LD_50_ values less than remdesivir ranging from 0.118 to 0.245 mg·kg^−1^/day.

Excluding compound 211, all the tested molecules showed LOAEL higher than that of remdesivir (0.003 g·kg^−1^), ranging from 0.008 to 0.398 g·kg^−1^.

Additionally, all molecules and remdesivir were expected to be mild ocular irritants, except molecules **87**, **98**, and **300**, which were non-irritant. On the other hand, the examined molecules were expected to be skin non-irritant except for molecules **28**, **94**, **193**, **211**, **300,** and remdesivir, which were mild irritants.

### 2.6. DFT Studies

**Figure 13:**
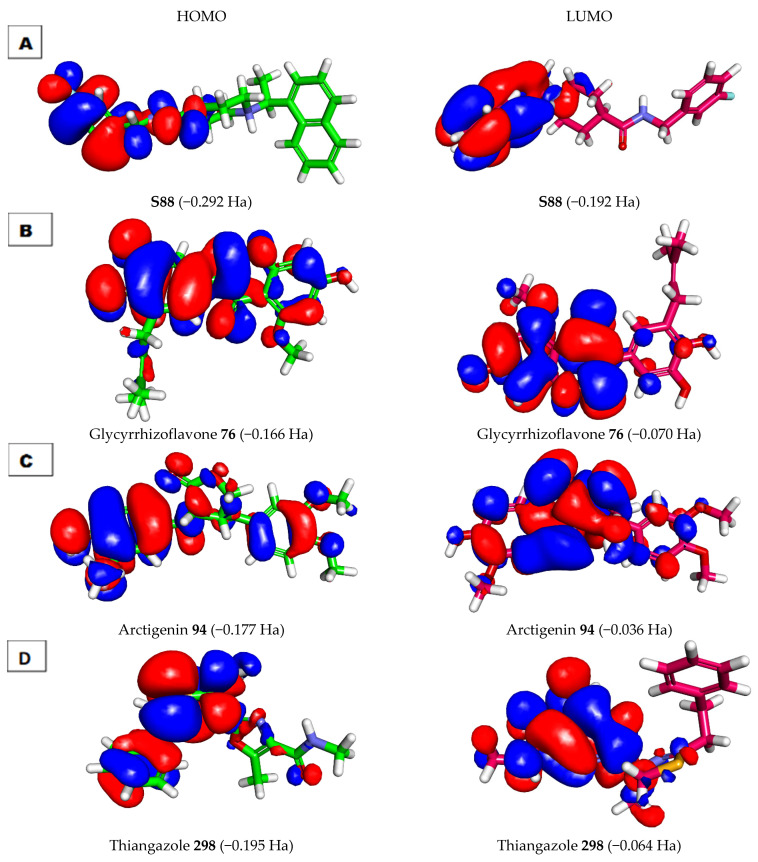
Molecular orbitals spatial distribution for (**A**) S88, (**B**) **76**, (**C**) **94**, and (**D**) **298**.

**Figure 14:**
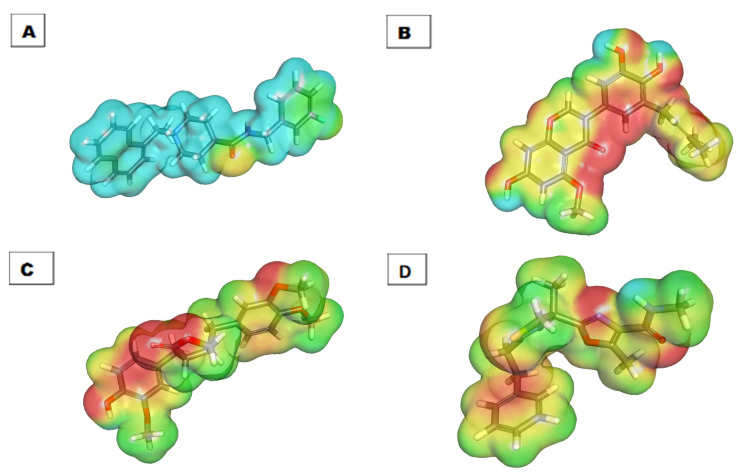
Molecular electrostatic potential map of (**A**) **S88**, (**B**) **76**, (**C**) **94**, and (**D**) **298**.

DFT parameters including binding energy [63], HOMO [64], LUMO [64], gap energy [65], and dipole moment [66,67] were studied for the most promising molecules, **76**, **94**, and **298,** using Discovery Studio software. **S88** was used as a reference. The results of the DFT studies are summarized in Table 6 and Figure 13 and Figure 14.

Molecules **76** and **94** showed higher values of dipole moment (1.700 and 3.582, respectively) than molecule **298** (1.094).

#### 2.6.1. Frontier Molecular Orbitals Analysis

Frontier molecular orbitals analysis can efficiently demonstrate active sites in addition to determining the kinetic stability and the chemical reactivity of a molecule [68]. The EHOMO and ELUMO of the tested molecules were computed using DMol3 implemented in Discovery Studio software [69]. The LUMO may be engaged in a nucleophilic attack, while the HOMO refers to the most probable site of an electrophilic attack. The HOMO energy represents the ionization potential of a drug, while that of the LUMO describes the electron affinity.

For gap energy, it was reported that a molecule is thought to be softer and more chemically reactive when its energy gap is small. In addition, a molecule was assumed to have greater chemical hardness and to be more stable when it had a large energy gap [70]. In this study, molecule **76** was found to have a low level of gap energy of 0.096 Ha, while molecules **94** and **298** were found to have high gap energy of 0.141 and 0.131, respectively. These findings indicate that compound **76** has higher reactivity than compounds **94** and **298**. On the contrary, compounds **94** and **298** may possess higher stability than compound **76**.

For the dipole moment values, compound **94** had a dipole moment value of 3.582. This value is nearly equal to that of S88 (3.621). The elevated dipole moment was expected to increase HBing, and non-bonded interactions in the compound–protein complexes were predicted to increase the binding affinity during SARS-CoV-2 inhibition. Compounds **76** and **298** had fewer values of the dipole moment of 1.700 and 1.094, respectively. From these findings, it can be concluded that compounds **76** and **94** have a higher chance of interacting with the target protein than compound **298** (Table 6 and Figure 13).

As shown in Figure 13B, the HOMO spatial distributions of molecule **76** were mainly distributed on the 3-(3,4-dihydroxyphenyl) -7-hydroxy-5-methoxy-4*H*-chromen-4-one moiety, while those of LUMO were located on the 7-hydroxy-5-methoxy-4*H*-chromen-4-one moiety (the electron acceptor zones).

The specific role of the HOMO center (3-(3,4-dihydroxyphenyl) -7-hydroxy-5-methoxy-4*H*-chromen-4-one moiety) in the binding of the receptor was previously confirmed by our docking experiments. As we noticed in Figure 13, the carbonyl group at position-4 of 4*H*-chromen-4-one (HOMO center) formed an H-bond acceptor with the phenolic OH group (LUMO center) of Tyr229. Furthermore, the LUMO of the accepting species (the two phenolic OH groups of catechol moiety) formed two H-bond donors with the HOMO of the donating species (OH group of Thr302 and OH group of Tyr274).

#### 2.6.2. Molecular Electrostatic Potential Maps (MEP)

MEP is a very helpful technique for understanding the 3D charge distributions over a molecule.

In MEP, the electronegative atoms are highlighted with red and can be acceptors in H-bonding interactions. On the other hand, the electron-poor atoms are highlighted in blue and are incorporated into H-bonds as donors. Finally, the neutral atoms are highlighted from green to yellow and incorporated in HIs [71].

The MEP map of molecule **76** shows that the negative potential sites are on oxygen atoms (seven red patches) and the positive potential sites are around the hydrogen atoms (six blue patches). This indicates that molecule **76** has seven positions available for H-bonding acceptors and six positions suitable for H-bond donors. This map defines the region in which the molecule can have non-covalent interactions (Figure 14).

The presented study preferred glycyrrhizoflavone (**76**) as the most relevant inhibitor of human coronavirus PLpro. Glycyrrhizoflavone is a flavonoid that has been isolated from licorice and *Glycyrrhiza glabra* roots [72]. Glycyrrhisoflavone exhibited potent antiviral activity against the human immunodeficiency virus by inhibiting giant cell formation in the infected cells and inhibiting viral transcription [73,74].

## 3. Conclusions

Several computational filtration methods (similarity assessment, fingerprints check, docking, ADMET, toxicity, and DFT) were carried out on 310 metabolites of natural origin that were reported as antivirals against PLpro, (PDB ID: 4OW0) and its co-crystallized ligand **S88**. The experiments predicted a high degree of binding between glycyrrhizoflavone (**76**) and PLpro. Accordingly, the potential of glycyrrhizoflavone to be an inhibitor against human coronavirus PLpro inhibitor is highly expected. More studies must be carried out on such a promising drug to affirm its inhibitory potential against PLpro.

## 4. Method

### 4.1. Molecular Similarity Detection

Was applied using Discovery Studio 4.0 software. Details have been discussed in detail in the Supplementary data.

### 4.2. Fingerprint Studies

Were applied using Discovery Studio 4.0 software. Details have been discussed in detail in the Supplementary data.

### 4.3. Docking Studies

Were applied using Discovery Studio 4.0 software. Details have been discussed in detail in the Supplementary data.

### 4.4. ADMET Analysis

Was applied using Discovery Studio 4.0 software. Details have been discussed in detail in the Supplementary data.

### 4.5. Toxicity Studies

Were applied using Discovery Studio 4.0 software. Details have been discussed in detail in the Supplementary data.

### 4.6. DFT Studies

Were applied using Discovery Studio 4.0 software. Details have been discussed in detail the Supplementary data.

## Figures and Tables

**Figure 6:**
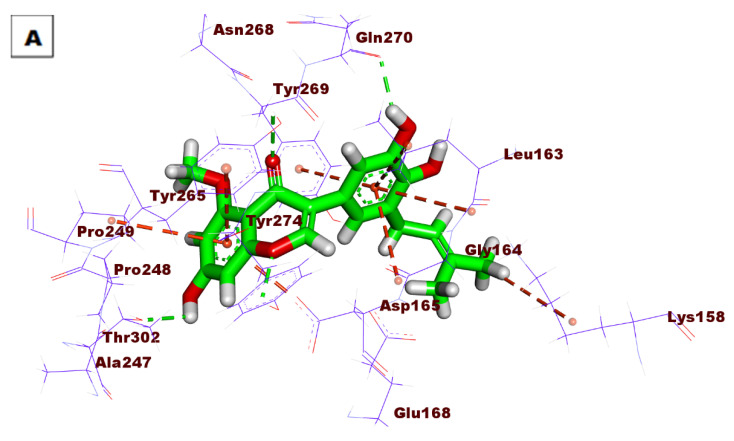

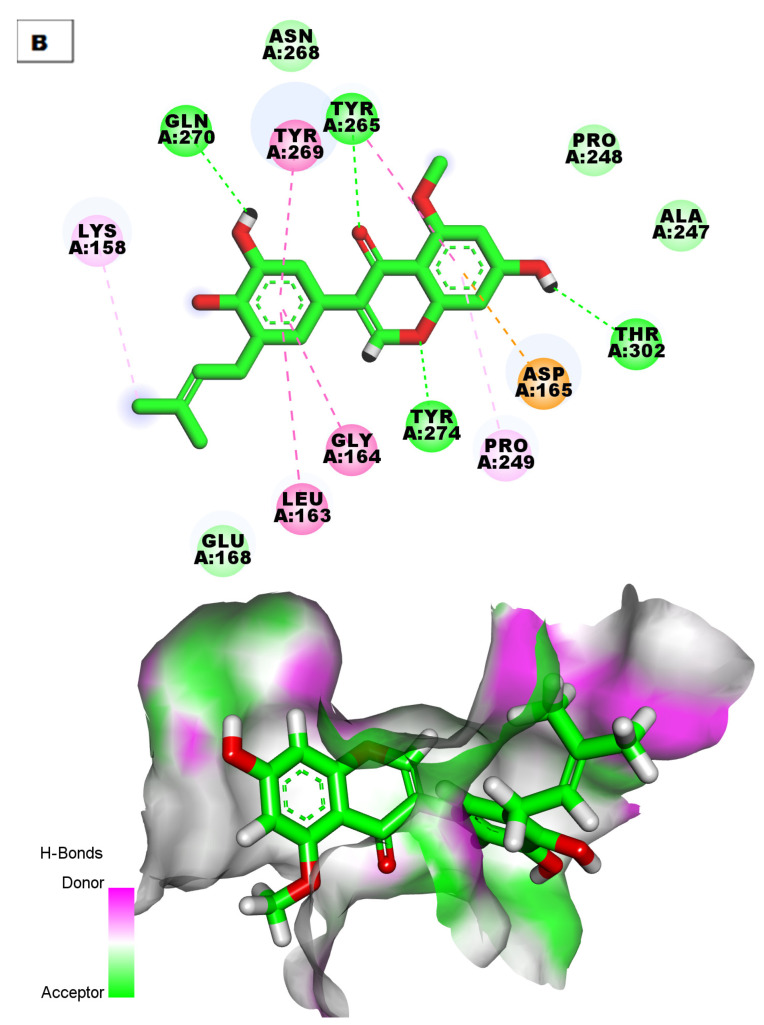
(**A**) Three-dimensional and (**B**) two-dimensional binding modes of compound **76** in the PLpro active site.

**Table 1:** Structural properties of the most similar molecules to **S88**.

Comp.	Molecular Formula	ALog p	M. Wt	HBA	HBD	Rotatable Bonds	Rings	Aromatic Rings	MFPSA	Minimum Distance
**4**	C_24_H_27_NO_4_	2.658	394.483	4	1	4	5	3	0.102	0.654
**28**	C_17_H_19_N_3_	1.457	266.361	1	2	1	4	3	0.119	0.693
**41**	C_24_H_27_NO_3_	3.131	378.484	3	1	3	5	3	0.083	0.546
**46**	C_20_H_20_NO_4_	3.936	338.377	4	1	3	4	3	0.149	0.709
**47**	C_21_H_22_NO_4_	4.161	352.404	4	0	4	4	3	0.11	0.714
**76**	C_21_H_20_O_6_	3.98	368.38	6	3	4	3	2	0.257	1.101
**87**	C_21_H_22_O_4_	4.667	338.397	4	2	6	2	2	0.178	1.102
**94**	C_21_H_24_O_6_	3.743	372.412	6	1	7	3	2	0.192	1.057
**98**	C_19_H_20_O_3_	4.784	296.36	3	2	6	2	2	0.153	1.100
**99**	C_18_H_18_O_2_	4.8	266.334	2	2	5	2	2	0.14	1.108
**101**	C_22_H_18_O_7_	3.584	394.374	7	0	4	5	3	0.192	0.356
**111**	C_23_H_30_O_5_	4.65	386.481	5	2	4	5	1	0.209	0.486
**127**	C_14_H_12_O_4_	2.466	244.243	4	1	1	3	2	0.235	0.539
**146**	C_21_H_30_O_2_	6.109	314.462	2	1	4	3	1	0.084	0.493
**147**	C_27_H_34_O_5_	3.325	437.548	5	0	4	5	1	0.182	0.412
**188**	C_14_H_16_BrN_3_OS	1.287	355.273	3	2	0	4	2	0.282	0.789
**189**	C_14_H_16_BrN_3_OS	1.287	355.273	3	2	0	4	2	0.282	0.789
**192**	C_21_H_18_BrN_3_O	3.919	408.291	3	2	3	5	3	0.168	0.418
**193**	C_21_H_20_N_4_S	2.122	361.483	3	1	3	5	4	0.167	0.509
**200**	C_15_H_8_N_2_O_2_	2.331	248.236	3	0	0	4	2	0.222	0.670
**211**	C_21_H_17_N_3_O	4.078	327.379	2	1	1	5	3	0.176	0.529
**215**	C_12_H_9_ClN_2_	3.043	216.666	1	0	0	3	3	0.084	0.582
**216**	C_12_H_8_C_l2_N_2_	3.707	251.111	1	0	0	3	3	0.076	0.558
**217**	C_12_H_8_C_l2_N_2_O	2.846	267.111	1	1	0	3	2	0.142	0.578
**227**	C_22_H_32_O_3_	5.507	344.488	3	1	1	4	1	0.101	0.798
**287**	C_29_H_37_NO_5_	4.1	479.608	5	3	2	4	1	0.196	0.679
**291**	C_15_H_19_NO_2_	2.932	245.317	2	2	5	2	2	0.198	0.524
**298**	C_18_H_21_N_3_O_2_S	2.716	343.443	4	1	5	3	2	0.252	0.600
**300**	C_23_H_25_NO_4_	5.22	379.449	4	0	7	3	2	0.149	0.473
**303**	C_19_H_26_ClNO_3_	3.006	350.86	4	1	6	2	1	0.158	0.650
**S88**	C_25_H_27_FN_2_O	3.098	391.501	1	2	5	4	3	0.083	

**Table 2:** Fingerprint similarity between the tested molecules and **S88**.

Comp.	Similarity	SA	SB	SC
**S88**	1.000	565	0	0
Brevicollin (**28**)	0.614	304	−70	261
Cryptopleurine (**41**)	0.642	401	60	164
Columbamine (**46**)	0.605	353	18	212
Palmatine (**47**)	0.584	363	57	202
Glycyrrhizoflavone (**76**)	0.561	329	21	236
Licochalcone A (**87**)	0.645	354	−16	211
Arctigenin (**94**)	0.591	355	36	210
Termilignan (**98**)	0.635	343	−25	222
Anolignan B (**99**)	0.615	346	−2	219
4,5-dihydroxy-6″-deoxybromotopsentin (**192**)	0.720	394	−18	171
Dercitin (**193**)	0.621	357	10	208
Tryptanthrin (**200**)	0.633	337	−33	228
6-Cyano-5-methoxy-12-methylindolo [2, 3A] carbazole (**211**)	0.594	329	−11	236
Thiangazole (**298**)	0.580	307	−36	258
Phenoxan (**300**)	0.574	354	52	211

**SA:** The number of bits in **S88** and target compound, **SB:** The number of bits in target compound but not **S88**, **SC**: The number bits in **S88** but not the target.

**Table 3:** Binding free energies (calculated ΔG in Kcal/mol) of the examined compounds and **S88** as a reference compound against PLpro.

Comp.	ΔG [Kcal/mol]	Comp.	ΔG [Kcal/mol]
**28**	−40.44	**99**	−39.43
**41**	−47.34	**192**	−30.85
**46**	−44.13	**193**	−44.02
**47**	−46.06	**200**	−41.31
**76**	−51.63	**211**	−37.33
**87**	−35.48	**298**	−48.46
**94**	−50.82	**300**	−33.61
**98**	−52.21	**S88**	−59.13

**Table 4:** Predicted ADMET descriptors for the examined molecules and remdesivir.

Comp.	BBB ^a^	HIA ^b^	Aq ^c^	CYP2D6 ^d^	Hepatotoxicity Probability ^e^	PPB ^f^
**28**	c	a	d	n	0.298	c
**41**	b	a	c	i	0.39	b
**46**	b	a	c	i	0.907	a
**47**	b	a	c	i	0.966	c
**76**	e	a	c	i	0.894	b
**87**	b	a	c	n	0.735	b
**94**	c	a	c	n	0.774	c
**98**	b	a	c	n	0.834	c
**99**	b	a	c	n	0.847	c
**192**	b	a	c	n	0.152	c
**193**	b	a	c	i	0.814	c
**200**	c	a	c	n	0.98	c
**211**	b	a	b	i	0.874	c
**298**	c	a	c	n	0.549	c
**300**	b	a	c	n	0.622	c
Remdesivir	e	d	d	n	1.777	b

^a^ BBB level, b is high, c is medium, d is low, e is very low. ^b^ HIA, a is good, b is moderate, c is poor, d is very poor. ^c^ Aq. solubility level, a is extremely low, b is very low, c is low, d is good, e is optimal. ^d^ CYP2D6, n is a non-inhibitor, i is an inhibitor. ^e^ Hepatotoxicity, if >0.5 is toxic, if <0.5 is non-toxic. ^f^ PPBb is >90%, c is >95%.

**Table 5:** Toxicity properties of tested molecules and remdesivir.

Comp.	FDA * Rat Carcinogenicity	TD_50_ (Rat) mg/kg Body Weight/Day	MTD *	LD_50_ *	LOAEL *	Ocular Irritancy ***	Skin Irritancy ***
**28**	s	9.571	0.050	0.939	0.077	m	m
**41**	n	0.219	0.042	0.202	0.018	m	n
**46**	n	0.730	0.081	1.248	0.009	m	n
**47**	n	0.169	0.035	1.446	0.008	m	n
**76**	n	19.216	0.153	0.362	0.150	m	n
**87**	n	48.173	0.113	0.364	0.030	n	n
**94**	n	8.907	0.091	9.209	0.107	m	m
**98**	n	35.370	0.103	1.133	0.398	n	n
**99**	m	69.077	0.240	2.040	0.301	m	n
**192**	n	0.857	1.099	0.348	0.016	m	n
**193**	s	1.587	0.012	0.352	0.048	m	m
**200**	s	7.568	0.055	0.689	0.277	m	n
**211**	s	0.604	0.013	0.245	0.001	m	m
**298**	n	65.542	0.018	0.118	0.019	m	n
**300**	s	13.502	0.029	0.405	0.029	n	m
Remdesivir	n	1.012	0.235	0.309	0.003	m	m

* s is single-carcinogen, m is multi-carcinogen n is non-carcinogen. *** n is nonirritant, m is mild irritant.

**Table 6:** Frontier molecular orbital of **76**, **94**, **298**, and **S88**.

Comp.	Total Energy (Ha)	Binding Energy (Ha)	HOMO Energy (Ha)	LUMO Energy (Ha)	Dipole Mag	Band Gap Energy (Ha)
**76**	−1252.956	−9.601	−0.166	−0.070	1.700	0.096
**94**	−1255.298	−10.037	−0.177	−0.036	3.582	0.141
**298**	−1401.286	−8.702	−0.195	−0.064	1.094	0.131
**S88**	−1242.952	−11.181	−0.292	−0.192	3.621	0.101

## Data Availability

All data are enclosed in the manuscript and supplementary data.

## Supplementary Materials

The following supporting information can be downloaded at: https://www.mdpi.com/article/10.3390/life12091407/s1. Chemical structures, names, molecular formulas of the examined compounds, detailed methodology and toxicity reports.
