# Supplementary material for: Discovery of Potential SARS-CoV-2 Papain-like Protease Natural Inhibitors Employing a Multi-Phase In Silico Approach
Source: Life (Basel). 2022 Sep 9;12(9):1407. doi: 10.3390/life12091407 (PMC9505301; doi:10.3390/life12091407)
Supplement: Supplementary file 1 [file life-12-01407-s001.zip › life-1884367-supplementary.pdf]

Supporting data

Discovery of Potential SARS-CoV-2 Papain-Like Protease Natural Inhibitors Employing a Multi-phase in silico Approach

Eslam B. Elkaeed*[a], Ahmed M. Metwaly[b,c], Mohamed S. Alesawy [d], Abdelrahman M. Saleh [d], Aisha A. Alsfouk[e], Ibrahim. H. Eissa[d]*

[a] Department of Pharmaceutical Sciences, College of Pharmacy, AlMaarefa University, Riyadh 13713, Saudi Arabia

[b] Pharmacognosy and Medicinal Plants Department, Faculty of Pharmacy (Boys), Al-Azhar University, Cairo 11884, Egypt.

[c] Biopharmaceutical Products Research Department, Genetic Engineering and Biotechnology Research Institute, City of Scientific Research and Technological Applications (SRTA-City), Alexandria 21934, Egypt

[d] Pharmaceutical Medicinal Chemistry & Drug Design Department, Faculty of Pharmacy (Boys), Al-Azhar University, Cairo11884, Egypt.

[e] Department of Pharmaceutical Sciences, College of Pharmacy, Princess Nourah bint Abdulrahman University, P.O. Box 84428, Riyadh 11671, Saudi Arabia

Content

Chemical structures, Names and Formulas of the examined natural antiviral compounds	
Method	Molecular Similarity
	Molecular Fingerprints
	Docking studies
	ADMET studies
	Toxicity studies
	DFT studies
Toxicity report	

Chemical Structures

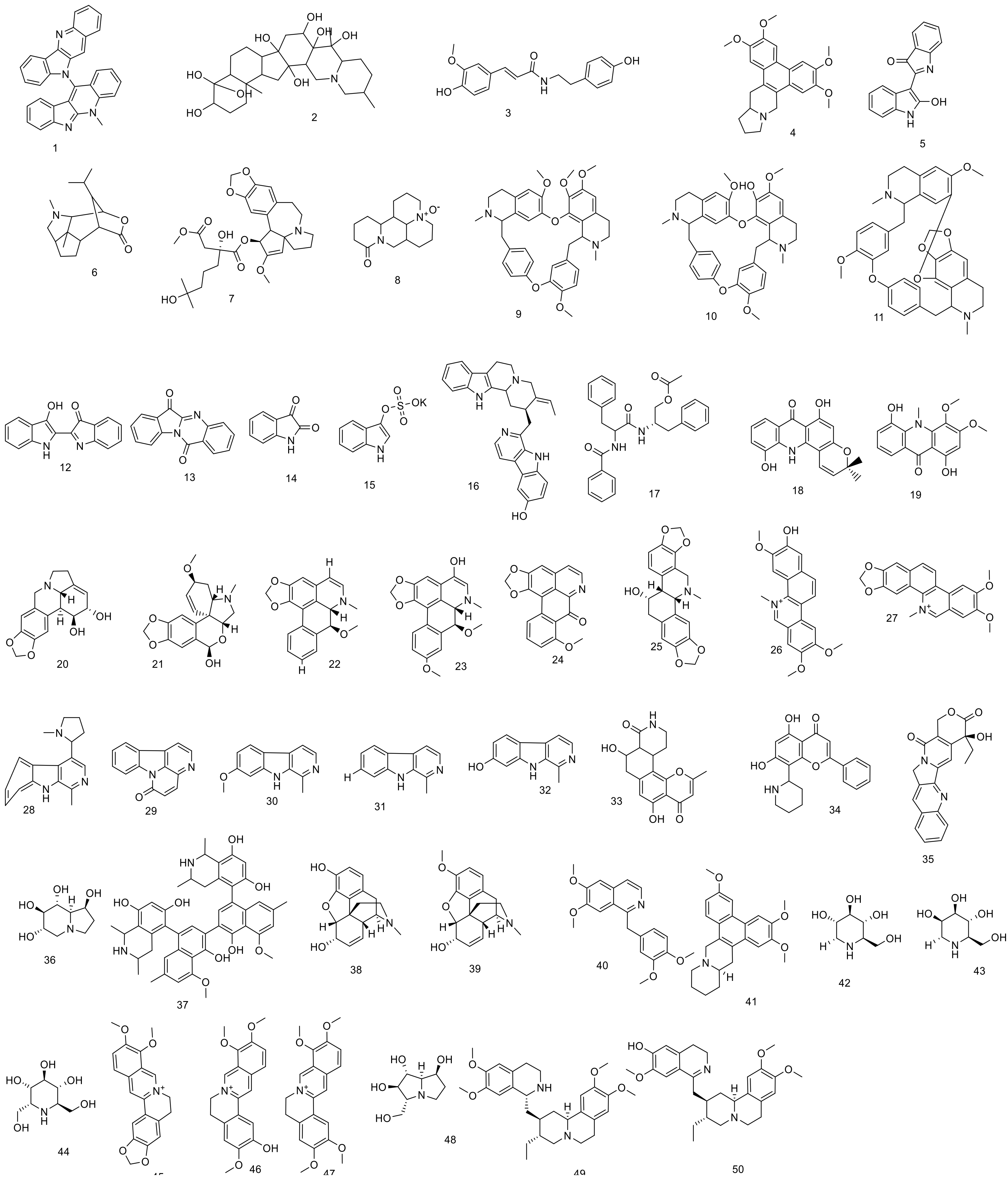

Figure S-1. Chemical structures of the examined 310 natural antiviral compounds

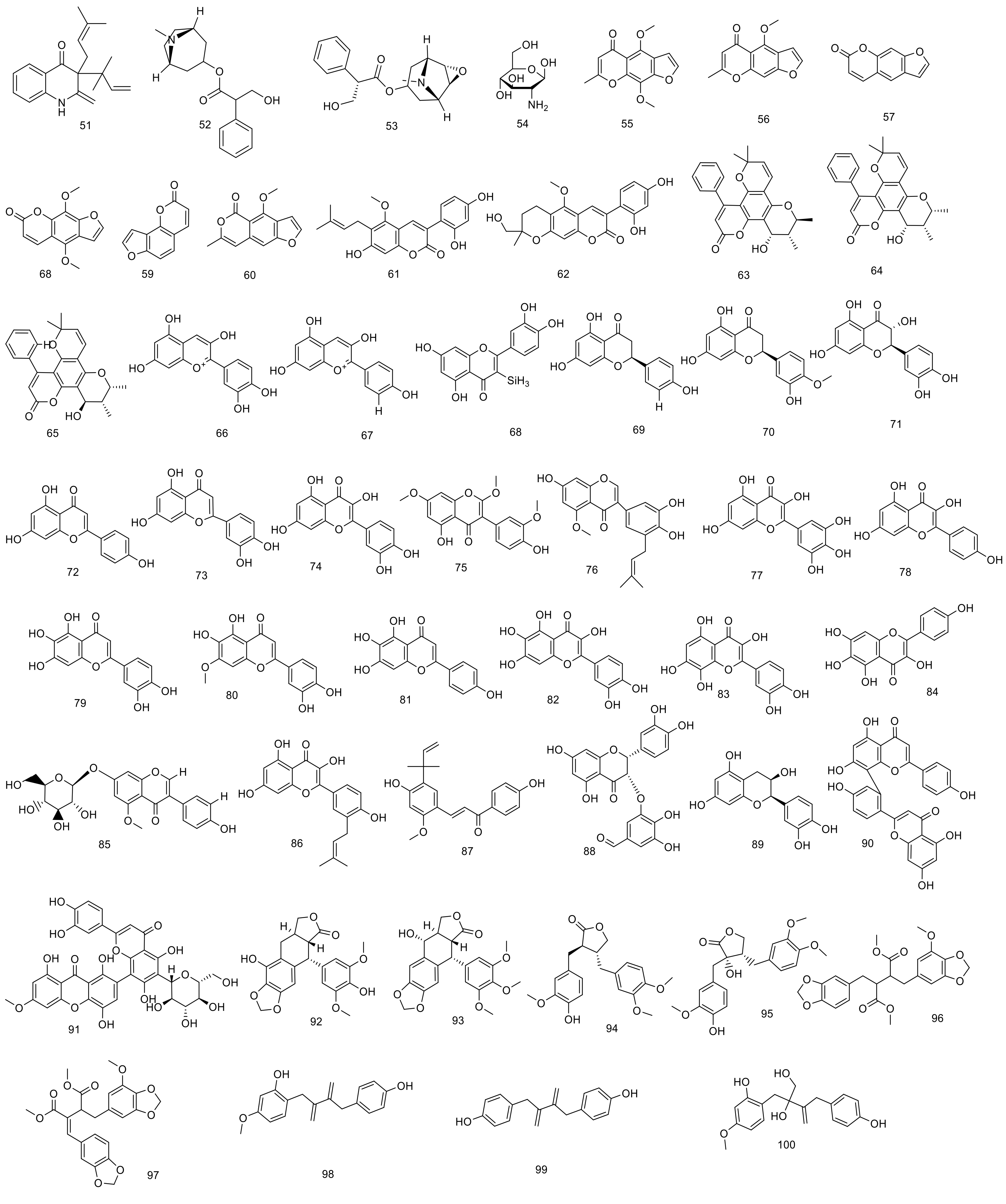

Figure S-1. Chemical structures of the examined 310 natural antiviral compounds

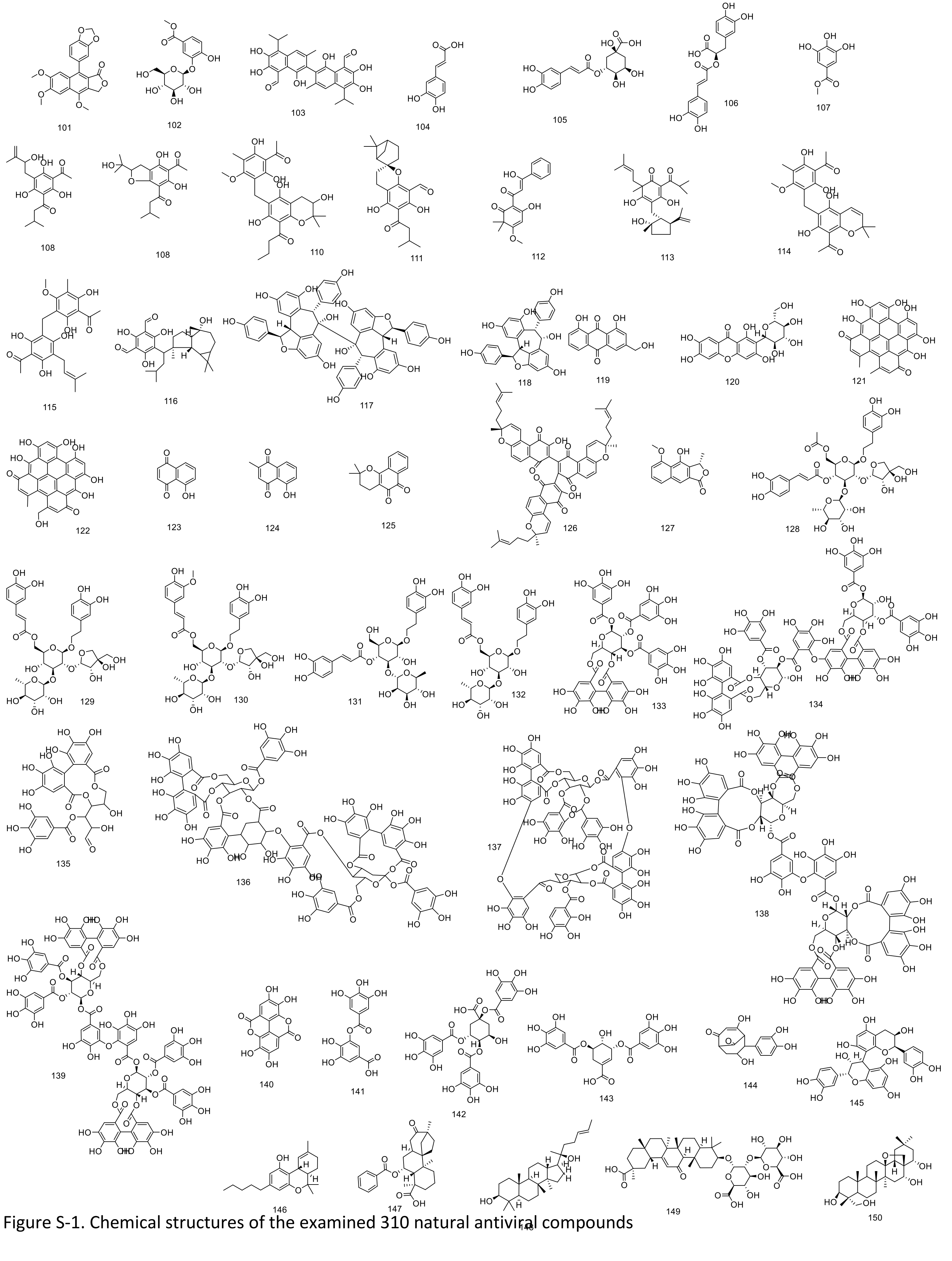

Figure S-1. Chemical structures of the examined 310 natural antiviral compounds

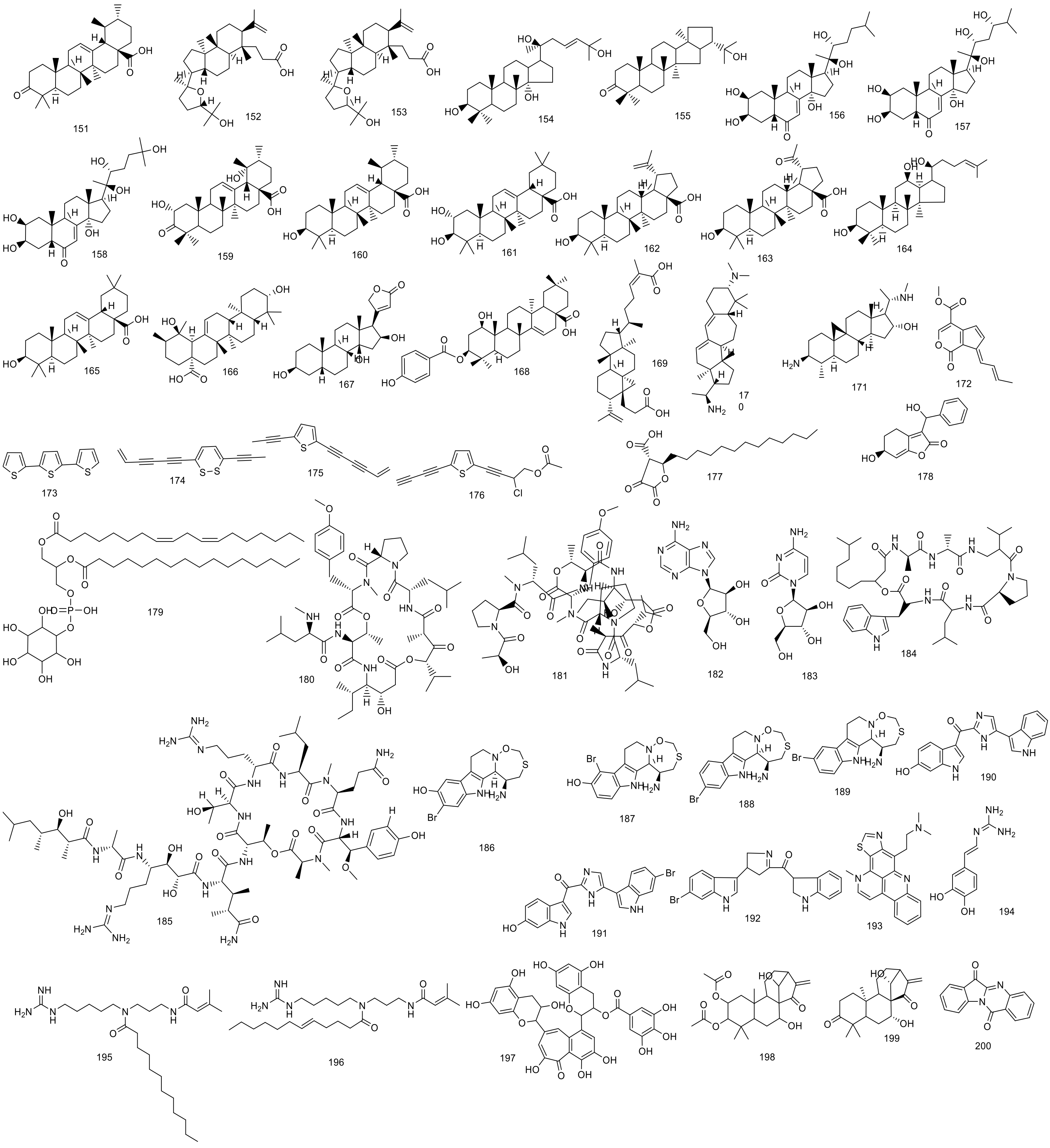

Figure S-1. Chemical structures of the examined 310 natural antiviral compounds

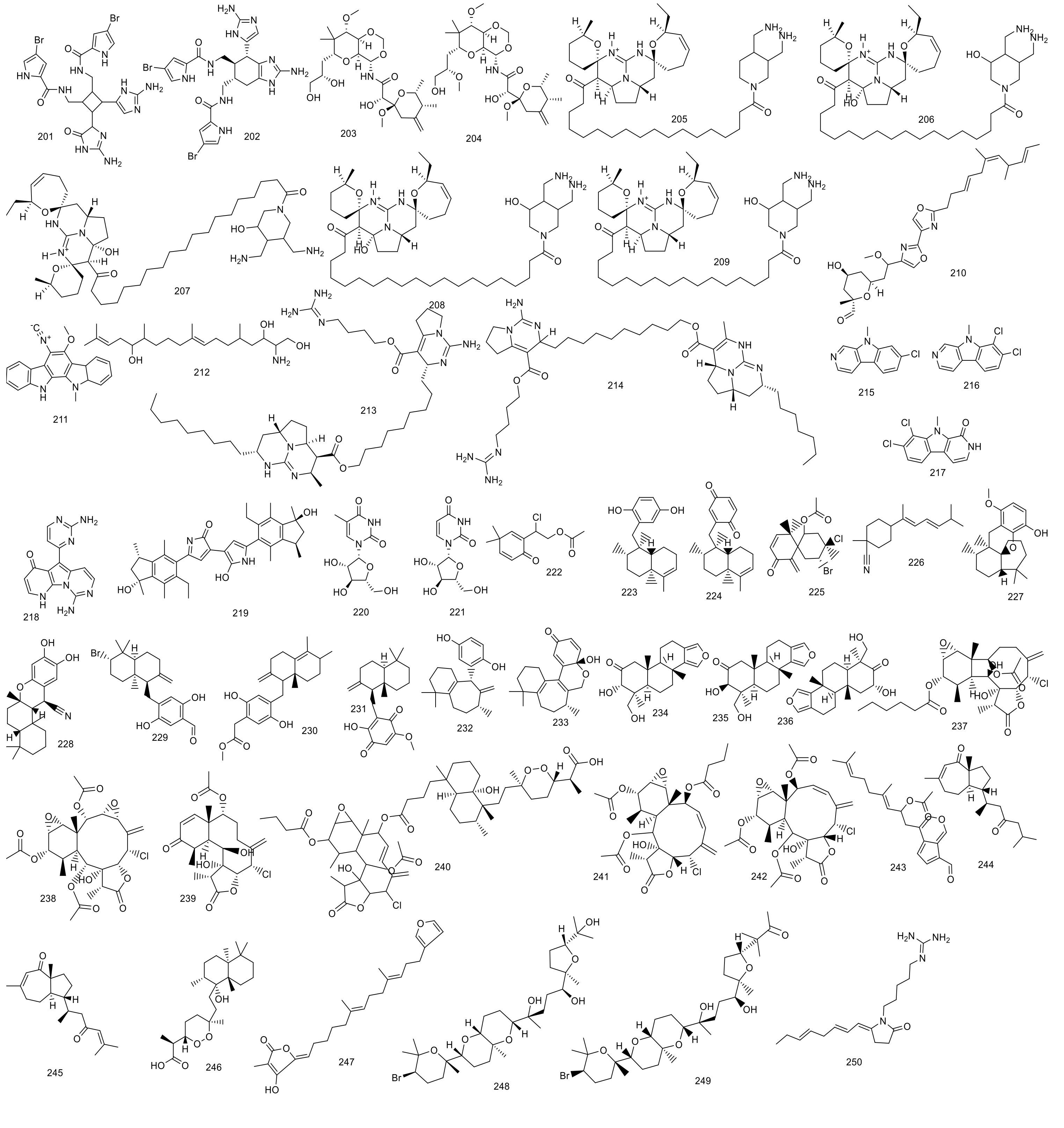

Figure S-1. Chemical structures of the examined 310 natural antiviral compounds

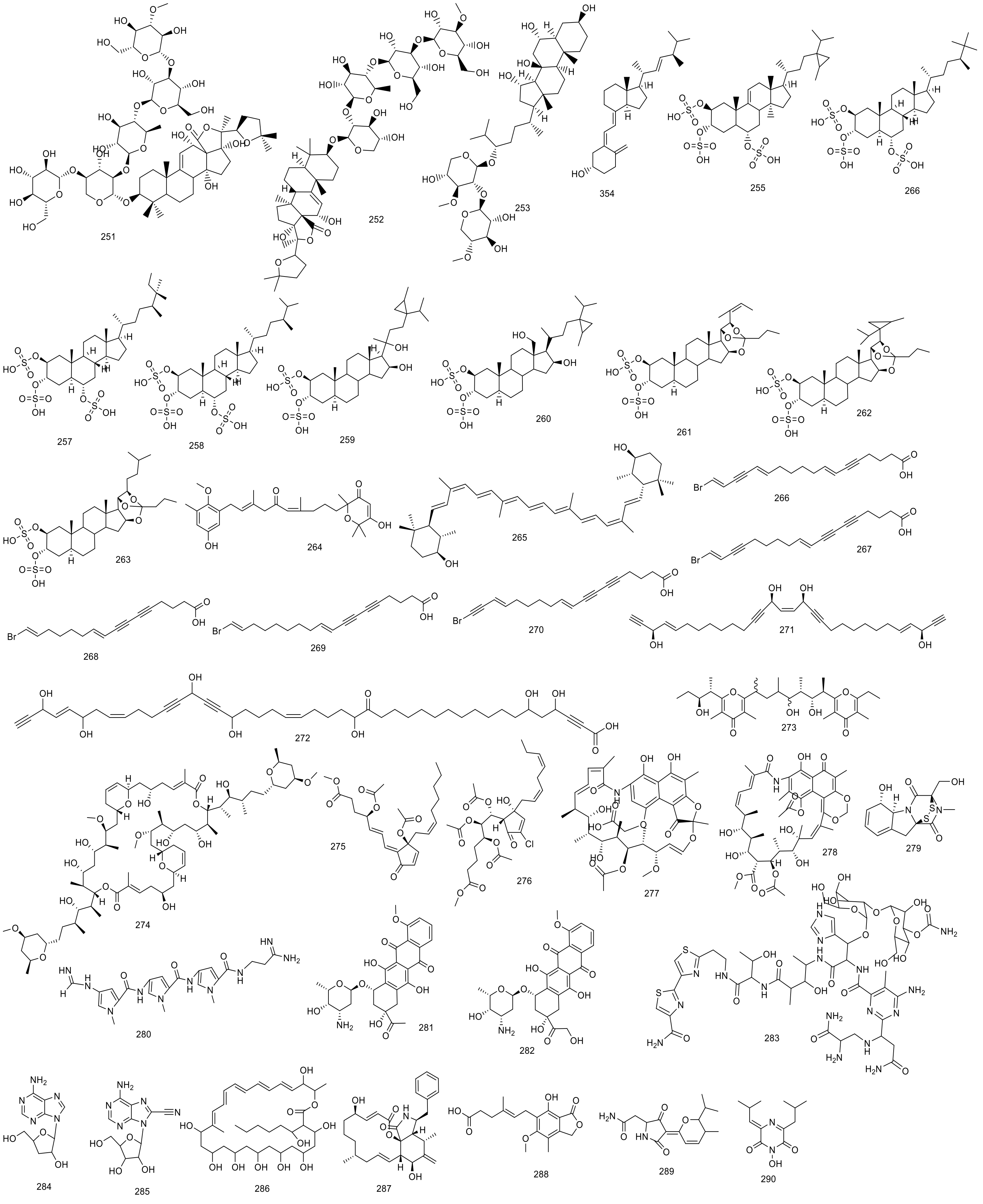

Figure S-1. Chemical structures of the examined 310 natural antiviral compounds

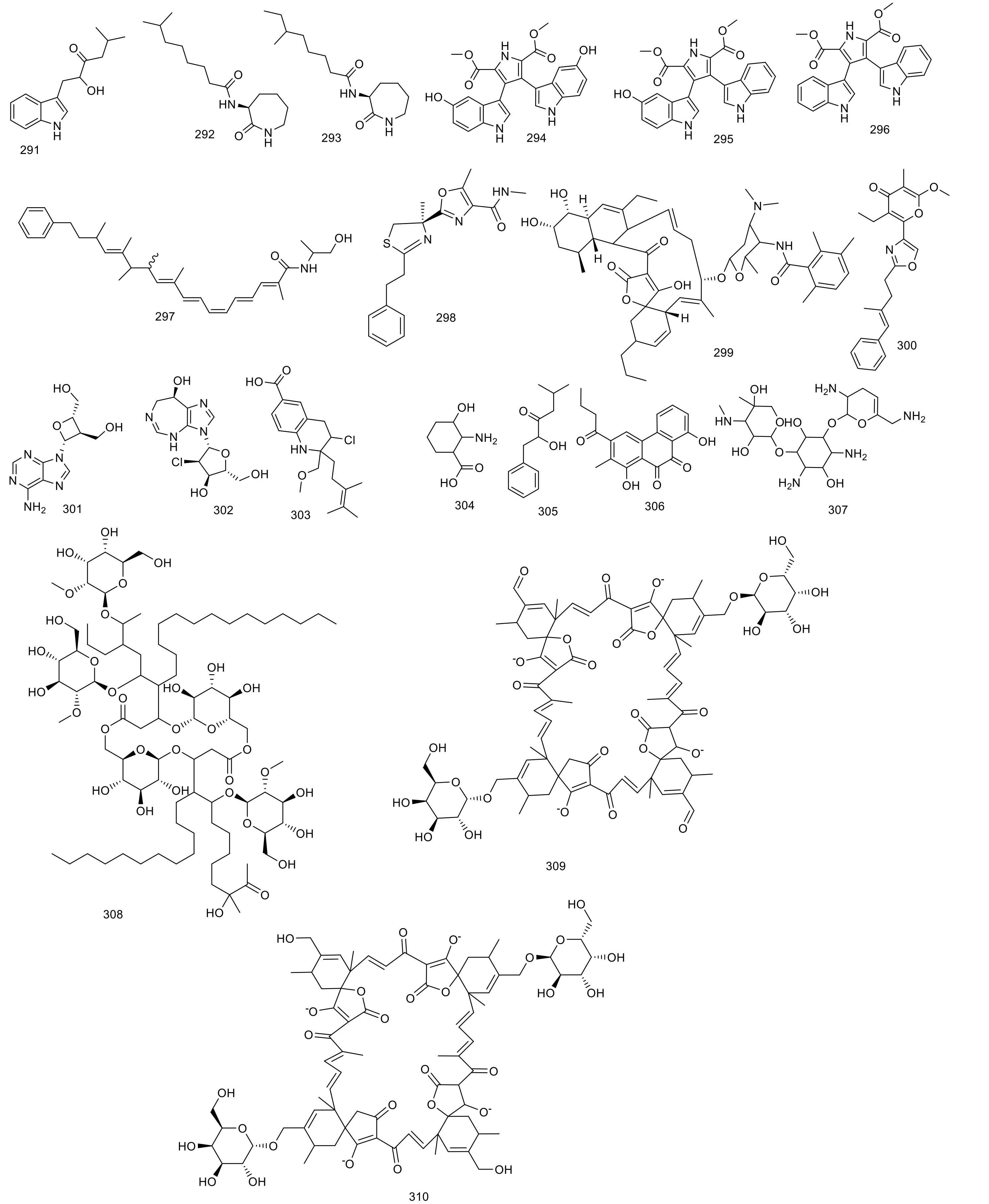

Figure S-1. Chemical structures of the examined 310 natural antiviral compounds

Common names and molecular formulas of the tested compounds

NO.	molecular formula	Name
1	$C_{31}H_{20}N_4$	Cryptoquindoline
2	$C_{27}H_{45}NO_8$	Cevine
3	$C_{18}H_{19}NO_4$	Moupinamide
4	$C_{24}H_{27}NO_4$	Tylophorine
5	$C_{16}H_{10}N_2O_2$	Indirubin
6	$C_{16}H_{25}NO_2$	Dendrobine
7	$C_{29}H_{39}NO_9$	Homoharringtonine
8	$C_{15}H_{24}N_2O_2$	Oxysophoridine
9	$C_{38}H_{42}N_2O_6$	Tetrandrine
10	$C_{37}H_{40}N_2O_6$	Fangchinoline
11	$C_{37}H_{38}N_2O_6$	Cepharanthine
12	$C_{16}H_{10}N_2O_2$	Indigo
13	$C_{15}H_8N_2O_2$	Tryptanthrin
14	$C_8H_5NO_2$	Isatin
15	$C_8H_6KNO_4S$	Indican
16	$C_{29}H_{28}N_4O$	10-hydroxyusambarensine
17	$C_{27}H_{28}N_2O_4$	Aurantiamide acetate
18	$C_{18}H_{15}NO_4$	Atalaphillidine
19	$C_{16}H_{15}NO_5$	Citrusinine i
20	$C_{16}H_{17}NO_4$	Lycorine
21	$C_{18}H_{21}NO_5$	Pretazettine
22	$C_{19}H_{17}NO_3$	Oliverine
23	$C_{20}H_{19}NO_5$	Pachystaudine
24	$C_{18}H_{11}NO_4$	Oxostephanine
25	$C_{20}H_{19}NO_5$	Chelidonine
26	$C_{21}H_{20}NO_4$	Fagaronine
27	$C_{21}H_{18}NO_4$	Nitidine
28	$C_{17}H_{19}N_3$	Brevicollin

29	C14H8N2O	6-canthinone
30	C13H12N2O	Harmine
31	C12H10N2	Harmane
32	C12H10N2O	Harmol
33	C17H17NO5	Schumannificine
34	C20H19NO4	O-demethyl-buchenavianine
35	C20H16N2O4	Camptothecin -10-ome-camptothecin
36	C8H15NO4	Castanospermine - alexine
37	C46H48N2O8	Michellamincs a
38	C17H19NO3	Morphine
39	C18H21NO3	Codeine
40	C20H21NO4	Papaverine
41	C24H27NO3	Cryptopleurine
42	C6H13NO4	1-deoxynojirimycin
43	C6H13NO4	1-deoxymannojirimycin
44	C7H15NO5	A-homonojirimycin
45	C20H18NO4	Berberine
46	C20H20NO4	Columbamine
47	C21H22NO4	Palmatine
48	C8H15NO4	Australine
49	C29H40N2O4	Emetine
50	C28H36N2O4	Sychotrine
51	C20H25NO	Buchapine
52	C17H23NO3	Atropine
53	C17H21NO4	Scopolamine
54	C6H13NO5	Glucosamine
55	C14H12O5	Khellin
56	C13H10O4	Visnagin
57	C11H6O3	Psoralen
58	C13H10O5	Sopimpinellin

59	C11H6O3	Angelicin
60	C13H10O4	Coriandrin
61	C21H20O6	Glycycoumarin
62	C21H20O7	Licopyranocoumarin
63	C25H24O5	Soulattrolide
64	C25H24O5	Inophyllums a
65	C25H24O5	Inophyllums b
66	C15H11O6	Cyanidin
67	C15H11O5	Pelargodin
68	C15H12O6Si	Catechin
69	C15H12O5	Naringin
70	C16H14O6	Hesperetin
71	C15H12O7	Taxifolin
72	C15H10O5	Apigenin
73	C15H10O6	Luteolin
74	C15H10O7	Quercetin
75	C18H16O7	Quercetagistrin
76	C21H20O6	Glycyrrhizoflavone
77	C15H10O8	Myricetin
78	C15H10O6	Kaempfero l
79	C15H10O7	6-hydroxyluteolin
80	C16H12O7	Pedalitin
81	C15H10O6	Scutellarein
82	C15H10O8	Quercetagetin
83	C15H10O8	Gossypetin
84	C15H10O7	6-hydroxykaempferol
85	C22H22O10	5-0-methylgenistein-7-glucoside
86	C20H18O6	Isolicoflavonol
87	C21H22O4	Licochalcone a
88	C22H16O10	Epicatchin-3-0-gallate

89	C15H14O6	Epicatchin
90	C30H18O10	Amentoflavone
91	C35H28O17	Swertifrancheside
92	C21H20O8	A-peltatin
93	C22H22O8	Podophyllotoxin
94	C21H24O6	Arctigenin
95	C21H24O7	Trachelogenin
96	C23H24O9	3-0 -demethylarcetagenin
97	C23H22O9	Ks-7-rhinacanthins e
98	C19H20O3	Termilignan
99	C18H18O2	Anolignan
100	C19H22O5	Thannilignan
101	C22H18O7	Justicidin a
102	C14H18O9	Woodorien
103	C30H30O8	Gossypoi
104	C9H8O4	Caffeic acid
105	C16H18O9	Chlorogenic acid
106	C18H16O8	Rosmarinic acid
107	C8H8O5	Methylgaltate
108	C18H24O6	Sessiliflorene
109	C18H24O6	Sessiliflorol
110	C26H32O9	Butyrylmallotochromanol
111	C23H30O5	Euglobal g3
112	C18H18O5	Syzygiol
113	C26H38O5	Chinesin ii
114	C24H26O8	Mallotochromene
115	C24H28O8	Mallotojaponin
116	C28H40O6	Macrocarpal a
117	C56H42O14	Dibalanocarpol
118	C28H22O7	Balanocarpol

119	C15H10O5	Aloe-emodin
120	C19H18O11	Mangiferin
121	C30H16O8	Hypericin
122	C30H16O9	Seudohypericin
123	C10H6O3	Juglone
124	C11H8O3	Plumbagin
125	C15H14O3	P-lapachone
126	C60H56O11	Conocurvone
127	C14H12O4	Isoeleutherin
128	C36H46O20	Luteosides a
129	C34H44O19	Luteosides b
130	C35H46O19	Luteosides c
131	C29H36O15	Verbascoside
132	C29H36O15	Isoverbascoside
133	C41H30O26	Eugeniin
134	C68H50O44	Oenothein b
135	C27H22O18	Gemin d
136	C82H62O52	Nobotanin b
137	C75H52O46	Camellin b
138	C82H54O52	Agrimoniin
139	C82H58O52	Coriarin a
140	C14H6O8	Octagalloylglucoses
141	C14H10O9	Octagalloylglucoses ellagic acid
142	C28H24O18	1,3,4-tri-o-gailoylquinic acid
143	C21H18O13	3.5-di- o -galloylshikimic acid
144	C15H14O6	Catgghinic acid
145	C30H26O12	Procyanidin b2
146	C21H30O2	A^-tetrahydrocannabinol
147	C27H34O5	Scopadulcic acid b
148	C29H50O2	Dammarenediol

149	C42H62O16	Glycyrrhizin
150	C30H50O5	Ursonic acid
151	C30H46O3	Protoprimulagenin
152	C30H50O4	Eichlerianic acid
153	C30H50O4	Shoreic acid
154	C29H50O4	Sofouquierol
155	C29H48O2	22-hydroxyhopanone
156	C27H44O6	Ponasterone a
157	C27H44O7	Pterosterone
158	C27H44O7	Ecdysterone
159	C30H46O5	2a,19a-dihydroxy-3-oxo-12-ursen-28-oic aci d
160	C30H48O3	Ursoli c acid
161	C30H48O4	Maslini c acid
162	C30H48O3	Betulinic acid
163	C29H46O4	Platanic acid
164	C29H50O3	Chikusetsusaponin iii
165	C30H48O3	Oleanolic acid
166	C30H48O4	Pomolic acid
167	C23H34O5	Digitoxin
168	C37H52O6	3-p- hydroxybenzoate
169	C30H46O4	Nigranoic acid
170	C26H44N2	Buxamine e
171	C24H42N2O	Cyclobuxamine h
172	C14H12O4	Fulvoplumerien
173	C12H8S3	Alpha-terthienyl
174	C13H8S2	Thiarubrine a
175	C13H8S	Thiophene-a
176	C14H9ClO2S	Acbp-thiophene
177	C18H30O5	Protolichesterinic acid
178	C15H14O4	Cochinolide

179	C43H79O13P	Phosphatidyl inositol
180	C49H78N6O12	Didemnin a
181	C57H89N7O15	Didemnin b
182	C10H13N5O4	9β-D-arabinofuranosyladenin
183	C9H13N3O5	l-|β-D-arabinosyl-cytosine
184	C45H69N7O8	Kahalalide e
185	C68H116N18O20	Callipeltin a
186	C14H16BrN3O2S	Eudistomin c
187	C14H16BrN3O2S	Eudistomin e
188	C14H16BrN3OS	Eudistomin k
189	C14H16BrN3OS	Eudistomin l
190	C20H14N4O2	Topsentin
191	C20H13BrN4O2	Bromotopsentin
192	C21H18BrN3O	4,5-dihydroxy-6''-deoxybromotopsentin
193	C21H20N4S	Dercitin
194	C9H11N3O2	Tubastrine
195	C26H51N5O2	Acarnidine a
196	C26H49N5O2	Acarnidine b
197	C24H34O7	Isotheaflavin 3'-gallate
198	C36H28O16	Leukamenin B
199	C20H28O4	Glaucocalyxin A
200	C15H8N2O2	Tryptanthrin
201	C22H24Br2N10O3	Oxysceptri n
202	C22H24Br2N10O2	Ageliferin
203	C24H41NO10	Mycalamide a
204	C25H43NO10	Mycalamide b
205	C46H81N6O4	Ptilomycalin a
206	C46H81N6O6	Crambesscidins 81 6
207	C47H83N6O6	Crambesscidins 830
208	C48H85N6O6	Crambesscidins 844

209	C46H81N6O5	Crambesscidins 800
210	C29H40N2O6	Hennoxazole a
211	C21H17N3O	6-cyano-5-methoxy-l2-methylindolo[2,3a]carbazole
212	C22H43NO3	Apiidiasphingosine
213	C42H73N9O4	Batzelladine a
214	C41H69N9O4	Batzelladine b
215	C12H9ClN2	Bauerine a
216	C12H8Cl2N2	Bauerine b
217	C12H8Cl2N2O	Bauerine c
218	C14H11N7O	Variolin b
219	C38H46N2O4	Trikendiol
220	C10H14N2O6	Spongothymidine
221	C9H12N2O6	Spongouridine
222	C12H15ClO3	Halogenated cyclohexadien one
223	C21H30O2	Avarol
224	C21H28O2	Avarone
225	C17H22BrClO3	Chamigrene derivative
226	C16H25N	Sesquiterpene isocyanid e
227	C22H32O3	Strongylin a
228	C22H29NO3	15-cyanopuupehenoi
229	C22H29BrO3	Peyssonol a
230	C24H32O4	Peyssonol b
231	C22H30O4	Hyatellaquinone
232	C21H28O2	Frondosin a
233	C21H26O3	Frondosin d
234	C20H28O4	Spongiadiol
235	C20H28O4	Pispongiadiol
236	C20H28O4	Isospongiadiol
237	C28H41ClO9	Solenolide a
238	C26H33ClO11	Solenolide e

239	C22H29ClO7	Solenolide d
240	C53H79ClO15	Brianthein v
241	C28H37ClO10	Brianthein y
242	C26H33ClO10	Brianthein z
243	C22H26O4	Halitunal
244	C20H32O2	Reiswigin a
245	C20H30O2	Reiswigin b
246	C25H44O5	Mycaperoxide b
247	C24H32O4	Variabilin
248	C30H53BrO7	Thyrsiferol
249	C32H55BrO7	Thyrsiferol acetate
250	C18H30N4O	Polyandrocarpldlne A
251	C59H94O30	Holothurinosid e a
252	C54H86O24	Desholothurin a
253	C39H68O13	Crossasteroids b
254	C28H44O	Calciferol d
255	C31H52O12S3	Ibisterol trisodium sulfate
256	C29H52O12S3	Alistano l trisodium sulfate
257	C30H54O12S3	26-methylhalistanol trisodium sulfat e
258	C28H50O12S3	25-demethylhalistanol trisodium sulfate
259	C30H52O10S2	Weinbersterols disodium sulfate a
260	C30H52O10S2	Weinbersterols disodium sulfate b
261	C29H46O11S2	Orthosterol disodium sulfates a
262	C32H52O11S2	Orthosterol disodium sulfates b
263	C30H50O11S2	Orthosterol disodium sulfates c
264	C28H38O6	Usneoidol z
265	C40H60O2	Cucumariaxanthin c
266	C18H21BrO2	18-bromo.(7£, 13£ , 17£) -octadeca-7,13, l7-triene -5,15-diynoic acid
267	C18H19BrO2	18-bromo-(9£:, 17£) -octadeca-9,17-diene-5,7,15-triynoic acid

268	C16H19BrO2	16-bromo-(9£,15£)-hexadeca-9, 15-diene-5,13 -diynoic acid
269	C18H23BrO2	18-bromo-(9£, he)-octadeca-9,17-diene-5,7-diynoic acid
270	C18H19BrO2	18-bromo-(9£:, 15£) -octadeca-9,15-diene-5,7,15-triynoic acid
271	C30H40O4	Petrosynol
272	C44H64O10	Petrosolic acid
273	C32H50O7	Onchitriol 1
274	C74H128O20	Misakinolide a
275	C25H34O7	Clavulone ii
276	C27H37ClO10	Punaglandin-1
277	C39H49NO14	Rifamycin b
278	C42H53NO15	Streptovaricin b
279	C13H14N2O4S2	Gliotoxin
280	C22H28N10O3	Distamycin a
281	C27H29NO10	Daunomycin
282	C27H29NO11	Doxorubicin
283	C50H73N17O21S2	Bleomycin a
284	C10H13N5O3	Cordyceptin
285	C11H12N6O4	Toyocamycin
286	C35H58O11	Filipin
287	C29H37NO5	Cytochalasin b
288	C17H20O6	*Mycophenolic acid*
289	C15H20N2O4	Briodionen
290	C12H18N2O3	Flutimide
291	C15H19NO2	Sattazolin
292	C15H28N2O2	Caprolactin a
293	C15H28N2O2	Caprolactin b
294	C24H19N3O6	Lycogarubin a
295	C24H19N3O5	Lycogarubin b
296	C24H19N3O4	Lycogarubin c
297	C33H47NO2	Fenalamide i

298	C18H21N3O2S	Thiangazole
299	C51H70N2O9	Mm46115
300	C23H25NO4	Phenoxan
301	C10H13N5O3	Pumilacidi n a
302	C11H15ClN4O4	Adechlorin
303	C19H26ClNO3	Virantmycin
304	C7H13NO3	Sf.1836 c
305	C13H18O2	Sattabaci n
306	C19H16O5	Sch 68631
307	C19H37N5O8	Mutactimycin a
308	C85H154O34	Cycloviracins bl
309	C79H88O29	Quatromicins a l
310	C79H92O29	Quatromicins a 3

Method

- **Preparation of the tested compounds for Molecular Similarity, fingerprints, ADMET, toxicity and DFT studies:**

In this protocol, the general-purpose panel was utilized with the activation of the Prepare ligand option. The change ionization was switched on the true option using the Rule based as an ionization method. In Rule based task, we used the carboxylate as an acid ionization. Additionally, the primary, secondary, and tertiary amines were selected as Base ionization. The ionization enumeration option was switched on the one protomer. Under the filter smart option, we selected all options. The false option was selected for tasks Generate tautomers, generate isomers, Fix bad valencies, and parallel processing. The generate coordinates task was switched on the 3D option. Finally, the duplicate structure task was activated on the remove option.

- **Molecular Similarity**

The molecular Similarity of the tested compounds were checked using Discovery studio 4.0. **S88** was used as a reference molecule. The study calculates the following parameters in a quantitative method

1. Number of rotatable bonds,
2. Number of rings,
3. Number of aromatic rings,
4. Number of hydrogen bond donors,
5. Number of hydrogen bond acceptors,
6. Octanol-water partition coefficient (ALog p),
7. Molecular weight, and
8. Molecular fractional polar surface area (MFPSA).

Running of Molecular Similarity protocol

In this protocol, after compounds preparation, the small molecules panel was utilized with the activation of the library analysis option. Then, the option of find similar molecules by numeric properties was activated. Furthermore, we selected the prepared compounds as the input ligands and S88 as a reference ligand. In addition, what to find task was switched on as most similar molecules (number of similar molecules = 30). The Euclidean distance was selected. Then, the output of the running protocol was visualized to give the output chart of structural similarity.

- **Fingerprints check**

The Fingerprints check of the tested compounds were checked using Discovery studio 4.0. **S88** was used as a reference molecule. The used fingerprints were based on the presence or absence of some parameters related to type of atoms, in detail,

1. Number of charges,
2. Hybridization,
3. Number of hydrogen bond acceptors,
4. Number of hydrogen bond donors,
5. Number of positive ionizable atoms,
6. Number of negative ionizable atoms,
7. Number of halogen atoms,
8. Number of aromatic rings
9. ALogP (octanol-water partition coefficient)

Running of Fingerprints check protocol

In this protocol, after compounds preparation, the small molecules panel was utilized with the activation of the library analysis option. Then, the option of find similar molecules by Fingerprints was activated. Furthermore, we selected the prepared compounds as the input ligands and S88 as a reference ligand. In addition, what to find task was switched on as most similar molecules (number of similar molecules = 15). The Tanimoto coefficient was selected. Then, the output of the running protocol was visualized to give the output results.

- **Molecular docking**

Protein Preparation:

The crystal structure of SARS papain-like protease (PLpro) (PDB ID: 4OW0, resolution: 2.10 Å was obtained from Protein Data Bank (https://www.rcsb.org). At first, the crystal structure of the PLpro complexed with the S88 as a co-crystallized ligand was prepared by removing crystallographic water molecules. Only one chain was retained besides the co-crystallized ligand (S88). The selected protein chain was protonated using the following setting. The used electrostatic

functional form was GB/VI with a distance cut-off of 15 Å. The used value of the dielectric constant was 2 with an 80 dielectric constant of the used solvent. The used Van der Waals functional form was 800R3 with a distance cut-off of 10 Å. Then, the energy of the protein chain was minimized using Hamiltonian AM1 implanted in Molecular Operating Environment (MOE 2019 and MMFF94x (Merck molecular force field) for structural optimization. Next, the active site of the target protein was defined for ligand docking and redocking (in case of validation of docking protocol). The active site of the protein was identified as the residues that fall within the 5 Å distance from the perimeter of the co-crystallized ligand.

Ligand Preparation: 2D structures of the synthesized compounds and the standard compound, sorafenib were drawn using ChemBioDraw Ultra 14.0 and saved in MDL-SD file format. The 3D structures of the ligands were protonated, and the structures were optimized by energy minimization using MM2 force-field and 10000 iteration steps of 2 fs. The conformationally optimized ligands were used for docking studies.

Docking Setup and Validation of Docking Protocol: The protein-ligand docking studies were carried out using MOE version 2019. Validation of the docking protocol was carried out by redocking the co-crystallized reference ligand (S88) against the isolated pocket of PLpro. The docking protocol was validated by comparing the heavy atoms RMSD value of the re-docked ligand pose with the corresponding co-crystallized reference ligand structure.

The docking setup for the tested compounds was established according to the protocol followed in the validation step. For each docking run, 30 docked solutions were generated using ASE for scoring function and rigid receptor for refinement. The pose with ideal binding mode was selected for further investigations. The docking results were visualized using Discovery Studio (DS) 4.0. Analysis of the docking results was carried out by comparing the interactions and docking score obtained for the docked ligands with that of the re-docked reference molecule (S88).

- **ADMET studies**

ADMET descriptors (absorption, distribution, metabolism, excretion and toxicity) of the compounds were determined using Discovery studio 4.0. **Remdesivir** was used as a reference molecule. At first, the CHARMM force field was applied then the tested compounds were prepared and minimized according to the preparation of small molecule protocol. The ADMET descriptors that applied including models for

1. Human intestinal absorption,
2. Aqueous solubility,
3. Blood brain barrier penetration,
4. Plasma protein binding,
5. Cytochrome P450 2D6 inhibition, and
6. Hepatotoxicity.

The examined molecules filtered to select those molecules that meet the rules specified by the set of selected SMARTS® rules.

.

Running of ADMET protocol

In this protocol, after compounds preparation, the small molecules panel was utilized with the activation of the ADMET descriptors option. Then, we selected the prepared compounds as the input ligands. Further, all the ADMET parameters (aqueous solubility, Blood brain barrier, intestinal absorption, CYP2D6, and plasma protein binding) were selected. Then, the output of the running protocol was visualized to give the ADMET chart.

- **Toxicity studies**

The toxicity parameters of the synthesized compounds were calculated using Discovery studio 4.0. Remdesivir was used as a reference molecule. Then different parameters were calculated from the toxicity prediction (extensible) protocol (TOPKAT) that evaluated the examined compounds' performance in experimental assays and animal models. TOPKAT computed and validated assessments of the toxic and environmental effects of the examined chemicals solely from their molecular structure. TOPKAT employs robust and cross-validated Quantitative Structure Toxicity Relationship (QSTR) models for assessing various measures of toxicity and utilizing the patented Optimal Predictive Space validation method to assist in interpreting the results.

The predicted models are

1. FDA rat carcinogenicity test,
2. Carcinogenic potentiality TD_{50} (the median toxic dose of a substance in which toxicity occurs in 50% of a species),
3. Maximum tolerated dose (MTD) in rats,

4. Oral LD_{50} in rats (the amount that kills 50% of test animals),
5. Chronic LOAEL (Lowest-observed-adverse-effect level) in rats,
6. Ocular irritancy and
7. Skin irritancy

Running of Toxicity protocol

In this protocol, after compounds preparation, the small molecules panel was utilized with the activation of the toxicity prediction (extensible) option. Then, we selected the prepared compounds as the input ligands. Further, the different toxicity models were selected from the model panel. The similarity search task was activated to be true. The detailed report task was switched on as a PDF file. Then, the output of the running protocol was visualized to give the toxicity PDF report.

- **DFT studies**

The DFT studies of the tested compounds were calculated using Discovery studio 4.0. At first, the CHARMM force field was applied then the compounds were prepared and minimized according to the preparation of small molecule protocol.

Running of DFT protocol

In this protocol, simulation panel was utilized with the activation of the calculate energy option from Tools panel. Then, we selected the prepared compounds as the input ligands the option. The used functional was the PWC of local density approximate (LDA). Additionally, the quality was adapted as Coarse, which utilizes the DN bases set and SCF density converge of 1.0 × 10−4, as employed from Accelrys in the DMl3 module of the Materials Studio package Then, the output of the running protocol was visualized to give the output results.

Toxicity Report

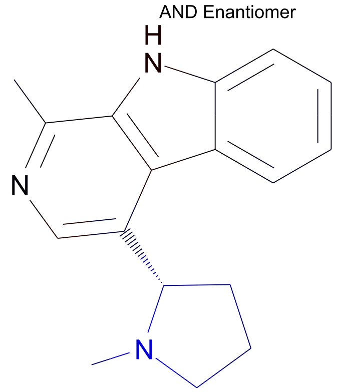

$C_{17}H_{19}N_3$
Molecular Weight: 265.35286
ALogP: 3.018
Rotatable Bonds: 1
Acceptors: 2
Donors: 1

Model Prediction

Prediction: Non-Carcinogen
Probability: 0.22
Enrichment: 0.686
Bayesian Score: -2.99
Mahalanobis Distance: 9.16
Mahalanobis Distance p-value: 0.807

Prediction: Positive if the Bayesian score is above the estimated best cutoff value from minimizing the false positive and false negative rate.
Probability: The esimated probability that the sample is in the positive category. This assumes that the Bayesian score follows a normal distribution and is different from the prediction using a cutoff.
Enrichment: An estimate of enrichment, that is, the increased likelihood (versus random) of this sample being in the category.
Bayesian Score: The standard Laplacian-modified Bayesian score.
Mahalanobis Distance: The Mahalanobis distance (MD) is the distance to the center of the training data. The larger the MD, the less trustworthy the prediction.
Mahalanobis Distance p-value: The p-value gives the fraction of training data with an MD greater than or equal to the one for the given sample, assuming normally distributed data. The smaller the p-value, the less trustworthy the prediciton. For highly non-normal X properties (e.g., fingerprints), the MD p-value is wildly inaccurate.

Structural Similar Compounds

Name	Granisetron	Temazepam	Diazepam
Structure	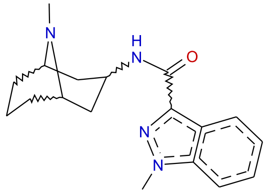	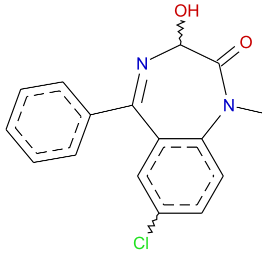	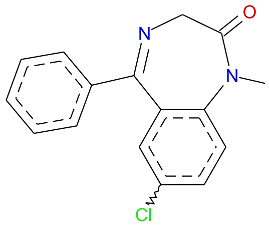
Actual Endpoint	Non-Carcinogen	Non-Carcinogen	Carcinogen
Predicted Endpoint	Non-Carcinogen	Non-Carcinogen	Non-Carcinogen
Distance	0.576	0.586	0.596
Reference	US FDA (Centre for Drug Eval.& Res./Off. Testing & Res.) Sept. 1997	US FDA (Centre for Drug Eval.& Res./Off. Testing & Res.) Sept. 1997	US FDA (Centre for Drug Eval.& Res./Off. Testing & Res.) Sept. 1997

Model Applicability

Unknown features are fingerprint features in the query molecule, but not found or appearing too infrequently in the training set.

1. All properties and OPS components are within expected ranges.

Feature Contribution

Top features for positive contribution

Fingerprint	Bit/Smiles	Feature Structure	Score	Carcinogen in training set
ECFP_6	-1661653144	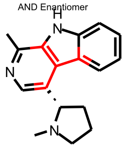 [*][c](:[*]):[c]1:[c](:[*]):[*]:[*]:[c]:1:[*]	0.442	2 out of 3

ECFP_6	1336304100	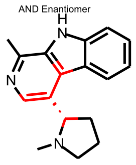 [*]C([*])[c](:[cH]:[*]):[c](:[*]):[*]	0.296	7 out of 16
ECFP_6	-152683720	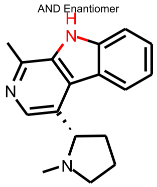 [*]:[nH]:[*]	0.245	7 out of 17

Top Features for negative contribution

Fingerprint	Bit/Smiles	Feature Structure	Score	Carcinogen in training set
ECFP_6	-484970154	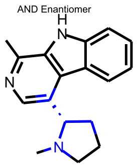 [*]N1[*][*]C[C@H]1[c](:[*]):[*]	-0.805	0 out of 4
ECFP_6	48510090	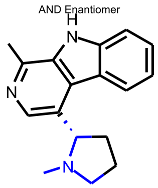 [*][C@@H]1[*][*]CN1C	-0.805	0 out of 4
ECFP_6	1652635785	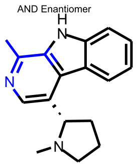 [*]:n:[c](C):[c](:[*]):[*]	-0.482	0 out of 2

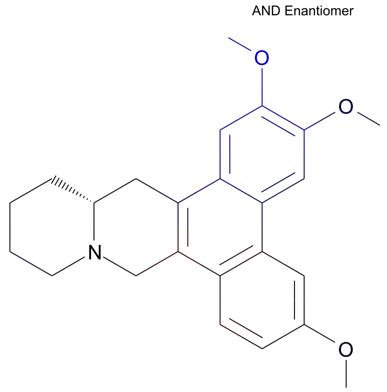

$C_{24}H_{27}NO_3$
Molecular Weight: 377.47608
ALogP: 4.691
Rotatable Bonds: 3
Acceptors: 4
Donors: 0

Model Prediction

Prediction: Non-Carcinogen
Probability: 0.212
Enrichment: 0.66
Bayesian Score: -4.06
Mahalanobis Distance: 10.3
Mahalanobis Distance p-value: 0.334

Prediction: Positive if the Bayesian score is above the estimated best cutoff value from minimizing the false positive and false negative rate.
Probability: The esimated probability that the sample is in the positive category. This assumes that the Bayesian score follows a normal distribution and is different from the prediction using a cutoff.
Enrichment: An estimate of enrichment, that is, the increased likelihood (versus random) of this sample being in the category.
Bayesian Score: The standard Laplacian-modified Bayesian score.
Mahalanobis Distance: The Mahalanobis distance (MD) is the distance to the center of the training data. The larger the MD, the less trustworthy the prediction.
Mahalanobis Distance p-value: The p-value gives the fraction of training data with an MD greater than or equal to the one for the given sample, assuming normally distributed data. The smaller the p-value, the less trustworthy the prediciton. For highly non-normal X properties (e.g., fingerprints), the MD p-value is wildly inaccurate.

Structural Similar Compounds

Name	Ethynodiol	Loratidine	Chlorpromazine
Structure	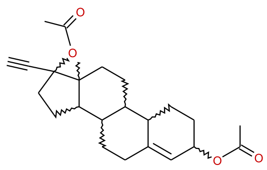	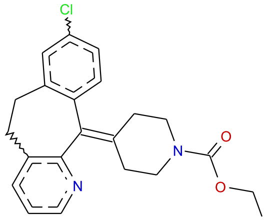	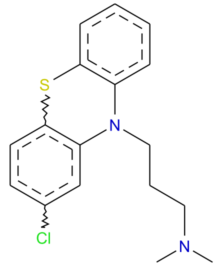
Actual Endpoint	Carcinogen	Non-Carcinogen	Non-Carcinogen
Predicted Endpoint	Carcinogen	Non-Carcinogen	Non-Carcinogen
Distance	0.567	0.579	0.620
Reference	US FDA (Centre for Drug Eval.& Res./Off. Testing & Res.) Sept. 1997	US FDA (Centre for Drug Eval.& Res./Off. Testing & Res.) Sept. 1997	US FDA (Centre for Drug Eval.& Res./Off. Testing & Res.) Sept. 1997

Model Applicability

Unknown features are fingerprint features in the query molecule, but not found or appearing too infrequently in the training set.

1. All properties and OPS components are within expected ranges.

Feature Contribution

Top features for positive contribution

Fingerprint	Bit/Smiles	Feature Structure	Score	Carcinogen in training set
ECFP_6	2082767335	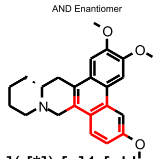 [*][c](:[*]):[c]1:[cH]:[cH]:[c]([*]):[*]:[c]:1:[*]	0.617	2 out of 2

ECFP_6	1978292697	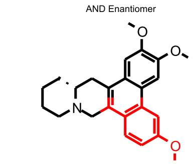 [*][c](:[*]):[c]1:[cH]:[cH]:[c](OC):[cH]: [c]:1:[*]	0.424	1 out of 1
ECFP_6	-1869628272	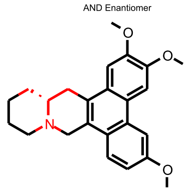 [*]CC(C[*])N([*])[*]	0.293	2 out of 4

Top Features for negative contribution

Fingerprint	Bit/Smiles	Feature Structure	Score	Carcinogen in training set
ECFP_6	-468366781	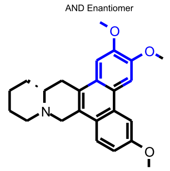 [*]O[c]1:[cH]:[*]:[c] (:[*]):[cH]:[c]:1OC	-0.805	0 out of 4
ECFP_6	1307307440	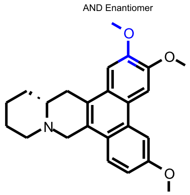 [*]:[c](:[*])OC	-0.558	4 out of 25
ECFP_6	-665049291	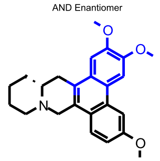 [*][c](:[*]):[c]1:[cH]:[c](OC):[c](OC):[c H]:[c]:1:[*]	-0.482	0 out of 2

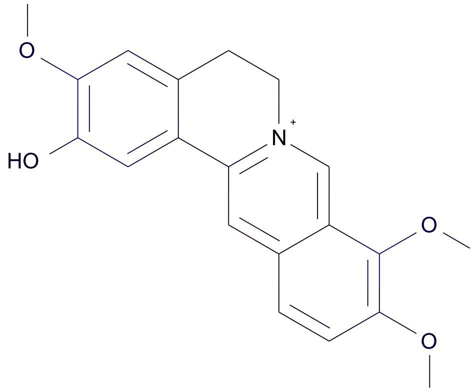

$C_{20}H_{20}NO_4$
Molecular Weight: 338.3771
ALogP: 3.936
Rotatable Bonds: 3
Acceptors: 4
Donors: 1

Structural Similar Compounds

Name	Indomethacin	Paroxetine	Nafenopin
Structure	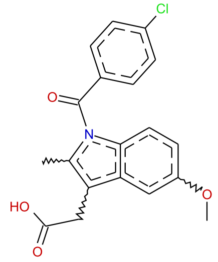	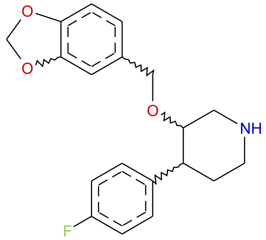	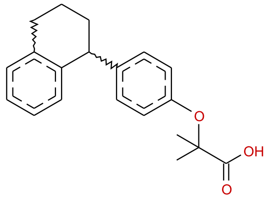
Actual Endpoint	Non-Carcinogen	Non-Carcinogen	Carcinogen
Predicted Endpoint	Non-Carcinogen	Non-Carcinogen	Carcinogen
Distance	0.551	0.583	0.604
Reference	US FDA (Centre for Drug Eval.& Res./Off. Testing & Res.) Sept. 1997	US FDA (Centre for Drug Eval.& Res./Off. Testing & Res.) Sept. 1997	US FDA (Centre for Drug Eval.& Res./Off. Testing & Res.) Sept. 1997

Model Prediction

Prediction: Non-Carcinogen
Probability: 0.208
Enrichment: 0.65
Bayesian Score: -4.66
Mahalanobis Distance: 12
Mahalanobis Distance p-value: 0.012

Prediction: Positive if the Bayesian score is above the estimated best cutoff value from minimizing the false positive and false negative rate.
Probability: The esimated probability that the sample is in the positive category. This assumes that the Bayesian score follows a normal distribution and is different from the prediction using a cutoff.
Enrichment: An estimate of enrichment, that is, the increased likelihood (versus random) of this sample being in the category.
Bayesian Score: The standard Laplacian-modified Bayesian score.
Mahalanobis Distance: The Mahalanobis distance (MD) is the distance to the center of the training data. The larger the MD, the less trustworthy the prediction.
Mahalanobis Distance p-value: The p-value gives the fraction of training data with an MD greater than or equal to the one for the given sample, assuming normally distributed data. The smaller the p-value, the less trustworthy the prediciton. For highly non-normal X properties (e.g., fingerprints), the MD p-value is wildly inaccurate.

Model Applicability

Unknown features are fingerprint features in the query molecule, but not found or appearing too infrequently in the training set.

1. All properties and OPS components are within expected ranges.
2. Unknown ECFP_2 feature: -1508366470: [*][n+](:[*]):[*]
3. Unknown ECFP_2 feature: 1508268466: [*]C[n+](:[c]([*]):[*]):c:[*]
4. Unknown ECFP_2 feature: -1333923932: [*]CC[n+](:[*]):[*]
5. Unknown ECFP_2 feature: 2078070048: [*][n+](:[*]):[c]([c](:[*]):[*]):c:[*]
6. Unknown ECFP_2 feature: 688916016: [*][n+](:[*]):c:[c](:[*]):[*]

Feature Contribution

Top features for positive contribution				
Fingerprint	Bit/Smiles	Feature Structure	Score	Carcinogen in training set

ECFP_6	51876938	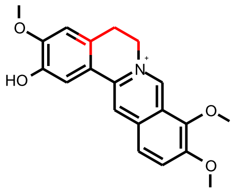 [*]CC[c](:[*]):[*]	0.473	16 out of 31
ECFP_6	-178525456	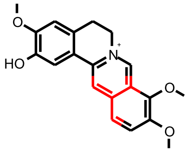 [*]:[cH]:[c](:[cH]:[*]):[c](:[*]):[*]	0.457	4 out of 7
ECFP_6	710652510	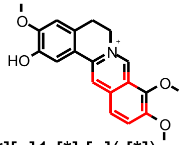 [*][c]1:[*]:[c](:[*]):[c](:[cH]:[*]):[cH]:[cH]:1	0.442	2 out of 3
**Top Features for negative contribution**				
**Fingerprint**	**Bit/Smiles**	**Feature Structure**	**Score**	**Carcinogen in training set**
ECFP_6	2077607946	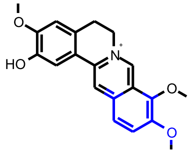 [*]O[c]1:[cH]:[cH]:[c](:[*]):[*]:[c]:1[*]	-1.15	0 out of 7
ECFP_6	1307307440	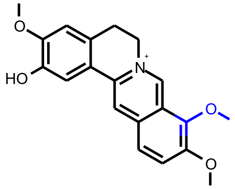 [*]:[c](:[*])OC	-0.558	4 out of 25

ECFP_6	1334400011	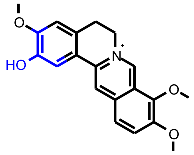 [*][c](:[*]):[c](O):[cH]:[*]	-0.496	3 out of 18

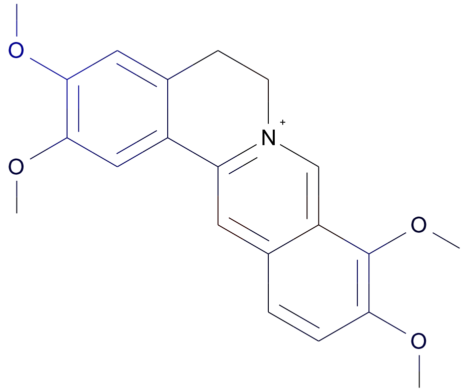

$C_{21}H_{22}NO_4$
Molecular Weight: 352.40368
ALogP: 4.161
Rotatable Bonds: 4
Acceptors: 4
Donors: 0

Structural Similar Compounds

Name	Ethynodiol	Chlorpromazine	Risperidone
Structure	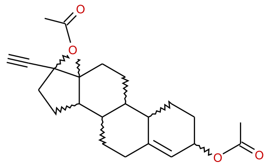	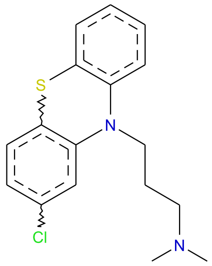	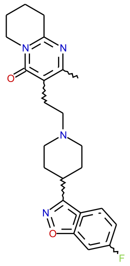
Actual Endpoint	Carcinogen	Non-Carcinogen	Carcinogen
Predicted Endpoint	Carcinogen	Non-Carcinogen	Carcinogen
Distance	0.573	0.593	0.607
Reference	US FDA (Centre for Drug Eval.& Res./Off. Testing & Res.) Sept. 1997	US FDA (Centre for Drug Eval.& Res./Off. Testing & Res.) Sept. 1997	US FDA (Centre for Drug Eval.& Res./Off. Testing & Res.) Sept. 1997

Model Prediction

Prediction: Non-Carcinogen
Probability: 0.206
Enrichment: 0.642
Bayesian Score: -5.41
Mahalanobis Distance: 11
Mahalanobis Distance p-value: 0.11

Prediction: Positive if the Bayesian score is above the estimated best cutoff value from minimizing the false positive and false negative rate.
Probability: The esimated probability that the sample is in the positive category. This assumes that the Bayesian score follows a normal distribution and is different from the prediction using a cutoff.
Enrichment: An estimate of enrichment, that is, the increased likelihood (versus random) of this sample being in the category.
Bayesian Score: The standard Laplacian-modified Bayesian score.
Mahalanobis Distance: The Mahalanobis distance (MD) is the distance to the center of the training data. The larger the MD, the less trustworthy the prediction.
Mahalanobis Distance p-value: The p-value gives the fraction of training data with an MD greater than or equal to the one for the given sample, assuming normally distributed data. The smaller the p-value, the less trustworthy the prediciton. For highly non-normal X properties (e.g., fingerprints), the MD p-value is wildly inaccurate.

Model Applicability

Unknown features are fingerprint features in the query molecule, but not found or appearing too infrequently in the training set.

1. All properties and OPS components are within expected ranges.
2. Unknown ECFP_2 feature: -1508366470: [*][n+](:[*]):[*]
3. Unknown ECFP_2 feature: 1508268466: [*]C[n+](:[c]([*]):[*]):c:[*]
4. Unknown ECFP_2 feature: -1333923932: [*]CC[n+](:[*]):[*]
5. Unknown ECFP_2 feature: 2078070048: [*][n+](:[*]):[c]([c](:[*]):[*]):c:[*]
6. Unknown ECFP_2 feature: 688916016: [*][n+](:[*]):c:[c](:[*]):[*]

Feature Contribution

Top features for positive contribution

Fingerprint	Bit/Smiles	Feature Structure	Score	Carcinogen in training set

ECFP_6	51876938	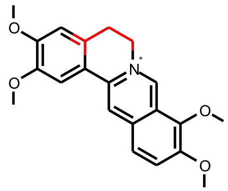 [*]CC[c](:[*]):[*]	0.473	16 out of 31
ECFP_6	-178525456	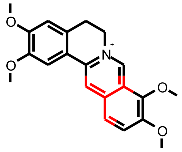 [*]:[cH]:[c](:[cH]:[*]):[c](:[*]):[*]	0.457	4 out of 7
ECFP_6	710652510	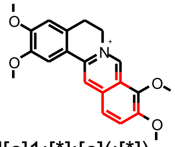 [*][c]1:[*]:[c](:[*]) :[c](:[cH]:[*]):[cH] :[cH]:1	0.442	2 out of 3
**Top Features for negative contribution**				
**Fingerprint**	**Bit/Smiles**	**Feature Structure**	**Score**	**Carcinogen in training set**
ECFP_6	2077607946	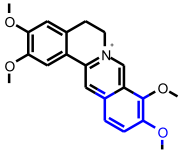 [*]O[c]1:[cH]:[cH]:[c](:[*]):[*]:[c]:1[*]	-1.15	0 out of 7
ECFP_6	-468366781	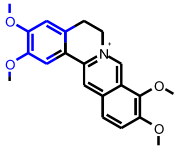 [*]O[c]1:[cH]:[*]:[c] (:[*]):[cH]:[c]:1OC	-0.805	0 out of 4

ECFP_6	1307307440	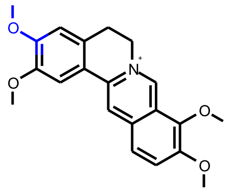 [*]:[c](:[*])OC	-0.558	4 out of 25

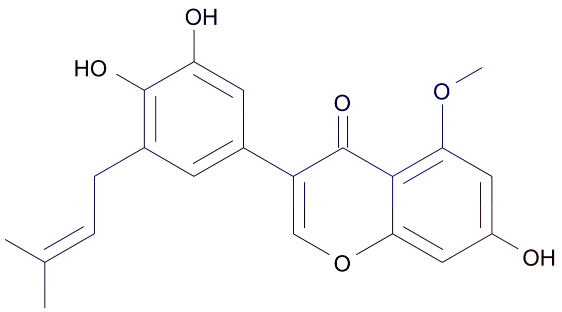

$C_{21}H_{20}O_6$
Molecular Weight: 368.3799
ALogP: 3.98
Rotatable Bonds: 4
Acceptors: 6
Donors: 3

Model Prediction

Prediction: Non-Carcinogen
Probability: 0.205
Enrichment: 0.639
Bayesian Score: -6.04
Mahalanobis Distance: 13.4
Mahalanobis Distance p-value: 0.000129

Prediction: Positive if the Bayesian score is above the estimated best cutoff value from minimizing the false positive and false negative rate.
Probability: The esimated probability that the sample is in the positive category. This assumes that the Bayesian score follows a normal distribution and is different from the prediction using a cutoff.
Enrichment: An estimate of enrichment, that is, the increased likelihood (versus random) of this sample being in the category.
Bayesian Score: The standard Laplacian-modified Bayesian score.
Mahalanobis Distance: The Mahalanobis distance (MD) is the distance to the center of the training data. The larger the MD, the less trustworthy the prediction.
Mahalanobis Distance p-value: The p-value gives the fraction of training data with an MD greater than or equal to the one for the given sample, assuming normally distributed data. The smaller the p-value, the less trustworthy the prediciton. For highly non-normal X properties (e.g., fingerprints), the MD p-value is wildly inaccurate.

Structural Similar Compounds

Name	Torsemide	Ursodiol	Niclosamide
Structure	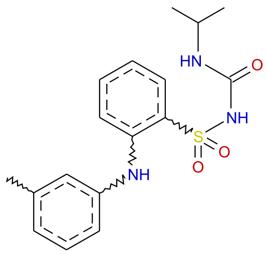	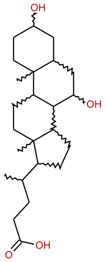	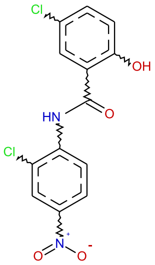
Actual Endpoint	Non-Carcinogen	Non-Carcinogen	Non-Carcinogen
Predicted Endpoint	Non-Carcinogen	Non-Carcinogen	Non-Carcinogen
Distance	0.669	0.675	0.719
Reference	US FDA (Centre for Drug Eval.& Res./Off. Testing & Res.) Sept. 1997	US FDA (Centre for Drug Eval.& Res./Off. Testing & Res.) Sept. 1997	US FDA (Centre for Drug Eval.& Res./Off. Testing & Res.) Sept. 1997

Model Applicability

Unknown features are fingerprint features in the query molecule, but not found or appearing too infrequently in the training set.

1. OPS PC25 out of range. Value: 2.6895. Training min, max, SD, explained variance: -2.879, 2.6681, 1.105, 0.0124.
2. Unknown ECFP_2 feature: 1717082529: [*]\C=C(/C(=[*])[*])\[c](:[*]):[*]
3. Unknown ECFP_2 feature: 471124258: [*]OC=C([*])[*]
4. Unknown ECFP_2 feature: -554736825: [*]=CO[c](:[*]):[*]
5. Unknown ECFP_2 feature: -1774681326: [*]C=C(C)C

Feature Contribution

Top features for positive contribution				
Fingerprint	Bit/Smiles	Feature Structure	Score	Carcinogen in training set

ECFP_6	-464490300	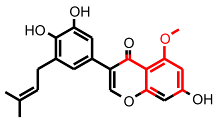 [*]C(=[*])[c]1:[c]([*]):[*]:[c]([*]):[cH] :[c]:1OC	0.424	1 out of 1
ECFP_6	-177786161	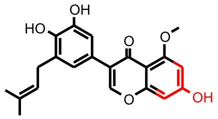 [*]:[cH]:[c](O):[cH]: [*]	0.406	7 out of 14
ECFP_6	-1925046727	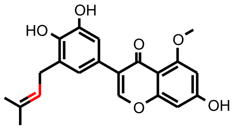 [*]C=[*]	0.391	11 out of 23
**Top Features for negative contribution**				
**Fingerprint**	**Bit/Smiles**	**Feature Structure**	**Score**	**Carcinogen in training set**
ECFP_6	1717462980	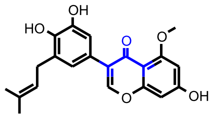 [*]C(=[*])C(=O)[c](:[*]):[*]	-1.25	0 out of 8
ECFP_6	-98561723	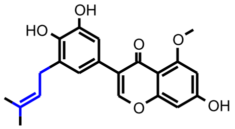 [*]CC=C([*])[*]	-0.657	0 out of 3

ECFP_6	1307307440	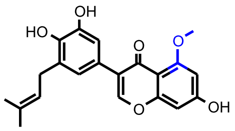<br>[*]:[c](:[*])OC	-0.558	4 out of 25

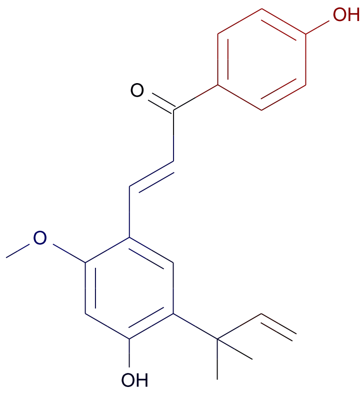

$C_{21}H_{22}O_4$
Molecular Weight: 338.39698
ALogP: 4.667
Rotatable Bonds: 6
Acceptors: 4
Donors: 2

Model Prediction

Prediction: Non-Carcinogen
Probability: 0.233
Enrichment: 0.728
Bayesian Score: -1.8
Mahalanobis Distance: 11.1
Mahalanobis Distance p-value: 0.0837

Prediction: Positive if the Bayesian score is above the estimated best cutoff value from minimizing the false positive and false negative rate.
Probability: The esimated probability that the sample is in the positive category. This assumes that the Bayesian score follows a normal distribution and is different from the prediction using a cutoff.
Enrichment: An estimate of enrichment, that is, the increased likelihood (versus random) of this sample being in the category.
Bayesian Score: The standard Laplacian-modified Bayesian score.
Mahalanobis Distance: The Mahalanobis distance (MD) is the distance to the center of the training data. The larger the MD, the less trustworthy the prediction.
Mahalanobis Distance p-value: The p-value gives the fraction of training data with an MD greater than or equal to the one for the given sample, assuming normally distributed data. The smaller the p-value, the less trustworthy the prediciton. For highly non-normal X properties (e.g., fingerprints), the MD p-value is wildly inaccurate.

Structural Similar Compounds

Name	Indomethacin	Diclofenac	Penbutalol
Structure	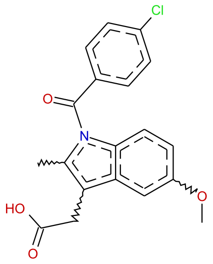	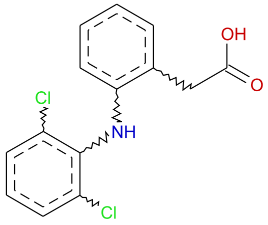	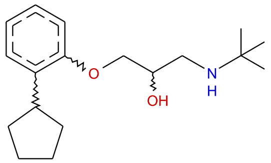
Actual Endpoint	Non-Carcinogen	Non-Carcinogen	Non-Carcinogen
Predicted Endpoint	Non-Carcinogen	Non-Carcinogen	Non-Carcinogen
Distance	0.628	0.631	0.637
Reference	US FDA (Centre for Drug Eval.& Res./Off. Testing & Res.) Sept. 1997	US FDA (Centre for Drug Eval.& Res./Off. Testing & Res.) Sept. 1997	US FDA (Centre for Drug Eval.& Res./Off. Testing & Res.) Sept. 1997

Model Applicability

Unknown features are fingerprint features in the query molecule, but not found or appearing too infrequently in the training set.

1. OPS PC13 out of range. Value: 6.3456. Training min, max, SD, explained variance: -3.9176, 5.0348, 1.522, 0.0236.
2. Unknown ECFP_2 feature: -144557007: [*]=CC(C)(C)[c](:[*]):[*]
3. Unknown ECFP_2 feature: -1193716553: [*]C([*])([*])C=C
4. Unknown ECFP_2 feature: 1335702447: [*][c](:[*]):[c](C=[*]):c:[*]
5. Unknown ECFP_2 feature: 1430764055: [*]=CC(=O)[c](:[*]):[*]

Feature Contribution

Top features for positive contribution				
Fingerprint	Bit/Smiles	Feature Structure	Score	Carcinogen in training set

ECFP_6	1419645508	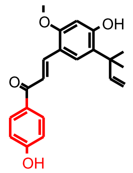 [*][c]1:[cH]:[cH]:[c](O):[cH]:[cH]:1	0.675	4 out of 5
ECFP_6	-790637051	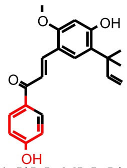 [*][c]1:[*]:[cH]:[c](O):[cH]:[cH]:1	0.615	6 out of 9
ECFP_6	1740779540	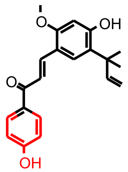 O[c]1:[cH]:[cH]:[*]:[cH]:[cH]:1	0.56	4 out of 6
**Top Features for negative contribution**				
**Fingerprint**	**Bit/Smiles**	**Feature Structure**	**Score**	**Carcinogen in training set**
ECFP_6	-1831055759	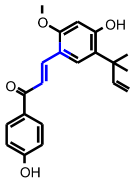 [*]\C=C\[c](:[*]):[*]	-0.805	0 out of 4
ECFP_6	1336678434	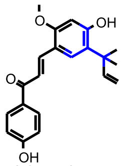 [*][c](:[*]):[c](:[cH]:[*])C([*])([*])[*]	-0.657	0 out of 3

ECFP_6	-470416293	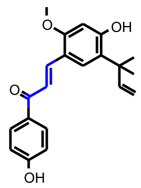<br>[*]\C=C\C(=[*])[*]	-0.657	0 out of 3

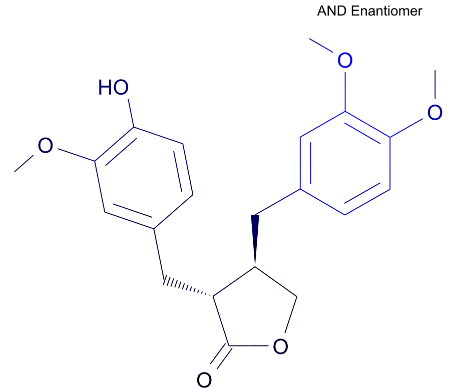

$C_{21}H_{24}O_6$
Molecular Weight: 372.41166
ALogP: 3.743
Rotatable Bonds: 7
Acceptors: 6
Donors: 1

Model Prediction

Prediction: Non-Carcinogen
Probability: 0.219
Enrichment: 0.685
Bayesian Score: -9.6
Mahalanobis Distance: 11.5
Mahalanobis Distance p-value: 0.0361

Prediction: Positive if the Bayesian score is above the estimated best cutoff value from minimizing the false positive and false negative rate.
Probability: The esimated probability that the sample is in the positive category. This assumes that the Bayesian score follows a normal distribution and is different from the prediction using a cutoff.
Enrichment: An estimate of enrichment, that is, the increased likelihood (versus random) of this sample being in the category.
Bayesian Score: The standard Laplacian-modified Bayesian score.
Mahalanobis Distance: The Mahalanobis distance (MD) is the distance to the center of the training data. The larger the MD, the less trustworthy the prediction.
Mahalanobis Distance p-value: The p-value gives the fraction of training data with an MD greater than or equal to the one for the given sample, assuming normally distributed data. The smaller the p-value, the less trustworthy the prediciton. For highly non-normal X properties (e.g., fingerprints), the MD p-value is wildly inaccurate.

Structural Similar Compounds

Name	Lovastatin	Moricizine	Felodipine
Structure	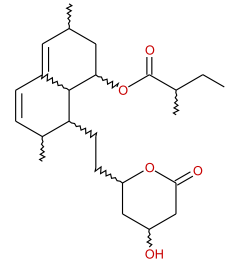	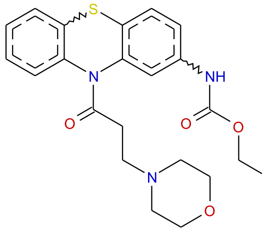	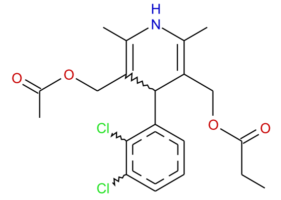
Actual Endpoint	Carcinogen	Carcinogen	Non-Carcinogen
Predicted Endpoint	Carcinogen	Carcinogen	Non-Carcinogen
Distance	0.570	0.597	0.598
Reference	US FDA (Centre for Drug Eval.& Res./Off. Testing & Res.) Sept. 1997	US FDA (Centre for Drug Eval.& Res./Off. Testing & Res.) Sept. 1997	US FDA (Centre for Drug Eval.& Res./Off. Testing & Res.) Sept. 1997

Model Applicability

Unknown features are fingerprint features in the query molecule, but not found or appearing too infrequently in the training set.

1. All properties and OPS components are within expected ranges.
2. Unknown ECFP_2 feature: 771121623: [*]C([*])C[c](:[*]):[*]

Feature Contribution

Top features for positive contribution				
Fingerprint	Bit/Smiles	Feature Structure	Score	Carcinogen in training set
ECFP_6	2106656448	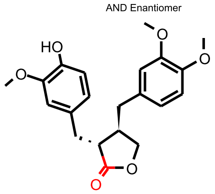 [*]C(=O)[*]	0.254	31 out of 77

ECFP_6	683445015	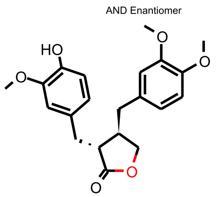<br>[*]O[*]	0.181	18 out of 48
ECFP_6	-992506539	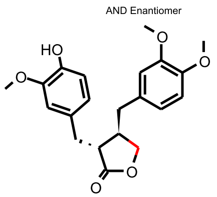<br>[*]C[*]	0.073	44 out of 132

Top Features for negative contribution

Fingerprint	Bit/Smiles	Feature Structure	Score	Carcinogen in training set
ECFP_6	2077607946	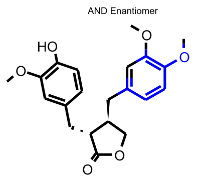<br>[*]O[c]1:[cH]:[cH]:[c](:[*]):[*]:[c]:1[*]	-1.15	0 out of 7
ECFP_6	-468366781	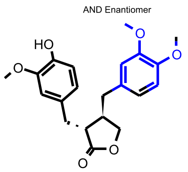<br>[*]O[c]1:[cH]:[*]:[c] (:[*]):[cH]:[c]:1OC	-0.805	0 out of 4
ECFP_6	1307307440	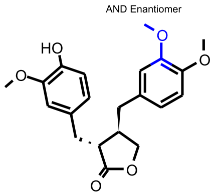<br>[*]:[c](:[*])OC	-0.558	4 out of 25

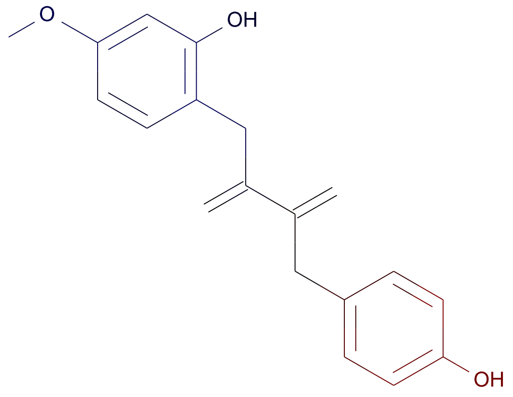

$C_{19}H_{20}O_3$

Molecular Weight: 296.3603

ALogP: 4.784

Rotatable Bonds: 6

Acceptors: 3

Donors: 2

Model Prediction

Prediction: Non-Carcinogen

Probability: 0.24

Enrichment: 0.749

Bayesian Score: -1.31

Mahalanobis Distance: 11.9

Mahalanobis Distance p-value: 0.0133

Prediction: Positive if the Bayesian score is above the estimated best cutoff value from minimizing the false positive and false negative rate.
Probability: The esimated probability that the sample is in the positive category. This assumes that the Bayesian score follows a normal distribution and is different from the prediction using a cutoff.
Enrichment: An estimate of enrichment, that is, the increased likelihood (versus random) of this sample being in the category.
Bayesian Score: The standard Laplacian-modified Bayesian score.
Mahalanobis Distance: The Mahalanobis distance (MD) is the distance to the center of the training data. The larger the MD, the less trustworthy the prediction.
Mahalanobis Distance p-value: The p-value gives the fraction of training data with an MD greater than or equal to the one for the given sample, assuming normally distributed data. The smaller the p-value, the less trustworthy the prediciton. For highly non-normal X properties (e.g., fingerprints), the MD p-value is wildly inaccurate.

Structural Similar Compounds

Name	Diethylstilbesterol	Diclofenac	Penbutalol
Structure	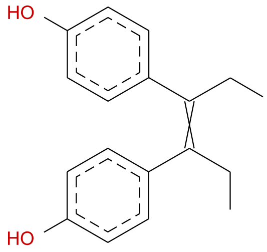	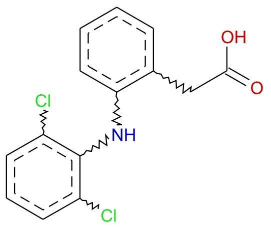	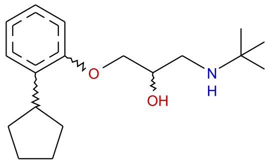
Actual Endpoint	Carcinogen	Non-Carcinogen	Non-Carcinogen
Predicted Endpoint	Carcinogen	Non-Carcinogen	Non-Carcinogen
Distance	0.560	0.585	0.597
Reference	US FDA (Centre for Drug Eval.& Res./Off. Testing & Res.) Sept. 1997	US FDA (Centre for Drug Eval.& Res./Off. Testing & Res.) Sept. 1997	US FDA (Centre for Drug Eval.& Res./Off. Testing & Res.) Sept. 1997

Model Applicability

Unknown features are fingerprint features in the query molecule, but not found or appearing too infrequently in the training set.

1. All properties and OPS components are within expected ranges.
2. Unknown ECFP_2 feature: -1505409543: [*]CC(=C)C(=[*])[*]
3. Unknown ECFP_2 feature: -2092468108: [*]C(=C)[*]

Feature Contribution

Top features for positive contribution

Fingerprint	Bit/Smiles	Feature Structure	Score	Carcinogen in training set
ECFP_6	1419645508	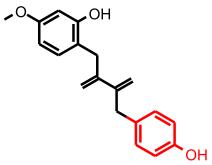 [*][c]1:[cH]:[cH]:[c](O):[cH]:[cH]:1	0.675	4 out of 5

ECFP_6	-790637051	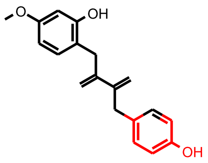 [*][c]1:[*]:[cH]:[c](O):[cH]:[cH]:1	0.615	6 out of 9
ECFP_6	1740779540	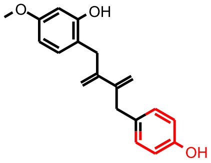 O[c]1:[cH]:[cH]:[*]:[cH]:[cH]:1	0.56	4 out of 6
**Top Features for negative contribution**				
**Fingerprint**	**Bit/Smiles**	**Feature Structure**	**Score**	**Carcinogen in training set**
ECFP_6	1307307440	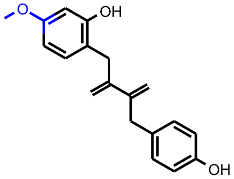 [*]:[c](:[*])OC	-0.558	4 out of 25
ECFP_6	1334400011	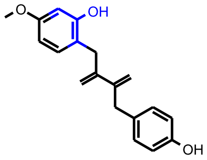 [*][c](:[*]):[c](O):[cH]:[*]	-0.496	3 out of 18
ECFP_6	864909220	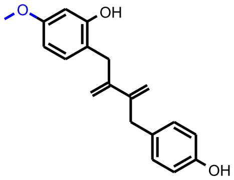 [*]OC	-0.466	7 out of 38

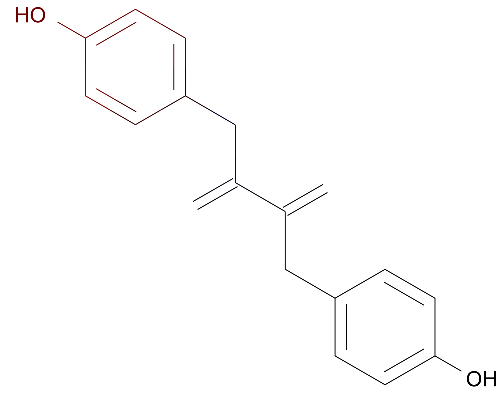

$C_{18}H_{18}O_2$
Molecular Weight: 266.33432
ALogP: 4.8
Rotatable Bonds: 5
Acceptors: 2
Donors: 2

Model Prediction

Prediction: Carcinogen
Probability: 0.301
Enrichment: 0.94
Bayesian Score: 1.63
Mahalanobis Distance: 11.1
Mahalanobis Distance p-value: 0.0876

Prediction: Positive if the Bayesian score is above the estimated best cutoff value from minimizing the false positive and false negative rate.
Probability: The esimated probability that the sample is in the positive category. This assumes that the Bayesian score follows a normal distribution and is different from the prediction using a cutoff.
Enrichment: An estimate of enrichment, that is, the increased likelihood (versus random) of this sample being in the category.
Bayesian Score: The standard Laplacian-modified Bayesian score.
Mahalanobis Distance: The Mahalanobis distance (MD) is the distance to the center of the training data. The larger the MD, the less trustworthy the prediction.
Mahalanobis Distance p-value: The p-value gives the fraction of training data with an MD greater than or equal to the one for the given sample, assuming normally distributed data. The smaller the p-value, the less trustworthy the prediciton. For highly non-normal X properties (e.g., fingerprints), the MD p-value is wildly inaccurate.

Structural Similar Compounds

Name	Diethylstilbesterol	Hexylresorcinol	Diclofenac
Structure	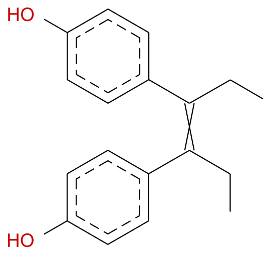	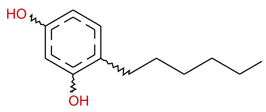	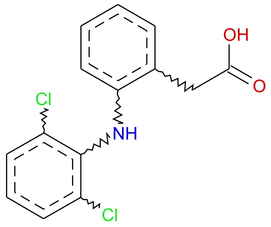
Actual Endpoint	Carcinogen	Non-Carcinogen	Non-Carcinogen
Predicted Endpoint	Carcinogen	Non-Carcinogen	Non-Carcinogen
Distance	0.424	0.572	0.576
Reference	US FDA (Centre for Drug Eval.& Res./Off. Testing & Res.) Sept. 1997	US FDA (Centre for Drug Eval.& Res./Off. Testing & Res.) Sept. 1997	US FDA (Centre for Drug Eval.& Res./Off. Testing & Res.) Sept. 1997

Model Applicability

Unknown features are fingerprint features in the query molecule, but not found or appearing too infrequently in the training set.

1. All properties and OPS components are within expected ranges.
2. Unknown ECFP_2 feature: -1505409543: [*]CC(=C)C(=[*])[*]
3. Unknown ECFP_2 feature: -2092468108: [*]C(=C)[*]

Feature Contribution

Top features for positive contribution				
**Fingerprint**	**Bit/Smiles**	**Feature Structure**	**Score**	**Carcinogen in training set**
ECFP_6	1419645508	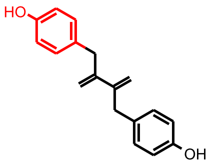 [*][c]1:[cH]:[cH]:[c](O):[cH]:[cH]:1	0.675	4 out of 5

ECFP_6	-790637051	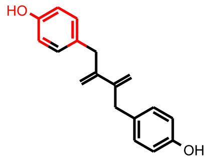 [*][c]1:[*]:[cH]:[c](O):[cH]:[cH]:1	0.615	6 out of 9
ECFP_6	1740779540	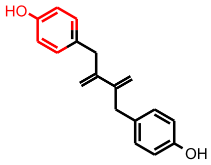 O[c]1:[cH]:[cH]:[*]:[cH]:[cH]:1	0.56	4 out of 6

Top Features for negative contribution

Fingerprint	Bit/Smiles	Feature Structure	Score	Carcinogen in training set
ECFP_6	771857573	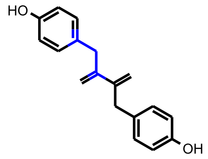 [*]C(=[*])C[c](:[*]): [*]	-0.459	1 out of 7
ECFP_6	1088861418	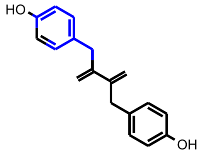 [*]C[c]1:[cH]:[*]:[c] ([*]):[cH]:[cH]:1	-0.225	4 out of 17
ECFP_6	1559650422	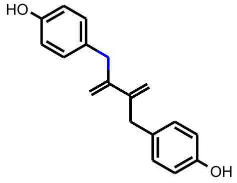 [*]C[*]	-0.164	50 out of 191

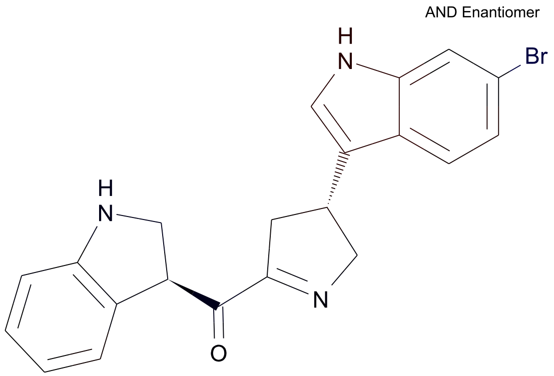

$C_{21}H_{18}BrN_3O$
Molecular Weight: 408.29112
ALogP: 3.919
Rotatable Bonds: 3
Acceptors: 3
Donors: 2

Structural Similar Compounds

Name	Mefloquine	Finasteride	Butorphanol
Structure	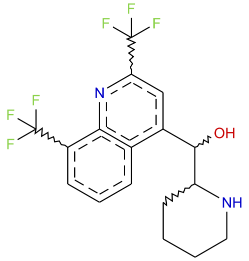	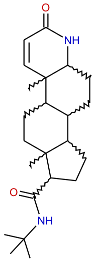	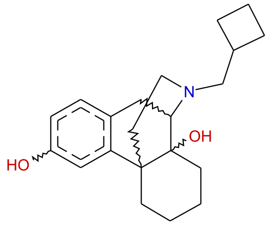
Actual Endpoint	Non-Carcinogen	Non-Carcinogen	Non-Carcinogen
Predicted Endpoint	Non-Carcinogen	Non-Carcinogen	Non-Carcinogen
Distance	0.561	0.614	0.632
Reference	US FDA (Centre for Drug Eval.& Res./Off. Testing & Res.) Sept. 1997	US FDA (Centre for Drug Eval.& Res./Off. Testing & Res.) Sept. 1997	US FDA (Centre for Drug Eval.& Res./Off. Testing & Res.) Sept. 1997

Model Prediction

Prediction: Non-Carcinogen
Probability: 0.236
Enrichment: 0.736
Bayesian Score: -1.61
Mahalanobis Distance: 11.5
Mahalanobis Distance p-value: 0.0409

Prediction: Positive if the Bayesian score is above the estimated best cutoff value from minimizing the false positive and false negative rate.
Probability: The esimated probability that the sample is in the positive category. This assumes that the Bayesian score follows a normal distribution and is different from the prediction using a cutoff.
Enrichment: An estimate of enrichment, that is, the increased likelihood (versus random) of this sample being in the category.
Bayesian Score: The standard Laplacian-modified Bayesian score.
Mahalanobis Distance: The Mahalanobis distance (MD) is the distance to the center of the training data. The larger the MD, the less trustworthy the prediction.
Mahalanobis Distance p-value: The p-value gives the fraction of training data with an MD greater than or equal to the one for the given sample, assuming normally distributed data. The smaller the p-value, the less trustworthy the prediciton. For highly non-normal X properties (e.g., fingerprints), the MD p-value is wildly inaccurate.

Model Applicability

Unknown features are fingerprint features in the query molecule, but not found or appearing too infrequently in the training set.

1. All properties and OPS components are within expected ranges.
2. Unknown ECFP_2 feature: -177935549: [*]:c:[c](Br):c:[*]
3. Unknown ECFP_2 feature: -116689887: [*][C@H]1[*][*]=NC1
4. Unknown ECFP_2 feature: 103000222: [*]C(=[*])C1=N[*][*]C1
5. Unknown ECFP_2 feature: 1431365708: [*]C([*])C(=O)C(=[*])[*]

Feature Contribution

Top features for positive contribution

Fingerprint	Bit/Smiles	Feature Structure	Score	Carcinogen in training set
ECFP_6	2082767335	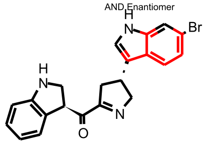 [*][c](:[*]):[c]1:[cH]:[cH]:[c]([*]):[*]:[c]:1:[*]	0.617	2 out of 2

ECFP_6	-953984246	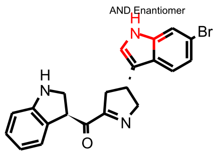 [*]:[c]1:[*]:[*]:[cH]:[nH]:1	0.364	4 out of 8
ECFP_6	1336304100	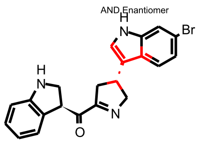 [*]C([*])[c](:[cH]:[*]):[c](:[*]):[*]	0.296	7 out of 16
**Top Features for negative contribution**				
**Fingerprint**	**Bit/Smiles**	**Feature Structure**	**Score**	**Carcinogen in training set**
ECFP_6	-302078100	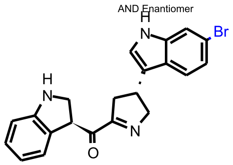 [*]Br	-0.805	0 out of 4
ECFP_6	459826767	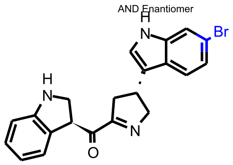 [*]:[c](:[*])Br	-0.482	0 out of 2
ECFP_6	-2095227870	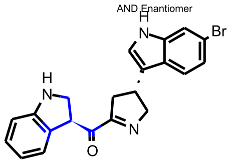 [*]C(=[*])[C@@H]1C[*][*]:[c]1:[*]	-0.27	0 out of 1

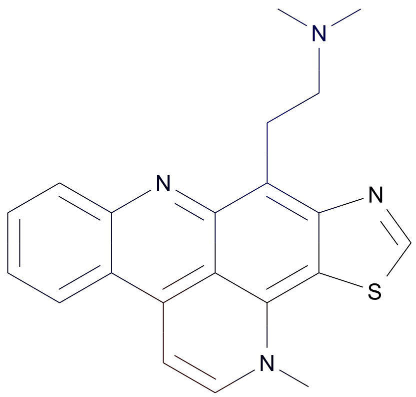

$C_{21}H_{20}N_4S$
Molecular Weight: 360.4753
ALogP: 3.682
Rotatable Bonds: 3
Acceptors: 4
Donors: 0

Structural Similar Compounds

Name	Chlormadinone	Risperidone	Chlorpromazine
Structure	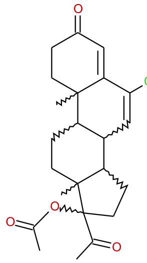	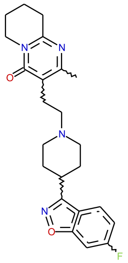	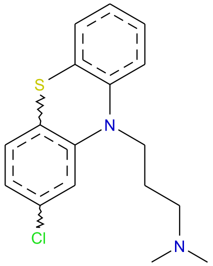
Actual Endpoint	Non-Carcinogen	Carcinogen	Non-Carcinogen
Predicted Endpoint	Carcinogen	Carcinogen	Non-Carcinogen
Distance	0.572	0.582	0.624
Reference	US FDA (Centre for Drug Eval.& Res./Off. Testing & Res.) Sept. 1997	US FDA (Centre for Drug Eval.& Res./Off. Testing & Res.) Sept. 1997	US FDA (Centre for Drug Eval.& Res./Off. Testing & Res.) Sept. 1997

Model Prediction

Prediction: Non-Carcinogen
Probability: 0.212
Enrichment: 0.663
Bayesian Score: -3.93
Mahalanobis Distance: 12.1
Mahalanobis Distance p-value: 0.00742

Prediction: Positive if the Bayesian score is above the estimated best cutoff value from minimizing the false positive and false negative rate.
Probability: The esimated probability that the sample is in the positive category. This assumes that the Bayesian score follows a normal distribution and is different from the prediction using a cutoff.
Enrichment: An estimate of enrichment, that is, the increased likelihood (versus random) of this sample being in the category.
Bayesian Score: The standard Laplacian-modified Bayesian score.
Mahalanobis Distance: The Mahalanobis distance (MD) is the distance to the center of the training data. The larger the MD, the less trustworthy the prediction.
Mahalanobis Distance p-value: The p-value gives the fraction of training data with an MD greater than or equal to the one for the given sample, assuming normally distributed data. The smaller the p-value, the less trustworthy the prediciton. For highly non-normal X properties (e.g., fingerprints), the MD p-value is wildly inaccurate.

Model Applicability

Unknown features are fingerprint features in the query molecule, but not found or appearing too infrequently in the training set.

1. All properties and OPS components are within expected ranges.
2. Unknown ECFP_2 feature: 1618095312: [*]=CN(C)[c](:[*]):[*]
3. Unknown ECFP_2 feature: -1658647648: [*]=C[c](:[c](:[*]):[*]):[c](:[*]):[*]
4. Unknown ECFP_2 feature: -1673960248: [*][c](:[*]):[c]1:s:[*]:[*]:[c]:1:[*]

Feature Contribution

Top features for positive contribution

Fingerprint	Bit/Smiles	Feature Structure	Score	Carcinogen in training set
ECFP_6	1745066357	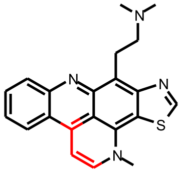 [*]\C=C/[c](:[*]):[*]	0.529	7 out of 12

ECFP_6	-1661653144	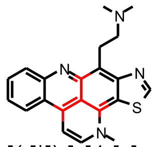 [*][c](:[*]):[c]1:[c](:[*]):[*]:[*]:[c]:1:[*]	0.442	2 out of 3
ECFP_6	935510419	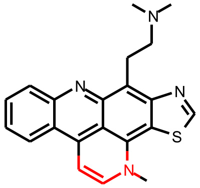 [*]\C=C/N([*])[*]	0.212	1 out of 2
**Top Features for negative contribution**				
**Fingerprint**	**Bit/Smiles**	**Feature Structure**	**Score**	**Carcinogen in training set**
ECFP_6	-512323383	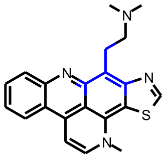 [*]C[c](:[c](:[*]):[*]):[c](:[*]):[*]	-0.935	0 out of 5
ECFP_6	629978456	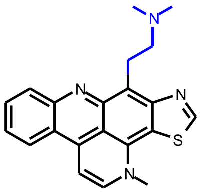 [*]CCN(C)C	-0.384	2 out of 11
ECFP_6	1048320787	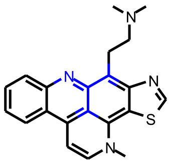 [*][c](:[*]):[c](:n:[*]):[c](:[*]):[*]	-0.356	1 out of 6

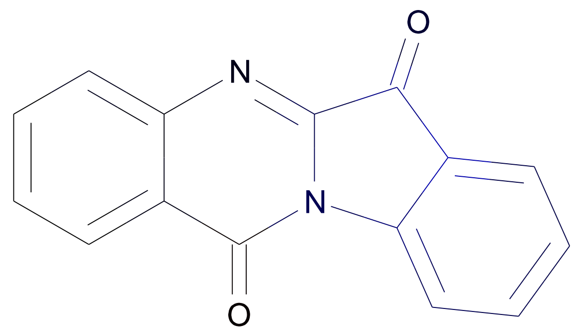

$C_{15}H_8N_2O_2$
Molecular Weight: 248.23621
ALogP: 2.331
Rotatable Bonds: 0
Acceptors: 3
Donors: 0

Model Prediction

Prediction: Non-Carcinogen
Probability: 0.21
Enrichment: 0.656
Bayesian Score: -4.27
Mahalanobis Distance: 8.98
Mahalanobis Distance p-value: 0.863

Prediction: Positive if the Bayesian score is above the estimated best cutoff value from minimizing the false positive and false negative rate.
Probability: The esimated probability that the sample is in the positive category. This assumes that the Bayesian score follows a normal distribution and is different from the prediction using a cutoff.
Enrichment: An estimate of enrichment, that is, the increased likelihood (versus random) of this sample being in the category.
Bayesian Score: The standard Laplacian-modified Bayesian score.
Mahalanobis Distance: The Mahalanobis distance (MD) is the distance to the center of the training data. The larger the MD, the less trustworthy the prediction.
Mahalanobis Distance p-value: The p-value gives the fraction of training data with an MD greater than or equal to the one for the given sample, assuming normally distributed data. The smaller the p-value, the less trustworthy the prediciton. For highly non-normal X properties (e.g., fingerprints), the MD p-value is wildly inaccurate.

Structural Similar Compounds

Name	Levamisole	Estazolam	Temazepam
Structure	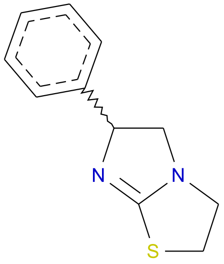	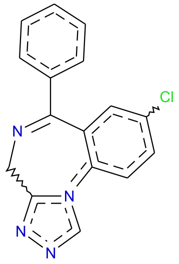	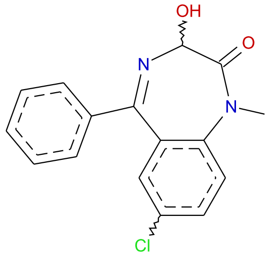
Actual Endpoint	Non-Carcinogen	Non-Carcinogen	Non-Carcinogen
Predicted Endpoint	Non-Carcinogen	Non-Carcinogen	Non-Carcinogen
Distance	0.556	0.621	0.635
Reference	US FDA (Centre for Drug Eval.& Res./Off. Testing & Res.) Sept. 1997	US FDA (Centre for Drug Eval.& Res./Off. Testing & Res.) Sept. 1997	US FDA (Centre for Drug Eval.& Res./Off. Testing & Res.) Sept. 1997

Model Applicability

Unknown features are fingerprint features in the query molecule, but not found or appearing too infrequently in the training set.

1. All properties and OPS components are within expected ranges.
2. Unknown ECFP_2 feature: -962771238: [*]C(=[*])N1C(=[*])[*][*]:[c]1:[*]

Feature Contribution

Top features for positive contribution

Fingerprint	Bit/Smiles	Feature Structure	Score	Carcinogen in training set
ECFP_6	2085698692	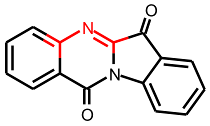 [*]C(=N[c](:[*]):[*])[*]	0.337	3 out of 6

ECFP_6	2106656448	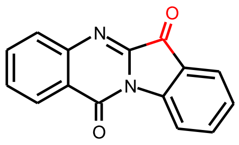 [*]C(=O)[*]	0.254	31 out of 77
ECFP_6	1945129186	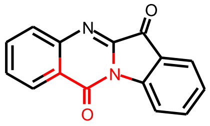 [*]N([*])C(=O)[c](:[*]):[*]	0.164	2 out of 5
**Top Features for negative contribution**				
**Fingerprint**	**Bit/Smiles**	**Feature Structure**	**Score**	**Carcinogen in training set**
ECFP_6	1717462980	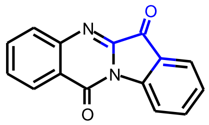 [*]C(=[*])C(=O)[c](:[*]):[*]	-1.25	0 out of 8
ECFP_6	-427397688	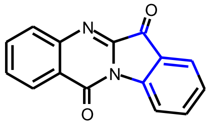 [*][c](:[*]):[c](:[cH]:[*])[c](:[*]):[*]	-0.476	5 out of 28
ECFP_6	-1236953626	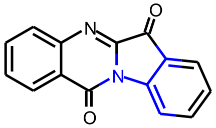 [*]N1[*][*][c](:[*]): [c]1:[cH]:[*]	-0.448	3 out of 17

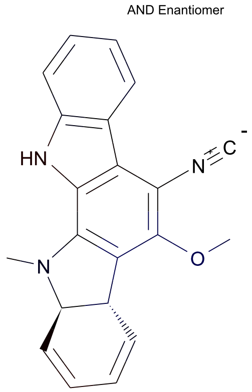

$C_{21}H_{17}N_3O$
Molecular Weight: 327.37918
ALogP: 4.078
Rotatable Bonds: 1
Acceptors: 2
Donors: 1

Model Prediction

Prediction: Non-Carcinogen
Probability: 0.229
Enrichment: 0.713
Bayesian Score: -2.17
Mahalanobis Distance: 12
Mahalanobis Distance p-value: 0.00998

Prediction: Positive if the Bayesian score is above the estimated best cutoff value from minimizing the false positive and false negative rate.
Probability: The esimated probability that the sample is in the positive category. This assumes that the Bayesian score follows a normal distribution and is different from the prediction using a cutoff.
Enrichment: An estimate of enrichment, that is, the increased likelihood (versus random) of this sample being in the category.
Bayesian Score: The standard Laplacian-modified Bayesian score.
Mahalanobis Distance: The Mahalanobis distance (MD) is the distance to the center of the training data. The larger the MD, the less trustworthy the prediction.
Mahalanobis Distance p-value: The p-value gives the fraction of training data with an MD greater than or equal to the one for the given sample, assuming normally distributed data. The smaller the p-value, the less trustworthy the prediciton. For highly non-normal X properties (e.g., fingerprints), the MD p-value is wildly inaccurate.

Structural Similar Compounds

Name	Levonorgestrel	Norethindrone	Norethynodrel
Structure	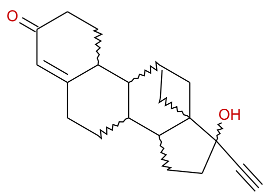	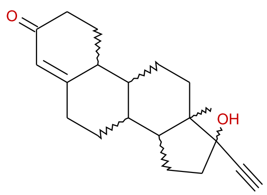	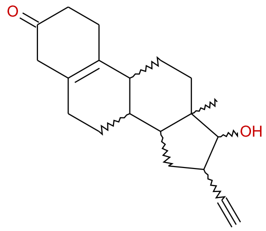
Actual Endpoint	Carcinogen	Carcinogen	Carcinogen
Predicted Endpoint	Carcinogen	Carcinogen	Carcinogen
Distance	0.587	0.590	0.593
Reference	US FDA (Centre for Drug Eval.& Res./Off. Testing & Res.) Sept. 1997	US FDA (Centre for Drug Eval.& Res./Off. Testing & Res.) Sept. 1997	US FDA (Centre for Drug Eval.& Res./Off. Testing & Res.) Sept. 1997

Model Applicability

Unknown features are fingerprint features in the query molecule, but not found or appearing too infrequently in the training set.

1. All properties and OPS components are within expected ranges.
2. Unknown ECFP_2 feature: 1029014155: [*][N+]#[*]
3. Unknown ECFP_2 feature: 726108635: [*]#[C-]
4. Unknown ECFP_2 feature: 1464683384: [*][c](:[*]):[c]([N+]#[*]):[c](:[*]):[*]
5. Unknown ECFP_2 feature: -11961319: [*]:[c](:[*])[N+]#[C-]
6. Unknown ECFP_2 feature: -1334780583: [*][N+]#[C-]

Feature Contribution

Top features for positive contribution				
**Fingerprint**	**Bit/Smiles**	**Feature Structure**	**Score**	**Carcinogen in training set**

ECFP_6	-1661653144	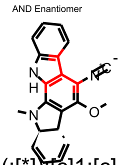 [*][c](:[*]):[c]1:[c](:[*]):[*]:[*]:[c]:1:[*]	0.442	2 out of 3
ECFP_6	1617733200	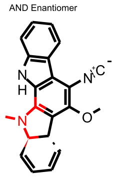 [*][C@@H]1[*][*]:[c](:[*])N1C	0.424	1 out of 1
ECFP_6	890368401	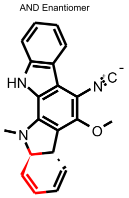 [*]\C=C/C([*])[*]	0.337	3 out of 6

Top Features for negative contribution

**Fingerprint**	**Bit/Smiles**	**Feature Structure**	**Score**	**Carcinogen in training set**
ECFP_6	1307307440	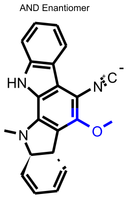 [*]:[c](:[*])OC	-0.558	4 out of 25
ECFP_6	-1565641546	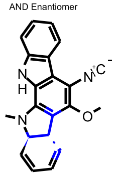 [*][C@H]1[*][*]:[c](:[*])[C@@H]1C=[*]	-0.482	0 out of 2

ECFP_6	-1659009760	AND Enantiomer 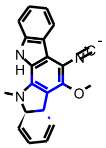 [*][C@@H]1[*][*][c](:[*]):[c]1:[c]([*]):[*]	-0.482	0 out of 2

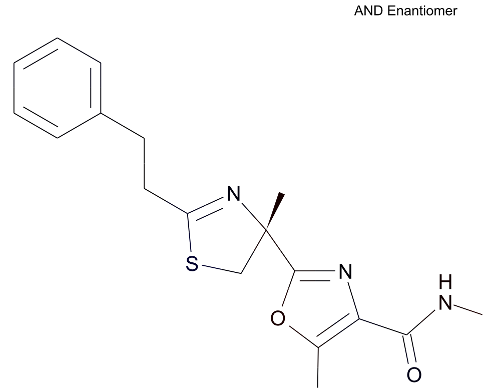

$C_{18}H_{21}N_3O_2S$
Molecular Weight: 343.44324
ALogP: 2.716
Rotatable Bonds: 5
Acceptors: 4
Donors: 1

Model Prediction

Prediction: Non-Carcinogen
Probability: 0.236
Enrichment: 0.737
Bayesian Score: -1.59
Mahalanobis Distance: 13.9
Mahalanobis Distance p-value: 1.95e-005

Prediction: Positive if the Bayesian score is above the estimated best cutoff value from minimizing the false positive and false negative rate.
Probability: The esimated probability that the sample is in the positive category. This assumes that the Bayesian score follows a normal distribution and is different from the prediction using a cutoff.
Enrichment: An estimate of enrichment, that is, the increased likelihood (versus random) of this sample being in the category.
Bayesian Score: The standard Laplacian-modified Bayesian score.
Mahalanobis Distance: The Mahalanobis distance (MD) is the distance to the center of the training data. The larger the MD, the less trustworthy the prediction.
Mahalanobis Distance p-value: The p-value gives the fraction of training data with an MD greater than or equal to the one for the given sample, assuming normally distributed data. The smaller the p-value, the less trustworthy the prediciton. For highly non-normal X properties (e.g., fingerprints), the MD p-value is wildly inaccurate.

Structural Similar Compounds

Name	Omeprazole	Lansoprazole	Oxaprocin
Structure	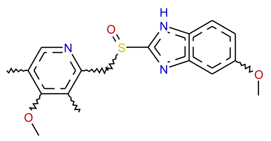	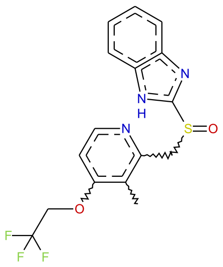	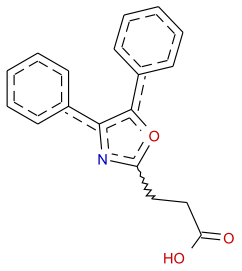
Actual Endpoint	Non-Carcinogen	Carcinogen	Non-Carcinogen
Predicted Endpoint	Non-Carcinogen	Carcinogen	Non-Carcinogen
Distance	0.550	0.557	0.571
Reference	US FDA (Centre for Drug Eval.& Res./Off. Testing & Res.) Sept. 1997	US FDA (Centre for Drug Eval.& Res./Off. Testing & Res.) Sept. 1997	US FDA (Centre for Drug Eval.& Res./Off. Testing & Res.) Sept. 1997

Model Applicability

Unknown features are fingerprint features in the query molecule, but not found or appearing too infrequently in the training set.

1. All properties and OPS components are within expected ranges.
2. Unknown ECFP_2 feature: 309047694: [*]C([*])([*])[c]1:o:[*]:[*]:n:1
3. Unknown ECFP_2 feature: 1576608821: [*][c]1:[*]:[*]:o:[c]:1C
4. Unknown ECFP_2 feature: -1073216586: [*]CC1=N[*][*]S1
5. Unknown ECFP_2 feature: 1920241679: [*]C1([*])[*]=[*]SC1
6. Unknown ECFP_2 feature: 618128563: [*]:[c](:[*])[C@]1(C)C[*][*]=N1

Feature Contribution

Top features for positive contribution				
Fingerprint	Bit/Smiles	Feature Structure	Score	Carcinogen in training set

ECFP_6	1338334141	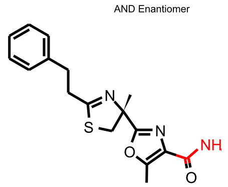 [*]C(=[*])NC	0.442	2 out of 3
ECFP_6	859433814	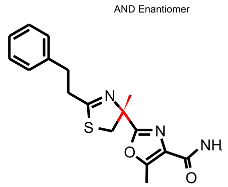 [*]C([*])([*])C	0.408	14 out of 29
ECFP_6	1203316083	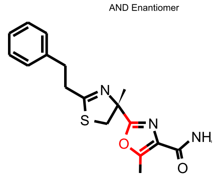 [*][c]1:[*]:[*]:[c]([*]):o:1	0.337	3 out of 6

Top Features for negative contribution

**Fingerprint**	**Bit/Smiles**	**Feature Structure**	**Score**	**Carcinogen in training set**
ECFP_6	912478223	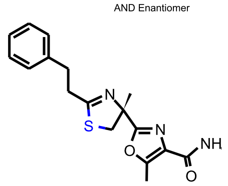 [*]S[*]	-0.638	1 out of 9
ECFP_6	1430169877	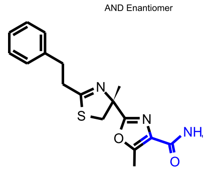 [*]NC(=O)[c](:[*]):[*]	-0.287	3 out of 14

ECFP_6	2127097785	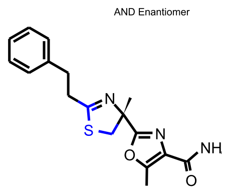 [*]C1=[*][*]CS1	-0.27	0 out of 1

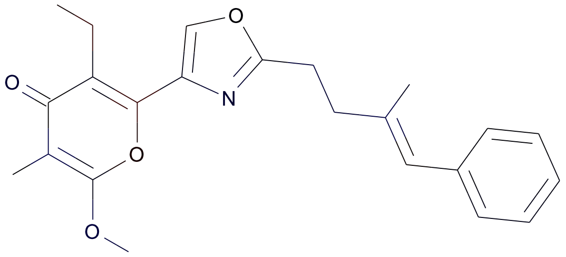

$C_{23}H_{25}NO_4$
Molecular Weight: 379.4489
ALogP: 5.22
Rotatable Bonds: 7
Acceptors: 4
Donors: 0

Model Prediction

Prediction: Non-Carcinogen
Probability: 0.228
Enrichment: 0.711
Bayesian Score: -2.24
Mahalanobis Distance: 15.1
Mahalanobis Distance p-value: 1.68e-007

Prediction: Positive if the Bayesian score is above the estimated best cutoff value from minimizing the false positive and false negative rate.
Probability: The esimated probability that the sample is in the positive category. This assumes that the Bayesian score follows a normal distribution and is different from the prediction using a cutoff.
Enrichment: An estimate of enrichment, that is, the increased likelihood (versus random) of this sample being in the category.
Bayesian Score: The standard Laplacian-modified Bayesian score.
Mahalanobis Distance: The Mahalanobis distance (MD) is the distance to the center of the training data. The larger the MD, the less trustworthy the prediction.
Mahalanobis Distance p-value: The p-value gives the fraction of training data with an MD greater than or equal to the one for the given sample, assuming normally distributed data. The smaller the p-value, the less trustworthy the prediciton. For highly non-normal X properties (e.g., fingerprints), the MD p-value is wildly inaccurate.

Structural Similar Compounds

Name	Permethrin	Etretinate	Flurazepam
Structure	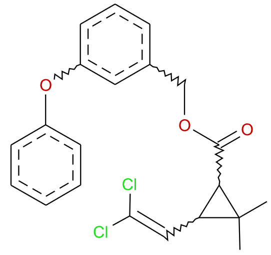	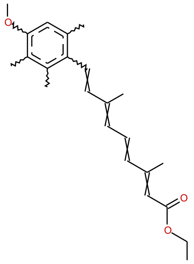	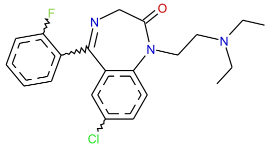
Actual Endpoint	Carcinogen	Non-Carcinogen	Non-Carcinogen
Predicted Endpoint	Carcinogen	Non-Carcinogen	Non-Carcinogen
Distance	0.563	0.618	0.619
Reference	US FDA (Centre for Drug Eval.& Res./Off. Testing & Res.) Sept. 1997	US FDA (Centre for Drug Eval.& Res./Off. Testing & Res.) Sept. 1997	US FDA (Centre for Drug Eval.& Res./Off. Testing & Res.) Sept. 1997

Model Applicability

Unknown features are fingerprint features in the query molecule, but not found or appearing too infrequently in the training set.

1. All properties and OPS components are within expected ranges.
2. Unknown ECFP_2 feature: 1796421070: [*]OC(=C([*])[*])[c](:[*]):[*]
3. Unknown ECFP_2 feature: 1792159373: [*]C(=C(C)C(=[*])[*])[*]
4. Unknown ECFP_2 feature: 1651701028: [*]OC(=C([*])[*])O[*]
5. Unknown ECFP_2 feature: -785659985: [*][c]1:[*]:[*]:o:c:1
6. Unknown ECFP_2 feature: -1832568576: [*]C(=C[c](:[*]):[*])[*]
7. Unknown ECFP_2 feature: -176483725: [*]=C[c](:c:[*]):c:[*]

Feature Contribution

Top features for positive contribution

Fingerprint	Bit/Smiles	Feature Structure	Score	Carcinogen in training set

ECFP_6	-770854792	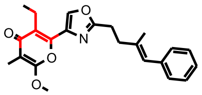 [*]CC(=C([*])[*])C(=[*])[*]	0.617	2 out of 2
ECFP_6	1667043235	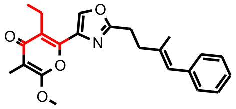 [*]C(=C(CC)C(=[*])[*])[*]	0.424	1 out of 1
ECFP_6	-308870089	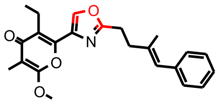 [*][c]1:[*]:[*]:[cH]:o:1	0.424	1 out of 1
**Top Features for negative contribution**				
**Fingerprint**	**Bit/Smiles**	**Feature Structure**	**Score**	**Carcinogen in training set**
ECFP_6	1717462980	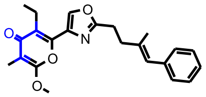 [*]C(=[*])C(=O)[c](:[*]):[*]	-1.25	0 out of 8
ECFP_6	1307307440	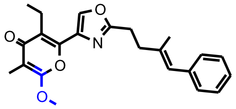 [*]:[c](:[*])OC	-0.558	4 out of 25

ECFP_6	864909220	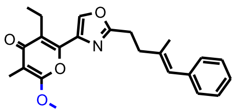 [*]OC	-0.466	7 out of 38

Remdesivir

TOPKAT_Mouse_Female_FDA_None_vs_Carcinogen

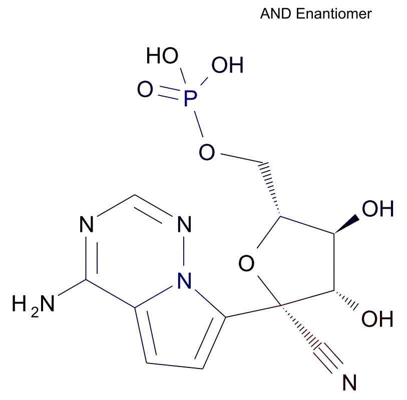

$C_{12}H_{14}N_5O_7P$

Molecular Weight: 371.24262

ALogP: -1.502

Rotatable Bonds: 4

Acceptors: 11

Donors: 5

Structural Similar Compounds

Name	Famotidine	Tetracycline	Oxytetracycline
Structure	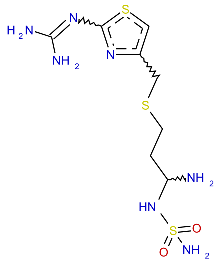	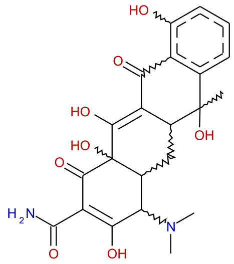	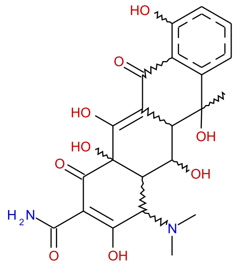
Actual Endpoint	Non-Carcinogen	Non-Carcinogen	Non-Carcinogen
Predicted Endpoint	Non-Carcinogen	Non-Carcinogen	Non-Carcinogen
Distance	0.846	0.848	0.870
Reference	US FDA (Centre for Drug Eval.& Res./Off. Testing & Res.) Sept. 1997	US FDA (Centre for Drug Eval.& Res./Off. Testing & Res.) Sept. 1997	US FDA (Centre for Drug Eval.& Res./Off. Testing & Res.) Sept. 1997

Model Prediction

Prediction: Non-Carcinogen

Probability: 0.206

Enrichment: 0.642

Bayesian Score: -7.17

Mahalanobis Distance: 12.9

Mahalanobis Distance p-value: 0.00074

Prediction: Positive if the Bayesian score is above the estimated best cutoff value from minimizing the false positive and false negative rate.
Probability: The esimated probability that the sample is in the positive category. This assumes that the Bayesian score follows a normal distribution and is different from the prediction using a cutoff.
Enrichment: An estimate of enrichment, that is, the increased likelihood (versus random) of this sample being in the category.
Bayesian Score: The standard Laplacian-modified Bayesian score.
Mahalanobis Distance: The Mahalanobis distance (MD) is the distance to the center of the training data. The larger the MD, the less trustworthy the prediction.
Mahalanobis Distance p-value: The p-value gives the fraction of training data with an MD greater than or equal to the one for the given sample, assuming normally distributed data. The smaller the p-value, the less trustworthy the prediciton. For highly non-normal X properties (e.g., fingerprints), the MD p-value is wildly inaccurate.

Model Applicability

Unknown features are fingerprint features in the query molecule, but not found or appearing too infrequently in the training set.

1. All properties and OPS components are within expected ranges.
2. Unknown ECFP_2 feature: 1126642748: [*]OP(=O)(O)O
3. Unknown ECFP_2 feature: -1250439909: [*]COP(=[*])([*])[*]
4. Unknown ECFP_2 feature: 1258791451: [*][C@H]1[*][*]O[C@]1(C#[*])[c](:[*]):[*]
5. Unknown ECFP_2 feature: -1507082173: [*][c]1:[*]:[*]:[c](:[*]):n:1:n:[*]
6. Unknown ECFP_2 feature: -66263742: [*]C([*])([*])[c]1:n(:[*]):[*]:[*]:c:1

Feature Contribution

Top features for positive contribution				
**Fingerprint**	**Bit/Smiles**	**Feature Structure**	**Score**	**Carcinogen in training set**

ECFP_6	-1114776580	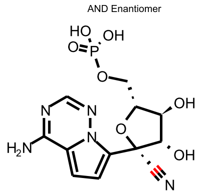 [*]C#[*]	0.755	11 out of 15
ECFP_6	-521596699	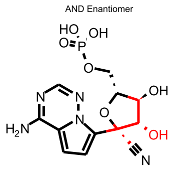 [*][C@@H]1[*][*]C([*])([*])[C@H]1O	0.451	3 out of 5
ECFP_6	-264833661	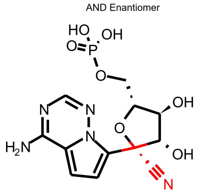 [*]C([*])([*])C#N	0.424	1 out of 1
**Top Features for negative contribution**				
**Fingerprint**	**Bit/Smiles**	**Feature Structure**	**Score**	**Carcinogen in training set**
ECFP_6	2100964382	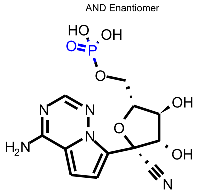 [*]P(=O)([*])[*]	-0.935	0 out of 5
ECFP_6	-826638028	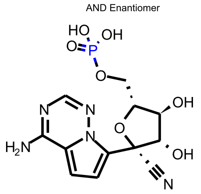 [*]P(=[*])([*])[*]	-0.935	0 out of 5

ECFP_6	1334415134	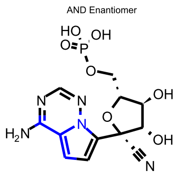 [*][c](:[*]):[c]1:[cH]:[*]:[*]:n:1:[*]	-0.935	0 out of 5

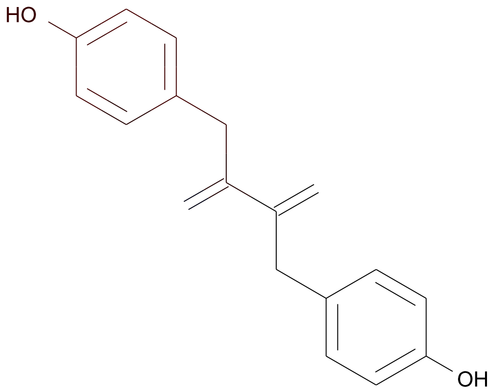

$C_{18}H_{18}O_2$
Molecular Weight: 266.33432
ALogP: 4.8
Rotatable Bonds: 5
Acceptors: 2
Donors: 2

Model Prediction

Prediction: Multiple-Carcinogen
Probability: 0.478
Enrichment: 1.17
Bayesian Score: 1.74
Mahalanobis Distance: 8.12
Mahalanobis Distance p-value: 0.244

Prediction: Positive if the Bayesian score is above the estimated best cutoff value from minimizing the false positive and false negative rate.
Probability: The esimated probability that the sample is in the positive category. This assumes that the Bayesian score follows a normal distribution and is different from the prediction using a cutoff.
Enrichment: An estimate of enrichment, that is, the increased likelihood (versus random) of this sample being in the category.
Bayesian Score: The standard Laplacian-modified Bayesian score.
Mahalanobis Distance: The Mahalanobis distance (MD) is the distance to the center of the training data. The larger the MD, the less trustworthy the prediction.
Mahalanobis Distance p-value: The p-value gives the fraction of training data with an MD greater than or equal to the one for the given sample, assuming normally distributed data. The smaller the p-value, the less trustworthy the prediciton. For highly non-normal X properties (e.g., fingerprints), the MD p-value is wildly inaccurate.

Structural Similar Compounds

Name	Diethylstilbesterol	Pronetalol	Nafenopin
Structure	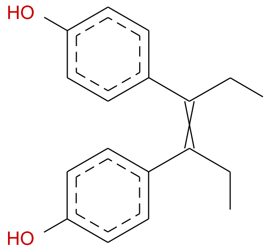	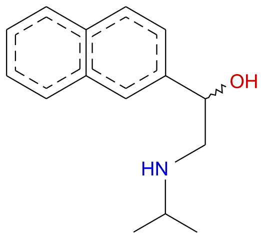	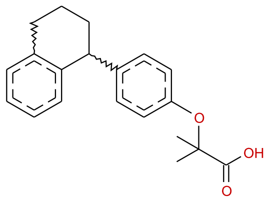
Actual Endpoint	Multiple-Carcinogen	Single-Carcinogen	Single-Carcinogen
Predicted Endpoint	Multiple-Carcinogen	Single-Carcinogen	Single-Carcinogen
Distance	0.389	0.669	0.676
Reference	US FDA (Centre for Drug Eval.& Res./Off. Testing & Res.) Sept. 1997	US FDA (Centre for Drug Eval.& Res./Off. Testing & Res.) Sept. 1997	US FDA (Centre for Drug Eval.& Res./Off. Testing & Res.) Sept. 1997

Model Applicability

Unknown features are fingerprint features in the query molecule, but not found or appearing too infrequently in the training set.

1. All properties and OPS components are within expected ranges.
2. Unknown ECFP_2 feature: -1505409543: [*]CC(=C)C(=[*])[*]
3. Unknown ECFP_2 feature: -2092468108: [*]C(=C)[*]

Feature Contribution

Top features for positive contribution

Fingerprint	Bit/Smiles	Feature Structure	Score	Multiple-Carcinogen in training set
ECFP_4	-177786161	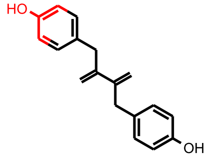 [*]:[cH]:[c](O):[cH]:[*]	0.441	5 out of 7

ECFP_4	-790637051	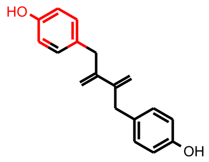 [*][c]1:[*]:[cH]:[c](O):[cH]:[cH]:1	0.371	4 out of 6
ECFP_4	771857573	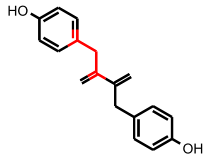 [*]C(=[*])C[c](:[*]):[*]	0.351	1 out of 1
**Top Features for negative contribution**				
**Fingerprint**	**Bit/Smiles**	**Feature Structure**	**Score**	**Multiple-Carcinogen in training set**
ECFP_4	1544874086	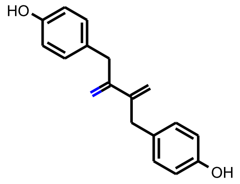 [*]=C	-0.342	0 out of 1
ECFP_4	-1100000244	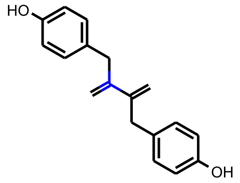 [*]C(=[*])[*]	-0.088	16 out of 43
ECFP_4	-182236392	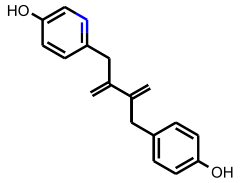 [*]:[cH]:[*]	-0.0651	34 out of 89

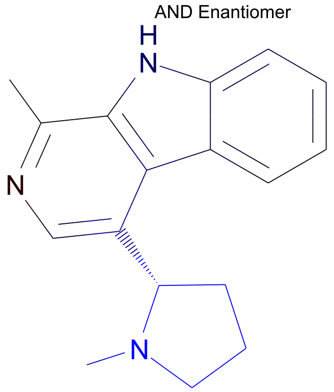

$C_{17}H_{19}N_3$
Molecular Weight: 265.35286
ALogP: 3.018
Rotatable Bonds: 1
Acceptors: 2
Donors: 1

Structural Similar Compounds

Name	Granisetron	Ondansetron	Temazepam
Structure	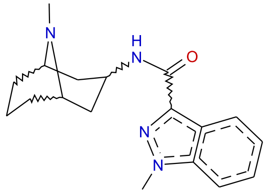	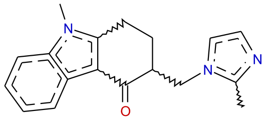	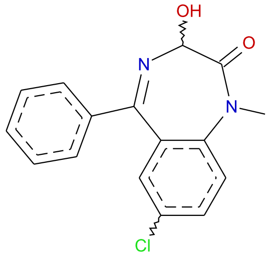
Actual Endpoint	Carcinogen	Non-Carcinogen	Non-Carcinogen
Predicted Endpoint	Carcinogen	Non-Carcinogen	Non-Carcinogen
Distance	0.583	0.592	0.607
Reference	US FDA (Centre for Drug Eval.& Res./Off. Testing & Res.) Sept. 1997	US FDA (Centre for Drug Eval.& Res./Off. Testing & Res.) Sept. 1997	US FDA (Centre for Drug Eval.& Res./Off. Testing & Res.) Sept. 1997

Model Prediction

Prediction: Non-Carcinogen
Probability: 0.184
Enrichment: 0.626
Bayesian Score: -6.04
Mahalanobis Distance: 13.2
Mahalanobis Distance p-value: 0.00015

Prediction: Positive if the Bayesian score is above the estimated best cutoff value from minimizing the false positive and false negative rate.
Probability: The esimated probability that the sample is in the positive category. This assumes that the Bayesian score follows a normal distribution and is different from the prediction using a cutoff.
Enrichment: An estimate of enrichment, that is, the increased likelihood (versus random) of this sample being in the category.
Bayesian Score: The standard Laplacian-modified Bayesian score.
Mahalanobis Distance: The Mahalanobis distance (MD) is the distance to the center of the training data. The larger the MD, the less trustworthy the prediction.
Mahalanobis Distance p-value: The p-value gives the fraction of training data with an MD greater than or equal to the one for the given sample, assuming normally distributed data. The smaller the p-value, the less trustworthy the prediciton. For highly non-normal X properties (e.g., fingerprints), the MD p-value is wildly inaccurate.

Model Applicability

Unknown features are fingerprint features in the query molecule, but not found or appearing too infrequently in the training set.

1. All properties and OPS components are within expected ranges.

Feature Contribution

Top features for positive contribution

Fingerprint	Bit/Smiles	Feature Structure	Score	Carcinogen in training set
FCFP_6	-387072142	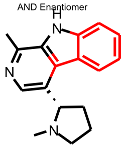 [*]:[c]1:[*]:[*]:[c]2:[cH]:[cH]:[cH]:[cH]:[c]:1:2	0.477	4 out of 8

FCFP_6	-1462709112	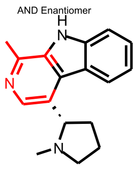 [*][c]1:[*]:[c](:[*]) :[c](C):n:[cH]:1	0.367	5 out of 12
FCFP_6	-1320007763	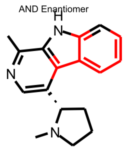 [*]:[c]1:[*]:[*]:[c]2 :[*]:[cH]:[cH]:[cH]: [c]:1:2	0.348	6 out of 15

Top Features for negative contribution

Fingerprint	Bit/Smiles	Feature Structure	Score	Carcinogen in training set
FCFP_6	309602933	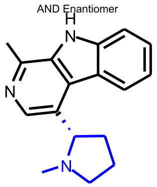 [*][C@@H]1CCCN1C	-1.4	1 out of 27
FCFP_6	155061250	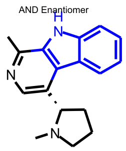 [*]:[c]1:[nH]:[c]2:[c H]:[cH]:[cH]:[cH]:[c]:2:[c]:1:[*]	-0.719	0 out of 4
FCFP_6	240509252	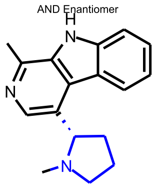 [*][C@@H]1CCCN1[*]	-0.596	1 out of 10

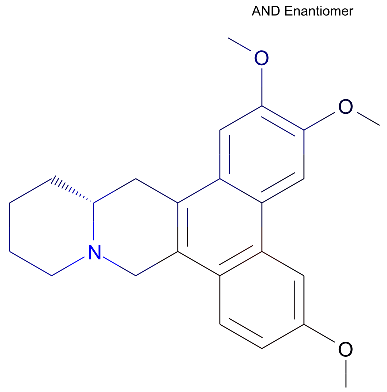

$C_{24}H_{27}NO_3$
Molecular Weight: 377.47608
ALogP: 4.691
Rotatable Bonds: 3
Acceptors: 4
Donors: 0

Model Prediction

Prediction: Non-Carcinogen
Probability: 0.171
Enrichment: 0.58
Bayesian Score: -7.07
Mahalanobis Distance: 12.8
Mahalanobis Distance p-value: 0.00048

Prediction: Positive if the Bayesian score is above the estimated best cutoff value from minimizing the false positive and false negative rate.
Probability: The esimated probability that the sample is in the positive category. This assumes that the Bayesian score follows a normal distribution and is different from the prediction using a cutoff.
Enrichment: An estimate of enrichment, that is, the increased likelihood (versus random) of this sample being in the category.
Bayesian Score: The standard Laplacian-modified Bayesian score.
Mahalanobis Distance: The Mahalanobis distance (MD) is the distance to the center of the training data. The larger the MD, the less trustworthy the prediction.
Mahalanobis Distance p-value: The p-value gives the fraction of training data with an MD greater than or equal to the one for the given sample, assuming normally distributed data. The smaller the p-value, the less trustworthy the prediciton. For highly non-normal X properties (e.g., fingerprints), the MD p-value is wildly inaccurate.

Structural Similar Compounds

Name	Ethynodiol	Loratidine	Chlorpromazine
Structure	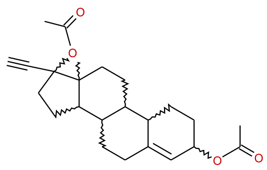	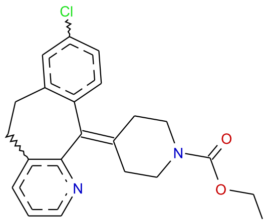	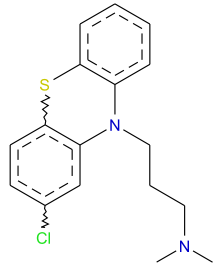
Actual Endpoint	Carcinogen	Carcinogen	Non-Carcinogen
Predicted Endpoint	Carcinogen	Carcinogen	Non-Carcinogen
Distance	0.580	0.583	0.583
Reference	US FDA (Centre for Drug Eval.& Res./Off. Testing & Res.) Sept. 1997	US FDA (Centre for Drug Eval.& Res./Off. Testing & Res.) Sept. 1997	US FDA (Centre for Drug Eval.& Res./Off. Testing & Res.) Sept. 1997

Model Applicability

Unknown features are fingerprint features in the query molecule, but not found or appearing too infrequently in the training set.

1. All properties and OPS components are within expected ranges.

Feature Contribution

Top features for positive contribution				
**Fingerprint**	**Bit/Smiles**	**Feature Structure**	**Score**	**Carcinogen in training set**
FCFP_6	-1550599384	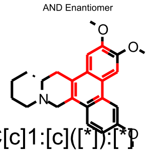 [*]C[c]1:[c]([*]):[*]:[c](:[*]):[c]2:[cH]:[*]:[c]([*]):[cH]:[c]:1:2	0.46	1 out of 1

FCFP_6	-1320007763	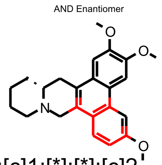 [*]:[c]1:[*]:[*]:[c]2:[*]:[cH]:[cH]:[cH]:[c]:1:2	0.348	6 out of 15
FCFP_6	277509858	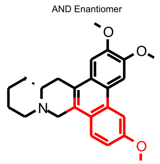 [*][c](:[*]):[c]1:[cH]:[cH]:[c](OC):[cH]:[c]:1:[*]	0.271	1 out of 2
**Top Features for negative contribution**				
**Fingerprint**	**Bit/Smiles**	**Feature Structure**	**Score**	**Carcinogen in training set**
FCFP_6	309602933	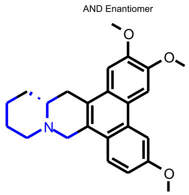 [*][C@@H]1CCCN1C	-1.4	1 out of 27
FCFP_6	-2000873681	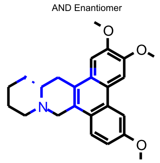 [*]C[C@@H]1C[c](:[c](:[*]):[*]):[c](:[*])[*]N1[*]	-0.719	0 out of 4
FCFP_6	1674955425	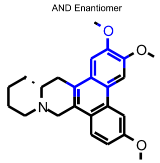 [*]O[c]1:[cH]:[c](:[c]([*]):[*]):[c](:[*]):[*]:[c]:1[*]	-0.719	0 out of 4

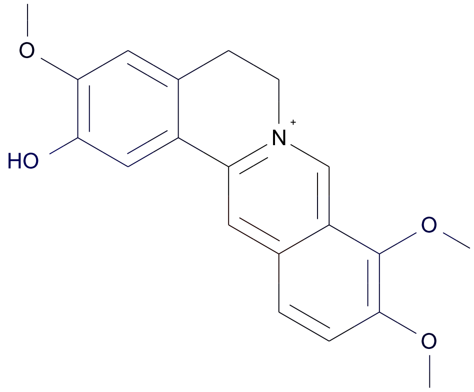

$C_{20}H_{20}NO_4$
Molecular Weight: 338.3771
ALogP: 3.936
Rotatable Bonds: 3
Acceptors: 4
Donors: 1

Model Prediction

Prediction: Non-Carcinogen
Probability: 0.2
Enrichment: 0.681
Bayesian Score: -4.95
Mahalanobis Distance: 13.5
Mahalanobis Distance p-value: 5.34e-005

Prediction: Positive if the Bayesian score is above the estimated best cutoff value from minimizing the false positive and false negative rate.
Probability: The esimated probability that the sample is in the positive category. This assumes that the Bayesian score follows a normal distribution and is different from the prediction using a cutoff.
Enrichment: An estimate of enrichment, that is, the increased likelihood (versus random) of this sample being in the category.
Bayesian Score: The standard Laplacian-modified Bayesian score.
Mahalanobis Distance: The Mahalanobis distance (MD) is the distance to the center of the training data. The larger the MD, the less trustworthy the prediction.
Mahalanobis Distance p-value: The p-value gives the fraction of training data with an MD greater than or equal to the one for the given sample, assuming normally distributed data. The smaller the p-value, the less trustworthy the prediciton. For highly non-normal X properties (e.g., fingerprints), the MD p-value is wildly inaccurate.

Structural Similar Compounds

Name	Indomethacin	Paroxetine	Nafenopin
Structure	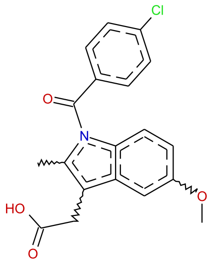	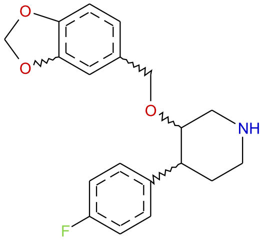	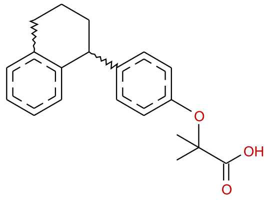
Actual Endpoint	Non-Carcinogen	Non-Carcinogen	Carcinogen
Predicted Endpoint	Non-Carcinogen	Non-Carcinogen	Carcinogen
Distance	0.529	0.559	0.590
Reference	US FDA (Centre for Drug Eval.& Res./Off. Testing & Res.) Sept. 1997	US FDA (Centre for Drug Eval.& Res./Off. Testing & Res.) Sept. 1997	US FDA (Centre for Drug Eval.& Res./Off. Testing & Res.) Sept. 1997

Model Applicability

Unknown features are fingerprint features in the query molecule, but not found or appearing too infrequently in the training set.

1. All properties and OPS components are within expected ranges.
2. Unknown FCFP_2 feature: 24: [*][n+](:[*]):[*]
3. Unknown FCFP_2 feature: 414371600: [*]C[n+](:[c]([*]):[*]):c:[*]
4. Unknown FCFP_2 feature: -150573739: [*]CC[n+](:[*]):[*]
5. Unknown FCFP_2 feature: -1861407456: [*][n+](:[*]):[c]([c](:[*]):[*]):c:[*]
6. Unknown FCFP_2 feature: 1618392993: [*][n+](:[*]):c:[c](:[*]):[*]

Feature Contribution

Top features for positive contribution				
Fingerprint	Bit/Smiles	Feature Structure	Score	Carcinogen in training set

FCFP_6	-105186863	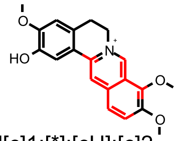 [*][c]1:[*]:[cH]:[c]2:[c]([*]):[*]:[cH]:[cH]:[c]:2:[cH]:1	0.38	2 out of 4
FCFP_6	-1320007763	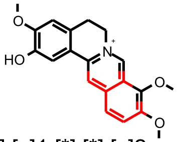 [*]:[c]1:[*]:[*]:[c]2:[*]:[cH]:[cH]:[cH]:[c]:1:2	0.348	6 out of 15
FCFP_6	1676877079	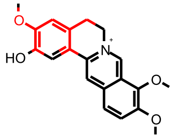 [*]C[c]1:[cH]:[c](O[*]):[c]([*]):[*]:[c]:1[*]	0.333	7 out of 18
**Top Features for negative contribution**				
**Fingerprint**	**Bit/Smiles**	**Feature Structure**	**Score**	**Carcinogen in training set**
FCFP_6	1028934530	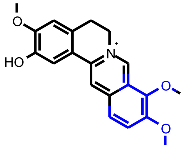 [*]O[c]1:[cH]:[*]:[c](:[*]):[cH]:[c]:1OC	-0.596	1 out of 10
FCFP_6	523826990	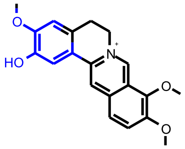 [*]O[c]1:[cH]:[*]:[c]([*]):[cH]:[c]:1O	-0.423	0 out of 2

FCFP_6	907007053	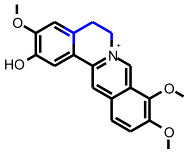 [*]C([*])C[c](:[*]):[*]	-0.366	11 out of 62

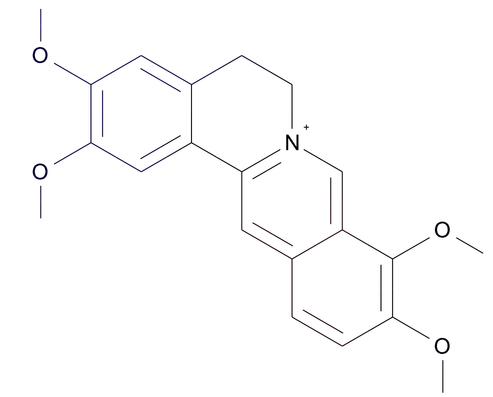

$C_{21}H_{22}NO_4$
Molecular Weight: 352.40368
ALogP: 4.161
Rotatable Bonds: 4
Acceptors: 4
Donors: 0

Structural Similar Compounds

Name	Chlorpromazine	Ethynodiol	Risperidone
Structure	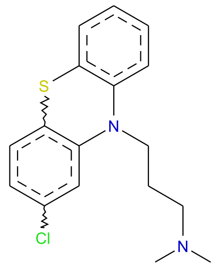	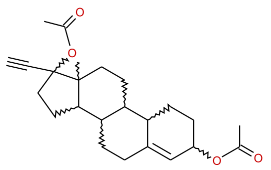	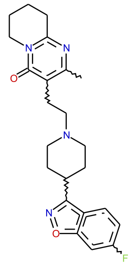
Actual Endpoint	Non-Carcinogen	Carcinogen	Non-Carcinogen
Predicted Endpoint	Non-Carcinogen	Carcinogen	Non-Carcinogen
Distance	0.583	0.587	0.612
Reference	US FDA (Centre for Drug Eval.& Res./Off. Testing & Res.) Sept. 1997	US FDA (Centre for Drug Eval.& Res./Off. Testing & Res.) Sept. 1997	US FDA (Centre for Drug Eval.& Res./Off. Testing & Res.) Sept. 1997

Model Prediction

Prediction: Carcinogen
Probability: 0.266
Enrichment: 0.904
Bayesian Score: -1.57
Mahalanobis Distance: 11.1
Mahalanobis Distance p-value: 0.057

Prediction: Positive if the Bayesian score is above the estimated best cutoff value from minimizing the false positive and false negative rate.
Probability: The esimated probability that the sample is in the positive category. This assumes that the Bayesian score follows a normal distribution and is different from the prediction using a cutoff.
Enrichment: An estimate of enrichment, that is, the increased likelihood (versus random) of this sample being in the category.
Bayesian Score: The standard Laplacian-modified Bayesian score.
Mahalanobis Distance: The Mahalanobis distance (MD) is the distance to the center of the training data. The larger the MD, the less trustworthy the prediction.
Mahalanobis Distance p-value: The p-value gives the fraction of training data with an MD greater than or equal to the one for the given sample, assuming normally distributed data. The smaller the p-value, the less trustworthy the prediciton. For highly non-normal X properties (e.g., fingerprints), the MD p-value is wildly inaccurate.

Model Applicability

Unknown features are fingerprint features in the query molecule, but not found or appearing too infrequently in the training set.

1. All properties and OPS components are within expected ranges.
2. Unknown FCFP_2 feature: 24: [*][n+](:[*]):[*]
3. Unknown FCFP_2 feature: 414371600: [*]C[n+](:[c]([*]):[*]):c:[*]
4. Unknown FCFP_2 feature: -150573739: [*]CC[n+](:[*]):[*]
5. Unknown FCFP_2 feature: -1861407456: [*][n+](:[*]):[c]([c](:[*]):[*]):c:[*]
6. Unknown FCFP_2 feature: 1618392993: [*][n+](:[*]):c:[c](:[*]):[*]

Feature Contribution

Top features for positive contribution				
Fingerprint	Bit/Smiles	Feature Structure	Score	Carcinogen in training set

FCFP_6	-105186863	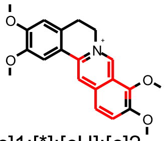 [*][c]1:[*]:[cH]:[c]2:[c]([*]):[*]:[cH]:[cH]:[c]:2:[cH]:1	0.38	2 out of 4
FCFP_6	-1320007763	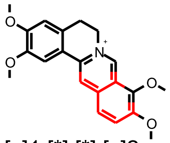 [*]:[c]1:[*]:[*]:[c]2:[*]:[cH]:[cH]:[cH]:[c]:1:2	0.348	6 out of 15
FCFP_6	1676877079	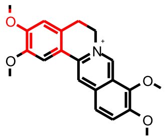 [*]C[c]1:[cH]:[c](O[*]):[c]([*]):[*]:[c]:1[*]	0.333	7 out of 18

Top Features for negative contribution

Fingerprint	Bit/Smiles	Feature Structure	Score	Carcinogen in training set
FCFP_6	1028934530	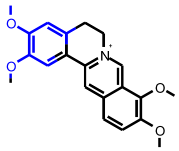 [*]O[c]1:[cH]:[*]:[c](:[*]):[cH]:[c]:1OC	-0.596	1 out of 10
FCFP_6	-1405834164	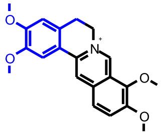 [*]C[c]1:[cH]:[c](OC):[c](OC):[cH]:[c]:1[*]	-0.423	0 out of 2

FCFP_6	907007053	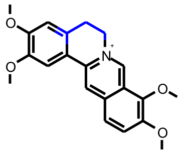<br>[*]C([*])C[c](:[*]):[<br>*]	-0.366	11 out of 62

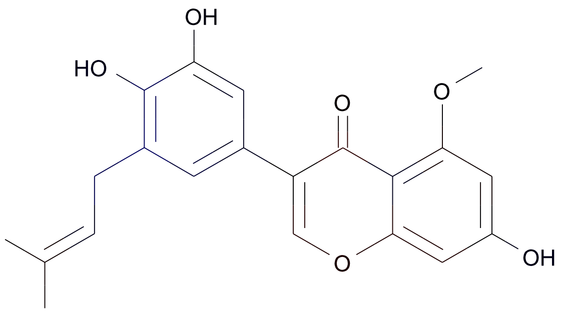

$C_{21}H_{20}O_6$
Molecular Weight: 368.3799
ALogP: 3.98
Rotatable Bonds: 4
Acceptors: 6
Donors: 3

Model Prediction

Prediction: Non-Carcinogen
Probability: 0.24
Enrichment: 0.816
Bayesian Score: -2.75
Mahalanobis Distance: 12.6
Mahalanobis Distance p-value: 0.00116

Prediction: Positive if the Bayesian score is above the estimated best cutoff value from minimizing the false positive and false negative rate.
Probability: The esimated probability that the sample is in the positive category. This assumes that the Bayesian score follows a normal distribution and is different from the prediction using a cutoff.
Enrichment: An estimate of enrichment, that is, the increased likelihood (versus random) of this sample being in the category.
Bayesian Score: The standard Laplacian-modified Bayesian score.
Mahalanobis Distance: The Mahalanobis distance (MD) is the distance to the center of the training data. The larger the MD, the less trustworthy the prediction.
Mahalanobis Distance p-value: The p-value gives the fraction of training data with an MD greater than or equal to the one for the given sample, assuming normally distributed data. The smaller the p-value, the less trustworthy the prediciton. For highly non-normal X properties (e.g., fingerprints), the MD p-value is wildly inaccurate.

Structural Similar Compounds

Name	Torsemide	Ursodiol	Nedocromil
Structure	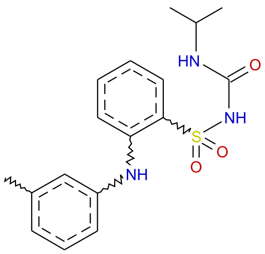	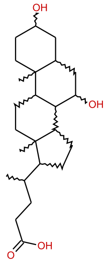	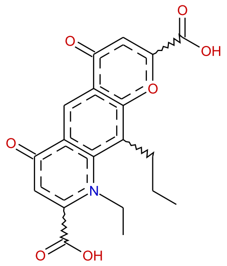
Actual Endpoint	Non-Carcinogen	Non-Carcinogen	Non-Carcinogen
Predicted Endpoint	Non-Carcinogen	Carcinogen	Non-Carcinogen
Distance	0.656	0.668	0.701
Reference	US FDA (Centre for Drug Eval.& Res./Off. Testing & Res.) Sept. 1997	US FDA (Centre for Drug Eval.& Res./Off. Testing & Res.) Sept. 1997	US FDA (Centre for Drug Eval.& Res./Off. Testing & Res.) Sept. 1997

Model Applicability

Unknown features are fingerprint features in the query molecule, but not found or appearing too infrequently in the training set.

1. All properties and OPS components are within expected ranges.

Feature Contribution

Top features for positive contribution

Fingerprint	Bit/Smiles	Feature Structure	Score	Carcinogen in training set
FCFP_6	451847724	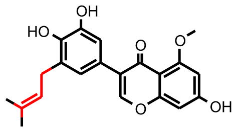 [*]CC=C([*])[*]	0.479	21 out of 48

FCFP_6	1247585207	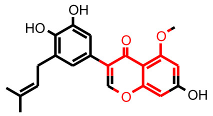 [*]O[c]1:[cH]:[*]:[cH]:[c]2O[*]=C([*])C(= O)[c]:1:2	0.46	1 out of 1
FCFP_6	-628297815	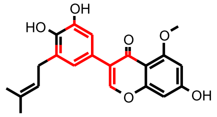 [*]\C=C(/C(=[*])[*])\ [c]1:[cH]:[c]([*]):[*]:[c]([*]):[cH]:1	0.38	2 out of 4

Top Features for negative contribution

**Fingerprint**	**Bit/Smiles**	**Feature Structure**	**Score**	**Carcinogen in training set**
FCFP_6	-451251206	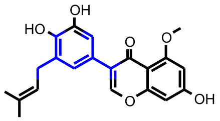 [*]C[c]1:[cH]:[c](:[c H]:[*]:[c]:1[*])C(=[*])[*]	-0.731	1 out of 12
FCFP_6	-1549192822	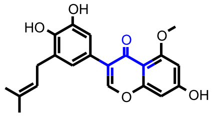 [*]C(=[*])C(=O)[c](:[*]):[*]	-0.489	3 out of 21
FCFP_6	668297787	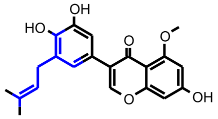 [*]C(=CC[c](:[cH]:[*]):[c]([*]):[*])[*]	-0.423	0 out of 2

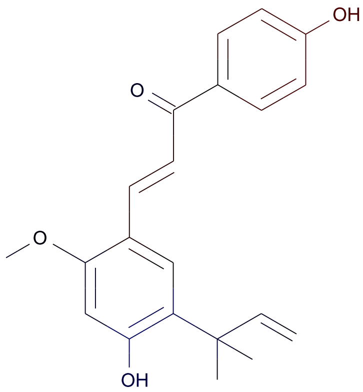

$C_{21}H_{22}O_4$
Molecular Weight: 338.39698
ALogP: 4.667
Rotatable Bonds: 6
Acceptors: 4
Donors: 2

Model Prediction

Prediction: Non-Carcinogen
Probability: 0.233
Enrichment: 0.792
Bayesian Score: -3.11
Mahalanobis Distance: 12.1
Mahalanobis Distance p-value: 0.00493

Prediction: Positive if the Bayesian score is above the estimated best cutoff value from minimizing the false positive and false negative rate.
Probability: The esimated probability that the sample is in the positive category. This assumes that the Bayesian score follows a normal distribution and is different from the prediction using a cutoff.
Enrichment: An estimate of enrichment, that is, the increased likelihood (versus random) of this sample being in the category.
Bayesian Score: The standard Laplacian-modified Bayesian score.
Mahalanobis Distance: The Mahalanobis distance (MD) is the distance to the center of the training data. The larger the MD, the less trustworthy the prediction.
Mahalanobis Distance p-value: The p-value gives the fraction of training data with an MD greater than or equal to the one for the given sample, assuming normally distributed data. The smaller the p-value, the less trustworthy the prediciton. For highly non-normal X properties (e.g., fingerprints), the MD p-value is wildly inaccurate.

Structural Similar Compounds

Name	Diclofenac	Penbutalol	Indomethacin
Structure	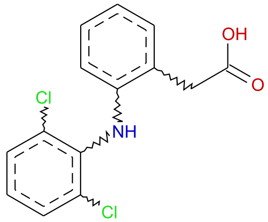	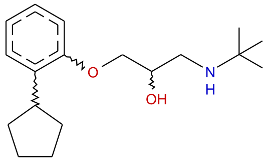	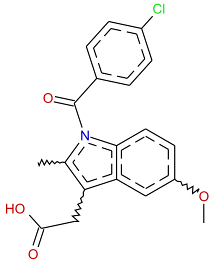
Actual Endpoint	Non-Carcinogen	Non-Carcinogen	Non-Carcinogen
Predicted Endpoint	Non-Carcinogen	Non-Carcinogen	Non-Carcinogen
Distance	0.617	0.621	0.629
Reference	US FDA (Centre for Drug Eval.& Res./Off. Testing & Res.) Sept. 1997	US FDA (Centre for Drug Eval.& Res./Off. Testing & Res.) Sept. 1997	US FDA (Centre for Drug Eval.& Res./Off. Testing & Res.) Sept. 1997

Model Applicability

Unknown features are fingerprint features in the query molecule, but not found or appearing too infrequently in the training set.

1. All properties and OPS components are within expected ranges.

Feature Contribution

Top features for positive contribution

Fingerprint	Bit/Smiles	Feature Structure	Score	Carcinogen in training set
FCFP_6	-146015125	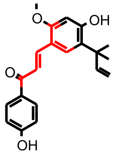 [*]C(=[*])\C=C\[c](:[cH]:[*]):[c]([*]):[*]	0.676	2 out of 2

FCFP_6	-1066794953	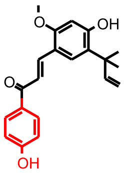 [*][c]1:[cH]:[cH]:[c](O):[cH]:[cH]:1	0.668	3 out of 4
FCFP_6	-1847351220	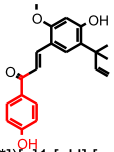 [*]C(=[*])[c]1:[cH]:[cH]:[c](O):[cH]:[cH]:1	0.547	3 out of 5

Top Features for negative contribution

Fingerprint	Bit/Smiles	Feature Structure	Score	Carcinogen in training set
FCFP_6	-451251206	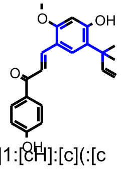 [*]C[c]1:[cH]:[c](:[cH]:[*]:[c]:1[*])C(=[*])[*]	-0.731	1 out of 12
FCFP_6	129344189	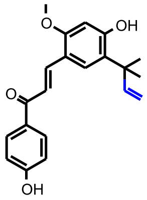 [*]C=C	-0.719	0 out of 4
FCFP_6	-1549192822	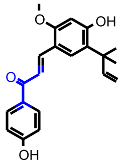 [*]C(=[*])C(=O)[c](:[*]):[*]	-0.489	3 out of 21

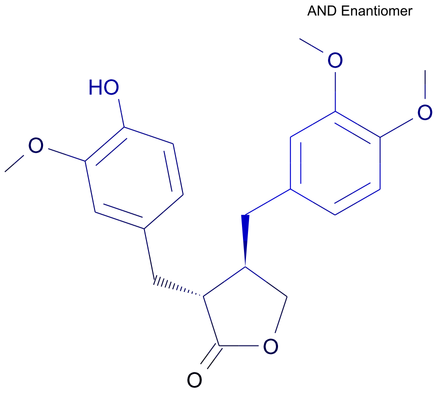

$C_{21}H_{24}O_6$
Molecular Weight: 372.41166
ALogP: 3.743
Rotatable Bonds: 7
Acceptors: 6
Donors: 1

Structural Similar Compounds

Name	Felodipine	Lovastatin	Diltiazem
Structure	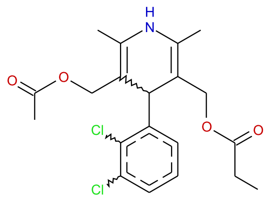	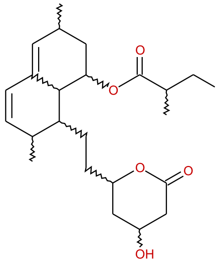	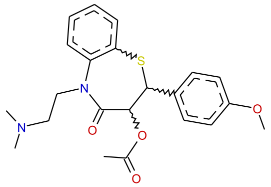
Actual Endpoint	Non-Carcinogen	Carcinogen	Non-Carcinogen
Predicted Endpoint	Non-Carcinogen	Carcinogen	Non-Carcinogen
Distance	0.570	0.577	0.580
Reference	US FDA (Centre for Drug Eval.& Res./Off. Testing & Res.) Sept. 1997	US FDA (Centre for Drug Eval.& Res./Off. Testing & Res.) Sept. 1997	US FDA (Centre for Drug Eval.& Res./Off. Testing & Res.) Sept. 1997

Model Prediction

Prediction: Non-Carcinogen
Probability: 0.147
Enrichment: 0.498
Bayesian Score: -9.34
Mahalanobis Distance: 12
Mahalanobis Distance p-value: 0.00584

Prediction: Positive if the Bayesian score is above the estimated best cutoff value from minimizing the false positive and false negative rate.
Probability: The esimated probability that the sample is in the positive category. This assumes that the Bayesian score follows a normal distribution and is different from the prediction using a cutoff.
Enrichment: An estimate of enrichment, that is, the increased likelihood (versus random) of this sample being in the category.
Bayesian Score: The standard Laplacian-modified Bayesian score.
Mahalanobis Distance: The Mahalanobis distance (MD) is the distance to the center of the training data. The larger the MD, the less trustworthy the prediction.
Mahalanobis Distance p-value: The p-value gives the fraction of training data with an MD greater than or equal to the one for the given sample, assuming normally distributed data. The smaller the p-value, the less trustworthy the prediciton. For highly non-normal X properties (e.g., fingerprints), the MD p-value is wildly inaccurate.

Model Applicability

Unknown features are fingerprint features in the query molecule, but not found or appearing too infrequently in the training set.

1. All properties and OPS components are within expected ranges.

Feature Contribution

Top features for positive contribution				
Fingerprint	Bit/Smiles	Feature Structure	Score	Carcinogen in training set
FCFP_6	-16971222	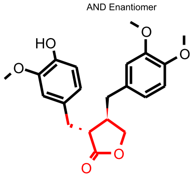 [*]C[C@@H]1[C@@H]([*])COC1=O	0.439	3 out of 6

FCFP_6	-1043339860	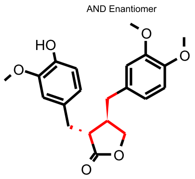 [*]C[C@H]1C[*][*][C@@H]1[*]	0.383	24 out of 61
FCFP_6	1676877079	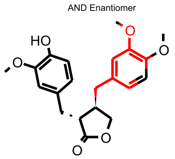 [*]C[c]1:[cH]:[c](O[*]):[c]([*]):[*]:[c]:1[*]	0.333	7 out of 18

Top Features for negative contribution

Fingerprint	Bit/Smiles	Feature Structure	Score	Carcinogen in training set
FCFP_6	-497728148	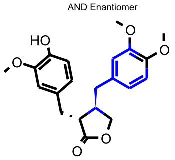 [*]C([*])C[c]1:[cH]:[cH]:[*]:[c]([*]):[cH]:1	-0.96	2 out of 26
FCFP_6	1588282714	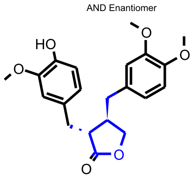 [*]C[C@H]1COC(=[*])[C@@H]1[*]	-0.839	0 out of 5
FCFP_6	-1038421835	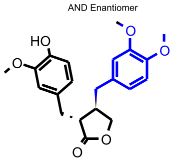 [*]C[c]1:[cH]:[cH]:[c](OC):[c](O[*]):[cH]:1	-0.719	0 out of 4

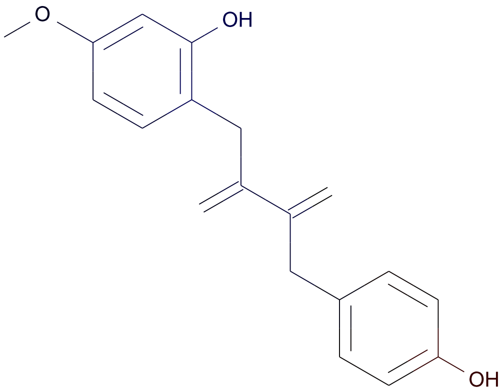

$C_{19}H_{20}O_3$
Molecular Weight: 296.3603
ALogP: 4.784
Rotatable Bonds: 6
Acceptors: 3
Donors: 2

Structural Similar Compounds

Name	Diethylstilbesterol	Penbutalol	Diclofenac
Structure	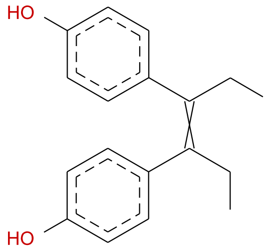	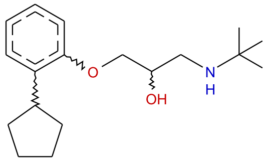	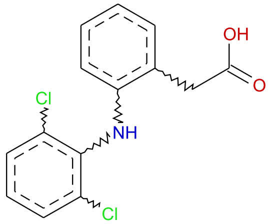
Actual Endpoint	Carcinogen	Non-Carcinogen	Non-Carcinogen
Predicted Endpoint	Carcinogen	Non-Carcinogen	Non-Carcinogen
Distance	0.533	0.573	0.575
Reference	US FDA (Centre for Drug Eval.& Res./Off. Testing & Res.) Sept. 1997	US FDA (Centre for Drug Eval.& Res./Off. Testing & Res.) Sept. 1997	US FDA (Centre for Drug Eval.& Res./Off. Testing & Res.) Sept. 1997

Model Prediction

Prediction: Non-Carcinogen
Probability: 0.24
Enrichment: 0.815
Bayesian Score: -2.77
Mahalanobis Distance: 11
Mahalanobis Distance p-value: 0.074

Prediction: Positive if the Bayesian score is above the estimated best cutoff value from minimizing the false positive and false negative rate.
Probability: The esimated probability that the sample is in the positive category. This assumes that the Bayesian score follows a normal distribution and is different from the prediction using a cutoff.
Enrichment: An estimate of enrichment, that is, the increased likelihood (versus random) of this sample being in the category.
Bayesian Score: The standard Laplacian-modified Bayesian score.
Mahalanobis Distance: The Mahalanobis distance (MD) is the distance to the center of the training data. The larger the MD, the less trustworthy the prediction.
Mahalanobis Distance p-value: The p-value gives the fraction of training data with an MD greater than or equal to the one for the given sample, assuming normally distributed data. The smaller the p-value, the less trustworthy the prediciton. For highly non-normal X properties (e.g., fingerprints), the MD p-value is wildly inaccurate.

Model Applicability

Unknown features are fingerprint features in the query molecule, but not found or appearing too infrequently in the training set.

1. All properties and OPS components are within expected ranges.

Feature Contribution

Top features for positive contribution				
Fingerprint	Bit/Smiles	Feature Structure	Score	Carcinogen in training set
FCFP_6	-1066794953	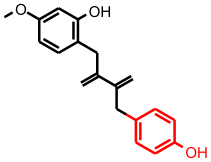 [*][c]1:[cH]:[cH]:[c](O):[cH]:[cH]:1	0.668	3 out of 4

FCFP_6	-1847351220	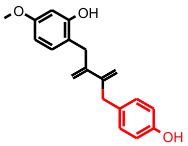 [*]C(=[*])[c]1:[cH]:[cH]:[c](O):[cH]:[cH]:1	0.547	3 out of 5
FCFP_6	-158888774	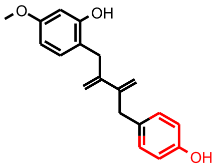 [*][c]1:[*]:[c]([*]):[cH]:[c](O):[cH]:1	0.367	5 out of 12
**Top Features for negative contribution**				
**Fingerprint**	**Bit/Smiles**	**Feature Structure**	**Score**	**Carcinogen in training set**
FCFP_6	-497728148	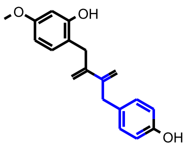 [*]C([*])C[c]1:[cH]:[cH]:[*]:[c]([*]):[cH]:1	-0.96	2 out of 26
FCFP_6	129344189	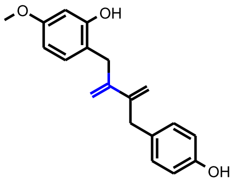 [*]C=C	-0.719	0 out of 4
FCFP_6	-1604301295	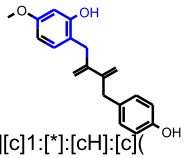 [*][c]1:[*]:[cH]:[c](:[c](O):[cH]:1)C([*])([*])[*]	-0.445	2 out of 14

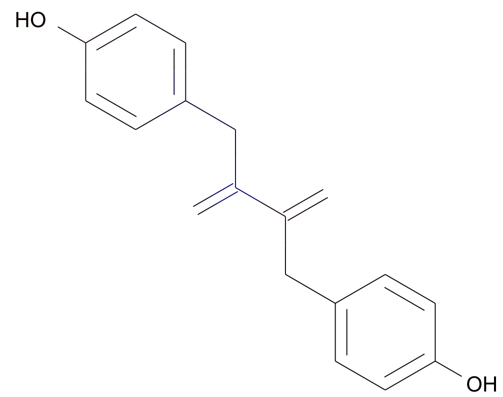

$C_{18}H_{18}O_2$
Molecular Weight: 266.33432
ALogP: 4.8
Rotatable Bonds: 5
Acceptors: 2
Donors: 2

Model Prediction

Prediction: Carcinogen
Probability: 0.27
Enrichment: 0.918
Bayesian Score: -1.4
Mahalanobis Distance: 9.3
Mahalanobis Distance p-value: 0.696

Prediction: Positive if the Bayesian score is above the estimated best cutoff value from minimizing the false positive and false negative rate.
Probability: The esimated probability that the sample is in the positive category. This assumes that the Bayesian score follows a normal distribution and is different from the prediction using a cutoff.
Enrichment: An estimate of enrichment, that is, the increased likelihood (versus random) of this sample being in the category.
Bayesian Score: The standard Laplacian-modified Bayesian score.
Mahalanobis Distance: The Mahalanobis distance (MD) is the distance to the center of the training data. The larger the MD, the less trustworthy the prediction.
Mahalanobis Distance p-value: The p-value gives the fraction of training data with an MD greater than or equal to the one for the given sample, assuming normally distributed data. The smaller the p-value, the less trustworthy the prediciton. For highly non-normal X properties (e.g., fingerprints), the MD p-value is wildly inaccurate.

Structural Similar Compounds

Name	Diethylstilbesterol	Hexylresorcinol	Diclofenac
Structure	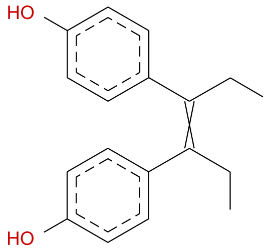	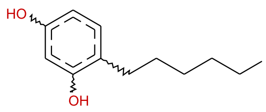	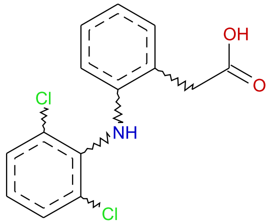
Actual Endpoint	Carcinogen	Non-Carcinogen	Non-Carcinogen
Predicted Endpoint	Carcinogen	Non-Carcinogen	Non-Carcinogen
Distance	0.375	0.495	0.568
Reference	US FDA (Centre for Drug Eval.& Res./Off. Testing & Res.) Sept. 1997	US FDA (Centre for Drug Eval.& Res./Off. Testing & Res.) Sept. 1997	US FDA (Centre for Drug Eval.& Res./Off. Testing & Res.) Sept. 1997

Model Applicability

Unknown features are fingerprint features in the query molecule, but not found or appearing too infrequently in the training set.

1. All properties and OPS components are within expected ranges.

Feature Contribution

Top features for positive contribution

Fingerprint	Bit/Smiles	Feature Structure	Score	Carcinogen in training set
FCFP_6	-1066794953	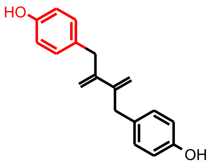 [*][c]1:[cH]:[cH]:[c](O):[cH]:[cH]:1	0.668	3 out of 4

FCFP_6	-1847351220	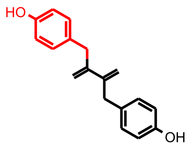 [*]C(=[*])[c]1:[cH]:[cH]:[c](O):[cH]:[cH]:1	0.547	3 out of 5
FCFP_6	-158888774	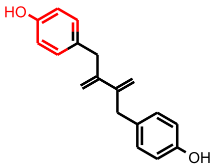 [*][c]1:[*]:[c]([*]):[cH]:[c](O):[cH]:1	0.367	5 out of 12

Top Features for negative contribution

Fingerprint	Bit/Smiles	Feature Structure	Score	Carcinogen in training set
FCFP_6	-497728148	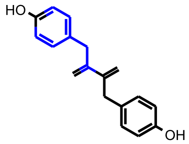 [*]C([*])C[c]1:[cH]:[cH]:[*]:[c]([*]):[cH]:1	-0.96	2 out of 26
FCFP_6	129344189	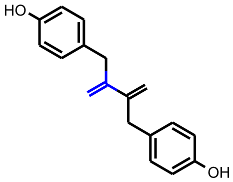 [*]C=C	-0.719	0 out of 4
FCFP_6	1985639687	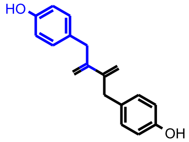 [*]C([*])C[c]1:[cH]:[cH]:[c](O):[c]([*]):[cH]:1	-0.423	0 out of 2

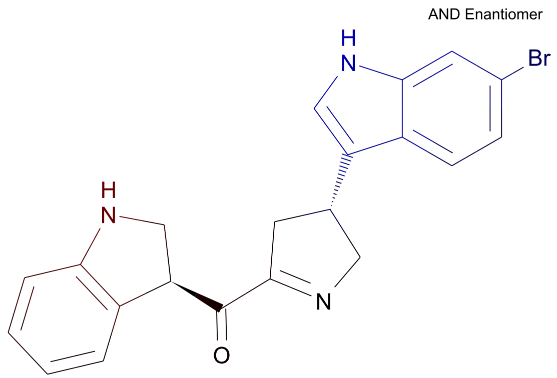

$C_{21}H_{18}BrN_3O$
Molecular Weight: 408.29112
ALogP: 3.919
Rotatable Bonds: 3
Acceptors: 3
Donors: 2

Model Prediction

Prediction: Non-Carcinogen
Probability: 0.217
Enrichment: 0.739
Bayesian Score: -3.95
Mahalanobis Distance: 15.7
Mahalanobis Distance p-value: 3.14e-009

Prediction: Positive if the Bayesian score is above the estimated best cutoff value from minimizing the false positive and false negative rate.
Probability: The esimated probability that the sample is in the positive category. This assumes that the Bayesian score follows a normal distribution and is different from the prediction using a cutoff.
Enrichment: An estimate of enrichment, that is, the increased likelihood (versus random) of this sample being in the category.
Bayesian Score: The standard Laplacian-modified Bayesian score.
Mahalanobis Distance: The Mahalanobis distance (MD) is the distance to the center of the training data. The larger the MD, the less trustworthy the prediction.
Mahalanobis Distance p-value: The p-value gives the fraction of training data with an MD greater than or equal to the one for the given sample, assuming normally distributed data. The smaller the p-value, the less trustworthy the prediciton. For highly non-normal X properties (e.g., fingerprints), the MD p-value is wildly inaccurate.

Structural Similar Compounds

Name	Mefloquine	Lorazepam	Diclofenac
Structure	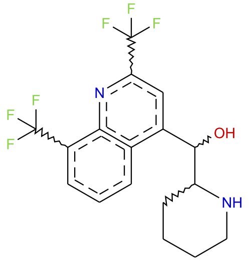	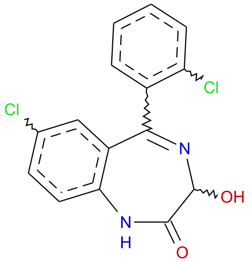	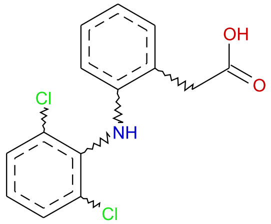
Actual Endpoint	Non-Carcinogen	Non-Carcinogen	Non-Carcinogen
Predicted Endpoint	Non-Carcinogen	Non-Carcinogen	Non-Carcinogen
Distance	0.545	0.604	0.615
Reference	US FDA (Centre for Drug Eval.& Res./Off. Testing & Res.) Sept. 1997	US FDA (Centre for Drug Eval.& Res./Off. Testing & Res.) Sept. 1997	US FDA (Centre for Drug Eval.& Res./Off. Testing & Res.) Sept. 1997

Model Applicability

Unknown features are fingerprint features in the query molecule, but not found or appearing too infrequently in the training set.

1. All properties and OPS components are within expected ranges.

Feature Contribution

Top features for positive contribution

Fingerprint	Bit/Smiles	Feature Structure	Score	Carcinogen in training set
FCFP_6	1294255210	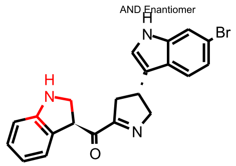 [*]:[c]1:[*][*]CN1	0.441	12 out of 28

FCFP_6	-773983804	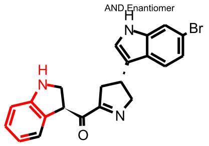<br>[*]1[*][c]2:[*]:[cH]:[cH]:[cH]:[c]:2N1	0.409	10 out of 24
FCFP_6	590925877	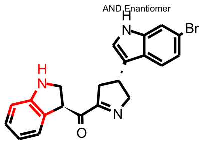<br>[*]:[cH]:[c]1N[*][*][c]:1:[*]	0.369	13 out of 33

Top Features for negative contribution

**Fingerprint**	**Bit/Smiles**	**Feature Structure**	**Score**	**Carcinogen in training set**
FCFP_6	1618184456	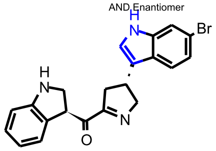<br>[*][c]1:[*]:[*]:[nH]:[cH]:1	-0.719	0 out of 4
FCFP_6	-1828565903	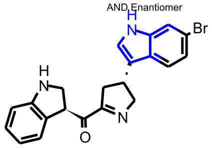<br>[*][c]1:[cH]:[nH]:[c](:[cH]:[*]):[c]:1:[*]	-0.719	0 out of 4
FCFP_6	-2128357774	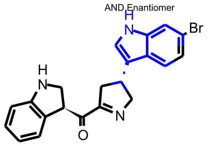<br>[*]C([*])[c]1:[cH]:[nH]:[c]2:[cH]:[c]([*]):[*]:[cH]:[c]:1:2	-0.582	0 out of 3

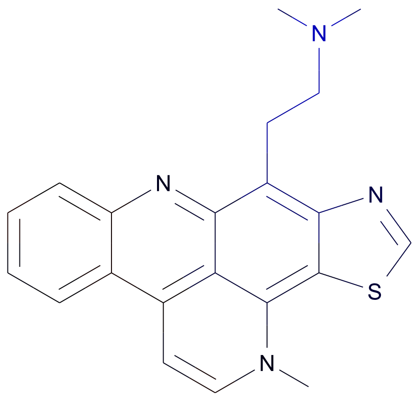

$C_{21}H_{20}N_4S$
Molecular Weight: 360.4753
ALogP: 3.682
Rotatable Bonds: 3
Acceptors: 4
Donors: 0

Model Prediction

Prediction: Non-Carcinogen
Probability: 0.188
Enrichment: 0.638
Bayesian Score: -5.79
Mahalanobis Distance: 13.8
Mahalanobis Distance p-value: 1.44e-005

Prediction: Positive if the Bayesian score is above the estimated best cutoff value from minimizing the false positive and false negative rate.
Probability: The esimated probability that the sample is in the positive category. This assumes that the Bayesian score follows a normal distribution and is different from the prediction using a cutoff.
Enrichment: An estimate of enrichment, that is, the increased likelihood (versus random) of this sample being in the category.
Bayesian Score: The standard Laplacian-modified Bayesian score.
Mahalanobis Distance: The Mahalanobis distance (MD) is the distance to the center of the training data. The larger the MD, the less trustworthy the prediction.
Mahalanobis Distance p-value: The p-value gives the fraction of training data with an MD greater than or equal to the one for the given sample, assuming normally distributed data. The smaller the p-value, the less trustworthy the prediciton. For highly non-normal X properties (e.g., fingerprints), the MD p-value is wildly inaccurate.

Structural Similar Compounds

Name	Risperidone	Chlormadinone	Indomethacin
Structure	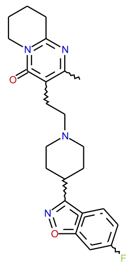	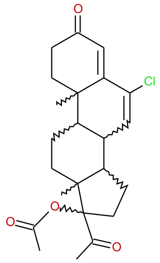	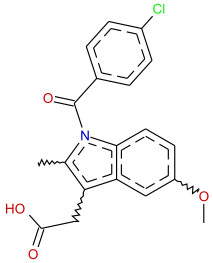
Actual Endpoint	Non-Carcinogen	Non-Carcinogen	Non-Carcinogen
Predicted Endpoint	Non-Carcinogen	Carcinogen	Non-Carcinogen
Distance	0.568	0.574	0.605
Reference	US FDA (Centre for Drug Eval.& Res./Off. Testing & Res.) Sept. 1997	US FDA (Centre for Drug Eval.& Res./Off. Testing & Res.) Sept. 1997	US FDA (Centre for Drug Eval.& Res./Off. Testing & Res.) Sept. 1997

Model Applicability

Unknown features are fingerprint features in the query molecule, but not found or appearing too infrequently in the training set.

1. All properties and OPS components are within expected ranges.

Feature Contribution

Top features for positive contribution				
**Fingerprint**	**Bit/Smiles**	**Feature Structure**	**Score**	**Carcinogen in training set**
FCFP_6	-387072142	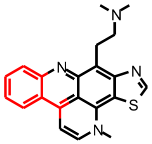 [*]:[c]1:[*]:[*]:[c]2:[cH]:[cH]:[cH]:[cH]:[c]:1:2	0.477	4 out of 8

FCFP_6	534778482	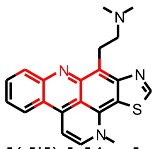 [*][c](:[*]):[c]1:n:[c](:[cH]:[*]):[c](:[*]):[*]:[c]:1:[*]	0.46	1 out of 1
FCFP_6	451371068	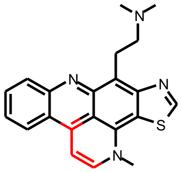 [*]\C=C\[c](:[*]):[*]	0.439	3 out of 6
**Top Features for negative contribution**				
**Fingerprint**	**Bit/Smiles**	**Feature Structure**	**Score**	**Carcinogen in training set**
FCFP_6	1990630846	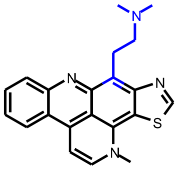 [*]:[c](:[*])CCN(C)C	-0.839	0 out of 5
FCFP_6	-124685461	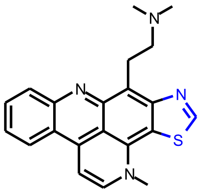 [*]1:[*]:s:[cH]:n:1	-0.731	1 out of 12
FCFP_6	-2001551693	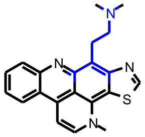 [*]N([*])CC[c](:[c](:[*]):[*]):[c](:[*]):[*]	-0.719	0 out of 4

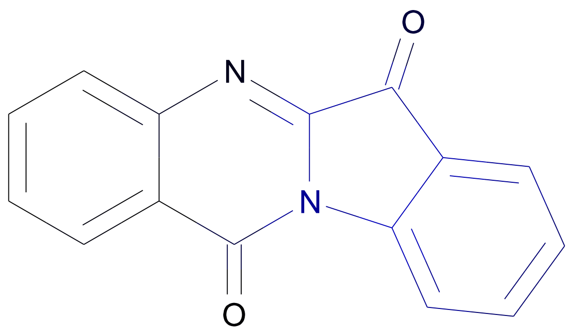

$C_{15}H_8N_2O_2$
Molecular Weight: 248.23621
ALogP: 2.331
Rotatable Bonds: 0
Acceptors: 3
Donors: 0

Model Prediction

Prediction: Non-Carcinogen
Probability: 0.221
Enrichment: 0.75
Bayesian Score: -3.76
Mahalanobis Distance: 10.6
Mahalanobis Distance p-value: 0.172

Prediction: Positive if the Bayesian score is above the estimated best cutoff value from minimizing the false positive and false negative rate.
Probability: The esimated probability that the sample is in the positive category. This assumes that the Bayesian score follows a normal distribution and is different from the prediction using a cutoff.
Enrichment: An estimate of enrichment, that is, the increased likelihood (versus random) of this sample being in the category.
Bayesian Score: The standard Laplacian-modified Bayesian score.
Mahalanobis Distance: The Mahalanobis distance (MD) is the distance to the center of the training data. The larger the MD, the less trustworthy the prediction.
Mahalanobis Distance p-value: The p-value gives the fraction of training data with an MD greater than or equal to the one for the given sample, assuming normally distributed data. The smaller the p-value, the less trustworthy the prediciton. For highly non-normal X properties (e.g., fingerprints), the MD p-value is wildly inaccurate.

Structural Similar Compounds

Name	Levamisole	Coumarin	Diazepam
Structure	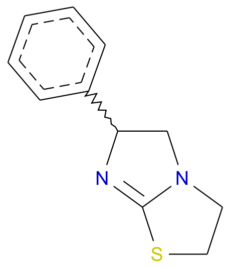	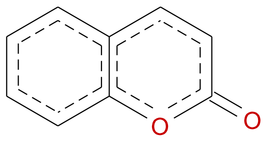	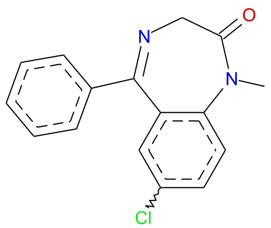
Actual Endpoint	Non-Carcinogen	Carcinogen	Carcinogen
Predicted Endpoint	Non-Carcinogen	Carcinogen	Non-Carcinogen
Distance	0.537	0.587	0.599
Reference	US FDA (Centre for Drug Eval.& Res./Off. Testing & Res.) Sept. 1997	US FDA (Centre for Drug Eval.& Res./Off. Testing & Res.) Sept. 1997	US FDA (Centre for Drug Eval.& Res./Off. Testing & Res.) Sept. 1997

Model Applicability

Unknown features are fingerprint features in the query molecule, but not found or appearing too infrequently in the training set.

1. All properties and OPS components are within expected ranges.

Feature Contribution

Top features for positive contribution

Fingerprint	Bit/Smiles	Feature Structure	Score	Carcinogen in training set
FCFP_6	1872154524	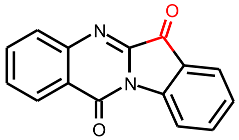 [*]C(=O)[*]	0.205	69 out of 213

FCFP_6	-1549163031	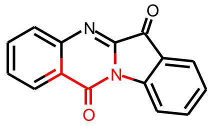 [*]N([*])C(=O)[c](:[*]):[*]	0.172	3 out of 9
FCFP_6	0	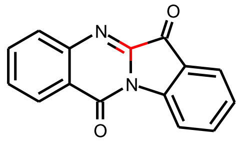 [*]C([*])[*]	0.114	90 out of 305
**Top Features for negative contribution**				
**Fingerprint**	**Bit/Smiles**	**Feature Structure**	**Score**	**Carcinogen in training set**
FCFP_6	564869443	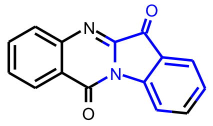 [*]N1C(=[*])C(=O)[c]2 :[cH]:[cH]:[*]:[cH]: [c]1:2	-0.719	0 out of 4
FCFP_6	159265197	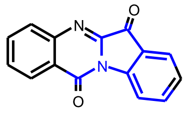 [*]C(=[*])N1C(=[*])C(=[*])[c]2:[cH]:[*]:[cH]:[cH]:[c]1:2	-0.666	1 out of 11
FCFP_6	-1549192822	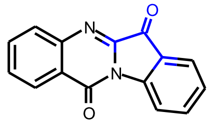 [*]C(=[*])C(=O)[c](:[*]):[*]	-0.489	3 out of 21

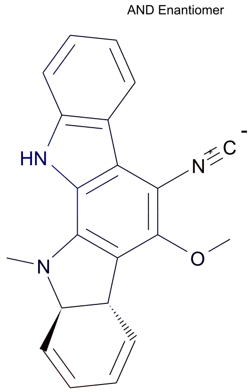

$C_{21}H_{17}N_3O$
Molecular Weight: 327.37918
ALogP: 4.078
Rotatable Bonds: 1
Acceptors: 2
Donors: 1

Model Prediction

Prediction: Non-Carcinogen
Probability: 0.235
Enrichment: 0.798
Bayesian Score: -3.03
Mahalanobis Distance: 13.7
Mahalanobis Distance p-value: 2.15e-005

Prediction: Positive if the Bayesian score is above the estimated best cutoff value from minimizing the false positive and false negative rate.
Probability: The esimated probability that the sample is in the positive category. This assumes that the Bayesian score follows a normal distribution and is different from the prediction using a cutoff.
Enrichment: An estimate of enrichment, that is, the increased likelihood (versus random) of this sample being in the category.
Bayesian Score: The standard Laplacian-modified Bayesian score.
Mahalanobis Distance: The Mahalanobis distance (MD) is the distance to the center of the training data. The larger the MD, the less trustworthy the prediction.
Mahalanobis Distance p-value: The p-value gives the fraction of training data with an MD greater than or equal to the one for the given sample, assuming normally distributed data. The smaller the p-value, the less trustworthy the prediciton. For highly non-normal X properties (e.g., fingerprints), the MD p-value is wildly inaccurate.

Structural Similar Compounds

Name	Levonorgestrel	Temazepam	Norethindrone
Structure	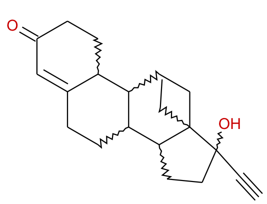	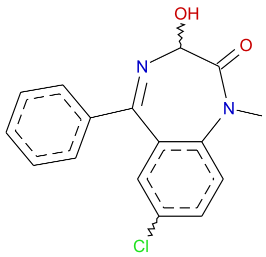	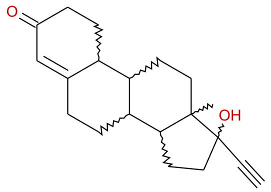
Actual Endpoint	Carcinogen	Non-Carcinogen	Carcinogen
Predicted Endpoint	Carcinogen	Non-Carcinogen	Carcinogen
Distance	0.588	0.590	0.592
Reference	US FDA (Centre for Drug Eval.& Res./Off. Testing & Res.) Sept. 1997	US FDA (Centre for Drug Eval.& Res./Off. Testing & Res.) Sept. 1997	US FDA (Centre for Drug Eval.& Res./Off. Testing & Res.) Sept. 1997

Model Applicability

Unknown features are fingerprint features in the query molecule, but not found or appearing too infrequently in the training set.

1. All properties and OPS components are within expected ranges.
2. Unknown FCFP_2 feature: 4: [*]#[C-]
3. Unknown FCFP_2 feature: -828984032: [*][c](:[*]):[c]([N+]#[*]):[c](:[*]):[*]
4. Unknown FCFP_2 feature: 1934974835: [*]:[c](:[*])[N+]#[C-]
5. Unknown FCFP_2 feature: -1487147388: [*][N+]#[C-]

Feature Contribution

Top features for positive contribution				
**Fingerprint**	**Bit/Smiles**	**Feature Structure**	**Score**	**Carcinogen in training set**
FCFP_6	451847724	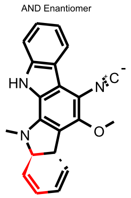 [*]CC=C([*])[*]	0.479	21 out of 48

FCFP_6	-387072142	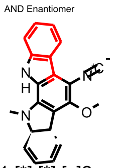 [*]:[c]1:[*]:[*]:[c]2:[cH]:[cH]:[cH]:[cH]:[c]:1:2	0.477	4 out of 8
FCFP_6	-1320007763	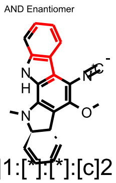 [*]:[c]1:[*]:[*]:[c]2:[*]:[cH]:[cH]:[cH]:[c]:1:2	0.348	6 out of 15

Top Features for negative contribution

**Fingerprint**	**Bit/Smiles**	**Feature Structure**	**Score**	**Carcinogen in training set**
FCFP_6	155061250	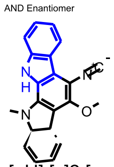 [*]:[c]1:[nH]:[c]2:[cH]:[cH]:[cH]:[cH]:[c]:2:[c]:1:[*]	-0.719	0 out of 4
FCFP_6	-1512836998	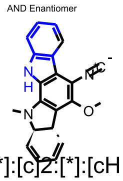 [*]1:[*]:[c]2:[*]:[cH]:[cH]:[cH]:[c]:2:[nH]:1	-0.521	1 out of 9
FCFP_6	-1043310069	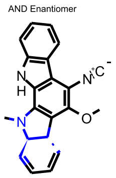 [*][C@H]1[*]:[*]N([*])[C@@H]1C=[*]	-0.406	10 out of 59

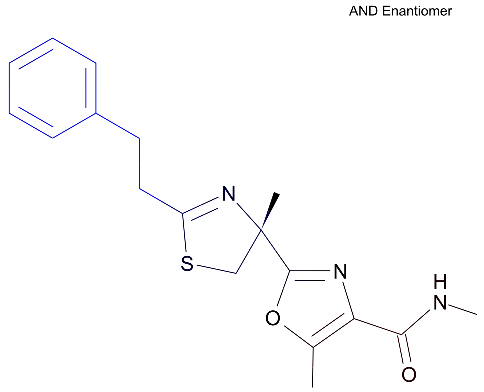

$C_{18}H_{21}N_3O_2S$
Molecular Weight: 343.44324
ALogP: 2.716
Rotatable Bonds: 5
Acceptors: 4
Donors: 1

Model Prediction

Prediction: Non-Carcinogen
Probability: 0.166
Enrichment: 0.563
Bayesian Score: -7.49
Mahalanobis Distance: 14.7
Mahalanobis Distance p-value: 3.05e-007

Prediction: Positive if the Bayesian score is above the estimated best cutoff value from minimizing the false positive and false negative rate.
Probability: The esimated probability that the sample is in the positive category. This assumes that the Bayesian score follows a normal distribution and is different from the prediction using a cutoff.
Enrichment: An estimate of enrichment, that is, the increased likelihood (versus random) of this sample being in the category.
Bayesian Score: The standard Laplacian-modified Bayesian score.
Mahalanobis Distance: The Mahalanobis distance (MD) is the distance to the center of the training data. The larger the MD, the less trustworthy the prediction.
Mahalanobis Distance p-value: The p-value gives the fraction of training data with an MD greater than or equal to the one for the given sample, assuming normally distributed data. The smaller the p-value, the less trustworthy the prediciton. For highly non-normal X properties (e.g., fingerprints), the MD p-value is wildly inaccurate.

Structural Similar Compounds

Name	Omeprazole	Lansoprazole	Oxaprocin
Structure	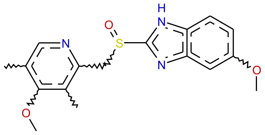	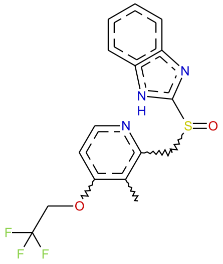	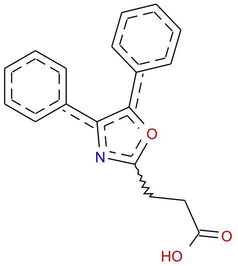
Actual Endpoint	Non-Carcinogen	Carcinogen	Carcinogen
Predicted Endpoint	Non-Carcinogen	Carcinogen	Carcinogen
Distance	0.533	0.539	0.575
Reference	US FDA (Centre for Drug Eval.& Res./Off. Testing & Res.) Sept. 1997	US FDA (Centre for Drug Eval.& Res./Off. Testing & Res.) Sept. 1997	US FDA (Centre for Drug Eval.& Res./Off. Testing & Res.) Sept. 1997

Model Applicability

Unknown features are fingerprint features in the query molecule, but not found or appearing too infrequently in the training set.

1. All properties and OPS components are within expected ranges.

Feature Contribution

Top features for positive contribution

Fingerprint	Bit/Smiles	Feature Structure	Score	Carcinogen in training set
FCFP_6	-1539132615	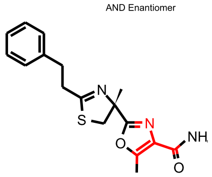 [*]:n:[c](C):[c](:[*]):[*]	0.328	19 out of 51

FCFP_6	1872154524	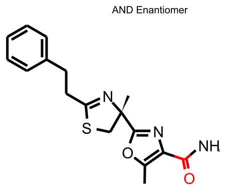<br>[*]C(=O)[*]	0.205	69 out of 213
FCFP_6	-1549103449	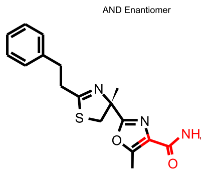<br>[*]NC(=O)[c](:[*]):[*]<br>]	0.204	7 out of 21

Top Features for negative contribution

Fingerprint	Bit/Smiles	Feature Structure	Score	Carcinogen in training set
FCFP_6	1981711554	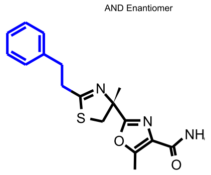<br>[*]CC[c]1:[cH]:[cH]:[<br>cH]:[cH]:[cH]:1	-1.42	0 out of 12
FCFP_6	1388176727	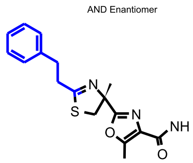<br>[*]C(=[*])CC[c]1:[cH]<br>:[cH]:[cH]:[cH]:[cH]<br>:1	-1.21	0 out of 9
FCFP_6	-497728148	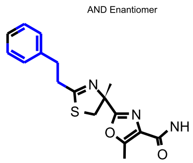<br>[*]C([*])C[c]1:[cH]:[<br>cH]:[*]:[c]([*]):[cH<br>]:1	-0.96	2 out of 26

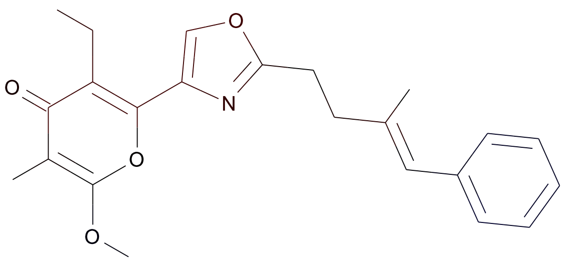

$C_{23}H_{25}NO_4$
Molecular Weight: 379.4489
ALogP: 5.22
Rotatable Bonds: 7
Acceptors: 4
Donors: 0

Model Prediction

Prediction: Carcinogen
Probability: 0.381
Enrichment: 1.29
Bayesian Score: 2.45
Mahalanobis Distance: 12.4
Mahalanobis Distance p-value: 0.00215

Prediction: Positive if the Bayesian score is above the estimated best cutoff value from minimizing the false positive and false negative rate.
Probability: The esimated probability that the sample is in the positive category. This assumes that the Bayesian score follows a normal distribution and is different from the prediction using a cutoff.
Enrichment: An estimate of enrichment, that is, the increased likelihood (versus random) of this sample being in the category.
Bayesian Score: The standard Laplacian-modified Bayesian score.
Mahalanobis Distance: The Mahalanobis distance (MD) is the distance to the center of the training data. The larger the MD, the less trustworthy the prediction.
Mahalanobis Distance p-value: The p-value gives the fraction of training data with an MD greater than or equal to the one for the given sample, assuming normally distributed data. The smaller the p-value, the less trustworthy the prediciton. For highly non-normal X properties (e.g., fingerprints), the MD p-value is wildly inaccurate.

Structural Similar Compounds

Name	Permethrin	Levomethadyl	Flurazepam
Structure	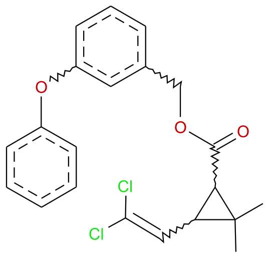	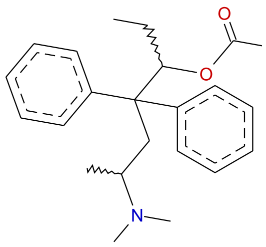	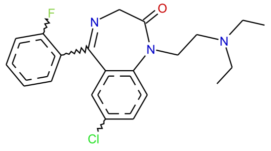
Actual Endpoint	Carcinogen	Non-Carcinogen	Non-Carcinogen
Predicted Endpoint	Carcinogen	Non-Carcinogen	Non-Carcinogen
Distance	0.559	0.611	0.614
Reference	US FDA (Centre for Drug Eval.& Res./Off. Testing & Res.) Sept. 1997	US FDA (Centre for Drug Eval.& Res./Off. Testing & Res.) Sept. 1997	US FDA (Centre for Drug Eval.& Res./Off. Testing & Res.) Sept. 1997

Model Applicability

Unknown features are fingerprint features in the query molecule, but not found or appearing too infrequently in the training set.

1. All properties and OPS components are within expected ranges.
2. Unknown FCFP_2 feature: -1678245750: [*]OC(=C([*])[*])[c](:[*]):[*]
3. Unknown FCFP_2 feature: -2115241127: [*]OC(=C([*])[*])O[*]

Feature Contribution

Top features for positive contribution

Fingerprint	Bit/Smiles	Feature Structure	Score	Carcinogen in training set
FCFP_6	-1993961799	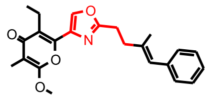 [*]CC[c]1:o:[cH]:[c]([*]):n:1	0.46	1 out of 1

FCFP_6	-2084412427	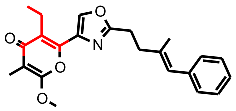 [*]C(=C(CC)C(=[*])[*])[*]	0.46	1 out of 1
FCFP_6	451371068	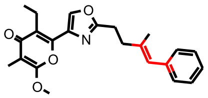 [*]\C=C\[c](:[*]):[*]	0.439	3 out of 6
**Top Features for negative contribution**				
**Fingerprint**	**Bit/Smiles**	**Feature Structure**	**Score**	**Carcinogen in training set**
FCFP_6	907007053	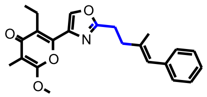 [*]C([*])C[c](:[*]):[*]	-0.366	11 out of 62
FCFP_6	136627117	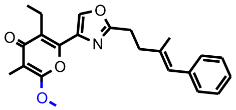 [*]OC	-0.252	10 out of 50
FCFP_6	-631817888	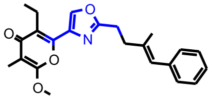 [*][c]1:n:[c](:o:[c]: 1C)C([*])([*])[*]	-0.233	0 out of 1

Remdesivir

TOPKAT_Mouse_Male_FDA_None_vs_Carcinogen

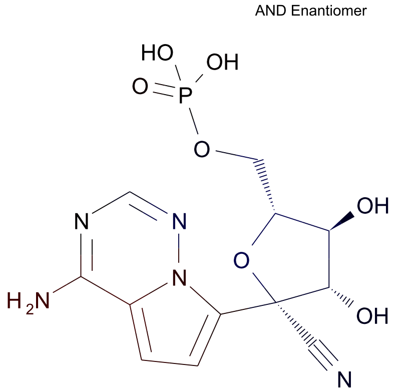

$C_{12}H_{14}N_5O_7P$
Molecular Weight: 371.24262
ALogP: -1.502
Rotatable Bonds: 4
Acceptors: 11
Donors: 5

Model Prediction

Prediction: Non-Carcinogen
Probability: 0.239
Enrichment: 0.812
Bayesian Score: -2.82
Mahalanobis Distance: 19.2
Mahalanobis Distance p-value: 7.81e-017

Prediction: Positive if the Bayesian score is above the estimated best cutoff value from minimizing the false positive and false negative rate.
Probability: The esimated probability that the sample is in the positive category. This assumes that the Bayesian score follows a normal distribution and is different from the prediction using a cutoff.
Enrichment: An estimate of enrichment, that is, the increased likelihood (versus random) of this sample being in the category.
Bayesian Score: The standard Laplacian-modified Bayesian score.
Mahalanobis Distance: The Mahalanobis distance (MD) is the distance to the center of the training data. The larger the MD, the less trustworthy the prediction.
Mahalanobis Distance p-value: The p-value gives the fraction of training data with an MD greater than or equal to the one for the given sample, assuming normally distributed data. The smaller the p-value, the less trustworthy the prediciton. For highly non-normal X properties (e.g., fingerprints), the MD p-value is wildly inaccurate.

Structural Similar Compounds

Name	Famotidine	Tetracycline	Ribavirin
Structure	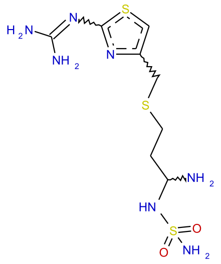	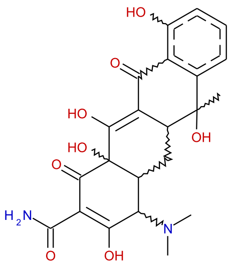	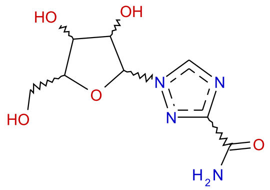
Actual Endpoint	Non-Carcinogen	Non-Carcinogen	Non-Carcinogen
Predicted Endpoint	Non-Carcinogen	Non-Carcinogen	Non-Carcinogen
Distance	0.813	0.843	0.860
Reference	US FDA (Centre for Drug Eval.& Res./Off. Testing & Res.) Sept. 1997	US FDA (Centre for Drug Eval.& Res./Off. Testing & Res.) Sept. 1997	US FDA (Centre for Drug Eval.& Res./Off. Testing & Res.) Sept. 1997

Model Applicability

Unknown features are fingerprint features in the query molecule, but not found or appearing too infrequently in the training set.

1. All properties and OPS components are within expected ranges.
2. Unknown FCFP_2 feature: 472180098: [*]OP(=O)(O)O
3. Unknown FCFP_2 feature: -332197802: [*][c]1:[*]:[*]:[c](:[*]):n:1:n:[*]

Feature Contribution

Top features for positive contribution

Fingerprint	Bit/Smiles	Feature Structure	Score	Carcinogen in training set
FCFP_6	-450797925	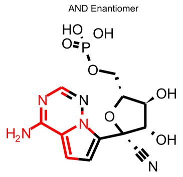 N[c]1:n:[cH]:[*]:n2:[*]:[*]:[cH]:[c]:1:2	0.676	2 out of 2

FCFP_6	-1151884458	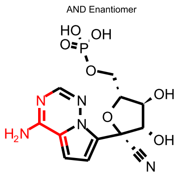 [*]:n:[c](N):[c](:[*]):[*]	0.348	6 out of 15
FCFP_6	-1280036918	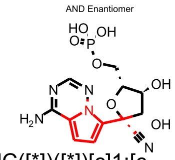 [*]C([*])([*])[c]1:[c H]:[cH]:[c](:[*]):n: 1:[*]	0.333	7 out of 18
**Top Features for negative contribution**				
**Fingerprint**	**Bit/Smiles**	**Feature Structure**	**Score**	**Carcinogen in training set**
FCFP_6	-124685461	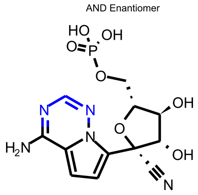 [*]1:[*]:s:[cH]:n:1	-0.731	1 out of 12
FCFP_6	422052003	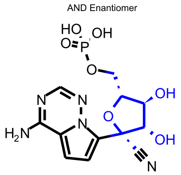 [*]C[C@H]1OC([*])([*])[C@@H](O)[C@@H]1O	-0.582	0 out of 3
FCFP_6	-1277879912	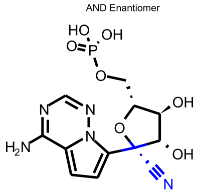 [*]C([*])([*])C#N	-0.582	0 out of 3

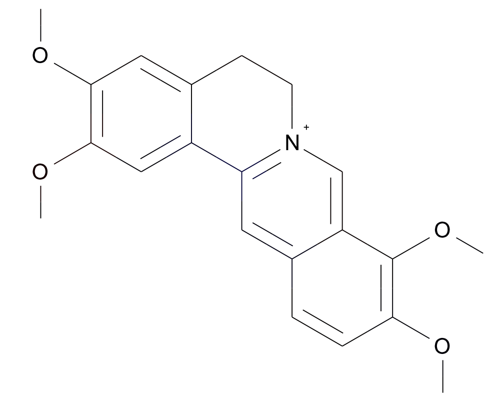

$C_{21}H_{22}NO_4$
Molecular Weight: 352.40368
ALogP: 4.161
Rotatable Bonds: 4
Acceptors: 4
Donors: 0

Structural Similar Compounds

Name	Ethynodiol	Loratidine	Phenylbutazone
Structure	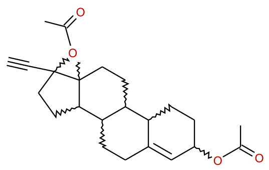	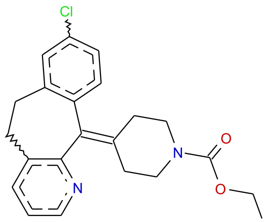	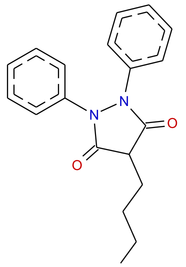
Actual Endpoint	Multiple-Carcinogen	Single-Carcinogen	Single-Carcinogen
Predicted Endpoint	Multiple-Carcinogen	Single-Carcinogen	Single-Carcinogen
Distance	0.587	0.671	0.704
Reference	US FDA (Centre for Drug Eval.& Res./Off. Testing & Res.) Sept. 1997	US FDA (Centre for Drug Eval.& Res./Off. Testing & Res.) Sept. 1997	US FDA (Centre for Drug Eval.& Res./Off. Testing & Res.) Sept. 1997

Model Prediction

Prediction: Multiple-Carcinogen
Probability: 0.223
Enrichment: 0.742
Bayesian Score: -1.41
Mahalanobis Distance: 13.5
Mahalanobis Distance p-value: 4.08e-005

Prediction: Positive if the Bayesian score is above the estimated best cutoff value from minimizing the false positive and false negative rate.
Probability: The esimated probability that the sample is in the positive category. This assumes that the Bayesian score follows a normal distribution and is different from the prediction using a cutoff.
Enrichment: An estimate of enrichment, that is, the increased likelihood (versus random) of this sample being in the category.
Bayesian Score: The standard Laplacian-modified Bayesian score.
Mahalanobis Distance: The Mahalanobis distance (MD) is the distance to the center of the training data. The larger the MD, the less trustworthy the prediction.
Mahalanobis Distance p-value: The p-value gives the fraction of training data with an MD greater than or equal to the one for the given sample, assuming normally distributed data. The smaller the p-value, the less trustworthy the prediciton. For highly non-normal X properties (e.g., fingerprints), the MD p-value is wildly inaccurate.

Model Applicability

Unknown features are fingerprint features in the query molecule, but not found or appearing too infrequently in the training set.

1. OPS PC9 out of range. Value: 3.2061. Training min, max, SD, explained variance: -3.148, 3.1804, 1.416, 0.0374.
2. Unknown FCFP_2 feature: 24: [*][n+](:[*]):[*]
3. Unknown FCFP_2 feature: 414371600: [*]C[n+](:[c]([*]):[*]):c:[*]
4. Unknown FCFP_2 feature: -150573739: [*]CC[n+](:[*]):[*]
5. Unknown FCFP_2 feature: -1861407456: [*][n+](:[*]):[c]([c](:[*]):[*]):c:[*]
6. Unknown FCFP_2 feature: 1618392993: [*][n+](:[*]):c:[c](:[*]):[*]

Feature Contribution

Top features for positive contribution

Fingerprint	Bit/Smiles	Feature Structure	Score	Multiple-Carcinogen in training set

FCFP_12	1028934530	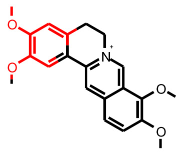<br>[*]O[c]1:[cH]:[*]:[c]([*]):[cH]:[c]:1OC	0.4	1 out of 1
FCFP_12	907007053	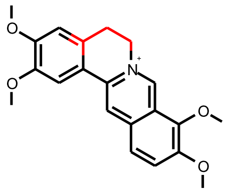<br>[*]CC[c](:[*]):[*]	0.235	5 out of 11
FCFP_12	-105186863	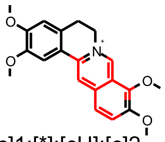<br>[*][c]1:[*]:[cH]:[c]2:[c]([*]):[*]:[cH]:[cH]:[c]:2:[cH]:1	0.174	1 out of 2
**Top Features for negative contribution**				
**Fingerprint**	**Bit/Smiles**	**Feature Structure**	**Score**	**Multiple-Carcinogen in training set**
FCFP_12	-1861645784	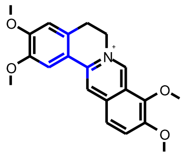<br>[*][c](:[*]):[c](:[cH]:[*])[c](:[*]):[*]	-0.519	0 out of 2
FCFP_12	1618154665	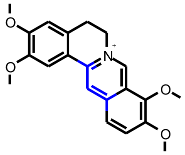<br>[*][c](:[*]):[cH]:[c](:[*]):[*]	-0.409	13 out of 59

FCFP_12	1036089772	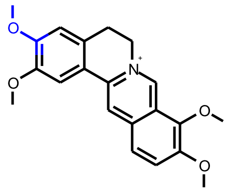 [*]:[c](:[*])OC	-0.365	3 out of 14

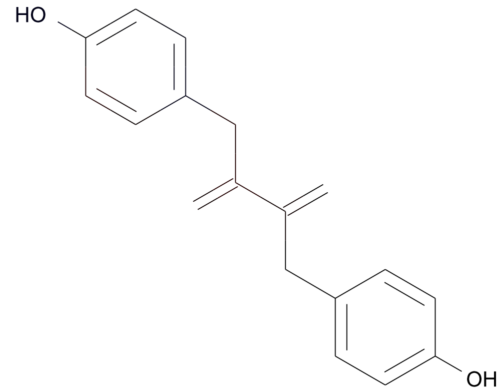

$C_{18}H_{18}O_2$
Molecular Weight: 266.33432
ALogP: 4.8
Rotatable Bonds: 5
Acceptors: 2
Donors: 2

Structural Similar Compounds

Name	Diethylstilbesterol	Pronetalol	Prilocaine
Structure	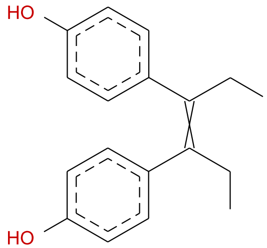	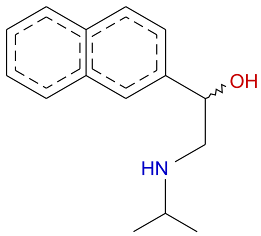	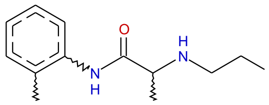
Actual Endpoint	Single-Carcinogen	Single-Carcinogen	Single-Carcinogen
Predicted Endpoint	Single-Carcinogen	Single-Carcinogen	Single-Carcinogen
Distance	0.442	0.680	0.695
Reference	US FDA (Centre for Drug Eval.& Res./Off. Testing & Res.) Sept. 1997	US FDA (Centre for Drug Eval.& Res./Off. Testing & Res.) Sept. 1997	US FDA (Centre for Drug Eval.& Res./Off. Testing & Res.) Sept. 1997

Model Prediction

Prediction: Multiple-Carcinogen
Probability: 0.236
Enrichment: 0.784
Bayesian Score: -0.885
Mahalanobis Distance: 14.5
Mahalanobis Distance p-value: 6.27e-006

Prediction: Positive if the Bayesian score is above the estimated best cutoff value from minimizing the false positive and false negative rate.
Probability: The esimated probability that the sample is in the positive category. This assumes that the Bayesian score follows a normal distribution and is different from the prediction using a cutoff.
Enrichment: An estimate of enrichment, that is, the increased likelihood (versus random) of this sample being in the category.
Bayesian Score: The standard Laplacian-modified Bayesian score.
Mahalanobis Distance: The Mahalanobis distance (MD) is the distance to the center of the training data. The larger the MD, the less trustworthy the prediction.
Mahalanobis Distance p-value: The p-value gives the fraction of training data with an MD greater than or equal to the one for the given sample, assuming normally distributed data. The smaller the p-value, the less trustworthy the prediciton. For highly non-normal X properties (e.g., fingerprints), the MD p-value is wildly inaccurate.

Model Applicability

Unknown features are fingerprint features in the query molecule, but not found or appearing too infrequently in the training set.

1. All properties and OPS components are within expected ranges.
2. Unknown FCFP_2 feature: 129344189: [*]C(=C)[*]

Feature Contribution

Top features for positive contribution

Fingerprint	Bit/Smiles	Feature Structure	Score	Multiple-Carcinogen in training set
FCFP_12	-1847351220	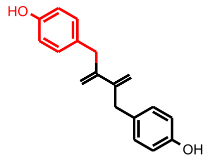 [*]C[c]1:[cH]:[cH]:[c](O):[cH]:[cH]:1	0.395	2 out of 3

FCFP_12	436886043	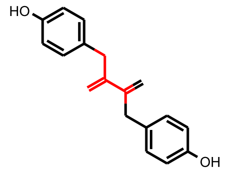 [*]CC(=C)C(=[*])[*]	0.27	7 out of 15
FCFP_12	907007053	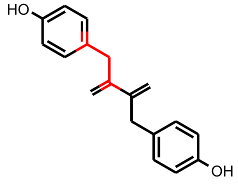 [*]CC[c](:[*]):[*]	0.235	5 out of 11

Top Features for negative contribution

**Fingerprint**	**Bit/Smiles**	**Feature Structure**	**Score**	**Multiple-Carcinogen in training set**
FCFP_12	7	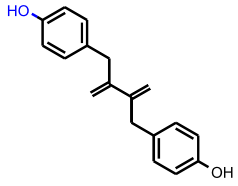 [*]O	-0.71	2 out of 15
FCFP_12	1618154665	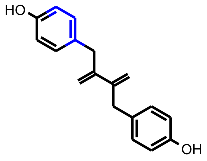 [*][c](:[*]):[cH]:[c](:[*]):[*]	-0.409	13 out of 59
FCFP_12	16	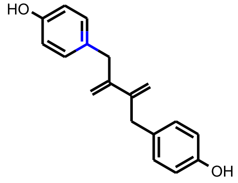 [*][c](:[*]):[*]	-0.308	16 out of 65

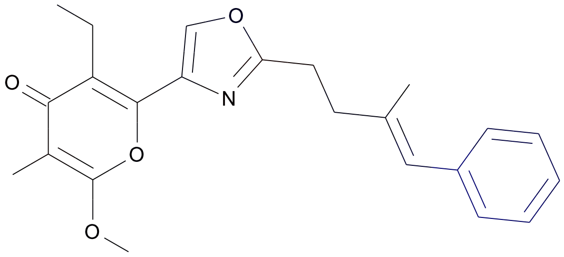

$C_{23}H_{25}NO_4$
Molecular Weight: 379.4489
ALogP: 5.22
Rotatable Bonds: 7
Acceptors: 4
Donors: 0

Structural Similar Compounds

Name	Permethrin	Etretinate	Ethynodiol
Structure	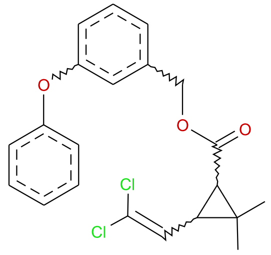	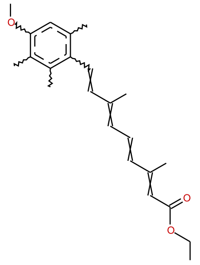	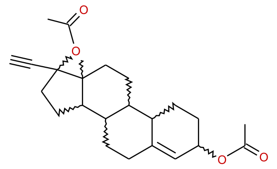
Actual Endpoint	Single-Carcinogen	Single-Carcinogen	Multiple-Carcinogen
Predicted Endpoint	Single-Carcinogen	Single-Carcinogen	Multiple-Carcinogen
Distance	0.591	0.642	0.680
Reference	US FDA (Centre for Drug Eval.& Res./Off. Testing & Res.) Sept. 1997	US FDA (Centre for Drug Eval.& Res./Off. Testing & Res.) Sept. 1997	US FDA (Centre for Drug Eval.& Res./Off. Testing & Res.) Sept. 1997

Model Prediction

Prediction: Multiple-Carcinogen
Probability: 0.203
Enrichment: 0.675
Bayesian Score: -2.39
Mahalanobis Distance: 12.1
Mahalanobis Distance p-value: 0.000545

Prediction: Positive if the Bayesian score is above the estimated best cutoff value from minimizing the false positive and false negative rate.
Probability: The esimated probability that the sample is in the positive category. This assumes that the Bayesian score follows a normal distribution and is different from the prediction using a cutoff.
Enrichment: An estimate of enrichment, that is, the increased likelihood (versus random) of this sample being in the category.
Bayesian Score: The standard Laplacian-modified Bayesian score.
Mahalanobis Distance: The Mahalanobis distance (MD) is the distance to the center of the training data. The larger the MD, the less trustworthy the prediction.
Mahalanobis Distance p-value: The p-value gives the fraction of training data with an MD greater than or equal to the one for the given sample, assuming normally distributed data. The smaller the p-value, the less trustworthy the prediciton. For highly non-normal X properties (e.g., fingerprints), the MD p-value is wildly inaccurate.

Model Applicability

Unknown features are fingerprint features in the query molecule, but not found or appearing too infrequently in the training set.

1. All properties and OPS components are within expected ranges.
2. Unknown FCFP_2 feature: -1678245750: [*]OC(=C([*])[*])[c](:[*]):[*]
3. Unknown FCFP_2 feature: -2115241127: [*]OC(=C([*])[*])O[*]

Feature Contribution

Top features for positive contribution

Fingerprint	Bit/Smiles	Feature Structure	Score	Multiple-Carcinogen in training set
FCFP_12	327438754	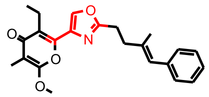 [*]C(=[*])[c]1:[cH]:o :[c]([*]):n:1	0.527	3 out of 4

FCFP_12	-634178238	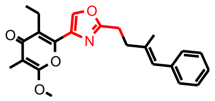 [*]C[c]1:o:[cH]:[c]([*]):n:1	0.4	1 out of 1
FCFP_12	436886043	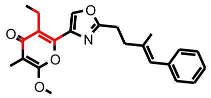 [*]CC(=C)C(=[*])[*]	0.27	7 out of 15

Top Features for negative contribution

Fingerprint	Bit/Smiles	Feature Structure	Score	Multiple-Carcinogen in training set
FCFP_12	991735244	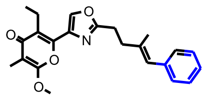 [*][c]1:[*]:[cH]:[cH] :[cH]:[cH]:1	-0.562	5 out of 28
FCFP_12	17	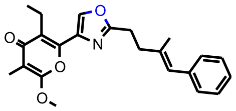 [*]:o:[*]	-0.53	5 out of 27
FCFP_12	1747237384	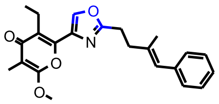 [*][c]1:[*]:[*]:[cH]: o:1	-0.424	5 out of 24

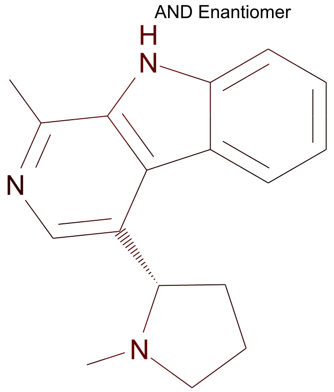

$C_{17}H_{19}N_3$
Molecular Weight: 265.35286
ALogP: 3.018
Rotatable Bonds: 1
Acceptors: 2
Donors: 1

Structural Similar Compounds

Name	3-AMINOPYRENE	CARBAMIC ACID; METHYL-; 1-(5;6;7;8-TETRAHYDRO)NAPHTHYL ESTER	p-Anisidine; N-cyclohexyl-
Structure	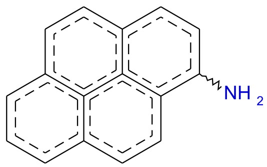	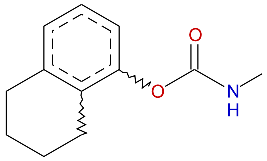	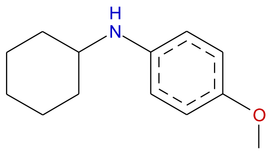
Actual Endpoint	Moderate_Severe	Mild	Moderate_Severe
Predicted Endpoint	Moderate_Severe	Mild	Mild
Distance	0.603	0.607	0.610
Reference	AIHAAP 30;470;69	28ZPAK-;163;72	Prehled Prumyslove Toxikologie; Organicke Latky; Marhold; J. -;723;86

Model Prediction

Prediction: Moderate_Severe
Probability: 0.916
Enrichment: 1.33
Bayesian Score: 4.18
Mahalanobis Distance: 8.07
Mahalanobis Distance p-value: 0.909

Prediction: Positive if the Bayesian score is above the estimated best cutoff value from minimizing the false positive and false negative rate.
Probability: The esimated probability that the sample is in the positive category. This assumes that the Bayesian score follows a normal distribution and is different from the prediction using a cutoff.
Enrichment: An estimate of enrichment, that is, the increased likelihood (versus random) of this sample being in the category.
Bayesian Score: The standard Laplacian-modified Bayesian score.
Mahalanobis Distance: The Mahalanobis distance (MD) is the distance to the center of the training data. The larger the MD, the less trustworthy the prediction.
Mahalanobis Distance p-value: The p-value gives the fraction of training data with an MD greater than or equal to the one for the given sample, assuming normally distributed data. The smaller the p-value, the less trustworthy the prediciton. For highly non-normal X properties (e.g., fingerprints), the MD p-value is wildly inaccurate.

Model Applicability

Unknown features are fingerprint features in the query molecule, but not found or appearing too infrequently in the training set.

1. All properties and OPS components are within expected ranges.

Feature Contribution

Top features for positive contribution				
**Fingerprint**	**Bit/Smiles**	**Feature Structure**	**Score**	**Moderate_Severe in training set**
FCFP_10	-587569116	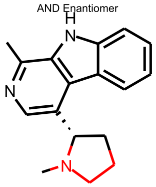 [*]N1[*][*]CC1	0.335	66 out of 71

FCFP_10	136388789	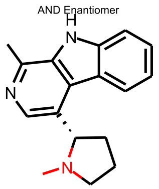 [*]N([*])C	0.331	28 out of 30
FCFP_10	-1343180157	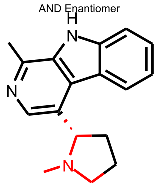 [*][C@@H]1[*][*]CN1C	0.317	63 out of 69
**Top Features for negative contribution**				
**Fingerprint**	**Bit/Smiles**	**Feature Structure**	**Score**	**Moderate_Severe in training set**
FCFP_10	-1320007763	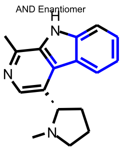 [*]:[c]1:[*]:[*]:[c]2:[*]:[cH]:[cH]:[cH]:[c]:1:2	-0.316	19 out of 40
FCFP_10	307419094	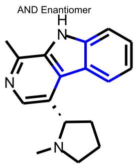 [*]:[cH]:[c]1:[c](:[*]):[*]:[*]:[c]:1:[*]	-0.29	21 out of 43
FCFP_10	991735244	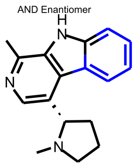 [*]:[c]1:[*]:[cH]:[cH]:[cH]:[cH]:1	-0.185	130 out of 237

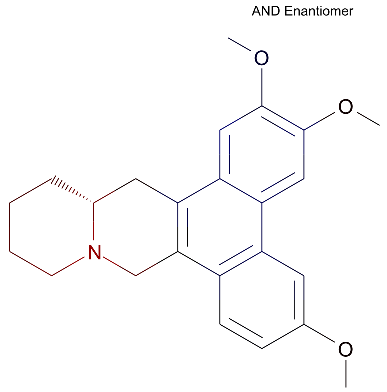

$C_{24}H_{27}NO_3$
Molecular Weight: 377.47608
ALogP: 4.691
Rotatable Bonds: 3
Acceptors: 4
Donors: 0

Structural Similar Compounds

Name	Carbamic acid; N-methyl-N-(1-(3;5-xylyloxy)-2-propyl)-;	PHOSPHOROUS ACID; TRIPHENYL ESTER	PROPANE;2;2-BIS(P-2;3-EPOXYPROPOXY)PHENYL)-
Structure	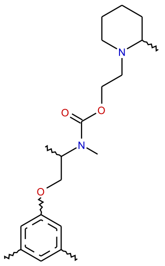	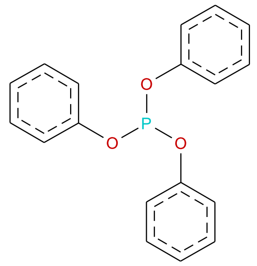	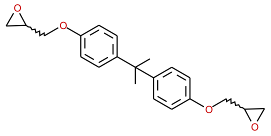
Actual Endpoint	Moderate_Severe	Mild	Moderate_Severe
Predicted Endpoint	Moderate_Severe	Mild	Moderate_Severe
Distance	0.575	0.646	0.659
Reference	Arzneimittel-Forschung 9;113;59	28ZPAK-;205;72	28ZPAK-;137;72

Model Prediction

Prediction: Moderate_Severe
Probability: 0.818
Enrichment: 1.19
Bayesian Score: -0.221
Mahalanobis Distance: 10.9
Mahalanobis Distance p-value: 0.00604

Prediction: Positive if the Bayesian score is above the estimated best cutoff value from minimizing the false positive and false negative rate.
Probability: The esimated probability that the sample is in the positive category. This assumes that the Bayesian score follows a normal distribution and is different from the prediction using a cutoff.
Enrichment: An estimate of enrichment, that is, the increased likelihood (versus random) of this sample being in the category.
Bayesian Score: The standard Laplacian-modified Bayesian score.
Mahalanobis Distance: The Mahalanobis distance (MD) is the distance to the center of the training data. The larger the MD, the less trustworthy the prediction.
Mahalanobis Distance p-value: The p-value gives the fraction of training data with an MD greater than or equal to the one for the given sample, assuming normally distributed data. The smaller the p-value, the less trustworthy the prediciton. For highly non-normal X properties (e.g., fingerprints), the MD p-value is wildly inaccurate.

Model Applicability

Unknown features are fingerprint features in the query molecule, but not found or appearing too infrequently in the training set.

1. All properties and OPS components are within expected ranges.

Feature Contribution

Top features for positive contribution

Fingerprint	Bit/Smiles	Feature Structure	Score	Moderate_Severe in training set
FCFP_10	906798516	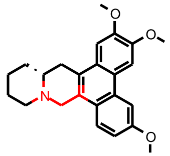 AND Enantiomer [*]N([*])C[c](:[*]):[*]	0.344	6 out of 6

FCFP_10	-587569116	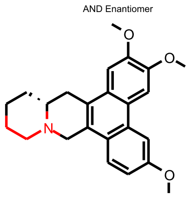 [*]N1[*][*]CC1	0.335	66 out of 71
FCFP_10	-1866659497	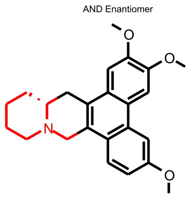 [*]CN1CCCC[C@@H]1[*]	0.332	5 out of 5

Top Features for negative contribution

Fingerprint	Bit/Smiles	Feature Structure	Score	Moderate_Severe in training set
FCFP_10	1878037426	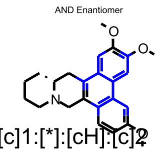 [*][c]1:[*]:[cH]:[c]2:[c]([*]):[*]:[c](:[*]):[c](:[cH]:[*]):[c]:2:[cH]:1	-1.09	0 out of 3
FCFP_10	-1977641857	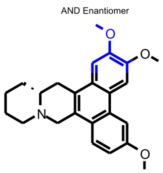 [*][c](:[*]):[c](OC):[cH]:[*]	-0.78	4 out of 15
FCFP_10	-1320007763	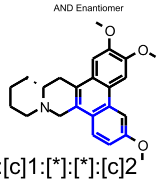 [*]:[c]1:[*]:[*]:[c]2:[*]:[cH]:[cH]:[cH]:[c]:1:2	-0.316	19 out of 40

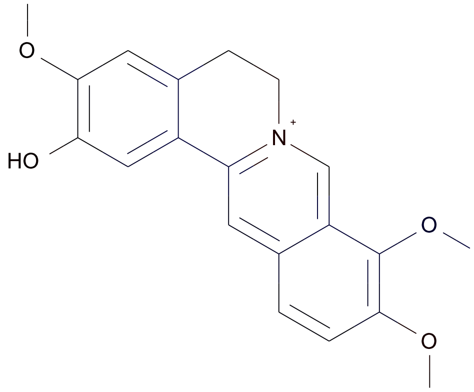

$C_{20}H_{20}NO_4$
Molecular Weight: 338.3771
ALogP: 3.936
Rotatable Bonds: 3
Acceptors: 4
Donors: 1

Model Prediction

Prediction: Mild
Probability: 0.782
Enrichment: 1.14
Bayesian Score: -1.62
Mahalanobis Distance: 7.3
Mahalanobis Distance p-value: 0.993

Prediction: Positive if the Bayesian score is above the estimated best cutoff value from minimizing the false positive and false negative rate.
Probability: The esimated probability that the sample is in the positive category. This assumes that the Bayesian score follows a normal distribution and is different from the prediction using a cutoff.
Enrichment: An estimate of enrichment, that is, the increased likelihood (versus random) of this sample being in the category.
Bayesian Score: The standard Laplacian-modified Bayesian score.
Mahalanobis Distance: The Mahalanobis distance (MD) is the distance to the center of the training data. The larger the MD, the less trustworthy the prediction.
Mahalanobis Distance p-value: The p-value gives the fraction of training data with an MD greater than or equal to the one for the given sample, assuming normally distributed data. The smaller the p-value, the less trustworthy the prediciton. For highly non-normal X properties (e.g., fingerprints), the MD p-value is wildly inaccurate.

Structural Similar Compounds

Name	1-BENZOYLAMINO-4-METHOXY-5-CHLORANTHRAQUINONE	BENZAMIDE; N-(5-CHLORO-1-ANTHRAQUINONYL)-	BENZILIC ACID; 4;4'-DICHLORO-; ISOPROPYL ESTER
Structure	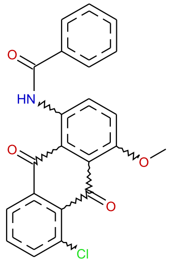	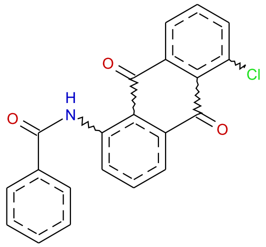	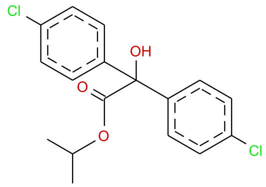
Actual Endpoint	Mild	Mild	Moderate_Severe
Predicted Endpoint	Mild	Mild	Moderate_Severe
Distance	0.585	0.608	0.611
Reference	28ZPAK-;90;72	28ZPAK 89;72	CIGET* -;-;77

Model Applicability

Unknown features are fingerprint features in the query molecule, but not found or appearing too infrequently in the training set.

1. All properties and OPS components are within expected ranges.
2. Unknown FCFP_2 feature: -150573739: [*]CC[n+](:[*]):[*]
3. Unknown FCFP_2 feature: -1861407456: [*][n+](:[*]):[c]([c](:[*]):[*]):c:[*]

Feature Contribution

Top features for positive contribution

Fingerprint	Bit/Smiles	Feature Structure	Score	Moderate_Severe in training set
FCFP_10	1028934530	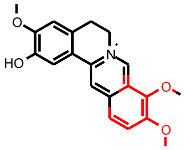 [*]O[c]1:[cH]:[*]:[c](:[*]):[cH]:[c]:1OC	0.256	2 out of 2

FCFP_10	7	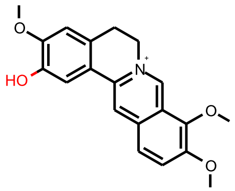 [*]O	0.219	117 out of 142
FCFP_10	523826990	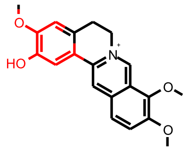 [*]O[c]1:[cH]:[*]:[c]([*]):[cH]:[c]:1O	0.186	1 out of 1

Top Features for negative contribution

Fingerprint	Bit/Smiles	Feature Structure	Score	Moderate_Severe in training set
FCFP_10	-1977641857	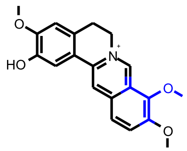 [*][c](:[*]):[c](OC):[cH]:[*]	-0.78	4 out of 15
FCFP_10	-1861645784	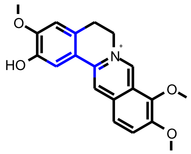 [*][c](:[*]):[c](:[cH]:[*])[c](:[*]):[*]	-0.598	9 out of 26
FCFP_10	-620676039	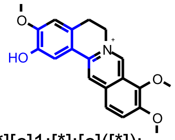 [*][c]1:[*]:[c]([*]):[c](:[cH]:[c]:1O)[c](:[*]):[*]	-0.507	0 out of 1

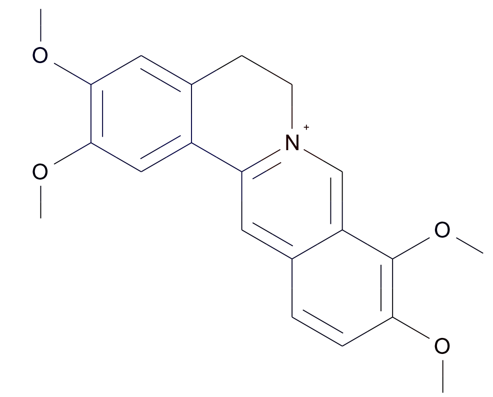

$C_{21}H_{22}NO_4$
Molecular Weight: 352.40368
ALogP: 4.161
Rotatable Bonds: 4
Acceptors: 4
Donors: 0

Model Prediction

Prediction: Mild
Probability: 0.741
Enrichment: 1.08
Bayesian Score: -2.71
Mahalanobis Distance: 6.35
Mahalanobis Distance p-value: 1

Prediction: Positive if the Bayesian score is above the estimated best cutoff value from minimizing the false positive and false negative rate.
Probability: The esimated probability that the sample is in the positive category. This assumes that the Bayesian score follows a normal distribution and is different from the prediction using a cutoff.
Enrichment: An estimate of enrichment, that is, the increased likelihood (versus random) of this sample being in the category.
Bayesian Score: The standard Laplacian-modified Bayesian score.
Mahalanobis Distance: The Mahalanobis distance (MD) is the distance to the center of the training data. The larger the MD, the less trustworthy the prediction.
Mahalanobis Distance p-value: The p-value gives the fraction of training data with an MD greater than or equal to the one for the given sample, assuming normally distributed data. The smaller the p-value, the less trustworthy the prediciton. For highly non-normal X properties (e.g., fingerprints), the MD p-value is wildly inaccurate.

Structural Similar Compounds

Name	PHOSPHOROUS ACID; TRIPHENYL ESTER	PROPANE;2;2-BIS(P-2;3-EPOXYPROPOXY)PHENYL)-	Carbamic acid; N-methyl-N-(1-(3;5-xylyloxy)-2-propyl)-;
Structure	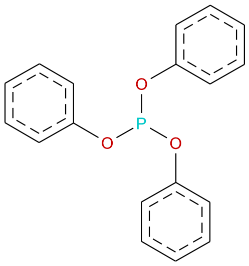	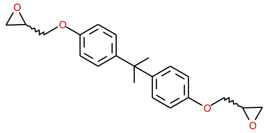	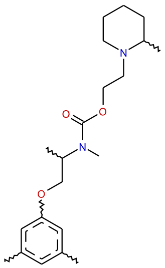
Actual Endpoint	Mild	Moderate_Severe	Moderate_Severe
Predicted Endpoint	Mild	Moderate_Severe	Moderate_Severe
Distance	0.577	0.593	0.596
Reference	28ZPAK-;205;72	28ZPAK-;137;72	Arzneimittel-Forschung 9;113;59

Model Applicability

Unknown features are fingerprint features in the query molecule, but not found or appearing too infrequently in the training set.

1. All properties and OPS components are within expected ranges.
2. Unknown FCFP_2 feature: -150573739: [*]CC[n+](:[*]):[*]
3. Unknown FCFP_2 feature: -1861407456: [*][n+](:[*]):[c]([c](:[*]):[*]):c:[*]

Feature Contribution

Top features for positive contribution

Fingerprint	Bit/Smiles	Feature Structure	Score	Moderate_Severe in training set
FCFP_10	1028934530	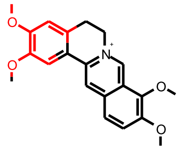 [*]O[c]1:[cH]:[*]:[c](:[*]):[cH]:[c]:1OC	0.256	2 out of 2

FCFP_10	24	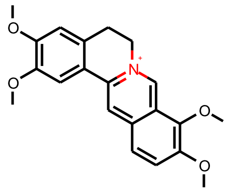 [*][n+](:[*]):[*]	0.186	1 out of 1
FCFP_10	414371600	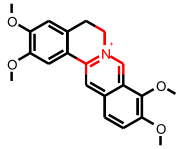 [*]C[n+](:[cH]:[*]):[c]([*]):[*]	0.186	1 out of 1

Top Features for negative contribution

Fingerprint	Bit/Smiles	Feature Structure	Score	Moderate_Severe in training set
FCFP_10	-1977641857	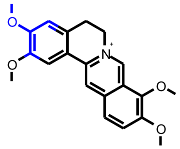 [*][c](:[*]):[c](OC):[cH]:[*]	-0.78	4 out of 15
FCFP_10	-1861645784	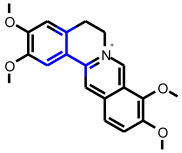 [*][c](:[*]):[c](:[cH]:[*])[c](:[*]):[*]	-0.598	9 out of 26
FCFP_10	-614861946	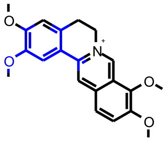 [*]O[c]1:[cH]:[c]([c](:[*]):[*]):[c]([*]):[*]:[c]:1[*]	-0.507	0 out of 1

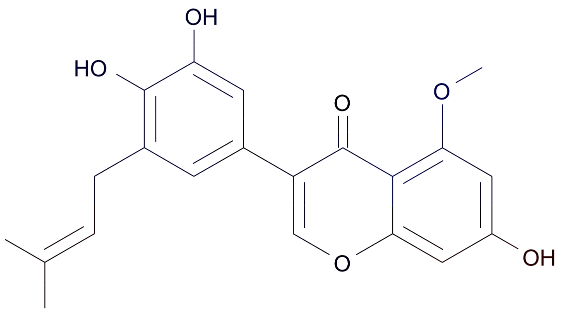

$C_{21}H_{20}O_6$
Molecular Weight: 368.3799
ALogP: 3.98
Rotatable Bonds: 4
Acceptors: 6
Donors: 3

Model Prediction

Prediction: Mild
Probability: 0.762
Enrichment: 1.11
Bayesian Score: -2.2
Mahalanobis Distance: 11.4
Mahalanobis Distance p-value: 0.000838

Prediction: Positive if the Bayesian score is above the estimated best cutoff value from minimizing the false positive and false negative rate.
Probability: The esimated probability that the sample is in the positive category. This assumes that the Bayesian score follows a normal distribution and is different from the prediction using a cutoff.
Enrichment: An estimate of enrichment, that is, the increased likelihood (versus random) of this sample being in the category.
Bayesian Score: The standard Laplacian-modified Bayesian score.
Mahalanobis Distance: The Mahalanobis distance (MD) is the distance to the center of the training data. The larger the MD, the less trustworthy the prediction.
Mahalanobis Distance p-value: The p-value gives the fraction of training data with an MD greater than or equal to the one for the given sample, assuming normally distributed data. The smaller the p-value, the less trustworthy the prediciton. For highly non-normal X properties (e.g., fingerprints), the MD p-value is wildly inaccurate.

Structural Similar Compounds

Name	4;4'-DIAMINO-1;1'-DIANTHRIMIDE	ANTHRAQUINONE; 1-AMINO-4-HYDROXY-2-PHENOXY-	ANTHRAQUINONE; 1-((2-HYDROXYETHYL)AMINO)-4-(METHYLAMINO)-
Structure	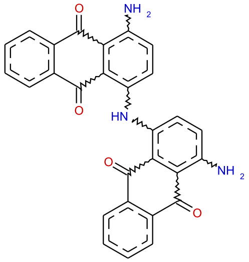	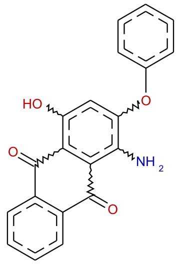	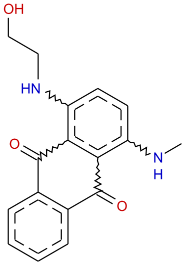
Actual Endpoint	Mild	Mild	Mild
Predicted Endpoint	Mild	Mild	Mild
Distance	0.683	0.729	0.739
Reference	28ZPAK-;125;72	28ZPAK 239;72	28ZPAK 245;72

Model Applicability

Unknown features are fingerprint features in the query molecule, but not found or appearing too infrequently in the training set.

1. All properties and OPS components are within expected ranges.

Feature Contribution

Top features for positive contribution

Fingerprint	Bit/Smiles	Feature Structure	Score	Moderate_Severe in training set
FCFP_10	-158888774	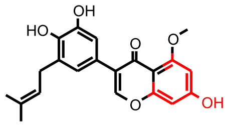 [*][c]1:[*]:[c]([*]):[cH]:[c](O):[cH]:1	0.356	24 out of 25

FCFP_10	7	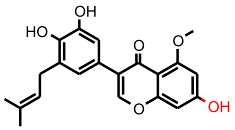 [*]O	0.219	117 out of 142
FCFP_10	436886043	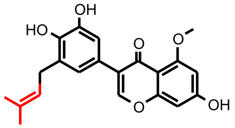 [*]C=C(C)C	0.16	25 out of 32
**Top Features for negative contribution**				
**Fingerprint**	**Bit/Smiles**	**Feature Structure**	**Score**	**Moderate_Severe in training set**
FCFP_10	-201608392	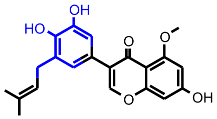 [*]C[c]1:[cH]:[*]:[cH]:[c](O):[c]:1O	-0.842	0 out of 2
FCFP_10	-1977641857	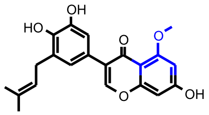 [*][c](:[*]):[c](OC):[cH]:[*]	-0.78	4 out of 15
FCFP_10	-1099193755	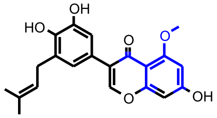 [*]C(=[*])[c]1:[c]([*]):[*]:[c]([*]):[cH]:[c]:1OC	-0.361	2 out of 5

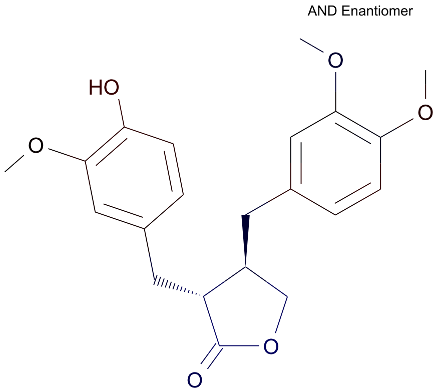

$C_{21}H_{24}O_6$
Molecular Weight: 372.41166
ALogP: 3.743
Rotatable Bonds: 7
Acceptors: 6
Donors: 1

Structural Similar Compounds

Name	COLCHICINE	1-BENZOYLAMINO-4-METHOXY-5-CHLORANTHRAQUINONE	Cinchoninamide; 2-butoxy-N-(2-(diethylamino)ethyl)-; monohydrochloride
Structure	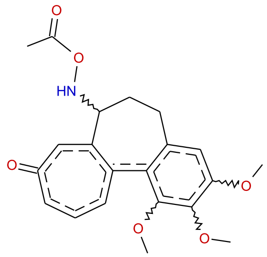	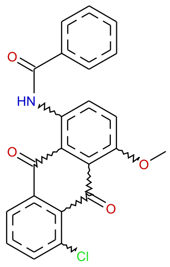	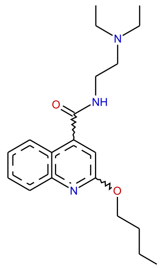
Actual Endpoint	Moderate_Severe	Mild	Moderate_Severe
Predicted Endpoint	Moderate_Severe	Mild	Moderate_Severe
Distance	0.628	0.682	0.696
Reference	AJOPAA 31;837;48	28ZPAK-;90;72	Arzneimittel-Forschung 8;181;58

Model Prediction

Prediction: Mild
Probability: 0.797
Enrichment: 1.16
Bayesian Score: -1.11
Mahalanobis Distance: 10.2
Mahalanobis Distance p-value: 0.0569

Prediction: Positive if the Bayesian score is above the estimated best cutoff value from minimizing the false positive and false negative rate.
Probability: The esimated probability that the sample is in the positive category. This assumes that the Bayesian score follows a normal distribution and is different from the prediction using a cutoff.
Enrichment: An estimate of enrichment, that is, the increased likelihood (versus random) of this sample being in the category.
Bayesian Score: The standard Laplacian-modified Bayesian score.
Mahalanobis Distance: The Mahalanobis distance (MD) is the distance to the center of the training data. The larger the MD, the less trustworthy the prediction.
Mahalanobis Distance p-value: The p-value gives the fraction of training data with an MD greater than or equal to the one for the given sample, assuming normally distributed data. The smaller the p-value, the less trustworthy the prediciton. For highly non-normal X properties (e.g., fingerprints), the MD p-value is wildly inaccurate.

Model Applicability

Unknown features are fingerprint features in the query molecule, but not found or appearing too infrequently in the training set.

1. All properties and OPS components are within expected ranges.

Feature Contribution

Top features for positive contribution

Fingerprint	Bit/Smiles	Feature Structure	Score	Moderate_Severe in training set
FCFP_10	1985089045	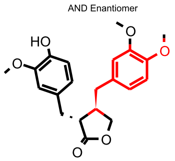 [*]O[c]1:[cH]:[cH]:[c](CC([*])[*]):[cH]:[c]:1[*]	0.385	16 out of 16

FCFP_10	-497728148	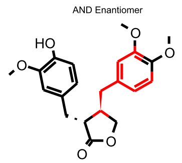<br>[*]C([*])C[c]1:[cH]:[cH]:[*]:[c]([*]):[cH]:1	0.356	24 out of 25
FCFP_10	1028934530	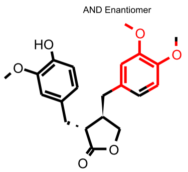<br>[*]O[c]1:[cH]:[*]:[c](:[*]):[cH]:[c]:1OC	0.256	2 out of 2
**Top Features for negative contribution**				
**Fingerprint**	**Bit/Smiles**	**Feature Structure**	**Score**	**Moderate_Severe in training set**
FCFP_10	-1084442457	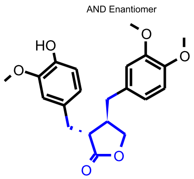<br>[*]C[C@H]1COC(=O)[C@@H]1C[*]	-1.09	0 out of 3
FCFP_10	-1977641857	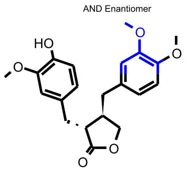<br>[*][c](:[*]):[c](OC):[cH]:[*]	-0.78	4 out of 15
FCFP_10	1588282714	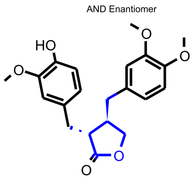<br>[*]C[C@H]1COC(=[*])[C@@H]1[*]	-0.427	10 out of 24

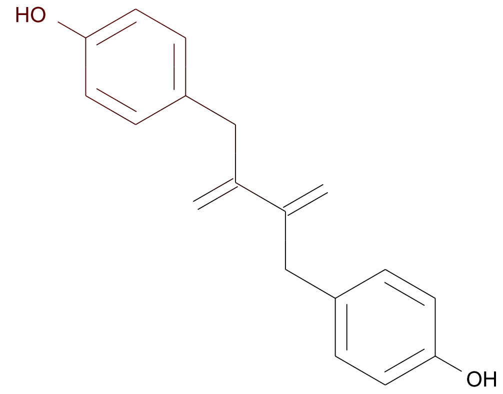

$C_{18}H_{18}O_2$
Molecular Weight: 266.33432
ALogP: 4.8
Rotatable Bonds: 5
Acceptors: 2
Donors: 2

Model Prediction

Prediction: Moderate_Severe
Probability: 0.857
Enrichment: 1.24
Bayesian Score: 2.07
Mahalanobis Distance: 6.93
Mahalanobis Distance p-value: 0.999

Prediction: Positive if the Bayesian score is above the estimated best cutoff value from minimizing the false positive and false negative rate.
Probability: The esimated probability that the sample is in the positive category. This assumes that the Bayesian score follows a normal distribution and is different from the prediction using a cutoff.
Enrichment: An estimate of enrichment, that is, the increased likelihood (versus random) of this sample being in the category.
Bayesian Score: The standard Laplacian-modified Bayesian score.
Mahalanobis Distance: The Mahalanobis distance (MD) is the distance to the center of the training data. The larger the MD, the less trustworthy the prediction.
Mahalanobis Distance p-value: The p-value gives the fraction of training data with an MD greater than or equal to the one for the given sample, assuming normally distributed data. The smaller the p-value, the less trustworthy the prediciton. For highly non-normal X properties (e.g., fingerprints), the MD p-value is wildly inaccurate.

Structural Similar Compounds

Name	PHENOL;4;4'-ISOPROPYLIDENEDI-	PHENOL;2;2'-METHYLENEBIS(4-CHLORO-	P-PHENYLENEDIAMINE; N;N'-DIPHENYL-
Structure	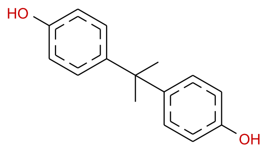	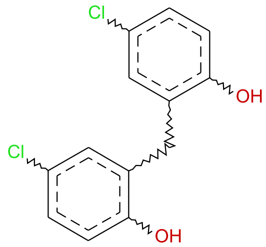	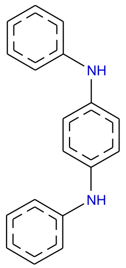
Actual Endpoint	Moderate_Severe	Moderate_Severe	Mild
Predicted Endpoint	Moderate_Severe	Moderate_Severe	Mild
Distance	0.507	0.510	0.555
Reference	28ZPAK-;58;72	28ZPAK-;82;72	28ZPAK-;73;72

Model Applicability

Unknown features are fingerprint features in the query molecule, but not found or appearing too infrequently in the training set.

1. All properties and OPS components are within expected ranges.

Feature Contribution

Top features for positive contribution

Fingerprint	Bit/Smiles	Feature Structure	Score	Moderate_Severe in training set
FCFP_10	-1066794953	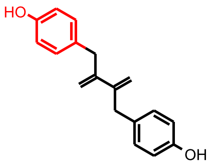 [*][c]1:[cH]:[cH]:[c](O):[cH]:[cH]:1	0.378	13 out of 13

FCFP_10	-158888774	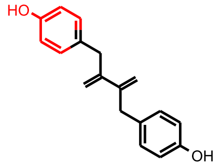 [*][c]1:[*]:[c]([*]):[cH]:[c](O):[cH]:1	0.356	24 out of 25
FCFP_10	-497728148	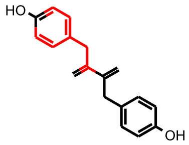 [*]C([*])C[c]1:[cH]:[cH]:[*]:[c]([*]):[cH]:1	0.356	24 out of 25

Top Features for negative contribution

**Fingerprint**	**Bit/Smiles**	**Feature Structure**	**Score**	**Moderate_Severe in training set**
FCFP_10	-453677277	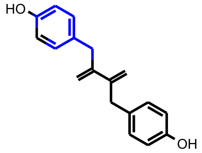 [*]C[c]1:[cH]:[*]:[c]([*]):[cH]:[cH]:1	-0.13	153 out of 264
FCFP_10	203677720	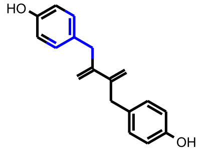 [*]C([*])[c](:[cH]:[*]):[c](:[*]):[*]	-0.0982	191 out of 319
FCFP_10	129344189	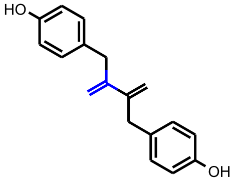 [*]C(=C)[*]	-0.0714	67 out of 109

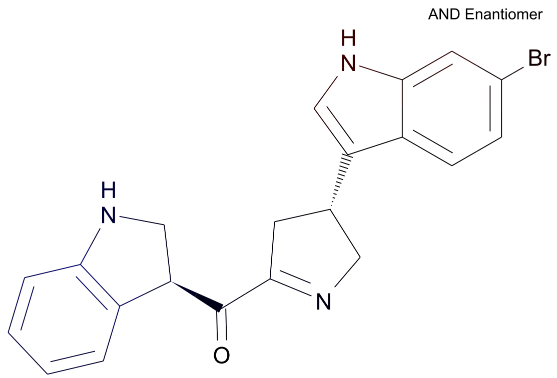

$C_{21}H_{18}BrN_3O$
Molecular Weight: 408.29112
ALogP: 3.919
Rotatable Bonds: 3
Acceptors: 3
Donors: 2

Model Prediction

Prediction: Mild
Probability: 0.785
Enrichment: 1.14
Bayesian Score: -1.52
Mahalanobis Distance: 11.8
Mahalanobis Distance p-value: 0.000179

Prediction: Positive if the Bayesian score is above the estimated best cutoff value from minimizing the false positive and false negative rate.
Probability: The esimated probability that the sample is in the positive category. This assumes that the Bayesian score follows a normal distribution and is different from the prediction using a cutoff.
Enrichment: An estimate of enrichment, that is, the increased likelihood (versus random) of this sample being in the category.
Bayesian Score: The standard Laplacian-modified Bayesian score.
Mahalanobis Distance: The Mahalanobis distance (MD) is the distance to the center of the training data. The larger the MD, the less trustworthy the prediction.
Mahalanobis Distance p-value: The p-value gives the fraction of training data with an MD greater than or equal to the one for the given sample, assuming normally distributed data. The smaller the p-value, the less trustworthy the prediciton. For highly non-normal X properties (e.g., fingerprints), the MD p-value is wildly inaccurate.

Structural Similar Compounds

Name	BENZAMIDE; N-(5-CHLORO-1-ANTHRAQUINONYL)-	1-AMINO-4-BENZOYLAMINO-ANTHRAQUINONE	1-BENZOYLAMINO-4-METHOXY-5-CHLORANTHRAQUINONE
Structure	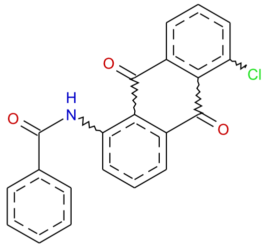	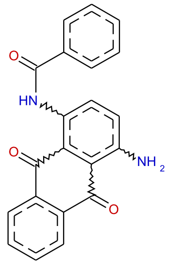	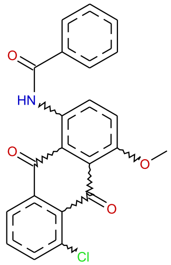
Actual Endpoint	Mild	Mild	Mild
Predicted Endpoint	Mild	Mild	Mild
Distance	0.679	0.685	0.694
Reference	28ZPAK 89;72	28ZPAK-;124;72	28ZPAK-;90;72

Model Applicability

Unknown features are fingerprint features in the query molecule, but not found or appearing too infrequently in the training set.

1. All properties and OPS components are within expected ranges.

Feature Contribution

Top features for positive contribution

Fingerprint	Bit/Smiles	Feature Structure	Score	Moderate_Severe in training set
FCFP_10	-745491832	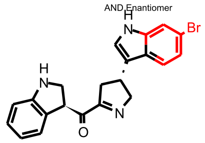 [*]:[c]1:[*]:[cH]:[cH]:[c](Br):[cH]:1	0.304	29 out of 32

FCFP_10	-1272709286	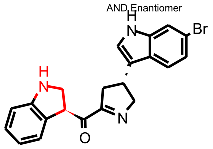<br>[*][C@H]1[*]:[*]NC1	0.285	234 out of 266
FCFP_10	1673997923	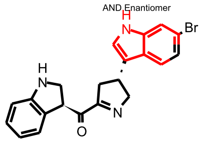<br>[*]:[c]1:[nH]:[c]2:[c<br>H]:[cH]:[*]:[cH]:[c]<br>:2:[c]:1:[*]	0.256	2 out of 2

Top Features for negative contribution

**Fingerprint**	**Bit/Smiles**	**Feature Structure**	**Score**	**Moderate_Severe in training set**
FCFP_10	565968762	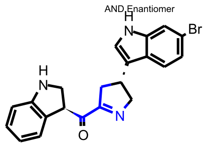<br>[*]C(=[*])C1=N[*][*]C<br>1	-0.372	17 out of 38
FCFP_10	1177578141	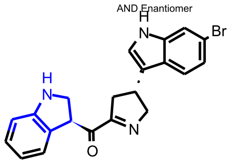<br>[*][C@@H]1CN[c](:[cH]<br>:[*]):[c]1:[*]	-0.361	2 out of 5
FCFP_10	1011367537	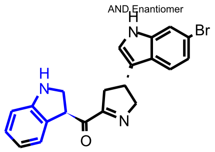<br>[*][C@@H]1CN[c]2:[cH]<br>:[cH]:[*]:[cH]:[c]1:<br>2	-0.329	4 out of 9

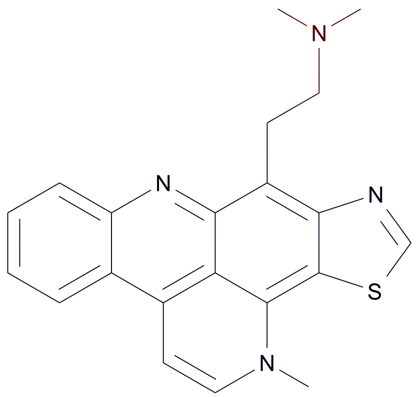

$C_{21}H_{20}N_4S$
Molecular Weight: 360.4753
ALogP: 3.682
Rotatable Bonds: 3
Acceptors: 4
Donors: 0

Model Prediction

Prediction: Mild
Probability: 0.808
Enrichment: 1.17
Bayesian Score: -0.697
Mahalanobis Distance: 11.9
Mahalanobis Distance p-value: 0.000124

Prediction: Positive if the Bayesian score is above the estimated best cutoff value from minimizing the false positive and false negative rate.
Probability: The esimated probability that the sample is in the positive category. This assumes that the Bayesian score follows a normal distribution and is different from the prediction using a cutoff.
Enrichment: An estimate of enrichment, that is, the increased likelihood (versus random) of this sample being in the category.
Bayesian Score: The standard Laplacian-modified Bayesian score.
Mahalanobis Distance: The Mahalanobis distance (MD) is the distance to the center of the training data. The larger the MD, the less trustworthy the prediction.
Mahalanobis Distance p-value: The p-value gives the fraction of training data with an MD greater than or equal to the one for the given sample, assuming normally distributed data. The smaller the p-value, the less trustworthy the prediciton. For highly non-normal X properties (e.g., fingerprints), the MD p-value is wildly inaccurate.

Structural Similar Compounds

Name	7H-BENZIMIDAZO(2;1-a)BENZ(de)ISOQUINOLIN-7-ONE; 10-METHOXY-	PHOSPHOROUS ACID; TRIPHENYL ESTER	PROPANE;2;2-BIS(P-2;3-EPOXYPROPOXY)PHENYL)-
Structure	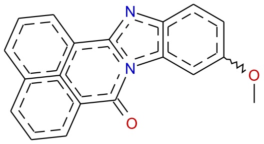	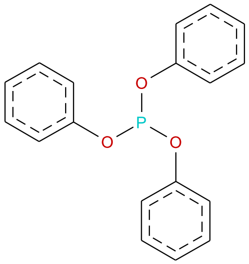	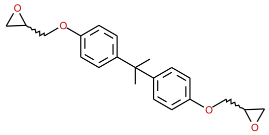
Actual Endpoint	Mild	Mild	Moderate_Severe
Predicted Endpoint	Mild	Mild	Moderate_Severe
Distance	0.608	0.619	0.634
Reference	28ZPAK 147;72	28ZPAK-;205;72	28ZPAK-;137;72

Model Applicability

Unknown features are fingerprint features in the query molecule, but not found or appearing too infrequently in the training set.

1. All properties and OPS components are within expected ranges.
2. Unknown FCFP_2 feature: -124685461: [*]1:[*]:s:c:n:1

Feature Contribution

Top features for positive contribution

Fingerprint	Bit/Smiles	Feature Structure	Score	Moderate_Severe in training set
FCFP_10	-14048077	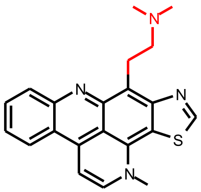 [*]CCN(C)C	0.386	17 out of 17

FCFP_10	-587569116	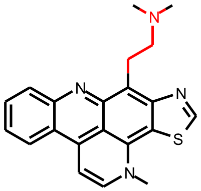<br>[*]N1[*][*]CC1	0.335	66 out of 71
FCFP_10	136388789	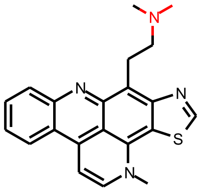<br>[*]N([*])C	0.331	28 out of 30
**Top Features for negative contribution**				
**Fingerprint**	**Bit/Smiles**	**Feature Structure**	**Score**	**Moderate_Severe in training set**
FCFP_10	-1251706662	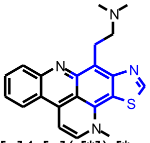<br>[*]C[c]1:[c](:[*]):[*<br>]:[c]([*]):[c]2:s:[c<br>H]:n:[c]:1:2	-0.507	0 out of 1
FCFP_10	451877515	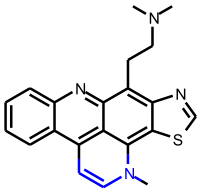<br>[*]OC=C([*])[*]	-0.35	16 out of 35
FCFP_10	-1320007763	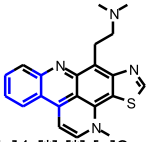<br>[*]:[c]1:[*]:[*]:[c]2<br>:[*]:[cH]:[cH]:[cH]:<br>[c]:1:2	-0.316	19 out of 40

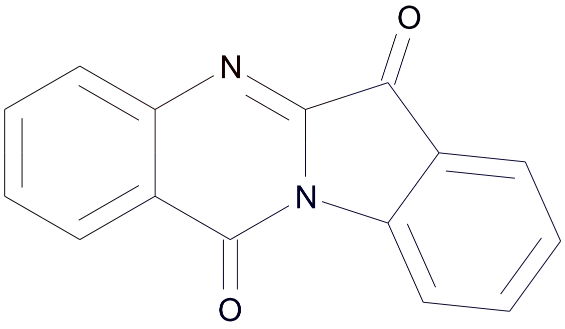

$C_{15}H_8N_2O_2$
Molecular Weight: 248.23621
ALogP: 2.331
Rotatable Bonds: 0
Acceptors: 3
Donors: 0

Model Prediction

Prediction: Mild
Probability: 0.781
Enrichment: 1.13
Bayesian Score: -1.65
Mahalanobis Distance: 5.82
Mahalanobis Distance p-value: 1

Prediction: Positive if the Bayesian score is above the estimated best cutoff value from minimizing the false positive and false negative rate.
Probability: The esimated probability that the sample is in the positive category. This assumes that the Bayesian score follows a normal distribution and is different from the prediction using a cutoff.
Enrichment: An estimate of enrichment, that is, the increased likelihood (versus random) of this sample being in the category.
Bayesian Score: The standard Laplacian-modified Bayesian score.
Mahalanobis Distance: The Mahalanobis distance (MD) is the distance to the center of the training data. The larger the MD, the less trustworthy the prediction.
Mahalanobis Distance p-value: The p-value gives the fraction of training data with an MD greater than or equal to the one for the given sample, assuming normally distributed data. The smaller the p-value, the less trustworthy the prediciton. For highly non-normal X properties (e.g., fingerprints), the MD p-value is wildly inaccurate.

Structural Similar Compounds

Name	ANTHRAQUINONE;1;5-DIMETHOXY	3;4-DIMETHYLPHENYLMALEIMIDE	QUINOXALINE-6-CARBONYLCHLORIDE;2;3-DICHLORO-
Structure	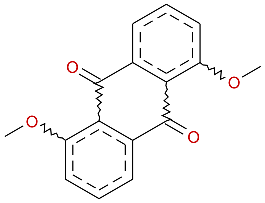	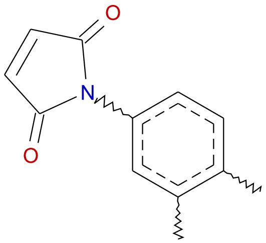	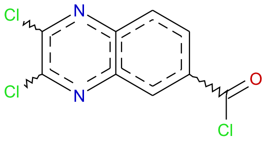
Actual Endpoint	Mild	Moderate_Severe	Moderate_Severe
Predicted Endpoint	Mild	Moderate_Severe	Moderate_Severe
Distance	0.559	0.565	0.565
Reference	28ZPAK-;113;72	AIHAAP 23;95;62	28ZPAK-;150;72

Model Applicability

Unknown features are fingerprint features in the query molecule, but not found or appearing too infrequently in the training set.

1. All properties and OPS components are within expected ranges.

Feature Contribution

Top features for positive contribution

Fingerprint	Bit/Smiles	Feature Structure	Score	Moderate_Severe in training set
FCFP_10	580453787	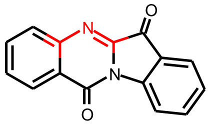 [*]C(=N[c](:[*]):[*])[*]	0.256	2 out of 2

FCFP_10	1150094517	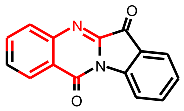 [*]C1=N[c]2:[cH]:[cH]:[*]:[cH]:[c]:2C(=[*])[*]1	0.186	1 out of 1
FCFP_10	675769755	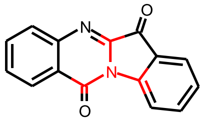 [*]=CN(C)[c](:[*]):[*]	0.116	12 out of 16
**Top Features for negative contribution**				
**Fingerprint**	**Bit/Smiles**	**Feature Structure**	**Score**	**Moderate_Severe in training set**
FCFP_10	-1549163031	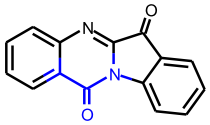 [*]N([*])C(=O)[c](:[*]):[*]	-0.657	5 out of 16
FCFP_10	-1698724694	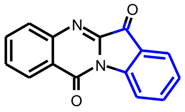 [*][C@@H]1[*][*][c]2:[cH]:[cH]:[cH]:[cH]:[c]1:2	-0.284	53 out of 107
FCFP_10	991735244	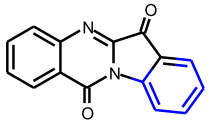 [*]:[c]1:[*]:[cH]:[cH]:[cH]:[cH]:1	-0.185	130 out of 237

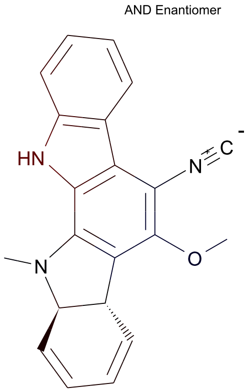

$C_{21}H_{17}N_3O$
Molecular Weight: 327.37918
ALogP: 4.078
Rotatable Bonds: 1
Acceptors: 2
Donors: 1

Structural Similar Compounds

Name	Anthraquinone; 1-bromo-4-(methylamino)-	BENZAMIDE; N-(5-CHLORO-1-ANTHRAQUINONYL)-	Anthraquinone; 1-amino-2;4-dibromo-
Structure	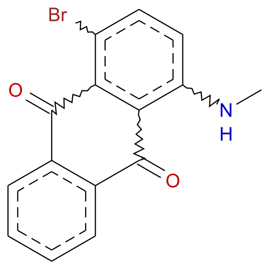	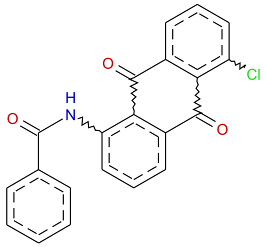	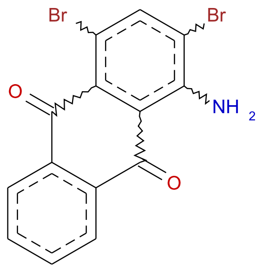
Actual Endpoint	Mild	Mild	Mild
Predicted Endpoint	Mild	Mild	Mild
Distance	0.595	0.596	0.637
Reference	Prehled Prumyslove Toxikologie; Organicke Latky; Marhold; J. pp 566;86	28ZPAK 89;72	Prehled Prumyslove Toxikologie; Organicke Latky; Marhold; J. pp 565;86

Model Prediction

Prediction: Moderate_Severe
Probability: 0.832
Enrichment: 1.21
Bayesian Score: 0.66
Mahalanobis Distance: 9.65
Mahalanobis Distance p-value: 0.199

Prediction: Positive if the Bayesian score is above the estimated best cutoff value from minimizing the false positive and false negative rate.
Probability: The esimated probability that the sample is in the positive category. This assumes that the Bayesian score follows a normal distribution and is different from the prediction using a cutoff.
Enrichment: An estimate of enrichment, that is, the increased likelihood (versus random) of this sample being in the category.
Bayesian Score: The standard Laplacian-modified Bayesian score.
Mahalanobis Distance: The Mahalanobis distance (MD) is the distance to the center of the training data. The larger the MD, the less trustworthy the prediction.
Mahalanobis Distance p-value: The p-value gives the fraction of training data with an MD greater than or equal to the one for the given sample, assuming normally distributed data. The smaller the p-value, the less trustworthy the prediciton. For highly non-normal X properties (e.g., fingerprints), the MD p-value is wildly inaccurate.

Model Applicability

Unknown features are fingerprint features in the query molecule, but not found or appearing too infrequently in the training set.

1. All properties and OPS components are within expected ranges.
2. Unknown FCFP_2 feature: 4: [*]#[C-]
3. Unknown FCFP_2 feature: -828984032: [*][c](:[*]):[c]([N+]#[*]):[c](:[*]):[*]
4. Unknown FCFP_2 feature: 1934974835: [*]:[c](:[*])[N+]#[C-]
5. Unknown FCFP_2 feature: -1487147388: [*][N+]#[C-]

Feature Contribution

Top features for positive contribution

Fingerprint	Bit/Smiles	Feature Structure	Score	Moderate_Severe in training set

FCFP_10	-525166915	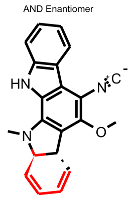 [*][C@H]1[*]C=CC=C1	0.317	4 out of 4
FCFP_10	155061250	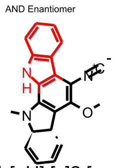 [*]:[c]1:[nH]:[c]2:[c<br>H]:[cH]:[cH]:[cH]:[c<br>]:2:[c]:1:[*]	0.256	2 out of 2
FCFP_10	1673997923	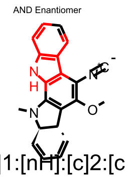 [*]:[c]1:[nH]:[c]2:[c<br>H]:[cH]:[*]:[cH]:[c]<br>:2:[c]:1:[*]	0.256	2 out of 2
**Top Features for negative contribution**				
**Fingerprint**	**Bit/Smiles**	**Feature Structure**	**Score**	**Moderate_Severe in training set**
FCFP_10	-1977641857	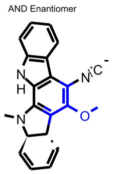 [*][c](:[*]):[c](OC):<br>[cH]:[*]	-0.78	4 out of 15
FCFP_10	-1320007763	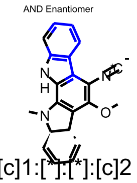 [*]:[c]1:[*]:[*]:[c]2<br>:[*]:[cH]:[cH]:[cH]:<br>[c]:1:2	-0.316	19 out of 40

FCFP_10	136627117	AND Enantiomer 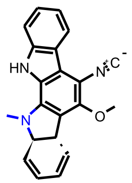 [*]OC	-0.316	46 out of 96

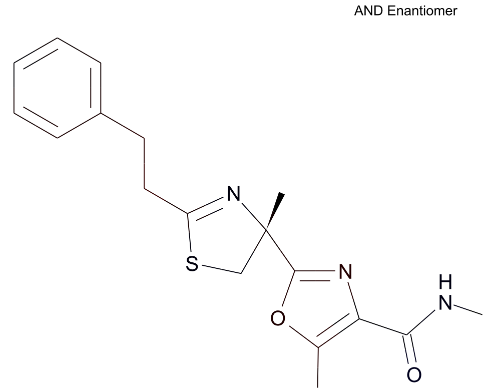

$C_{18}H_{21}N_3O_2S$
Molecular Weight: 343.44324
ALogP: 2.716
Rotatable Bonds: 5
Acceptors: 4
Donors: 1

Structural Similar Compounds

Name	ACETIC ACID; 2-(-CHLOROMETHY-1-NAPHTHYLTHIO)-	Anthraquinone; 1-bromo-4-(methylamino)-	BENZOIC ACID; 5-(CHLOROSULFONYL)-2;4-DICHLORO-
Structure	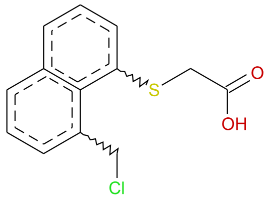	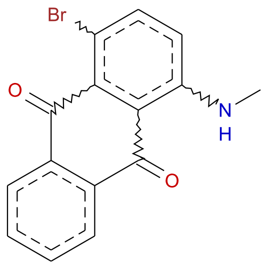	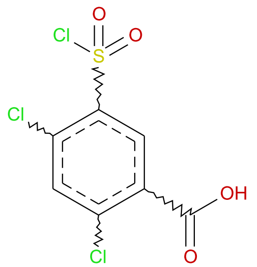
Actual Endpoint	Moderate_Severe	Mild	Moderate_Severe
Predicted Endpoint	Moderate_Severe	Mild	Moderate_Severe
Distance	0.638	0.647	0.649
Reference	28ZPAK-;173;72	Prehled Prumyslove Toxikologie; Organicke Latky; Marhold; J. pp 566;86	FCTOD7 20;573;82

Model Prediction

Prediction: Moderate_Severe
Probability: 0.831
Enrichment: 1.21
Bayesian Score: 0.566
Mahalanobis Distance: 9.82
Mahalanobis Distance p-value: 0.14

Prediction: Positive if the Bayesian score is above the estimated best cutoff value from minimizing the false positive and false negative rate.
Probability: The esimated probability that the sample is in the positive category. This assumes that the Bayesian score follows a normal distribution and is different from the prediction using a cutoff.
Enrichment: An estimate of enrichment, that is, the increased likelihood (versus random) of this sample being in the category.
Bayesian Score: The standard Laplacian-modified Bayesian score.
Mahalanobis Distance: The Mahalanobis distance (MD) is the distance to the center of the training data. The larger the MD, the less trustworthy the prediction.
Mahalanobis Distance p-value: The p-value gives the fraction of training data with an MD greater than or equal to the one for the given sample, assuming normally distributed data. The smaller the p-value, the less trustworthy the prediciton. For highly non-normal X properties (e.g., fingerprints), the MD p-value is wildly inaccurate.

Model Applicability

Unknown features are fingerprint features in the query molecule, but not found or appearing too infrequently in the training set.

1. All properties and OPS components are within expected ranges.
2. Unknown FCFP_2 feature: -836603894: [*]:[c](:[*])[C@]1(C)C[*][*]=N1

Feature Contribution

Top features for positive contribution

Fingerprint	Bit/Smiles	Feature Structure	Score	Moderate_Severe in training set
FCFP_10	1388176727	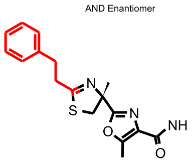 [*]C(=[*])CC[c]1:[cH]:[cH]:[cH]:[cH]:[cH]:1	0.389	19 out of 19

FCFP_10	-497728148	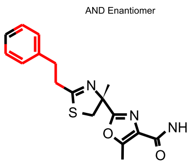 [*]C([*])C[c]1:[cH]:[cH]:[*]:[c]([*]):[cH]:1	0.356	24 out of 25
FCFP_10	-1539162406	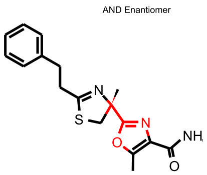 [*]C([*])([*])[c]1:o:[*]:[*]:n:1	0.294	3 out of 3
**Top Features for negative contribution**				
**Fingerprint**	**Bit/Smiles**	**Feature Structure**	**Score**	**Moderate_Severe in training set**
FCFP_10	-1549103449	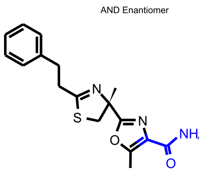 [*]NC(=O)[c](:[*]):[*]	-0.504	2 out of 6
FCFP_10	-1698724694	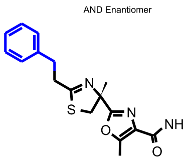 [*][C@@H]1[*][*][c]2:[cH]:[cH]:[cH]:[cH]:[c]1:2	-0.284	53 out of 107
FCFP_10	136686699	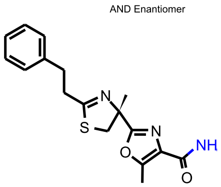 [*]NC	-0.243	6 out of 12

Remdesivir

TOPKAT_Ocular_Irritancy_Mild_vs_Moderate_Severe

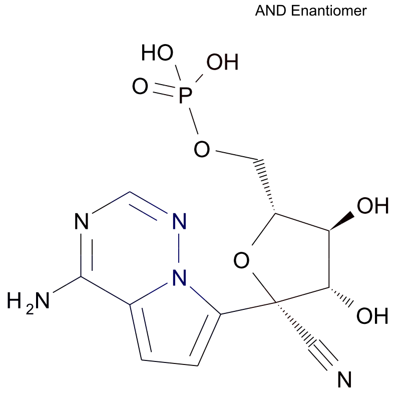

$C_{12}H_{14}N_5O_7P$
Molecular Weight: 371.24262
ALogP: -1.502
Rotatable Bonds: 4
Acceptors: 11
Donors: 5

Structural Similar Compounds

Name	1;3;6-NAPHTHALENE TRISULFONIC ACID;7-AMINO-	Methanol; (s-triazine-2;4;6-triyltrinitrilo)hexa-	2;2'-Biphenyldisulfonic acid; 4;4'-diamino-
Structure	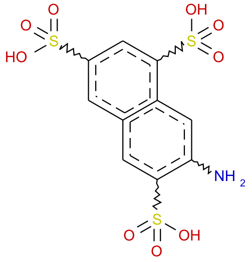	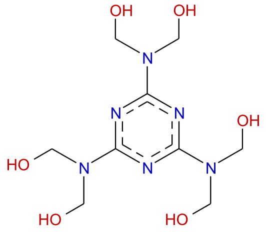	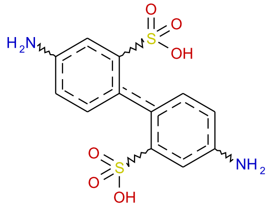
Actual Endpoint	Mild	Moderate_Severe	Mild
Predicted Endpoint	Mild	Moderate_Severe	Mild
Distance	0.776	0.802	0.878
Reference	28ZPAK-;190;72	Prehled Prumyslove Toxikologie; Organicke Latky; Marhold; J. -;876;86	Prehled Prumyslove Toxikologie; Organicke Latky; Marhold; J. pp 1061;86

Model Prediction

Prediction: Mild
Probability: 0.789
Enrichment: 1.15
Bayesian Score: -1.39
Mahalanobis Distance: 13.8
Mahalanobis Distance p-value: 1.42e-009

Prediction: Positive if the Bayesian score is above the estimated best cutoff value from minimizing the false positive and false negative rate.
Probability: The esimated probability that the sample is in the positive category. This assumes that the Bayesian score follows a normal distribution and is different from the prediction using a cutoff.
Enrichment: An estimate of enrichment, that is, the increased likelihood (versus random) of this sample being in the category.
Bayesian Score: The standard Laplacian-modified Bayesian score.
Mahalanobis Distance: The Mahalanobis distance (MD) is the distance to the center of the training data. The larger the MD, the less trustworthy the prediction.
Mahalanobis Distance p-value: The p-value gives the fraction of training data with an MD greater than or equal to the one for the given sample, assuming normally distributed data. The smaller the p-value, the less trustworthy the prediciton. For highly non-normal X properties (e.g., fingerprints), the MD p-value is wildly inaccurate.

Model Applicability

Unknown features are fingerprint features in the query molecule, but not found or appearing too infrequently in the training set.

1. OPS PC17 out of range. Value: 4.6782. Training min, max, SD, explained variance: -4.348, 3.9505, 1.094, 0.0146.
2. Unknown FCFP_2 feature: 472180098: [*]OP(=O)(O)O
3. Unknown FCFP_2 feature: -836603894: [*]:[c](:[*])[C@]1(C)C[*][*]=N1
4. Unknown FCFP_2 feature: -124685461: [*]1:[*]:s:c:n:1
5. Unknown FCFP_2 feature: -1151884458: [*]:n:[c](N):[c](:[*]):[*]

Feature Contribution

Top features for positive contribution

Fingerprint	Bit/Smiles	Feature Structure	Score	Moderate_Severe in training set

FCFP_10	1070061035	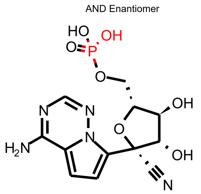 [*]P(=[*])([*])O	0.239	284 out of 338
FCFP_10	-1539132615	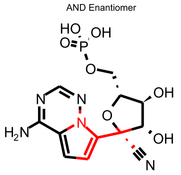 [*]:n:[c](C):[c](:[*]):[*]	0.224	11 out of 13
FCFP_10	-1043250487	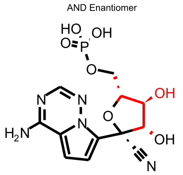 [*][C@@H]1[*][*][C@H] ([*])C1O	0.22	62 out of 75
**Top Features for negative contribution**				
**Fingerprint**	**Bit/Smiles**	**Feature Structure**	**Score**	**Moderate_Severe in training set**
FCFP_10	4427049	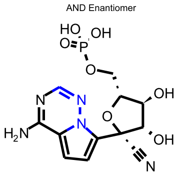 [*]:[cH]:n:n(:[*]):[*]	-1.29	0 out of 4
FCFP_10	-332197802	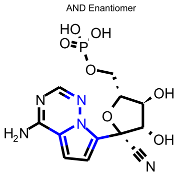 [*][c]1:[*]:[*]:[c](: [*]):n:1:n:[*]	-0.507	0 out of 1

FCFP_10	713358128	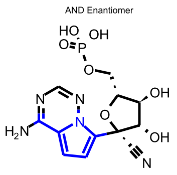 [*]:n:[c]1:[cH]:[cH]:[cH]:[*]:[c]:1:[*]	-0.307	8 out of 17

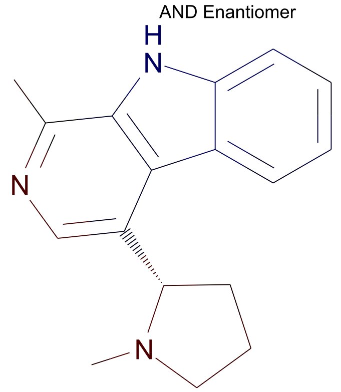

$C_{17}H_{19}N_3$

Molecular Weight: 265.35286

ALogP: 3.018

Rotatable Bonds: 1

Acceptors: 2

Donors: 1

Structural Similar Compounds

Name	3-AMINOPYRENE	p-Anisidine; N-cyclohexyl-	Ethanol; 2-(2;4-dichlorophenoxy)-
Structure	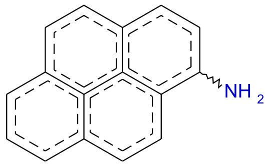	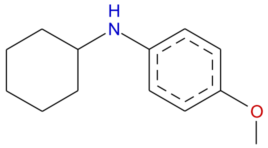	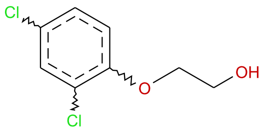
Actual Endpoint	Severe	Moderate	Severe
Predicted Endpoint	Severe	Moderate	Severe
Distance	0.601	0.609	0.615
Reference	AIHAAP 30;470;69	Prehled Prumyslove Toxikologie; Organicke Latky; Marhold; J. -;723;86	Prehled Prumyslove Toxikologie; Organicke Latky; Marhold; J. -;530;86

Model Prediction

Prediction: Severe

Probability: 0.736

Enrichment: 1.19

Bayesian Score: 1.55

Mahalanobis Distance: 5.29

Mahalanobis Distance p-value: 1

Prediction: Positive if the Bayesian score is above the estimated best cutoff value from minimizing the false positive and false negative rate.
Probability: The esimated probability that the sample is in the positive category. This assumes that the Bayesian score follows a normal distribution and is different from the prediction using a cutoff.
Enrichment: An estimate of enrichment, that is, the increased likelihood (versus random) of this sample being in the category.
Bayesian Score: The standard Laplacian-modified Bayesian score.
Mahalanobis Distance: The Mahalanobis distance (MD) is the distance to the center of the training data. The larger the MD, the less trustworthy the prediction.
Mahalanobis Distance p-value: The p-value gives the fraction of training data with an MD greater than or equal to the one for the given sample, assuming normally distributed data. The smaller the p-value, the less trustworthy the prediciton. For highly non-normal X properties (e.g., fingerprints), the MD p-value is wildly inaccurate.

Model Applicability

Unknown features are fingerprint features in the query molecule, but not found or appearing too infrequently in the training set.

1. All properties and OPS components are within expected ranges.

Feature Contribution

Top features for positive contribution

Fingerprint	Bit/Smiles	Feature Structure	Score	Severe in training set
SCFP_12	-496201075	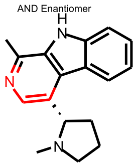 [*][c](:[*]):[cH]:n:[*]	0.378	12 out of 13

SCFP_12	-673674794	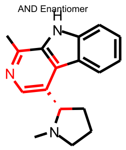 [*]C([*])[c]1:[cH]:n:[c]([*]):[*]:[c]:1:[*]	0.376	4 out of 4
SCFP_12	1194442465	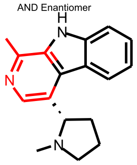 [*][c]1:[*]:[c](:[*]):[c](C):n:[cH]:1	0.376	4 out of 4

Top Features for negative contribution

Fingerprint	Bit/Smiles	Feature Structure	Score	Severe in training set
SCFP_12	125623987	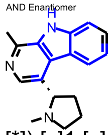 [*][c](:[*]):[c]1:[c](:[*]):[nH]:[c]2:[cH]:[*]:[cH]:[cH]:[c]:1:2	-0.475	0 out of 1
SCFP_12	-710590418	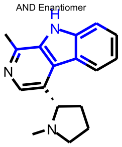 [*][c](:[*]):[c]1:[nH]:[c](:[cH]:[*]):[c](:[*]):[c]:1:[*]	-0.475	0 out of 1
SCFP_12	2091994126	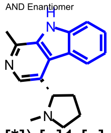 [*][c](:[*]):[c]1:[c](:[*]):[nH]:[c]2:[cH]:[cH]:[cH]:[cH]:[c]:1:2	-0.475	0 out of 1

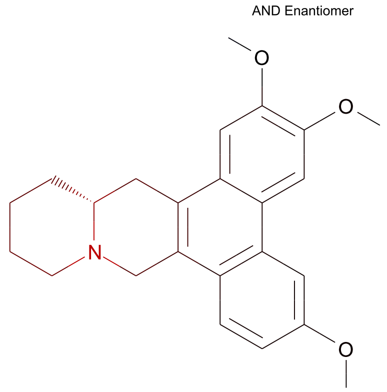

$C_{24}H_{27}NO_3$
Molecular Weight: 377.47608
ALogP: 4.691
Rotatable Bonds: 3
Acceptors: 4
Donors: 0

Structural Similar Compounds

Name	Carbamic acid; N-methyl-N-(1-(3;5-xylyloxy)-2-propyl)-;	PROPANE;2;2-BIS(P-2;3-EPOXYPROPOXY)PHENYL)-	ACETIC ACID;(2;4;5-TRICHLOROPHENOXY)-;BUTYL ESTER
Structure	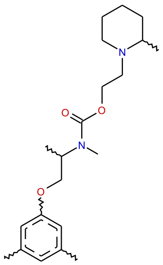	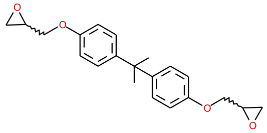	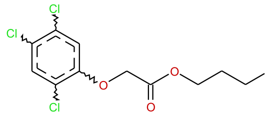
Actual Endpoint	Severe	Severe	Moderate
Predicted Endpoint	Severe	Severe	Moderate
Distance	0.575	0.658	0.663
Reference	Arzneimittel-Forschung 9;113;59	28ZPAK-;137;72	28ZPAK-;85;72

Model Prediction

Prediction: Severe

Probability: 0.845

Enrichment: 1.36

Bayesian Score: 4.92

Mahalanobis Distance: 10.4

Mahalanobis Distance p-value: 0.0147

Prediction: Positive if the Bayesian score is above the estimated best cutoff value from minimizing the false positive and false negative rate.
Probability: The esimated probability that the sample is in the positive category. This assumes that the Bayesian score follows a normal distribution and is different from the prediction using a cutoff.
Enrichment: An estimate of enrichment, that is, the increased likelihood (versus random) of this sample being in the category.
Bayesian Score: The standard Laplacian-modified Bayesian score.
Mahalanobis Distance: The Mahalanobis distance (MD) is the distance to the center of the training data. The larger the MD, the less trustworthy the prediction.
Mahalanobis Distance p-value: The p-value gives the fraction of training data with an MD greater than or equal to the one for the given sample, assuming normally distributed data. The smaller the p-value, the less trustworthy the prediciton. For highly non-normal X properties (e.g., fingerprints), the MD p-value is wildly inaccurate.

Model Applicability

Unknown features are fingerprint features in the query molecule, but not found or appearing too infrequently in the training set.

1. All properties and OPS components are within expected ranges.

Feature Contribution

Top features for positive contribution				
Fingerprint	Bit/Smiles	Feature Structure	Score	Severe in training set
SCFP_12	-1430588017	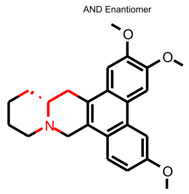 [*]CC(C[*])N([*])[*]	0.469	21 out of 21

SCFP_12	1173392318	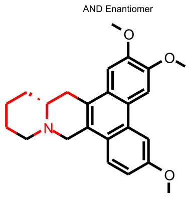 [*]C[C@H]1CCC[*]N1[*]	0.445	11 out of 11
SCFP_12	2088794301	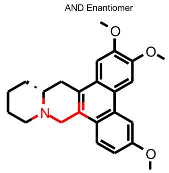 [*]N([*])C[c](:[*]):[*]	0.42	7 out of 7

Top Features for negative contribution

Fingerprint	Bit/Smiles	Feature Structure	Score	Severe in training set
SCFP_12	136239834	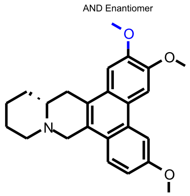 [*]OC	-0.368	14 out of 34
SCFP_12	591469355	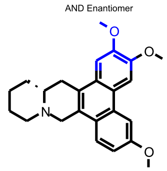 [*][c](:[*]):[c](OC): [cH]:[*]	-0.135	2 out of 4
SCFP_12	112346096	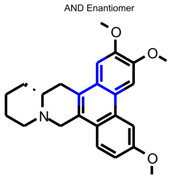 [*]:[cH]:[c]1:[c](:[*]):[*]:[*]:[c]:1:[*]	0	13 out of 21

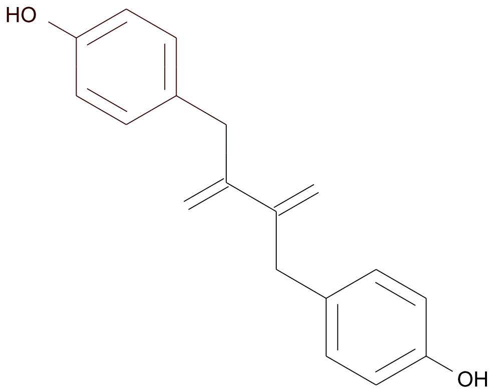

$C_{18}H_{18}O_2$
Molecular Weight: 266.33432
ALogP: 4.8
Rotatable Bonds: 5
Acceptors: 2
Donors: 2

Model Prediction

Prediction: Severe
Probability: 0.721
Enrichment: 1.16
Bayesian Score: 0.997
Mahalanobis Distance: 7.34
Mahalanobis Distance p-value: 0.971

Prediction: Positive if the Bayesian score is above the estimated best cutoff value from minimizing the false positive and false negative rate.
Probability: The esimated probability that the sample is in the positive category. This assumes that the Bayesian score follows a normal distribution and is different from the prediction using a cutoff.
Enrichment: An estimate of enrichment, that is, the increased likelihood (versus random) of this sample being in the category.
Bayesian Score: The standard Laplacian-modified Bayesian score.
Mahalanobis Distance: The Mahalanobis distance (MD) is the distance to the center of the training data. The larger the MD, the less trustworthy the prediction.
Mahalanobis Distance p-value: The p-value gives the fraction of training data with an MD greater than or equal to the one for the given sample, assuming normally distributed data. The smaller the p-value, the less trustworthy the prediciton. For highly non-normal X properties (e.g., fingerprints), the MD p-value is wildly inaccurate.

Structural Similar Compounds

Name	PHENOL;2;2'-METHYLENEBIS(4-CHLORO-	PHENOL;4;4'-ISOPROPYLIDENEDI-	RESORCINOL; 4-HEXYL-
Structure	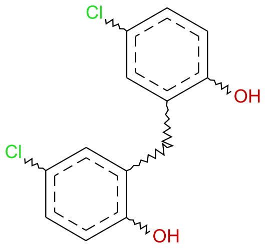	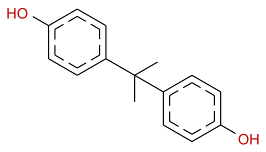	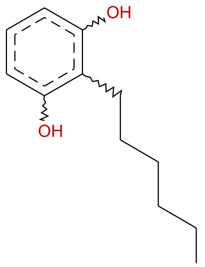
Actual Endpoint	Severe	Moderate	Severe
Predicted Endpoint	Severe	Moderate	Severe
Distance	0.509	0.517	0.584
Reference	28ZPAK-;82;72	28ZPAK-;58;72	AEPPAE 219;119;53

Model Applicability

Unknown features are fingerprint features in the query molecule, but not found or appearing too infrequently in the training set.

1. All properties and OPS components are within expected ranges.

Feature Contribution

Top features for positive contribution

Fingerprint	Bit/Smiles	Feature Structure	Score	Severe in training set
SCFP_12	611156666	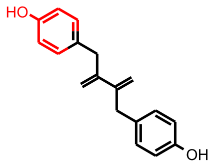 O[c]1:[cH]:[cH]:[*]:[cH]:[cH]:1	0.298	20 out of 24

SCFP_12	-1850560426	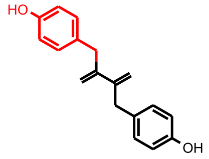 [*]C[c]1:[cH]:[cH]:[c](O):[cH]:[cH]:1	0.287	6 out of 7
SCFP_12	470101049	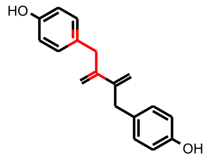 [*]C(=[*])C[c](:[*]): [*]	0.218	1 out of 1
**Top Features for negative contribution**				
**Fingerprint**	**Bit/Smiles**	**Feature Structure**	**Score**	**Severe in training set**
SCFP_12	1	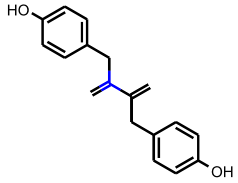 [*]C(=[*])[*]	-0.0647	188 out of 330
SCFP_12	0	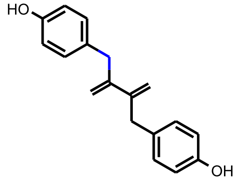 [*]C([*])[*]	0	463 out of 727
SCFP_12	3	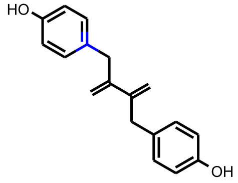 [*]:[cH]:[*]	0	162 out of 280

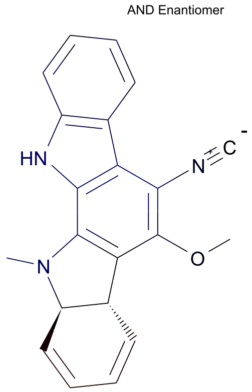

$C_{21}H_{17}N_3O$
Molecular Weight: 327.37918
ALogP: 4.078
Rotatable Bonds: 1
Acceptors: 2
Donors: 1

Model Prediction

Prediction: Moderate
Probability: 0.543
Enrichment: 0.877
Bayesian Score: -3.35
Mahalanobis Distance: 13.8
Mahalanobis Distance p-value: 4.68e-010

Prediction: Positive if the Bayesian score is above the estimated best cutoff value from minimizing the false positive and false negative rate.
Probability: The esimated probability that the sample is in the positive category. This assumes that the Bayesian score follows a normal distribution and is different from the prediction using a cutoff.
Enrichment: An estimate of enrichment, that is, the increased likelihood (versus random) of this sample being in the category.
Bayesian Score: The standard Laplacian-modified Bayesian score.
Mahalanobis Distance: The Mahalanobis distance (MD) is the distance to the center of the training data. The larger the MD, the less trustworthy the prediction.
Mahalanobis Distance p-value: The p-value gives the fraction of training data with an MD greater than or equal to the one for the given sample, assuming normally distributed data. The smaller the p-value, the less trustworthy the prediciton. For highly non-normal X properties (e.g., fingerprints), the MD p-value is wildly inaccurate.

Structural Similar Compounds

Name	BENZILIC ACID; 4;4'-DICHLORO-; ISOPROPYL ESTER	ACETIC ACID; 2-(-CHLOROMETHY-1-NAPHTHYLTHIO)-	ANTHRAQUINONE;1-(2;4;6-TRIMETHYLPHENYLAMINO)-
Structure	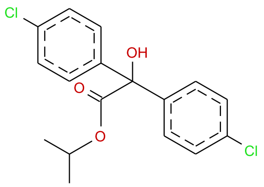	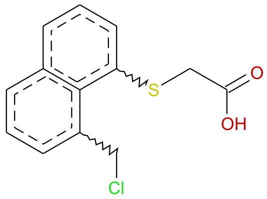	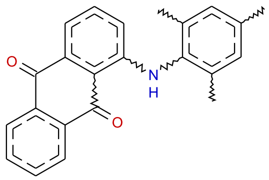
Actual Endpoint	Severe	Severe	Moderate
Predicted Endpoint	Severe	Severe	Moderate
Distance	0.637	0.668	0.691
Reference	CIGET* -;-;77	28ZPAK-;173;72	28ZPAK-;242;72

Model Applicability

Unknown features are fingerprint features in the query molecule, but not found or appearing too infrequently in the training set.

1. All properties and OPS components are within expected ranges.

Feature Contribution

Top features for positive contribution

Fingerprint	Bit/Smiles	Feature Structure	Score	Severe in training set
SCFP_12	-92192314	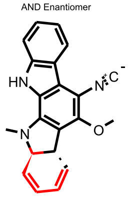 [*][C@H]1[*]C=CC=C1	0.348	3 out of 3

SCFP_12	622342378	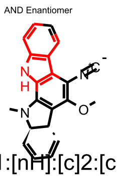 [*]:[c]1:[nH]:[c]2:[cH]:[cH]:[*]:[cH]:[c]:2:[c]:1:[*]	0.213	4 out of 5
SCFP_12	403834996	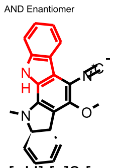 [*]:[c]1:[nH]:[c]2:[cH]:[cH]:[cH]:[cH]:[c]:2:[c]:1:[*]	0.213	4 out of 5
**Top Features for negative contribution**				
**Fingerprint**	**Bit/Smiles**	**Feature Structure**	**Score**	**Severe in training set**
SCFP_12	136418580	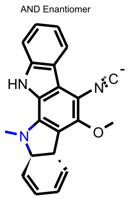 [*]N([*])C	-0.796	0 out of 2
SCFP_12	1334669481	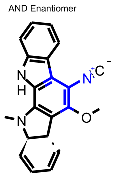 [*][c](:[*]):[c]([N+]#[*]):[c](:[*]):[*]	-0.685	28 out of 93
SCFP_12	-710590418	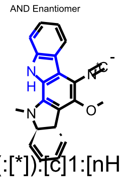 [*][c](:[*]):[c]1:[nH]:[c](:[cH]:[*]):[c](:[*]):[c]:1:[*]	-0.475	0 out of 1

AND Enantiomer

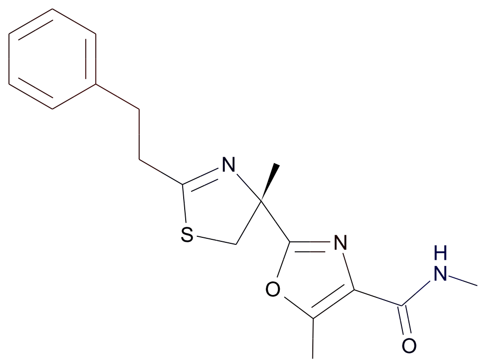

$C_{18}H_{21}N_3O_2S$
Molecular Weight: 343.44324
ALogP: 2.716
Rotatable Bonds: 5
Acceptors: 4
Donors: 1

Structural Similar Compounds

Name	ACETIC ACID; 2-(-CHLOROMETHY-1-NAPHTHYLTHIO)-	BENZOIC ACID; 5-(CHLOROSULFONYL)-2;4-DICHLORO-	Ammonium; ((N-anthraquinon-2-yl)aminomethylene)dimethyl-; chloride
Structure	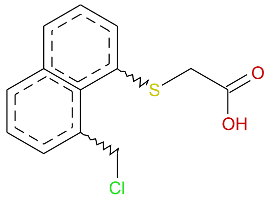	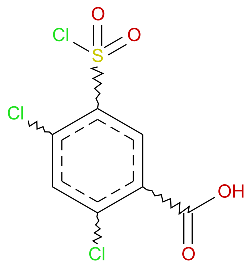	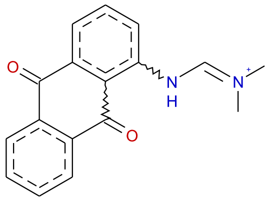
Actual Endpoint	Severe	Severe	Severe
Predicted Endpoint	Severe	Severe	Moderate
Distance	0.638	0.657	0.678
Reference	28ZPAK-;173;72	FCTOD7 20;573;82	Prehled Prumyslove Toxikologie; Organicke Latky; Marhold; J. -;732;86

Model Prediction

Prediction: Moderate
Probability: 0.675
Enrichment: 1.09
Bayesian Score: -0.475
Mahalanobis Distance: 10.4
Mahalanobis Distance p-value: 0.0114

Prediction: Positive if the Bayesian score is above the estimated best cutoff value from minimizing the false positive and false negative rate.
Probability: The esimated probability that the sample is in the positive category. This assumes that the Bayesian score follows a normal distribution and is different from the prediction using a cutoff.
Enrichment: An estimate of enrichment, that is, the increased likelihood (versus random) of this sample being in the category.
Bayesian Score: The standard Laplacian-modified Bayesian score.
Mahalanobis Distance: The Mahalanobis distance (MD) is the distance to the center of the training data. The larger the MD, the less trustworthy the prediction.
Mahalanobis Distance p-value: The p-value gives the fraction of training data with an MD greater than or equal to the one for the given sample, assuming normally distributed data. The smaller the p-value, the less trustworthy the prediciton. For highly non-normal X properties (e.g., fingerprints), the MD p-value is wildly inaccurate.

Model Applicability

Unknown features are fingerprint features in the query molecule, but not found or appearing too infrequently in the training set.

1. All properties and OPS components are within expected ranges.

Feature Contribution

Top features for positive contribution

Fingerprint	Bit/Smiles	Feature Structure	Score	Severe in training set
SCFP_12	-1640858361	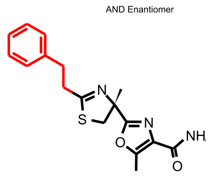 [*]CC[c]1:[cH]:[cH]:[cH]:[cH]:[cH]:1	0.376	4 out of 4

SCFP_12	-1272709286	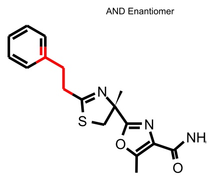 [*]C([*])C[c](:[*]):[*]	0.231	24 out of 31
SCFP_12	-2056510245	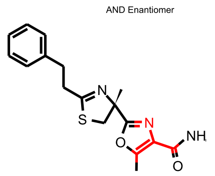 [*]C(=[*])[c]1:n:[*]:[*]:[c]:1[*]	0.218	1 out of 1

Top Features for negative contribution

Fingerprint	Bit/Smiles	Feature Structure	Score	Severe in training set
SCFP_12	6	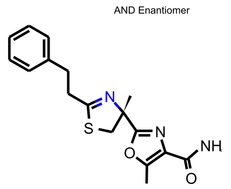 [*]N=[*]	-0.369	6 out of 15
SCFP_12	-111024397	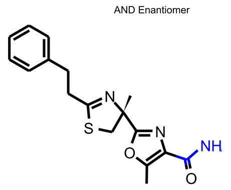 [*]C(=[*])NC	-0.345	1 out of 3
SCFP_12	1257084377	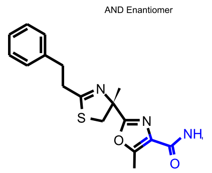 [*]NC(=O)[c](:[*]):[*]	-0.345	1 out of 3

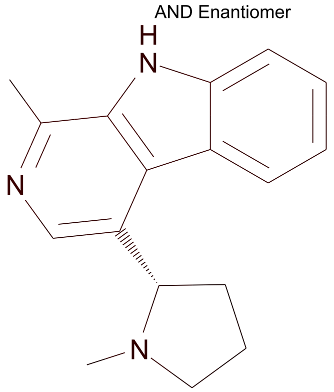

$C_{17}H_{19}N_3$
Molecular Weight: 265.35286
ALogP: 3.018
Rotatable Bonds: 1
Acceptors: 2
Donors: 1

Structural Similar Compounds

Name	3-AMINOPYRENE	p-Anisidine; N-cyclohexyl-	CARBAMIC ACID; METHYL-; 1-(5;6;7;8-TETRAHYDRO)NAPHTHYL ESTER
Structure	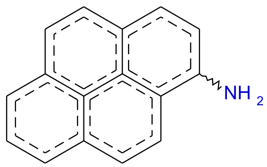	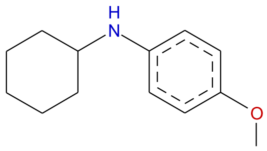	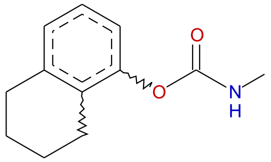
Actual Endpoint	Irritant	Irritant	Irritant
Predicted Endpoint	Non-Irritant	Irritant	Irritant
Distance	0.597	0.601	0.603
Reference	AIHAAP 30;470;69	Prehled Prumyslove Toxikologie; Organicke Latky; Marhold; J. -;723;86	28ZPAK-;163;72

Model Prediction

Prediction: Irritant
Probability: 1
Enrichment: 1.18
Bayesian Score: 3.41
Mahalanobis Distance: 4.74
Mahalanobis Distance p-value: 1

Prediction: Positive if the Bayesian score is above the estimated best cutoff value from minimizing the false positive and false negative rate.
Probability: The esimated probability that the sample is in the positive category. This assumes that the Bayesian score follows a normal distribution and is different from the prediction using a cutoff.
Enrichment: An estimate of enrichment, that is, the increased likelihood (versus random) of this sample being in the category.
Bayesian Score: The standard Laplacian-modified Bayesian score.
Mahalanobis Distance: The Mahalanobis distance (MD) is the distance to the center of the training data. The larger the MD, the less trustworthy the prediction.
Mahalanobis Distance p-value: The p-value gives the fraction of training data with an MD greater than or equal to the one for the given sample, assuming normally distributed data. The smaller the p-value, the less trustworthy the prediciton. For highly non-normal X properties (e.g., fingerprints), the MD p-value is wildly inaccurate.

Model Applicability

Unknown features are fingerprint features in the query molecule, but not found or appearing too infrequently in the training set.

1. All properties and OPS components are within expected ranges.

Feature Contribution

Top features for positive contribution

Fingerprint	Bit/Smiles	Feature Structure	Score	Irritant in training set
FCFP_12	1747237384	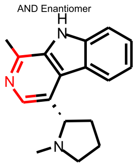 [*][c](:[*]):n:[cH]:[*]	0.208	44 out of 44

FCFP_12	136388789	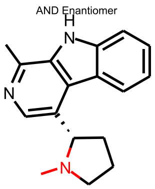 [*]N([*])C	0.206	30 out of 30
FCFP_12	-124655670	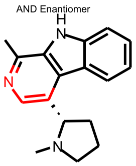 [*][c](:[*]):[cH]:n:[*]	0.2	16 out of 16
**Top Features for negative contribution**				
**Fingerprint**	**Bit/Smiles**	**Feature Structure**	**Score**	**Irritant in training set**
FCFP_12	-1272798659	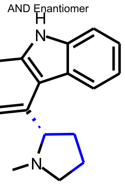 [*][C@H]1[*][*]CC1	0	517 out of 643
FCFP_12	19	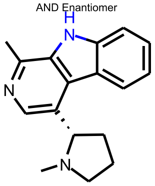 [*]:[nH]:[*]	0	5 out of 6
FCFP_12	0	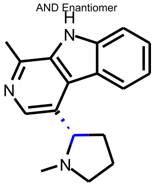 [*]C([*])[*]	0	1184 out of 1397

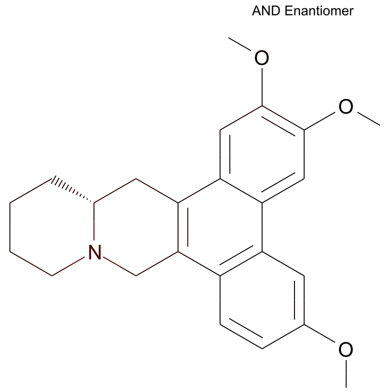

$C_{24}H_{27}NO_3$
Molecular Weight: 377.47608
ALogP: 4.691
Rotatable Bonds: 3
Acceptors: 4
Donors: 0

Structural Similar Compounds

Name	Carbamic acid; N-methyl-N-(1-(3;5-xylyloxy)-2-propyl)-;	ANTHRAQUINONE;1;5-DIPHENOXY-	PHOSPHOROUS ACID; TRIPHENYL ESTER
Structure	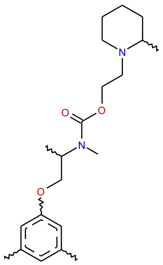	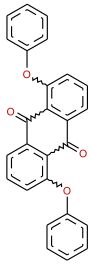	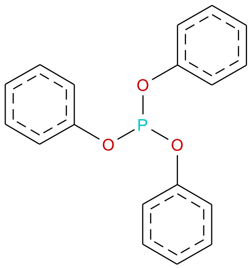
Actual Endpoint	Irritant	Non-Irritant	Irritant
Predicted Endpoint	Irritant	Non-Irritant	Irritant
Distance	0.568	0.618	0.639
Reference	Arzneimittel-Forschung 9;113;59	28ZPAK-;113;72	28ZPAK-;205;72

Model Prediction

Prediction: Irritant
Probability: 1
Enrichment: 1.18
Bayesian Score: 1.64
Mahalanobis Distance: 8.09
Mahalanobis Distance p-value: 0.906

Prediction: Positive if the Bayesian score is above the estimated best cutoff value from minimizing the false positive and false negative rate.
Probability: The esimated probability that the sample is in the positive category. This assumes that the Bayesian score follows a normal distribution and is different from the prediction using a cutoff.
Enrichment: An estimate of enrichment, that is, the increased likelihood (versus random) of this sample being in the category.
Bayesian Score: The standard Laplacian-modified Bayesian score.
Mahalanobis Distance: The Mahalanobis distance (MD) is the distance to the center of the training data. The larger the MD, the less trustworthy the prediction.
Mahalanobis Distance p-value: The p-value gives the fraction of training data with an MD greater than or equal to the one for the given sample, assuming normally distributed data. The smaller the p-value, the less trustworthy the prediciton. For highly non-normal X properties (e.g., fingerprints), the MD p-value is wildly inaccurate.

Model Applicability

Unknown features are fingerprint features in the query molecule, but not found or appearing too infrequently in the training set.

1. All properties and OPS components are within expected ranges.

Feature Contribution

Top features for positive contribution

Fingerprint	Bit/Smiles	Feature Structure	Score	Irritant in training set
FCFP_12	-1946918893	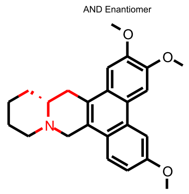 [*]CC(C[*])N([*])[*]	0.187	8 out of 8

FCFP_12	906798516	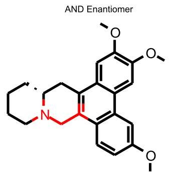 [*]N([*])C[c](:[*]):[*]	0.18	6 out of 6
FCFP_12	1848358530	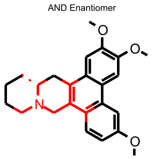 [*]CN1C[c](:[c](:[*]):[*]):[c](:[*])[*][C@H]1[*]	0.175	5 out of 5
**Top Features for negative contribution**				
**Fingerprint**	**Bit/Smiles**	**Feature Structure**	**Score**	**Irritant in training set**
FCFP_12	1175638033	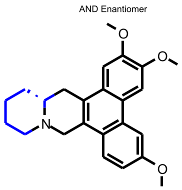 [*][C@@H]1[*]CCCC1	-0.133	207 out of 293
FCFP_12	1878037426	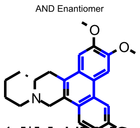 [*][c]1:[*]:[cH]:[c]2:[c]([*]):[*]:[c](:[*]):[c](:[cH]:[*]):[c]:2:[cH]:1	-0.0561	3 out of 4
FCFP_12	203677720	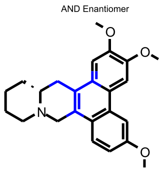 [*]C([*])[c](:[cH]:[*]):[c](:[*]):[*]	0	319 out of 382

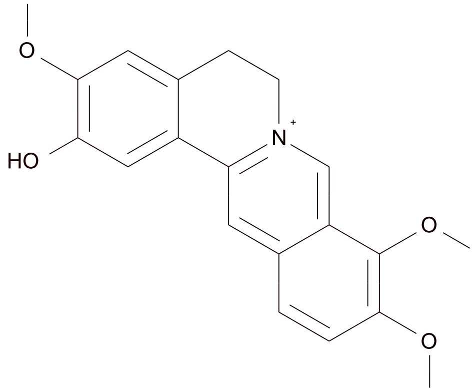

$C_{20}H_{20}NO_4$
Molecular Weight: 338.3771
ALogP: 3.936
Rotatable Bonds: 3
Acceptors: 4
Donors: 1

Model Prediction

Prediction: Irritant
Probability: 1
Enrichment: 1.18
Bayesian Score: 1.37
Mahalanobis Distance: 5.95
Mahalanobis Distance p-value: 1

Prediction: Positive if the Bayesian score is above the estimated best cutoff value from minimizing the false positive and false negative rate.
Probability: The esimated probability that the sample is in the positive category. This assumes that the Bayesian score follows a normal distribution and is different from the prediction using a cutoff.
Enrichment: An estimate of enrichment, that is, the increased likelihood (versus random) of this sample being in the category.
Bayesian Score: The standard Laplacian-modified Bayesian score.
Mahalanobis Distance: The Mahalanobis distance (MD) is the distance to the center of the training data. The larger the MD, the less trustworthy the prediction.
Mahalanobis Distance p-value: The p-value gives the fraction of training data with an MD greater than or equal to the one for the given sample, assuming normally distributed data. The smaller the p-value, the less trustworthy the prediciton. For highly non-normal X properties (e.g., fingerprints), the MD p-value is wildly inaccurate.

Structural Similar Compounds

Name	1-BENZOYLAMINO-4-METHOXY-5-CHLORANTHRAQUINONE	BENZILIC ACID; 4;4'-DICHLORO-; ISOPROPYL ESTER	BENZAMIDE; N-(5-CHLORO-1-ANTHRAQUINONYL)-
Structure	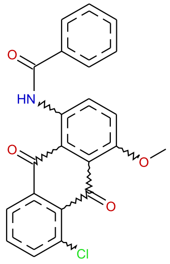	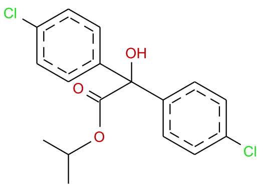	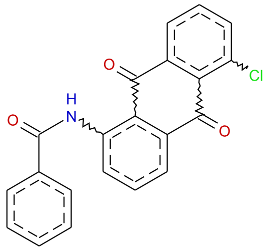
Actual Endpoint	Irritant	Irritant	Irritant
Predicted Endpoint	Irritant	Irritant	Irritant
Distance	0.583	0.602	0.604
Reference	28ZPAK-;90;72	CIGET* -;-;77	28ZPAK 89;72

Model Applicability

Unknown features are fingerprint features in the query molecule, but not found or appearing too infrequently in the training set.

1. All properties and OPS components are within expected ranges.
2. Unknown FCFP_2 feature: -150573739: [*]CC[n+](:[*]):[*]
3. Unknown FCFP_2 feature: -1861407456: [*][n+](:[*]):[c]([c](:[*]):[*]):c:[*]

Feature Contribution

Top features for positive contribution				
**Fingerprint**	**Bit/Smiles**	**Feature Structure**	**Score**	**Irritant in training set**
FCFP_12	1028934530	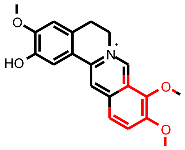 [*]O[c]1:[cH]:[*]:[c](:[*]):[cH]:[c]:1OC	0.137	2 out of 2

FCFP_12	7	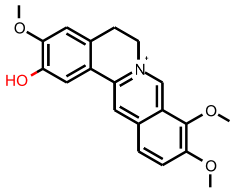<br>[*]O	0.119	142 out of 156
FCFP_12	1676877079	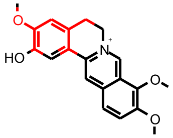<br>[*]C[c]1:[cH]:[c](O[*]):[c]([*]):[*]:[c]:1[*]	0.107	10 out of 11

Top Features for negative contribution

Fingerprint	Bit/Smiles	Feature Structure	Score	Irritant in training set
FCFP_12	203677720	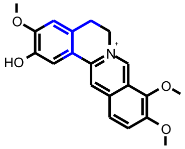<br>[*]C([*])[c](:[cH]:[*]):[c](:[*]):[*]	0	319 out of 382
FCFP_12	-1861645784	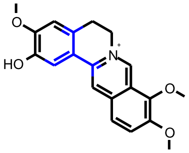<br>[*][c](:[*]):[c](:[cH]:[*])[c](:[*]):[*]	0	26 out of 31
FCFP_12	307419094	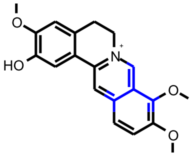<br>[*]:[cH]:[c]1:[c](:[*]):[*]:[*]:[c]:1:[*]	0	43 out of 52

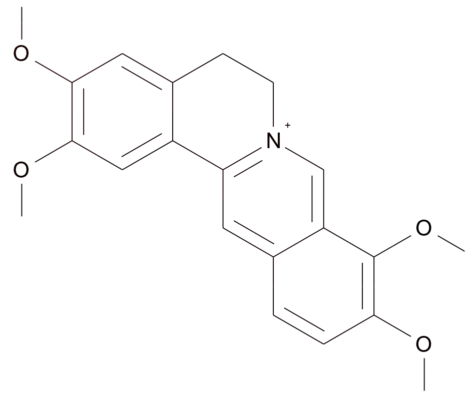

$C_{21}H_{22}NO_4$
Molecular Weight: 352.40368
ALogP: 4.161
Rotatable Bonds: 4
Acceptors: 4
Donors: 0

Model Prediction

Prediction: Irritant
Probability: 1
Enrichment: 1.18
Bayesian Score: 0.713
Mahalanobis Distance: 5.98
Mahalanobis Distance p-value: 1

Prediction: Positive if the Bayesian score is above the estimated best cutoff value from minimizing the false positive and false negative rate.
Probability: The esimated probability that the sample is in the positive category. This assumes that the Bayesian score follows a normal distribution and is different from the prediction using a cutoff.
Enrichment: An estimate of enrichment, that is, the increased likelihood (versus random) of this sample being in the category.
Bayesian Score: The standard Laplacian-modified Bayesian score.
Mahalanobis Distance: The Mahalanobis distance (MD) is the distance to the center of the training data. The larger the MD, the less trustworthy the prediction.
Mahalanobis Distance p-value: The p-value gives the fraction of training data with an MD greater than or equal to the one for the given sample, assuming normally distributed data. The smaller the p-value, the less trustworthy the prediciton. For highly non-normal X properties (e.g., fingerprints), the MD p-value is wildly inaccurate.

Structural Similar Compounds

Name	PHOSPHOROUS ACID; TRIPHENYL ESTER	PROPANE;2;2-BIS(P-2;3-EPOXYPROPOXY)PHENYL)-	Carbamic acid; N-methyl-N-(1-(3;5-xylyloxy)-2-propyl)-;
Structure	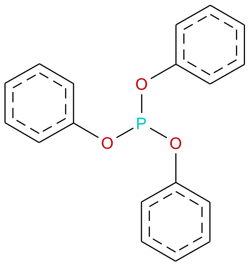	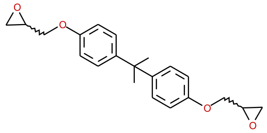	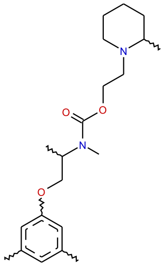
Actual Endpoint	Irritant	Irritant	Irritant
Predicted Endpoint	Irritant	Irritant	Irritant
Distance	0.573	0.583	0.586
Reference	28ZPAK-;205;72	28ZPAK-;137;72	Arzneimittel-Forschung 9;113;59

Model Applicability

Unknown features are fingerprint features in the query molecule, but not found or appearing too infrequently in the training set.

1. All properties and OPS components are within expected ranges.
2. Unknown FCFP_2 feature: -150573739: [*]CC[n+](:[*]):[*]
3. Unknown FCFP_2 feature: -1861407456: [*][n+](:[*]):[c]([c](:[*]):[*]):c:[*]

Feature Contribution

Top features for positive contribution				
**Fingerprint**	**Bit/Smiles**	**Feature Structure**	**Score**	**Irritant in training set**
FCFP_12	1028934530	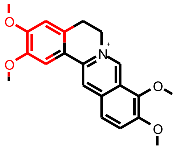 [*]O[c]1:[cH]:[*]:[c](:[*]):[cH]:[c]:1OC	0.137	2 out of 2

FCFP_12	1676877079	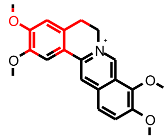 [*]C[c]1:[cH]:[c](O[*]):[c]([*]):[*]:[c]:1[*]	0.107	10 out of 11
FCFP_12	-1370111440	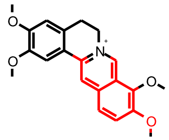 [*]O[c]1:[cH]:[cH]:[c]2:[cH]:[c]([*]):[*]:[cH]:[c]:2:[c]:1[*]	0.101	1 out of 1

Top Features for negative contribution

Fingerprint	Bit/Smiles	Feature Structure	Score	Irritant in training set
FCFP_12	-105186863	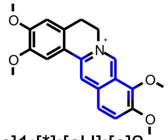 [*][c]1:[*]:[cH]:[c]2:[c]([*]):[*]:[cH]:[cH]:[c]:2:[cH]:1	0	27 out of 32
FCFP_12	-1977641857	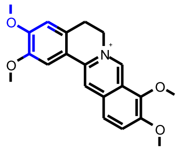 [*][c](:[*]):[c](OC):[cH]:[*]	0	15 out of 19
FCFP_12	136627117	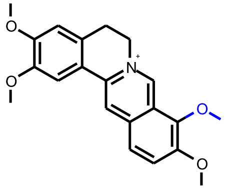 [*]OC	0	96 out of 113

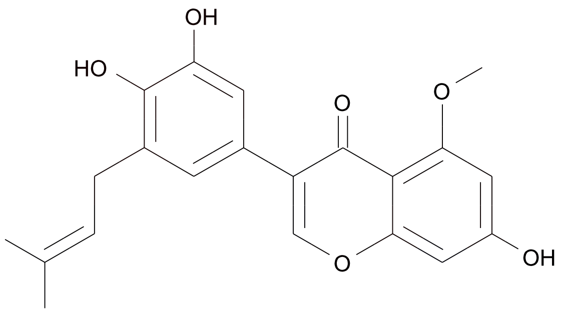

$C_{21}H_{20}O_6$
Molecular Weight: 368.3799
ALogP: 3.98
Rotatable Bonds: 4
Acceptors: 6
Donors: 3

Structural Similar Compounds

Name	Disperse Black 9	4;4'-DIAMINO-1;1'-DIANTHRIMIDE	ANTHRAQUINONE; 1-((2-HYDROXYETHYL)AMINO)-4-(METHYLAMINO)-
Structure	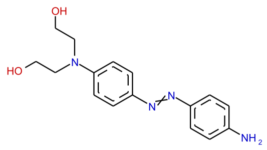	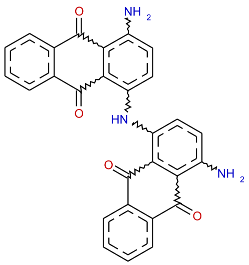	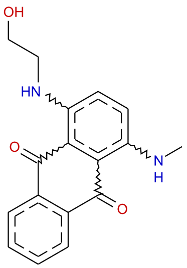
Actual Endpoint	Non-Irritant	Irritant	Irritant
Predicted Endpoint	Non-Irritant	Irritant	Irritant
Distance	0.673	0.675	0.714
Reference	J. Am. Coll. Toxicol. 5(3):205;1986	28ZPAK-;125;72	28ZPAK 245;72

Model Prediction

Prediction: Irritant
Probability: 1
Enrichment: 1.18
Bayesian Score: 0.548
Mahalanobis Distance: 12.4
Mahalanobis Distance p-value: 6.15e-006

Prediction: Positive if the Bayesian score is above the estimated best cutoff value from minimizing the false positive and false negative rate.
Probability: The esimated probability that the sample is in the positive category. This assumes that the Bayesian score follows a normal distribution and is different from the prediction using a cutoff.
Enrichment: An estimate of enrichment, that is, the increased likelihood (versus random) of this sample being in the category.
Bayesian Score: The standard Laplacian-modified Bayesian score.
Mahalanobis Distance: The Mahalanobis distance (MD) is the distance to the center of the training data. The larger the MD, the less trustworthy the prediction.
Mahalanobis Distance p-value: The p-value gives the fraction of training data with an MD greater than or equal to the one for the given sample, assuming normally distributed data. The smaller the p-value, the less trustworthy the prediciton. For highly non-normal X properties (e.g., fingerprints), the MD p-value is wildly inaccurate.

Model Applicability

Unknown features are fingerprint features in the query molecule, but not found or appearing too infrequently in the training set.

1. All properties and OPS components are within expected ranges.

Feature Contribution

Top features for positive contribution

Fingerprint	Bit/Smiles	Feature Structure	Score	Irritant in training set
FCFP_12	-1099193755	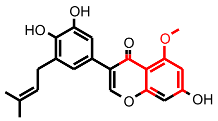 [*]C(=[*])[c]1:[c]([*]):[*]:[c]([*]):[cH]:[c]:1OC	0.175	5 out of 5

FCFP_12	-204034463	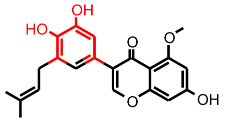 [*][c]1:[*]:[c]([*]):[c](O):[c](O):[cH]:1	0.175	5 out of 5
FCFP_12	-201608392	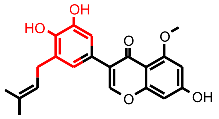 [*]C[c]1:[cH]:[*]:[cH]:[c](O):[c]:1O	0.137	2 out of 2
**Top Features for negative contribution**				
**Fingerprint**	**Bit/Smiles**	**Feature Structure**	**Score**	**Irritant in training set**
FCFP_12	1673930087	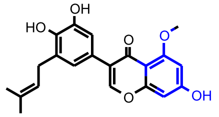 [*]O[c]1:[cH]:[c](O):[cH]:[*]:[c]:1[*]	-0.218	5 out of 8
FCFP_12	-628297815	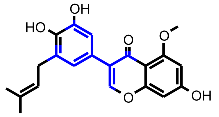 [*]\C=C(/C(=[*])[*])\[c]1:[cH]:[c]([*]):[*]:[c]([*]):[cH]:1	-0.132	2 out of 3
FCFP_12	-1582522951	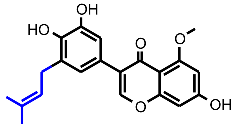 [*]CC=C(C)C	-0.0561	3 out of 4

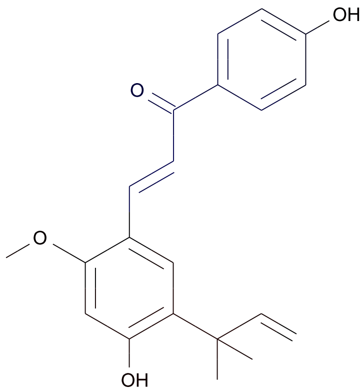

$C_{21}H_{22}O_4$
Molecular Weight: 338.39698
ALogP: 4.667
Rotatable Bonds: 6
Acceptors: 4
Donors: 2

Structural Similar Compounds

Name	2;2';-Dihydroxy-4;4'-dimethoxybenzophenone	2;2'-Dihydroxy-4-methoxybenzophenone	ANTHRAQUINONE; 1-AMINO-4-HYDROXY-2-PHENOXY-
Structure	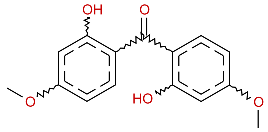	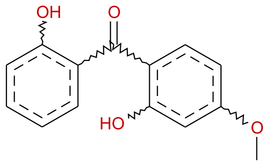	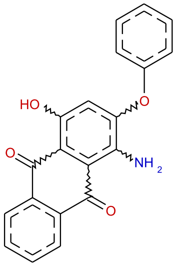
Actual Endpoint	Non-Irritant	Non-Irritant	Irritant
Predicted Endpoint	Non-Irritant	Non-Irritant	Irritant
Distance	0.671	0.684	0.686
Reference	J. Am. Coll. Toxicol. 2(5):35;1983	J. Am. Coll. Toxicol. 2(5):35;1983	28ZPAK 239;72

Model Prediction

Prediction: Non-Irritant
Probability: 0.972
Enrichment: 1.14
Bayesian Score: -0.917
Mahalanobis Distance: 8.59
Mahalanobis Distance p-value: 0.726

Prediction: Positive if the Bayesian score is above the estimated best cutoff value from minimizing the false positive and false negative rate.
Probability: The esimated probability that the sample is in the positive category. This assumes that the Bayesian score follows a normal distribution and is different from the prediction using a cutoff.
Enrichment: An estimate of enrichment, that is, the increased likelihood (versus random) of this sample being in the category.
Bayesian Score: The standard Laplacian-modified Bayesian score.
Mahalanobis Distance: The Mahalanobis distance (MD) is the distance to the center of the training data. The larger the MD, the less trustworthy the prediction.
Mahalanobis Distance p-value: The p-value gives the fraction of training data with an MD greater than or equal to the one for the given sample, assuming normally distributed data. The smaller the p-value, the less trustworthy the prediciton. For highly non-normal X properties (e.g., fingerprints), the MD p-value is wildly inaccurate.

Model Applicability

Unknown features are fingerprint features in the query molecule, but not found or appearing too infrequently in the training set.

1. All properties and OPS components are within expected ranges.

Feature Contribution

Top features for positive contribution				
**Fingerprint**	**Bit/Smiles**	**Feature Structure**	**Score**	**Irritant in training set**
FCFP_12	-836633685	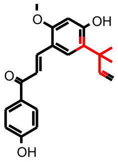 [*]=CC(C)(C)[c](:[*]) :[*]	0.192	10 out of 10

FCFP_12	-1099193755	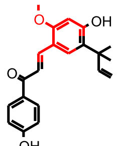<br>[*]C(=[*])[c]1:[c]([*]):[*]:[c]([*]):[cH]:[c]:1OC	0.175	5 out of 5
FCFP_12	243949920	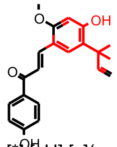<br>[*][c]1:[*]:[cH]:[c](O):[c](:[cH]:1)C(C)(C)C=[*]	0.175	5 out of 5

Top Features for negative contribution

Fingerprint	Bit/Smiles	Feature Structure	Score	Irritant in training set
FCFP_12	1244036906	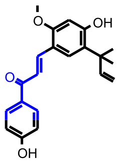<br>[*]\C=C\C(=O)[c](:[cH]:[*]):[cH]:[*]	-0.592	0 out of 1
FCFP_12	-1463030495	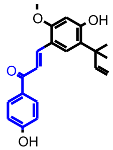<br>[*]\C=C\C(=O)[c]1:[cH]:[cH]:[c]([*]):[cH]:[cH]:1	-0.592	0 out of 1
FCFP_12	-146015125	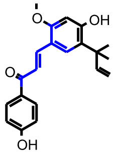<br>[*]C(=[*])\C=C\[c](:[cH]:[*]):[c]([*]):[*]	-0.268	1 out of 2

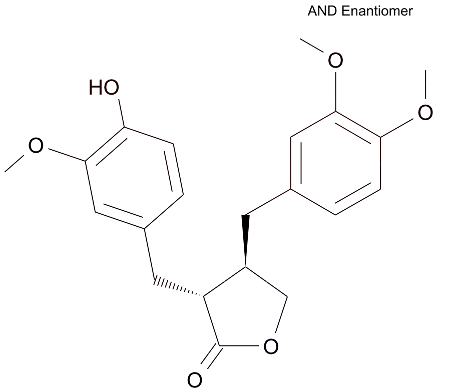

$C_{21}H_{24}O_6$

Molecular Weight: 372.41166

ALogP: 3.743

Rotatable Bonds: 7

Acceptors: 6

Donors: 1

Structural Similar Compounds

Name	COLCHICINE	1-BENZOYLAMINO-4-METHOXY-5-CHLORANTHRAQUINONE	Benzoic acid; p-(N-butyl-2-(butylamino)acetamido)-; butyl ester;
Structure	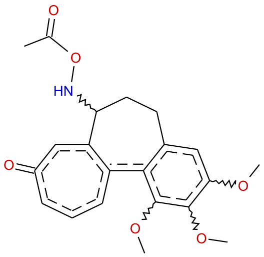	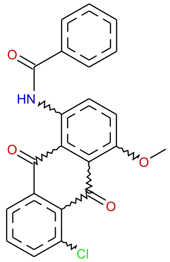	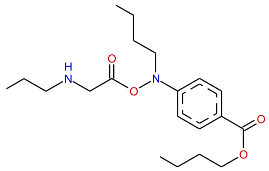
Actual Endpoint	Irritant	Irritant	Irritant
Predicted Endpoint	Irritant	Irritant	Non-Irritant
Distance	0.615	0.672	0.682
Reference	AJOPAA 31;837;48	28ZPAK-;90;72	Arzneimittel-Forschung 8;609;58

Model Prediction

Prediction: Irritant

Probability: 1

Enrichment: 1.18

Bayesian Score: 0.777

Mahalanobis Distance: 11

Mahalanobis Distance p-value: 0.00382

Prediction: Positive if the Bayesian score is above the estimated best cutoff value from minimizing the false positive and false negative rate.
Probability: The esimated probability that the sample is in the positive category. This assumes that the Bayesian score follows a normal distribution and is different from the prediction using a cutoff.
Enrichment: An estimate of enrichment, that is, the increased likelihood (versus random) of this sample being in the category.
Bayesian Score: The standard Laplacian-modified Bayesian score.
Mahalanobis Distance: The Mahalanobis distance (MD) is the distance to the center of the training data. The larger the MD, the less trustworthy the prediction.
Mahalanobis Distance p-value: The p-value gives the fraction of training data with an MD greater than or equal to the one for the given sample, assuming normally distributed data. The smaller the p-value, the less trustworthy the prediciton. For highly non-normal X properties (e.g., fingerprints), the MD p-value is wildly inaccurate.

Model Applicability

Unknown features are fingerprint features in the query molecule, but not found or appearing too infrequently in the training set.

1. All properties and OPS components are within expected ranges.

Feature Contribution

Top features for positive contribution

Fingerprint	Bit/Smiles	Feature Structure	Score	Irritant in training set
FCFP_12	-1084442457	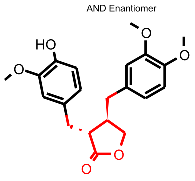 [*]C[C@H]1COC(=O)[C@@H]1C[*]	0.156	3 out of 3

FCFP_12	1028934530	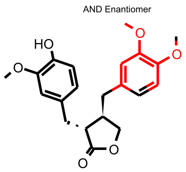 [*]O[c]1:[cH]:[*]:[c](:[*]):[cH]:[c]:1OC	0.137	2 out of 2
FCFP_12	7	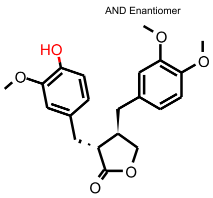 [*]O	0.119	142 out of 156

Top Features for negative contribution

Fingerprint	Bit/Smiles	Feature Structure	Score	Irritant in training set
FCFP_12	1588282714	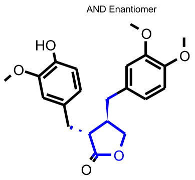 [*]C[C@H]1COC(=[*])[C@@H]1[*]	-0.401	24 out of 45
FCFP_12	-432846198	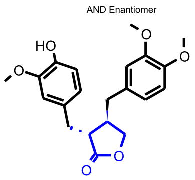 [*][C@H]1COC(=O)[C@@H]1[*]	-0.229	91 out of 142
FCFP_12	565998553	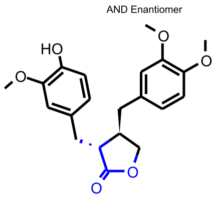 [*][C@@H]1[*][*]OC1=O	-0.0662	198 out of 262

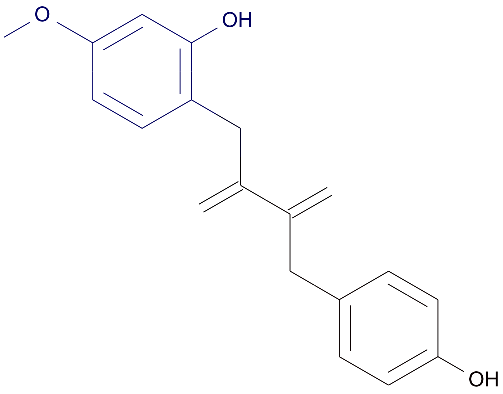

$C_{19}H_{20}O_3$
Molecular Weight: 296.3603
ALogP: 4.784
Rotatable Bonds: 6
Acceptors: 3
Donors: 2

Model Prediction

Prediction: Non-Irritant
Probability: 0.966
Enrichment: 1.14
Bayesian Score: -1.58
Mahalanobis Distance: 10.3
Mahalanobis Distance p-value: 0.0379

Prediction: Positive if the Bayesian score is above the estimated best cutoff value from minimizing the false positive and false negative rate.
Probability: The esimated probability that the sample is in the positive category. This assumes that the Bayesian score follows a normal distribution and is different from the prediction using a cutoff.
Enrichment: An estimate of enrichment, that is, the increased likelihood (versus random) of this sample being in the category.
Bayesian Score: The standard Laplacian-modified Bayesian score.
Mahalanobis Distance: The Mahalanobis distance (MD) is the distance to the center of the training data. The larger the MD, the less trustworthy the prediction.
Mahalanobis Distance p-value: The p-value gives the fraction of training data with an MD greater than or equal to the one for the given sample, assuming normally distributed data. The smaller the p-value, the less trustworthy the prediciton. For highly non-normal X properties (e.g., fingerprints), the MD p-value is wildly inaccurate.

Structural Similar Compounds

Name	PHENOL;2;2'-METHYLENEBIS(4-CHLORO-	PHENOL;4;4'-ISOPROPYLIDENEDI-	P-PHENYLENEDIAMINE; N-PHENYL-N'-CYCLOHEXYL-
Structure	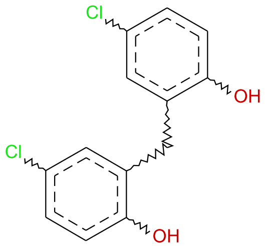	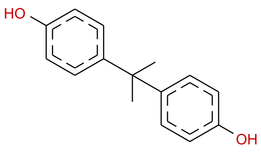	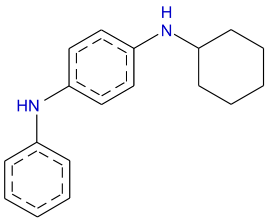
Actual Endpoint	Irritant	Irritant	Irritant
Predicted Endpoint	Irritant	Irritant	Irritant
Distance	0.587	0.614	0.634
Reference	28ZPAK-;82;72	28ZPAK-;58;72	28ZPAK-;73;72

Model Applicability

Unknown features are fingerprint features in the query molecule, but not found or appearing too infrequently in the training set.

1. All properties and OPS components are within expected ranges.

Feature Contribution

Top features for positive contribution

Fingerprint	Bit/Smiles	Feature Structure	Score	Irritant in training set
FCFP_12	7	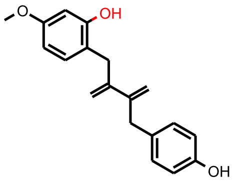 [*]O	0.119	142 out of 156

FCFP_12	1985639687	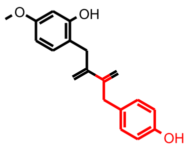 [*]C([*])C[c]1:[cH]:[cH]:[c](O):[c]([*]):[cH]:1	0.101	1 out of 1
FCFP_12	1036089772	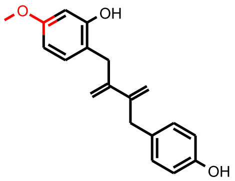 [*]:[c](:[*])OC	0.0988	75 out of 84

Top Features for negative contribution

Fingerprint	Bit/Smiles	Feature Structure	Score	Irritant in training set
FCFP_12	1800760657	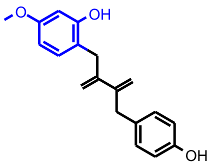 [*][c]1:[cH]:[cH]:[c](OC):[cH]:[c]:1O	-1.23	0 out of 3
FCFP_12	1863797348	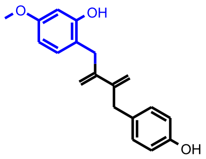 [*]C[c]1:[cH]:[cH]:[c](OC):[cH]:[c]:1O	-0.749	1 out of 4
FCFP_12	1673930087	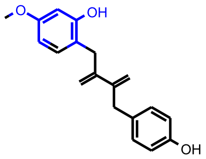 [*]O[c]1:[cH]:[c](O):[cH]:[*]:[c]:1[*]	-0.218	5 out of 8

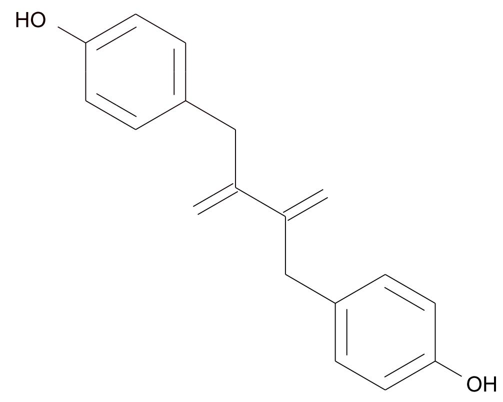

$C_{18}H_{18}O_2$
Molecular Weight: 266.33432
ALogP: 4.8
Rotatable Bonds: 5
Acceptors: 2
Donors: 2

Model Prediction

Prediction: Irritant
Probability: 1
Enrichment: 1.18
Bayesian Score: 0.417
Mahalanobis Distance: 9.16
Mahalanobis Distance p-value: 0.426

Prediction: Positive if the Bayesian score is above the estimated best cutoff value from minimizing the false positive and false negative rate.
Probability: The esimated probability that the sample is in the positive category. This assumes that the Bayesian score follows a normal distribution and is different from the prediction using a cutoff.
Enrichment: An estimate of enrichment, that is, the increased likelihood (versus random) of this sample being in the category.
Bayesian Score: The standard Laplacian-modified Bayesian score.
Mahalanobis Distance: The Mahalanobis distance (MD) is the distance to the center of the training data. The larger the MD, the less trustworthy the prediction.
Mahalanobis Distance p-value: The p-value gives the fraction of training data with an MD greater than or equal to the one for the given sample, assuming normally distributed data. The smaller the p-value, the less trustworthy the prediciton. For highly non-normal X properties (e.g., fingerprints), the MD p-value is wildly inaccurate.

Structural Similar Compounds

Name	PHENOL;4;4'-ISOPROPYLIDENEDI-	PHENOL;2;2'-METHYLENEBIS(4-CHLORO-	P-PHENYLENEDIAMINE; N;N'-DIPHENYL-
Structure	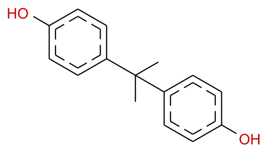	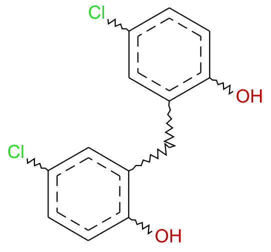	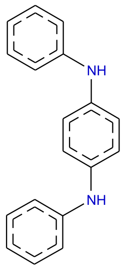
Actual Endpoint	Irritant	Irritant	Irritant
Predicted Endpoint	Irritant	Irritant	Irritant
Distance	0.499	0.505	0.556
Reference	28ZPAK-;58;72	28ZPAK-;82;72	28ZPAK-;73;72

Model Applicability

Unknown features are fingerprint features in the query molecule, but not found or appearing too infrequently in the training set.

1. All properties and OPS components are within expected ranges.

Feature Contribution

Top features for positive contribution				
**Fingerprint**	**Bit/Smiles**	**Feature Structure**	**Score**	**Irritant in training set**
FCFP_12	7	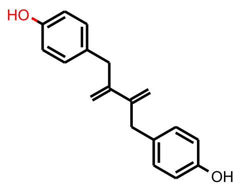 [*]O	0.119	142 out of 156

FCFP_12	1985639687	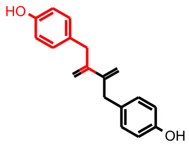 [*]C([*])C[c]1:[cH]:[cH]:[c](O):[c]([*]):[cH]:1	0.101	1 out of 1
FCFP_12	946589555	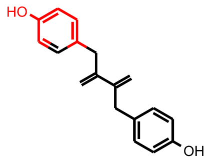 [*][c]1:[*]:[cH]:[c](O):[cH]:[cH]:1	0.096	57 out of 64

Top Features for negative contribution

Fingerprint	Bit/Smiles	Feature Structure	Score	Irritant in training set
FCFP_12	-1066794953	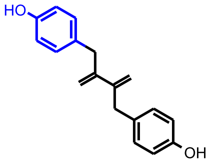 [*][c]1:[cH]:[cH]:[c](O):[cH]:[cH]:1	-0.0509	13 out of 17
FCFP_12	436886043	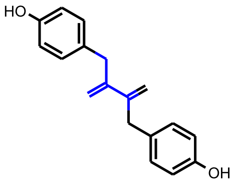 [*]C=C(C)C	0	32 out of 41
FCFP_12	203677720	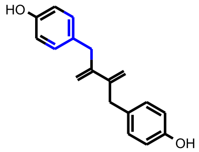 [*]C([*])[c](:[cH]:[*]):[c](:[*]):[*]	0	319 out of 382

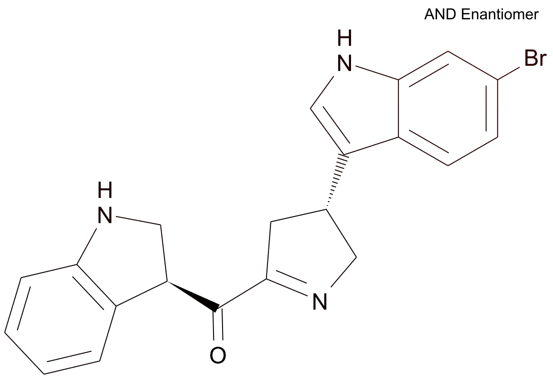

$C_{21}H_{18}BrN_3O$
Molecular Weight: 408.29112
ALogP: 3.919
Rotatable Bonds: 3
Acceptors: 3
Donors: 2

Model Prediction

Prediction: Irritant
Probability: 1
Enrichment: 1.18
Bayesian Score: 2.03
Mahalanobis Distance: 8.57
Mahalanobis Distance p-value: 0.733

Prediction: Positive if the Bayesian score is above the estimated best cutoff value from minimizing the false positive and false negative rate.
Probability: The esimated probability that the sample is in the positive category. This assumes that the Bayesian score follows a normal distribution and is different from the prediction using a cutoff.
Enrichment: An estimate of enrichment, that is, the increased likelihood (versus random) of this sample being in the category.
Bayesian Score: The standard Laplacian-modified Bayesian score.
Mahalanobis Distance: The Mahalanobis distance (MD) is the distance to the center of the training data. The larger the MD, the less trustworthy the prediction.
Mahalanobis Distance p-value: The p-value gives the fraction of training data with an MD greater than or equal to the one for the given sample, assuming normally distributed data. The smaller the p-value, the less trustworthy the prediciton. For highly non-normal X properties (e.g., fingerprints), the MD p-value is wildly inaccurate.

Structural Similar Compounds

Name	1-AMINO-4-BENZOYLAMINO-ANTHRAQUINONE	BENZAMIDE; N-(5-CHLORO-1-ANTHRAQUINONYL)-	5-NORBORNENE-2;3-DICARBOXYLIC ACID; 1;4;5;6;7;7-HEXACHLORO-
Structure	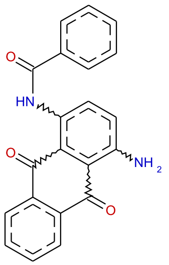	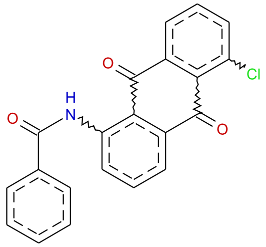	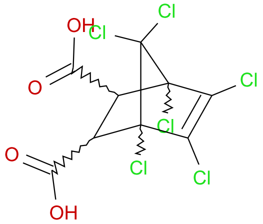
Actual Endpoint	Irritant	Irritant	Irritant
Predicted Endpoint	Irritant	Irritant	Irritant
Distance	0.679	0.683	0.683
Reference	28ZPAK-;124;72	28ZPAK 89;72	28ZPAK-;92;72

Model Applicability

Unknown features are fingerprint features in the query molecule, but not found or appearing too infrequently in the training set.

1. All properties and OPS components are within expected ranges.

Feature Contribution

Top features for positive contribution				
Fingerprint	Bit/Smiles	Feature Structure	Score	Irritant in training set
FCFP_12	-745491832	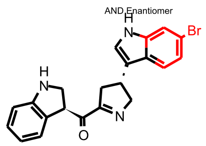 [*]:[c]1:[*]:[cH]:[cH]:[c](Br):[cH]:1	0.177	32 out of 33

FCFP_12	71476542	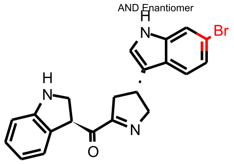 [*]:[c](:[*])Br	0.175	81 out of 84
FCFP_12	2005402822	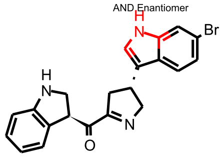 [*]:[c]1:[*]:[*]:[c](:[*]):[nH]:1	0.175	5 out of 5

Top Features for negative contribution

Fingerprint	Bit/Smiles	Feature Structure	Score	Irritant in training set
FCFP_12	1177578141	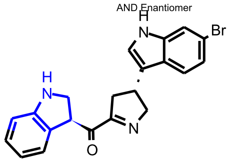 [*][C@@H]1CN[c](:[cH] :[*]):[c]1:[*]	-0.218	5 out of 8
FCFP_12	-1698724694	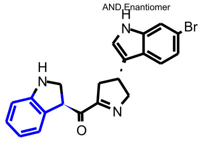 [*][C@@H]1[*][*][c]2: [cH]:[cH]:[cH]:[cH]: [c]1:2	-0.0964	107 out of 146
FCFP_12	991735244	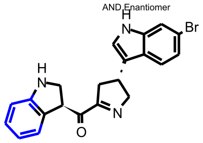 [*]:[c]1:[*]:[cH]:[cH]:[cH]:[cH]:1	0	237 out of 291

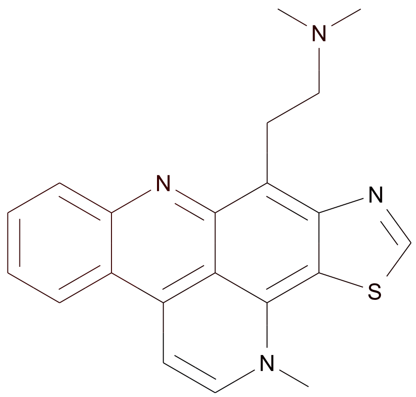

$C_{21}H_{20}N_4S$
Molecular Weight: 360.4753
ALogP: 3.682
Rotatable Bonds: 3
Acceptors: 4
Donors: 0

Model Prediction

Prediction: Irritant
Probability: 1
Enrichment: 1.18
Bayesian Score: 2.36
Mahalanobis Distance: 9.42
Mahalanobis Distance p-value: 0.291

Prediction: Positive if the Bayesian score is above the estimated best cutoff value from minimizing the false positive and false negative rate.
Probability: The esimated probability that the sample is in the positive category. This assumes that the Bayesian score follows a normal distribution and is different from the prediction using a cutoff.
Enrichment: An estimate of enrichment, that is, the increased likelihood (versus random) of this sample being in the category.
Bayesian Score: The standard Laplacian-modified Bayesian score.
Mahalanobis Distance: The Mahalanobis distance (MD) is the distance to the center of the training data. The larger the MD, the less trustworthy the prediction.
Mahalanobis Distance p-value: The p-value gives the fraction of training data with an MD greater than or equal to the one for the given sample, assuming normally distributed data. The smaller the p-value, the less trustworthy the prediciton. For highly non-normal X properties (e.g., fingerprints), the MD p-value is wildly inaccurate.

Structural Similar Compounds

Name	7H-BENZIMIDAZO(2;1-a)BENZ(de)ISOQUINOLIN-7-ONE; 10-METHOXY-	PHOSPHOROUS ACID; TRIPHENYL ESTER	PROPANE;2;2-BIS(P-2;3-EPOXYPROPOXY)PHENYL)-
Structure	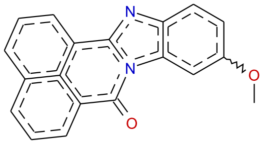	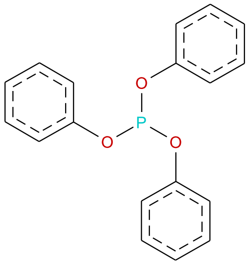	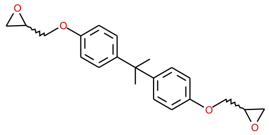
Actual Endpoint	Irritant	Irritant	Irritant
Predicted Endpoint	Irritant	Irritant	Irritant
Distance	0.601	0.608	0.623
Reference	28ZPAK 147;72	28ZPAK-;205;72	28ZPAK-;137;72

Model Applicability

Unknown features are fingerprint features in the query molecule, but not found or appearing too infrequently in the training set.

1. All properties and OPS components are within expected ranges.
2. Unknown FCFP_2 feature: -124685461: [*]1:[*]:s:c:n:1

Feature Contribution

Top features for positive contribution

Fingerprint	Bit/Smiles	Feature Structure	Score	Irritant in training set
FCFP_12	1747237384	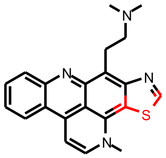 [*][c](:[*]):n:[cH]:[*]	0.208	44 out of 44

FCFP_12	136388789	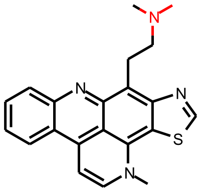 [*]N([*])C	0.206	30 out of 30
FCFP_12	178336375	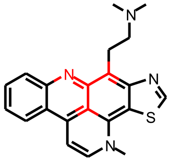 [*][c](:[*]):[c](:n:[*]):[c](:[*]):[*]	0.202	19 out of 19

Top Features for negative contribution

Fingerprint	Bit/Smiles	Feature Structure	Score	Irritant in training set
FCFP_12	451371068	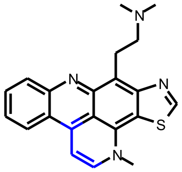 [*]\C=C\[c](:[*]):[*]	-0.167	6 out of 9
FCFP_12	0	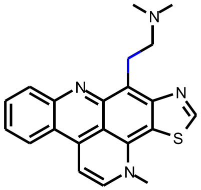 [*]C([*])[*]	0	1184 out of 1397
FCFP_12	307419094	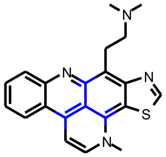 [*]:[cH]:[c]1:[c](:[*]):[*]:[*]:[c]:1:[*]	0	43 out of 52

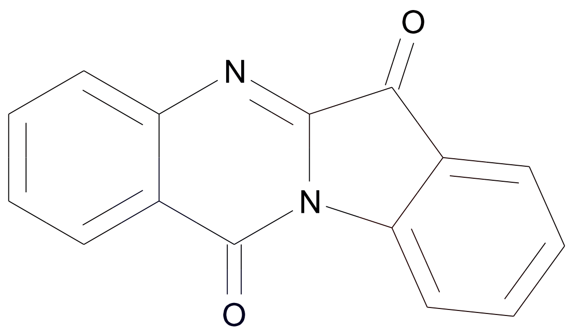

$C_{15}H_8N_2O_2$
Molecular Weight: 248.23621
ALogP: 2.331
Rotatable Bonds: 0
Acceptors: 3
Donors: 0

Structural Similar Compounds

Name	ANTHRAQUINONE;1;5-DIMETHOXY	QUINOXALINE-6-CARBONYLCHLORIDE;2;3-DICHLORO-	3;4-DIMETHYLPHENYLMALEIMIDE
Structure	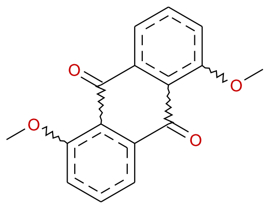	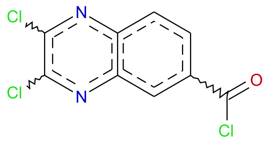	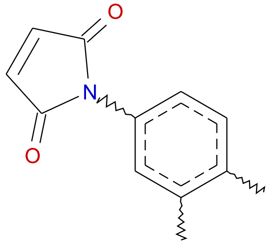
Actual Endpoint	Irritant	Irritant	Irritant
Predicted Endpoint	Irritant	Irritant	Irritant
Distance	0.553	0.554	0.561
Reference	28ZPAK-;113;72	28ZPAK-;150;72	AIHAAP 23;95;62

Model Prediction

Prediction: Irritant
Probability: 0.975
Enrichment: 1.15
Bayesian Score: -0.0737
Mahalanobis Distance: 5.66
Mahalanobis Distance p-value: 1

Prediction: Positive if the Bayesian score is above the estimated best cutoff value from minimizing the false positive and false negative rate.
Probability: The esimated probability that the sample is in the positive category. This assumes that the Bayesian score follows a normal distribution and is different from the prediction using a cutoff.
Enrichment: An estimate of enrichment, that is, the increased likelihood (versus random) of this sample being in the category.
Bayesian Score: The standard Laplacian-modified Bayesian score.
Mahalanobis Distance: The Mahalanobis distance (MD) is the distance to the center of the training data. The larger the MD, the less trustworthy the prediction.
Mahalanobis Distance p-value: The p-value gives the fraction of training data with an MD greater than or equal to the one for the given sample, assuming normally distributed data. The smaller the p-value, the less trustworthy the prediciton. For highly non-normal X properties (e.g., fingerprints), the MD p-value is wildly inaccurate.

Model Applicability

Unknown features are fingerprint features in the query molecule, but not found or appearing too infrequently in the training set.

1. All properties and OPS components are within expected ranges.

Feature Contribution

Top features for positive contribution

Fingerprint	Bit/Smiles	Feature Structure	Score	Irritant in training set
FCFP_12	159265197	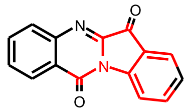 [*]C(=[*])N1C(=[*])C(=[*])[c]2:[cH]:[*]:[cH]:[cH]:[c]1:2	0.156	3 out of 3

FCFP_12	-1432259023	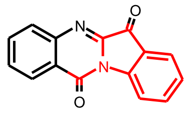 [*]C(=[*])N1C(=[*])C(=[*])[c]2:[cH]:[cH]:[cH]:[cH]:[c]1:2	0.137	2 out of 2
FCFP_12	1150094517	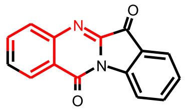 [*]C1=N[c]2:[cH]:[cH]:[*]:[cH]:[c]:2C(=[*])[*]1	0.101	1 out of 1

Top Features for negative contribution

Fingerprint	Bit/Smiles	Feature Structure	Score	Irritant in training set
FCFP_12	-1549163031	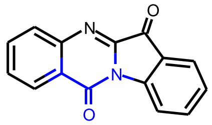 [*]N([*])C(=O)[c](:[*]):[*]	-0.623	16 out of 38
FCFP_12	580453787	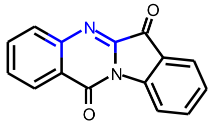 [*]C(=N[c](:[*]):[*])[*]	-0.132	2 out of 3
FCFP_12	-1698724694	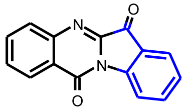 [*][C@@H]1[*][*][c]2:[cH]:[cH]:[cH]:[cH]:[c]1:2	-0.0964	107 out of 146

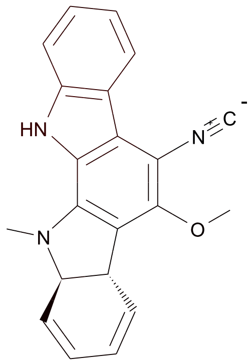

$C_{21}H_{17}N_3O$

Molecular Weight: 327.37918

ALogP: 4.078

Rotatable Bonds: 1

Acceptors: 2

Donors: 1

Structural Similar Compounds

Name	Anthraquinone; 1-bromo-4-(methylamino)-	BENZAMIDE; N-(5-CHLORO-1-ANTHRAQUINONYL)-	N;S-DIBENZOYL-O-AMINOTHIOPHENOL
Structure	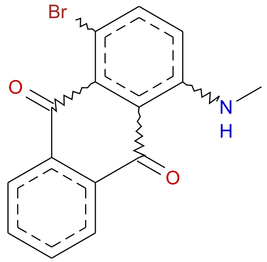	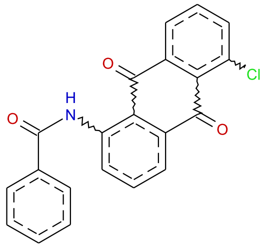	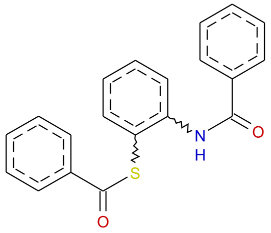
Actual Endpoint	Irritant	Irritant	Irritant
Predicted Endpoint	Irritant	Irritant	Irritant
Distance	0.587	0.592	0.627
Reference	Prehled Prumyslove Toxikologie; Organicke Latky; Marhold; J. pp 566;86	28ZPAK 89;72	28ZPAK-;175;72

Model Prediction

Prediction: Irritant

Probability: 1

Enrichment: 1.18

Bayesian Score: 1.74

Mahalanobis Distance: 9.16

Mahalanobis Distance p-value: 0.425

Prediction: Positive if the Bayesian score is above the estimated best cutoff value from minimizing the false positive and false negative rate.
Probability: The esimated probability that the sample is in the positive category. This assumes that the Bayesian score follows a normal distribution and is different from the prediction using a cutoff.
Enrichment: An estimate of enrichment, that is, the increased likelihood (versus random) of this sample being in the category.
Bayesian Score: The standard Laplacian-modified Bayesian score.
Mahalanobis Distance: The Mahalanobis distance (MD) is the distance to the center of the training data. The larger the MD, the less trustworthy the prediction.
Mahalanobis Distance p-value: The p-value gives the fraction of training data with an MD greater than or equal to the one for the given sample, assuming normally distributed data. The smaller the p-value, the less trustworthy the prediciton. For highly non-normal X properties (e.g., fingerprints), the MD p-value is wildly inaccurate.

Model Applicability

Unknown features are fingerprint features in the query molecule, but not found or appearing too infrequently in the training set.

1. All properties and OPS components are within expected ranges.
2. Unknown FCFP_2 feature: 4: [*]#[C-]
3. Unknown FCFP_2 feature: -828984032: [*][c](:[*]):[c]([N+]#[*]):[c](:[*]):[*]
4. Unknown FCFP_2 feature: 1934974835: [*]:[c](:[*])[N+]#[C-]
5. Unknown FCFP_2 feature: -1487147388: [*][N+]#[C-]

Feature Contribution

Top features for positive contribution				
**Fingerprint**	**Bit/Smiles**	**Feature Structure**	**Score**	**Irritant in training set**

FCFP_12	2005402822	AND Enantiomer 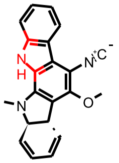 [*]:[c]1:[*]:[*]:[c](:[*]):[nH]:1	0.175	5 out of 5
FCFP_12	-525166915	AND Enantiomer 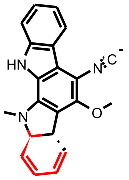 [*][C@H]1[*]C=CC=C1	0.167	4 out of 4
FCFP_12	-1192617147	AND Enantiomer 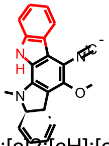 [*]1:[*]:[c]2:[cH]:[cH]:[cH]:[cH]:[c]:2:[nH]:1	0.156	3 out of 3

Top Features for negative contribution

Fingerprint	Bit/Smiles	Feature Structure	Score	Irritant in training set
FCFP_12	8	AND Enantiomer 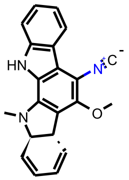 [*][N+]#[*]	-0.0561	3 out of 4
FCFP_12	0	AND Enantiomer 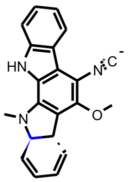 [*]C([*])[*]	0	1184 out of 1397

FCFP_12	307419094	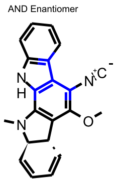 [*]:[cH]:[c]1:[c](:[*]):[*]:[*]:[c]:1:[*]	0	43 out of 52

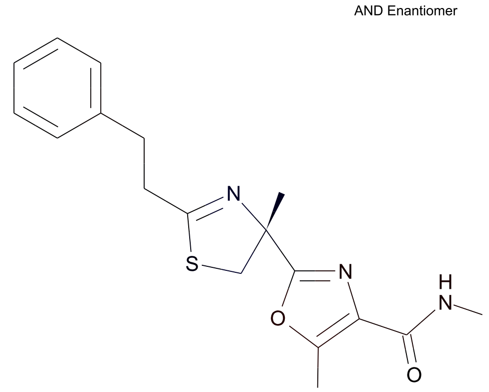

$C_{18}H_{21}N_3O_2S$
Molecular Weight: 343.44324
ALogP: 2.716
Rotatable Bonds: 5
Acceptors: 4
Donors: 1

Structural Similar Compounds

Name	ACETIC ACID; 2-(-CHLOROMETHY-1-NAPHTHYLTHIO)-	Anthraquinone; 1-bromo-4-(methylamino)-	1-BENZOYLAMINO-4-METHOXY-5-CHLORANTHRAQUINONE
Structure	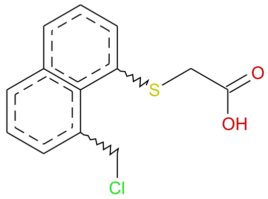	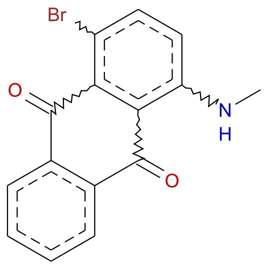	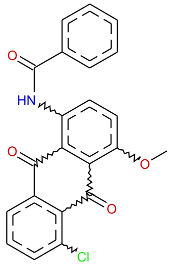
Actual Endpoint	Irritant	Irritant	Irritant
Predicted Endpoint	Irritant	Irritant	Irritant
Distance	0.629	0.639	0.641
Reference	28ZPAK-;173;72	Prehled Prumyslove Toxikologie; Organicke Latky; Marhold; J. pp 566;86	28ZPAK-;90;72

Model Prediction

Prediction: Irritant
Probability: 1
Enrichment: 1.18
Bayesian Score: 0.693
Mahalanobis Distance: 9.32
Mahalanobis Distance p-value: 0.341

Prediction: Positive if the Bayesian score is above the estimated best cutoff value from minimizing the false positive and false negative rate.
Probability: The esimated probability that the sample is in the positive category. This assumes that the Bayesian score follows a normal distribution and is different from the prediction using a cutoff.
Enrichment: An estimate of enrichment, that is, the increased likelihood (versus random) of this sample being in the category.
Bayesian Score: The standard Laplacian-modified Bayesian score.
Mahalanobis Distance: The Mahalanobis distance (MD) is the distance to the center of the training data. The larger the MD, the less trustworthy the prediction.
Mahalanobis Distance p-value: The p-value gives the fraction of training data with an MD greater than or equal to the one for the given sample, assuming normally distributed data. The smaller the p-value, the less trustworthy the prediciton. For highly non-normal X properties (e.g., fingerprints), the MD p-value is wildly inaccurate.

Model Applicability

Unknown features are fingerprint features in the query molecule, but not found or appearing too infrequently in the training set.

1. All properties and OPS components are within expected ranges.

Feature Contribution

Top features for positive contribution				
**Fingerprint**	**Bit/Smiles**	**Feature Structure**	**Score**	**Irritant in training set**
FCFP_12	1747237384	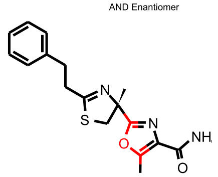 [*][c](:[*]):n:[cH]:[*]	0.208	44 out of 44

FCFP_12	-1539132615	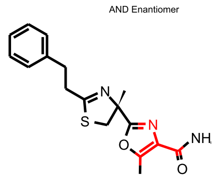 [*]:n:[c](C):[c](:[*]<br>):[*]	0.197	13 out of 13
FCFP_12	17	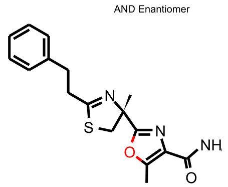 [*]:n:[*]	0.189	48 out of 49
**Top Features for negative contribution**				
**Fingerprint**	**Bit/Smiles**	**Feature Structure**	**Score**	**Irritant in training set**
FCFP_12	-836603894	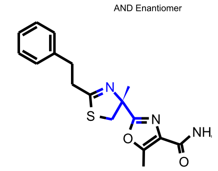 [*]:[c](:[*])[C@]1(C)<br>C[*][*]=N1	-0.592	0 out of 1
FCFP_12	-432846198	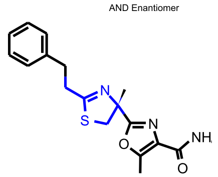 [*][C@H]1COC(=O)[C@@H<br>]1[*]	-0.229	91 out of 142
FCFP_12	1981711554	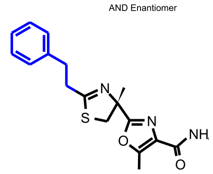 [*]CC[c]1:[cH]:[cH]:[<br>cH]:[cH]:[cH]:1	-0.103	5 out of 7

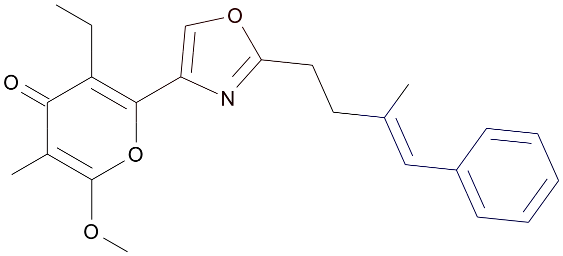

$C_{23}H_{25}NO_4$
Molecular Weight: 379.4489
ALogP: 5.22
Rotatable Bonds: 7
Acceptors: 4
Donors: 0

Model Prediction

Prediction: Non-Irritant
Probability: 0.97
Enrichment: 1.14
Bayesian Score: -1.11
Mahalanobis Distance: 9.1
Mahalanobis Distance p-value: 0.455

Prediction: Positive if the Bayesian score is above the estimated best cutoff value from minimizing the false positive and false negative rate.
Probability: The esimated probability that the sample is in the positive category. This assumes that the Bayesian score follows a normal distribution and is different from the prediction using a cutoff.
Enrichment: An estimate of enrichment, that is, the increased likelihood (versus random) of this sample being in the category.
Bayesian Score: The standard Laplacian-modified Bayesian score.
Mahalanobis Distance: The Mahalanobis distance (MD) is the distance to the center of the training data. The larger the MD, the less trustworthy the prediction.
Mahalanobis Distance p-value: The p-value gives the fraction of training data with an MD greater than or equal to the one for the given sample, assuming normally distributed data. The smaller the p-value, the less trustworthy the prediciton. For highly non-normal X properties (e.g., fingerprints), the MD p-value is wildly inaccurate.

Structural Similar Compounds

Name	Carbamic acid; N-methyl-N-(1-(3;5-xylyloxy)-2-propyl)-;	ANTHRAQUINONE;1;5-DIPHENOXY-	Butyl Benzyl Phthalate
Structure	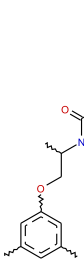	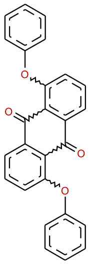	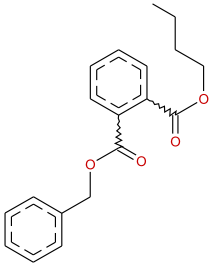
Actual Endpoint	Irritant	Non-Irritant	Non-Irritant
Predicted Endpoint	Irritant	Non-Irritant	Non-Irritant
Distance	0.565	0.568	0.593
Reference	Arzneimittel-Forschung 9;113;59	28ZPAK-;113;72	J. Am. Coll. Toxicol. 11(1):1;1992

Model Applicability

Unknown features are fingerprint features in the query molecule, but not found or appearing too infrequently in the training set.

1. All properties and OPS components are within expected ranges.
2. Unknown FCFP_2 feature: -1678245750: [*]OC(=C([*])[*])[c](:[*]):[*]
3. Unknown FCFP_2 feature: -2115241127: [*]OC(=C([*])[*])O[*]

Feature Contribution

Top features for positive contribution

Fingerprint	Bit/Smiles	Feature Structure	Score	Irritant in training set
FCFP_12	1747237384	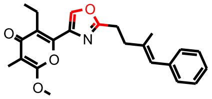 [*][c](:[*]):n:[cH]:[*]	0.208	44 out of 44

FCFP_12	-124655670	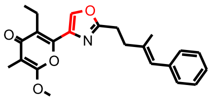 [*][c](:[*]):[cH]:n:[*]	0.2	16 out of 16
FCFP_12	-1539132615	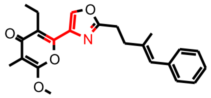 [*]:n:[c](C):[c](:[*]):[*]	0.197	13 out of 13

Top Features for negative contribution

**Fingerprint**	**Bit/Smiles**	**Feature Structure**	**Score**	**Irritant in training set**
FCFP_12	-751355393	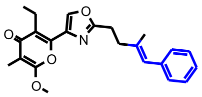 [*]C(=C[c]1:[cH]:[cH]:[cH]:[cH]:[cH]:1)[*]	-0.961	0 out of 2
FCFP_12	-1078052987	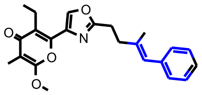 [*]C(=C[c]1:[cH]:[cH]:[*]:[cH]:[cH]:1)[*]	-0.344	2 out of 4
FCFP_12	451371068	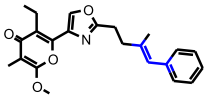 [*]\C=C\[c](:[*]):[*]	-0.167	6 out of 9

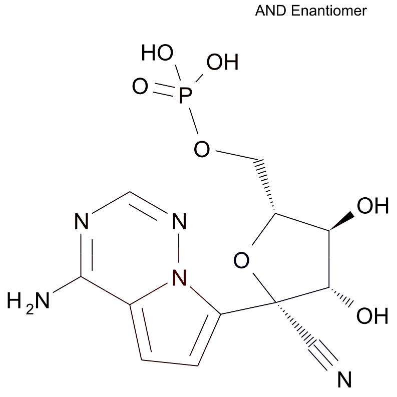

$C_{12}H_{14}N_5O_7P$
Molecular Weight: 371.24262
ALogP: -1.502
Rotatable Bonds: 4
Acceptors: 11
Donors: 5

Structural Similar Compounds

Name	1;3;6-NAPHTHALENE TRISULFONIC ACID;7-AMINO-	Methanol; (s-triazine-2;4;6-triyltrinitrilo)hexa-	2;2'-Biphenyldisulfonic acid; 4;4'-diamino-
Structure	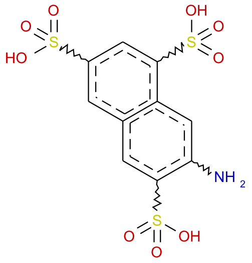	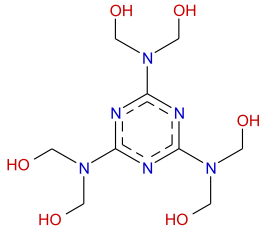	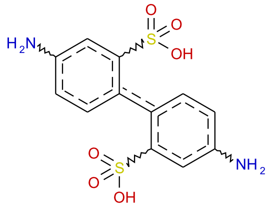
Actual Endpoint	Irritant	Irritant	Irritant
Predicted Endpoint	Irritant	Irritant	Irritant
Distance	0.766	0.795	0.859
Reference	28ZPAK-;190;72	Prehled Prumyslove Toxikologie; Organicke Latky; Marhold; J. -;876;86	Prehled Prumyslove Toxikologie; Organicke Latky; Marhold; J. pp 1061;86

Model Prediction

Prediction: Irritant
Probability: 1
Enrichment: 1.18
Bayesian Score: 1.33
Mahalanobis Distance: 10.7
Mahalanobis Distance p-value: 0.0147

Prediction: Positive if the Bayesian score is above the estimated best cutoff value from minimizing the false positive and false negative rate.
Probability: The esimated probability that the sample is in the positive category. This assumes that the Bayesian score follows a normal distribution and is different from the prediction using a cutoff.
Enrichment: An estimate of enrichment, that is, the increased likelihood (versus random) of this sample being in the category.
Bayesian Score: The standard Laplacian-modified Bayesian score.
Mahalanobis Distance: The Mahalanobis distance (MD) is the distance to the center of the training data. The larger the MD, the less trustworthy the prediction.
Mahalanobis Distance p-value: The p-value gives the fraction of training data with an MD greater than or equal to the one for the given sample, assuming normally distributed data. The smaller the p-value, the less trustworthy the prediciton. For highly non-normal X properties (e.g., fingerprints), the MD p-value is wildly inaccurate.

Model Applicability

Unknown features are fingerprint features in the query molecule, but not found or appearing too infrequently in the training set.

1. All properties and OPS components are within expected ranges.
2. Unknown FCFP_2 feature: 472180098: [*]OP(=O)(O)O
3. Unknown FCFP_2 feature: -124685461: [*]1:[*]:s:c:n:1
4. Unknown FCFP_2 feature: -1151884458: [*]:n:[c](N):[c](:[*]):[*]

Feature Contribution

Top features for positive contribution				
Fingerprint	Bit/Smiles	Feature Structure	Score	Irritant in training set

FCFP_12	1747237384	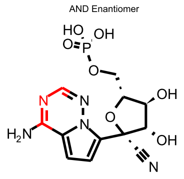<br>[*][c](:[*]):n:[cH]:[*]	0.208	44 out of 44
FCFP_12	178336375	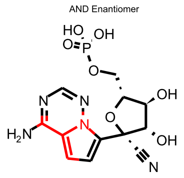<br>[*][c](:[*]):[c](:n:[*]):[c](:[*]):[*]	0.202	19 out of 19
FCFP_12	713358128	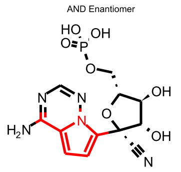<br>[*]:n:[c]1:[cH]:[cH]:[cH]:[*]:[c]:1:[*]	0.2	17 out of 17

Top Features for negative contribution

Fingerprint	Bit/Smiles	Feature Structure	Score	Irritant in training set
FCFP_12	-836603894	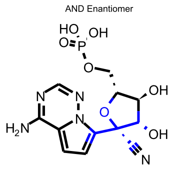<br>[*]:[c](:[*])[C@]1(C)C[*][*]=N1	-0.592	0 out of 1
FCFP_12	-1277879912	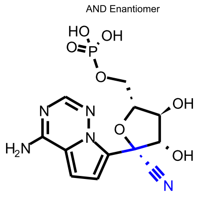<br>[*]C([*])([*])C#N	-0.0939	33 out of 45

FCFP_12	1872154524	AND Enantiomer 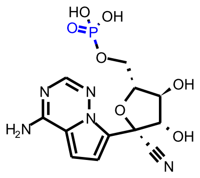 [*]C(=O)[*]	0	563 out of 690

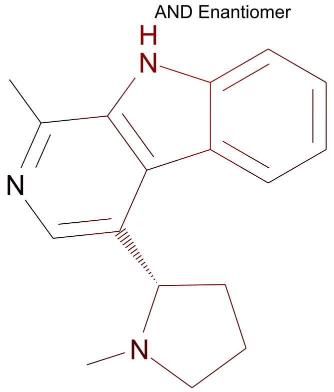

$C_{17}H_{19}N_3$
Molecular Weight: 265.35286
ALogP: 3.018
Rotatable Bonds: 1
Acceptors: 2
Donors: 1

Structural Similar Compounds

Name	Granisetron	Temazepam	Diazepam
Structure	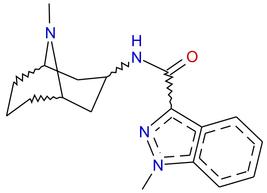	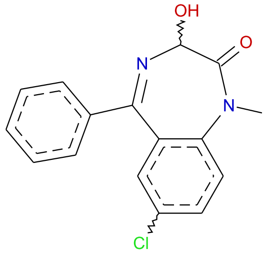	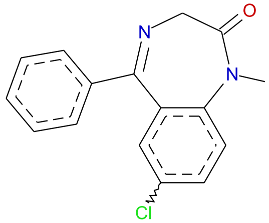
Actual Endpoint	Carcinogen	Non-Carcinogen	Non-Carcinogen
Predicted Endpoint	Carcinogen	Non-Carcinogen	Non-Carcinogen
Distance	0.595	0.598	0.606
Reference	US FDA (Centre for Drug Eval.& Res./Off. Testing & Res.) Sept. 1997	US FDA (Centre for Drug Eval.& Res./Off. Testing & Res.) Sept. 1997	US FDA (Centre for Drug Eval.& Res./Off. Testing & Res.) Sept. 1997

Model Prediction

Prediction: Carcinogen
Probability: 0.428
Enrichment: 1.33
Bayesian Score: 5.03
Mahalanobis Distance: 11.1
Mahalanobis Distance p-value: 0.0593

Prediction: Positive if the Bayesian score is above the estimated best cutoff value from minimizing the false positive and false negative rate.
Probability: The esimated probability that the sample is in the positive category. This assumes that the Bayesian score follows a normal distribution and is different from the prediction using a cutoff.
Enrichment: An estimate of enrichment, that is, the increased likelihood (versus random) of this sample being in the category.
Bayesian Score: The standard Laplacian-modified Bayesian score.
Mahalanobis Distance: The Mahalanobis distance (MD) is the distance to the center of the training data. The larger the MD, the less trustworthy the prediction.
Mahalanobis Distance p-value: The p-value gives the fraction of training data with an MD greater than or equal to the one for the given sample, assuming normally distributed data. The smaller the p-value, the less trustworthy the prediciton. For highly non-normal X properties (e.g., fingerprints), the MD p-value is wildly inaccurate.

Model Applicability

Unknown features are fingerprint features in the query molecule, but not found or appearing too infrequently in the training set.

1. All properties and OPS components are within expected ranges.

Feature Contribution

Top features for positive contribution				
**Fingerprint**	**Bit/Smiles**	**Feature Structure**	**Score**	**Carcinogen in training set**
ECFP_12	558201926	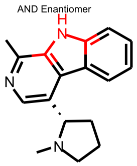 [*]:[c]1:[*]:[*]:[c](:[*]):[nH]:1	0.539	5 out of 8

ECFP_12	1099224616	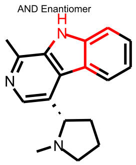 [*]:[cH]:[c]1:[nH]:[*]:[*]:[c]:1:[*]	0.456	6 out of 11
ECFP_12	1639827160	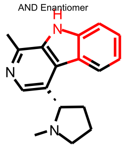 [*]1:[*]:[c]2:[*]:[cH]:[cH]:[cH]:[c]:2:[n H]:1	0.45	4 out of 7
**Top Features for negative contribution**				
**Fingerprint**	**Bit/Smiles**	**Feature Structure**	**Score**	**Carcinogen in training set**
ECFP_12	1997021792	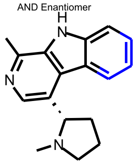 [*]:[cH]:[cH]:[cH]:[*]	-0.296	36 out of 156
ECFP_12	866343404	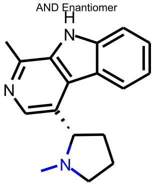 [*]N([*])C	-0.281	4 out of 18
ECFP_12	978230116	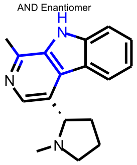 [*][c](:[*]):[c]1:[nH]:[*]:[*]:[c]:1:[*]	-0.272	0 out of 1

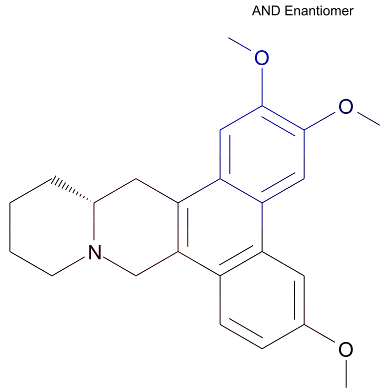

$C_{24}H_{27}NO_3$
Molecular Weight: 377.47608
ALogP: 4.691
Rotatable Bonds: 3
Acceptors: 4
Donors: 0

Model Prediction

Prediction: Carcinogen
Probability: 0.274
Enrichment: 0.85
Bayesian Score: -1.31
Mahalanobis Distance: 10.8
Mahalanobis Distance p-value: 0.108

Prediction: Positive if the Bayesian score is above the estimated best cutoff value from minimizing the false positive and false negative rate.
Probability: The esimated probability that the sample is in the positive category. This assumes that the Bayesian score follows a normal distribution and is different from the prediction using a cutoff.
Enrichment: An estimate of enrichment, that is, the increased likelihood (versus random) of this sample being in the category.
Bayesian Score: The standard Laplacian-modified Bayesian score.
Mahalanobis Distance: The Mahalanobis distance (MD) is the distance to the center of the training data. The larger the MD, the less trustworthy the prediction.
Mahalanobis Distance p-value: The p-value gives the fraction of training data with an MD greater than or equal to the one for the given sample, assuming normally distributed data. The smaller the p-value, the less trustworthy the prediciton. For highly non-normal X properties (e.g., fingerprints), the MD p-value is wildly inaccurate.

Structural Similar Compounds

Name	Ethynodiol	Loratidine	Chlorpromazine
Structure	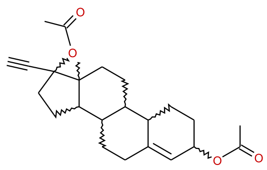	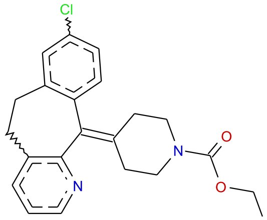	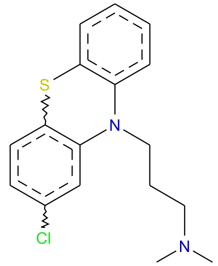
Actual Endpoint	Carcinogen	Non-Carcinogen	Non-Carcinogen
Predicted Endpoint	Carcinogen	Non-Carcinogen	Non-Carcinogen
Distance	0.576	0.594	0.632
Reference	US FDA (Centre for Drug Eval.& Res./Off. Testing & Res.) Sept. 1997	US FDA (Centre for Drug Eval.& Res./Off. Testing & Res.) Sept. 1997	US FDA (Centre for Drug Eval.& Res./Off. Testing & Res.) Sept. 1997

Model Applicability

Unknown features are fingerprint features in the query molecule, but not found or appearing too infrequently in the training set.

1. All properties and OPS components are within expected ranges.

Feature Contribution

Top features for positive contribution

Fingerprint	Bit/Smiles	Feature Structure	Score	Carcinogen in training set
ECFP_12	-1103661462	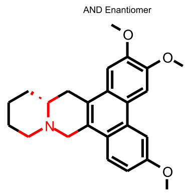 [*]CN(C[*])C([*])[*]	0.553	4 out of 6

ECFP_12	53207596	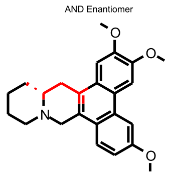 [*]C([*])C[c](:[*]):[*]	0.459	8 out of 15
ECFP_12	2082767335	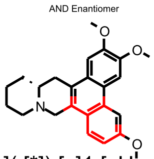 [*][c](:[*]):[c]1:[cH]:[cH]:[c]([*]):[*]:[c]:1:[*]	0.437	2 out of 3

Top Features for negative contribution

Fingerprint	Bit/Smiles	Feature Structure	Score	Carcinogen in training set
ECFP_12	-468366781	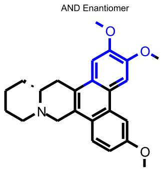 [*]O[c]1:[cH]:[*]:[c](:[*]):[cH]:[c]:1OC	-0.941	0 out of 5
ECFP_12	-2063600634	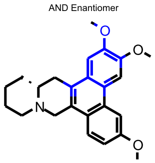 [*]O[c]1:[cH]:[c](:[c]([*]):[*]):[c](:[*]):[*]:[c]:1[*]	-0.661	0 out of 3
ECFP_12	-665049291	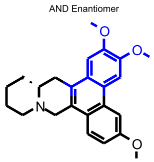 [*][c](:[*]):[c]1:[cH]:[c](OC):[c](OC):[cH]:[c]:1:[*]	-0.661	0 out of 3

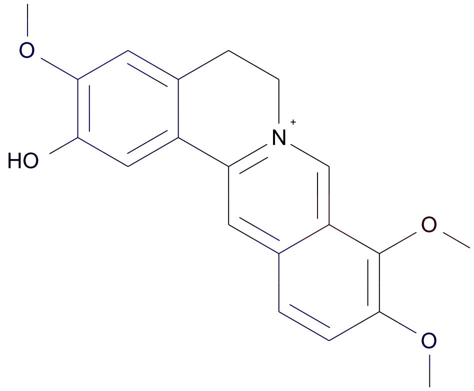

$C_{20}H_{20}NO_4$
Molecular Weight: 338.3771
ALogP: 3.936
Rotatable Bonds: 3
Acceptors: 4
Donors: 1

Model Prediction

Prediction: Non-Carcinogen
Probability: 0.242
Enrichment: 0.753
Bayesian Score: -3.31
Mahalanobis Distance: 12.3
Mahalanobis Distance p-value: 0.00192

Prediction: Positive if the Bayesian score is above the estimated best cutoff value from minimizing the false positive and false negative rate.
Probability: The esimated probability that the sample is in the positive category. This assumes that the Bayesian score follows a normal distribution and is different from the prediction using a cutoff.
Enrichment: An estimate of enrichment, that is, the increased likelihood (versus random) of this sample being in the category.
Bayesian Score: The standard Laplacian-modified Bayesian score.
Mahalanobis Distance: The Mahalanobis distance (MD) is the distance to the center of the training data. The larger the MD, the less trustworthy the prediction.
Mahalanobis Distance p-value: The p-value gives the fraction of training data with an MD greater than or equal to the one for the given sample, assuming normally distributed data. The smaller the p-value, the less trustworthy the prediciton. For highly non-normal X properties (e.g., fingerprints), the MD p-value is wildly inaccurate.

Structural Similar Compounds

Name	Indomethacin	Paroxetine	Quinine
Structure	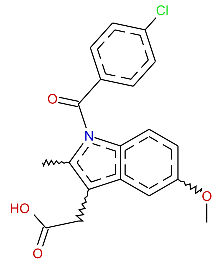	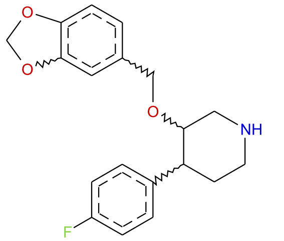	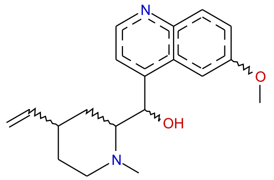
Actual Endpoint	Non-Carcinogen	Non-Carcinogen	Non-Carcinogen
Predicted Endpoint	Non-Carcinogen	Non-Carcinogen	Non-Carcinogen
Distance	0.565	0.592	0.598
Reference	US FDA (Centre for Drug Eval.& Res./Off. Testing & Res.) Sept. 1997	US FDA (Centre for Drug Eval.& Res./Off. Testing & Res.) Sept. 1997	US FDA (Centre for Drug Eval.& Res./Off. Testing & Res.) Sept. 1997

Model Applicability

Unknown features are fingerprint features in the query molecule, but not found or appearing too infrequently in the training set.

1. All properties and OPS components are within expected ranges.
2. Unknown ECFP_2 feature: -1508366470: [*][n+](:[*]):[*]
3. Unknown ECFP_2 feature: 1508268466: [*]C[n+](:[c]([*]):[*]):c:[*]
4. Unknown ECFP_2 feature: -1333923932: [*]CC[n+](:[*]):[*]
5. Unknown ECFP_2 feature: 2078070048: [*][n+](:[*]):[c]([c](:[*]):[*]):c:[*]
6. Unknown ECFP_2 feature: 688916016: [*][n+](:[*]):c:[c](:[*]):[*]

Feature Contribution

Top features for positive contribution				
**Fingerprint**	**Bit/Smiles**	**Feature Structure**	**Score**	**Carcinogen in training set**

ECFP_12	2052151141	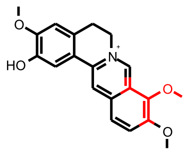 [*][c](:[*]):[c](OC):[c](:[*]):[*]	0.668	4 out of 5
ECFP_12	-1531301414	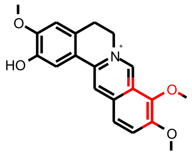 [*]O[c](:[c]([*]):[*]):[c](:[*]):[*]	0.454	5 out of 9
ECFP_12	51876938	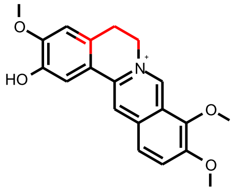 [*]CC[c](:[*]):[*]	0.232	18 out of 45
**Top Features for negative contribution**				
**Fingerprint**	**Bit/Smiles**	**Feature Structure**	**Score**	**Carcinogen in training set**
ECFP_12	2077607946	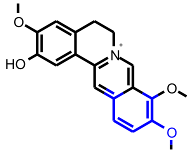 [*]O[c]1:[cH]:[cH]:[c](:[*]):[*]:[c]:1[*]	-1.25	0 out of 8
ECFP_12	1408898974	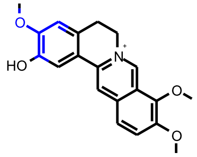 [*]O[c](:[cH]:[*]):[c]([*]):[*]	-0.517	5 out of 29

ECFP_12	1680623188	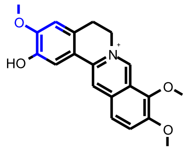<br>[*][c](:[*]):[c](OC):[cH]:[*]	-0.295	3 out of 14

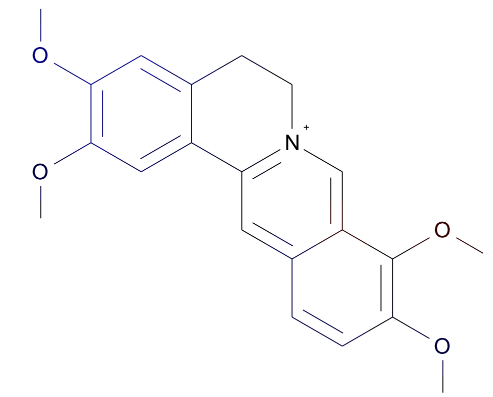

$C_{21}H_{22}NO_4$
Molecular Weight: 352.40368
ALogP: 4.161
Rotatable Bonds: 4
Acceptors: 4
Donors: 0

Structural Similar Compounds

Name	Ethynodiol	Chlorpromazine	Risperidone
Structure	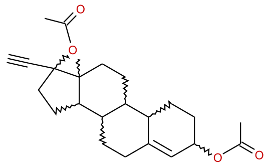	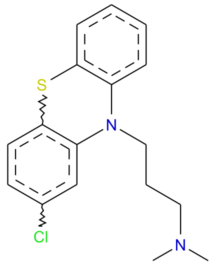	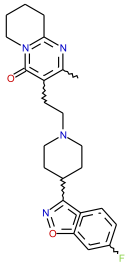
Actual Endpoint	Carcinogen	Non-Carcinogen	Carcinogen
Predicted Endpoint	Carcinogen	Non-Carcinogen	Carcinogen
Distance	0.583	0.604	0.619
Reference	US FDA (Centre for Drug Eval.& Res./Off. Testing & Res.) Sept. 1997	US FDA (Centre for Drug Eval.& Res./Off. Testing & Res.) Sept. 1997	US FDA (Centre for Drug Eval.& Res./Off. Testing & Res.) Sept. 1997

Model Prediction

Prediction: Non-Carcinogen
Probability: 0.235
Enrichment: 0.729
Bayesian Score: -3.87
Mahalanobis Distance: 11
Mahalanobis Distance p-value: 0.062

Prediction: Positive if the Bayesian score is above the estimated best cutoff value from minimizing the false positive and false negative rate.
Probability: The esimated probability that the sample is in the positive category. This assumes that the Bayesian score follows a normal distribution and is different from the prediction using a cutoff.
Enrichment: An estimate of enrichment, that is, the increased likelihood (versus random) of this sample being in the category.
Bayesian Score: The standard Laplacian-modified Bayesian score.
Mahalanobis Distance: The Mahalanobis distance (MD) is the distance to the center of the training data. The larger the MD, the less trustworthy the prediction.
Mahalanobis Distance p-value: The p-value gives the fraction of training data with an MD greater than or equal to the one for the given sample, assuming normally distributed data. The smaller the p-value, the less trustworthy the prediciton. For highly non-normal X properties (e.g., fingerprints), the MD p-value is wildly inaccurate.

Model Applicability

Unknown features are fingerprint features in the query molecule, but not found or appearing too infrequently in the training set.

1. All properties and OPS components are within expected ranges.
2. Unknown ECFP_2 feature: -1508366470: [*][n+](:[*]):[*]
3. Unknown ECFP_2 feature: 1508268466: [*]C[n+](:[c]([*]):[*]):c:[*]
4. Unknown ECFP_2 feature: -1333923932: [*]CC[n+](:[*]):[*]
5. Unknown ECFP_2 feature: 2078070048: [*][n+](:[*]):[c]([c](:[*]):[*]):c:[*]
6. Unknown ECFP_2 feature: 688916016: [*][n+](:[*]):c:[c](:[*]):[*]

Feature Contribution

Top features for positive contribution

Fingerprint	Bit/Smiles	Feature Structure	Score	Carcinogen in training set

ECFP_12	2052151141	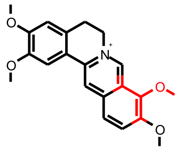 [*][c](:[*]):[c](OC):[c](:[*]):[*]	0.668	4 out of 5
ECFP_12	-1531301414	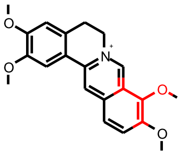 [*]O[c](:[c]([*]):[*]):[c](:[*]):[*]	0.454	5 out of 9
ECFP_12	51876938	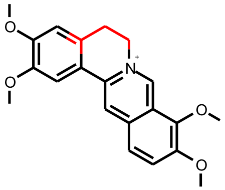 [*]CC[c](:[*]):[*]	0.232	18 out of 45

Top Features for negative contribution

Fingerprint	Bit/Smiles	Feature Structure	Score	Carcinogen in training set
ECFP_12	2077607946	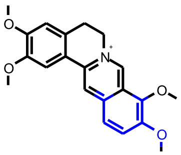 [*]O[c]1:[cH]:[cH]:[c](:[*]):[*]:[c]:1[*]	-1.25	0 out of 8
ECFP_12	-468366781	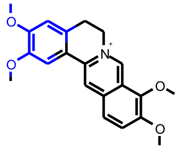 [*]O[c]1:[cH]:[*]:[c](:[*]):[cH]:[c]:1OC	-0.941	0 out of 5

ECFP_12	1408898974	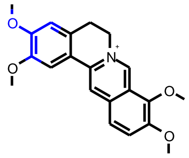 [*]O[c](:[cH]:[*]):[c]([*]):[*]	-0.517	5 out of 29

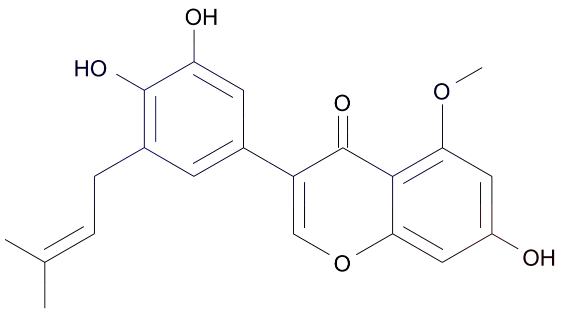

$C_{21}H_{20}O_6$
Molecular Weight: 368.3799
ALogP: 3.98
Rotatable Bonds: 4
Acceptors: 6
Donors: 3

Model Prediction

Prediction: Non-Carcinogen
Probability: 0.237
Enrichment: 0.736
Bayesian Score: -3.7
Mahalanobis Distance: 12.5
Mahalanobis Distance p-value: 0.000951

Prediction: Positive if the Bayesian score is above the estimated best cutoff value from minimizing the false positive and false negative rate.
Probability: The esimated probability that the sample is in the positive category. This assumes that the Bayesian score follows a normal distribution and is different from the prediction using a cutoff.
Enrichment: An estimate of enrichment, that is, the increased likelihood (versus random) of this sample being in the category.
Bayesian Score: The standard Laplacian-modified Bayesian score.
Mahalanobis Distance: The Mahalanobis distance (MD) is the distance to the center of the training data. The larger the MD, the less trustworthy the prediction.
Mahalanobis Distance p-value: The p-value gives the fraction of training data with an MD greater than or equal to the one for the given sample, assuming normally distributed data. The smaller the p-value, the less trustworthy the prediciton. For highly non-normal X properties (e.g., fingerprints), the MD p-value is wildly inaccurate.

Structural Similar Compounds

Name	Torsemide	Ursodiol	Clorazepate
Structure	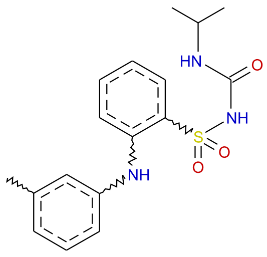	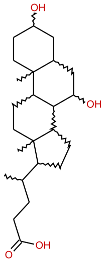	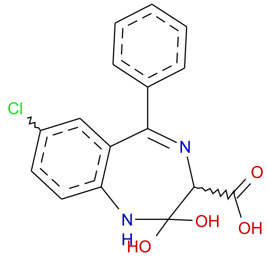
Actual Endpoint	Carcinogen	Carcinogen	Non-Carcinogen
Predicted Endpoint	Carcinogen	Carcinogen	Non-Carcinogen
Distance	0.684	0.689	0.709
Reference	US FDA (Centre for Drug Eval.& Res./Off. Testing & Res.) Sept. 1997	US FDA (Centre for Drug Eval.& Res./Off. Testing & Res.) Sept. 1997	US FDA (Centre for Drug Eval.& Res./Off. Testing & Res.) Sept. 1997

Model Applicability

Unknown features are fingerprint features in the query molecule, but not found or appearing too infrequently in the training set.

1. All properties and OPS components are within expected ranges.
2. Unknown ECFP_2 feature: 1717082529: [*]\C=C(/C(=[*])[*])\[c](:[*]):[*]
3. Unknown ECFP_2 feature: 471124258: [*]OC=C([*])[*]
4. Unknown ECFP_2 feature: -554736825: [*]=CO[c](:[*]):[*]
5. Unknown ECFP_2 feature: -1774681326: [*]C=C(C)C

Feature Contribution

Top features for positive contribution				
**Fingerprint**	**Bit/Smiles**	**Feature Structure**	**Score**	**Carcinogen in training set**
ECFP_12	-464490300	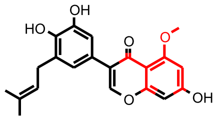 [*]C(=[*])[c]1:[c]([*]):[*]:[c]([*]):[cH]:[c]:1OC	0.421	1 out of 1

ECFP_12	-1925046727	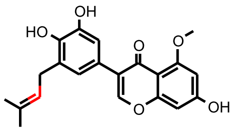 [*]C=[*]	0.407	16 out of 33
ECFP_12	-177786161	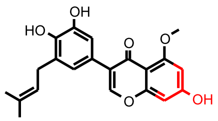 [*]:[cH]:[c](O):[cH]:[*]	0.341	7 out of 15
**Top Features for negative contribution**				
**Fingerprint**	**Bit/Smiles**	**Feature Structure**	**Score**	**Carcinogen in training set**
ECFP_12	-1660913849	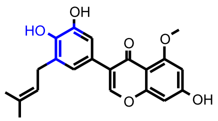 [*][c](:[*]):[c](O):[c]([*]):[*]	-0.941	0 out of 5
ECFP_12	1408898974	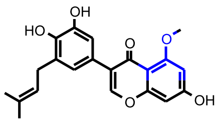 [*]O[c](:[cH]:[*]):[c]([*]):[*]	-0.517	5 out of 29
ECFP_12	-181568884	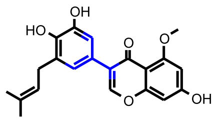 [*]C(=[*])[c](:[cH]:[*]):[cH]:[*]	-0.505	3 out of 18

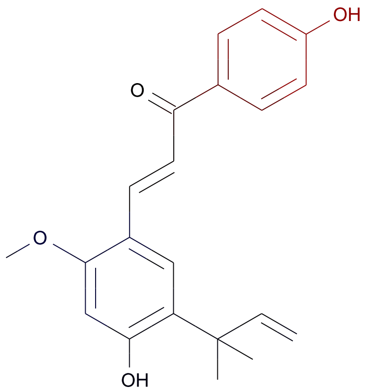

$C_{21}H_{22}O_4$
Molecular Weight: 338.39698
ALogP: 4.667
Rotatable Bonds: 6
Acceptors: 4
Donors: 2

Model Prediction

Prediction: Carcinogen
Probability: 0.297
Enrichment: 0.923
Bayesian Score: -0.0588
Mahalanobis Distance: 11.1
Mahalanobis Distance p-value: 0.0495

Prediction: Positive if the Bayesian score is above the estimated best cutoff value from minimizing the false positive and false negative rate.
Probability: The esimated probability that the sample is in the positive category. This assumes that the Bayesian score follows a normal distribution and is different from the prediction using a cutoff.
Enrichment: An estimate of enrichment, that is, the increased likelihood (versus random) of this sample being in the category.
Bayesian Score: The standard Laplacian-modified Bayesian score.
Mahalanobis Distance: The Mahalanobis distance (MD) is the distance to the center of the training data. The larger the MD, the less trustworthy the prediction.
Mahalanobis Distance p-value: The p-value gives the fraction of training data with an MD greater than or equal to the one for the given sample, assuming normally distributed data. The smaller the p-value, the less trustworthy the prediciton. For highly non-normal X properties (e.g., fingerprints), the MD p-value is wildly inaccurate.

Structural Similar Compounds

Name	Ethacrynic acid	Diclofenac	Indomethacin
Structure	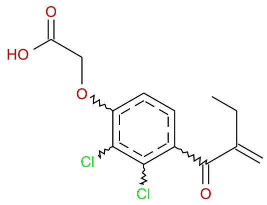	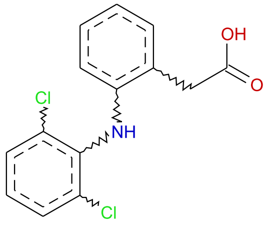	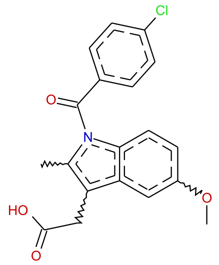
Actual Endpoint	Non-Carcinogen	Non-Carcinogen	Non-Carcinogen
Predicted Endpoint	Non-Carcinogen	Non-Carcinogen	Non-Carcinogen
Distance	0.631	0.642	0.648
Reference	US FDA (Centre for Drug Eval.& Res./Off. Testing & Res.) Sept. 1997	US FDA (Centre for Drug Eval.& Res./Off. Testing & Res.) Sept. 1997	US FDA (Centre for Drug Eval.& Res./Off. Testing & Res.) Sept. 1997

Model Applicability

Unknown features are fingerprint features in the query molecule, but not found or appearing too infrequently in the training set.

1. All properties and OPS components are within expected ranges.
2. Unknown ECFP_2 feature: -144557007: [*]=CC(C)(C)[c](:[*]):[*]
3. Unknown ECFP_2 feature: -1193716553: [*]C([*])([*])C=C
4. Unknown ECFP_2 feature: 1335702447: [*][c](:[*]):[c](C=[*]):c:[*]
5. Unknown ECFP_2 feature: 1430764055: [*]=CC(=O)[c](:[*]):[*]

Feature Contribution

Top features for positive contribution				
**Fingerprint**	**Bit/Smiles**	**Feature Structure**	**Score**	**Carcinogen in training set**
ECFP_12	1419645508	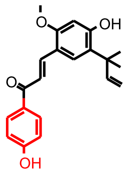 [*][c]1:[cH]:[cH]:[c](O):[cH]:[cH]:1	0.736	5 out of 6

ECFP_12	1740779540	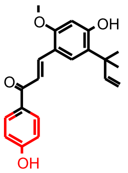 O[c]1:[cH]:[cH]:[*]:[cH]:[cH]:1	0.633	5 out of 7
ECFP_12	1187082817	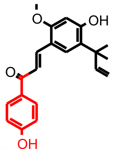 [*]C(=[*])[c]1:[cH]:[cH]:[c](O):[cH]:[cH]:1	0.613	2 out of 2

Top Features for negative contribution

Fingerprint	Bit/Smiles	Feature Structure	Score	Carcinogen in training set
ECFP_12	1408898974	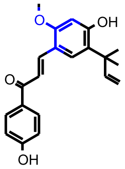 [*]O[c](:[cH]:[*]):[c]([*]):[*]	-0.517	5 out of 29
ECFP_12	-2090955291	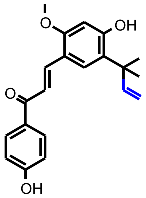 [*]C=C	-0.485	0 out of 2
ECFP_12	1680623188	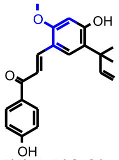 [*][c](:[*]):[c](OC):[cH]:[*]	-0.295	3 out of 14

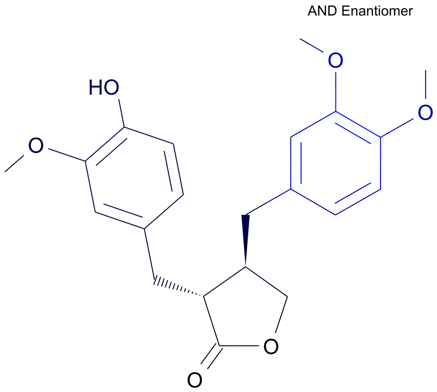

$C_{21}H_{24}O_6$
Molecular Weight: 372.41166
ALogP: 3.743
Rotatable Bonds: 7
Acceptors: 6
Donors: 1

Model Prediction

Prediction: Non-Carcinogen
Probability: 0.196
Enrichment: 0.608
Bayesian Score: -7.72
Mahalanobis Distance: 11
Mahalanobis Distance p-value: 0.0661

Prediction: Positive if the Bayesian score is above the estimated best cutoff value from minimizing the false positive and false negative rate.
Probability: The esimated probability that the sample is in the positive category. This assumes that the Bayesian score follows a normal distribution and is different from the prediction using a cutoff.
Enrichment: An estimate of enrichment, that is, the increased likelihood (versus random) of this sample being in the category.
Bayesian Score: The standard Laplacian-modified Bayesian score.
Mahalanobis Distance: The Mahalanobis distance (MD) is the distance to the center of the training data. The larger the MD, the less trustworthy the prediction.
Mahalanobis Distance p-value: The p-value gives the fraction of training data with an MD greater than or equal to the one for the given sample, assuming normally distributed data. The smaller the p-value, the less trustworthy the prediciton. For highly non-normal X properties (e.g., fingerprints), the MD p-value is wildly inaccurate.

Structural Similar Compounds

Name	Lovastatin	Felodipine	Moricizine
Structure	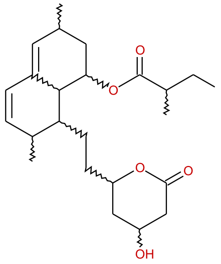	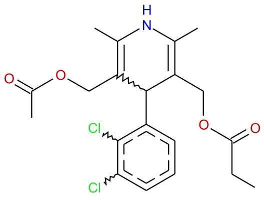	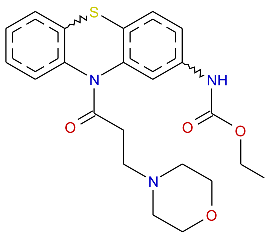
Actual Endpoint	Non-Carcinogen	Non-Carcinogen	Carcinogen
Predicted Endpoint	Carcinogen	Non-Carcinogen	Carcinogen
Distance	0.587	0.611	0.612
Reference	US FDA (Centre for Drug Eval.& Res./Off. Testing & Res.) Sept. 1997	US FDA (Centre for Drug Eval.& Res./Off. Testing & Res.) Sept. 1997	US FDA (Centre for Drug Eval.& Res./Off. Testing & Res.) Sept. 1997

Model Applicability

Unknown features are fingerprint features in the query molecule, but not found or appearing too infrequently in the training set.

1. All properties and OPS components are within expected ranges.
2. Unknown ECFP_2 feature: 771121623: [*]C([*])C[c](:[*]):[*]

Feature Contribution

Top features for positive contribution

Fingerprint	Bit/Smiles	Feature Structure	Score	Carcinogen in training set
ECFP_12	-556429595	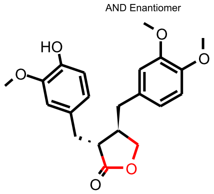 [*]=C1[*][*]CO1	0.33	3 out of 6

ECFP_12	683445015	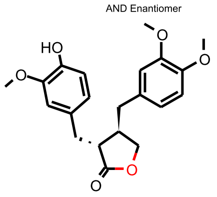 [*]O[*]	0.294	28 out of 66
ECFP_12	2106656448	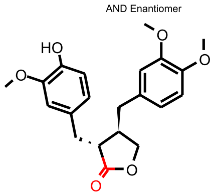 [*]C(=O)[*]	0.141	30 out of 83

Top Features for negative contribution

Fingerprint	Bit/Smiles	Feature Structure	Score	Carcinogen in training set
ECFP_12	2077607946	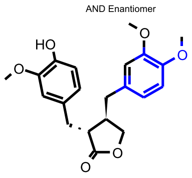 [*]O[c]1:[cH]:[cH]:[c](:[*]):[*]:[c]:1[*]	-1.25	0 out of 8
ECFP_12	-468366781	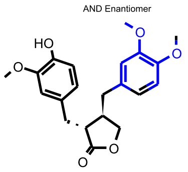 [*]O[c]1:[cH]:[*]:[c] (:[*]):[cH]:[c]:1OC	-0.941	0 out of 5
ECFP_12	1408898974	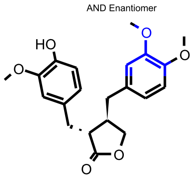 [*]O[c](:[cH]:[*]):[c]([*]):[*]	-0.517	5 out of 29

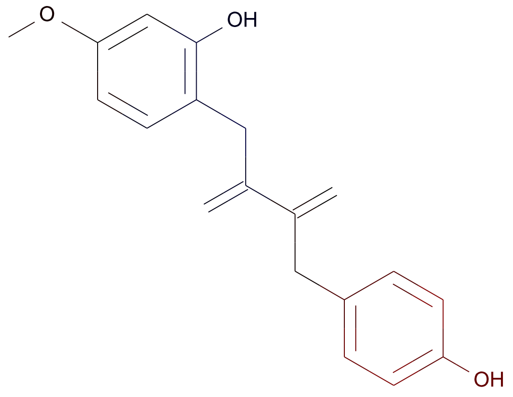

$C_{19}H_{20}O_3$
Molecular Weight: 296.3603
ALogP: 4.784
Rotatable Bonds: 6
Acceptors: 3
Donors: 2

Model Prediction

Prediction: Carcinogen
Probability: 0.296
Enrichment: 0.918
Bayesian Score: -0.145
Mahalanobis Distance: 10.9
Mahalanobis Distance p-value: 0.0759

Prediction: Positive if the Bayesian score is above the estimated best cutoff value from minimizing the false positive and false negative rate.
Probability: The esimated probability that the sample is in the positive category. This assumes that the Bayesian score follows a normal distribution and is different from the prediction using a cutoff.
Enrichment: An estimate of enrichment, that is, the increased likelihood (versus random) of this sample being in the category.
Bayesian Score: The standard Laplacian-modified Bayesian score.
Mahalanobis Distance: The Mahalanobis distance (MD) is the distance to the center of the training data. The larger the MD, the less trustworthy the prediction.
Mahalanobis Distance p-value: The p-value gives the fraction of training data with an MD greater than or equal to the one for the given sample, assuming normally distributed data. The smaller the p-value, the less trustworthy the prediciton. For highly non-normal X properties (e.g., fingerprints), the MD p-value is wildly inaccurate.

Structural Similar Compounds

Name	Diethylstilbesterol	Diclofenac	Penbutalol
Structure	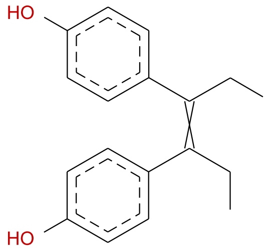	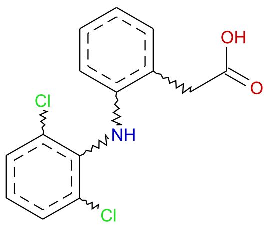	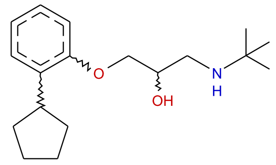
Actual Endpoint	Carcinogen	Non-Carcinogen	Non-Carcinogen
Predicted Endpoint	Carcinogen	Non-Carcinogen	Non-Carcinogen
Distance	0.594	0.596	0.609
Reference	US FDA (Centre for Drug Eval.& Res./Off. Testing & Res.) Sept. 1997	US FDA (Centre for Drug Eval.& Res./Off. Testing & Res.) Sept. 1997	US FDA (Centre for Drug Eval.& Res./Off. Testing & Res.) Sept. 1997

Model Applicability

Unknown features are fingerprint features in the query molecule, but not found or appearing too infrequently in the training set.

1. All properties and OPS components are within expected ranges.

Feature Contribution

Top features for positive contribution

Fingerprint	Bit/Smiles	Feature Structure	Score	Carcinogen in training set
ECFP_12	1419645508	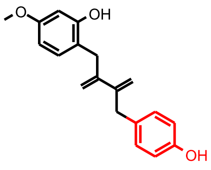 [*][c]1:[cH]:[cH]:[c](O):[cH]:[cH]:1	0.736	5 out of 6

ECFP_12	1740779540	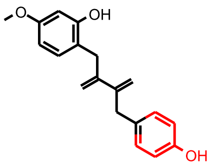 O[c]1:[cH]:[cH]:[*]:[cH]:[cH]:1	0.633	5 out of 7
ECFP_12	1737023319	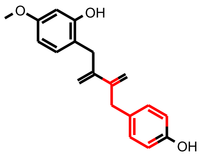 [*]C(=[*])C[c]1:[cH]:[cH]:[*]:[cH]:[cH]:1	0.613	2 out of 2
**Top Features for negative contribution**				
**Fingerprint**	**Bit/Smiles**	**Feature Structure**	**Score**	**Carcinogen in training set**
ECFP_12	-603928036	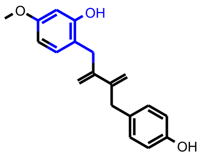 [*]C[c]1:[cH]:[*]:[c]([*]):[cH]:[c]:1O	-0.272	0 out of 1
ECFP_12	-1505409543	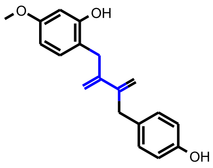 [*]CC(=C)C(=[*])[*]	-0.272	0 out of 1
ECFP_12	-2092468108	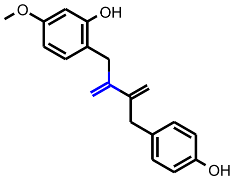 [*]C(=C)[*]	-0.272	0 out of 1

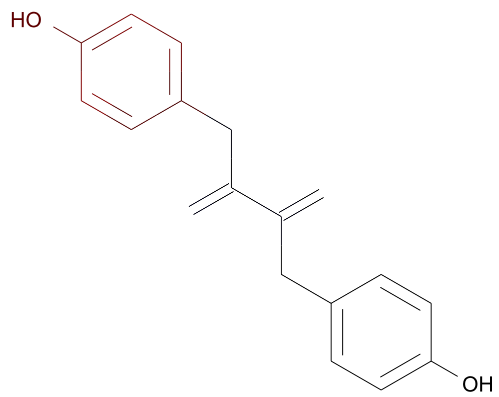

$C_{18}H_{18}O_2$
Molecular Weight: 266.33432
ALogP: 4.8
Rotatable Bonds: 5
Acceptors: 2
Donors: 2

Model Prediction

Prediction: Carcinogen
Probability: 0.341
Enrichment: 1.06
Bayesian Score: 1.91
Mahalanobis Distance: 10.3
Mahalanobis Distance p-value: 0.23

Prediction: Positive if the Bayesian score is above the estimated best cutoff value from minimizing the false positive and false negative rate.
Probability: The esimated probability that the sample is in the positive category. This assumes that the Bayesian score follows a normal distribution and is different from the prediction using a cutoff.
Enrichment: An estimate of enrichment, that is, the increased likelihood (versus random) of this sample being in the category.
Bayesian Score: The standard Laplacian-modified Bayesian score.
Mahalanobis Distance: The Mahalanobis distance (MD) is the distance to the center of the training data. The larger the MD, the less trustworthy the prediction.
Mahalanobis Distance p-value: The p-value gives the fraction of training data with an MD greater than or equal to the one for the given sample, assuming normally distributed data. The smaller the p-value, the less trustworthy the prediciton. For highly non-normal X properties (e.g., fingerprints), the MD p-value is wildly inaccurate.

Structural Similar Compounds

Name	Diethylstilbesterol	Dienestrol	Diclofenac
Structure	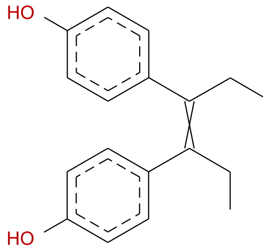	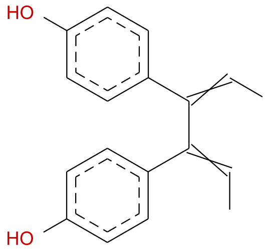	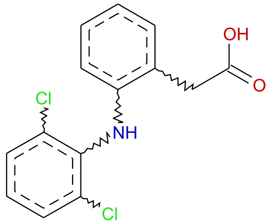
Actual Endpoint	Carcinogen	Carcinogen	Non-Carcinogen
Predicted Endpoint	Carcinogen	Carcinogen	Non-Carcinogen
Distance	0.465	0.530	0.591
Reference	US FDA (Centre for Drug Eval.& Res./Off. Testing & Res.) Sept. 1997	US FDA (Centre for Drug Eval.& Res./Off. Testing & Res.) Sept. 1997	US FDA (Centre for Drug Eval.& Res./Off. Testing & Res.) Sept. 1997

Model Applicability

Unknown features are fingerprint features in the query molecule, but not found or appearing too infrequently in the training set.

1. All properties and OPS components are within expected ranges.

Feature Contribution

Top features for positive contribution

Fingerprint	Bit/Smiles	Feature Structure	Score	Carcinogen in training set
ECFP_12	1419645508	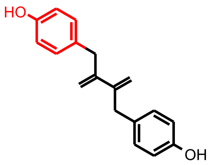 [*][c]1:[cH]:[cH]:[c](O):[cH]:[cH]:1	0.736	5 out of 6

ECFP_12	1740779540	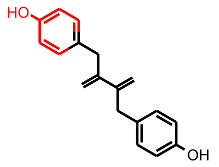 O[c]1:[cH]:[cH]:[*]:[cH]:[cH]:1	0.633	5 out of 7
ECFP_12	1737023319	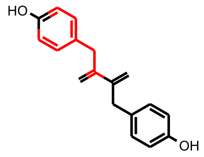 [*]C(=[*])C[c]1:[cH]:[cH]:[*]:[cH]:[cH]:1	0.613	2 out of 2
**Top Features for negative contribution**				
**Fingerprint**	**Bit/Smiles**	**Feature Structure**	**Score**	**Carcinogen in training set**
ECFP_12	-1505409543	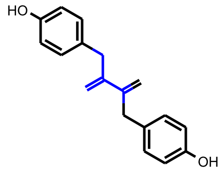 [*]CC(=C)C(=[*])[*]	-0.272	0 out of 1
ECFP_12	-2092468108	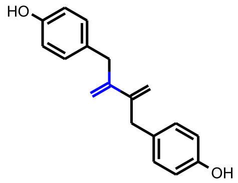 [*]C(=C)[*]	-0.272	0 out of 1
ECFP_12	1185660268	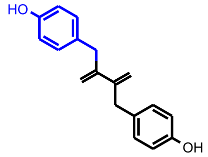 [*]C[c]1:[cH]:[cH]:[c](O):[cH]:[cH]:1	-0.272	0 out of 1

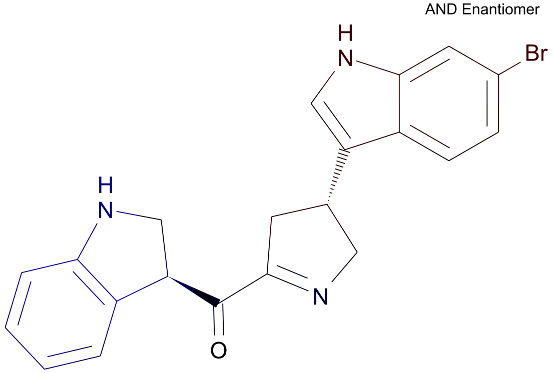

$C_{21}H_{18}BrN_3O$
Molecular Weight: 408.29112
ALogP: 3.919
Rotatable Bonds: 3
Acceptors: 3
Donors: 2

Model Prediction

Prediction: Non-Carcinogen
Probability: 0.265
Enrichment: 0.823
Bayesian Score: -1.82
Mahalanobis Distance: 12.1
Mahalanobis Distance p-value: 0.0031

Prediction: Positive if the Bayesian score is above the estimated best cutoff value from minimizing the false positive and false negative rate.
Probability: The esimated probability that the sample is in the positive category. This assumes that the Bayesian score follows a normal distribution and is different from the prediction using a cutoff.
Enrichment: An estimate of enrichment, that is, the increased likelihood (versus random) of this sample being in the category.
Bayesian Score: The standard Laplacian-modified Bayesian score.
Mahalanobis Distance: The Mahalanobis distance (MD) is the distance to the center of the training data. The larger the MD, the less trustworthy the prediction.
Mahalanobis Distance p-value: The p-value gives the fraction of training data with an MD greater than or equal to the one for the given sample, assuming normally distributed data. The smaller the p-value, the less trustworthy the prediciton. For highly non-normal X properties (e.g., fingerprints), the MD p-value is wildly inaccurate.

Structural Similar Compounds

Name	Mefloquine	Finasteride	Butorphanol
Structure	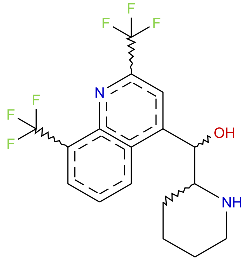	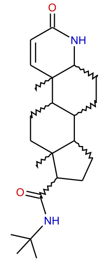	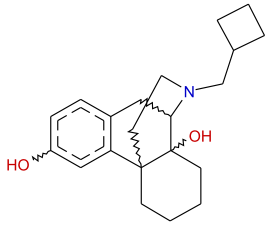
Actual Endpoint	Non-Carcinogen	Non-Carcinogen	Non-Carcinogen
Predicted Endpoint	Non-Carcinogen	Non-Carcinogen	Non-Carcinogen
Distance	0.571	0.626	0.641
Reference	US FDA (Centre for Drug Eval.& Res./Off. Testing & Res.) Sept. 1997	US FDA (Centre for Drug Eval.& Res./Off. Testing & Res.) Sept. 1997	US FDA (Centre for Drug Eval.& Res./Off. Testing & Res.) Sept. 1997

Model Applicability

Unknown features are fingerprint features in the query molecule, but not found or appearing too infrequently in the training set.

1. All properties and OPS components are within expected ranges.
2. Unknown ECFP_2 feature: -177935549: [*]:c:[c](Br):c:[*]
3. Unknown ECFP_2 feature: -116689887: [*][C@H]1[*][*]=NC1
4. Unknown ECFP_2 feature: 103000222: [*]C(=[*])C1=N[*][*]C1
5. Unknown ECFP_2 feature: 1431365708: [*]C([*])C(=O)C(=[*])[*]

Feature Contribution

Top features for positive contribution

Fingerprint	Bit/Smiles	Feature Structure	Score	Carcinogen in training set
ECFP_12	459826767	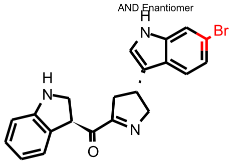 [*]:[c](:[*])Br	0.613	2 out of 2

ECFP_12	-302078100	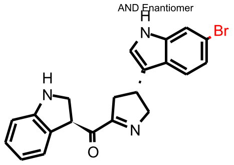 [*]Br	0.575	3 out of 4
ECFP_12	53207596	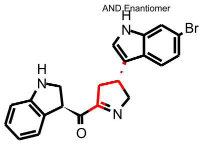 [*]C([*])C[c](:[*]):[*]	0.459	8 out of 15

Top Features for negative contribution

Fingerprint	Bit/Smiles	Feature Structure	Score	Carcinogen in training set
ECFP_12	1335833675	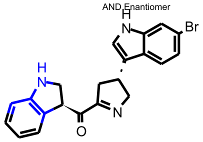 [*]:[cH]:[c]1N[*][*][c]:1:[*]	-1.25	0 out of 8
ECFP_12	2090054846	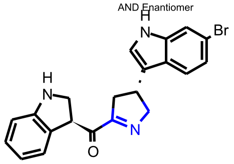 [*]C1=NC[*][*]1	-0.797	1 out of 11
ECFP_12	769323258	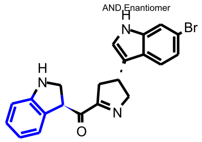 [*][C@@H]1[*][*][c]2:[cH]:[cH]:[cH]:[cH]:[c]1:2	-0.56	1 out of 8

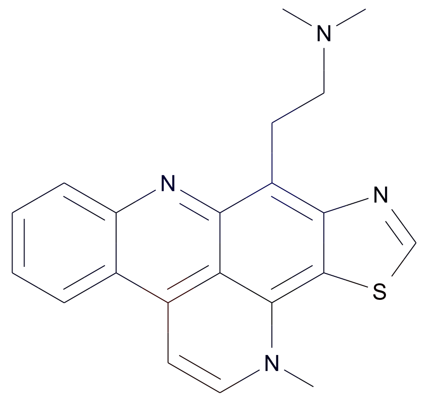

$C_{21}H_{20}N_4S$
Molecular Weight: 360.4753
ALogP: 3.682
Rotatable Bonds: 3
Acceptors: 4
Donors: 0

Structural Similar Compounds

Name	Chlormadinone	Risperidone	Metiapine
Structure	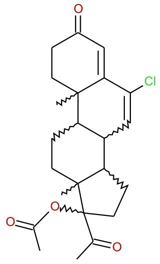	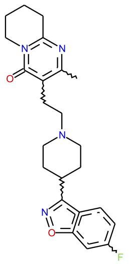	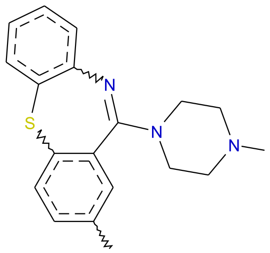
Actual Endpoint	Non-Carcinogen	Carcinogen	Non-Carcinogen
Predicted Endpoint	Non-Carcinogen	Carcinogen	Non-Carcinogen
Distance	0.576	0.592	0.618
Reference	US FDA (Centre for Drug Eval.& Res./Off. Testing & Res.) Sept. 1997	US FDA (Centre for Drug Eval.& Res./Off. Testing & Res.) Sept. 1997	US FDA (Centre for Drug Eval.& Res./Off. Testing & Res.) Sept. 1997

Model Prediction

Prediction: Non-Carcinogen
Probability: 0.262
Enrichment: 0.812
Bayesian Score: -2.04
Mahalanobis Distance: 12.1
Mahalanobis Distance p-value: 0.00305

Prediction: Positive if the Bayesian score is above the estimated best cutoff value from minimizing the false positive and false negative rate.
Probability: The esimated probability that the sample is in the positive category. This assumes that the Bayesian score follows a normal distribution and is different from the prediction using a cutoff.
Enrichment: An estimate of enrichment, that is, the increased likelihood (versus random) of this sample being in the category.
Bayesian Score: The standard Laplacian-modified Bayesian score.
Mahalanobis Distance: The Mahalanobis distance (MD) is the distance to the center of the training data. The larger the MD, the less trustworthy the prediction.
Mahalanobis Distance p-value: The p-value gives the fraction of training data with an MD greater than or equal to the one for the given sample, assuming normally distributed data. The smaller the p-value, the less trustworthy the prediciton. For highly non-normal X properties (e.g., fingerprints), the MD p-value is wildly inaccurate.

Model Applicability

Unknown features are fingerprint features in the query molecule, but not found or appearing too infrequently in the training set.

1. All properties and OPS components are within expected ranges.
2. Unknown ECFP_2 feature: 1618095312: [*]=CN(C)[c](:[*]):[*]
3. Unknown ECFP_2 feature: -1658647648: [*]=C[c](:[c](:[*]):[*]):[c](:[*]):[*]
4. Unknown ECFP_2 feature: -1673960248: [*][c](:[*]):[c]1:s:[*]:[*]:[c]:1:[*]

Feature Contribution

Top features for positive contribution

Fingerprint	Bit/Smiles	Feature Structure	Score	Carcinogen in training set
ECFP_12	914325265	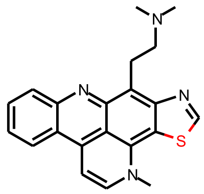 [*]:s:[*]	0.516	8 out of 14

ECFP_12	-1661653144	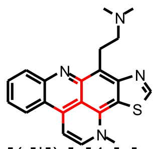 [*][c](:[*]):[c]1:[c](:[*]):[*]:[*]:[c]:1:[*]	0.437	2 out of 3
ECFP_12	1306977740	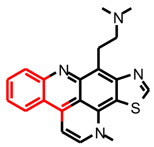 [*]:[c]1:[*]:[*]:[c]2:[cH]:[cH]:[cH]:[cH]:[c]:1:2	0.271	4 out of 9

Top Features for negative contribution

Fingerprint	Bit/Smiles	Feature Structure	Score	Carcinogen in training set
ECFP_12	-512323383	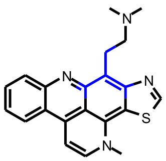 [*]C[c](:[c](:[*]):[*]):[c](:[*]):[*]	-0.941	0 out of 5
ECFP_12	-586331102	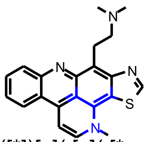 [*]N([*])[c](:[c](:[*]):[*]):[c](:[*]):[*]	-0.485	0 out of 2
ECFP_12	1997021792	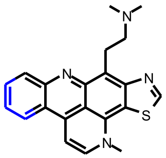 [*]:[cH]:[cH]:[cH]:[*]	-0.296	36 out of 156

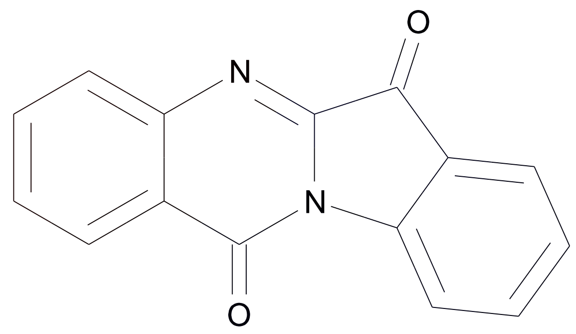

$C_{15}H_8N_2O_2$

Molecular Weight: 248.23621

ALogP: 2.331

Rotatable Bonds: 0

Acceptors: 3

Donors: 0

Structural Similar Compounds

Name	Methoxsalen; 8-	Levamisole	Estazolam
Structure	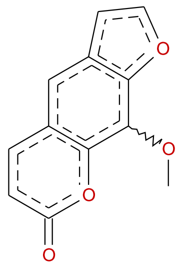	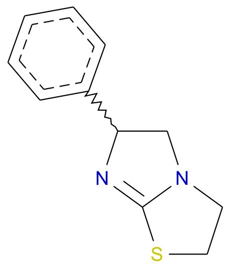	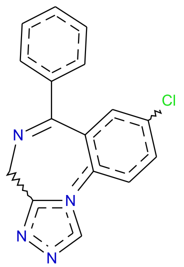
Actual Endpoint	Carcinogen	Non-Carcinogen	Non-Carcinogen
Predicted Endpoint	Carcinogen	Non-Carcinogen	Non-Carcinogen
Distance	0.564	0.565	0.631
Reference	US FDA (Centre for Drug Eval.& Res./Off. Testing & Res.) Sept. 1997	US FDA (Centre for Drug Eval.& Res./Off. Testing & Res.) Sept. 1997	US FDA (Centre for Drug Eval.& Res./Off. Testing & Res.) Sept. 1997

Model Prediction

Prediction: Carcinogen

Probability: 0.288

Enrichment: 0.895

Bayesian Score: -0.521

Mahalanobis Distance: 7.01

Mahalanobis Distance p-value: 1

Prediction: Positive if the Bayesian score is above the estimated best cutoff value from minimizing the false positive and false negative rate.
Probability: The esimated probability that the sample is in the positive category. This assumes that the Bayesian score follows a normal distribution and is different from the prediction using a cutoff.
Enrichment: An estimate of enrichment, that is, the increased likelihood (versus random) of this sample being in the category.
Bayesian Score: The standard Laplacian-modified Bayesian score.
Mahalanobis Distance: The Mahalanobis distance (MD) is the distance to the center of the training data. The larger the MD, the less trustworthy the prediction.
Mahalanobis Distance p-value: The p-value gives the fraction of training data with an MD greater than or equal to the one for the given sample, assuming normally distributed data. The smaller the p-value, the less trustworthy the prediciton. For highly non-normal X properties (e.g., fingerprints), the MD p-value is wildly inaccurate.

Model Applicability

Unknown features are fingerprint features in the query molecule, but not found or appearing too infrequently in the training set.

1. All properties and OPS components are within expected ranges.
2. Unknown ECFP_2 feature: -962771238: [*]C(=[*])N1C(=[*])[*][*]:[c]1:[*]

Feature Contribution

Top features for positive contribution

Fingerprint	Bit/Smiles	Feature Structure	Score	Carcinogen in training set
ECFP_12	-1106753576	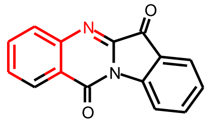 [*][c]1:[*]:[cH]:[cH]:[cH]:[c]:1N=[*]	0.208	1 out of 2

ECFP_12	359396774	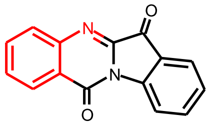 [*][c]1:[cH]:[cH]:[cH]:[cH]:[c]:1N=[*]	0.208	1 out of 2
ECFP_12	2106656448	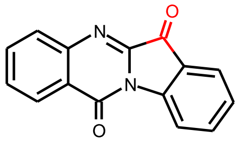 [*]C(=O)[*]	0.141	30 out of 83

Top Features for negative contribution

**Fingerprint**	**Bit/Smiles**	**Feature Structure**	**Score**	**Carcinogen in training set**
ECFP_12	1717462980	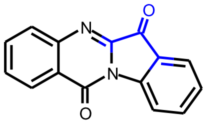 [*]C(=[*])C(=O)[c](:[*]):[*]	-0.363	1 out of 6
ECFP_12	1997021792	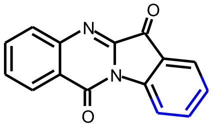 [*]:[cH]:[cH]:[cH]:[*]	-0.296	36 out of 156
ECFP_12	1945129186	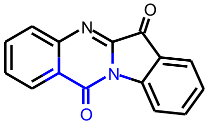 [*]N([*])C(=O)[c](:[*]):[*]	-0.248	1 out of 5

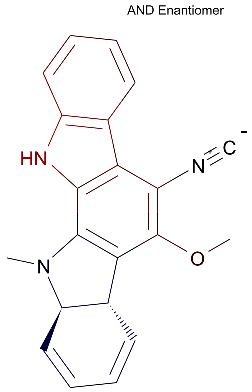

$C_{21}H_{17}N_3O$
Molecular Weight: 327.37918
ALogP: 4.078
Rotatable Bonds: 1
Acceptors: 2
Donors: 1

Model Prediction

Prediction: Carcinogen
Probability: 0.331
Enrichment: 1.03
Bayesian Score: 1.5
Mahalanobis Distance: 11.3
Mahalanobis Distance p-value: 0.0312

Prediction: Positive if the Bayesian score is above the estimated best cutoff value from minimizing the false positive and false negative rate.
Probability: The esimated probability that the sample is in the positive category. This assumes that the Bayesian score follows a normal distribution and is different from the prediction using a cutoff.
Enrichment: An estimate of enrichment, that is, the increased likelihood (versus random) of this sample being in the category.
Bayesian Score: The standard Laplacian-modified Bayesian score.
Mahalanobis Distance: The Mahalanobis distance (MD) is the distance to the center of the training data. The larger the MD, the less trustworthy the prediction.
Mahalanobis Distance p-value: The p-value gives the fraction of training data with an MD greater than or equal to the one for the given sample, assuming normally distributed data. The smaller the p-value, the less trustworthy the prediciton. For highly non-normal X properties (e.g., fingerprints), the MD p-value is wildly inaccurate.

Structural Similar Compounds

Name	Levonorgestrel	Norethindrone	Norethynodrel
Structure	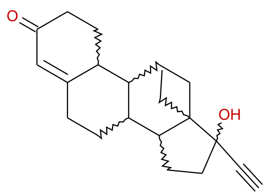	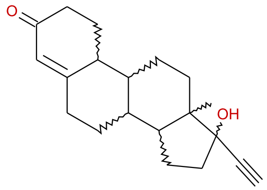	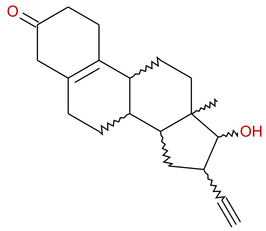
Actual Endpoint	Non-Carcinogen	Carcinogen	Carcinogen
Predicted Endpoint	Carcinogen	Carcinogen	Carcinogen
Distance	0.591	0.595	0.596
Reference	US FDA (Centre for Drug Eval.& Res./Off. Testing & Res.) Sept. 1997	US FDA (Centre for Drug Eval.& Res./Off. Testing & Res.) Sept. 1997	US FDA (Centre for Drug Eval.& Res./Off. Testing & Res.) Sept. 1997

Model Applicability

Unknown features are fingerprint features in the query molecule, but not found or appearing too infrequently in the training set.

1. All properties and OPS components are within expected ranges.
2. Unknown ECFP_2 feature: 1029014155: [*][N+]#[*]
3. Unknown ECFP_2 feature: 726108635: [*]#[C-]
4. Unknown ECFP_2 feature: 1464683384: [*][c](:[*]):[c]([N+]#[*]):[c](:[*]):[*]
5. Unknown ECFP_2 feature: -11961319: [*]:[c](:[*])[N+]#[C-]
6. Unknown ECFP_2 feature: -1334780583: [*][N+]#[C-]

Feature Contribution

Top features for positive contribution

Fingerprint	Bit/Smiles	Feature Structure	Score	Carcinogen in training set

ECFP_12	2052151141	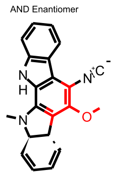<br>[*][c](:[*]):[c](OC):<br>[c](:[*]):[*]	0.668	4 out of 5
ECFP_12	558201926	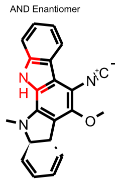<br>[*]:[c]1:[*]:[*]:[c](<br>:[*]):[nH]:1	0.539	5 out of 8
ECFP_12	1099224616	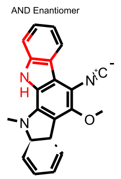<br>[*]:[cH]:[c]1:[nH]:[*<br>]:[*]:[c]:1:[*]	0.456	6 out of 11

Top Features for negative contribution

Fingerprint	Bit/Smiles	Feature Structure	Score	Carcinogen in training set
ECFP_12	-586331102	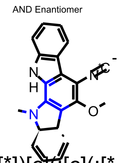<br>[*]N([*])[c](:[c](:[*<br>]):[*]):[c](:[*]):[*<br>]	-0.485	0 out of 2
ECFP_12	-1565641546	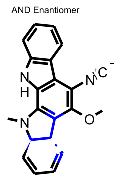<br>[*][C@H]1[*][*]:[c](:<br>[*])[C@@H]1C=[*]	-0.485	0 out of 2

ECFP_12	890368401	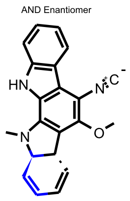<br>[*]\C=C/C([*])[*]	-0.466	1 out of 7

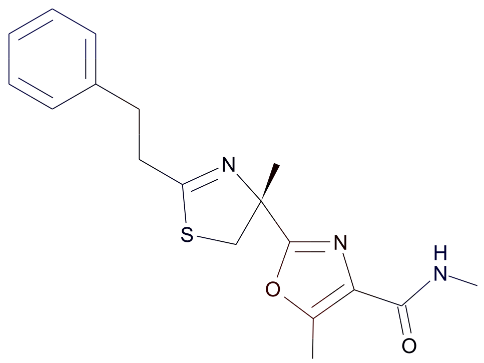

$C_{18}H_{21}N_3O_2S$
Molecular Weight: 343.44324
ALogP: 2.716
Rotatable Bonds: 5
Acceptors: 4
Donors: 1

Structural Similar Compounds

Name	Omeprazole	Lansoprazole	Oxaprocin
Structure	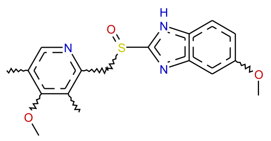	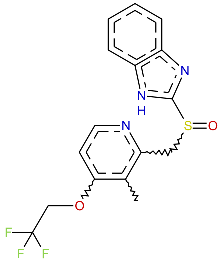	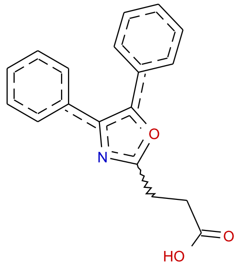
Actual Endpoint	Carcinogen	Carcinogen	Non-Carcinogen
Predicted Endpoint	Carcinogen	Carcinogen	Non-Carcinogen
Distance	0.566	0.571	0.600
Reference	US FDA (Centre for Drug Eval.& Res./Off. Testing & Res.) Sept. 1997	US FDA (Centre for Drug Eval.& Res./Off. Testing & Res.) Sept. 1997	US FDA (Centre for Drug Eval.& Res./Off. Testing & Res.) Sept. 1997

Model Prediction

Prediction: Non-Carcinogen
Probability: 0.257
Enrichment: 0.798
Bayesian Score: -2.33
Mahalanobis Distance: 13.2
Mahalanobis Distance p-value: 5.7e-005

Prediction: Positive if the Bayesian score is above the estimated best cutoff value from minimizing the false positive and false negative rate.
Probability: The esimated probability that the sample is in the positive category. This assumes that the Bayesian score follows a normal distribution and is different from the prediction using a cutoff.
Enrichment: An estimate of enrichment, that is, the increased likelihood (versus random) of this sample being in the category.
Bayesian Score: The standard Laplacian-modified Bayesian score.
Mahalanobis Distance: The Mahalanobis distance (MD) is the distance to the center of the training data. The larger the MD, the less trustworthy the prediction.
Mahalanobis Distance p-value: The p-value gives the fraction of training data with an MD greater than or equal to the one for the given sample, assuming normally distributed data. The smaller the p-value, the less trustworthy the prediciton. For highly non-normal X properties (e.g., fingerprints), the MD p-value is wildly inaccurate.

Model Applicability

Unknown features are fingerprint features in the query molecule, but not found or appearing too infrequently in the training set.

1. All properties and OPS components are within expected ranges.
2. Unknown ECFP_2 feature: 309047694: [*]C([*])([*])[c]1:o:[*]:[*]:n:1
3. Unknown ECFP_2 feature: 1576608821: [*][c]1:[*]:[*]:o:[c]:1C
4. Unknown ECFP_2 feature: -1073216586: [*]CC1=N[*][*]S1
5. Unknown ECFP_2 feature: 1920241679: [*]C1([*])[*]=[*]SC1
6. Unknown ECFP_2 feature: 618128563: [*]:[c](:[*])[C@]1(C)C[*][*]=N1

Feature Contribution

Top features for positive contribution				
Fingerprint	Bit/Smiles	Feature Structure	Score	Carcinogen in training set

ECFP_12	1203316083	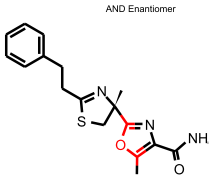 [*][c]1:[*]:[*]:[c]([*]):o:1	0.681	9 out of 13
ECFP_12	1051700121	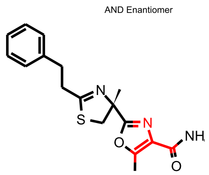 [*]C(=[*])[c]1:n:[*]: [*]:[c]:1[*]	0.437	2 out of 3
ECFP_12	683445015	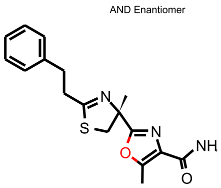 [*]O[*]	0.294	28 out of 66

Top Features for negative contribution

Fingerprint	Bit/Smiles	Feature Structure	Score	Carcinogen in training set
ECFP_12	1571214559	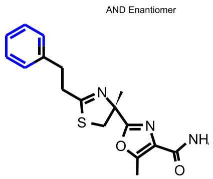 [*]1:[cH]:[cH]:[cH]:[cH]:[cH]:1	-0.56	11 out of 64
ECFP_12	-281505363	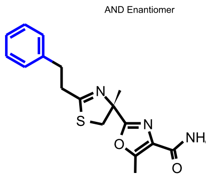 [*][c]1:[cH]:[cH]:[cH]:[cH]:[cH]:1	-0.56	11 out of 64

ECFP_12	1338334141	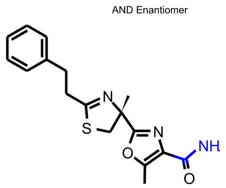 [*]C(=[*])NC	-0.485	0 out of 2

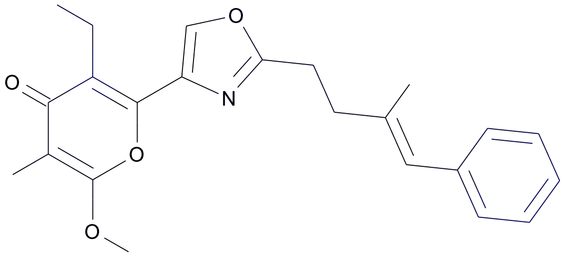

$C_{23}H_{25}NO_4$
Molecular Weight: 379.4489
ALogP: 5.22
Rotatable Bonds: 7
Acceptors: 4
Donors: 0

Model Prediction

Prediction: Non-Carcinogen
Probability: 0.229
Enrichment: 0.713
Bayesian Score: -4.3
Mahalanobis Distance: 14.7
Mahalanobis Distance p-value: 9.04e-008

Prediction: Positive if the Bayesian score is above the estimated best cutoff value from minimizing the false positive and false negative rate.
Probability: The esimated probability that the sample is in the positive category. This assumes that the Bayesian score follows a normal distribution and is different from the prediction using a cutoff.
Enrichment: An estimate of enrichment, that is, the increased likelihood (versus random) of this sample being in the category.
Bayesian Score: The standard Laplacian-modified Bayesian score.
Mahalanobis Distance: The Mahalanobis distance (MD) is the distance to the center of the training data. The larger the MD, the less trustworthy the prediction.
Mahalanobis Distance p-value: The p-value gives the fraction of training data with an MD greater than or equal to the one for the given sample, assuming normally distributed data. The smaller the p-value, the less trustworthy the prediciton. For highly non-normal X properties (e.g., fingerprints), the MD p-value is wildly inaccurate.

Structural Similar Compounds

Name	Permethrin	Chlorotrianisene	Flurazepam
Structure	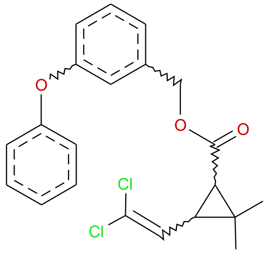	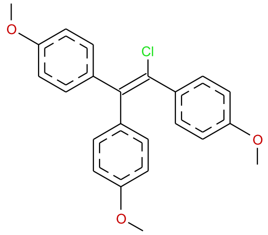	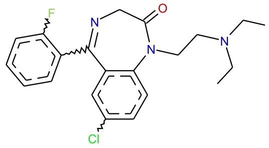
Actual Endpoint	Non-Carcinogen	Non-Carcinogen	Non-Carcinogen
Predicted Endpoint	Non-Carcinogen	Non-Carcinogen	Non-Carcinogen
Distance	0.583	0.625	0.635
Reference	US FDA (Centre for Drug Eval.& Res./Off. Testing & Res.) Sept. 1997	US FDA (Centre for Drug Eval.& Res./Off. Testing & Res.) Sept. 1997	US FDA (Centre for Drug Eval.& Res./Off. Testing & Res.) Sept. 1997

Model Applicability

Unknown features are fingerprint features in the query molecule, but not found or appearing too infrequently in the training set.

1. All properties and OPS components are within expected ranges.
2. Unknown ECFP_2 feature: 1651701028: [*]OC(=C([*])[*])O[*]
3. Unknown ECFP_2 feature: -785659985: [*][c]1:[*]:[*]:o:c:1
4. Unknown ECFP_2 feature: -1832568576: [*]C(=C[c](:[*]):[*])[*]
5. Unknown ECFP_2 feature: -176483725: [*]=C[c](:c:[*]):c:[*]

Feature Contribution

Top features for positive contribution

Fingerprint	Bit/Smiles	Feature Structure	Score	Carcinogen in training set
ECFP_12	-1925046727	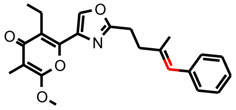 [*]C=[*]	0.407	16 out of 33

ECFP_12	683445015	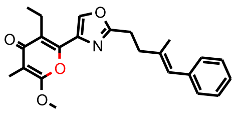 [*]O[*]	0.294	28 out of 66
ECFP_12	-428002189	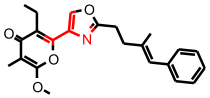 [*]C(=[*])[c]1:[cH]:[*]:[*]:n:1	0.208	1 out of 2

Top Features for negative contribution

Fingerprint	Bit/Smiles	Feature Structure	Score	Carcinogen in training set
ECFP_12	767488533	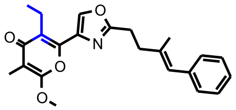 [*]C(=[*])CC	-0.941	0 out of 5
ECFP_12	1571214559	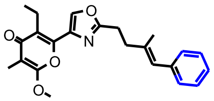 [*]1:[cH]:[cH]:[cH]:[cH]:[cH]:1	-0.56	11 out of 64
ECFP_12	-281505363	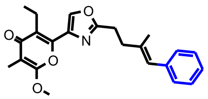 [*][c]1:[cH]:[cH]:[cH]:[cH]:[cH]:1	-0.56	11 out of 64

Remdesivir

TOPKAT_Rat_Female_FDA_None_vs_Carcinogen

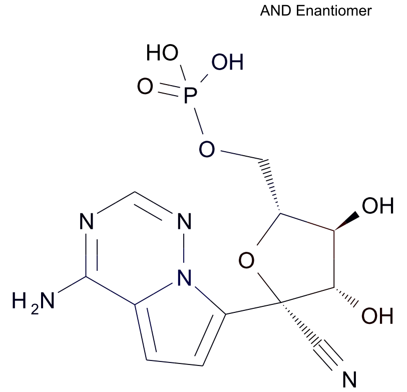

$C_{12}H_{14}N_5O_7P$
Molecular Weight: 371.24262
ALogP: -1.502
Rotatable Bonds: 4
Acceptors: 11
Donors: 5

Model Prediction

Prediction: Non-Carcinogen
Probability: 0.243
Enrichment: 0.756
Bayesian Score: -3.24
Mahalanobis Distance: 13.8
Mahalanobis Distance p-value: 5.04e-006

Prediction: Positive if the Bayesian score is above the estimated best cutoff value from minimizing the false positive and false negative rate.
Probability: The esimated probability that the sample is in the positive category. This assumes that the Bayesian score follows a normal distribution and is different from the prediction using a cutoff.
Enrichment: An estimate of enrichment, that is, the increased likelihood (versus random) of this sample being in the category.
Bayesian Score: The standard Laplacian-modified Bayesian score.
Mahalanobis Distance: The Mahalanobis distance (MD) is the distance to the center of the training data. The larger the MD, the less trustworthy the prediction.
Mahalanobis Distance p-value: The p-value gives the fraction of training data with an MD greater than or equal to the one for the given sample, assuming normally distributed data. The smaller the p-value, the less trustworthy the prediciton. For highly non-normal X properties (e.g., fingerprints), the MD p-value is wildly inaccurate.

Structural Similar Compounds

Name	Streptozocin	Tetracycline	Famotidine
Structure	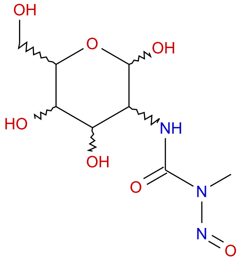	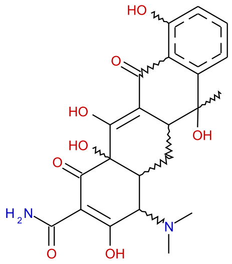	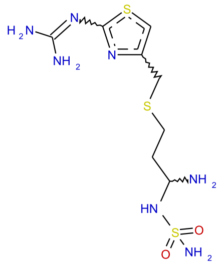
Actual Endpoint	Carcinogen	Non-Carcinogen	Non-Carcinogen
Predicted Endpoint	Carcinogen	Non-Carcinogen	Non-Carcinogen
Distance	0.810	0.858	0.861
Reference	US FDA (Centre for Drug Eval.& Res./Off. Testing & Res.) Sept. 1997	US FDA (Centre for Drug Eval.& Res./Off. Testing & Res.) Sept. 1997	US FDA (Centre for Drug Eval.& Res./Off. Testing & Res.) Sept. 1997

Model Applicability

Unknown features are fingerprint features in the query molecule, but not found or appearing too infrequently in the training set.

1. All properties and OPS components are within expected ranges.
2. Unknown ECFP_2 feature: 1126642748: [*]OP(=O)(O)O
3. Unknown ECFP_2 feature: -1250439909: [*]COP(=[*])([*])[*]
4. Unknown ECFP_2 feature: 1258791451: [*][C@H]1[*][*]O[C@]1(C#[*])[c](:[*]):[*]
5. Unknown ECFP_2 feature: -1507082173: [*][c]1:[*]:[*]:[c](:[*]):n:1:n:[*]
6. Unknown ECFP_2 feature: -66263742: [*]C([*])([*])[c]1:n(:[*]):[*]:[*]:c:1

Feature Contribution

Top features for positive contribution				
Fingerprint	Bit/Smiles	Feature Structure	Score	Carcinogen in training set

ECFP_12	-553149446	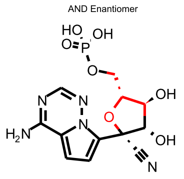 [*]C[C@H]1O[*][*][C@@H]1[*]	0.575	3 out of 4
ECFP_12	-1114776580	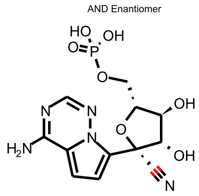 [*]C#[*]	0.461	10 out of 19
ECFP_12	-521596699	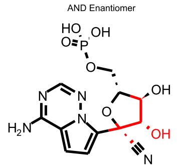 [*][C@@H]1[*][*]C([*])([*])[C@H]1O	0.445	3 out of 5

Top Features for negative contribution

Fingerprint	Bit/Smiles	Feature Structure	Score	Carcinogen in training set
ECFP_12	-1687549011	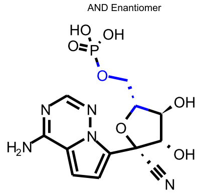 [*]OCC([*])[*]	-0.661	0 out of 3
ECFP_12	2024329577	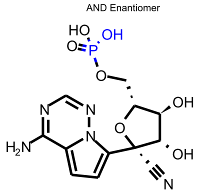 [*]P(=[*])([*])O	-0.661	0 out of 3

ECFP_12	-1734834311	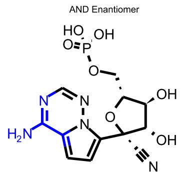 [*]:n:[c](N):[c](:[*])<br>):[*]	-0.56	1 out of 8

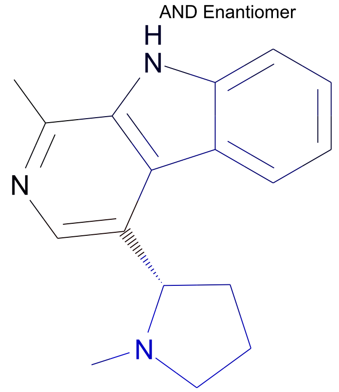

$C_{17}H_{19}N_3$
Molecular Weight: 265.35286
ALogP: 3.018
Rotatable Bonds: 1
Acceptors: 2
Donors: 1

Model Prediction

Prediction: Single-Carcinogen
Probability: 0.269
Enrichment: 0.72
Bayesian Score: -5.67
Mahalanobis Distance: 10.6
Mahalanobis Distance p-value: 0.0245

Prediction: Positive if the Bayesian score is above the estimated best cutoff value from minimizing the false positive and false negative rate.
Probability: The esimated probability that the sample is in the positive category. This assumes that the Bayesian score follows a normal distribution and is different from the prediction using a cutoff.
Enrichment: An estimate of enrichment, that is, the increased likelihood (versus random) of this sample being in the category.
Bayesian Score: The standard Laplacian-modified Bayesian score.
Mahalanobis Distance: The Mahalanobis distance (MD) is the distance to the center of the training data. The larger the MD, the less trustworthy the prediction.
Mahalanobis Distance p-value: The p-value gives the fraction of training data with an MD greater than or equal to the one for the given sample, assuming normally distributed data. The smaller the p-value, the less trustworthy the prediciton. For highly non-normal X properties (e.g., fingerprints), the MD p-value is wildly inaccurate.

Structural Similar Compounds

Name	Granisetron	Nicotine	Pergolide
Structure	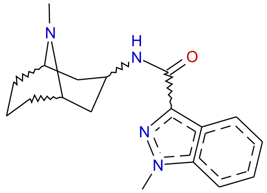	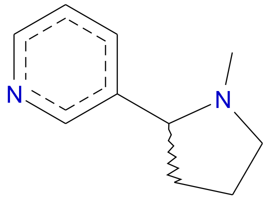	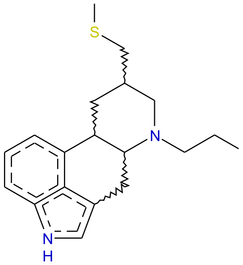
Actual Endpoint	Single-Carcinogen	Single-Carcinogen	Single-Carcinogen
Predicted Endpoint	Single-Carcinogen	Single-Carcinogen	Single-Carcinogen
Distance	0.550	0.558	0.567
Reference	US FDA (Centre for Drug Eval.& Res./Off. Testing & Res.) Sept. 1997	US FDA (Centre for Drug Eval.& Res./Off. Testing & Res.) Sept. 1997	US FDA (Centre for Drug Eval.& Res./Off. Testing & Res.) Sept. 1997

Model Applicability

Unknown features are fingerprint features in the query molecule, but not found or appearing too infrequently in the training set.

1. All properties and OPS components are within expected ranges.

Feature Contribution

Top features for positive contribution

Fingerprint	Bit/Smiles	Feature Structure	Score	Multiple-Carcinogen in training set
SCFP_4	136686699	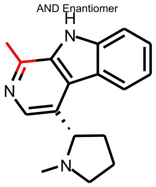 [*]:[c](:[*])C	0.391	7 out of 14

SCFP_4	149003983	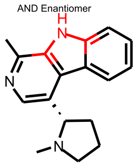 [*]:[c]1:[*]:[*]:[c](:[*]):[nH]:1	0.266	11 out of 26
SCFP_4	-496201075	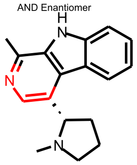 [*][c](:[*]):[cH]:n:[*]	0.257	6 out of 14

Top Features for negative contribution

Fingerprint	Bit/Smiles	Feature Structure	Score	Multiple-Carcinogen in training set
SCFP_4	-182915287	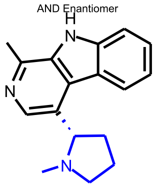 [*][C@@H]1CCCN1C	-1.42	0 out of 10
SCFP_4	622342378	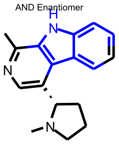 [*]:[c]1:[nH]:[c]2:[cH]:[cH]:[*]:[cH]:[c]:2:[c]:1:[*]	-0.816	0 out of 4
SCFP_4	1893791786	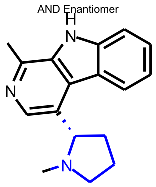 [*][C@@H]1CCCN1[*]	-0.816	0 out of 4

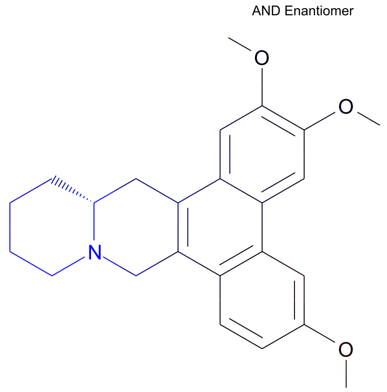

$C_{24}H_{27}NO_3$
Molecular Weight: 377.47608
ALogP: 4.691
Rotatable Bonds: 3
Acceptors: 4
Donors: 0

Structural Similar Compounds

Name	Ethynodiol	Risperidone	Clomipramine
Structure	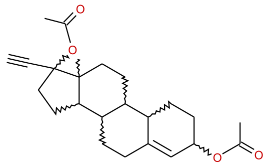	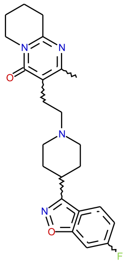	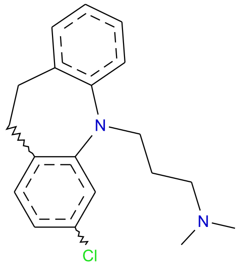
Actual Endpoint	Single-Carcinogen	Single-Carcinogen	Single-Carcinogen
Predicted Endpoint	Single-Carcinogen	Single-Carcinogen	Single-Carcinogen
Distance	0.570	0.609	0.657
Reference	US FDA (Centre for Drug Eval.& Res./Off. Testing & Res.) Sept. 1997	US FDA (Centre for Drug Eval.& Res./Off. Testing & Res.) Sept. 1997	US FDA (Centre for Drug Eval.& Res./Off. Testing & Res.) Sept. 1997

Model Prediction

Prediction: Single-Carcinogen
Probability: 0.0643
Enrichment: 0.172
Bayesian Score: -10.4
Mahalanobis Distance: 12.6
Mahalanobis Distance p-value: 0.000496

Prediction: Positive if the Bayesian score is above the estimated best cutoff value from minimizing the false positive and false negative rate.
Probability: The esimated probability that the sample is in the positive category. This assumes that the Bayesian score follows a normal distribution and is different from the prediction using a cutoff.
Enrichment: An estimate of enrichment, that is, the increased likelihood (versus random) of this sample being in the category.
Bayesian Score: The standard Laplacian-modified Bayesian score.
Mahalanobis Distance: The Mahalanobis distance (MD) is the distance to the center of the training data. The larger the MD, the less trustworthy the prediction.
Mahalanobis Distance p-value: The p-value gives the fraction of training data with an MD greater than or equal to the one for the given sample, assuming normally distributed data. The smaller the p-value, the less trustworthy the prediciton. For highly non-normal X properties (e.g., fingerprints), the MD p-value is wildly inaccurate.

Model Applicability

Unknown features are fingerprint features in the query molecule, but not found or appearing too infrequently in the training set.

1. All properties and OPS components are within expected ranges.

Feature Contribution

Top features for positive contribution

Fingerprint	Bit/Smiles	Feature Structure	Score	Multiple-Carcinogen in training set
SCFP_4	1237755852	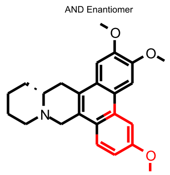 [*]:[c]1:[*]:[cH]:[cH]:[c](OC):[cH]:1	0.295	5 out of 11

SCFP_4	-1374800107	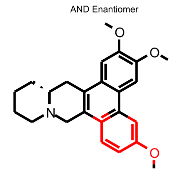 [*]O[c]1:[cH]:[*]:[c](:[*]):[cH]:[cH]:1	0.288	10 out of 23
SCFP_4	3	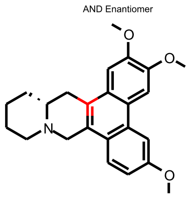 [*]:[cH]:[*]	0.199	36 out of 93
**Top Features for negative contribution**				
**Fingerprint**	**Bit/Smiles**	**Feature Structure**	**Score**	**Multiple-Carcinogen in training set**
SCFP_4	-182915287	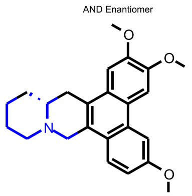 [*][C@@H]1CCCN1C	-1.42	0 out of 10
SCFP_4	1175638033	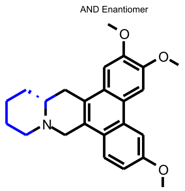 [*][C@@H]1[*]CCCC1	-1.17	0 out of 7
SCFP_4	-1272709286	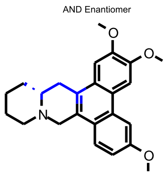 [*]C([*])C[c](:[*]):[*]	-1.16	1 out of 17

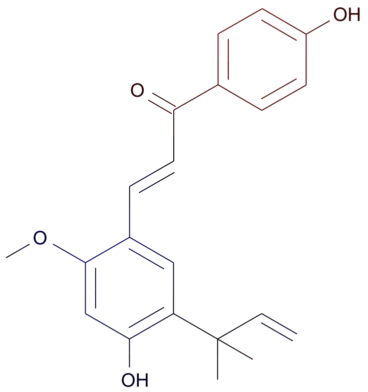

$C_{21}H_{22}O_4$
Molecular Weight: 338.39698
ALogP: 4.667
Rotatable Bonds: 6
Acceptors: 4
Donors: 2

Model Prediction

Prediction: Multiple-Carcinogen
Probability: 0.514
Enrichment: 1.37
Bayesian Score: 0.216
Mahalanobis Distance: 19.4
Mahalanobis Distance p-value: 2.1e-010

Prediction: Positive if the Bayesian score is above the estimated best cutoff value from minimizing the false positive and false negative rate.
Probability: The esimated probability that the sample is in the positive category. This assumes that the Bayesian score follows a normal distribution and is different from the prediction using a cutoff.
Enrichment: An estimate of enrichment, that is, the increased likelihood (versus random) of this sample being in the category.
Bayesian Score: The standard Laplacian-modified Bayesian score.
Mahalanobis Distance: The Mahalanobis distance (MD) is the distance to the center of the training data. The larger the MD, the less trustworthy the prediction.
Mahalanobis Distance p-value: The p-value gives the fraction of training data with an MD greater than or equal to the one for the given sample, assuming normally distributed data. The smaller the p-value, the less trustworthy the prediciton. For highly non-normal X properties (e.g., fingerprints), the MD p-value is wildly inaccurate.

Structural Similar Compounds

Name	Phenolphthalein	Diethylstilbesterol	Cytembena
Structure	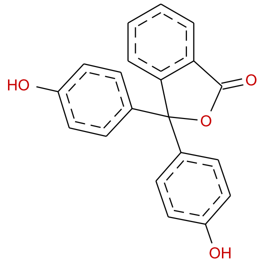	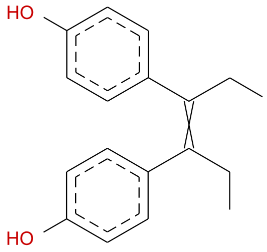	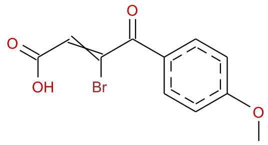
Actual Endpoint	Single-Carcinogen	Multiple-Carcinogen	Multiple-Carcinogen
Predicted Endpoint	Single-Carcinogen	Multiple-Carcinogen	Multiple-Carcinogen
Distance	0.605	0.624	0.651
Reference	US FDA (Centre for Drug Eval.& Res./Off. Testing & Res.) Sept. 1997	US FDA (Centre for Drug Eval.& Res./Off. Testing & Res.) Sept. 1997	US FDA (Centre for Drug Eval.& Res./Off. Testing & Res.) Sept. 1997

Model Applicability

Unknown features are fingerprint features in the query molecule, but not found or appearing too infrequently in the training set.

1. All properties and OPS components are within expected ranges.

Feature Contribution

Top features for positive contribution

Fingerprint	Bit/Smiles	Feature Structure	Score	Multiple-Carcinogen in training set
SCFP_4	611156666	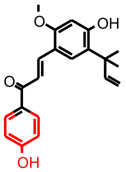 O[c]1:[cH]:[cH]:[*]:[cH]:[cH]:1	0.627	5 out of 7

SCFP_4	387787917	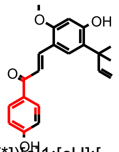 [*]C(=[*])[c]1:[cH]:[*]:[c]([*]):[cH]:[cH]:1	0.449	6 out of 11
SCFP_4	1327653783	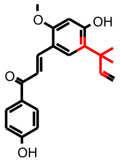 [*]=CC(C)(C)[c](:[*]):[*]	0.419	1 out of 1
**Top Features for negative contribution**				
**Fingerprint**	**Bit/Smiles**	**Feature Structure**	**Score**	**Multiple-Carcinogen in training set**
SCFP_4	-1931277081	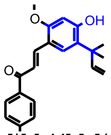 [*][c]1:[*]:[cH]:[c](:[c](O):[cH]:1)C([*])([*])[*]	-0.489	0 out of 2
SCFP_4	-1971137145	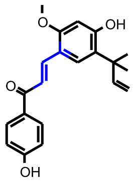 [*]\C=C\[c](:[*]):[*]	-0.489	0 out of 2
SCFP_4	130348166	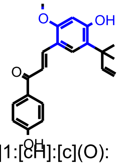 [*]O[c]1:[cH]:[c](O):[c]([*]):[*]:[c]:1[*]	-0.489	0 out of 2

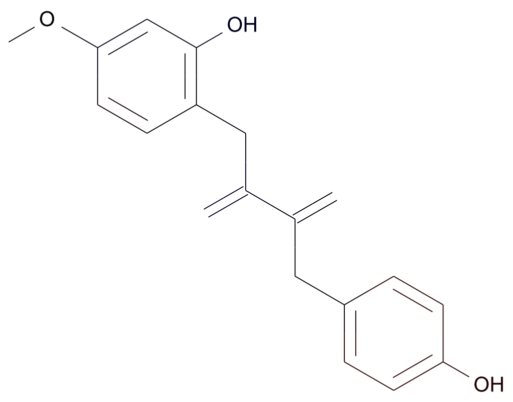

$C_{19}H_{20}O_3$
Molecular Weight: 296.3603
ALogP: 4.784
Rotatable Bonds: 6
Acceptors: 3
Donors: 2

Model Prediction

Prediction: Single-Carcinogen
Probability: 0.503
Enrichment: 1.35
Bayesian Score: -0.258
Mahalanobis Distance: 16.7
Mahalanobis Distance p-value: 7.49e-008

Prediction: Positive if the Bayesian score is above the estimated best cutoff value from minimizing the false positive and false negative rate.
Probability: The esimated probability that the sample is in the positive category. This assumes that the Bayesian score follows a normal distribution and is different from the prediction using a cutoff.
Enrichment: An estimate of enrichment, that is, the increased likelihood (versus random) of this sample being in the category.
Bayesian Score: The standard Laplacian-modified Bayesian score.
Mahalanobis Distance: The Mahalanobis distance (MD) is the distance to the center of the training data. The larger the MD, the less trustworthy the prediction.
Mahalanobis Distance p-value: The p-value gives the fraction of training data with an MD greater than or equal to the one for the given sample, assuming normally distributed data. The smaller the p-value, the less trustworthy the prediciton. For highly non-normal X properties (e.g., fingerprints), the MD p-value is wildly inaccurate.

Structural Similar Compounds

Name	Diethylstilbesterol	Dienestrol	Gemfibrozil
Structure	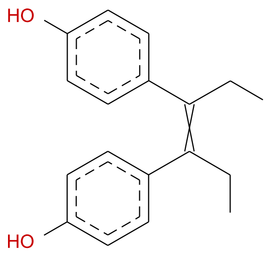	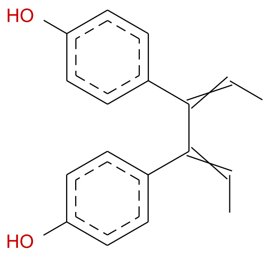	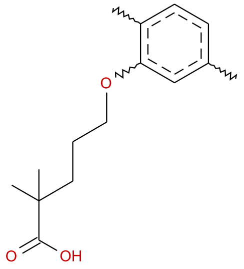
Actual Endpoint	Multiple-Carcinogen	Multiple-Carcinogen	Single-Carcinogen
Predicted Endpoint	Multiple-Carcinogen	Multiple-Carcinogen	Single-Carcinogen
Distance	0.526	0.571	0.599
Reference	US FDA (Centre for Drug Eval.& Res./Off. Testing & Res.) Sept. 1997	US FDA (Centre for Drug Eval.& Res./Off. Testing & Res.) Sept. 1997	US FDA (Centre for Drug Eval.& Res./Off. Testing & Res.) Sept. 1997

Model Applicability

Unknown features are fingerprint features in the query molecule, but not found or appearing too infrequently in the training set.

1. All properties and OPS components are within expected ranges.

Feature Contribution

Top features for positive contribution

Fingerprint	Bit/Smiles	Feature Structure	Score	Multiple-Carcinogen in training set
SCFP_4	611156666	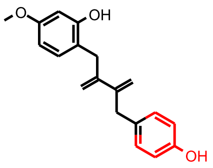 O[c]1:[cH]:[cH]:[*]:[cH]:[cH]:1	0.627	5 out of 7

SCFP_4	1237755852	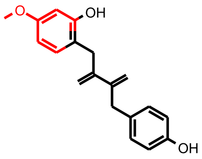 [*]:[c]1:[*]:[cH]:[cH]:[c](OC):[cH]:1	0.295	5 out of 11
SCFP_4	-1374800107	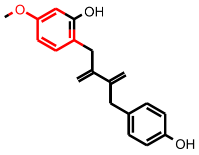 [*]O[c]1:[cH]:[*]:[c] (:[*]):[cH]:[cH]:1	0.288	10 out of 23
**Top Features for negative contribution**				
**Fingerprint**	**Bit/Smiles**	**Feature Structure**	**Score**	**Multiple-Carcinogen in training set**
SCFP_4	-1931277081	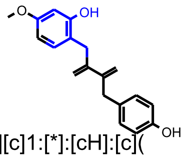 [*][c]1:[*]:[cH]:[c](:[c](O):[cH]:1)C([*])([*])[*]	-0.489	0 out of 2
SCFP_4	1872184315	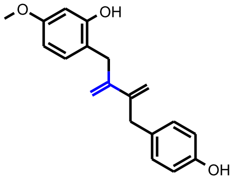 [*]C=C	-0.489	0 out of 2
SCFP_4	130348166	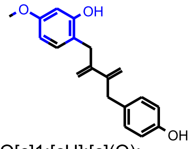 [*]O[c]1:[cH]:[c](O): [c]([*]):[*]:[c]:1[*]	-0.489	0 out of 2

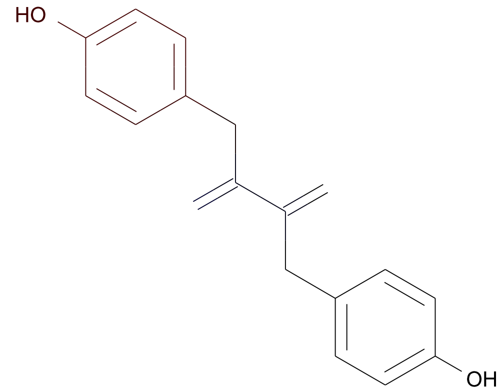

$C_{18}H_{18}O_2$
Molecular Weight: 266.33432
ALogP: 4.8
Rotatable Bonds: 5
Acceptors: 2
Donors: 2

Model Prediction

Prediction: Multiple-Carcinogen
Probability: 0.576
Enrichment: 1.54
Bayesian Score: 2.7
Mahalanobis Distance: 15.5
Mahalanobis Distance p-value: 1.07e-006

Prediction: Positive if the Bayesian score is above the estimated best cutoff value from minimizing the false positive and false negative rate.
Probability: The esimated probability that the sample is in the positive category. This assumes that the Bayesian score follows a normal distribution and is different from the prediction using a cutoff.
Enrichment: An estimate of enrichment, that is, the increased likelihood (versus random) of this sample being in the category.
Bayesian Score: The standard Laplacian-modified Bayesian score.
Mahalanobis Distance: The Mahalanobis distance (MD) is the distance to the center of the training data. The larger the MD, the less trustworthy the prediction.
Mahalanobis Distance p-value: The p-value gives the fraction of training data with an MD greater than or equal to the one for the given sample, assuming normally distributed data. The smaller the p-value, the less trustworthy the prediciton. For highly non-normal X properties (e.g., fingerprints), the MD p-value is wildly inaccurate.

Structural Similar Compounds

Name	Diethylstilbesterol	Dienestrol	Gemfibrozil
Structure	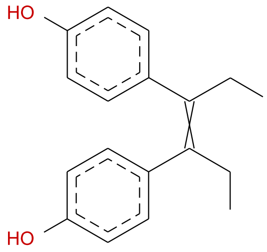	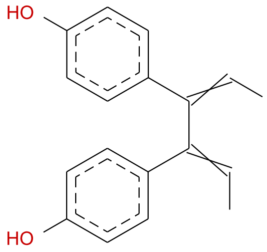	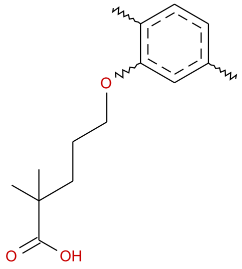
Actual Endpoint	Multiple-Carcinogen	Multiple-Carcinogen	Single-Carcinogen
Predicted Endpoint	Multiple-Carcinogen	Multiple-Carcinogen	Single-Carcinogen
Distance	0.387	0.439	0.616
Reference	US FDA (Centre for Drug Eval.& Res./Off. Testing & Res.) Sept. 1997	US FDA (Centre for Drug Eval.& Res./Off. Testing & Res.) Sept. 1997	US FDA (Centre for Drug Eval.& Res./Off. Testing & Res.) Sept. 1997

Model Applicability

Unknown features are fingerprint features in the query molecule, but not found or appearing too infrequently in the training set.

1. All properties and OPS components are within expected ranges.

Feature Contribution

Top features for positive contribution

Fingerprint	Bit/Smiles	Feature Structure	Score	Multiple-Carcinogen in training set
SCFP_4	611156666	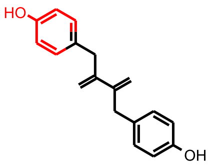 O[c]1:[cH]:[cH]:[*]:[cH]:[cH]:1	0.627	5 out of 7

SCFP_4	-1374800107	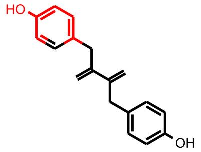 [*]O[c]1:[cH]:[*]:[c](:[*]):[cH]:[cH]:1	0.288	10 out of 23
SCFP_4	470101049	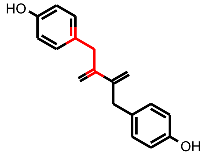 [*]C(=[*])C[c](:[*]):[*]	0.204	1 out of 2
**Top Features for negative contribution**				
**Fingerprint**	**Bit/Smiles**	**Feature Structure**	**Score**	**Multiple-Carcinogen in training set**
SCFP_4	1872184315	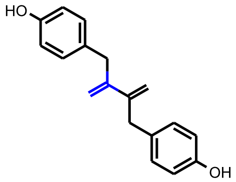 [*]C=C	-0.489	0 out of 2
SCFP_4	55464376	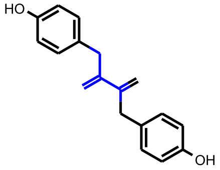 [*]CC(=C)C(=[*])[*]	-0.398	2 out of 11
SCFP_4	-496409612	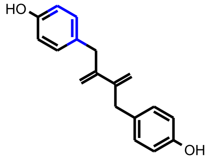 [*]:[cH]:[cH]:[cH]:[*]	0	27 out of 82

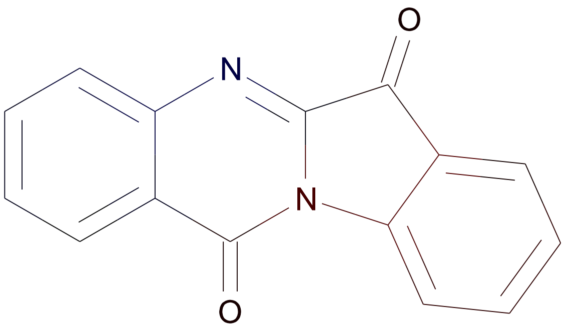

$C_{15}H_8N_2O_2$
Molecular Weight: 248.23621
ALogP: 2.331
Rotatable Bonds: 0
Acceptors: 3
Donors: 0

Model Prediction

Prediction: Multiple-Carcinogen
Probability: 0.528
Enrichment: 1.41
Bayesian Score: 1.13
Mahalanobis Distance: 11.6
Mahalanobis Distance p-value: 0.00401

Prediction: Positive if the Bayesian score is above the estimated best cutoff value from minimizing the false positive and false negative rate.
Probability: The esimated probability that the sample is in the positive category. This assumes that the Bayesian score follows a normal distribution and is different from the prediction using a cutoff.
Enrichment: An estimate of enrichment, that is, the increased likelihood (versus random) of this sample being in the category.
Bayesian Score: The standard Laplacian-modified Bayesian score.
Mahalanobis Distance: The Mahalanobis distance (MD) is the distance to the center of the training data. The larger the MD, the less trustworthy the prediction.
Mahalanobis Distance p-value: The p-value gives the fraction of training data with an MD greater than or equal to the one for the given sample, assuming normally distributed data. The smaller the p-value, the less trustworthy the prediciton. For highly non-normal X properties (e.g., fingerprints), the MD p-value is wildly inaccurate.

Structural Similar Compounds

Name	Methoxsalen; 8-	Carbamazepine	Granisetron
Structure	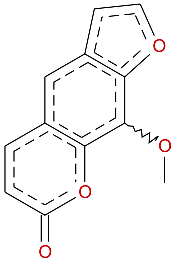	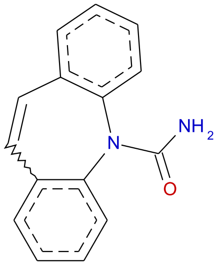	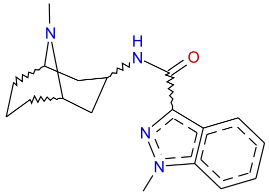
Actual Endpoint	Single-Carcinogen	Single-Carcinogen	Single-Carcinogen
Predicted Endpoint	Single-Carcinogen	Multiple-Carcinogen	Single-Carcinogen
Distance	0.530	0.625	0.686
Reference	US FDA (Centre for Drug Eval.& Res./Off. Testing & Res.) Sept. 1997	US FDA (Centre for Drug Eval.& Res./Off. Testing & Res.) Sept. 1997	US FDA (Centre for Drug Eval.& Res./Off. Testing & Res.) Sept. 1997

Model Applicability

Unknown features are fingerprint features in the query molecule, but not found or appearing too infrequently in the training set.

1. All properties and OPS components are within expected ranges.

Feature Contribution

Top features for positive contribution

Fingerprint	Bit/Smiles	Feature Structure	Score	Multiple-Carcinogen in training set
SCFP_4	-1375926917	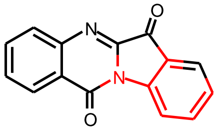 [*]N1[*][*][c]2:[*]:[cH]:[cH]:[cH]:[c]1:2	0.522	6 out of 10

SCFP_4	1205586762	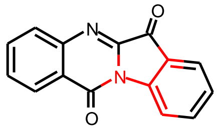 [*]N1[*][*][c](:[*]):[c]1:[cH]:[*]	0.451	7 out of 13
SCFP_4	387787917	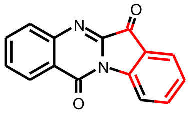 [*]C(=[*])[c]1:[cH]:[*]:[c]([*]):[cH]:[cH]:1	0.449	6 out of 11
**Top Features for negative contribution**				
**Fingerprint**	**Bit/Smiles**	**Feature Structure**	**Score**	**Multiple-Carcinogen in training set**
SCFP_4	1851000357	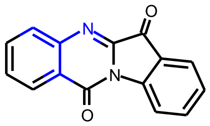 [*][c](:[*]):[c](:[cH]:[*])N=[*]	-0.489	0 out of 2
SCFP_4	-1377141613	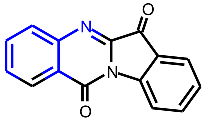 [*][c]1:[*]:[cH]:[cH]:[cH]:[c]:1N=[*]	-0.489	0 out of 2
SCFP_4	353445762	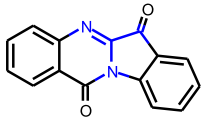 [*]\N=C\1/N([*])[*]:[*]C1=[*]	-0.274	0 out of 1

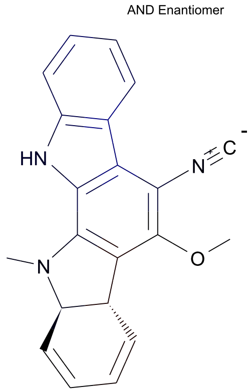

$C_{21}H_{17}N_3O$
Molecular Weight: 327.37918
ALogP: 4.078
Rotatable Bonds: 1
Acceptors: 2
Donors: 1

Structural Similar Compounds

Name	Mestranol	Norethindrone	Norethynodrel
Structure	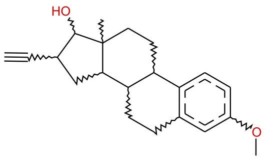	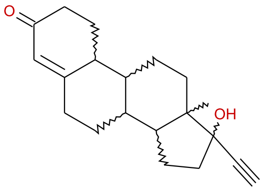	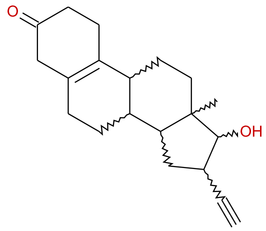
Actual Endpoint	Single-Carcinogen	Multiple-Carcinogen	Single-Carcinogen
Predicted Endpoint	Single-Carcinogen	Single-Carcinogen	Single-Carcinogen
Distance	0.567	0.569	0.575
Reference	US FDA (Centre for Drug Eval.& Res./Off. Testing & Res.) Sept. 1997	US FDA (Centre for Drug Eval.& Res./Off. Testing & Res.) Sept. 1997	US FDA (Centre for Drug Eval.& Res./Off. Testing & Res.) Sept. 1997

Model Prediction

Prediction: Single-Carcinogen
Probability: 0.448
Enrichment: 1.2
Bayesian Score: -2.04
Mahalanobis Distance: 20.8
Mahalanobis Distance p-value: 1.14e-011

Prediction: Positive if the Bayesian score is above the estimated best cutoff value from minimizing the false positive and false negative rate.
Probability: The esimated probability that the sample is in the positive category. This assumes that the Bayesian score follows a normal distribution and is different from the prediction using a cutoff.
Enrichment: An estimate of enrichment, that is, the increased likelihood (versus random) of this sample being in the category.
Bayesian Score: The standard Laplacian-modified Bayesian score.
Mahalanobis Distance: The Mahalanobis distance (MD) is the distance to the center of the training data. The larger the MD, the less trustworthy the prediction.
Mahalanobis Distance p-value: The p-value gives the fraction of training data with an MD greater than or equal to the one for the given sample, assuming normally distributed data. The smaller the p-value, the less trustworthy the prediciton. For highly non-normal X properties (e.g., fingerprints), the MD p-value is wildly inaccurate.

Model Applicability

Unknown features are fingerprint features in the query molecule, but not found or appearing too infrequently in the training set.

1. All properties and OPS components are within expected ranges.

Feature Contribution

Top features for positive contribution

Fingerprint	Bit/Smiles	Feature Structure	Score	Multiple-Carcinogen in training set
SCFP_4	699559848	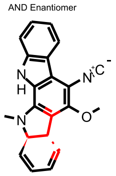 [*][C@H]1[*][*]:[c](:[*])[C@@H]1C=[*]	0.419	1 out of 1

SCFP_4	-1971196727	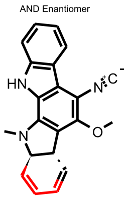 [*]\C=C\C(=[*])[*]	0.295	5 out of 11
SCFP_4	149003983	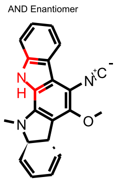 [*]:[c]1:[*]:[*]:[c](:[*]):[nH]:1	0.266	11 out of 26

Top Features for negative contribution

**Fingerprint**	**Bit/Smiles**	**Feature Structure**	**Score**	**Multiple-Carcinogen in training set**
SCFP_4	622342378	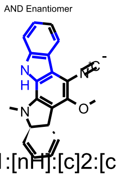 [*]:[c]1:[nH]:[c]2:[cH]:[cH]:[*]:[cH]:[c]:2:[c]:1:[*]	-0.816	0 out of 4
SCFP_4	112346096	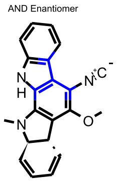 [*]:[cH]:[c]1:[c](:[*]):[*]:[*]:[c]:1:[*]	-0.73	1 out of 10
SCFP_4	-1853624961	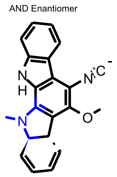 [*][C@@H]1[*][*]:[c](:[*])N1C	-0.651	1 out of 9

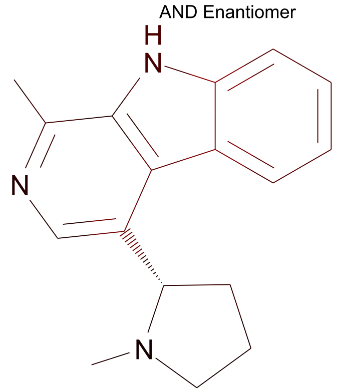

$C_{17}H_{19}N_3$

Molecular Weight: 265.35286

ALogP: 3.018

Rotatable Bonds: 1

Acceptors: 2

Donors: 1

Model Prediction

Prediction: Carcinogen

Probability: 0.515

Enrichment: 1.54

Bayesian Score: 4.75

Mahalanobis Distance: 12

Mahalanobis Distance p-value: 0.029

Prediction: Positive if the Bayesian score is above the estimated best cutoff value from minimizing the false positive and false negative rate.
Probability: The esimated probability that the sample is in the positive category. This assumes that the Bayesian score follows a normal distribution and is different from the prediction using a cutoff.
Enrichment: An estimate of enrichment, that is, the increased likelihood (versus random) of this sample being in the category.
Bayesian Score: The standard Laplacian-modified Bayesian score.
Mahalanobis Distance: The Mahalanobis distance (MD) is the distance to the center of the training data. The larger the MD, the less trustworthy the prediction.
Mahalanobis Distance p-value: The p-value gives the fraction of training data with an MD greater than or equal to the one for the given sample, assuming normally distributed data. The smaller the p-value, the less trustworthy the prediciton. For highly non-normal X properties (e.g., fingerprints), the MD p-value is wildly inaccurate.

Structural Similar Compounds

Name	Granisetron	Ondansetron	Nicotine
Structure	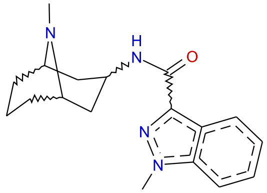	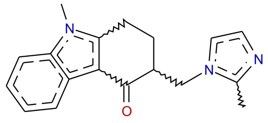	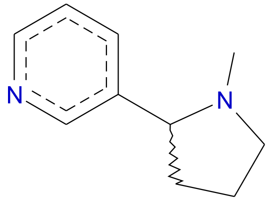
Actual Endpoint	Carcinogen	Non-Carcinogen	Carcinogen
Predicted Endpoint	Carcinogen	Non-Carcinogen	Carcinogen
Distance	0.572	0.577	0.611
Reference	US FDA (Centre for Drug Eval.& Res./Off. Testing & Res.) Sept. 1997	US FDA (Centre for Drug Eval.& Res./Off. Testing & Res.) Sept. 1997	US FDA (Centre for Drug Eval.& Res./Off. Testing & Res.) Sept. 1997

Model Applicability

Unknown features are fingerprint features in the query molecule, but not found or appearing too infrequently in the training set.

1. All properties and OPS components are within expected ranges.

Feature Contribution

Top features for positive contribution

Fingerprint	Bit/Smiles	Feature Structure	Score	Carcinogen in training set
SCFP_6	1651620003	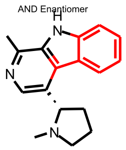 [*]:[c]1:[*]:[*]:[c]2:[cH]:[cH]:[cH]:[cH]:[c]:1:2	0.643	7 out of 10

SCFP_6	1172191254	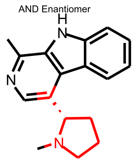 [*]N1CCC[C@H]1[c](:[*]):[*]	0.603	2 out of 2
SCFP_6	-1379673609	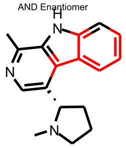 [*]:[c]1:[*]:[*]:[c]2:[*]:[cH]:[cH]:[cH]:[c]:1:2	0.526	11 out of 19
**Top Features for negative contribution**				
**Fingerprint**	**Bit/Smiles**	**Feature Structure**	**Score**	**Carcinogen in training set**
SCFP_6	1893791786	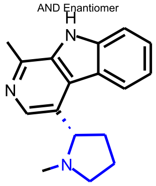 [*][C@@H]1CCCN1[*]	-0.766	2 out of 17
SCFP_6	403834996	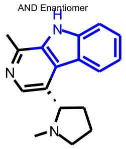 [*]:[c]1:[nH]:[c]2:[cH]:[cH]:[cH]:[cH]:[c]:2:[c]:1:[*]	-0.264	1 out of 5
SCFP_6	2088704928	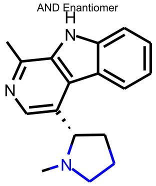 [*]N1[*][*]CC1	-0.233	29 out of 115

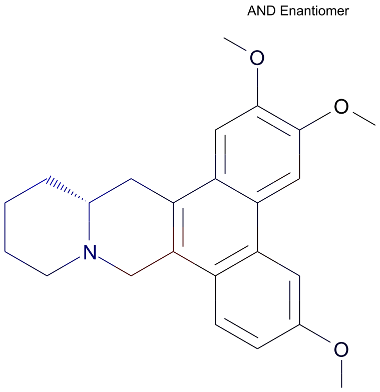

$C_{24}H_{27}NO_3$
Molecular Weight: 377.47608
ALogP: 4.691
Rotatable Bonds: 3
Acceptors: 4
Donors: 0

Model Prediction

Prediction: Non-Carcinogen
Probability: 0.26
Enrichment: 0.777
Bayesian Score: -3.92
Mahalanobis Distance: 13.7
Mahalanobis Distance p-value: 0.000117

Prediction: Positive if the Bayesian score is above the estimated best cutoff value from minimizing the false positive and false negative rate.
Probability: The esimated probability that the sample is in the positive category. This assumes that the Bayesian score follows a normal distribution and is different from the prediction using a cutoff.
Enrichment: An estimate of enrichment, that is, the increased likelihood (versus random) of this sample being in the category.
Bayesian Score: The standard Laplacian-modified Bayesian score.
Mahalanobis Distance: The Mahalanobis distance (MD) is the distance to the center of the training data. The larger the MD, the less trustworthy the prediction.
Mahalanobis Distance p-value: The p-value gives the fraction of training data with an MD greater than or equal to the one for the given sample, assuming normally distributed data. The smaller the p-value, the less trustworthy the prediciton. For highly non-normal X properties (e.g., fingerprints), the MD p-value is wildly inaccurate.

Structural Similar Compounds

Name	Ethynodiol	Loratidine	Chlorpromazine
Structure	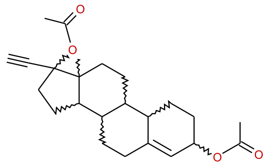	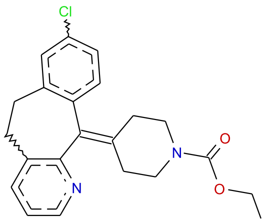	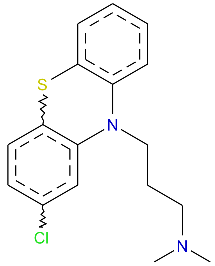
Actual Endpoint	Carcinogen	Non-Carcinogen	Carcinogen
Predicted Endpoint	Carcinogen	Non-Carcinogen	Carcinogen
Distance	0.581	0.591	0.608
Reference	US FDA (Centre for Drug Eval.& Res./Off. Testing & Res.) Sept. 1997	US FDA (Centre for Drug Eval.& Res./Off. Testing & Res.) Sept. 1997	US FDA (Centre for Drug Eval.& Res./Off. Testing & Res.) Sept. 1997

Model Applicability

Unknown features are fingerprint features in the query molecule, but not found or appearing too infrequently in the training set.

1. All properties and OPS components are within expected ranges.

Feature Contribution

Top features for positive contribution

Fingerprint	Bit/Smiles	Feature Structure	Score	Carcinogen in training set
SCFP_6	1453622480	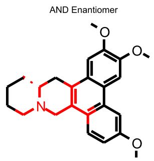 [*]CN1C[c](:[c](:[*]):[*]):[c](:[*])[*][C@H]1[*]	0.561	3 out of 4

SCFP_6	-1379673609	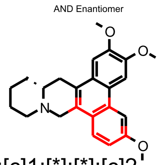 [*]:[c]1:[*]:[*]:[c]2 :[*]:[cH]:[cH]:[cH]: [c]:1:2	0.526	11 out of 19
SCFP_6	2088794301	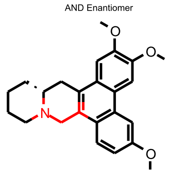 [*]N([*])C[c](:[*]):[*]	0.432	4 out of 7
**Top Features for negative contribution**				
**Fingerprint**	**Bit/Smiles**	**Feature Structure**	**Score**	**Carcinogen in training set**
SCFP_6	-1852113435	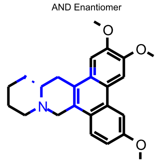 [*]C[C@@H]1C[c](:[c](:[*]):[*]):[c](:[*]) [*]N1[*]	-0.825	0 out of 4
SCFP_6	-576781090	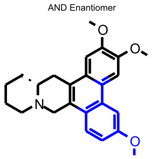 [*]:[c](:[*]):[c]1:[c H]:[c](OC):[cH]:[cH] :[c]:1:[*]	-0.825	0 out of 4
SCFP_6	1175638033	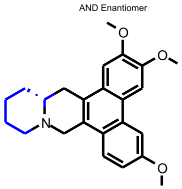 [*][C@@H]1[*]CCCC1	-0.812	4 out of 32

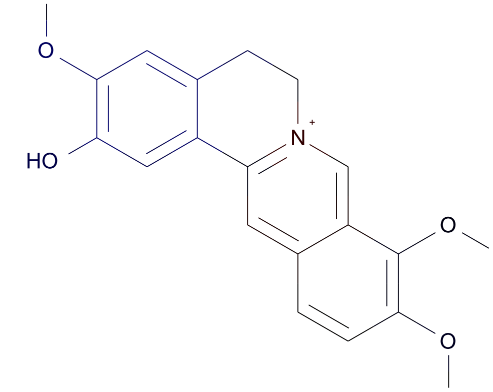

$C_{20}H_{20}NO_4$
Molecular Weight: 338.3771
ALogP: 3.936
Rotatable Bonds: 3
Acceptors: 4
Donors: 1

Model Prediction

Prediction: Non-Carcinogen
Probability: 0.276
Enrichment: 0.825
Bayesian Score: -3.17
Mahalanobis Distance: 14.4
Mahalanobis Distance p-value: 7.56e-006

Prediction: Positive if the Bayesian score is above the estimated best cutoff value from minimizing the false positive and false negative rate.
Probability: The esimated probability that the sample is in the positive category. This assumes that the Bayesian score follows a normal distribution and is different from the prediction using a cutoff.
Enrichment: An estimate of enrichment, that is, the increased likelihood (versus random) of this sample being in the category.
Bayesian Score: The standard Laplacian-modified Bayesian score.
Mahalanobis Distance: The Mahalanobis distance (MD) is the distance to the center of the training data. The larger the MD, the less trustworthy the prediction.
Mahalanobis Distance p-value: The p-value gives the fraction of training data with an MD greater than or equal to the one for the given sample, assuming normally distributed data. The smaller the p-value, the less trustworthy the prediciton. For highly non-normal X properties (e.g., fingerprints), the MD p-value is wildly inaccurate.

Structural Similar Compounds

Name	Indomethacin	Paroxetine	Quinine
Structure	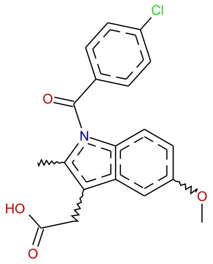	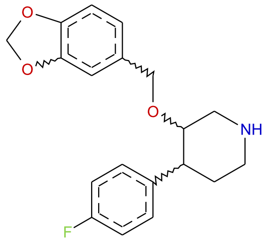	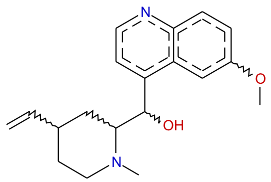
Actual Endpoint	Non-Carcinogen	Non-Carcinogen	Non-Carcinogen
Predicted Endpoint	Non-Carcinogen	Non-Carcinogen	Non-Carcinogen
Distance	0.534	0.558	0.566
Reference	US FDA (Centre for Drug Eval.& Res./Off. Testing & Res.) Sept. 1997	US FDA (Centre for Drug Eval.& Res./Off. Testing & Res.) Sept. 1997	US FDA (Centre for Drug Eval.& Res./Off. Testing & Res.) Sept. 1997

Model Applicability

Unknown features are fingerprint features in the query molecule, but not found or appearing too infrequently in the training set.

1. All properties and OPS components are within expected ranges.

Feature Contribution

Top features for positive contribution

Fingerprint	Bit/Smiles	Feature Structure	Score	Carcinogen in training set
SCFP_6	-1379673609	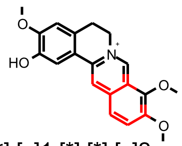 [*]:[c]1:[*]:[*]:[c]2 :[*]:[cH]:[cH]:[cH]: [c]:1:2	0.526	11 out of 19

SCFP_6	-1798344807	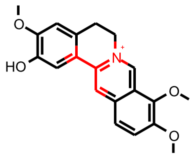<br>[*][n+](:[*]):[c](:[c<br>H]:[*])[c](:[*]):[*]	0.313	3 out of 6
SCFP_6	112346096	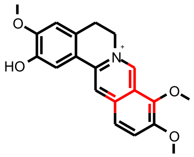<br>[*]:[cH]:[c]1:[c](:[*<br>]):[*]:[*]:[c]:1:[*]	0.276	13 out of 30
**Top Features for negative contribution**				
**Fingerprint**	**Bit/Smiles**	**Feature Structure**	**Score**	**Carcinogen in training set**
SCFP_6	2116304939	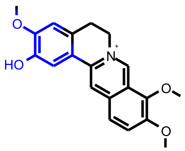<br>[*]O[c]1:[cH]:[*]:[c]<br>([*]):[cH]:[c]:1O	-0.825	0 out of 4
SCFP_6	125999298	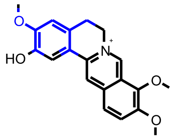<br>[*]C[c]1:[cH]:[c](O[*<br>]):[c]([*]):[*]:[c]:<br>1[*]	-0.7	3 out of 22
SCFP_6	-1889730273	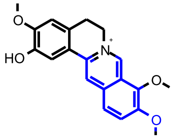<br>[*]O[c]1:[cH]:[cH]:[c<br>]2:[cH]:[c]([*]):[*]<br>:[cH]:[c]:2:[c]:1[*]	-0.496	0 out of 2

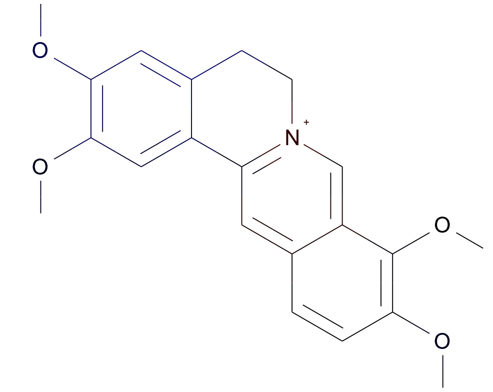

$C_{21}H_{22}NO_4$
Molecular Weight: 352.40368
ALogP: 4.161
Rotatable Bonds: 4
Acceptors: 4
Donors: 0

Model Prediction

Prediction: Non-Carcinogen
Probability: 0.321
Enrichment: 0.962
Bayesian Score: -1.27
Mahalanobis Distance: 13.9
Mahalanobis Distance p-value: 6.43e-005

Prediction: Positive if the Bayesian score is above the estimated best cutoff value from minimizing the false positive and false negative rate.
Probability: The esimated probability that the sample is in the positive category. This assumes that the Bayesian score follows a normal distribution and is different from the prediction using a cutoff.
Enrichment: An estimate of enrichment, that is, the increased likelihood (versus random) of this sample being in the category.
Bayesian Score: The standard Laplacian-modified Bayesian score.
Mahalanobis Distance: The Mahalanobis distance (MD) is the distance to the center of the training data. The larger the MD, the less trustworthy the prediction.
Mahalanobis Distance p-value: The p-value gives the fraction of training data with an MD greater than or equal to the one for the given sample, assuming normally distributed data. The smaller the p-value, the less trustworthy the prediciton. For highly non-normal X properties (e.g., fingerprints), the MD p-value is wildly inaccurate.

Structural Similar Compounds

Name	Ethynodiol	Chlorpromazine	Indomethacin
Structure	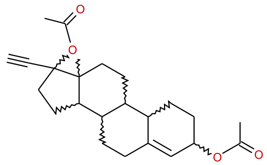	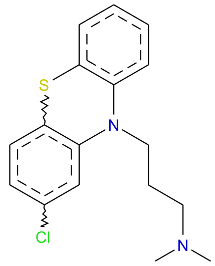	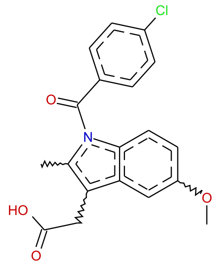
Actual Endpoint	Carcinogen	Carcinogen	Non-Carcinogen
Predicted Endpoint	Carcinogen	Carcinogen	Non-Carcinogen
Distance	0.590	0.604	0.614
Reference	US FDA (Centre for Drug Eval.& Res./Off. Testing & Res.) Sept. 1997	US FDA (Centre for Drug Eval.& Res./Off. Testing & Res.) Sept. 1997	US FDA (Centre for Drug Eval.& Res./Off. Testing & Res.) Sept. 1997

Model Applicability

Unknown features are fingerprint features in the query molecule, but not found or appearing too infrequently in the training set.

1. All properties and OPS components are within expected ranges.

Feature Contribution

Top features for positive contribution

Fingerprint	Bit/Smiles	Feature Structure	Score	Carcinogen in training set
SCFP_6	-1379673609	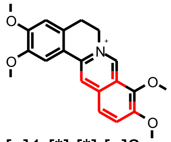 [*]:[c]1:[*]:[*]:[c]2:[*]:[cH]:[cH]:[cH]:[c]:1:2	0.526	11 out of 19

SCFP_6	-1798344807	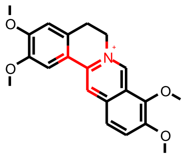 [*][n+](:[*]):[c](:[cH]:[*])[c](:[*]):[*]	0.313	3 out of 6
SCFP_6	112346096	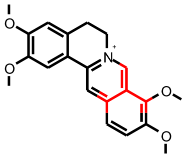 [*]:[cH]:[c]1:[c](:[*]):[*]:[*]:[c]:1:[*]	0.276	13 out of 30

Top Features for negative contribution

Fingerprint	Bit/Smiles	Feature Structure	Score	Carcinogen in training set
SCFP_6	125999298	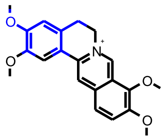 [*]C[c]1:[cH]:[c](O[*]):[c]([*]):[*]:[c]:1[*]	-0.7	3 out of 22
SCFP_6	-1889730273	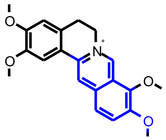 [*]O[c]1:[cH]:[cH]:[c]2:[cH]:[c]([*]):[*]:[cH]:[c]:2:[c]:1[*]	-0.496	0 out of 2
SCFP_6	-375917587	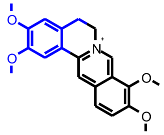 [*]C[c]1:[cH]:[c](OC):[c](OC):[cH]:[c]:1[*]	-0.496	0 out of 2

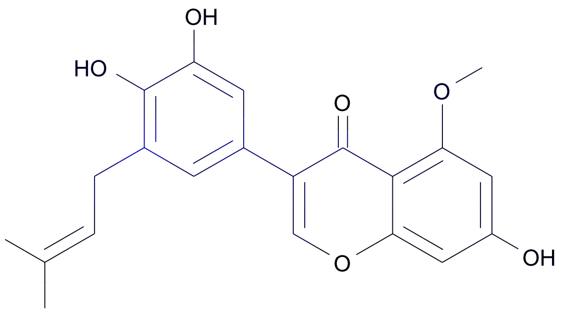

$C_{21}H_{20}O_6$
Molecular Weight: 368.3799
ALogP: 3.98
Rotatable Bonds: 4
Acceptors: 6
Donors: 3

Model Prediction

Prediction: Non-Carcinogen
Probability: 0.217
Enrichment: 0.65
Bayesian Score: -6.22
Mahalanobis Distance: 13.6
Mahalanobis Distance p-value: 0.00022

Prediction: Positive if the Bayesian score is above the estimated best cutoff value from minimizing the false positive and false negative rate.
Probability: The esimated probability that the sample is in the positive category. This assumes that the Bayesian score follows a normal distribution and is different from the prediction using a cutoff.
Enrichment: An estimate of enrichment, that is, the increased likelihood (versus random) of this sample being in the category.
Bayesian Score: The standard Laplacian-modified Bayesian score.
Mahalanobis Distance: The Mahalanobis distance (MD) is the distance to the center of the training data. The larger the MD, the less trustworthy the prediction.
Mahalanobis Distance p-value: The p-value gives the fraction of training data with an MD greater than or equal to the one for the given sample, assuming normally distributed data. The smaller the p-value, the less trustworthy the prediciton. For highly non-normal X properties (e.g., fingerprints), the MD p-value is wildly inaccurate.

Structural Similar Compounds

Name	Torsemide	Ursodiol	Clorazepate
Structure	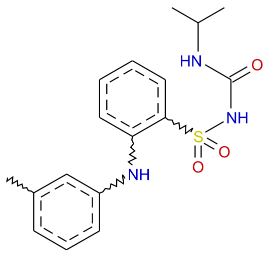	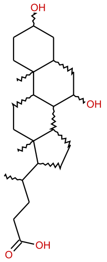	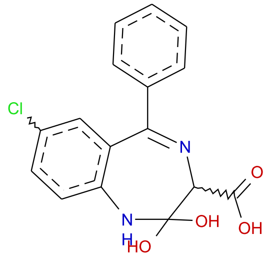
Actual Endpoint	Carcinogen	Non-Carcinogen	Non-Carcinogen
Predicted Endpoint	Carcinogen	Non-Carcinogen	Non-Carcinogen
Distance	0.666	0.675	0.691
Reference	US FDA (Centre for Drug Eval.& Res./Off. Testing & Res.) Sept. 1997	US FDA (Centre for Drug Eval.& Res./Off. Testing & Res.) Sept. 1997	US FDA (Centre for Drug Eval.& Res./Off. Testing & Res.) Sept. 1997

Model Applicability

Unknown features are fingerprint features in the query molecule, but not found or appearing too infrequently in the training set.

1. All properties and OPS components are within expected ranges.

Feature Contribution

Top features for positive contribution				
**Fingerprint**	**Bit/Smiles**	**Feature Structure**	**Score**	**Carcinogen in training set**
SCFP_6	392579710	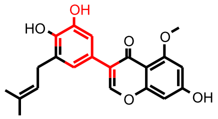 [*]C(=[*])[c]1:[cH]:[*]:[c]([*]):[c](O):[cH]:1	0.425	2 out of 3

SCFP_6	55434585	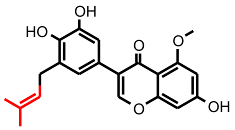 [*]C=C(C)C	0.331	12 out of 26
SCFP_6	1157879834	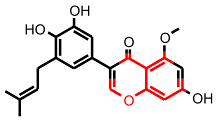 [*][c]1:[*]:[c]([*]):[c]2C(=[*])[*]=CO[c]:2:[cH]:1	0.198	1 out of 2
**Top Features for negative contribution**				
**Fingerprint**	**Bit/Smiles**	**Feature Structure**	**Score**	**Carcinogen in training set**
SCFP_6	2116304939	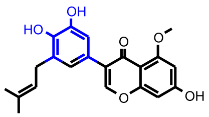 [*]O[c]1:[cH]:[*]:[c]([*]):[cH]:[c]:1O	-0.825	0 out of 4
SCFP_6	388230842	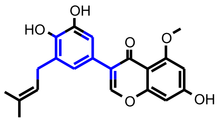 [*]C[c]1:[cH]:[c](:[cH]:[*]:[c]:1[*])C(=[*])[*]	-0.674	0 out of 3
SCFP_6	74606309	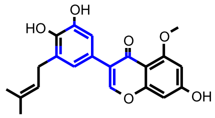 [*]\C=C(/C(=[*])[*])\[c]1:[cH]:[c]([*]):[*]:[c]([*]):[cH]:1	-0.674	0 out of 3

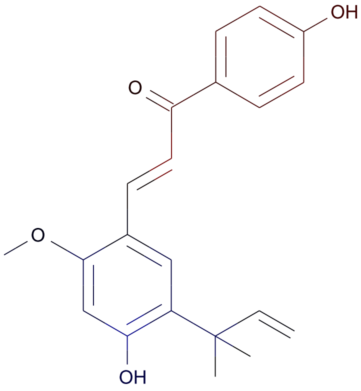

$C_{21}H_{22}O_4$
Molecular Weight: 338.39698
ALogP: 4.667
Rotatable Bonds: 6
Acceptors: 4
Donors: 2

Model Prediction

Prediction: Non-Carcinogen
Probability: 0.334
Enrichment: 0.999
Bayesian Score: -0.805
Mahalanobis Distance: 13.5
Mahalanobis Distance p-value: 0.000323

Prediction: Positive if the Bayesian score is above the estimated best cutoff value from minimizing the false positive and false negative rate.
Probability: The esimated probability that the sample is in the positive category. This assumes that the Bayesian score follows a normal distribution and is different from the prediction using a cutoff.
Enrichment: An estimate of enrichment, that is, the increased likelihood (versus random) of this sample being in the category.
Bayesian Score: The standard Laplacian-modified Bayesian score.
Mahalanobis Distance: The Mahalanobis distance (MD) is the distance to the center of the training data. The larger the MD, the less trustworthy the prediction.
Mahalanobis Distance p-value: The p-value gives the fraction of training data with an MD greater than or equal to the one for the given sample, assuming normally distributed data. The smaller the p-value, the less trustworthy the prediciton. For highly non-normal X properties (e.g., fingerprints), the MD p-value is wildly inaccurate.

Structural Similar Compounds

Name	Ethacrynic acid	Indomethacin	Diclofenac
Structure	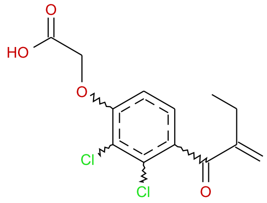	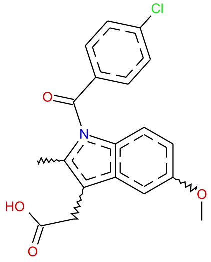	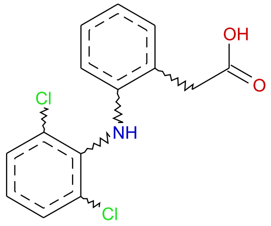
Actual Endpoint	Non-Carcinogen	Non-Carcinogen	Non-Carcinogen
Predicted Endpoint	Non-Carcinogen	Non-Carcinogen	Non-Carcinogen
Distance	0.576	0.612	0.621
Reference	US FDA (Centre for Drug Eval.& Res./Off. Testing & Res.) Sept. 1997	US FDA (Centre for Drug Eval.& Res./Off. Testing & Res.) Sept. 1997	US FDA (Centre for Drug Eval.& Res./Off. Testing & Res.) Sept. 1997

Model Applicability

Unknown features are fingerprint features in the query molecule, but not found or appearing too infrequently in the training set.

1. All properties and OPS components are within expected ranges.

Feature Contribution

Top features for positive contribution				
**Fingerprint**	**Bit/Smiles**	**Feature Structure**	**Score**	**Carcinogen in training set**
SCFP_6	966282057	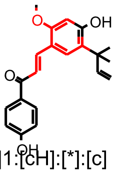 [*]O[c]1:[cH]:[*]:[c]([*]):[cH]:[c]:1\C=C\[*]	0.603	2 out of 2

SCFP_6	1958008606	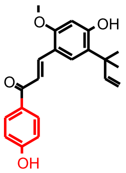 [*][c]1:[cH]:[cH]:[c](O):[cH]:[cH]:1	0.536	4 out of 6
SCFP_6	-1971137145	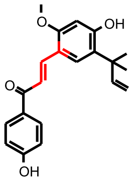 [*]\C=C\[c](:[*]):[*]	0.434	5 out of 9

Top Features for negative contribution

Fingerprint	Bit/Smiles	Feature Structure	Score	Carcinogen in training set
SCFP_6	-1931277081	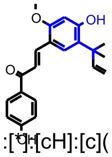 [*][c]1:[*]:[cH]:[c](:[c](O):[cH]:1)C([*])([*])[*]	-0.674	0 out of 3
SCFP_6	388230842	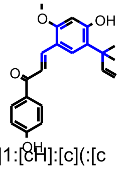 [*]C[c]1:[cH]:[c](:[cH]:[*]:[c]:1[*])C(=[*])[*]	-0.674	0 out of 3
SCFP_6	503541685	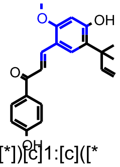 [*]C(=[*])[c]1:[c]([*]):[*]:[c]([*]):[cH]:[c]:1OC	-0.484	1 out of 7

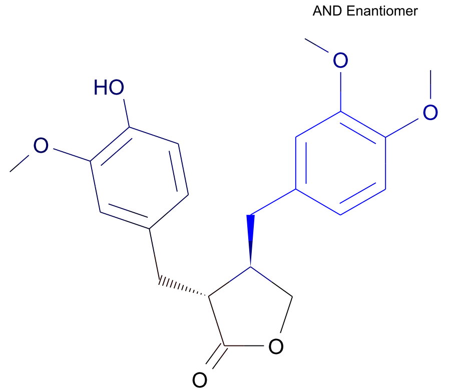

$C_{21}H_{24}O_6$

Molecular Weight: 372.41166

ALogP: 3.743

Rotatable Bonds: 7

Acceptors: 6

Donors: 1

Model Prediction

Prediction: Non-Carcinogen

Probability: 0.177

Enrichment: 0.529

Bayesian Score: -9.09

Mahalanobis Distance: 14.3

Mahalanobis Distance p-value: 1.45e-005

Prediction: Positive if the Bayesian score is above the estimated best cutoff value from minimizing the false positive and false negative rate.
Probability: The esimated probability that the sample is in the positive category. This assumes that the Bayesian score follows a normal distribution and is different from the prediction using a cutoff.
Enrichment: An estimate of enrichment, that is, the increased likelihood (versus random) of this sample being in the category.
Bayesian Score: The standard Laplacian-modified Bayesian score.
Mahalanobis Distance: The Mahalanobis distance (MD) is the distance to the center of the training data. The larger the MD, the less trustworthy the prediction.
Mahalanobis Distance p-value: The p-value gives the fraction of training data with an MD greater than or equal to the one for the given sample, assuming normally distributed data. The smaller the p-value, the less trustworthy the prediciton. For highly non-normal X properties (e.g., fingerprints), the MD p-value is wildly inaccurate.

Structural Similar Compounds

Name	Lovastatin	Felodipine	Diltiazem
Structure	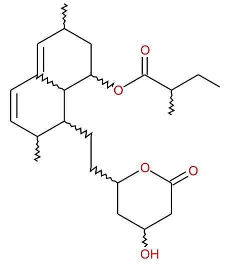	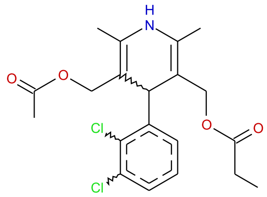	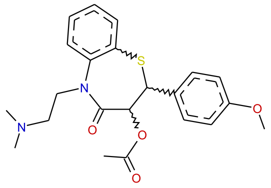
Actual Endpoint	Carcinogen	Carcinogen	Non-Carcinogen
Predicted Endpoint	Carcinogen	Carcinogen	Non-Carcinogen
Distance	0.565	0.575	0.589
Reference	US FDA (Centre for Drug Eval.& Res./Off. Testing & Res.) Sept. 1997	US FDA (Centre for Drug Eval.& Res./Off. Testing & Res.) Sept. 1997	US FDA (Centre for Drug Eval.& Res./Off. Testing & Res.) Sept. 1997

Model Applicability

Unknown features are fingerprint features in the query molecule, but not found or appearing too infrequently in the training set.

1. All properties and OPS components are within expected ranges.

Feature Contribution

Top features for positive contribution

Fingerprint	Bit/Smiles	Feature Structure	Score	Carcinogen in training set
SCFP_6	-1104485307	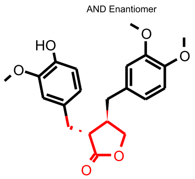 [*]C[C@@H]1[C@@H]([*])COC1=O	0.615	5 out of 7

SCFP_6	-1848330701	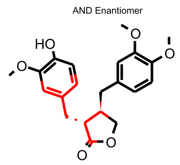<br>[*][C@H]1[*][*]C(=[*])[C@@H]1C[c](:[cH]:[*]):[cH]:[*]	0.415	1 out of 1
SCFP_6	-1043310069	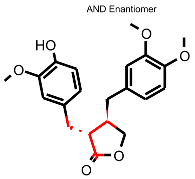<br>[*]C[C@@H]1[C@@H]([*])[*][*]C1=[*]	0.296	15 out of 34

Top Features for negative contribution

Fingerprint	Bit/Smiles	Feature Structure	Score	Carcinogen in training set
SCFP_6	-1642341584	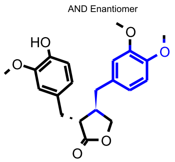<br>[*]O[c]1:[cH]:[cH]:[c](CC([*])[*]):[cH]:[c]:1[*]	-1.27	0 out of 8
SCFP_6	-1211866396	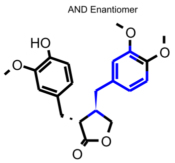<br>[*]C([*])C[c]1:[cH]:[cH]:[*]:[c]([*]):[cH]:1	-1.1	2 out of 25
SCFP_6	2116304939	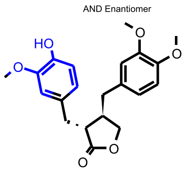<br>[*]O[c]1:[cH]:[*]:[c]([*]):[cH]:[c]:1O	-0.825	0 out of 4

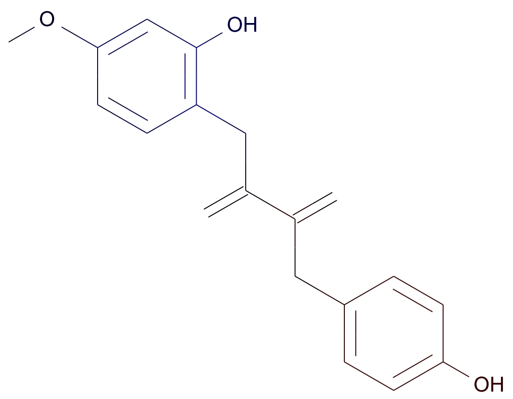

$C_{19}H_{20}O_3$
Molecular Weight: 296.3603
ALogP: 4.784
Rotatable Bonds: 6
Acceptors: 3
Donors: 2

Model Prediction

Prediction: Non-Carcinogen
Probability: 0.308
Enrichment: 0.921
Bayesian Score: -1.8
Mahalanobis Distance: 12.3
Mahalanobis Distance p-value: 0.0119

Prediction: Positive if the Bayesian score is above the estimated best cutoff value from minimizing the false positive and false negative rate.
Probability: The esimated probability that the sample is in the positive category. This assumes that the Bayesian score follows a normal distribution and is different from the prediction using a cutoff.
Enrichment: An estimate of enrichment, that is, the increased likelihood (versus random) of this sample being in the category.
Bayesian Score: The standard Laplacian-modified Bayesian score.
Mahalanobis Distance: The Mahalanobis distance (MD) is the distance to the center of the training data. The larger the MD, the less trustworthy the prediction.
Mahalanobis Distance p-value: The p-value gives the fraction of training data with an MD greater than or equal to the one for the given sample, assuming normally distributed data. The smaller the p-value, the less trustworthy the prediciton. For highly non-normal X properties (e.g., fingerprints), the MD p-value is wildly inaccurate.

Structural Similar Compounds

Name	Diethylstilbesterol	Penbutalol	Diclofenac
Structure	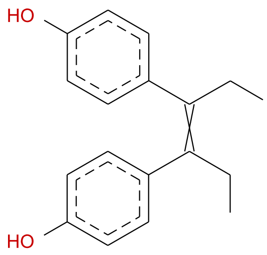	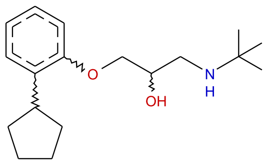	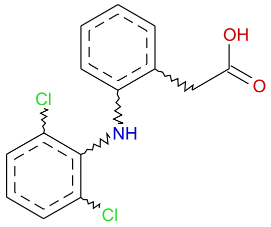
Actual Endpoint	Carcinogen	Non-Carcinogen	Non-Carcinogen
Predicted Endpoint	Carcinogen	Non-Carcinogen	Non-Carcinogen
Distance	0.558	0.573	0.573
Reference	US FDA (Centre for Drug Eval.& Res./Off. Testing & Res.) Sept. 1997	US FDA (Centre for Drug Eval.& Res./Off. Testing & Res.) Sept. 1997	US FDA (Centre for Drug Eval.& Res./Off. Testing & Res.) Sept. 1997

Model Applicability

Unknown features are fingerprint features in the query molecule, but not found or appearing too infrequently in the training set.

1. All properties and OPS components are within expected ranges.

Feature Contribution

Top features for positive contribution

Fingerprint	Bit/Smiles	Feature Structure	Score	Carcinogen in training set
SCFP_6	1958008606	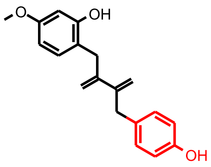 [*][c]1:[cH]:[cH]:[c](O):[cH]:[cH]:1	0.536	4 out of 6

SCFP_6	55464376	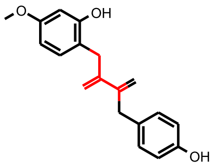 [*]CC(=C)C(=[*])[*]	0.345	14 out of 30
SCFP_6	1578073306	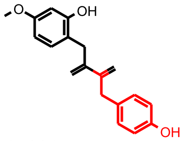 [*]C(=[*])C[c]1:[cH]:[cH]:[c](O):[cH]:[cH]:1	0.198	1 out of 2
**Top Features for negative contribution**				
**Fingerprint**	**Bit/Smiles**	**Feature Structure**	**Score**	**Carcinogen in training set**
SCFP_6	-1931277081	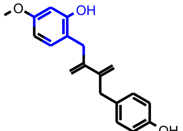 [*][c]1:[*]:[cH]:[c](:[c](O):[cH]:1)C([*])([*])[*]	-0.674	0 out of 3
SCFP_6	130348166	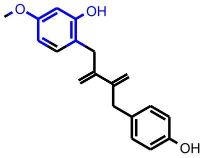 [*]O[c]1:[cH]:[c](O):[cH]:[*]:[c]:1[*]	-0.264	1 out of 5
SCFP_6	1872184315	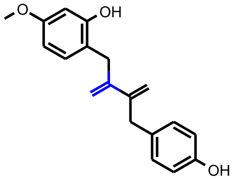 [*]C=C	-0.264	1 out of 5

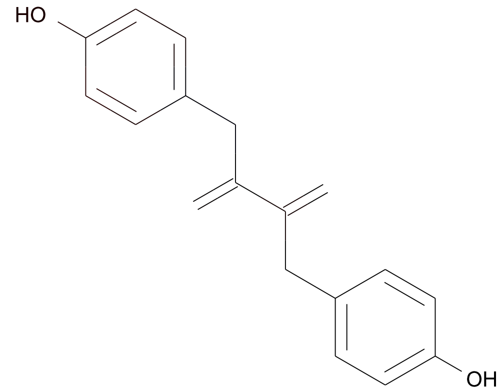

$C_{18}H_{18}O_2$
Molecular Weight: 266.33432
ALogP: 4.8
Rotatable Bonds: 5
Acceptors: 2
Donors: 2

Model Prediction

Prediction: Carcinogen
Probability: 0.351
Enrichment: 1.05
Bayesian Score: -0.195
Mahalanobis Distance: 10.3
Mahalanobis Distance p-value: 0.489

Prediction: Positive if the Bayesian score is above the estimated best cutoff value from minimizing the false positive and false negative rate.
Probability: The esimated probability that the sample is in the positive category. This assumes that the Bayesian score follows a normal distribution and is different from the prediction using a cutoff.
Enrichment: An estimate of enrichment, that is, the increased likelihood (versus random) of this sample being in the category.
Bayesian Score: The standard Laplacian-modified Bayesian score.
Mahalanobis Distance: The Mahalanobis distance (MD) is the distance to the center of the training data. The larger the MD, the less trustworthy the prediction.
Mahalanobis Distance p-value: The p-value gives the fraction of training data with an MD greater than or equal to the one for the given sample, assuming normally distributed data. The smaller the p-value, the less trustworthy the prediciton. For highly non-normal X properties (e.g., fingerprints), the MD p-value is wildly inaccurate.

Structural Similar Compounds

Name	Diethylstilbesterol	Dienestrol	Hexylresorcinol
Structure	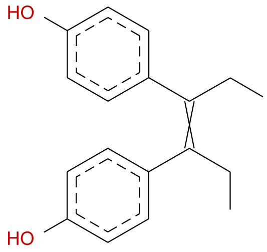	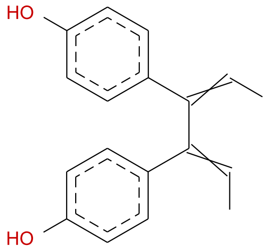	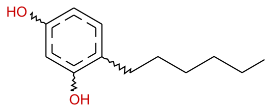
Actual Endpoint	Carcinogen	Non-Carcinogen	Non-Carcinogen
Predicted Endpoint	Carcinogen	Non-Carcinogen	Non-Carcinogen
Distance	0.426	0.479	0.518
Reference	US FDA (Centre for Drug Eval.& Res./Off. Testing & Res.) Sept. 1997	US FDA (Centre for Drug Eval.& Res./Off. Testing & Res.) Sept. 1997	US FDA (Centre for Drug Eval.& Res./Off. Testing & Res.) Sept. 1997

Model Applicability

Unknown features are fingerprint features in the query molecule, but not found or appearing too infrequently in the training set.

1. All properties and OPS components are within expected ranges.

Feature Contribution

Top features for positive contribution

Fingerprint	Bit/Smiles	Feature Structure	Score	Carcinogen in training set
SCFP_6	1958008606	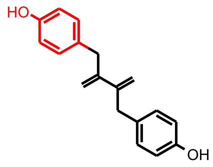 [*][c]1:[cH]:[cH]:[c](O):[cH]:[cH]:1	0.536	4 out of 6

SCFP_6	55464376	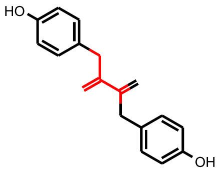 [*]CC(=C)C(=[*])[*]	0.345	14 out of 30
SCFP_6	1578073306	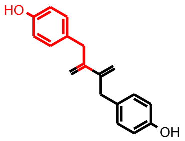 [*]C(=[*])C[c]1:[cH]:[cH]:[c](O):[cH]:[cH]:1	0.198	1 out of 2

Top Features for negative contribution

Fingerprint	Bit/Smiles	Feature Structure	Score	Carcinogen in training set
SCFP_6	1872184315	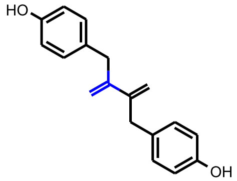 [*]C=C	-0.264	1 out of 5
SCFP_6	470101049	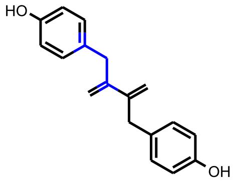 [*]=CC[c](:[*]):[*]	-0.259	2 out of 9
SCFP_6	-1379148975	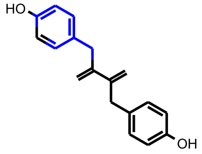 [*]C[c]1:[cH]:[*]:[c]([*]):[cH]:[cH]:1	-0.257	34 out of 138

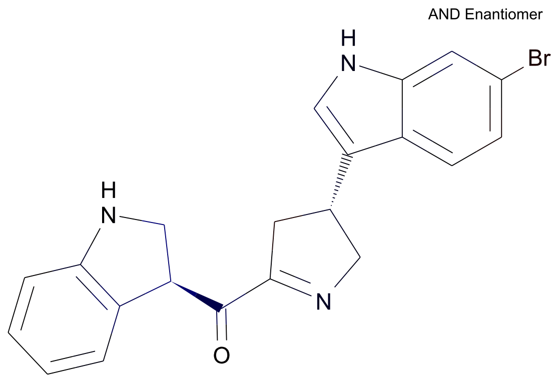

$C_{21}H_{18}BrN_3O$
Molecular Weight: 408.29112
ALogP: 3.919
Rotatable Bonds: 3
Acceptors: 3
Donors: 2

Model Prediction

Prediction: Non-Carcinogen
Probability: 0.31
Enrichment: 0.928
Bayesian Score: -1.71
Mahalanobis Distance: 14.9
Mahalanobis Distance p-value: 8.24e-007

Prediction: Positive if the Bayesian score is above the estimated best cutoff value from minimizing the false positive and false negative rate.
Probability: The esimated probability that the sample is in the positive category. This assumes that the Bayesian score follows a normal distribution and is different from the prediction using a cutoff.
Enrichment: An estimate of enrichment, that is, the increased likelihood (versus random) of this sample being in the category.
Bayesian Score: The standard Laplacian-modified Bayesian score.
Mahalanobis Distance: The Mahalanobis distance (MD) is the distance to the center of the training data. The larger the MD, the less trustworthy the prediction.
Mahalanobis Distance p-value: The p-value gives the fraction of training data with an MD greater than or equal to the one for the given sample, assuming normally distributed data. The smaller the p-value, the less trustworthy the prediciton. For highly non-normal X properties (e.g., fingerprints), the MD p-value is wildly inaccurate.

Structural Similar Compounds

Name	Mefloquine	Finasteride	Butorphanol
Structure	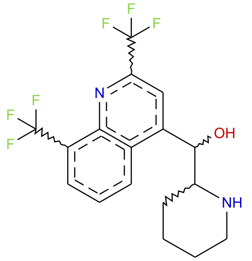	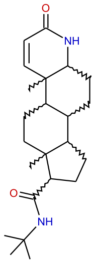	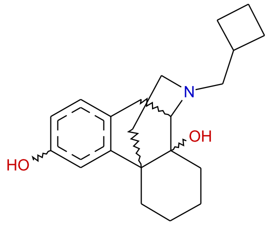
Actual Endpoint	Non-Carcinogen	Carcinogen	Non-Carcinogen
Predicted Endpoint	Non-Carcinogen	Carcinogen	Non-Carcinogen
Distance	0.548	0.630	0.641
Reference	US FDA (Centre for Drug Eval.& Res./Off. Testing & Res.) Sept. 1997	US FDA (Centre for Drug Eval.& Res./Off. Testing & Res.) Sept. 1997	US FDA (Centre for Drug Eval.& Res./Off. Testing & Res.) Sept. 1997

Model Applicability

Unknown features are fingerprint features in the query molecule, but not found or appearing too infrequently in the training set.

1. All properties and OPS components are within expected ranges.

Feature Contribution

Top features for positive contribution				
**Fingerprint**	**Bit/Smiles**	**Feature Structure**	**Score**	**Carcinogen in training set**
SCFP_6	-1379673609	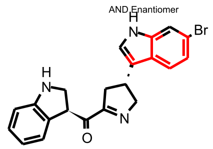 [*]:[c]1:[*]:[*]:[c]2:[*]:[cH]:[cH]:[cH]:[c]:1:2	0.526	11 out of 19

SCFP_6	-1380909229	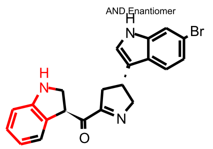 [*]1[*][c]2:[*]:[cH]:[cH]:[cH]:[c]:2N1	0.287	17 out of 39
SCFP_6	112346096	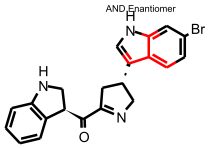 [*]:[cH]:[c]1:[c](:[*]):[*]:[*]:[c]:1:[*]	0.276	13 out of 30
**Top Features for negative contribution**				
**Fingerprint**	**Bit/Smiles**	**Feature Structure**	**Score**	**Carcinogen in training set**
SCFP_6	699559848	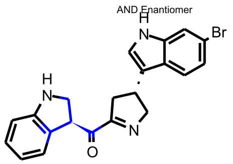 [*]C(=[*])[C@@H]1C[*] [*]:[c]1:[*]	-1.44	0 out of 10
SCFP_6	1582538652	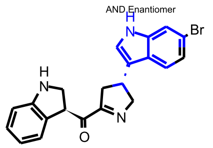 [*]C([*])[c]1:[cH]:[nH]:[c]2:[cH]:[c]([*]):[*]:[cH]:[c]:1:2	-0.674	0 out of 3
SCFP_6	18117904	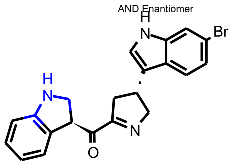 [*]:[c]1:[*][*]CN1	-0.578	1 out of 8

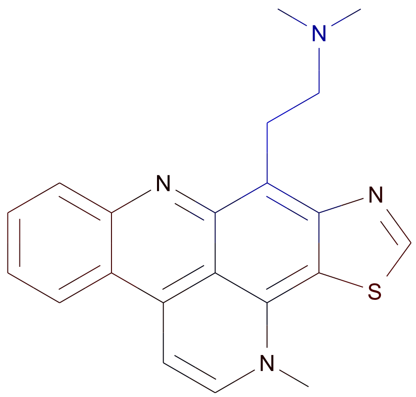

$C_{21}H_{20}N_4S$
Molecular Weight: 360.4753
ALogP: 3.682
Rotatable Bonds: 3
Acceptors: 4
Donors: 0

Structural Similar Compounds

Name	Chlormadinone	Risperidone	Metiapine
Structure	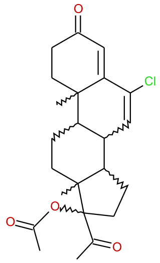	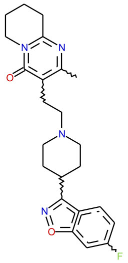	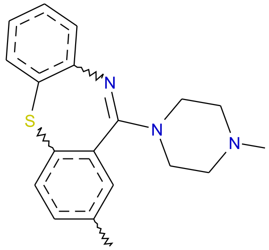
Actual Endpoint	Non-Carcinogen	Carcinogen	Non-Carcinogen
Predicted Endpoint	Non-Carcinogen	Carcinogen	Non-Carcinogen
Distance	0.577	0.578	0.591
Reference	US FDA (Centre for Drug Eval.& Res./Off. Testing & Res.) Sept. 1997	US FDA (Centre for Drug Eval.& Res./Off. Testing & Res.) Sept. 1997	US FDA (Centre for Drug Eval.& Res./Off. Testing & Res.) Sept. 1997

Model Prediction

Prediction: Carcinogen
Probability: 0.367
Enrichment: 1.1
Bayesian Score: 0.37
Mahalanobis Distance: 14.6
Mahalanobis Distance p-value: 3.96e-006

Prediction: Positive if the Bayesian score is above the estimated best cutoff value from minimizing the false positive and false negative rate.
Probability: The esimated probability that the sample is in the positive category. This assumes that the Bayesian score follows a normal distribution and is different from the prediction using a cutoff.
Enrichment: An estimate of enrichment, that is, the increased likelihood (versus random) of this sample being in the category.
Bayesian Score: The standard Laplacian-modified Bayesian score.
Mahalanobis Distance: The Mahalanobis distance (MD) is the distance to the center of the training data. The larger the MD, the less trustworthy the prediction.
Mahalanobis Distance p-value: The p-value gives the fraction of training data with an MD greater than or equal to the one for the given sample, assuming normally distributed data. The smaller the p-value, the less trustworthy the prediciton. For highly non-normal X properties (e.g., fingerprints), the MD p-value is wildly inaccurate.

Model Applicability

Unknown features are fingerprint features in the query molecule, but not found or appearing too infrequently in the training set.

1. All properties and OPS components are within expected ranges.

Feature Contribution

Top features for positive contribution

Fingerprint	Bit/Smiles	Feature Structure	Score	Carcinogen in training set
SCFP_6	1651620003	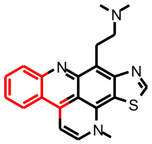 [*]:[c]1:[*]:[*]:[c]2:[cH]:[cH]:[cH]:[cH]:[c]:1:2	0.643	7 out of 10

SCFP_6	-1379673609	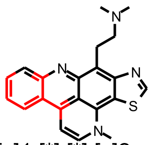<br>[*]:[c]1:[*]:[*]:[c]2<br>:[*]:[cH]:[cH]:[cH]:<br>[c]:1:2	0.526	11 out of 19
SCFP_6	1655199790	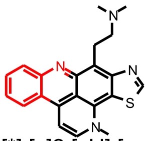<br>[*]1:[*]:[c]2:[cH]:[c<br>H]:[cH]:[cH]:[c]:2:[<br>nH]:1	0.52	5 out of 8
**Top Features for negative contribution**				
**Fingerprint**	**Bit/Smiles**	**Feature Structure**	**Score**	**Carcinogen in training set**
SCFP_6	-1853006127	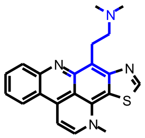<br>[*]N([*])CC[c](:[c](:<br>[*]):[*]):[c](:[*]):<br>[*]	-1.07	0 out of 6
SCFP_6	-183632224	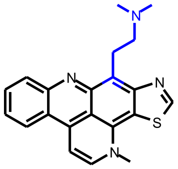<br>[*]:[c](:[*])CCN(C)C	-0.957	0 out of 5
SCFP_6	1882572951	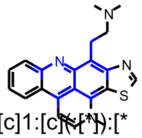<br>[*]C[c]1:[c](:[*]):[*<br>]:[c]([*]):[c]2:[c](<br>[*]):[*]:[c](:[*]):n<br>:[c]:1:2	-0.496	0 out of 2

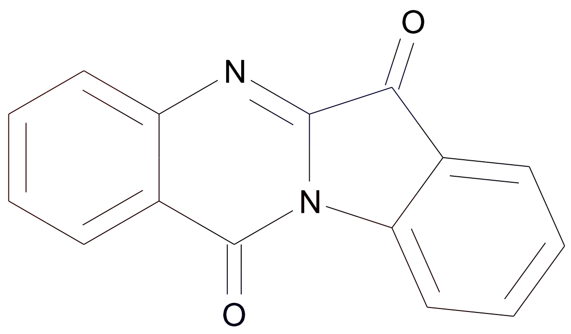

$C_{15}H_8N_2O_2$
Molecular Weight: 248.23621
ALogP: 2.331
Rotatable Bonds: 0
Acceptors: 3
Donors: 0

Model Prediction

Prediction: Carcinogen
Probability: 0.375
Enrichment: 1.12
Bayesian Score: 0.618
Mahalanobis Distance: 11.3
Mahalanobis Distance p-value: 0.13

Prediction: Positive if the Bayesian score is above the estimated best cutoff value from minimizing the false positive and false negative rate.
Probability: The esimated probability that the sample is in the positive category. This assumes that the Bayesian score follows a normal distribution and is different from the prediction using a cutoff.
Enrichment: An estimate of enrichment, that is, the increased likelihood (versus random) of this sample being in the category.
Bayesian Score: The standard Laplacian-modified Bayesian score.
Mahalanobis Distance: The Mahalanobis distance (MD) is the distance to the center of the training data. The larger the MD, the less trustworthy the prediction.
Mahalanobis Distance p-value: The p-value gives the fraction of training data with an MD greater than or equal to the one for the given sample, assuming normally distributed data. The smaller the p-value, the less trustworthy the prediciton. For highly non-normal X properties (e.g., fingerprints), the MD p-value is wildly inaccurate.

Structural Similar Compounds

Name	Methoxsalen; 8-	Levamisole	Estazolam
Structure	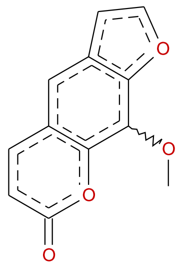	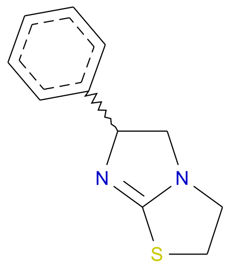	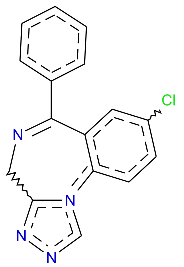
Actual Endpoint	Carcinogen	Non-Carcinogen	Non-Carcinogen
Predicted Endpoint	Carcinogen	Non-Carcinogen	Non-Carcinogen
Distance	0.551	0.569	0.614
Reference	US FDA (Centre for Drug Eval.& Res./Off. Testing & Res.) Sept. 1997	US FDA (Centre for Drug Eval.& Res./Off. Testing & Res.) Sept. 1997	US FDA (Centre for Drug Eval.& Res./Off. Testing & Res.) Sept. 1997

Model Applicability

Unknown features are fingerprint features in the query molecule, but not found or appearing too infrequently in the training set.

1. All properties and OPS components are within expected ranges.

Feature Contribution

Top features for positive contribution				
**Fingerprint**	**Bit/Smiles**	**Feature Structure**	**Score**	**Carcinogen in training set**
SCFP_6	-1377141613	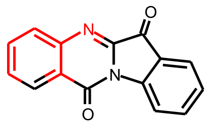 [*][c]1:[*]:[cH]:[cH]:[cH]:[c]:1N=[*]	0.429	3 out of 5

SCFP_6	1648492661	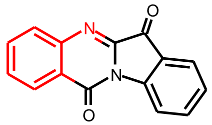 [*][c]1:[cH]:[cH]:[cH]:[cH]:[c]:1N=[*]	0.425	2 out of 3
SCFP_6	528802270	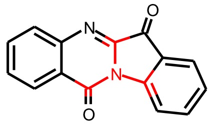 [*]C(=[*])N1C(=[*])[*][*]:[c]1:[*]	0.415	1 out of 1
**Top Features for negative contribution**				
**Fingerprint**	**Bit/Smiles**	**Feature Structure**	**Score**	**Carcinogen in training set**
SCFP_6	-1325723550	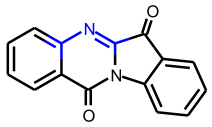 [*]C(=N[c](:[*]):[*]) [*]	-0.664	1 out of 9
SCFP_6	1257084377	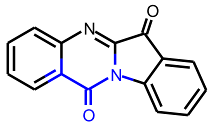 [*]N([*])C(=O)[c](:[*]):[*]	-0.436	4 out of 21
SCFP_6	353445762	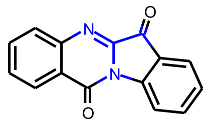 [*]\N=C\1/N([*])[*]:[*]C1=[*]	-0.278	0 out of 1

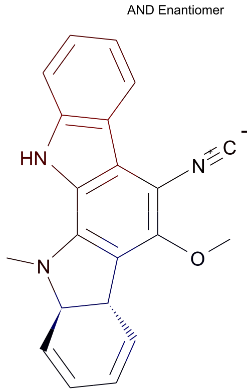

$C_{21}H_{17}N_3O$
Molecular Weight: 327.37918
ALogP: 4.078
Rotatable Bonds: 1
Acceptors: 2
Donors: 1

Structural Similar Compounds

Name	Levonorgestrel	Danazol	Norethindrone
Structure	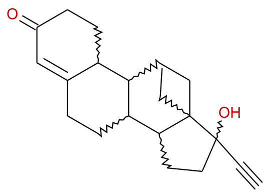	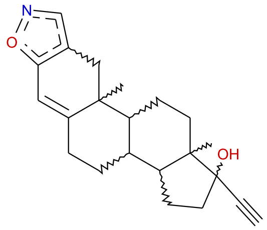	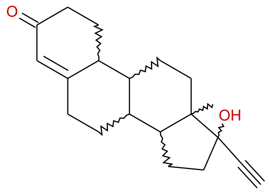
Actual Endpoint	Non-Carcinogen	Non-Carcinogen	Carcinogen
Predicted Endpoint	Carcinogen	Non-Carcinogen	Carcinogen
Distance	0.581	0.583	0.584
Reference	US FDA (Centre for Drug Eval.& Res./Off. Testing & Res.) Sept. 1997	US FDA (Centre for Drug Eval.& Res./Off. Testing & Res.) Sept. 1997	US FDA (Centre for Drug Eval.& Res./Off. Testing & Res.) Sept. 1997

Model Prediction

Prediction: Carcinogen
Probability: 0.402
Enrichment: 1.2
Bayesian Score: 1.5
Mahalanobis Distance: 19.6
Mahalanobis Distance p-value: 1.26e-017

Prediction: Positive if the Bayesian score is above the estimated best cutoff value from minimizing the false positive and false negative rate.
Probability: The esimated probability that the sample is in the positive category. This assumes that the Bayesian score follows a normal distribution and is different from the prediction using a cutoff.
Enrichment: An estimate of enrichment, that is, the increased likelihood (versus random) of this sample being in the category.
Bayesian Score: The standard Laplacian-modified Bayesian score.
Mahalanobis Distance: The Mahalanobis distance (MD) is the distance to the center of the training data. The larger the MD, the less trustworthy the prediction.
Mahalanobis Distance p-value: The p-value gives the fraction of training data with an MD greater than or equal to the one for the given sample, assuming normally distributed data. The smaller the p-value, the less trustworthy the prediciton. For highly non-normal X properties (e.g., fingerprints), the MD p-value is wildly inaccurate.

Model Applicability

Unknown features are fingerprint features in the query molecule, but not found or appearing too infrequently in the training set.

1. OPS PC33 out of range. Value: -3.1478. Training min, max, SD, explained variance: -2.8117, 3.0977, 0.9346, 0.0081.

Feature Contribution

Top features for positive contribution

Fingerprint	Bit/Smiles	Feature Structure	Score	Carcinogen in training set
SCFP_6	1651620003	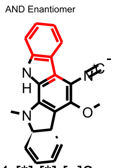 [*]:[c]1:[*]:[*]:[c]2:[cH]:[cH]:[cH]:[cH]:[c]:1:2	0.643	7 out of 10

SCFP_6	-1379673609	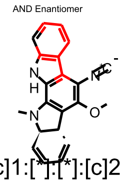 [*]:[c]1:[*]:[*]:[c]2:[*]:[cH]:[cH]:[cH]:[c]:1:2	0.526	11 out of 19
SCFP_6	1655199790	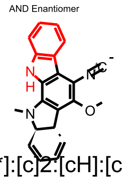 [*]1:[*]:[c]2:[cH]:[cH]:[cH]:[cH]:[c]:2:[nH]:1	0.52	5 out of 8

Top Features for negative contribution

Fingerprint	Bit/Smiles	Feature Structure	Score	Carcinogen in training set
SCFP_6	699559848	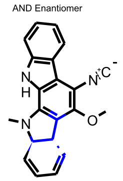 [*]C(=[*])[C@@H]1C[*][*]:[c]1:[*]	-1.44	0 out of 10
SCFP_6	-92192314	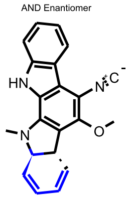 [*][C@H]1[*]C=CC=C1	-0.674	0 out of 3
SCFP_6	403834996	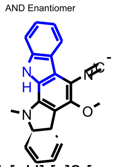 [*]:[c]1:[nH]:[c]2:[cH]:[cH]:[cH]:[cH]:[c]:2:[c]:1:[*]	-0.264	1 out of 5

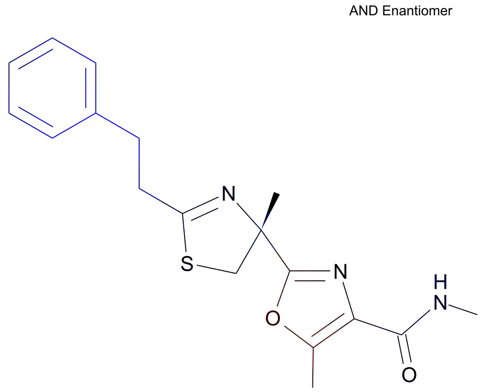

$C_{18}H_{21}N_3O_2S$
Molecular Weight: 343.44324
ALogP: 2.716
Rotatable Bonds: 5
Acceptors: 4
Donors: 1

Model Prediction

Prediction: Non-Carcinogen
Probability: 0.242
Enrichment: 0.723
Bayesian Score: -4.84
Mahalanobis Distance: 14.4
Mahalanobis Distance p-value: 1.01e-005

Prediction: Positive if the Bayesian score is above the estimated best cutoff value from minimizing the false positive and false negative rate.
Probability: The esimated probability that the sample is in the positive category. This assumes that the Bayesian score follows a normal distribution and is different from the prediction using a cutoff.
Enrichment: An estimate of enrichment, that is, the increased likelihood (versus random) of this sample being in the category.
Bayesian Score: The standard Laplacian-modified Bayesian score.
Mahalanobis Distance: The Mahalanobis distance (MD) is the distance to the center of the training data. The larger the MD, the less trustworthy the prediction.
Mahalanobis Distance p-value: The p-value gives the fraction of training data with an MD greater than or equal to the one for the given sample, assuming normally distributed data. The smaller the p-value, the less trustworthy the prediciton. For highly non-normal X properties (e.g., fingerprints), the MD p-value is wildly inaccurate.

Structural Similar Compounds

Name	Omeprazole	Lansoprazole	Oxaprocin
Structure	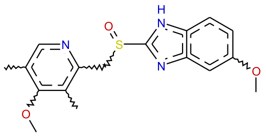	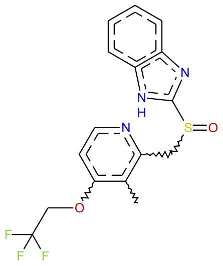	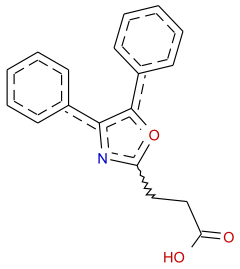
Actual Endpoint	Carcinogen	Carcinogen	Non-Carcinogen
Predicted Endpoint	Carcinogen	Carcinogen	Non-Carcinogen
Distance	0.552	0.560	0.580
Reference	US FDA (Centre for Drug Eval.& Res./Off. Testing & Res.) Sept. 1997	US FDA (Centre for Drug Eval.& Res./Off. Testing & Res.) Sept. 1997	US FDA (Centre for Drug Eval.& Res./Off. Testing & Res.) Sept. 1997

Model Applicability

Unknown features are fingerprint features in the query molecule, but not found or appearing too infrequently in the training set.

1. OPS PC25 out of range. Value: 4.2746. Training min, max, SD, explained variance: -2.9604, 3.8685, 1.096, 0.0112.

Feature Contribution

Top features for positive contribution				
**Fingerprint**	**Bit/Smiles**	**Feature Structure**	**Score**	**Carcinogen in training set**
SCFP_6	794417578	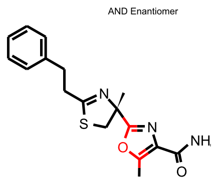 [*][c]1:[*]:[*]:[c]([*]):o:1	0.6	9 out of 14

SCFP_6	2109463705	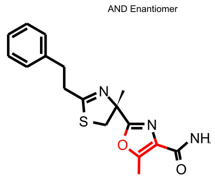 [*][c]1:[*]:[*]:o:[c]:1C	0.561	3 out of 4
SCFP_6	136686699	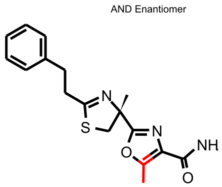 [*]:[c](:[*])C	0.287	17 out of 39
**Top Features for negative contribution**				
**Fingerprint**	**Bit/Smiles**	**Feature Structure**	**Score**	**Carcinogen in training set**
SCFP_6	-1211866396	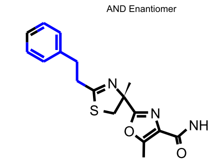 [*]C([*])C[c]1:[cH]:[cH]:[*]:[c]([*]):[cH]:1	-1.1	2 out of 25
SCFP_6	-1640858361	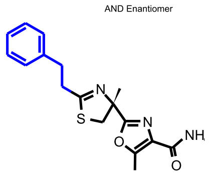 [*]CC[c]1:[cH]:[cH]:[cH]:[cH]:[cH]:1	-0.817	1 out of 11
SCFP_6	-1852892018	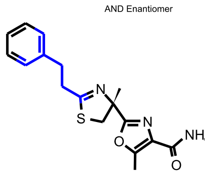 [*]C(=[*])CC[c](:[cH]:[*]):[cH]:[*]	-0.674	0 out of 3

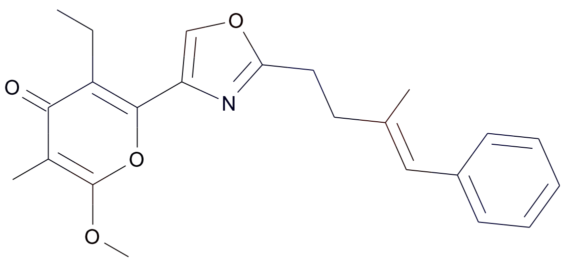

$C_{23}H_{25}NO_4$
Molecular Weight: 379.4489
ALogP: 5.22
Rotatable Bonds: 7
Acceptors: 4
Donors: 0

Model Prediction

Prediction: Non-Carcinogen
Probability: 0.337
Enrichment: 1.01
Bayesian Score: -0.673
Mahalanobis Distance: 14.9
Mahalanobis Distance p-value: 1.08e-006

Prediction: Positive if the Bayesian score is above the estimated best cutoff value from minimizing the false positive and false negative rate.
Probability: The esimated probability that the sample is in the positive category. This assumes that the Bayesian score follows a normal distribution and is different from the prediction using a cutoff.
Enrichment: An estimate of enrichment, that is, the increased likelihood (versus random) of this sample being in the category.
Bayesian Score: The standard Laplacian-modified Bayesian score.
Mahalanobis Distance: The Mahalanobis distance (MD) is the distance to the center of the training data. The larger the MD, the less trustworthy the prediction.
Mahalanobis Distance p-value: The p-value gives the fraction of training data with an MD greater than or equal to the one for the given sample, assuming normally distributed data. The smaller the p-value, the less trustworthy the prediciton. For highly non-normal X properties (e.g., fingerprints), the MD p-value is wildly inaccurate.

Structural Similar Compounds

Name	Permethrin	Chlorotrianisene	Etretinate
Structure	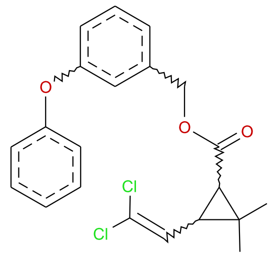	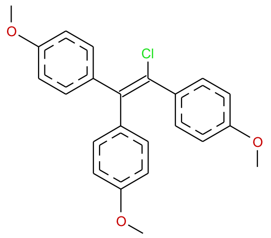	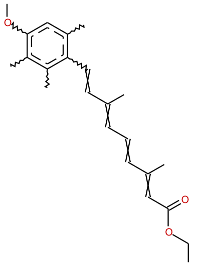
Actual Endpoint	Non-Carcinogen	Non-Carcinogen	Non-Carcinogen
Predicted Endpoint	Non-Carcinogen	Non-Carcinogen	Non-Carcinogen
Distance	0.566	0.605	0.607
Reference	US FDA (Centre for Drug Eval.& Res./Off. Testing & Res.) Sept. 1997	US FDA (Centre for Drug Eval.& Res./Off. Testing & Res.) Sept. 1997	US FDA (Centre for Drug Eval.& Res./Off. Testing & Res.) Sept. 1997

Model Applicability

Unknown features are fingerprint features in the query molecule, but not found or appearing too infrequently in the training set.

1. All properties and OPS components are within expected ranges.

Feature Contribution

Top features for positive contribution

Fingerprint	Bit/Smiles	Feature Structure	Score	Carcinogen in training set
SCFP_6	794417578	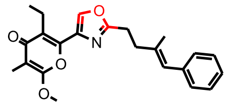 [*][c]1:[*]:[*]:[c]([*]):o:1	0.6	9 out of 14

SCFP_6	-1971137145	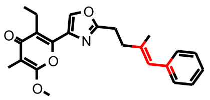 [*]\C=C\[c](:[*]):[*]	0.434	5 out of 9
SCFP_6	-496111702	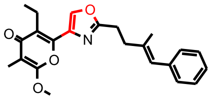 [*][c]1:[*]:[*]:o:[cH]:1	0.425	2 out of 3
**Top Features for negative contribution**				
**Fingerprint**	**Bit/Smiles**	**Feature Structure**	**Score**	**Carcinogen in training set**
SCFP_6	1653911926	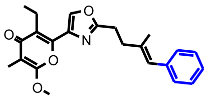 [*][c]1:[cH]:[cH]:[cH]:[cH]:[cH]:1	-0.504	12 out of 64
SCFP_6	-1012437048	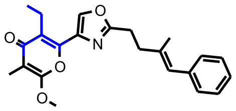 [*]C(=C(CC)C(=[*])[*])[*]	-0.496	0 out of 2
SCFP_6	-1272709286	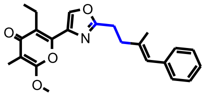 [*]C([*])C[c](:[*]):[*]	-0.459	12 out of 61

Remdesivir

TOPKAT_Rat_Male_FDA_None_vs_Carcinogen

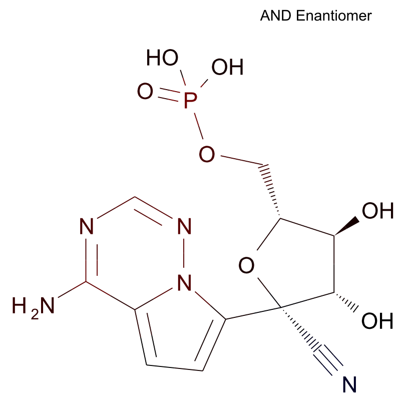

$C_{12}H_{14}N_5O_7P$
Molecular Weight: 371.24262
ALogP: -1.502
Rotatable Bonds: 4
Acceptors: 11
Donors: 5

Model Prediction

Prediction: Carcinogen
Probability: 0.481
Enrichment: 1.44
Bayesian Score: 3.82
Mahalanobis Distance: 14.1
Mahalanobis Distance p-value: 3.32e-005

Prediction: Positive if the Bayesian score is above the estimated best cutoff value from minimizing the false positive and false negative rate.
Probability: The esimated probability that the sample is in the positive category. This assumes that the Bayesian score follows a normal distribution and is different from the prediction using a cutoff.
Enrichment: An estimate of enrichment, that is, the increased likelihood (versus random) of this sample being in the category.
Bayesian Score: The standard Laplacian-modified Bayesian score.
Mahalanobis Distance: The Mahalanobis distance (MD) is the distance to the center of the training data. The larger the MD, the less trustworthy the prediction.
Mahalanobis Distance p-value: The p-value gives the fraction of training data with an MD greater than or equal to the one for the given sample, assuming normally distributed data. The smaller the p-value, the less trustworthy the prediciton. For highly non-normal X properties (e.g., fingerprints), the MD p-value is wildly inaccurate.

Structural Similar Compounds

Name	Streptozocin	Famotidine	Tetracycline
Structure	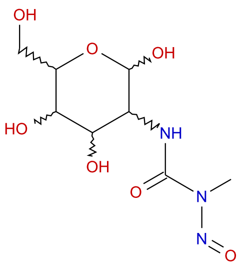	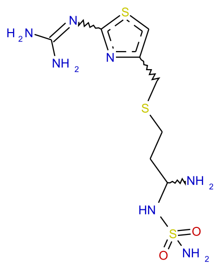	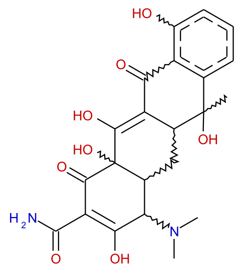
Actual Endpoint	Carcinogen	Non-Carcinogen	Non-Carcinogen
Predicted Endpoint	Carcinogen	Non-Carcinogen	Non-Carcinogen
Distance	0.789	0.850	0.856
Reference	US FDA (Centre for Drug Eval.& Res./Off. Testing & Res.) Sept. 1997	US FDA (Centre for Drug Eval.& Res./Off. Testing & Res.) Sept. 1997	US FDA (Centre for Drug Eval.& Res./Off. Testing & Res.) Sept. 1997

Model Applicability

Unknown features are fingerprint features in the query molecule, but not found or appearing too infrequently in the training set.

1. All properties and OPS components are within expected ranges.

Feature Contribution

Top features for positive contribution

Fingerprint	Bit/Smiles	Feature Structure	Score	Carcinogen in training set
SCFP_6	-1029620989	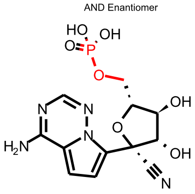 AND Enantiomer [*]COP(=[*])([*])[*]	0.712	3 out of 3

SCFP_6	1245795878	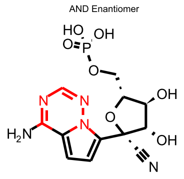 [*][c]1:[*]:n(:[*]):n :[cH]:n:1	0.603	2 out of 2
SCFP_6	149212520	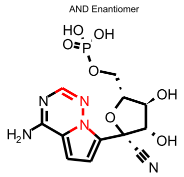 [*]:[cH]:n:n(:[*]):[*]	0.543	9 out of 15

Top Features for negative contribution

Fingerprint	Bit/Smiles	Feature Structure	Score	Carcinogen in training set
SCFP_6	-1019297400	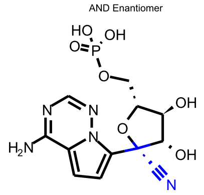 [*]C([*])([*])C#N	-0.674	0 out of 3
SCFP_6	194135988	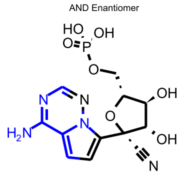 N[c]1:n:[cH]:[*]:n2:[*]:[*]:[cH]:[c]:1:2	-0.278	0 out of 1
SCFP_6	-424515134	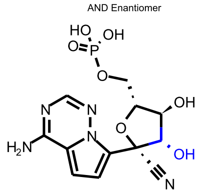 [*]C([*])O	-0.157	30 out of 110

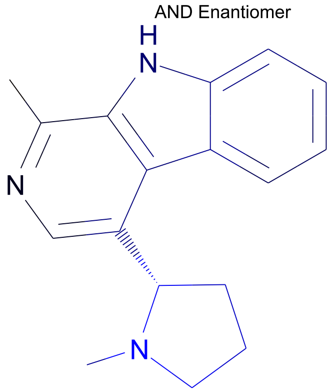

$C_{17}H_{19}N_3$
Molecular Weight: 265.35286
ALogP: 3.018
Rotatable Bonds: 1
Acceptors: 2
Donors: 1

Structural Similar Compounds

Name	Granisetron	Norethynodrel	Carbamazepine
Structure	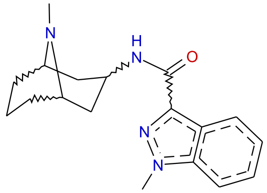	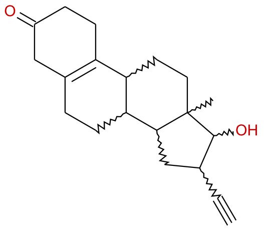	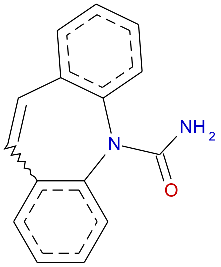
Actual Endpoint	Single-Carcinogen	Multiple-Carcinogen	Single-Carcinogen
Predicted Endpoint	Single-Carcinogen	Multiple-Carcinogen	Single-Carcinogen
Distance	0.594	0.627	0.647
Reference	US FDA (Centre for Drug Eval.& Res./Off. Testing & Res.) Sept. 1997	US FDA (Centre for Drug Eval.& Res./Off. Testing & Res.) Sept. 1997	US FDA (Centre for Drug Eval.& Res./Off. Testing & Res.) Sept. 1997

Model Prediction

Prediction: Single-Carcinogen
Probability: 0.267
Enrichment: 0.645
Bayesian Score: -8.93
Mahalanobis Distance: 11.7
Mahalanobis Distance p-value: 0.00774

Prediction: Positive if the Bayesian score is above the estimated best cutoff value from minimizing the false positive and false negative rate.
Probability: The esimated probability that the sample is in the positive category. This assumes that the Bayesian score follows a normal distribution and is different from the prediction using a cutoff.
Enrichment: An estimate of enrichment, that is, the increased likelihood (versus random) of this sample being in the category.
Bayesian Score: The standard Laplacian-modified Bayesian score.
Mahalanobis Distance: The Mahalanobis distance (MD) is the distance to the center of the training data. The larger the MD, the less trustworthy the prediction.
Mahalanobis Distance p-value: The p-value gives the fraction of training data with an MD greater than or equal to the one for the given sample, assuming normally distributed data. The smaller the p-value, the less trustworthy the prediciton. For highly non-normal X properties (e.g., fingerprints), the MD p-value is wildly inaccurate.

Model Applicability

Unknown features are fingerprint features in the query molecule, but not found or appearing too infrequently in the training set.

1. All properties and OPS components are within expected ranges.

Feature Contribution

Top features for positive contribution

Fingerprint	Bit/Smiles	Feature Structure	Score	Multiple-Carcinogen in training set
SCFP_8	136686699	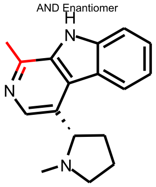 AND Enantiomer [*]:[c](:[*])C	0.332	9 out of 17

SCFP_8	-496201075	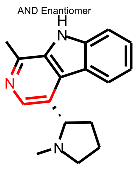 [*][c](:[*]):[cH]:n:[*]	0.216	7 out of 15
SCFP_8	2109165795	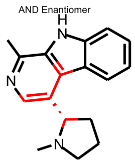 [*]C([*])[c](:[cH]:[*]):[c](:[*]):[*]	0.207	19 out of 42
**Top Features for negative contribution**				
**Fingerprint**	**Bit/Smiles**	**Feature Structure**	**Score**	**Multiple-Carcinogen in training set**
SCFP_8	-182915287	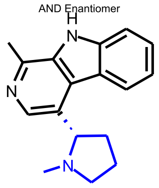 [*][C@@H]1CCCN1C	-0.84	1 out of 10
SCFP_8	698956551	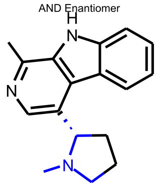 [*][C@@H]1[*][*]CN1C	-0.769	3 out of 21
SCFP_8	2088704928	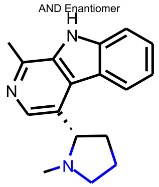 [*]N1[*][*]CC1	-0.653	5 out of 29

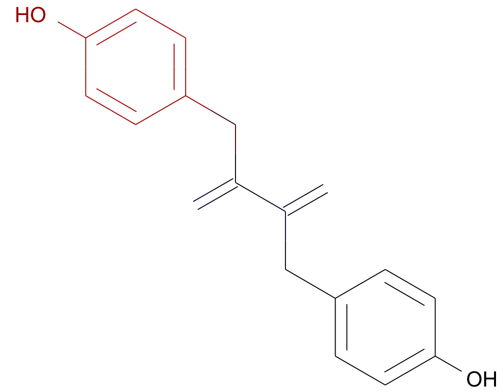

$C_{18}H_{18}O_2$
Molecular Weight: 266.33432
ALogP: 4.8
Rotatable Bonds: 5
Acceptors: 2
Donors: 2

Structural Similar Compounds

Name	Diethylstilbesterol	Gemfibrozil	Nafenopin
Structure	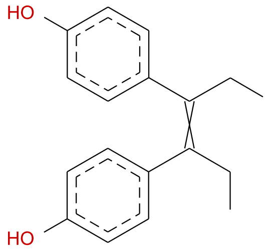	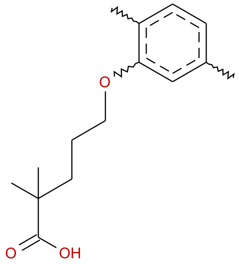	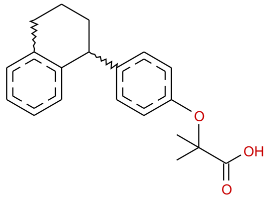
Actual Endpoint	Multiple-Carcinogen	Multiple-Carcinogen	Multiple-Carcinogen
Predicted Endpoint	Multiple-Carcinogen	Multiple-Carcinogen	Multiple-Carcinogen
Distance	0.455	0.664	0.668
Reference	US FDA (Centre for Drug Eval.& Res./Off. Testing & Res.) Sept. 1997	US FDA (Centre for Drug Eval.& Res./Off. Testing & Res.) Sept. 1997	US FDA (Centre for Drug Eval.& Res./Off. Testing & Res.) Sept. 1997

Model Prediction

Prediction: Multiple-Carcinogen
Probability: 0.564
Enrichment: 1.36
Bayesian Score: 4.28
Mahalanobis Distance: 11.6
Mahalanobis Distance p-value: 0.00965

Prediction: Positive if the Bayesian score is above the estimated best cutoff value from minimizing the false positive and false negative rate.
Probability: The esimated probability that the sample is in the positive category. This assumes that the Bayesian score follows a normal distribution and is different from the prediction using a cutoff.
Enrichment: An estimate of enrichment, that is, the increased likelihood (versus random) of this sample being in the category.
Bayesian Score: The standard Laplacian-modified Bayesian score.
Mahalanobis Distance: The Mahalanobis distance (MD) is the distance to the center of the training data. The larger the MD, the less trustworthy the prediction.
Mahalanobis Distance p-value: The p-value gives the fraction of training data with an MD greater than or equal to the one for the given sample, assuming normally distributed data. The smaller the p-value, the less trustworthy the prediciton. For highly non-normal X properties (e.g., fingerprints), the MD p-value is wildly inaccurate.

Model Applicability

Unknown features are fingerprint features in the query molecule, but not found or appearing too infrequently in the training set.

1. All properties and OPS components are within expected ranges.

Feature Contribution

Top features for positive contribution

Fingerprint	Bit/Smiles	Feature Structure	Score	Multiple-Carcinogen in training set
SCFP_8	1958008606	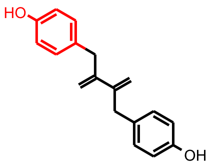 [*][c]1:[cH]:[cH]:[c](O):[cH]:[cH]:1	0.712	4 out of 4

SCFP_8	611156666	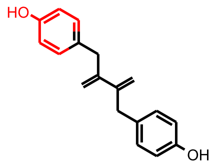 O[c]1:[cH]:[cH]:[*]:[cH]:[cH]:1	0.635	5 out of 6
SCFP_8	-1850560426	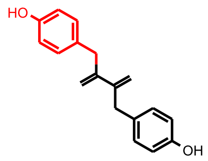 [*]C[c]1:[cH]:[cH]:[c](O):[cH]:[cH]:1	0.553	2 out of 2

Top Features for negative contribution

Fingerprint	Bit/Smiles	Feature Structure	Score	Multiple-Carcinogen in training set
SCFP_8	55464376	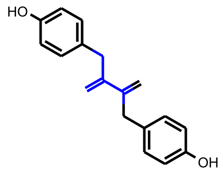 [*]CC(=C)C(=[*])[*]	-0.707	2 out of 14
SCFP_8	1872184315	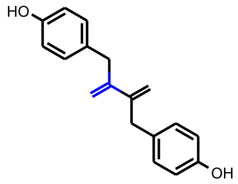 [*]C(=C)[*]	-0.31	0 out of 1
SCFP_8	-496409612	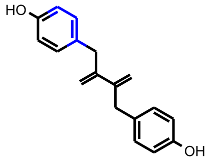 [*]:[cH]:[cH]:[cH]:[*]	0	31 out of 86

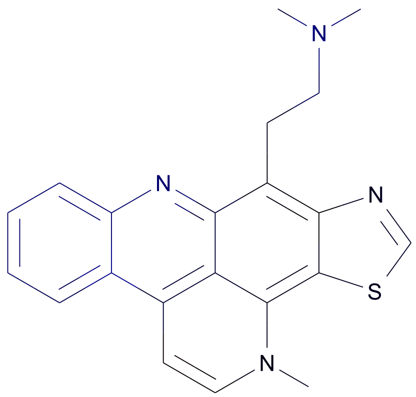

$C_{21}H_{20}N_4S$
Molecular Weight: 360.4753
ALogP: 3.682
Rotatable Bonds: 3
Acceptors: 4
Donors: 0

Structural Similar Compounds

Name	Risperidone	Chlorpromazine	Ethynodiol
Structure	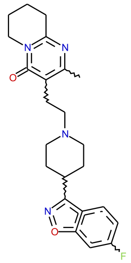	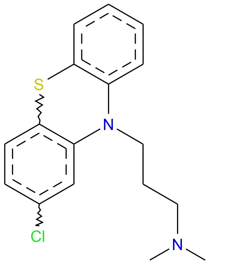	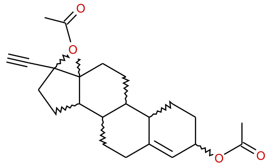
Actual Endpoint	Single-Carcinogen	Single-Carcinogen	Single-Carcinogen
Predicted Endpoint	Single-Carcinogen	Single-Carcinogen	Single-Carcinogen
Distance	0.590	0.645	0.652
Reference	US FDA (Centre for Drug Eval.& Res./Off. Testing & Res.) Sept. 1997	US FDA (Centre for Drug Eval.& Res./Off. Testing & Res.) Sept. 1997	US FDA (Centre for Drug Eval.& Res./Off. Testing & Res.) Sept. 1997

Model Prediction

Prediction: Single-Carcinogen
Probability: 0.38
Enrichment: 0.917
Bayesian Score: -6.58
Mahalanobis Distance: 18
Mahalanobis Distance p-value: 2.05e-008

Prediction: Positive if the Bayesian score is above the estimated best cutoff value from minimizing the false positive and false negative rate.
Probability: The esimated probability that the sample is in the positive category. This assumes that the Bayesian score follows a normal distribution and is different from the prediction using a cutoff.
Enrichment: An estimate of enrichment, that is, the increased likelihood (versus random) of this sample being in the category.
Bayesian Score: The standard Laplacian-modified Bayesian score.
Mahalanobis Distance: The Mahalanobis distance (MD) is the distance to the center of the training data. The larger the MD, the less trustworthy the prediction.
Mahalanobis Distance p-value: The p-value gives the fraction of training data with an MD greater than or equal to the one for the given sample, assuming normally distributed data. The smaller the p-value, the less trustworthy the prediciton. For highly non-normal X properties (e.g., fingerprints), the MD p-value is wildly inaccurate.

Model Applicability

Unknown features are fingerprint features in the query molecule, but not found or appearing too infrequently in the training set.

1. All properties and OPS components are within expected ranges.

Feature Contribution

Top features for positive contribution

Fingerprint	Bit/Smiles	Feature Structure	Score	Multiple-Carcinogen in training set
SCFP_8	-1971137145	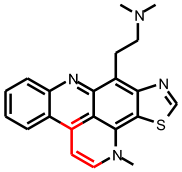 [*]\C=C/[c](:[*]):[*]	0.351	3 out of 5

SCFP_8	10	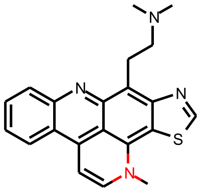 [*]N([*])[*]	0.226	18 out of 39
SCFP_8	2109165795	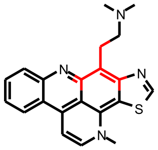 [*]C([*])[c](:[cH]:[*]):[c](:[*]):[*]	0.207	19 out of 42

Top Features for negative contribution

Fingerprint	Bit/Smiles	Feature Structure	Score	Multiple-Carcinogen in training set
SCFP_8	698956551	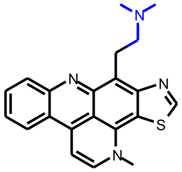 [*][C@@H]1[*][*]CN1C	-0.769	3 out of 21
SCFP_8	2088704928	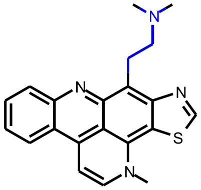 [*]N1[*][*]CC1	-0.653	5 out of 29
SCFP_8	-1381862798	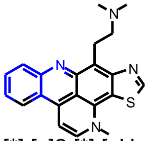 [*]1:[*]:[c]2:[*]:[cH]:[cH]:[cH]:[c]:2:[nH]:1	-0.572	1 out of 7

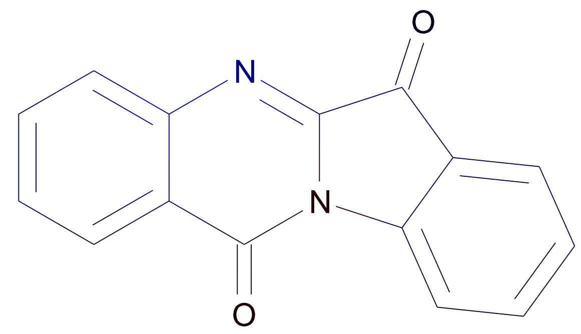

$C_{15}H_8N_2O_2$
Molecular Weight: 248.23621
ALogP: 2.331
Rotatable Bonds: 0
Acceptors: 3
Donors: 0

Model Prediction

Prediction: Single-Carcinogen
Probability: 0.499
Enrichment: 1.2
Bayesian Score: -3.45
Mahalanobis Distance: 8.67
Mahalanobis Distance p-value: 0.458

Prediction: Positive if the Bayesian score is above the estimated best cutoff value from minimizing the false positive and false negative rate.
Probability: The esimated probability that the sample is in the positive category. This assumes that the Bayesian score follows a normal distribution and is different from the prediction using a cutoff.
Enrichment: An estimate of enrichment, that is, the increased likelihood (versus random) of this sample being in the category.
Bayesian Score: The standard Laplacian-modified Bayesian score.
Mahalanobis Distance: The Mahalanobis distance (MD) is the distance to the center of the training data. The larger the MD, the less trustworthy the prediction.
Mahalanobis Distance p-value: The p-value gives the fraction of training data with an MD greater than or equal to the one for the given sample, assuming normally distributed data. The smaller the p-value, the less trustworthy the prediciton. For highly non-normal X properties (e.g., fingerprints), the MD p-value is wildly inaccurate.

Structural Similar Compounds

Name	Methoxsalen; 8-	Coumarin	Antipyrine
Structure	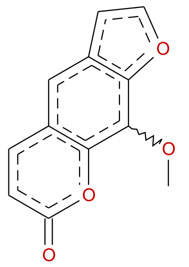	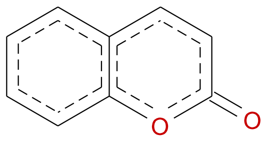	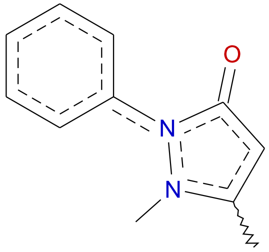
Actual Endpoint	Multiple-Carcinogen	Multiple-Carcinogen	Single-Carcinogen
Predicted Endpoint	Multiple-Carcinogen	Multiple-Carcinogen	Single-Carcinogen
Distance	0.562	0.657	0.667
Reference	US FDA (Centre for Drug Eval.& Res./Off. Testing & Res.) Sept. 1997	US FDA (Centre for Drug Eval.& Res./Off. Testing & Res.) Sept. 1997	US FDA (Centre for Drug Eval.& Res./Off. Testing & Res.) Sept. 1997

Model Applicability

Unknown features are fingerprint features in the query molecule, but not found or appearing too infrequently in the training set.

1. All properties and OPS components are within expected ranges.

Feature Contribution

Top features for positive contribution

Fingerprint	Bit/Smiles	Feature Structure	Score	Multiple-Carcinogen in training set
SCFP_8	1257084377	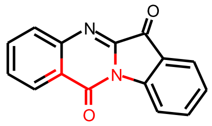 [*]N([*])C(=O)[c](:[*]):[*]	0.489	3 out of 4

SCFP_8	1205586762	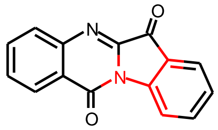<br>[*]N1[*][*][c](:[*]):<br>[c]1:[cH]:[*]	0.267	6 out of 12
SCFP_8	9	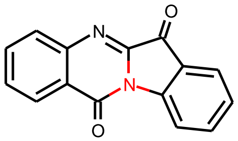<br>[*]N([*])[*]	0.253	19 out of 40

Top Features for negative contribution

Fingerprint	Bit/Smiles	Feature Structure	Score	Multiple-Carcinogen in training set
SCFP_8	-1980302127	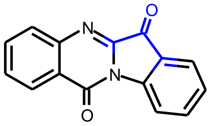<br>[*]=C1[*][*]:[c](:[*]<br>)C1=O	-0.737	0 out of 3
SCFP_8	-1377141613	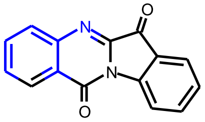<br>[*][c]1:[*]:[cH]:[cH]<br>:[cH]:[c]:1N=[*]	-0.737	0 out of 3
SCFP_8	1851000357	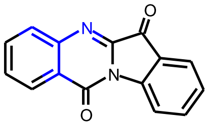<br>[*][c](:[*]):[c](:[cH<br>]:[*])N=[*]	-0.737	0 out of 3

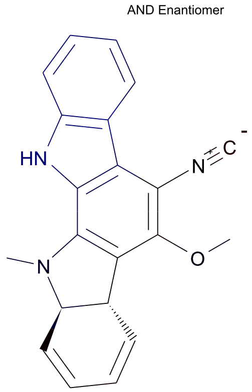

$C_{21}H_{17}N_3O$
Molecular Weight: 327.37918
ALogP: 4.078
Rotatable Bonds: 1
Acceptors: 2
Donors: 1

Model Prediction

Prediction: Single-Carcinogen
Probability: 0.491
Enrichment: 1.19
Bayesian Score: -3.73
Mahalanobis Distance: 25
Mahalanobis Distance p-value: 1.94e-014

Prediction: Positive if the Bayesian score is above the estimated best cutoff value from minimizing the false positive and false negative rate.
Probability: The esimated probability that the sample is in the positive category. This assumes that the Bayesian score follows a normal distribution and is different from the prediction using a cutoff.
Enrichment: An estimate of enrichment, that is, the increased likelihood (versus random) of this sample being in the category.
Bayesian Score: The standard Laplacian-modified Bayesian score.
Mahalanobis Distance: The Mahalanobis distance (MD) is the distance to the center of the training data. The larger the MD, the less trustworthy the prediction.
Mahalanobis Distance p-value: The p-value gives the fraction of training data with an MD greater than or equal to the one for the given sample, assuming normally distributed data. The smaller the p-value, the less trustworthy the prediciton. For highly non-normal X properties (e.g., fingerprints), the MD p-value is wildly inaccurate.

Structural Similar Compounds

Name	Norethindrone	Norethynodrel	Mestranol
Structure	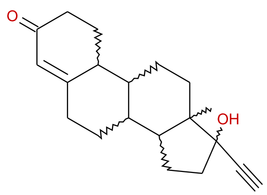	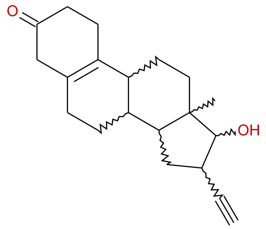	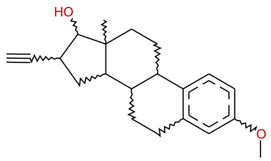
Actual Endpoint	Multiple-Carcinogen	Multiple-Carcinogen	Single-Carcinogen
Predicted Endpoint	Multiple-Carcinogen	Multiple-Carcinogen	Multiple-Carcinogen
Distance	0.590	0.592	0.607
Reference	US FDA (Centre for Drug Eval.& Res./Off. Testing & Res.) Sept. 1997	US FDA (Centre for Drug Eval.& Res./Off. Testing & Res.) Sept. 1997	US FDA (Centre for Drug Eval.& Res./Off. Testing & Res.) Sept. 1997

Model Applicability

Unknown features are fingerprint features in the query molecule, but not found or appearing too infrequently in the training set.

1. All properties and OPS components are within expected ranges.

Feature Contribution

Top features for positive contribution

Fingerprint	Bit/Smiles	Feature Structure	Score	Multiple-Carcinogen in training set
SCFP_8	2	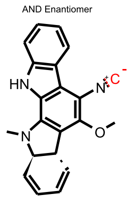 [*]#[C-]	0.584	6 out of 8

SCFP_8	-1971196727	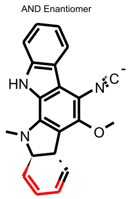 [*]\C=C/C=[*]	0.332	9 out of 17
SCFP_8	10	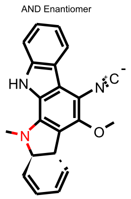 [*]N([*])[*]	0.226	18 out of 39

Top Features for negative contribution

Fingerprint	Bit/Smiles	Feature Structure	Score	Multiple-Carcinogen in training set
SCFP_8	-1853624961	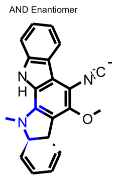 [*][C@@H]1[*][*]:[c](:[*])N1C	-0.669	1 out of 8
SCFP_8	-1381862798	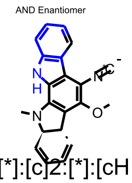 [*]1:[*]:[c]2:[*]:[cH]:[cH]:[cH]:[c]:2:[n H]:1	-0.572	1 out of 7
SCFP_8	136239834	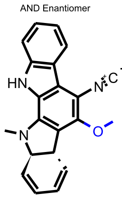 [*]OC	-0.358	3 out of 13

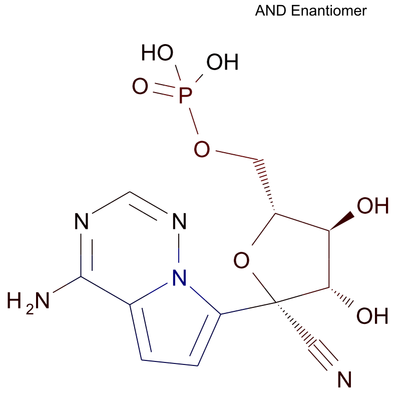

$C_{12}H_{14}N_5O_7P$
Molecular Weight: 371.24262
ALogP: -1.502
Rotatable Bonds: 4
Acceptors: 11
Donors: 5

Structural Similar Compounds

Name	Streptozocin	Minocycline	Ribavirin
Structure	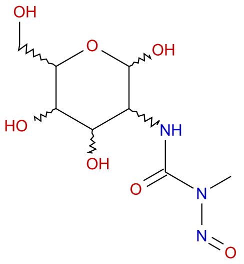	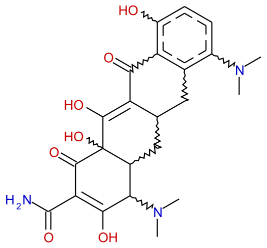	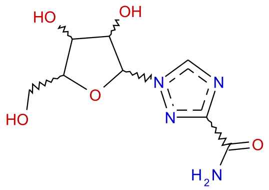
Actual Endpoint	Multiple-Carcinogen	Single-Carcinogen	Multiple-Carcinogen
Predicted Endpoint	Multiple-Carcinogen	Single-Carcinogen	Multiple-Carcinogen
Distance	0.817	0.908	0.929
Reference	US FDA (Centre for Drug Eval.& Res./Off. Testing & Res.) Sept. 1997	US FDA (Centre for Drug Eval.& Res./Off. Testing & Res.) Sept. 1997	US FDA (Centre for Drug Eval.& Res./Off. Testing & Res.) Sept. 1997

Model Prediction

Prediction: Multiple-Carcinogen
Probability: 0.556
Enrichment: 1.34
Bayesian Score: 3.52
Mahalanobis Distance: 14
Mahalanobis Distance p-value: 8.72e-005

Prediction: Positive if the Bayesian score is above the estimated best cutoff value from minimizing the false positive and false negative rate.
Probability: The esimated probability that the sample is in the positive category. This assumes that the Bayesian score follows a normal distribution and is different from the prediction using a cutoff.
Enrichment: An estimate of enrichment, that is, the increased likelihood (versus random) of this sample being in the category.
Bayesian Score: The standard Laplacian-modified Bayesian score.
Mahalanobis Distance: The Mahalanobis distance (MD) is the distance to the center of the training data. The larger the MD, the less trustworthy the prediction.
Mahalanobis Distance p-value: The p-value gives the fraction of training data with an MD greater than or equal to the one for the given sample, assuming normally distributed data. The smaller the p-value, the less trustworthy the prediciton. For highly non-normal X properties (e.g., fingerprints), the MD p-value is wildly inaccurate.

Model Applicability

Unknown features are fingerprint features in the query molecule, but not found or appearing too infrequently in the training set.

1. Num_H_Acceptors out of range. Value: 11. Training min, max, mean, SD: 0, 9, 3.8906, 2.196.

Feature Contribution

Top features for positive contribution

Fingerprint	Bit/Smiles	Feature Structure	Score	Multiple-Carcinogen in training set
SCFP_8	-1029620989	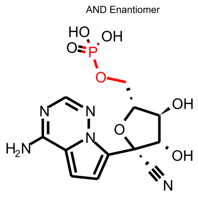 [*]COP(=[*])([*])[*]	0.649	3 out of 3

SCFP_8	2	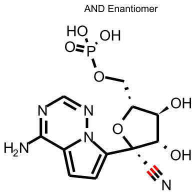 [*]#[C-]	0.584	6 out of 8
SCFP_8	-1486266146	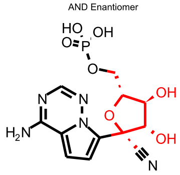 [*]C[C@H]1OC([*])([*])[C@@H](O)[C@@H]1O	0.553	2 out of 2

Top Features for negative contribution

**Fingerprint**	**Bit/Smiles**	**Feature Structure**	**Score**	**Multiple-Carcinogen in training set**
SCFP_8	-1381862798	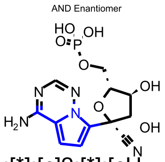 [*]1:[*]:[c]2:[*]:[cH]:[cH]:[cH]:[c]:2:[nH]:1	-0.572	1 out of 7
SCFP_8	1245795878	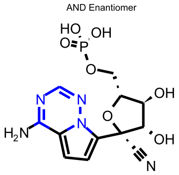 [*][c]1:[*]:n(:[*]):n:[cH]:n:1	-0.546	0 out of 2
SCFP_8	-1375522316	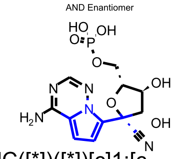 [*]C([*])([*])[c]1:[cH]:[cH]:[c](:[*]):n:1:[*]	-0.546	0 out of 2

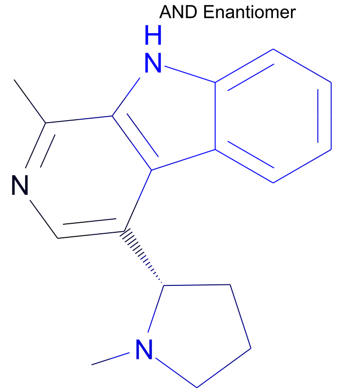

$C_{17}H_{19}N_3$
Molecular Weight: 265.35286
ALogP: 3.018
Rotatable Bonds: 1
Acceptors: 2
Donors: 1

Model Prediction

Prediction: Mild

Probability: 0.0343

Enrichment: 0.0932

Bayesian Score: -12.2

Mahalanobis Distance: 8.18

Mahalanobis Distance p-value: 0.704

Prediction: Positive if the Bayesian score is above the estimated best cutoff value from minimizing the false positive and false negative rate.
Probability: The esimated probability that the sample is in the positive category. This assumes that the Bayesian score follows a normal distribution and is different from the prediction using a cutoff.
Enrichment: An estimate of enrichment, that is, the increased likelihood (versus random) of this sample being in the category.
Bayesian Score: The standard Laplacian-modified Bayesian score.
Mahalanobis Distance: The Mahalanobis distance (MD) is the distance to the center of the training data. The larger the MD, the less trustworthy the prediction.
Mahalanobis Distance p-value: The p-value gives the fraction of training data with an MD greater than or equal to the one for the given sample, assuming normally distributed data. The smaller the p-value, the less trustworthy the prediciton. For highly non-normal X properties (e.g., fingerprints), the MD p-value is wildly inaccurate.

Structural Similar Compounds

Name	Ethanol, 2-(2,4-dichlorophenoxy)-	4-Biphenylol, 3-chloro-	Salicylic acid, p-tolyl ester
Structure	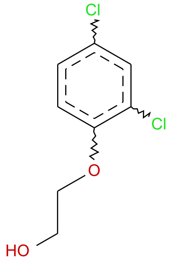	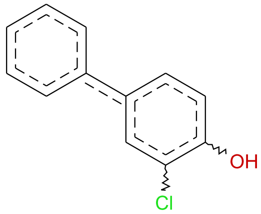	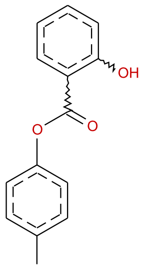
Actual Endpoint	Mild	Mild	Moderate_Severe
Predicted Endpoint	Mild	Mild	Moderate_Severe
Distance	0.642	0.661	0.675
Reference	85JCAE "Prehled Prumyslove Toxikologie; Organicke Latky," Marhold, J., Prague , Czechoslovakia, Avicenum, 1986 Volume(issue)/page/year: -,530,1986	85JCAE "Prehled Prumyslove Toxikologie; Organicke Latky," Marhold, J., Prague , Czechoslovakia, Avicenum, 1986 Volume(issue)/page/year: -,532,1986	FCTOD7 Food and Chemical Toxicology. (Pergamon Press Inc., Maxwell House, Fa irview Park, Elmsford, NY 10523) V.20- 1982- Volume(issue)/page/year: 21,835 ,1983

Model Applicability

Unknown features are fingerprint features in the query molecule, but not found or appearing too infrequently in the training set.

1. All properties and OPS components are within expected ranges.
2. Unknown FCFP_2 feature: 1871533475: [*]N1[*][*]C[C@H]1[c](:[*]):[*]

Feature Contribution

Top features for positive contribution

Fingerprint	Bit/Smiles	Feature Structure	Score	Moderate_Severe in training set

FCFP_12	-1272798659	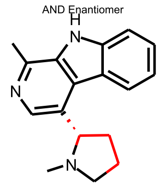<br>[*][C@H]1[*][*]CC1	0.204	227 out of 513
FCFP_12	-1462709112	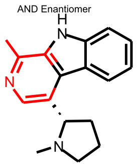<br>[*][c]1:[*]:[c](:[*])<br>:[c](C):n:[cH]:1	0.068	2 out of 5
**Top Features for negative contribution**				
**Fingerprint**	**Bit/Smiles**	**Feature Structure**	**Score**	**Moderate_Severe in training set**
FCFP_12	240509252	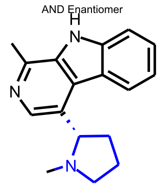<br>[*][C@@H]1CCCN1[*]	-1.15	0 out of 6
FCFP_12	309602933	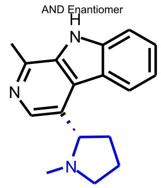<br>[*][C@@H]1CCCN1C	-1.03	0 out of 5
FCFP_12	307419094	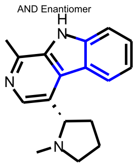<br>[*]:[cH]:[c]1:[c](:[*<br>]):[*]:[*]:[c]:1:[*]	-0.915	2 out of 18

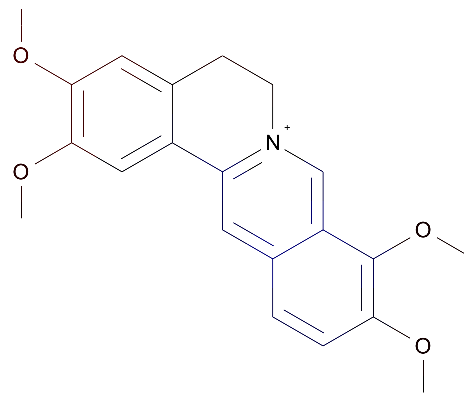

$C_{21}H_{22}NO_4$
Molecular Weight: 352.40368
ALogP: 4.161
Rotatable Bonds: 4
Acceptors: 4
Donors: 0

Structural Similar Compounds

Name	Anthranilic acid, N-(3-(p-tert-butylphenyl)-2-methylpropylidene)-, methyl ester	Pregna-3,5-diene-21-carboxylic acid, 3-ethoxy-17-hydroxy-, gamma-la ctone, (17-alpha)-	Acetic acid, phenyl-, 4-allyl-2-methoxyphenyl ester
Structure	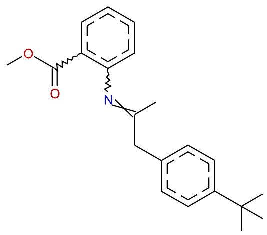	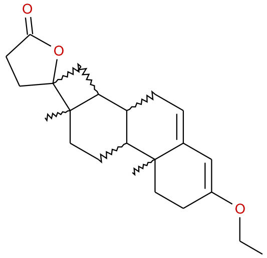	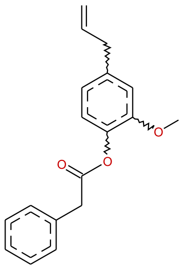
Actual Endpoint	Moderate_Severe	Mild	Moderate_Severe
Predicted Endpoint	Moderate_Severe	Mild	Moderate_Severe
Distance	0.614	0.626	0.660
Reference	FCTOD7 Food and Chemical Toxicology. (Pergamon Press Inc., Maxwell House, Fa irview Park, Elmsford, NY 10523) V.20- 1982- Volume(issue)/page/year: 20,729 ,1982	ATDAEI Acute Toxicity Data. Journal of the American College of Toxicology, Pa rt B. (Mary Ann Liebert, Inc., 1651 Third Ave., New York, NY 10128) V.1- 1990- Volume(issue)/page/year: 1,156,1992	FCTXAV Food and Cosmetics Toxicology. (London, UK) V.1-19, 1963-81. For pu blisher information, see FCTOD7. Volume(issue)/page/year: 16,753,1978

Model Prediction

Prediction: Mild
Probability: 0.306
Enrichment: 0.83
Bayesian Score: -2.56
Mahalanobis Distance: 5.92
Mahalanobis Distance p-value: 1

Prediction: Positive if the Bayesian score is above the estimated best cutoff value from minimizing the false positive and false negative rate.
Probability: The esimated probability that the sample is in the positive category. This assumes that the Bayesian score follows a normal distribution and is different from the prediction using a cutoff.
Enrichment: An estimate of enrichment, that is, the increased likelihood (versus random) of this sample being in the category.
Bayesian Score: The standard Laplacian-modified Bayesian score.
Mahalanobis Distance: The Mahalanobis distance (MD) is the distance to the center of the training data. The larger the MD, the less trustworthy the prediction.
Mahalanobis Distance p-value: The p-value gives the fraction of training data with an MD greater than or equal to the one for the given sample, assuming normally distributed data. The smaller the p-value, the less trustworthy the prediciton. For highly non-normal X properties (e.g., fingerprints), the MD p-value is wildly inaccurate.

Model Applicability

Unknown features are fingerprint features in the query molecule, but not found or appearing too infrequently in the training set.

1. All properties and OPS components are within expected ranges.
2. Unknown FCFP_2 feature: 24: [*][n+](:[*]):[*]
3. Unknown FCFP_2 feature: 414371600: [*]C[n+](:[c]([*]):[*]):c:[*]
4. Unknown FCFP_2 feature: -150573739: [*]CC[n+](:[*]):[*]
5. Unknown FCFP_2 feature: -1861407456: [*][n+](:[*]):[c]([c](:[*]):[*]):c:[*]
6. Unknown FCFP_2 feature: 1618392993: [*][n+](:[*]):c:[c](:[*]):[*]

Feature Contribution

Top features for positive contribution

Fingerprint	Bit/Smiles	Feature Structure	Score	Moderate_Severe in training set

FCFP_12	-1977641857	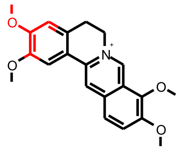 [*][c](:[*]):[c](OC):[cH]:[*]	0.416	18 out of 32
FCFP_12	-1370111440	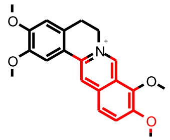 [*]O[c]1:[cH]:[cH]:[c]2:[cH]:[c]([*]):[*]:[cH]:[c]:2:[c]:1[*]	0.385	1 out of 1
FCFP_12	136627117	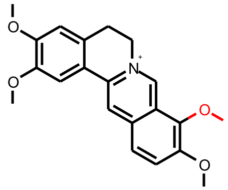 [*]OC	0.361	47 out of 90
**Top Features for negative contribution**				
**Fingerprint**	**Bit/Smiles**	**Feature Structure**	**Score**	**Moderate_Severe in training set**
FCFP_12	307419094	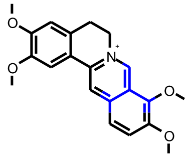 [*]:[cH]:[c]1:[c](:[*]):[*]:[*]:[c]:1:[*]	-0.915	2 out of 18
FCFP_12	-105186863	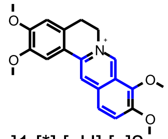 [*][c]1:[*]:[cH]:[c]2:[c]([*]):[*]:[cH]:[cH]:[c]:2:[cH]:1	-0.909	1 out of 11

FCFP_12	-1861645784	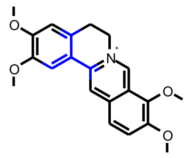 [*][c](:[*]):[c](:[cH]:[*])[c](:[*]):[*]	-0.834	1 out of 10

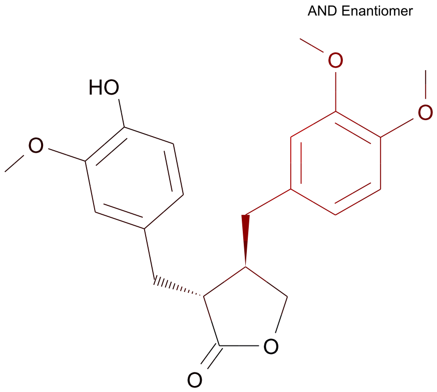

$C_{21}H_{24}O_6$
Molecular Weight: 372.41166
ALogP: 3.743
Rotatable Bonds: 7
Acceptors: 6
Donors: 1

Model Prediction

Prediction: Moderate_Severe

Probability: 0.523

Enrichment: 1.42

Bayesian Score: 2.75

Mahalanobis Distance: 9.59

Mahalanobis Distance p-value: 0.0889

Prediction: Positive if the Bayesian score is above the estimated best cutoff value from minimizing the false positive and false negative rate.
Probability: The esimated probability that the sample is in the positive category. This assumes that the Bayesian score follows a normal distribution and is different from the prediction using a cutoff.
Enrichment: An estimate of enrichment, that is, the increased likelihood (versus random) of this sample being in the category.
Bayesian Score: The standard Laplacian-modified Bayesian score.
Mahalanobis Distance: The Mahalanobis distance (MD) is the distance to the center of the training data. The larger the MD, the less trustworthy the prediction.
Mahalanobis Distance p-value: The p-value gives the fraction of training data with an MD greater than or equal to the one for the given sample, assuming normally distributed data. The smaller the p-value, the less trustworthy the prediciton. For highly non-normal X properties (e.g., fingerprints), the MD p-value is wildly inaccurate.

Structural Similar Compounds

**Name**	**1,4-Pentadien-3-one, 1,5-bis(p-azidophenyl)-**	**Pregna-1,4-diene-3,20-dione, 21-(acetyloxy)-11-hydroxy-6-methyl-17- (1-oxopropoxy)-, (6-alpha,11-beta)-**	**Diethylene glycol, dibenzoate**
Structure	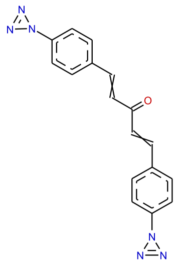	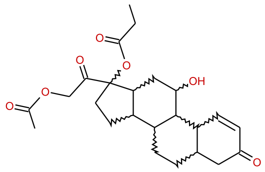	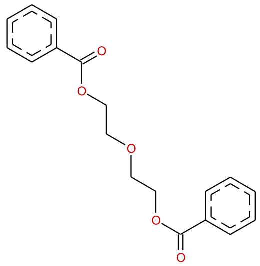
Actual Endpoint	Mild	Mild	Mild
Predicted Endpoint	Mild	Mild	Mild
Distance	0.741	0.760	0.760
Reference	85JCAE "Prehled Prumyslove Toxikologie; Organicke Latky," Marhold, J., Prague , Czechoslovakia, Avicenum, 1986 Volume(issue)/page/year: -,733,1986	YACHDS Yakuri to Chiryo. Pharmacology and Therapeutics. (Raifu Saiensu Shup pan K.K., 2-5-13, Yaesu, Chuo-ku, Tokyo 104, Japan) V.1- 1972- Volume(issue) /page/year: 19,3103,1991	85JCAE "Prehled Prumyslove Toxikologie; Organicke Latky," Marhold, J., Prague , Czechoslovakia, Avicenum, 1986 Volume(issue)/page/year: -,716,1986

Model Applicability

Unknown features are fingerprint features in the query molecule, but not found or appearing too infrequently in the training set.

1. All properties and OPS components are within expected ranges.

Feature Contribution

Top features for positive contribution				
**Fingerprint**	**Bit/Smiles**	**Feature Structure**	**Score**	**Moderate_Severe in training set**

FCFP_12	-2005486458	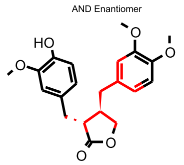<br>[*][C@H]1[*][*]C[C@@H]1C[c](:[cH]:[*]):[cH]:[*]	0.717	4 out of 4
FCFP_12	1985089045	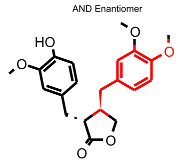<br>[*]O[c]1:[cH]:[cH]:[c](CC([*])[*]):[cH]:[c]:1[*]	0.435	5 out of 8
FCFP_12	-1977641857	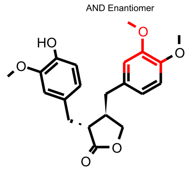<br>[*][c](:[*]):[c](OC):[cH]:[*]	0.416	18 out of 32
**Top Features for negative contribution**				
**Fingerprint**	**Bit/Smiles**	**Feature Structure**	**Score**	**Moderate_Severe in training set**
FCFP_12	1588282714	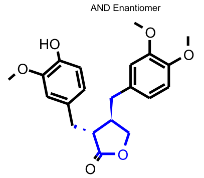<br>[*]C[C@H]1COC(=[*])[C@@H]1[*]	-0.26	7 out of 26
FCFP_12	7	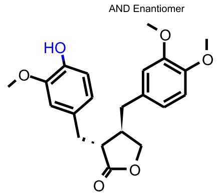<br>[*]O	-0.223	20 out of 70

FCFP_12	74595001	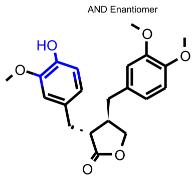 [*][c](:[*]):[c](O):[cH]:[*]	-0.197	9 out of 31

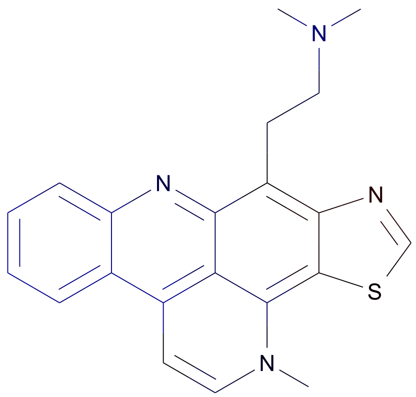

$C_{21}H_{20}N_4S$
Molecular Weight: 360.4753
ALogP: 3.682
Rotatable Bonds: 3
Acceptors: 4
Donors: 0

Structural Similar Compounds

Name	Pregna-3,5-diene-21-carboxylic acid, 3-ethoxy-17-hydroxy-, gamma-la ctone, (17-alpha)-	Benzenesulfonic acid, p-chlorophenyl ester	Ether, 4-chlorophenyl (4'-chloro-2'-nitro)phenyl
Structure	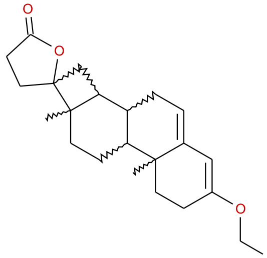	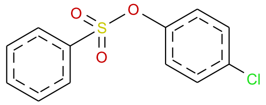	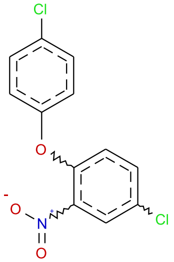
Actual Endpoint	Mild	Mild	Mild
Predicted Endpoint	Mild	Mild	Mild
Distance	0.682	0.694	0.695
Reference	ATDAEI Acute Toxicity Data. Journal of the American College of Toxicology, Pa rt B. (Mary Ann Liebert, Inc., 1651 Third Ave., New York, NY 10128) V.1- 1990- Volume(issue)/page/year: 1,156,1992	85JCAE "Prehled Prumyslove Toxikologie; Organicke Latky," Marhold, J., Prague , Czechoslovakia, Avicenum, 1986 Volume(issue)/page/year: -,1068,1986	28ZPAK "Sbornik Vysledku Toxixologickeho Vysetreni Latek A Pripravku," Marhol d, J.V., Institut Pro Vychovu Vedoucicn Pracovniku Chemickeho Prumyclu Praha, Cz echoslovakia, 1972 Volume(issue)/page/year: -,84,19

Model Prediction

Prediction: Mild
Probability: 0.108
Enrichment: 0.294
Bayesian Score: -8.03
Mahalanobis Distance: 8
Mahalanobis Distance p-value: 0.783

Prediction: Positive if the Bayesian score is above the estimated best cutoff value from minimizing the false positive and false negative rate.
Probability: The esimated probability that the sample is in the positive category. This assumes that the Bayesian score follows a normal distribution and is different from the prediction using a cutoff.
Enrichment: An estimate of enrichment, that is, the increased likelihood (versus random) of this sample being in the category.
Bayesian Score: The standard Laplacian-modified Bayesian score.
Mahalanobis Distance: The Mahalanobis distance (MD) is the distance to the center of the training data. The larger the MD, the less trustworthy the prediction.
Mahalanobis Distance p-value: The p-value gives the fraction of training data with an MD greater than or equal to the one for the given sample, assuming normally distributed data. The smaller the p-value, the less trustworthy the prediciton. For highly non-normal X properties (e.g., fingerprints), the MD p-value is wildly inaccurate.

Model Applicability

Unknown features are fingerprint features in the query molecule, but not found or appearing too infrequently in the training set.

1. All properties and OPS components are within expected ranges.

Feature Contribution

Top features for positive contribution

Fingerprint	Bit/Smiles	Feature Structure	Score	Moderate_Severe in training set

FCFP_12	136627117	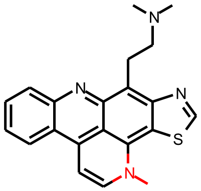 [*]OC	0.361	47 out of 90
FCFP_12	907007053	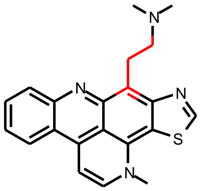 [*]CC[c](:[*]):[*]	0.245	27 out of 58
FCFP_12	-124685461	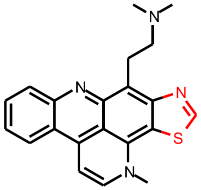 [*]1:[*]:s:[cH]:n:1	0.206	2 out of 4
**Top Features for negative contribution**				
**Fingerprint**	**Bit/Smiles**	**Feature Structure**	**Score**	**Moderate_Severe in training set**
FCFP_12	675769755	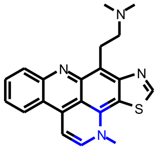 [*]=CN(C)[c](:[*]):[*]	-1.05	1 out of 13
FCFP_12	307419094	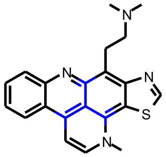 [*]:[cH]:[c]1:[c](:[*]):[*]:[*]:[c]:1:[*]	-0.915	2 out of 18

FCFP_12	-587569116	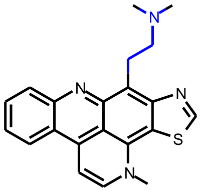 [*]N1[*][*]CC1	-0.764	4 out of 27

AND Enantiomer

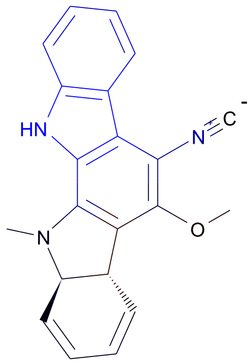

$C_{21}H_{17}N_3O$

Molecular Weight: 327.37918

ALogP: 4.078

Rotatable Bonds: 1

Acceptors: 2

Donors: 1

Model Prediction

Prediction: Mild

Probability: 0.04

Enrichment: 0.109

Bayesian Score: -11.7

Mahalanobis Distance: 9.19

Mahalanobis Distance p-value: 0.203

Prediction: Positive if the Bayesian score is above the estimated best cutoff value from minimizing the false positive and false negative rate.
Probability: The esimated probability that the sample is in the positive category. This assumes that the Bayesian score follows a normal distribution and is different from the prediction using a cutoff.
Enrichment: An estimate of enrichment, that is, the increased likelihood (versus random) of this sample being in the category.
Bayesian Score: The standard Laplacian-modified Bayesian score.
Mahalanobis Distance: The Mahalanobis distance (MD) is the distance to the center of the training data. The larger the MD, the less trustworthy the prediction.
Mahalanobis Distance p-value: The p-value gives the fraction of training data with an MD greater than or equal to the one for the given sample, assuming normally distributed data. The smaller the p-value, the less trustworthy the prediciton. For highly non-normal X properties (e.g., fingerprints), the MD p-value is wildly inaccurate.

Structural Similar Compounds

Name	Benzenesulfonamide, N-methyl-2,4,5-trichloro-	Aniline, 2,4-bis(o-methylphenoxy)-	Anthraquinone, 1-(2,4,6-trimethylphenylamino)-
Structure	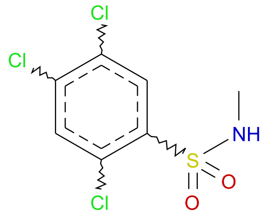	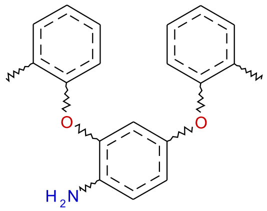	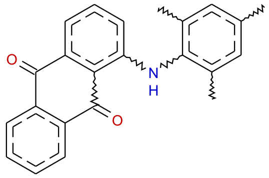
Actual Endpoint	Mild	Mild	Mild
Predicted Endpoint	Mild	Mild	Mild
Distance	0.660	0.679	0.682
Reference	85JCAE "Prehled Prumyslove Toxikologie; Organicke Latky," Marhold, J., Prague , Czechoslovakia, Avicenum, 1986 Volume(issue)/page/year: -,1074,1986	85JCAE "Prehled Prumyslove Toxikologie; Organicke Latky," Marhold, J., Prague , Czechoslovakia, Avicenum, 1986 Volume(issue)/page/year: -,725,1986	28ZPAK "Sbornik Vysledku Toxixologickeho Vysetreni Latek A Pripravku," Marhol d, J.V., Institut Pro Vychovu Vedoucicn Pracovniku Chemickeho Prumyclu Praha, Cz echoslovakia, 1972 Volume(issue)/page/year: -,242,1

Model Applicability

Unknown features are fingerprint features in the query molecule, but not found or appearing too infrequently in the training set.

1. All properties and OPS components are within expected ranges.
2. Unknown FCFP_2 feature: 4: [*]#[C-]
3. Unknown FCFP_2 feature: 1934974835: [*]:[c](:[*])[N+]#[C-]
4. Unknown FCFP_2 feature: -1487147388: [*][N+]#[C-]

Feature Contribution

Top features for positive contribution				
Fingerprint	Bit/Smiles	Feature Structure	Score	Moderate_Severe in training set

FCFP_12	1186303932	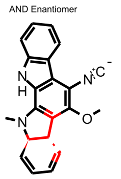 [*][C@H]1[*][*]:[c](:[*])[C@@H]1C=[*]	0.63	9 out of 12
FCFP_12	-1977641857	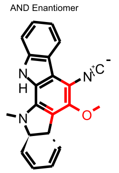 [*][c](:[*]):[c](OC):[cH]:[*]	0.416	18 out of 32
FCFP_12	136627117	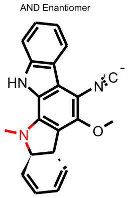 [*]OC	0.361	47 out of 90

Top Features for negative contribution

Fingerprint	Bit/Smiles	Feature Structure	Score	Moderate_Severe in training set
FCFP_12	8	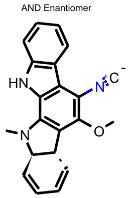 [*][N+]#[*]	-1.6	0 out of 11
FCFP_12	-828984032	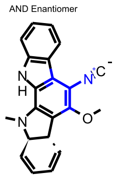 [*][c](:[*]):[c]([N+]#[*]):[c](:[*]):[*]	-1.36	0 out of 8

FCFP_12	675769755	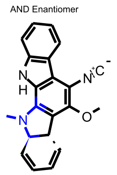 [*]=CN(C)[c](:[*]):[*<br>]	-1.05	1 out of 13

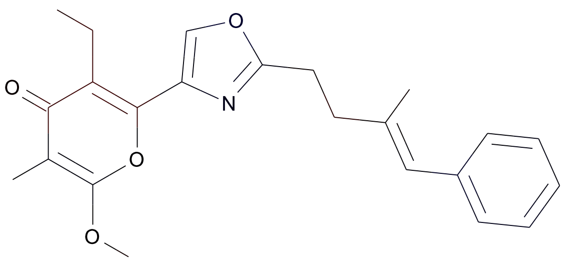

$C_{23}H_{25}NO_4$
Molecular Weight: 379.4489
ALogP: 5.22
Rotatable Bonds: 7
Acceptors: 4
Donors: 0

Structural Similar Compounds

Name	Phosphorothioic acid, O-ethyl S-propyl O-(2,4,6-trichlorophenyl) es ter	Propanoic acid, 2-(4-((5-(trifluoromethyl)-2-pyridinyl)oxy)phenoxy) -, butyl ester	Carbamic acid, ((dibutylamino)thio)methyl -, 2,2-dimethyl-2,3-dihydr o-7-benzofuranyl ester
Structure	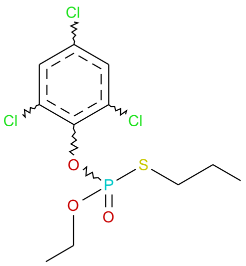	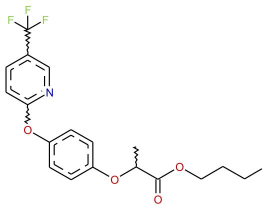	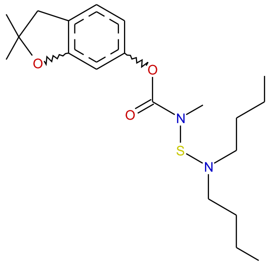
Actual Endpoint	Moderate_Severe	Mild	Mild
Predicted Endpoint	Mild	Mild	Mild
Distance	0.570	0.622	0.628
Reference	NTIS** National Technical Information Service. (Springfield, VA 22161) Forme rly U.S. Clearinghouse for Scientific & Technical Information. Volume(issue)/pag e/year: OTS0535844	NNGADV Nippon Noyaku Gakkaishi. Journal of the Pesticide Science Society of Japan. (Nippon Noyaku Gakkai, 1-43-11, Komagome, Toshima-ku, Tokyo 170, Japan) V.1- 1976- Volume(issue)/page/year: 15,305,1990	NTIS** National Technical Information Service. (Springfield, VA 22161) Forme rly U.S. Clearinghouse for Scientific & Technical Information. Volume(issue)/pag e/year: OTS0539690

Model Prediction

Prediction: Mild
Probability: 0.368
Enrichment: 1
Bayesian Score: -1.11
Mahalanobis Distance: 8.67
Mahalanobis Distance p-value: 0.444

Prediction: Positive if the Bayesian score is above the estimated best cutoff value from minimizing the false positive and false negative rate.
Probability: The esimated probability that the sample is in the positive category. This assumes that the Bayesian score follows a normal distribution and is different from the prediction using a cutoff.
Enrichment: An estimate of enrichment, that is, the increased likelihood (versus random) of this sample being in the category.
Bayesian Score: The standard Laplacian-modified Bayesian score.
Mahalanobis Distance: The Mahalanobis distance (MD) is the distance to the center of the training data. The larger the MD, the less trustworthy the prediction.
Mahalanobis Distance p-value: The p-value gives the fraction of training data with an MD greater than or equal to the one for the given sample, assuming normally distributed data. The smaller the p-value, the less trustworthy the prediciton. For highly non-normal X properties (e.g., fingerprints), the MD p-value is wildly inaccurate.

Model Applicability

Unknown features are fingerprint features in the query molecule, but not found or appearing too infrequently in the training set.

1. All properties and OPS components are within expected ranges.

Feature Contribution

Top features for positive contribution				
Fingerprint	Bit/Smiles	Feature Structure	Score	Moderate_Severe in training set

FCFP_12	436886043	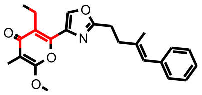 [*]CC(=C([*])[*])C(=[*])[*]	0.503	68 out of 113
FCFP_12	-2084412427	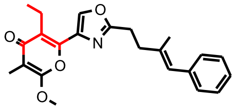 [*]C(=C(CC)C(=[*])[*])[*]	0.365	2 out of 3
FCFP_12	136627117	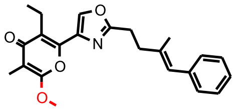 [*]OC	0.361	47 out of 90
**Top Features for negative contribution**				
**Fingerprint**	**Bit/Smiles**	**Feature Structure**	**Score**	**Moderate_Severe in training set**
FCFP_12	17	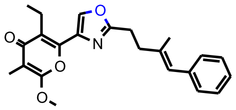 [*]:n:[*]	-0.332	8 out of 32
FCFP_12	-2115241127	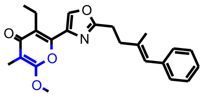 [*]OC(=C([*])[*])O[*]	-0.308	0 out of 1

FCFP_12	-1539162406	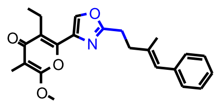 [*]C[c]1:o:[*]:[*]:n:1	-0.308	0 out of 1

Remdesivir

TOPKAT_Skin_Irritancy_Mild_vs_Moderate_Severe

AND Enantiomer

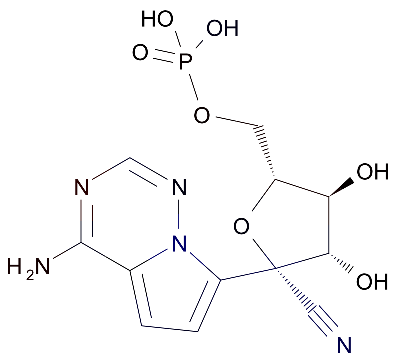

$C_{12}H_{14}N_5O_7P$
Molecular Weight: 371.24262
ALogP: -1.502
Rotatable Bonds: 4
Acceptors: 11
Donors: 5

Structural Similar Compounds

Name	1,3,6-Naphthalenetrisulfonic acid, 7-amino-	2,7-Anthracenedisulfonic acid, 9,10-dihydro-4,5-diamino-9,10-dioxo- 1-hydroxy-, disodium salt	1,5-Naphthalenedisulfonic acid, 2-amino-
Structure	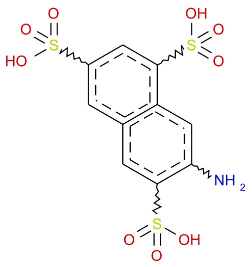	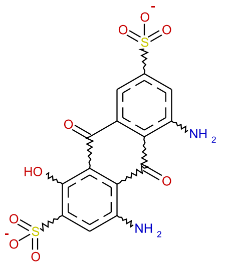	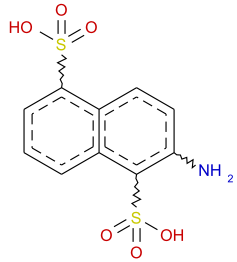
Actual Endpoint	Mild	Mild	Mild
Predicted Endpoint	Mild	Mild	Mild
Distance	0.759	1.033	1.137
Reference	85JCAE "Prehled Prumyslove Toxikologie; Organicke Latky," Marhold, J., Prague , Czechoslovakia, Avicenum, 1986 Volume(issue)/page/year: -,1058,1986	28ZPAK "Sbornik Vysledku Toxixologickeho Vysetreni Latek A Pripravku," Marhold d, J.V., Institut Pro Vychovu Vedoucicn Pracovniku Chemickeho Prumyclu Praha, Cz echoslovakia, 1972 Volume(issue)/page/year: -,239,1	85JCAE "Prehled Prumyslove Toxikologie; Organicke Latky," Marhold, J., Prague , Czechoslovakia, Avicenum, 1986 Volume(issue)/page/year: -,1058,1986

Model Prediction

Prediction: Mild
Probability: 0.0911
Enrichment: 0.247
Bayesian Score: -8.73
Mahalanobis Distance: 13.5
Mahalanobis Distance p-value: 1.21e-009

Prediction: Positive if the Bayesian score is above the estimated best cutoff value from minimizing the false positive and false negative rate.
Probability: The esimated probability that the sample is in the positive category. This assumes that the Bayesian score follows a normal distribution and is different from the prediction using a cutoff.
Enrichment: An estimate of enrichment, that is, the increased likelihood (versus random) of this sample being in the category.
Bayesian Score: The standard Laplacian-modified Bayesian score.
Mahalanobis Distance: The Mahalanobis distance (MD) is the distance to the center of the training data. The larger the MD, the less trustworthy the prediction.
Mahalanobis Distance p-value: The p-value gives the fraction of training data with an MD greater than or equal to the one for the given sample, assuming normally distributed data. The smaller the p-value, the less trustworthy the prediciton. For highly non-normal X properties (e.g., fingerprints), the MD p-value is wildly inaccurate.

Model Applicability

Unknown features are fingerprint features in the query molecule, but not found or appearing too infrequently in the training set.

1. All properties and OPS components are within expected ranges.
2. Unknown FCFP_2 feature: 472180098: [*]OP(=O)(O)O
3. Unknown FCFP_2 feature: -332197802: [*][c]1:[*]:[*]:[c](:[*]):n:1:n:[*]

Feature Contribution

Top features for positive contribution

Fingerprint	Bit/Smiles	Feature Structure	Score	Moderate_Severe in training set

FCFP_12	76292238	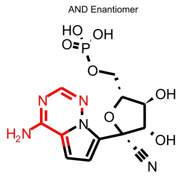<br>[*]:[c]1:[*]:n:[cH]:n:[c]:1N	0.385	1 out of 1
FCFP_12	-1151884458	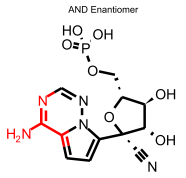<br>[*]:n:[c](N):[c](:[*]):[*]	0.385	1 out of 1
FCFP_12	-124685461	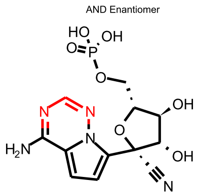<br>[*]1:[*]:s:[cH]:n:1	0.206	2 out of 4
**Top Features for negative contribution**				
**Fingerprint**	**Bit/Smiles**	**Feature Structure**	**Score**	**Moderate_Severe in training set**
FCFP_12	4427049	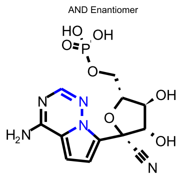<br>[*]:[cH]:n:n(:[*]):[*]	-0.893	0 out of 4
FCFP_12	-1277879912	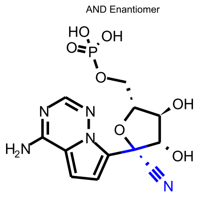<br>[*]C([*])([*])C#N	-0.548	5 out of 26

FCFP_12	-836603894	AND Enantiomer 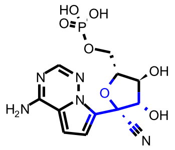 [*][C@H]1[*][*]O[C@]1(C#[*])[c](:[*]):[*]	-0.543	0 out of 2

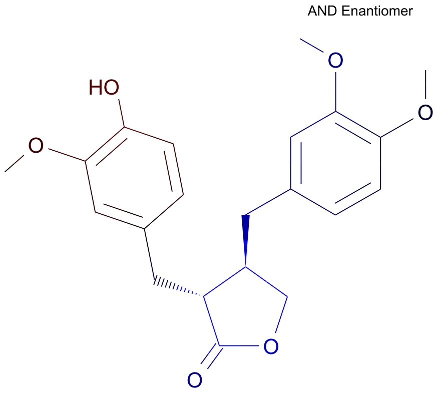

$C_{21}H_{24}O_6$
Molecular Weight: 372.41166
ALogP: 3.743
Rotatable Bonds: 7
Acceptors: 6
Donors: 1

Model Prediction

Prediction: Moderate
Probability: 0.234
Enrichment: 0.529
Bayesian Score: -7.65
Mahalanobis Distance: 10.8
Mahalanobis Distance p-value: 0.000133

Prediction: Positive if the Bayesian score is above the estimated best cutoff value from minimizing the false positive and false negative rate.
Probability: The esimated probability that the sample is in the positive category. This assumes that the Bayesian score follows a normal distribution and is different from the prediction using a cutoff.
Enrichment: An estimate of enrichment, that is, the increased likelihood (versus random) of this sample being in the category.
Bayesian Score: The standard Laplacian-modified Bayesian score.
Mahalanobis Distance: The Mahalanobis distance (MD) is the distance to the center of the training data. The larger the MD, the less trustworthy the prediction.
Mahalanobis Distance p-value: The p-value gives the fraction of training data with an MD greater than or equal to the one for the given sample, assuming normally distributed data. The smaller the p-value, the less trustworthy the prediciton. For highly non-normal X properties (e.g., fingerprints), the MD p-value is wildly inaccurate.

Structural Similar Compounds

Name	1-Piperazineacetic acid, 4-(2-hydroxyethyl)-alpha-phenyl-, 2,6-xyly l ester, monohydrochloride	Phosphorothioic acid, O-ethyl S-propyl O-(2,4,6-trichlorophenyl) es ter	Benzoic acid, p-amidino-, propyl ester
Structure	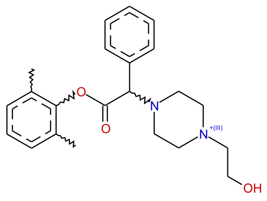	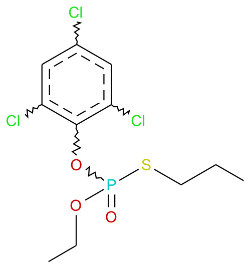	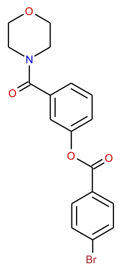
Actual Endpoint	Severe	Moderate	Severe
Predicted Endpoint	Severe	Moderate	Severe
Distance	0.775	0.873	0.890
Reference	BCFAAI Bollettino Chimico Farmaceutico. (Societa Editoriale Farmaceutica, Vi a Ausonio 12, 20123 Milan, Italy) V.33- 1894- Volume(issue)/page/year: 107,3 10,1968	NTIS** National Technical Information Service. (Springfield, VA 22161) Forme rly U.S. Clearinghouse for Scientific & Technical Information. Volume(issue)/pag e/year: OTS0535844	JAPMA8 Journal of the American Pharmaceutical Association, Scientific Edition . (Washington, DC) V.29-49, 1940-60. For publisher information, see JPMSAE. V olume(issue)/page/year: 41,202,1952

Model Applicability

Unknown features are fingerprint features in the query molecule, but not found or appearing too infrequently in the training set.

1. All properties and OPS components are within expected ranges.

Feature Contribution

Top features for positive contribution				
Fingerprint	Bit/Smiles	Feature Structure	Score	Severe in training set

SCFP_12	2116304939	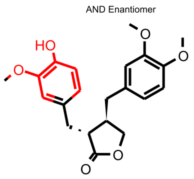 [*]O[c]1:[cH]:[*]:[cH]:[cH]:[c]:1O	0.59	5 out of 6
SCFP_12	-424425761	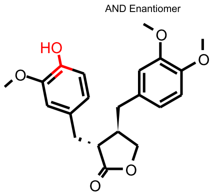 [*]:[c](:[*])O	0.582	23 out of 32
SCFP_12	1570454387	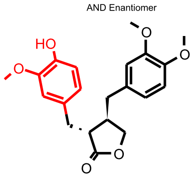 [*][c]1:[cH]:[cH]:[c] (O):[c](OC):[cH]:1	0.327	2 out of 3
**Top Features for negative contribution**				
**Fingerprint**	**Bit/Smiles**	**Feature Structure**	**Score**	**Severe in training set**
SCFP_12	-1031053674	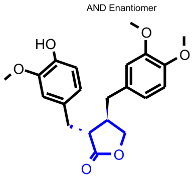 [*][C@H]1COC(=O)[C@@H]1[*]	-1.32	3 out of 36
SCFP_12	795806223	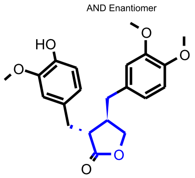 [*]C[C@H]1COC(=[*])[C @@H]1[*]	-0.936	0 out of 4

SCFP_12	276223760	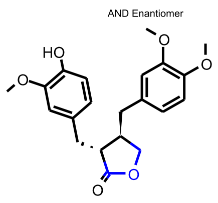 [*]=C1[*][*]CO1	-0.802	25 out of 147

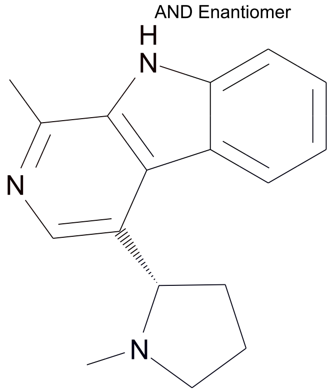

$C_{17}H_{19}N_3$
Molecular Weight: 265.35286
ALogP: 3.018
Rotatable Bonds: 1
Acceptors: 2
Donors: 1

Model Prediction

Prediction: Irritant
Probability: 0.977
Enrichment: 1.06
Bayesian Score: -0.289
Mahalanobis Distance: 7.45
Mahalanobis Distance p-value: 0.977

Prediction: Positive if the Bayesian score is above the estimated best cutoff value from minimizing the false positive and false negative rate.
Probability: The esimated probability that the sample is in the positive category. This assumes that the Bayesian score follows a normal distribution and is different from the prediction using a cutoff.
Enrichment: An estimate of enrichment, that is, the increased likelihood (versus random) of this sample being in the category.
Bayesian Score: The standard Laplacian-modified Bayesian score.
Mahalanobis Distance: The Mahalanobis distance (MD) is the distance to the center of the training data. The larger the MD, the less trustworthy the prediction.
Mahalanobis Distance p-value: The p-value gives the fraction of training data with an MD greater than or equal to the one for the given sample, assuming normally distributed data. The smaller the p-value, the less trustworthy the prediciton. For highly non-normal X properties (e.g., fingerprints), the MD p-value is wildly inaccurate.

Structural Similar Compounds

Name	Carbamic acid, methyl-, 1-(5,6,7,8-tetrahydro)naphthyl ester	Piperazine, 1-phenyl-	Carbamic acid, methyl-, 1-naphthyl ester
Structure	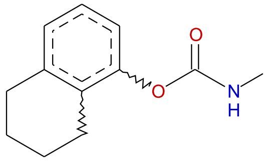	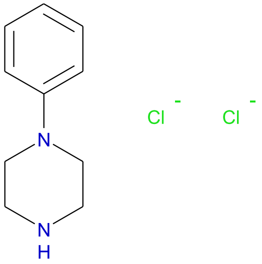	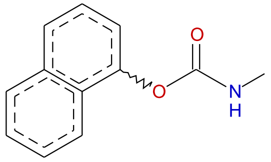
Actual Endpoint	Non-Irritant	Irritant	Irritant
Predicted Endpoint	Non-Irritant	Irritant	Non-Irritant
Distance	0.629	0.629	0.640
Reference	28ZPAK -,499,72	85JCAE "Prehled Prumyslove Toxikologie; Organicke Latky," Marhold, J., Prague , Czechoslovakia, Avicenum, 1986 Volume(issue)/page/year: -,868,1986	JAFCAU Journal of Agricultural and Food Chemistry. (American Chemical Soc., Distribution Office Dept. 223, POB 57136, West End Stn., Washington, DC 20037) V.1- 1953- Volume(issue)/page/year: 9,30,1961

Model Applicability

Unknown features are fingerprint features in the query molecule, but not found or appearing too infrequently in the training set.

1. All properties and OPS components are within expected ranges.

Feature Contribution

Top features for positive contribution				
**Fingerprint**	**Bit/Smiles**	**Feature Structure**	**Score**	**Irritant in training set**

FCFP_12	-124655670	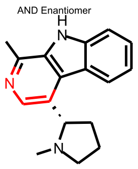 [*][c](:[*]):[cH]:n:[*]	0.0821	13 out of 13
FCFP_12	-1539132615	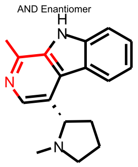 [*]:n:[c](C):[c](:[*]):[*]	0.0795	9 out of 9
FCFP_12	-1462709112	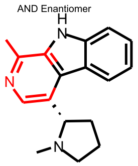 [*][c]1:[*]:[c](:[*]):[c](C):n:[cH]:1	0.0756	6 out of 6

Top Features for negative contribution

**Fingerprint**	**Bit/Smiles**	**Feature Structure**	**Score**	**Irritant in training set**
FCFP_12	309602933	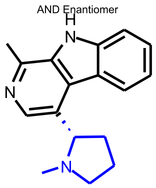 [*][C@@H]1CCCN1C	-0.205	11 out of 15
FCFP_12	240509252	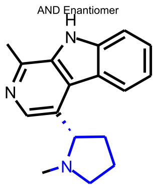 [*][C@@H]1CCCN1[*]	-0.159	10 out of 13

FCFP_12	-1320007763	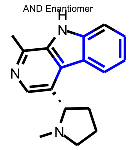 [*]:[c]1:[*]:[*]:[c]2:[*]:[cH]:[cH]:[cH]:[c]:1:2	-0.0893	20 out of 24

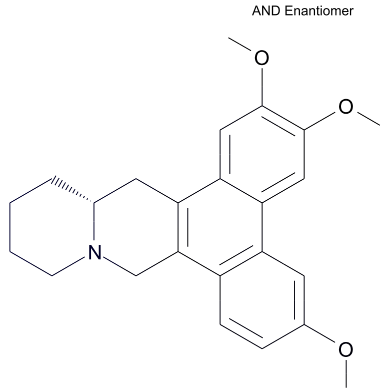

$C_{24}H_{27}NO_3$
Molecular Weight: 377.47608
ALogP: 4.691
Rotatable Bonds: 3
Acceptors: 4
Donors: 0

Model Prediction

Prediction: Non-Irritant
Probability: 0.958
Enrichment: 1.04
Bayesian Score: -1.61
Mahalanobis Distance: 9.83
Mahalanobis Distance p-value: 0.0945

Prediction: Positive if the Bayesian score is above the estimated best cutoff value from minimizing the false positive and false negative rate.
Probability: The esimated probability that the sample is in the positive category. This assumes that the Bayesian score follows a normal distribution and is different from the prediction using a cutoff.
Enrichment: An estimate of enrichment, that is, the increased likelihood (versus random) of this sample being in the category.
Bayesian Score: The standard Laplacian-modified Bayesian score.
Mahalanobis Distance: The Mahalanobis distance (MD) is the distance to the center of the training data. The larger the MD, the less trustworthy the prediction.
Mahalanobis Distance p-value: The p-value gives the fraction of training data with an MD greater than or equal to the one for the given sample, assuming normally distributed data. The smaller the p-value, the less trustworthy the prediciton. For highly non-normal X properties (e.g., fingerprints), the MD p-value is wildly inaccurate.

Structural Similar Compounds

Name	1-Piperazineacetic acid, alpha,4-dimethyl-, 2,6-diethylphenyl ester , dihydrochloride	1-Piperazineacetic acid, 4-methyl-, 2,6-diethylphenyl ester, dihydr ochloride	1-Piperazineacetic acid, alpha,4-dimethyl-, 2,6-diisopropylphenyl e ster, dihydrochloride
Structure	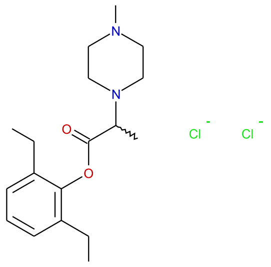	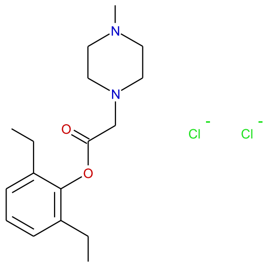	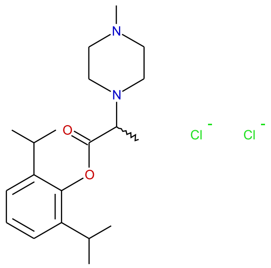
Actual Endpoint	Irritant	Irritant	Irritant
Predicted Endpoint	Irritant	Irritant	Irritant
Distance	0.587	0.601	0.611
Reference	BCFAAI Bollettino Chimico Farmaceutico. (Societa Editoriale Farmaceutica, Vi a Ausonio 12, 20123 Milan, Italy) V.33- 1894- Volume(issue)/page/year: 107,3 10,1968	BCFAAI Bollettino Chimico Farmaceutico. (Societa Editoriale Farmaceutica, Vi a Ausonio 12, 20123 Milan, Italy) V.33- 1894- Volume(issue)/page/year: 107,3 10,1968	BCFAAI Bollettino Chimico Farmaceutico. (Societa Editoriale Farmaceutica, Vi a Ausonio 12, 20123 Milan, Italy) V.33- 1894- Volume(issue)/page/year: 107,3 10,1968

Model Applicability

Unknown features are fingerprint features in the query molecule, but not found or appearing too infrequently in the training set.

1. All properties and OPS components are within expected ranges.

Feature Contribution

Top features for positive contribution				
**Fingerprint**	**Bit/Smiles**	**Feature Structure**	**Score**	**Irritant in training set**

FCFP_12	906798516	AND Enantiomer 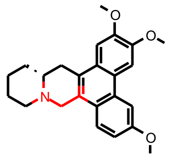 [*]N([*])C[c](:[*]):[*]	0.0703	4 out of 4
FCFP_12	1848358530	AND Enantiomer 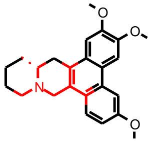 [*]CN1C[c](:[c](:[*]):[*]):[c](:[*])[*][C@H]1[*]	0.0658	3 out of 3
**Top Features for negative contribution**				
**Fingerprint**	**Bit/Smiles**	**Feature Structure**	**Score**	**Irritant in training set**
FCFP_12	-1235505740	AND Enantiomer 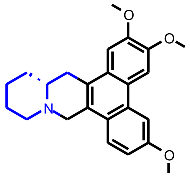 [*]C[C@H]1CCCCN1[*]	-0.222	2 out of 3
FCFP_12	-1866659497	AND Enantiomer 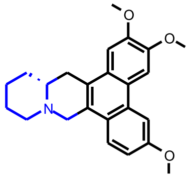 [*]CN1CCCC[C@@H]1[*]	-0.21	5 out of 7
FCFP_12	309602933	AND Enantiomer 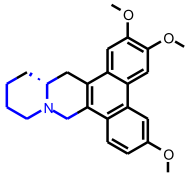 [*][C@@H]1CCCN1C	-0.205	11 out of 15

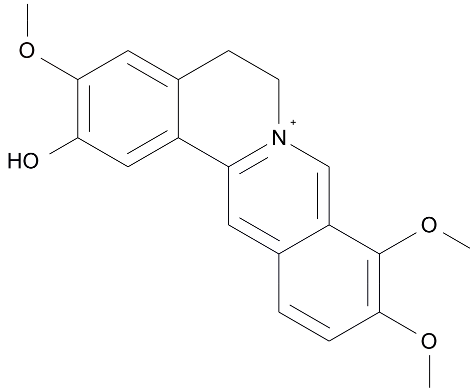

$C_{20}H_{20}NO_4$
Molecular Weight: 338.3771
ALogP: 3.936
Rotatable Bonds: 3
Acceptors: 4
Donors: 1

Structural Similar Compounds

Name	Aniline, 2,4-bis(o-methylphenoxy)-	s-Triazine, 2,4-dichloro-6-(o-chloroanilino)-	1-Piperazineacetic acid, 4-(2-hydroxyethyl)-alpha-phenyl-, 2,6-xyly l ester, monohydrochloride
Structure	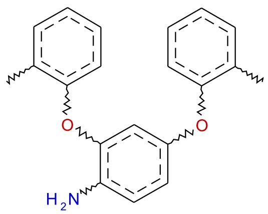	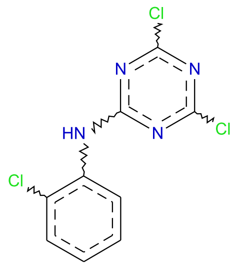	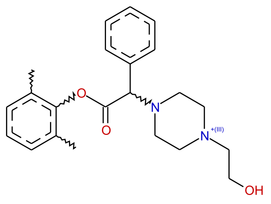
Actual Endpoint	Irritant	Irritant	Irritant
Predicted Endpoint	Non-Irritant	Non-Irritant	Irritant
Distance	0.649	0.657	0.667
Reference	85JCAE "Prehled Prumyslove Toxikologie; Organicke Latky," Marhold, J., Prague , Czechoslovakia, Avicenum, 1986 Volume(issue)/page/year: -,725,1986	34ZIAG* -,235,69	BCFAAI Bollettino Chimico Farmaceutico. (Societa Editoriale Farmaceutica, Vi a Ausonio 12, 20123 Milan, Italy) V.33- 1894- Volume(issue)/page/year: 107,3 10,1968

Model Prediction

Prediction: Non-Irritant
Probability: 0.97
Enrichment: 1.05
Bayesian Score: -1.01
Mahalanobis Distance: 7.12
Mahalanobis Distance p-value: 0.994

Prediction: Positive if the Bayesian score is above the estimated best cutoff value from minimizing the false positive and false negative rate.
Probability: The esimated probability that the sample is in the positive category. This assumes that the Bayesian score follows a normal distribution and is different from the prediction using a cutoff.
Enrichment: An estimate of enrichment, that is, the increased likelihood (versus random) of this sample being in the category.
Bayesian Score: The standard Laplacian-modified Bayesian score.
Mahalanobis Distance: The Mahalanobis distance (MD) is the distance to the center of the training data. The larger the MD, the less trustworthy the prediction.
Mahalanobis Distance p-value: The p-value gives the fraction of training data with an MD greater than or equal to the one for the given sample, assuming normally distributed data. The smaller the p-value, the less trustworthy the prediciton. For highly non-normal X properties (e.g., fingerprints), the MD p-value is wildly inaccurate.

Model Applicability

Unknown features are fingerprint features in the query molecule, but not found or appearing too infrequently in the training set.

1. All properties and OPS components are within expected ranges.
2. Unknown FCFP_2 feature: -150573739: [*]CC[n+](:[*]):[*]
3. Unknown FCFP_2 feature: -1861407456: [*][n+](:[*]):[c]([c](:[*]):[*]):c:[*]

Feature Contribution

Top features for positive contribution

Fingerprint	Bit/Smiles	Feature Structure	Score	Irritant in training set

FCFP_12	523826990	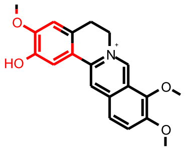 [*]O[c]1:[cH]:[*]:[c]([*]):[cH]:[c]:1O	0.0756	6 out of 6
FCFP_12	301073077	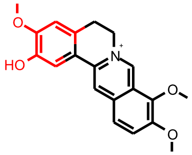 [*][c]1:[*]:[cH]:[c](O):[c](OC):[cH]:1	0.0734	5 out of 5
FCFP_12	715979708	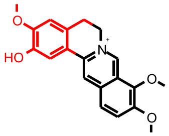 [*]C[c]1:[cH]:[c](OC):[c](O):[cH]:[c]:1[*]	0.0703	4 out of 4
**Top Features for negative contribution**				
**Fingerprint**	**Bit/Smiles**	**Feature Structure**	**Score**	**Irritant in training set**
FCFP_12	-1861645784	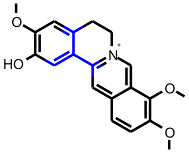 [*][c](:[*]):[c](:[cH]:[*])[c](:[*]):[*]	-0.125	12 out of 15
FCFP_12	7	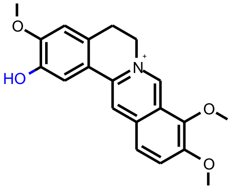 [*]O	-0.118	104 out of 128

FCFP_12	-549108873	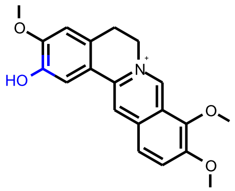 [*]:[c](:[*])O	-0.11	54 out of 66

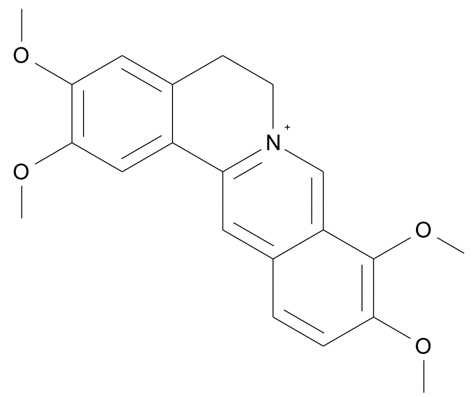

$C_{21}H_{22}NO_4$
Molecular Weight: 352.40368
ALogP: 4.161
Rotatable Bonds: 4
Acceptors: 4
Donors: 0

Structural Similar Compounds

Name	1-Piperazineacetic acid, 4-methyl-, 2,6-diethylphenyl ester, dihydr ochloride	1-Piperazineacetic acid, alpha,4-dimethyl-, 2,6-diethylphenyl ester , dihydrochloride	Anthranilic acid, N-(3-(p-tert-butylphenyl)-2-methylpropylidene)-, methyl ester
Structure	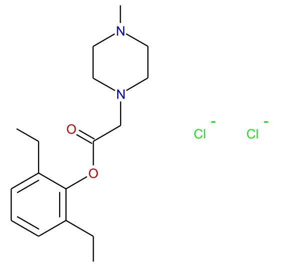	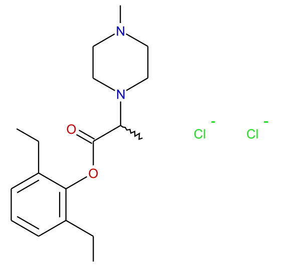	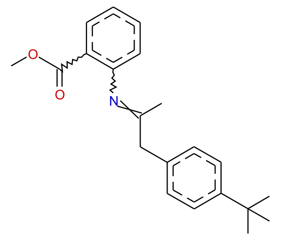
Actual Endpoint	Irritant	Irritant	Irritant
Predicted Endpoint	Irritant	Irritant	Irritant
Distance	0.578	0.599	0.621
Reference	BCFAAI Bollettino Chimico Farmaceutico. (Societa Editoriale Farmaceutica, Vi a Ausonio 12, 20123 Milan, Italy) V.33- 1894- Volume(issue)/page/year: 107,3 10,1968	BCFAAI Bollettino Chimico Farmaceutico. (Societa Editoriale Farmaceutica, Vi a Ausonio 12, 20123 Milan, Italy) V.33- 1894- Volume(issue)/page/year: 107,3 10,1968	FCTOD7 Food and Chemical Toxicology. (Pergamon Press Inc., Maxwell House, Fa irview Park, Elmsford, NY 10523) V.20- 1982- Volume(issue)/page/year: 20,729 ,1982

Model Prediction

Prediction: Irritant
Probability: 0.974
Enrichment: 1.06
Bayesian Score: -0.643
Mahalanobis Distance: 6.16
Mahalanobis Distance p-value: 1

Prediction: Positive if the Bayesian score is above the estimated best cutoff value from minimizing the false positive and false negative rate.
Probability: The esimated probability that the sample is in the positive category. This assumes that the Bayesian score follows a normal distribution and is different from the prediction using a cutoff.
Enrichment: An estimate of enrichment, that is, the increased likelihood (versus random) of this sample being in the category.
Bayesian Score: The standard Laplacian-modified Bayesian score.
Mahalanobis Distance: The Mahalanobis distance (MD) is the distance to the center of the training data. The larger the MD, the less trustworthy the prediction.
Mahalanobis Distance p-value: The p-value gives the fraction of training data with an MD greater than or equal to the one for the given sample, assuming normally distributed data. The smaller the p-value, the less trustworthy the prediciton. For highly non-normal X properties (e.g., fingerprints), the MD p-value is wildly inaccurate.

Model Applicability

Unknown features are fingerprint features in the query molecule, but not found or appearing too infrequently in the training set.

1. All properties and OPS components are within expected ranges.
2. Unknown FCFP_2 feature: -150573739: [*]CC[n+](:[*]):[*]
3. Unknown FCFP_2 feature: -1861407456: [*][n+](:[*]):[c]([c](:[*]):[*]):c:[*]

Feature Contribution

Top features for positive contribution

Fingerprint	Bit/Smiles	Feature Structure	Score	Irritant in training set

FCFP_12	-1405834164	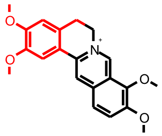 [*]C[c]1:[cH]:[c](OC):[c](OC):[cH]:[c]:1[*]	0.0734	5 out of 5
**Top Features for negative contribution**				
**Fingerprint**	**Bit/Smiles**	**Feature Structure**	**Score**	**Irritant in training set**
FCFP_12	-1861645784	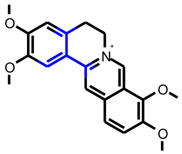 [*][c](:[*]):[c](:[cH]:[*])[c](:[*]):[*]	-0.125	12 out of 15
FCFP_12	-1320007763	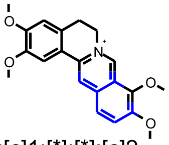 [*]:[c]1:[*]:[*]:[c]2:[*]:[cH]:[cH]:[cH]:[c]:1:2	-0.0893	20 out of 24
FCFP_12	1674451008	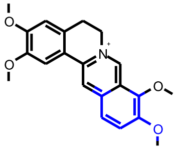 [*]O[c]1:[cH]:[*]:[c](:[*]):[cH]:[cH]:1	-0.0873	93 out of 111

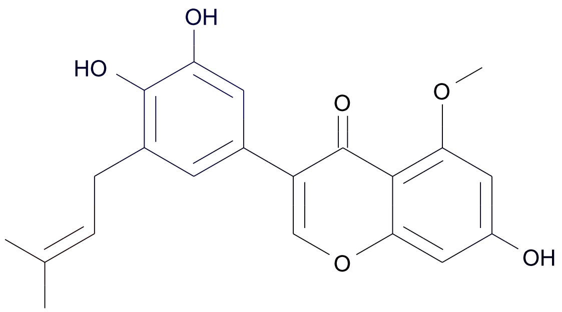

$C_{21}H_{20}O_6$
Molecular Weight: 368.3799
ALogP: 3.98
Rotatable Bonds: 4
Acceptors: 6
Donors: 3

Model Prediction

Prediction: Non-Irritant
Probability: 0.952
Enrichment: 1.03
Bayesian Score: -1.81
Mahalanobis Distance: 13.7
Mahalanobis Distance p-value: 7.94e-010

Prediction: Positive if the Bayesian score is above the estimated best cutoff value from minimizing the false positive and false negative rate.
Probability: The esimated probability that the sample is in the positive category. This assumes that the Bayesian score follows a normal distribution and is different from the prediction using a cutoff.
Enrichment: An estimate of enrichment, that is, the increased likelihood (versus random) of this sample being in the category.
Bayesian Score: The standard Laplacian-modified Bayesian score.
Mahalanobis Distance: The Mahalanobis distance (MD) is the distance to the center of the training data. The larger the MD, the less trustworthy the prediction.
Mahalanobis Distance p-value: The p-value gives the fraction of training data with an MD greater than or equal to the one for the given sample, assuming normally distributed data. The smaller the p-value, the less trustworthy the prediciton. For highly non-normal X properties (e.g., fingerprints), the MD p-value is wildly inaccurate.

Structural Similar Compounds

Name	5-Norbornene-2,3-dicarboxylic acid, 1,4,5,6,7,7-hexachloro-	1-Amino-2-bromo-4-hydroxyanthraquinone	8-Methylamino-4-hydroxy-2-naphthalene sulfonic acid
Structure	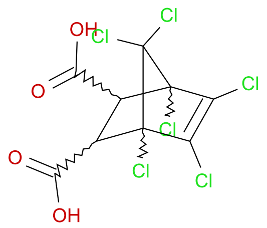	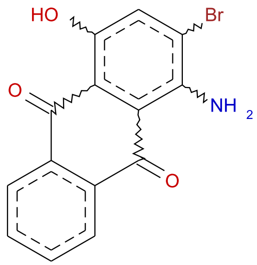	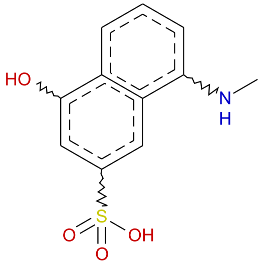
Actual Endpoint	Irritant	Non-Irritant	Non-Irritant
Predicted Endpoint	Irritant	Non-Irritant	Non-Irritant
Distance	0.827	0.853	0.854
Reference	85JCAE "Prehled Prumyslove Toxikologie; Organicke Latky," Marhold, J., Prague , Czechoslovakia, Avicenum, 1986 Volume(issue)/page/year: -,581,1986	28ZPAK -,83,72	28ZPAK -,190,72

Model Applicability

Unknown features are fingerprint features in the query molecule, but not found or appearing too infrequently in the training set.

1. All properties and OPS components are within expected ranges.

Feature Contribution

Top features for positive contribution				
Fingerprint	Bit/Smiles	Feature Structure	Score	Irritant in training set

FCFP_12	-1582522951	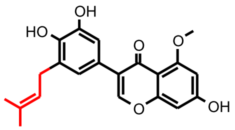 [*]CC=C(C)C	0.0868	48 out of 48
FCFP_12	451877515	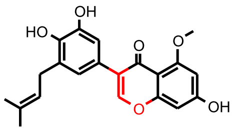 [*]OC=C([*])[*]	0.0821	13 out of 13
FCFP_12	436886043	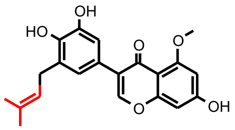 [*]C=C(C)C	0.0804	129 out of 130
**Top Features for negative contribution**				
**Fingerprint**	**Bit/Smiles**	**Feature Structure**	**Score**	**Irritant in training set**
FCFP_12	-201608392	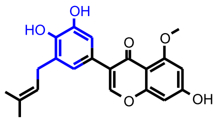 [*]C[c]1:[cH]:[*]:[cH]:[c](O):[c]:1O	-0.65	0 out of 1
FCFP_12	949015626	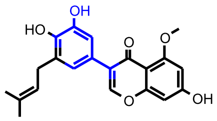 [*]C(=[*])[c]1:[cH]:[*]:[c]([*]):[c](O):[cH]:1	-0.222	2 out of 3

FCFP_12	-204034463	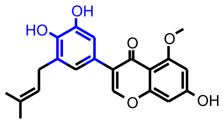 [*][c]1:[*]:[c]([*]):[c](O):[c](O):[cH]:1	-0.222	2 out of 3

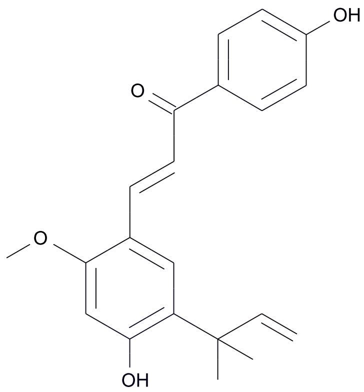

$C_{21}H_{22}O_4$
Molecular Weight: 338.39698
ALogP: 4.667
Rotatable Bonds: 6
Acceptors: 4
Donors: 2

Structural Similar Compounds

Name	Sulfide, bis(4-t-butyl-m-cresyl)-	1-Piperazineacetic acid, 4-(2-hydroxyethyl)-alpha-phenyl-, 2,6-xyly l ester, monohydrochloride	Aniline, 2,4-bis(o-methylphenoxy)-
Structure	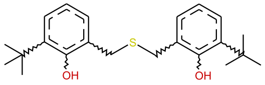	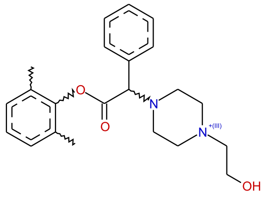	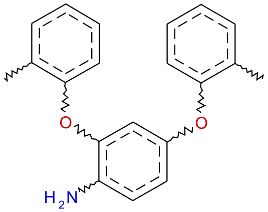
Actual Endpoint	Irritant	Irritant	Irritant
Predicted Endpoint	Irritant	Irritant	Non-Irritant
Distance	0.623	0.713	0.756
Reference	AMIHBC AMA Archives of Industrial Hygiene and Occupational Medicine. (Chicago , IL) V.2-10, 1950-54. For publisher information, see AEHLAU. Volume(issue)/pag e/year: 5,311,1952	BCFAAI Bollettino Chimico Farmaceutico. (Societa Editoriale Farmaceutica, Vi a Ausonio 12, 20123 Milan, Italy) V.33- 1894- Volume(issue)/page/year: 107,3 10,1968	85JCAE "Prehled Prumyslove Toxikologie; Organicke Latky," Marhold, J., Prague , Czechoslovakia, Avicenum, 1986 Volume(issue)/page/year: -,725,1986

Model Prediction

Prediction: Non-Irritant
Probability: 0.962
Enrichment: 1.04
Bayesian Score: -1.45
Mahalanobis Distance: 8.54
Mahalanobis Distance p-value: 0.667

Prediction: Positive if the Bayesian score is above the estimated best cutoff value from minimizing the false positive and false negative rate.
Probability: The esimated probability that the sample is in the positive category. This assumes that the Bayesian score follows a normal distribution and is different from the prediction using a cutoff.
Enrichment: An estimate of enrichment, that is, the increased likelihood (versus random) of this sample being in the category.
Bayesian Score: The standard Laplacian-modified Bayesian score.
Mahalanobis Distance: The Mahalanobis distance (MD) is the distance to the center of the training data. The larger the MD, the less trustworthy the prediction.
Mahalanobis Distance p-value: The p-value gives the fraction of training data with an MD greater than or equal to the one for the given sample, assuming normally distributed data. The smaller the p-value, the less trustworthy the prediciton. For highly non-normal X properties (e.g., fingerprints), the MD p-value is wildly inaccurate.

Model Applicability

Unknown features are fingerprint features in the query molecule, but not found or appearing too infrequently in the training set.

1. All properties and OPS components are within expected ranges.

Feature Contribution

Top features for positive contribution

Fingerprint	Bit/Smiles	Feature Structure	Score	Irritant in training set

FCFP_12	-146015125	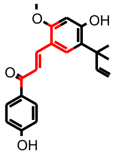 [*]C(=[*])\C=C\[c](:[cH]:[*]):[c]([*]):[*]	0.085	24 out of 24
FCFP_12	-836633685	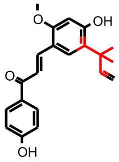 [*]=CC(C)(C)[c](:[*]):[*]	0.0829	15 out of 15
FCFP_12	451847724	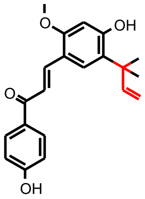 [*]CC=C([*])[*]	0.0737	270 out of 274
**Top Features for negative contribution**				
**Fingerprint**	**Bit/Smiles**	**Feature Structure**	**Score**	**Irritant in training set**
FCFP_12	-1604301295	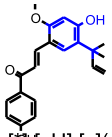 [*][c]1:[*]:[cH]:[c](:[c](O):[cH]:1)C([*])([*])[*]	-0.18	22 out of 29
FCFP_12	-451251206	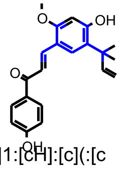 [*]C[c]1:[cH]:[c](:[cH]:[*]:[c]:1[*])C(=[*])[*]	-0.132	44 out of 55

FCFP_12	7	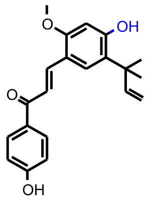 [*]O	-0.118	104 out of 128

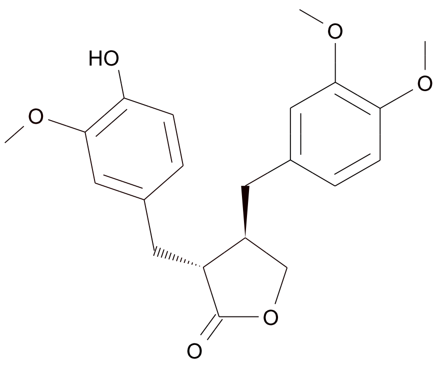

$C_{21}H_{24}O_6$
Molecular Weight: 372.41166
ALogP: 3.743
Rotatable Bonds: 7
Acceptors: 6
Donors: 1

Model Prediction

Prediction: Irritant
Probability: 0.997
Enrichment: 1.08
Bayesian Score: 0.306
Mahalanobis Distance: 9.52
Mahalanobis Distance p-value: 0.183

Prediction: Positive if the Bayesian score is above the estimated best cutoff value from minimizing the false positive and false negative rate.
Probability: The esimated probability that the sample is in the positive category. This assumes that the Bayesian score follows a normal distribution and is different from the prediction using a cutoff.
Enrichment: An estimate of enrichment, that is, the increased likelihood (versus random) of this sample being in the category.
Bayesian Score: The standard Laplacian-modified Bayesian score.
Mahalanobis Distance: The Mahalanobis distance (MD) is the distance to the center of the training data. The larger the MD, the less trustworthy the prediction.
Mahalanobis Distance p-value: The p-value gives the fraction of training data with an MD greater than or equal to the one for the given sample, assuming normally distributed data. The smaller the p-value, the less trustworthy the prediciton. For highly non-normal X properties (e.g., fingerprints), the MD p-value is wildly inaccurate.

Structural Similar Compounds

Name	1-Piperazineacetic acid, 4-(2-hydroxyethyl)-alpha-phenyl-, 2,6-xyly l ester, monohydrochloride	1,4-Pentadien-3-one, 1,5-bis(p-azidophenyl)-	Diethylene glycol, dibenzoate
Structure	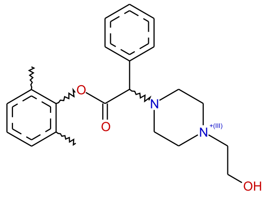	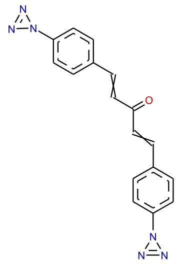	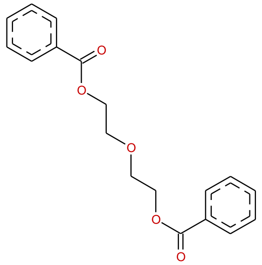
Actual Endpoint	Irritant	Irritant	Irritant
Predicted Endpoint	Irritant	Irritant	Irritant
Distance	0.694	0.738	0.760
Reference	BCFAAI Bollettino Chimico Farmaceutico. (Societa Editoriale Farmaceutica, Vi a Ausonio 12, 20123 Milan, Italy) V.33- 1894- Volume(issue)/page/year: 107,3 10,1968	85JCAE "Prehled Prumyslove Toxikologie; Organicke Latky," Marhold, J., Prague , Czechoslovakia, Avicenum, 1986 Volume(issue)/page/year: -,733,1986	85JCAE "Prehled Prumyslove Toxikologie; Organicke Latky," Marhold, J., Prague , Czechoslovakia, Avicenum, 1986 Volume(issue)/page/year: -,716,1986

Model Applicability

Unknown features are fingerprint features in the query molecule, but not found or appearing too infrequently in the training set.

1. All properties and OPS components are within expected ranges.

Feature Contribution

Top features for positive contribution

Fingerprint	Bit/Smiles	Feature Structure	Score	Irritant in training set

FCFP_12	1588282714	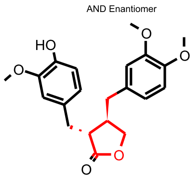<br>[*]C[C@H]1COC(=[*])[C<br>@@H]1[*]	0.0855	28 out of 28
FCFP_12	-1038421835	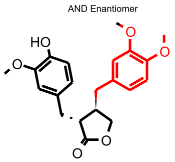<br>[*]C[c]1:[cH]:[cH]:[c<br>](OC):[c](O[*]):[cH]<br>:1	0.0795	9 out of 9
FCFP_12	1985089045	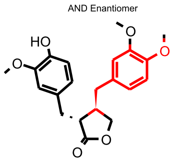<br>[*]O[c]1:[cH]:[cH]:[c<br>](CC([*])[*]):[cH]:[<br>c]:1[*]	0.0785	8 out of 8
**Top Features for negative contribution**				
**Fingerprint**	**Bit/Smiles**	**Feature Structure**	**Score**	**Irritant in training set**
FCFP_12	7	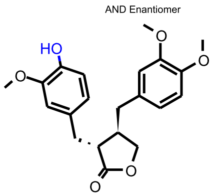<br>[*]O	-0.118	104 out of 128
FCFP_12	-549108873	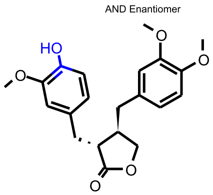<br>[*]:[c](:[*])O	-0.11	54 out of 66

FCFP_12	74595001	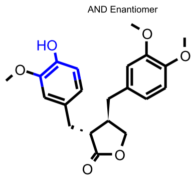 [*][c](:[*]):[c](O):[cH]:[*]	-0.11	54 out of 66

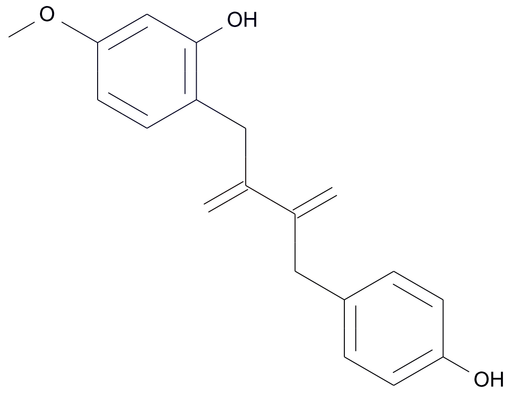

$C_{19}H_{20}O_3$
Molecular Weight: 296.3603
ALogP: 4.784
Rotatable Bonds: 6
Acceptors: 3
Donors: 2

Model Prediction

Prediction: Non-Irritant
Probability: 0.967
Enrichment: 1.05
Bayesian Score: -1.19
Mahalanobis Distance: 9.63
Mahalanobis Distance p-value: 0.148

Prediction: Positive if the Bayesian score is above the estimated best cutoff value from minimizing the false positive and false negative rate.
Probability: The esimated probability that the sample is in the positive category. This assumes that the Bayesian score follows a normal distribution and is different from the prediction using a cutoff.
Enrichment: An estimate of enrichment, that is, the increased likelihood (versus random) of this sample being in the category.
Bayesian Score: The standard Laplacian-modified Bayesian score.
Mahalanobis Distance: The Mahalanobis distance (MD) is the distance to the center of the training data. The larger the MD, the less trustworthy the prediction.
Mahalanobis Distance p-value: The p-value gives the fraction of training data with an MD greater than or equal to the one for the given sample, assuming normally distributed data. The smaller the p-value, the less trustworthy the prediciton. For highly non-normal X properties (e.g., fingerprints), the MD p-value is wildly inaccurate.

Structural Similar Compounds

Name	Sulfide, bis(4-t-butyl-m-cresyl)-	Phenol, 2,2'-methylenebis(4-chloro-	Phenol, 4,4'-isopropylidenedi-
Structure	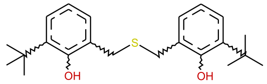	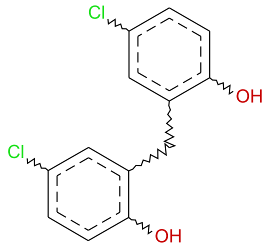	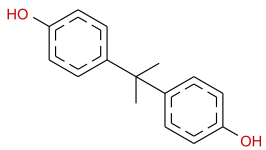
Actual Endpoint	Irritant	Irritant	Irritant
Predicted Endpoint	Irritant	Non-Irritant	Non-Irritant
Distance	0.656	0.658	0.684
Reference	AMIHBC AMA Archives of Industrial Hygiene and Occupational Medicine. (Chicago , IL) V.2-10, 1950-54. For publisher information, see AEHLAU. Volume(issue)/pag e/year: 5,311,1952	85JCAE "Prehled Prumyslove Toxikologie; Organicke Latky," Marhold, J., Prague , Czechoslovakia, Avicenum, 1986 Volume(issue)/page/year: -,533,1986	85JCAE "Prehled Prumyslove Toxikologie; Organicke Latky," Marhold, J., Prague , Czechoslovakia, Avicenum, 1986 Volume(issue)/page/year: -,238,1986

Model Applicability

Unknown features are fingerprint features in the query molecule, but not found or appearing too infrequently in the training set.

1. All properties and OPS components are within expected ranges.

Feature Contribution

Top features for positive contribution

Fingerprint	Bit/Smiles	Feature Structure	Score	Irritant in training set
FCFP_12	436886043	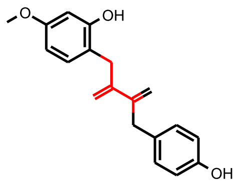 [*]C=C(C)C	0.0804	129 out of 130

FCFP_12	1985639687	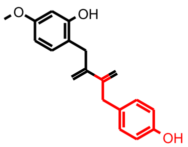<br>[*]C([*])C[c]1:[cH]:[<br>cH]:[c](O):[c]([*]):<br>[cH]:1	0.0703	4 out of 4
FCFP_12	-542873837	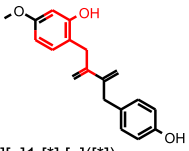<br>[*][c]1:[*]:[c]([*]):<br>[c](O):[c](CC=[*]):[<br>cH]:1	0.0658	3 out of 3

Top Features for negative contribution

**Fingerprint**	**Bit/Smiles**	**Feature Structure**	**Score**	**Irritant in training set**
FCFP_12	-1604301295	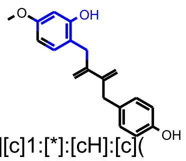<br>[*][c]1:[*]:[cH]:[c](<br>:[c](O):[cH]:1)C([*]<br>)([*])[*]	-0.18	22 out of 29
FCFP_12	7	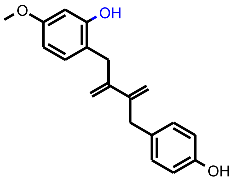<br>[*]O	-0.118	104 out of 128
FCFP_12	-549108873	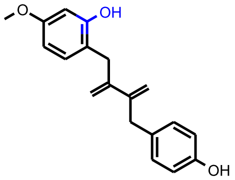<br>[*]:[c](:[*])O	-0.11	54 out of 66

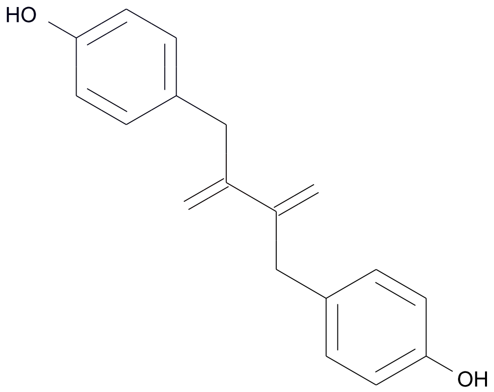

$C_{18}H_{18}O_2$

Molecular Weight: 266.33432

ALogP: 4.8

Rotatable Bonds: 5

Acceptors: 2

Donors: 2

Model Prediction

Prediction: Non-Irritant

Probability: 0.972

Enrichment: 1.06

Bayesian Score: -0.841

Mahalanobis Distance: 8.66

Mahalanobis Distance p-value: 0.605

Prediction: Positive if the Bayesian score is above the estimated best cutoff value from minimizing the false positive and false negative rate.
Probability: The esimated probability that the sample is in the positive category. This assumes that the Bayesian score follows a normal distribution and is different from the prediction using a cutoff.
Enrichment: An estimate of enrichment, that is, the increased likelihood (versus random) of this sample being in the category.
Bayesian Score: The standard Laplacian-modified Bayesian score.
Mahalanobis Distance: The Mahalanobis distance (MD) is the distance to the center of the training data. The larger the MD, the less trustworthy the prediction.
Mahalanobis Distance p-value: The p-value gives the fraction of training data with an MD greater than or equal to the one for the given sample, assuming normally distributed data. The smaller the p-value, the less trustworthy the prediciton. For highly non-normal X properties (e.g., fingerprints), the MD p-value is wildly inaccurate.

Structural Similar Compounds

Name	Phenol, 4,4'-isopropylidenedi-	Phenol, 2,2'-methylenebis(4-chloro-	p-Phenylenediamine, N,N-diphenyl-
Structure	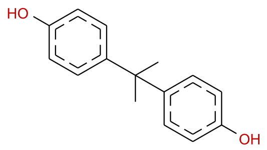	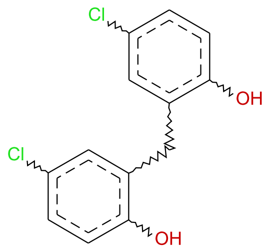	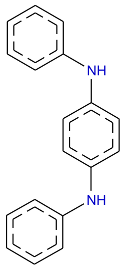
Actual Endpoint	Irritant	Irritant	Non-Irritant
Predicted Endpoint	Non-Irritant	Non-Irritant	Non-Irritant
Distance	0.544	0.557	0.599
Reference	85JCAE "Prehled Prumyslove Toxikologie; Organicke Latky," Marhold, J., Prague , Czechoslovakia, Avicenum, 1986 Volume(issue)/page/year: -,238,1986	85JCAE "Prehled Prumyslove Toxikologie; Organicke Latky," Marhold, J., Prague , Czechoslovakia, Avicenum, 1986 Volume(issue)/page/year: -,533,1986	28ZPAK -,73,72

Model Applicability

Unknown features are fingerprint features in the query molecule, but not found or appearing too infrequently in the training set.

1. All properties and OPS components are within expected ranges.

Feature Contribution

Top features for positive contribution

Fingerprint	Bit/Smiles	Feature Structure	Score	Irritant in training set
FCFP_12	436886043	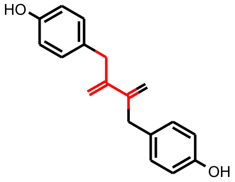 [*]C=C(C)C	0.0804	129 out of 130

FCFP_12	1985639687	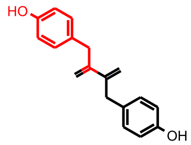 [*]C([*])C[c]1:[cH]:[cH]:[c](O):[c]([*]):[cH]:1	0.0703	4 out of 4
FCFP_12	129344189	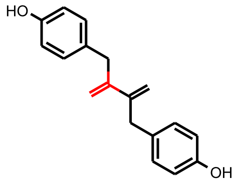 [*]C=C	0.056	122 out of 126

Top Features for negative contribution

Fingerprint	Bit/Smiles	Feature Structure	Score	Irritant in training set
FCFP_12	7	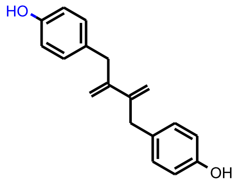 [*]O	-0.118	104 out of 128
FCFP_12	-549108873	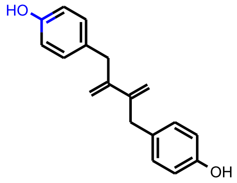 [*]:[c](:[*])O	-0.11	54 out of 66
FCFP_12	74595001	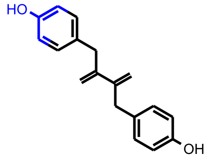 [*][c](:[*]):[c](O):[cH]:[*]	-0.11	54 out of 66

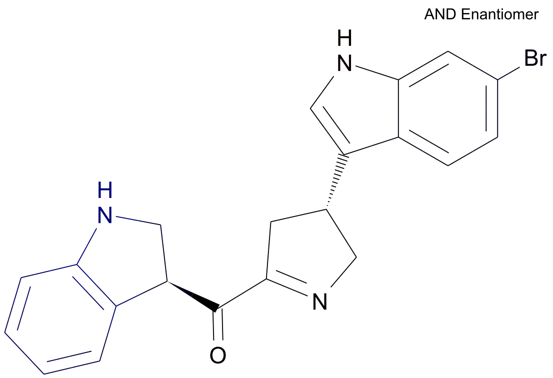

$C_{21}H_{18}BrN_3O$

Molecular Weight: 408.29112

ALogP: 3.919

Rotatable Bonds: 3

Acceptors: 3

Donors: 2

Model Prediction

Prediction: Non-Irritant

Probability: 0.854

Enrichment: 0.928

Bayesian Score: -3.17

Mahalanobis Distance: 10.6

Mahalanobis Distance p-value: 0.011

Prediction: Positive if the Bayesian score is above the estimated best cutoff value from minimizing the false positive and false negative rate.
Probability: The esimated probability that the sample is in the positive category. This assumes that the Bayesian score follows a normal distribution and is different from the prediction using a cutoff.
Enrichment: An estimate of enrichment, that is, the increased likelihood (versus random) of this sample being in the category.
Bayesian Score: The standard Laplacian-modified Bayesian score.
Mahalanobis Distance: The Mahalanobis distance (MD) is the distance to the center of the training data. The larger the MD, the less trustworthy the prediction.
Mahalanobis Distance p-value: The p-value gives the fraction of training data with an MD greater than or equal to the one for the given sample, assuming normally distributed data. The smaller the p-value, the less trustworthy the prediciton. For highly non-normal X properties (e.g., fingerprints), the MD p-value is wildly inaccurate.

Structural Similar Compounds

Name	1-Amino-2,4-dibromanthraquinone	5-Norbornene-2,3-dicarboxylic acid, 1,4,5,6,7,7-hexachloro-	Sulfide, bis(4-t-butyl-m-cresyl)-
Structure	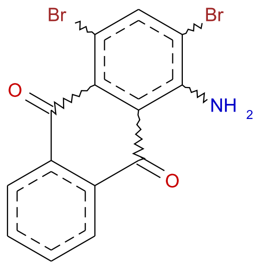	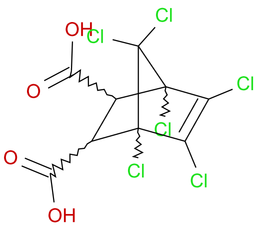	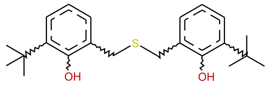
Actual Endpoint	Non-Irritant	Irritant	Irritant
Predicted Endpoint	Non-Irritant	Irritant	Irritant
Distance	0.709	0.710	0.753
Reference	28ZPAK -,88,72	85JCAE "Prehled Prumyslove Toxikologie; Organicke Latky," Marhold, J., Prague , Czechoslovakia, Avicenum, 1986 Volume(issue)/page/year: -,581,1986	AMIHBC AMA Archives of Industrial Hygiene and Occupational Medicine. (Chicago , IL) V.2-10, 1950-54. For publisher information, see AEHLAU. Volume(issue)/pag e/year: 5,311,1952

Model Applicability

Unknown features are fingerprint features in the query molecule, but not found or appearing too infrequently in the training set.

1. All properties and OPS components are within expected ranges.
2. Unknown FCFP_2 feature: 1618184456: [*][c]1:[*]:[*]:[nH]:c:1

Feature Contribution

Top features for positive contribution				
**Fingerprint**	**Bit/Smiles**	**Feature Structure**	**Score**	**Irritant in training set**

FCFP_12	1186303932	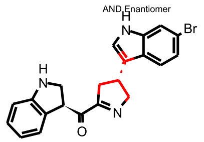 [*]:[c](:[*])C1C[*]=[*]C1	0.0838	18 out of 18
FCFP_12	580930443	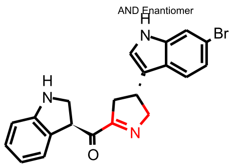 [*]C1=NC[*][*]1	0.0772	7 out of 7
FCFP_12	565968762	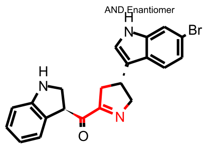 [*]C(=[*])C1=N[*][*]C 1	0.075	78 out of 79
**Top Features for negative contribution**				
**Fingerprint**	**Bit/Smiles**	**Feature Structure**	**Score**	**Irritant in training set**
FCFP_12	1294255210	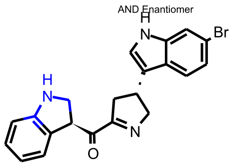 [*]:[c]1:[*][*]CN1	-0.486	12 out of 22
FCFP_12	-1724769936	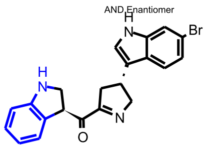 [*]1[*][c]2:[cH]:[cH] :[cH]:[cH]:[c]:2N1	-0.475	11 out of 20

FCFP_12	-773983804	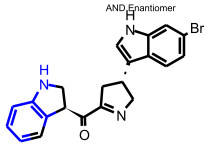<br>[*]1[*][c]2:[*]:[cH]:[cH]:[cH]:[c]:2N1	-0.444	46 out of 79

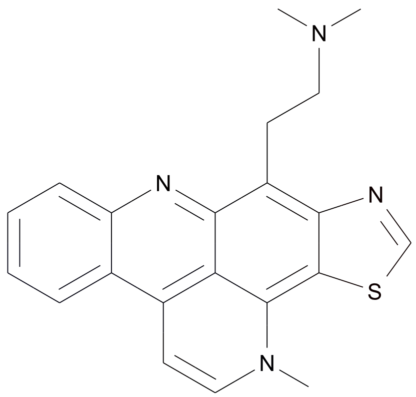

$C_{21}H_{20}N_4S$
Molecular Weight: 360.4753
ALogP: 3.682
Rotatable Bonds: 3
Acceptors: 4
Donors: 0

Structural Similar Compounds

Name	Benzoic acid, p-amidino-, propyl ester	Phosphoric acid, 1,2-dibromo-2,2-dichloroethyl dimethyl ester	Phosphorous acid, triphenyl ester
Structure	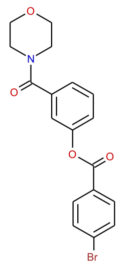	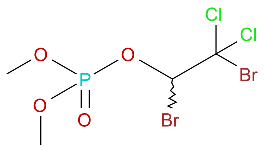	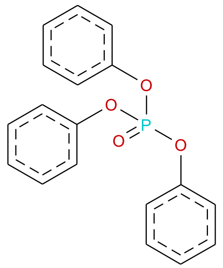
Actual Endpoint	Irritant	Irritant	Irritant
Predicted Endpoint	Non-Irritant	Irritant	Irritant
Distance	0.573	0.660	0.666
Reference	JAPMA8 Journal of the American Pharmaceutical Association, Scientific Edition . (Washington, DC) V.29-49, 1940-60. For publisher information, see JPMSAE. V olume(issue)/page/year: 41,202,1952	TXAPA9 Toxicology and Applied Pharmacology. (Academic Press, Inc., 1 E. Firs t St., Duluth, MN 55802) V.1- 1959- Volume(issue)/page/year: 21,369,1972	AMIHBC AMA Archives of Industrial Hygiene and Occupational Medicine. (Chicago , IL) V.2-10, 1950-54. For publisher information, see AEHLAU. Volume(issue)/pag e/year: 5,311,1952

Model Prediction

Prediction: Irritant
Probability: 0.976
Enrichment: 1.06
Bayesian Score: -0.454
Mahalanobis Distance: 8.09
Mahalanobis Distance p-value: 0.856

Prediction: Positive if the Bayesian score is above the estimated best cutoff value from minimizing the false positive and false negative rate.
Probability: The esimated probability that the sample is in the positive category. This assumes that the Bayesian score follows a normal distribution and is different from the prediction using a cutoff.
Enrichment: An estimate of enrichment, that is, the increased likelihood (versus random) of this sample being in the category.
Bayesian Score: The standard Laplacian-modified Bayesian score.
Mahalanobis Distance: The Mahalanobis distance (MD) is the distance to the center of the training data. The larger the MD, the less trustworthy the prediction.
Mahalanobis Distance p-value: The p-value gives the fraction of training data with an MD greater than or equal to the one for the given sample, assuming normally distributed data. The smaller the p-value, the less trustworthy the prediciton. For highly non-normal X properties (e.g., fingerprints), the MD p-value is wildly inaccurate.

Model Applicability

Unknown features are fingerprint features in the query molecule, but not found or appearing too infrequently in the training set.

1. All properties and OPS components are within expected ranges.

Feature Contribution

Top features for positive contribution				
Fingerprint	Bit/Smiles	Feature Structure	Score	Irritant in training set

FCFP_12	451877515	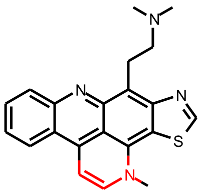 [*]OC=C([*])[*]	0.0821	13 out of 13
FCFP_12	-14048077	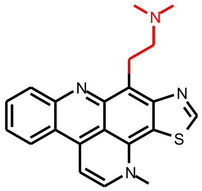 [*]CCN(C)C	0.0803	10 out of 10
FCFP_12	-124685461	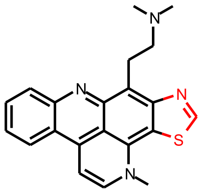 [*]1:[*]:s:[cH]:n:1	0.0734	5 out of 5
**Top Features for negative contribution**				
**Fingerprint**	**Bit/Smiles**	**Feature Structure**	**Score**	**Irritant in training set**
FCFP_12	-1320007763	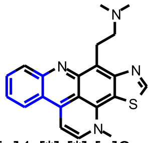 [*]:[c]1:[*]:[*]:[c]2 :[*]:[cH]:[cH]:[cH]: [c]:1:2	-0.0893	20 out of 24
FCFP_12	675769755	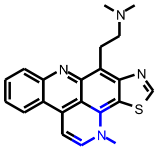 [*]=CN(C)[c](:[*]):[*]	-0.088	15 out of 18

FCFP_12	1618154665	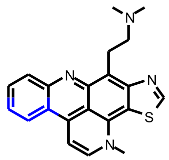 [*]:[cH]:[cH]:[cH]:[*]	-0.0845	412 out of 490

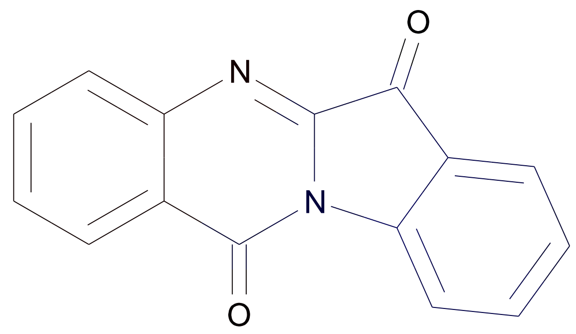

$C_{15}H_8N_2O_2$

Molecular Weight: 248.23621

ALogP: 2.331

Rotatable Bonds: 0

Acceptors: 3

Donors: 0

Model Prediction

Prediction: Non-Irritant

Probability: 0.961

Enrichment: 1.04

Bayesian Score: -1.49

Mahalanobis Distance: 5.6

Mahalanobis Distance p-value: 1

Prediction: Positive if the Bayesian score is above the estimated best cutoff value from minimizing the false positive and false negative rate.
Probability: The esimated probability that the sample is in the positive category. This assumes that the Bayesian score follows a normal distribution and is different from the prediction using a cutoff.
Enrichment: An estimate of enrichment, that is, the increased likelihood (versus random) of this sample being in the category.
Bayesian Score: The standard Laplacian-modified Bayesian score.
Mahalanobis Distance: The Mahalanobis distance (MD) is the distance to the center of the training data. The larger the MD, the less trustworthy the prediction.
Mahalanobis Distance p-value: The p-value gives the fraction of training data with an MD greater than or equal to the one for the given sample, assuming normally distributed data. The smaller the p-value, the less trustworthy the prediciton. For highly non-normal X properties (e.g., fingerprints), the MD p-value is wildly inaccurate.

Structural Similar Compounds

Name	3-Phosphabicyclo(4.4.0)decane, P-chloro-5,10-dimethyl-2,4-dioxa-P-thiono-	Thiophene, tetrahydro-3,3,4,4-tetrachloro-, 1,1-dioxide	Maleimide, N-(p-chlorophenyl)-
Structure	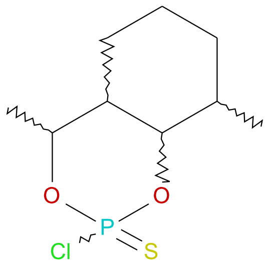	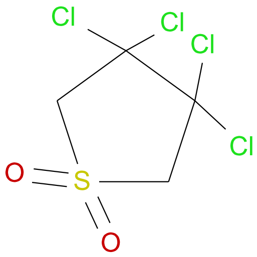	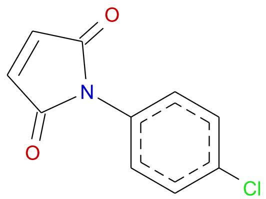
Actual Endpoint	Irritant	Irritant	Irritant
Predicted Endpoint	Irritant	Irritant	Non-Irritant
Distance	0.584	0.595	0.596
Reference	85JCAE "Prehled Prumyslove Toxikologie; Organicke Latky," Marhold, J., Prague , Czechoslovakia, Avicenum, 1986 Volume(issue)/page/year: -,1190,1986	NTIS** National Technical Information Service. (Springfield, VA 22161) Forme rly U.S. Clearinghouse for Scientific & Technical Information. Volume(issue)/pag e/year: OTS0555216	SCCUR* Shell Chemical Company. Unpublished Report. (2401 Crow Canyon Rd., S an Romon, CA 94583) Volume(issue)/page/year: -,7,1961

Model Applicability

Unknown features are fingerprint features in the query molecule, but not found or appearing too infrequently in the training set.

1. All properties and OPS components are within expected ranges.

Feature Contribution

Top features for positive contribution

Fingerprint	Bit/Smiles	Feature Structure	Score	Irritant in training set

FCFP_12	580453787	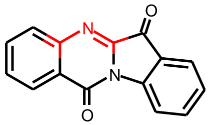<br>[*]C(=N[c](:[*]):[*])[*]	0.0795	9 out of 9
FCFP_12	1150094517	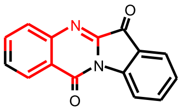<br>[*]C1=N[c]2:[cH]:[cH]:[*]:[cH]:[c]:2C(=[*])[*]1	0.0756	6 out of 6
FCFP_12	562091192	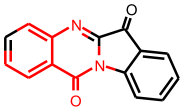<br>[*]N1[*]=N[c]2:[cH]:[*]:[cH]:[cH]:[c]:2C1=O	0.0756	6 out of 6
**Top Features for negative contribution**				
**Fingerprint**	**Bit/Smiles**	**Feature Structure**	**Score**	**Irritant in training set**
FCFP_12	-1432259023	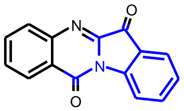<br>[*]C(=[*])N1C(=[*])C(=[*])[c]2:[cH]:[cH]:[cH]:[cH]:[c]1:2	-0.627	1 out of 3
FCFP_12	159265197	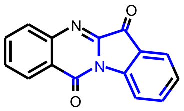<br>[*]C(=[*])N1C(=[*])C(=[*])[c]2:[cH]:[*]:[cH]:[cH]:[c]1:2	-0.44	2 out of 4

FCFP_12	675769755	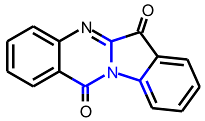 [*]=CN(C)[c](:[*]):[*]]	-0.088	15 out of 18

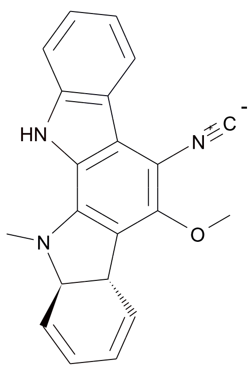

$C_{21}H_{17}N_3O$

Molecular Weight: 327.37918

ALogP: 4.078

Rotatable Bonds: 1

Acceptors: 2

Donors: 1

Model Prediction

Prediction: Irritant

Probability: 0.995

Enrichment: 1.08

Bayesian Score: -0.0162

Mahalanobis Distance: 9.51

Mahalanobis Distance p-value: 0.187

Prediction: Positive if the Bayesian score is above the estimated best cutoff value from minimizing the false positive and false negative rate.
Probability: The esimated probability that the sample is in the positive category. This assumes that the Bayesian score follows a normal distribution and is different from the prediction using a cutoff.
Enrichment: An estimate of enrichment, that is, the increased likelihood (versus random) of this sample being in the category.
Bayesian Score: The standard Laplacian-modified Bayesian score.
Mahalanobis Distance: The Mahalanobis distance (MD) is the distance to the center of the training data. The larger the MD, the less trustworthy the prediction.
Mahalanobis Distance p-value: The p-value gives the fraction of training data with an MD greater than or equal to the one for the given sample, assuming normally distributed data. The smaller the p-value, the less trustworthy the prediciton. For highly non-normal X properties (e.g., fingerprints), the MD p-value is wildly inaccurate.

Structural Similar Compounds

Name	1-Amino-2,4-dibromanthraquinone	Benzenesulfonamide, N-methyl-2,4,5-trichloro-	Aniline, 2,4-bis(o-methylphenoxy)-
Structure	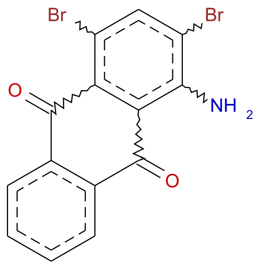	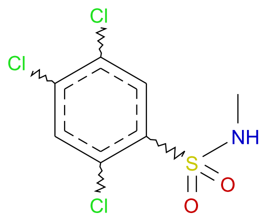	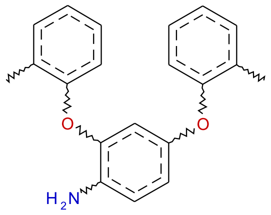
Actual Endpoint	Non-Irritant	Irritant	Irritant
Predicted Endpoint	Non-Irritant	Non-Irritant	Non-Irritant
Distance	0.653	0.664	0.685
Reference	28ZPAK -,88,72	85JCAE "Prehled Prumyslove Toxikologie; Organicke Latky," Marhold, J., Prague , Czechoslovakia, Avicenum, 1986 Volume(issue)/page/year: -,1074,1986	85JCAE "Prehled Prumyslove Toxikologie; Organicke Latky," Marhold, J., Prague , Czechoslovakia, Avicenum, 1986 Volume(issue)/page/year: -,725,1986

Model Applicability

Unknown features are fingerprint features in the query molecule, but not found or appearing too infrequently in the training set.

1. All properties and OPS components are within expected ranges.
2. Unknown FCFP_2 feature: 4: [*]#[C-]
3. Unknown FCFP_2 feature: 1934974835: [*]:[c](:[*])[N+]#[C-]
4. Unknown FCFP_2 feature: -1487147388: [*][N+]#[C-]

Feature Contribution

Top features for positive contribution

Fingerprint	Bit/Smiles	Feature Structure	Score	Irritant in training set

FCFP_12	8	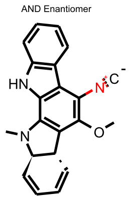 [*][N+]#[*]	0.0843	20 out of 20
FCFP_12	1186303932	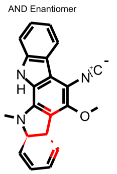 [*]:[c](:[*])C1C[*]=[*]C1	0.0838	18 out of 18
FCFP_12	-828984032	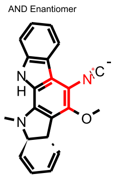 [*][c](:[*]):[c]([N+]#[*]):[c](:[*]):[*]	0.0795	9 out of 9
**Top Features for negative contribution**				
**Fingerprint**	**Bit/Smiles**	**Feature Structure**	**Score**	**Irritant in training set**
FCFP_12	-1320007763	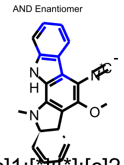 [*]:[c]1:[*]:[*]:[c]2:[*]:[cH]:[cH]:[cH]:[c]:1:2	-0.0893	20 out of 24
FCFP_12	675769755	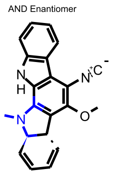 [*]=CN(C)[c](:[*]):[*]	-0.088	15 out of 18

FCFP_12	1618154665	AND Enantiomer<br>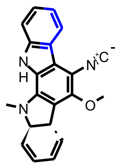<br><br>[*]:[cH]:[cH]:[cH]:[*<br>]	-0.0845	412 out of 490

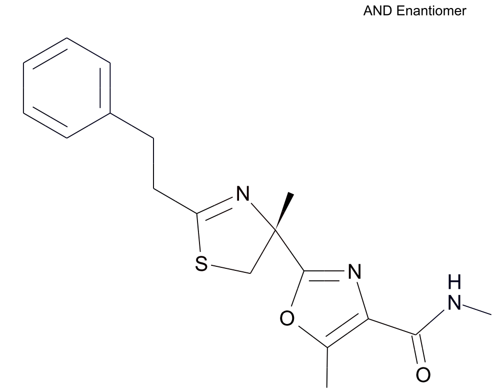

$C_{18}H_{21}N_3O_2S$
Molecular Weight: 343.44324
ALogP: 2.716
Rotatable Bonds: 5
Acceptors: 4
Donors: 1

Model Prediction

Prediction: Non-Irritant
Probability: 0.971
Enrichment: 1.05
Bayesian Score: -0.946
Mahalanobis Distance: 10.6
Mahalanobis Distance p-value: 0.0105

Prediction: Positive if the Bayesian score is above the estimated best cutoff value from minimizing the false positive and false negative rate.
Probability: The esimated probability that the sample is in the positive category. This assumes that the Bayesian score follows a normal distribution and is different from the prediction using a cutoff.
Enrichment: An estimate of enrichment, that is, the increased likelihood (versus random) of this sample being in the category.
Bayesian Score: The standard Laplacian-modified Bayesian score.
Mahalanobis Distance: The Mahalanobis distance (MD) is the distance to the center of the training data. The larger the MD, the less trustworthy the prediction.
Mahalanobis Distance p-value: The p-value gives the fraction of training data with an MD greater than or equal to the one for the given sample, assuming normally distributed data. The smaller the p-value, the less trustworthy the prediciton. For highly non-normal X properties (e.g., fingerprints), the MD p-value is wildly inaccurate.

Structural Similar Compounds

Name	Benzoic acid, 5-(chlorosulfonyl)-2,4-dichloro-	Phosphorothioic acid, O,O-dimethyl O-(3,5,6-trichloro-2-pyridyl) es ter	Benzenesulfonic acid, 3-(diethylamino)-, sodium salt
Structure	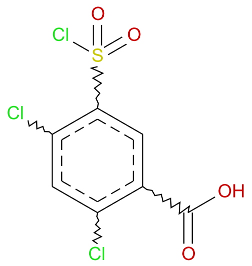	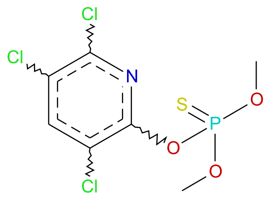	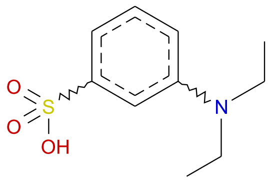
Actual Endpoint	Irritant	Irritant	Irritant
Predicted Endpoint	Non-Irritant	Irritant	Non-Irritant
Distance	0.694	0.732	0.736
Reference	FCTOD7 Food and Chemical Toxicology. (Pergamon Press Inc., Maxwell House, Fa irview Park, Elmsford, NY 10523) V.20- 1982- Volume(issue)/page/year: 20,563 ,1982	TXAPA9 Toxicology and Applied Pharmacology. (Academic Press, Inc., 1 E. Firs t St., Duluth, MN 55802) V.1- 1959- Volume(issue)/page/year: 21,369,1972	85JCAE "Prehled Prumyslove Toxikologie; Organicke Latky," Marhold, J., Prague , Czechoslovakia, Avicenum, 1986 Volume(issue)/page/year: -,1056,1986

Model Applicability

Unknown features are fingerprint features in the query molecule, but not found or appearing too infrequently in the training set.

1. All properties and OPS components are within expected ranges.

Feature Contribution

Top features for positive contribution

Fingerprint	Bit/Smiles	Feature Structure	Score	Irritant in training set

FCFP_12	-1539132615	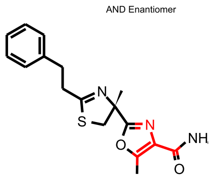 [*]:n:[c](C):[c](:[*]):[*]	0.0795	9 out of 9
FCFP_12	580930443	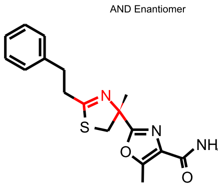 [*]C1=NC[*][*]1	0.0772	7 out of 7
FCFP_12	-1549103449	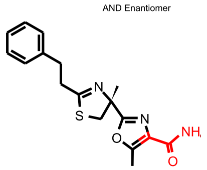 [*]NC(=O)[c](:[*]):[*]	0.0734	5 out of 5
**Top Features for negative contribution**				
**Fingerprint**	**Bit/Smiles**	**Feature Structure**	**Score**	**Irritant in training set**
FCFP_12	136686699	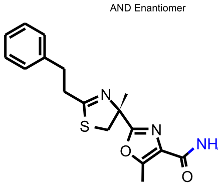 [*]NC	-0.484	3 out of 6
FCFP_12	1388176727	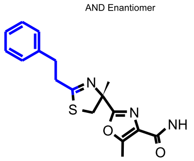 [*]C(=[*])CC[c]1:[cH] :[cH]:[cH]:[cH]:[cH] :1	-0.12	8 out of 10

FCFP_12	1618154665	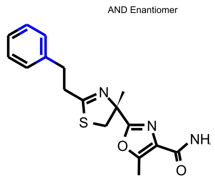<br>[*]:[cH]:[cH]:[cH]:[*<br>]	-0.0845	412 out of 490

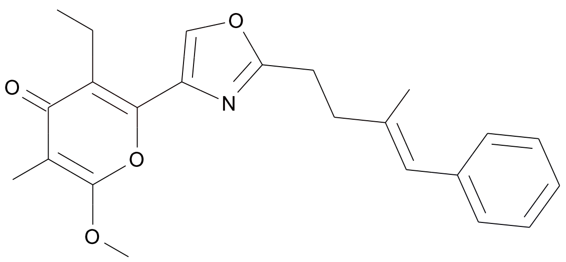

$C_{23}H_{25}NO_4$
Molecular Weight: 379.4489
ALogP: 5.22
Rotatable Bonds: 7
Acceptors: 4
Donors: 0

Model Prediction

Prediction: Irritant
Probability: 0.996
Enrichment: 1.08
Bayesian Score: 0.18
Mahalanobis Distance: 9.14
Mahalanobis Distance p-value: 0.347

Prediction: Positive if the Bayesian score is above the estimated best cutoff value from minimizing the false positive and false negative rate.
Probability: The esimated probability that the sample is in the positive category. This assumes that the Bayesian score follows a normal distribution and is different from the prediction using a cutoff.
Enrichment: An estimate of enrichment, that is, the increased likelihood (versus random) of this sample being in the category.
Bayesian Score: The standard Laplacian-modified Bayesian score.
Mahalanobis Distance: The Mahalanobis distance (MD) is the distance to the center of the training data. The larger the MD, the less trustworthy the prediction.
Mahalanobis Distance p-value: The p-value gives the fraction of training data with an MD greater than or equal to the one for the given sample, assuming normally distributed data. The smaller the p-value, the less trustworthy the prediciton. For highly non-normal X properties (e.g., fingerprints), the MD p-value is wildly inaccurate.

Structural Similar Compounds

Name	Phosphorothioic acid, O-ethyl S-propyl O-(2,4,6-trichlorophenyl) es ter	Phosphorous acid, triphenyl ester	Propanoic acid, 2-(4-((5-(trifluoromethyl)-2-pyridinyl)oxy)phenoxy) -, butyl ester
Structure	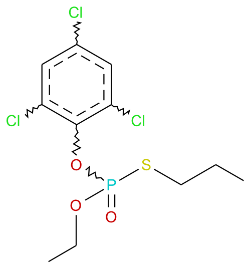	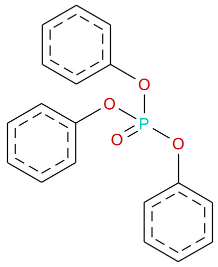	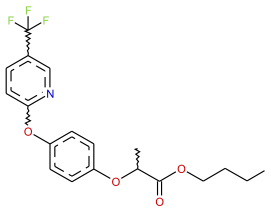
Actual Endpoint	Irritant	Irritant	Irritant
Predicted Endpoint	Irritant	Irritant	Irritant
Distance	0.570	0.601	0.628
Reference	NTIS** National Technical Information Service. (Springfield, VA 22161) Forme rly U.S. Clearinghouse for Scientific & Technical Information. Volume(issue)/pag e/year: OTS0535844	AMIHBC AMA Archives of Industrial Hygiene and Occupational Medicine. (Chicago , IL) V.2-10, 1950-54. For publisher information, see AEHLAU. Volume(issue)/pag e/year: 5,311,1952	NNGADV Nippon Noyaku Gakkaishi. Journal of the Pesticide Science Society of Japan. (Nippon Noyaku Gakkai, 1-43-11, Komagome, Toshima-ku, Tokyo 170, Japan) V.1-1976- Volume(issue)/page/year: 15,305,1990

Model Applicability

Unknown features are fingerprint features in the query molecule, but not found or appearing too infrequently in the training set.

1. All properties and OPS components are within expected ranges.

Feature Contribution

Top features for positive contribution				
Fingerprint	Bit/Smiles	Feature Structure	Score	Irritant in training set

FCFP_12	-124655670	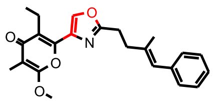 [*][c](:[*]):[cH]:n:[*]	0.0821	13 out of 13
FCFP_12	436886043	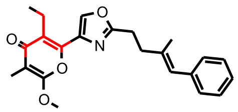 [*]C=C(C)C	0.0804	129 out of 130
FCFP_12	-1539132615	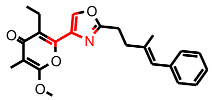 [*]:n:[c](C):[c](:[*]):[*]	0.0795	9 out of 9

Top Features for negative contribution

Fingerprint	Bit/Smiles	Feature Structure	Score	Irritant in training set
FCFP_12	1618154665	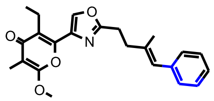 [*]:[cH]:[cH]:[cH]:[*]	-0.0845	412 out of 490
FCFP_12	16	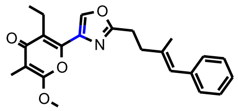 [*]:[cH]:[*]	-0.0843	423 out of 503

FCFP_12	991735244	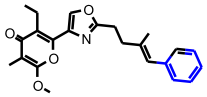 [*]:[c]1:[*]:[cH]:[cH]:[cH]:[cH]:1	-0.0805	249 out of 295

Remdesivir

TOPKAT_Skin_Irritancy_None_vs_Irritant

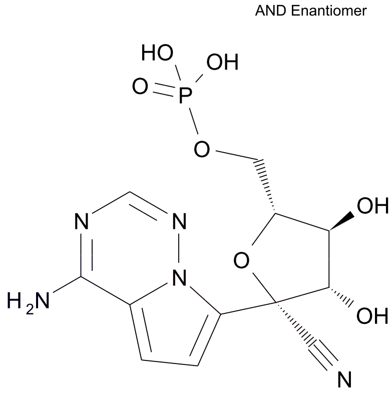

$C_{12}H_{14}N_5O_7P$

Molecular Weight: 371.24262

ALogP: -1.502

Rotatable Bonds: 4

Acceptors: 11

Donors: 5

Structural Similar Compounds

Name	1,3,6-Naphthalenetrisulfonic acid, 7-amino-	2,2'-Benzidine disulfonic acid	2,7-Anthracenedisulfonic acid, 9,10-dihydro-4,5-diamino-9,10-dioxo- 1-hydroxy-, disodium salt
Structure	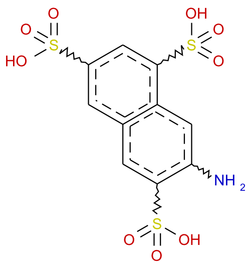	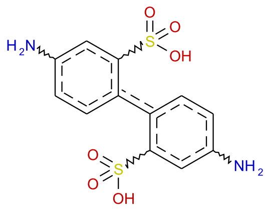	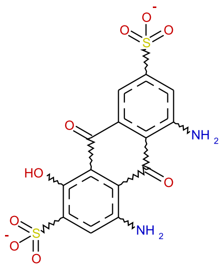
Actual Endpoint	Irritant	Non-Irritant	Irritant
Predicted Endpoint	Non-Irritant	Non-Irritant	Non-Irritant
Distance	0.755	0.896	1.025
Reference	85JCAE "Prehled Prumyslove Toxikologie; Organicke Latky," Marhold, J., Prague , Czechoslovakia, Avicenum, 1986 Volume(issue)/page/year: -,1058,1986	28ZPAK -,191,72	28ZPAK "Sbornik Vysledku Toxixologickeho Vysetreni Latek A Pripravku," Marhol d, J.V., Institut Pro Vychovu Vedoucicn Pracovniku Chemickeho Prumyclu Praha, Cz echoslovakia, 1972 Volume(issue)/page/year: -,239,1

Model Prediction

Prediction: Irritant

Probability: 0.976

Enrichment: 1.06

Bayesian Score: -0.492

Mahalanobis Distance: 13.2

Mahalanobis Distance p-value: 3.18e-008

Prediction: Positive if the Bayesian score is above the estimated best cutoff value from minimizing the false positive and false negative rate.
Probability: The esimated probability that the sample is in the positive category. This assumes that the Bayesian score follows a normal distribution and is different from the prediction using a cutoff.
Enrichment: An estimate of enrichment, that is, the increased likelihood (versus random) of this sample being in the category.
Bayesian Score: The standard Laplacian-modified Bayesian score.
Mahalanobis Distance: The Mahalanobis distance (MD) is the distance to the center of the training data. The larger the MD, the less trustworthy the prediction.
Mahalanobis Distance p-value: The p-value gives the fraction of training data with an MD greater than or equal to the one for the given sample, assuming normally distributed data. The smaller the p-value, the less trustworthy the prediciton. For highly non-normal X properties (e.g., fingerprints), the MD p-value is wildly inaccurate.

Model Applicability

Unknown features are fingerprint features in the query molecule, but not found or appearing too infrequently in the training set.

1. All properties and OPS components are within expected ranges.
2. Unknown FCFP_2 feature: 472180098: [*]OP(=O)(O)O
3. Unknown FCFP_2 feature: -332197802: [*][c]1:[*]:[*]:[c](:[*]):n:1:n:[*]

Feature Contribution

Top features for positive contribution

Fingerprint	Bit/Smiles	Feature Structure	Score	Irritant in training set

FCFP_12	654335567	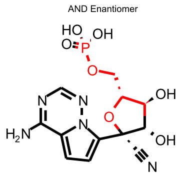 [*][C@H]1[*][*]O[C@@H]1COP(=[*])([*])[*]	0.0856	29 out of 29
FCFP_12	-1539132615	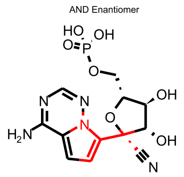 [*]:n:[c](C):[c](:[*]):[*]	0.0795	9 out of 9
FCFP_12	-1280036918	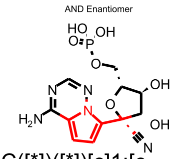 [*]C([*])([*])[c]1:[cH]:[cH]:[c](:[*]):n:1:[*]	0.0772	7 out of 7

Top Features for negative contribution

Fingerprint	Bit/Smiles	Feature Structure	Score	Irritant in training set
FCFP_12	1069584379	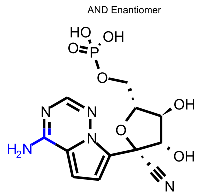 [*]:[c](:[*])N	-0.439	38 out of 65
FCFP_12	1618154665	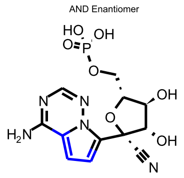 [*]:[cH]:[cH]:[cH]:[*]	-0.0845	412 out of 490

FCFP_12	16	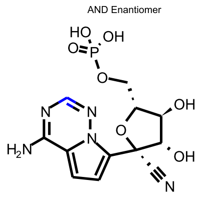 [*]:[cH]:[*]	-0.0843	423 out of 503

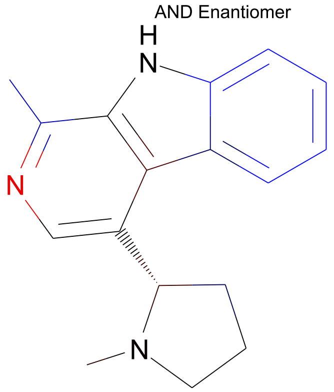

$C_{17}H_{19}N_3$

Molecular Weight: 265.35286

ALogP: 3.018

Rotatable Bonds: 1

Acceptors: 2

Donors: 1

Model Prediction

Prediction: 15.5

Unit: mg/kg_body_weight/day

Mahalanobis Distance: 11.1

Mahalanobis Distance p-value: 0.000881

Mahalanobis Distance: The Mahalanobis distance (MD) is a generalization of the Euclidean distance that accounts for correlations among the X properties. It is calculated as the distance to the center of the training data. The larger the MD, the less trustworthy the prediction.
Mahalanobis Distance p-value: The p-value gives the fraction of training data with an MD greater than or equal to the one for the given sample, assuming normally distributed data. The smaller the p-value, the less trustworthy the prediciton. For highly non-normal X properties (e.g., fingerprints), the MD p-value is wildly inaccurate.

Structural Similar Compounds

Name	429	1140	Carbazole
Structure	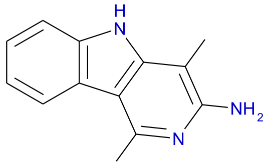	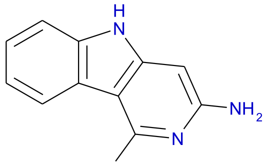	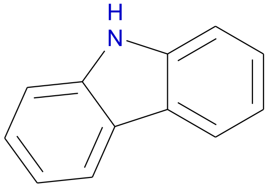
Actual Endpoint (-log C)	3.71523	4.19462	3.00841
Predicted Endpoint (-log C)	4.22904	3.93599	3.7131
Distance	0.573	0.606	0.607
Reference	CPDB	CPDB	CPDB

Model Applicability

Unknown features are fingerprint features in the query molecule, but not found or appearing too infrequently in the training set.

1. All properties and OPS components are within expected ranges.
2. Unknown ECFP_2 feature: 48510090: [*][C@@H]1[*][*]CN1C

Feature Contribution

Top features for positive contribution			
**Fingerprint**	**Bit/Smiles**	**Feature Structure**	**Score**
ECFP_6	655739385	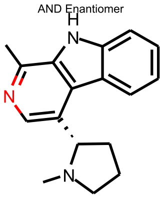 [*]:n:[*]	0.229

ECFP_6	1333660716	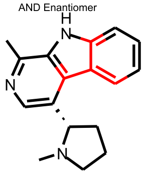 [*]:[cH]:[c]1:[c](:[*]):[*]:[*]:[c]:1:[*]	0.0746
ECFP_6	-167460056	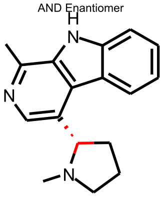 [*]C([*])[*]	0.0596
**Top Features for negative contribution**			
**Fingerprint**	**Bit/Smiles**	**Feature Structure**	**Score**
ECFP_6	1996767644	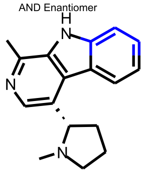 [*]:[cH]:[cH]:[c](:[*]):[*]	-0.251
ECFP_6	642810091	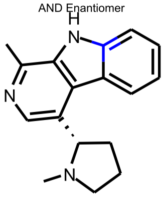 [*]:[c](:[*]):[*]	-0.247
ECFP_6	-182236392	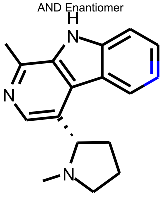 [*]:[cH]:[*]	-0.232

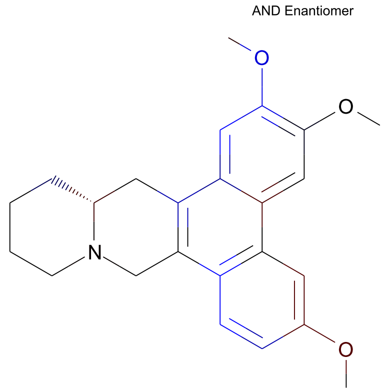

$C_{24}H_{27}NO_3$
Molecular Weight: 377.47608
ALogP: 4.691
Rotatable Bonds: 3
Acceptors: 4
Donors: 0

Model Prediction

Prediction: 7.85
Unit: mg/kg_body_weight/day
Mahalanobis Distance: 10.9
Mahalanobis Distance p-value: 0.0022

Mahalanobis Distance: The Mahalanobis distance (MD) is a generalization of the Euclidean distance that accounts for correlations among the X properties. It is calculated as the distance to the center of the training data. The larger the MD, the less trustworthy the prediction.
Mahalanobis Distance p-value: The p-value gives the fraction of training data with an MD greater than or equal to the one for the given sample, assuming normally distributed data. The smaller the p-value, the less trustworthy the prediciton. For highly non-normal X properties (e.g., fingerprints), the MD p-value is wildly inaccurate.

Structural Similar Compounds

Name	C.I. vat yellow 4	348	Phenylbutazone
Structure	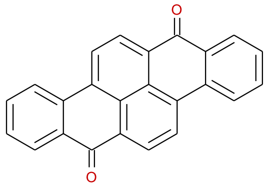	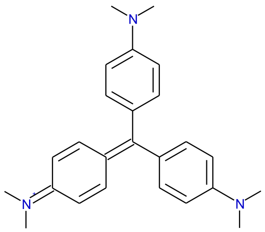	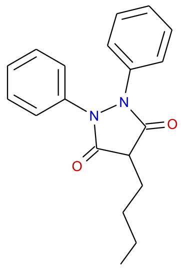
Actual Endpoint (-log C)	1.48417	3.61451	2.9413
Predicted Endpoint (-log C)	4.45029	5.05011	3.29616
Distance	0.777	0.796	0.810
Reference	CPDB	CPDB	CPDB

Model Applicability

Unknown features are fingerprint features in the query molecule, but not found or appearing too infrequently in the training set.

1. OPS PC23 out of range. Value: 3.4957. Training min, max, SD, explained variance: -2.6901, 3.3252, 1.05, 0.0138.
2. Unknown ECFP_2 feature: -1869628272: [*]CC(C[*])N([*])[*]

Feature Contribution

Top features for positive contribution

Fingerprint	Bit/Smiles	Feature Structure	Score
ECFP_6	-176455838	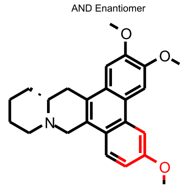 [*]O[c](:[cH]:[*]):[cH]:[*]	0.0818

ECFP_6	1333660716	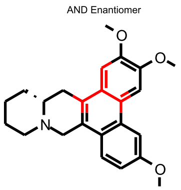<br>[*]:[cH]:[c]1:[c](:[*]):[*]:[*]:[c]:1:[*]	0.0746
ECFP_6	-167460056	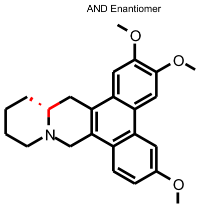<br>[*]C([*])[*]	0.0596

Top Features for negative contribution

Fingerprint	Bit/Smiles	Feature Structure	Score
ECFP_6	1996767644	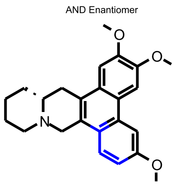<br>[*]:[cH]:[cH]:[c](:[*]):[*]	-0.251
ECFP_6	642810091	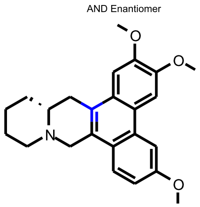<br>[*]:[c](:[*]):[*]	-0.247
ECFP_6	-182236392	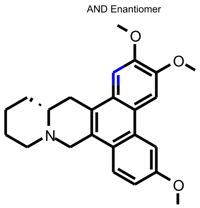<br>[*]:[cH]:[*]	-0.232

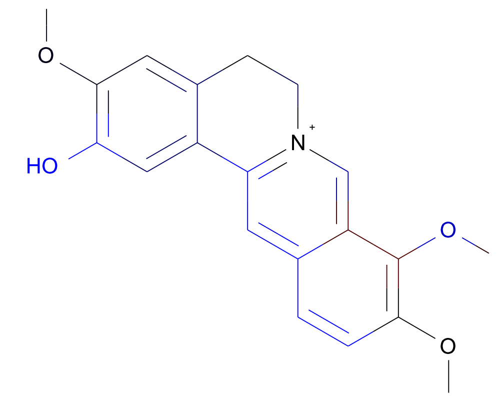

$C_{20}H_{20}NO_4$
Molecular Weight: 338.3771
ALogP: 3.936
Rotatable Bonds: 3
Acceptors: 4
Donors: 1

Model Prediction

Prediction: 86.7
Unit: mg/kg_body_weight/day
Mahalanobis Distance: 13
Mahalanobis Distance p-value: 9.46e-008

Mahalanobis Distance: The Mahalanobis distance (MD) is a generalization of the Euclidean distance that accounts for correlations among the X properties. It is calculated as the distance to the center of the training data. The larger the MD, the less trustworthy the prediction.
Mahalanobis Distance p-value: The p-value gives the fraction of training data with an MD greater than or equal to the one for the given sample, assuming normally distributed data. The smaller the p-value, the less trustworthy the prediciton. For highly non-normal X properties (e.g., fingerprints), the MD p-value is wildly inaccurate.

Structural Similar Compounds

Name	Phenolphthalein	646	Sterigmatocystin s
Structure	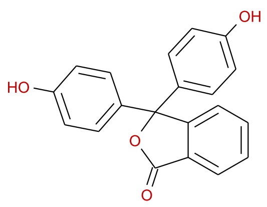	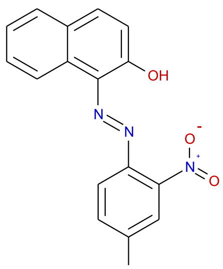	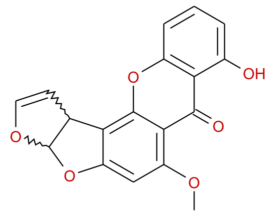
Actual Endpoint (-log C)	2.43468	0.937339	5.55284
Predicted Endpoint (-log C)	3.66084	3.26294	3.6442
Distance	0.630	0.692	0.733
Reference	CPDB	CPDB	CPDB

Model Applicability

Unknown features are fingerprint features in the query molecule, but not found or appearing too infrequently in the training set.

1. OPS PC13 out of range. Value: -3.3264. Training min, max, SD, explained variance: -3.068, 3.6909, 1.329, 0.0220.
2. Unknown ECFP_2 feature: 1508268466: [*]C[n+](:[c]([*]):[*]):c:[*]
3. Unknown ECFP_2 feature: -1333923932: [*]CC[n+](:[*]):[*]
4. Unknown ECFP_2 feature: 2078070048: [*][n+](:[*]):[c]([c](:[*]):[*]):c:[*]
5. Unknown ECFP_2 feature: 688916016: [*][n+](:[*]):c:[c](:[*]):[*]

Feature Contribution

Top features for positive contribution			
Fingerprint	Bit/Smiles	Feature Structure	Score
ECFP_6	1333660716	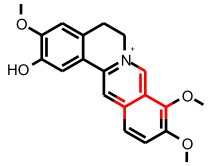 [*]:[cH]:[c]1:[c](:[*]):[*]:[*]:[c]:1:[*]	0.0746

ECFP_6	734603939	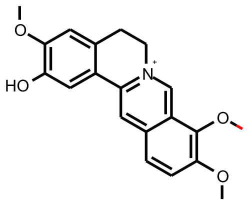<br>[*]C	0.0424
ECFP_6	1307307440	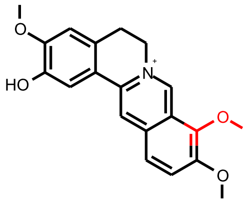<br>[*]:[c](:[*])OC	0.0156

Top Features for negative contribution

Fingerprint	Bit/Smiles	Feature Structure	Score
ECFP_6	2019062761	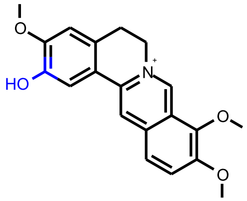<br>[*]:[c](:[*])O	-0.258
ECFP_6	1996767644	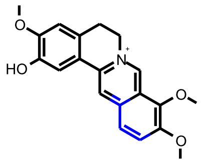<br>[*]:[cH]:[cH]:[c](:[*<br>]):[*]	-0.251
ECFP_6	642810091	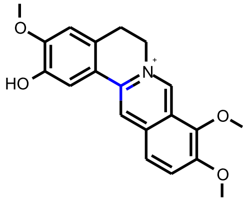<br>[*]:[c](:[*]):[*]	-0.247

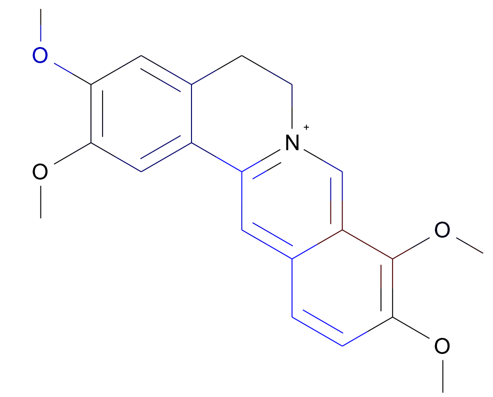

$C_{21}H_{22}NO_4$
Molecular Weight: 352.40368
ALogP: 4.161
Rotatable Bonds: 4
Acceptors: 4
Donors: 0

Model Prediction

Prediction: 41.4
Unit: mg/kg_body_weight/day
Mahalanobis Distance: 12.7
Mahalanobis Distance p-value: 5.2e-007

Mahalanobis Distance: The Mahalanobis distance (MD) is a generalization of the Euclidean distance that accounts for correlations among the X properties. It is calculated as the distance to the center of the training data. The larger the MD, the less trustworthy the prediction.
Mahalanobis Distance p-value: The p-value gives the fraction of training data with an MD greater than or equal to the one for the given sample, assuming normally distributed data. The smaller the p-value, the less trustworthy the prediciton. For highly non-normal X properties (e.g., fingerprints), the MD p-value is wildly inaccurate.

Structural Similar Compounds

Name	Phenylbutazone	348	Methyl clofenapate
Structure	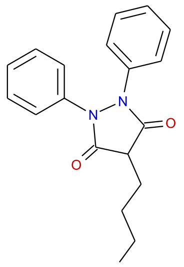	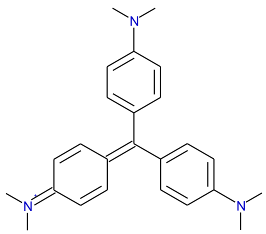	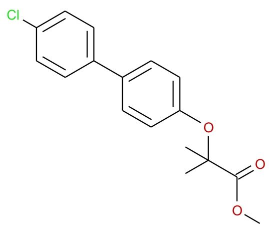
Actual Endpoint (-log C)	2.9413	3.61451	5.0843
Predicted Endpoint (-log C)	3.29616	5.05011	3.36581
Distance	0.724	0.747	0.761
Reference	CPDB	CPDB	CPDB

Model Applicability

Unknown features are fingerprint features in the query molecule, but not found or appearing too infrequently in the training set.

1. All properties and OPS components are within expected ranges.
2. Unknown ECFP_2 feature: 1508268466: [*]C[n+](:[c]([*]):[*]):c:[*]
3. Unknown ECFP_2 feature: -1333923932: [*]CC[n+](:[*]):[*]
4. Unknown ECFP_2 feature: 2078070048: [*][n+](:[*]):[c]([c](:[*]):[*]):c:[*]
5. Unknown ECFP_2 feature: 688916016: [*][n+](:[*]):c:[c](:[*]):[*]

Feature Contribution

Top features for positive contribution

Fingerprint	Bit/Smiles	Feature Structure	Score
ECFP_6	1333660716	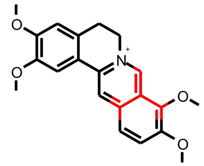 [*]:[cH]:[c]1:[c](:[*]):[*]:[*]:[c]:1:[*]	0.0746

ECFP_6	734603939	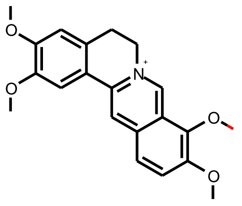 [*]C	0.0424
ECFP_6	1307307440	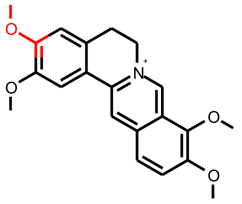 [*]:[c](:[*])OC	0.0156

Top Features for negative contribution

Fingerprint	Bit/Smiles	Feature Structure	Score
ECFP_6	1996767644	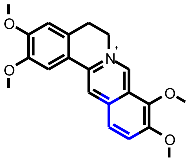 [*]:[cH]:[cH]:[c](:[*]):[*]	-0.251
ECFP_6	642810091	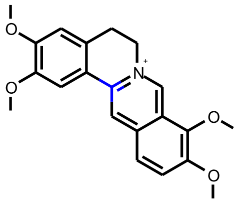 [*]:[c](:[*]):[*]	-0.247
ECFP_6	-182236392	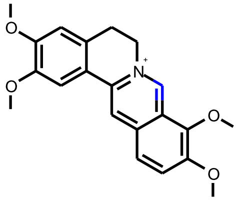 [*]:[cH]:[*]	-0.232

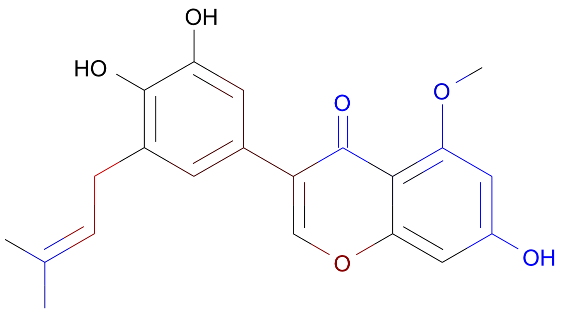

$C_{21}H_{20}O_6$

Molecular Weight: 368.3799

ALogP: 3.98

Rotatable Bonds: 4

Acceptors: 6

Donors: 3

Structural Similar Compounds

Name	542	Ochratoxin A	[4-Chloro-6-(2,3-xylidino)-2-pyri-midinylthio]acetic acid s
Structure	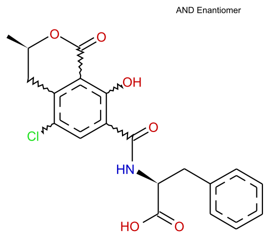	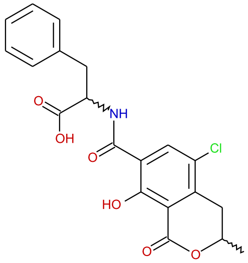	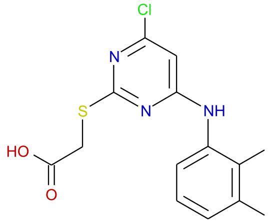
Actual Endpoint (-log C)	4.79932	4.79932	4.47685
Predicted Endpoint (-log C)	3.6353	3.6353	3.8529
Distance	0.562	0.562	0.653
Reference	CPDB	CPDB	CPDB

Model Prediction

Prediction: 40.8

Unit: mg/kg_body_weight/day

Mahalanobis Distance: 13.7

Mahalanobis Distance p-value: 2.07e-009

Mahalanobis Distance: The Mahalanobis distance (MD) is a generalization of the Euclidean distance that accounts for correlations among the X properties. It is calculated as the distance to the center of the training data. The larger the MD, the less trustworthy the prediction.
Mahalanobis Distance p-value: The p-value gives the fraction of training data with an MD greater than or equal to the one for the given sample, assuming normally distributed data. The smaller the p-value, the less trustworthy the prediciton. For highly non-normal X properties (e.g., fingerprints), the MD p-value is wildly inaccurate.

Model Applicability

Unknown features are fingerprint features in the query molecule, but not found or appearing too infrequently in the training set.

1. All properties and OPS components are within expected ranges.
2. Unknown ECFP_2 feature: 1717082529: [*]\C=C(/C(=[*])[*])\[c](:[*]):[*]

Feature Contribution

Top features for positive contribution

Fingerprint	Bit/Smiles	Feature Structure	Score
ECFP_6	1559650422	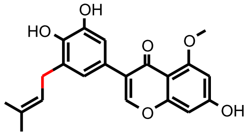 [*]C[*]	0.203

ECFP_6	-1925046727	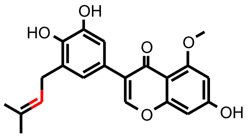 [*]C=[*]	0.145
ECFP_6	683445015	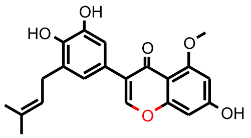 [*]O[*]	0.136
**Top Features for negative contribution**			
**Fingerprint**	**Bit/Smiles**	**Feature Structure**	**Score**
ECFP_6	2106656448	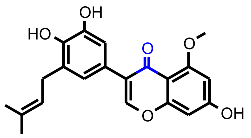 [*]C(=O)[*]	-0.275
ECFP_6	2019062761	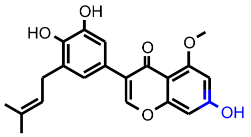 [*]:[c](:[*])O	-0.258
ECFP_6	642810091	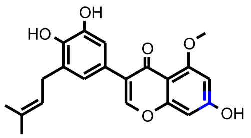 [*]:[c](:[*]):[*]	-0.247

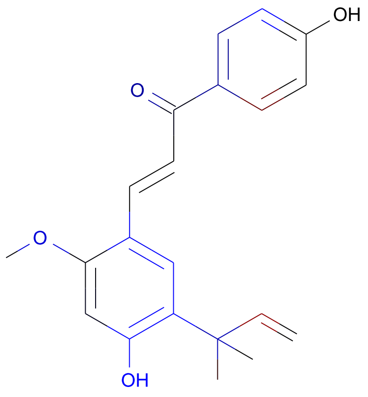

$C_{21}H_{22}O_4$
Molecular Weight: 338.39698
ALogP: 4.667
Rotatable Bonds: 6
Acceptors: 4
Donors: 2

Model Prediction

Prediction: 345
Unit: mg/kg_body_weight/day
Mahalanobis Distance: 11.6
Mahalanobis Distance p-value: 0.000118

Mahalanobis Distance: The Mahalanobis distance (MD) is a generalization of the Euclidean distance that accounts for correlations among the X properties. It is calculated as the distance to the center of the training data. The larger the MD, the less trustworthy the prediction.
Mahalanobis Distance p-value: The p-value gives the fraction of training data with an MD greater than or equal to the one for the given sample, assuming normally distributed data. The smaller the p-value, the less trustworthy the prediciton. For highly non-normal X properties (e.g., fingerprints), the MD p-value is wildly inaccurate.

Structural Similar Compounds

Name	455	Diethylstilbestrol	5
Structure	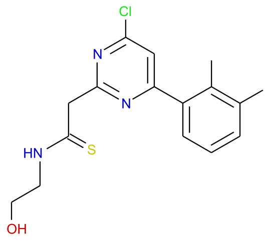	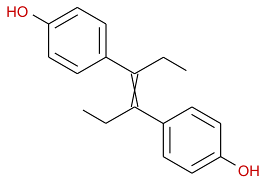	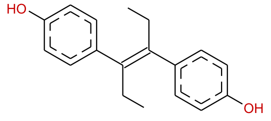
Actual Endpoint (-log C)	3.87681	6.83653	6.85816
Predicted Endpoint (-log C)	3.77582	3.82521	3.82521
Distance	0.596	0.615	0.615
Reference	CPDB	CPDB	CPDB

Model Applicability

Unknown features are fingerprint features in the query molecule, but not found or appearing too infrequently in the training set.

1. OPS PC8 out of range. Value: -4.5127. Training min, max, SD, explained variance: -4.4834, 4.8926, 1.605, 0.0321.
2. Unknown ECFP_2 feature: -144557007: [*]=CC(C)(C)[c](:[*]):[*]
3. Unknown ECFP_2 feature: -1193716553: [*]C([*])([*])C=C
4. Unknown ECFP_2 feature: 1430764055: [*]=CC(=O)[c](:[*]):[*]

Feature Contribution

Top features for positive contribution

Fingerprint	Bit/Smiles	Feature Structure	Score
ECFP_6	-1925046727	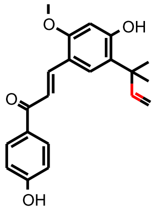 [*]C=[*]	0.145

ECFP_6	-175146122	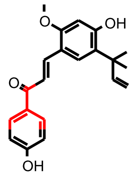 [*]C(=[*])[c](:[cH]:[*]):[cH]:[*]	0.107
ECFP_6	734603939	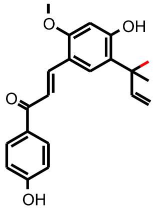 [*]C	0.0424
**Top Features for negative contribution**			
**Fingerprint**	**Bit/Smiles**	**Feature Structure**	**Score**
ECFP_6	2019062761	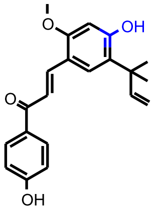 [*]:[c](:[*])O	-0.258
ECFP_6	1996767644	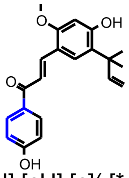 [*]:[cH]:[cH]:[c](:[*]):[*]	-0.251
ECFP_6	642810091	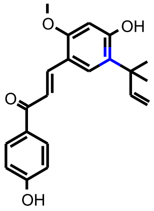 [*]:[c](:[*]):[*]	-0.247

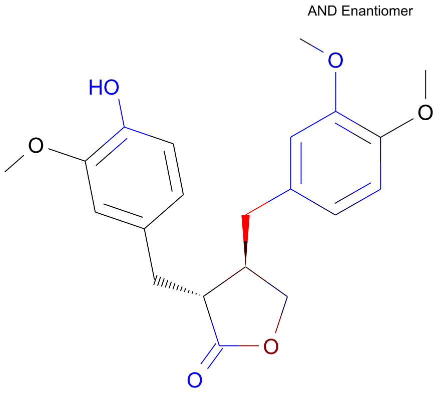

$C_{21}H_{24}O_6$
Molecular Weight: 372.41166
ALogP: 3.743
Rotatable Bonds: 7
Acceptors: 6
Donors: 1

Model Prediction

Prediction: 154
Unit: mg/kg_body_weight/day
Mahalanobis Distance: 10.5
Mahalanobis Distance p-value: 0.00961

Mahalanobis Distance: The Mahalanobis distance (MD) is a generalization of the Euclidean distance that accounts for correlations among the X properties. It is calculated as the distance to the center of the training data. The larger the MD, the less trustworthy the prediction.
Mahalanobis Distance p-value: The p-value gives the fraction of training data with an MD greater than or equal to the one for the given sample, assuming normally distributed data. The smaller the p-value, the less trustworthy the prediciton. For highly non-normal X properties (e.g., fingerprints), the MD p-value is wildly inaccurate.

Structural Similar Compounds

Name	Acifluorfen	Compound LY171883	832
Structure	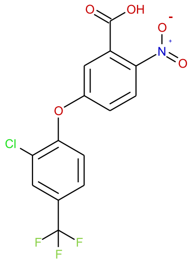	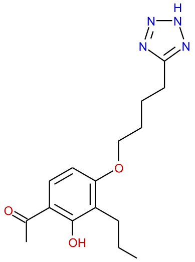	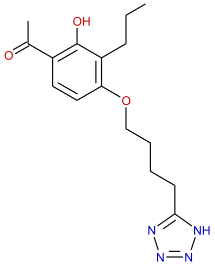
Actual Endpoint (-log C)	3.40908	3.45372	3.45372
Predicted Endpoint (-log C)	3.10974	2.84749	2.80429
Distance	0.674	0.696	0.704
Reference	CPDB	CPDB	CPDB

Model Applicability

Unknown features are fingerprint features in the query molecule, but not found or appearing too infrequently in the training set.

1. All properties and OPS components are within expected ranges.
2. Unknown ECFP_2 feature: -1794289895: [*]C[C@H]1C[*][*][C@@H]1[*]
3. Unknown ECFP_2 feature: -409631777: [*]C[C@@H]1[C@@H]([*])[*][*]C1=[*]
4. Unknown ECFP_2 feature: 771121623: [*]C([*])C[c](:[*]):[*]
5. Unknown ECFP_2 feature: -1886208901: [*][C@@H]1[*][*]OC1=O

Feature Contribution

Top features for positive contribution

Fingerprint	Bit/Smiles	Feature Structure	Score
ECFP_6	1559650422	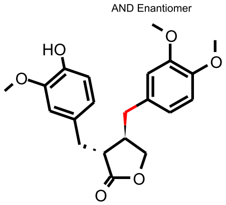 [*]C[*]	0.203

ECFP_6	-2024255407	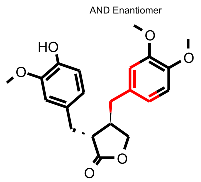 [*]C[c](:[cH]:[*]):[cH]:[*]	0.172
ECFP_6	683445015	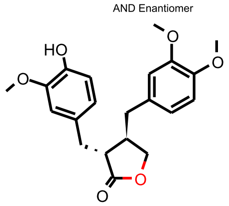 [*]O[*]	0.136
**Top Features for negative contribution**			
**Fingerprint**	**Bit/Smiles**	**Feature Structure**	**Score**
ECFP_6	2106656448	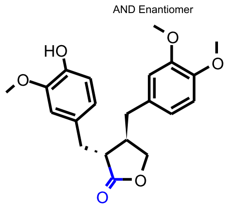 [*]C(=O)[*]	-0.275
ECFP_6	2019062761	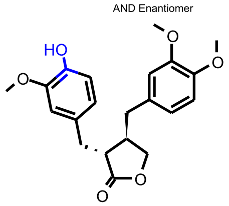 [*]:[c](:[*])O	-0.258
ECFP_6	1996767644	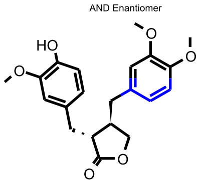 [*]:[cH]:[cH]:[c](:[*]):[*]	-0.251

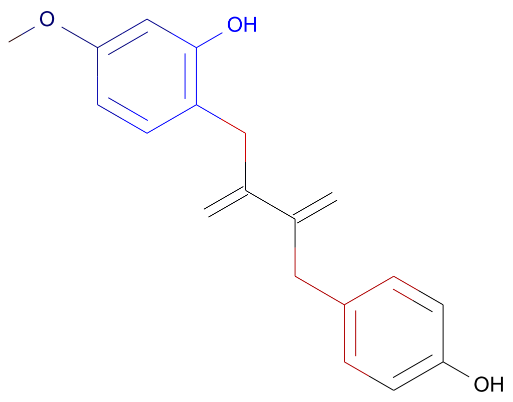

$C_{19}H_{20}O_3$
Molecular Weight: 296.3603
ALogP: 4.784
Rotatable Bonds: 6
Acceptors: 3
Donors: 2

Model Prediction

Prediction: 94.4
Unit: mg/kg_body_weight/day
Mahalanobis Distance: 14.8
Mahalanobis Distance p-value: 2.04e-012

Mahalanobis Distance: The Mahalanobis distance (MD) is a generalization of the Euclidean distance that accounts for correlations among the X properties. It is calculated as the distance to the center of the training data. The larger the MD, the less trustworthy the prediction.
Mahalanobis Distance p-value: The p-value gives the fraction of training data with an MD greater than or equal to the one for the given sample, assuming normally distributed data. The smaller the p-value, the less trustworthy the prediciton. For highly non-normal X properties (e.g., fingerprints), the MD p-value is wildly inaccurate.

Structural Similar Compounds

Name	Diethylstilbestrol	5	Clobuzarit s
Structure	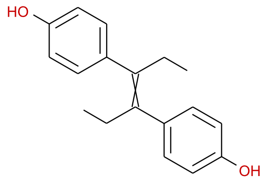	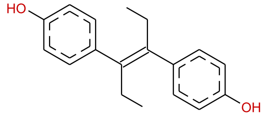	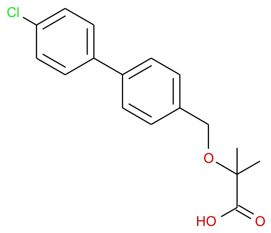
Actual Endpoint (-log C)	6.83653	6.85816	3.29645
Predicted Endpoint (-log C)	3.82521	3.82521	3.52771
Distance	0.527	0.527	0.578
Reference	CPDB	CPDB	CPDB

Model Applicability

Unknown features are fingerprint features in the query molecule, but not found or appearing too infrequently in the training set.

1. All properties and OPS components are within expected ranges.
2. Unknown ECFP_2 feature: -1505409543: [*]CC(=C)C(=[*])[*]

Feature Contribution

Top features for positive contribution

Fingerprint	Bit/Smiles	Feature Structure	Score
ECFP_6	1559650422	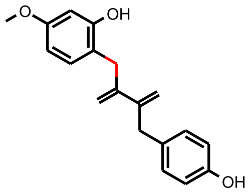 [*]C[*]	0.203

ECFP_6	-2024255407	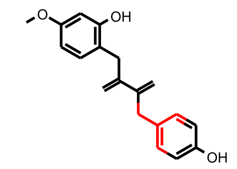 [*]C[c](:[cH]:[*]):[cH]:[*]	0.172
ECFP_6	-176455838	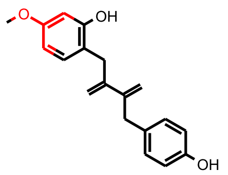 [*]O[c](:[cH]:[*]):[cH]:[*]	0.0818
**Top Features for negative contribution**			
**Fingerprint**	**Bit/Smiles**	**Feature Structure**	**Score**
ECFP_6	2019062761	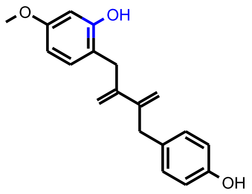 [*]:[c](:[*])O	-0.258
ECFP_6	1996767644	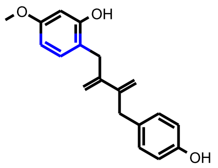 [*]:[cH]:[cH]:[c](:[*]):[*]	-0.251
ECFP_6	642810091	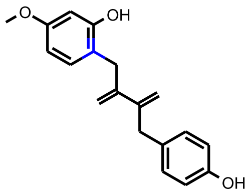 [*]:[c](:[*]):[*]	-0.247

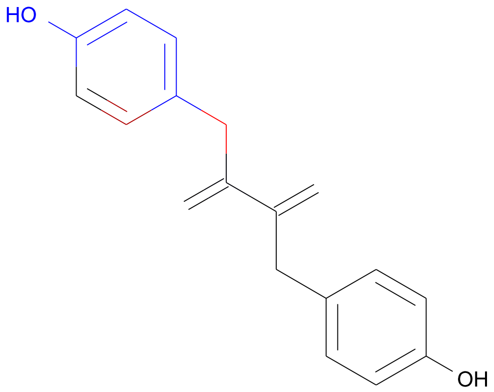

$C_{18}H_{18}O_2$
Molecular Weight: 266.33432
ALogP: 4.8
Rotatable Bonds: 5
Acceptors: 2
Donors: 2

Model Prediction

Prediction: 46
Unit: mg/kg_body_weight/day
Mahalanobis Distance: 12.5
Mahalanobis Distance p-value: 1.72e-006

Mahalanobis Distance: The Mahalanobis distance (MD) is a generalization of the Euclidean distance that accounts for correlations among the X properties. It is calculated as the distance to the center of the training data. The larger the MD, the less trustworthy the prediction.
Mahalanobis Distance p-value: The p-value gives the fraction of training data with an MD greater than or equal to the one for the given sample, assuming normally distributed data. The smaller the p-value, the less trustworthy the prediciton. For highly non-normal X properties (e.g., fingerprints), the MD p-value is wildly inaccurate.

Structural Similar Compounds

Name	5	Diethylstilbestrol	Clobuzarit s
Structure	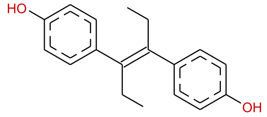	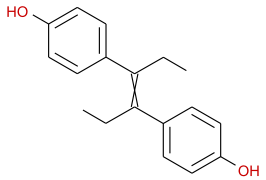	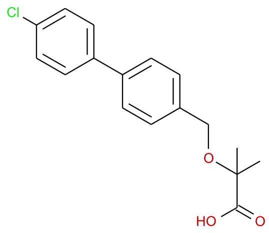
Actual Endpoint (-log C)	6.85816	6.83653	3.29645
Predicted Endpoint (-log C)	3.82521	3.82521	3.52771
Distance	0.408	0.408	0.587
Reference	CPDB	CPDB	CPDB

Model Applicability

Unknown features are fingerprint features in the query molecule, but not found or appearing too infrequently in the training set.

1. All properties and OPS components are within expected ranges.
2. Unknown ECFP_2 feature: -1505409543: [*]CC(=C)C(=[*])[*]

Feature Contribution

Top features for positive contribution			
**Fingerprint**	**Bit/Smiles**	**Feature Structure**	**Score**
ECFP_6	1559650422	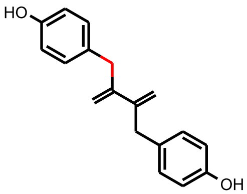 [*]C[*]	0.203

ECFP_6	-2024255407	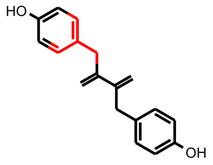 [*]C[c](:[cH]:[*]):[cH]:[*]	0.172
ECFP_6	1544874086	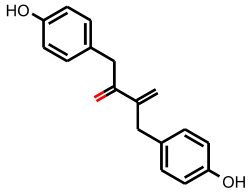 [*]=C	0.00135
**Top Features for negative contribution**			
**Fingerprint**	**Bit/Smiles**	**Feature Structure**	**Score**
ECFP_6	2019062761	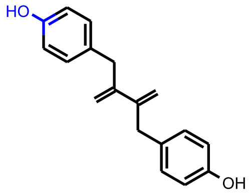 [*]:[c](:[*])O	-0.258
ECFP_6	1996767644	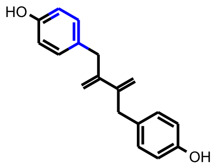 [*]:[cH]:[cH]:[c](:[*]):[*]	-0.251
ECFP_6	642810091	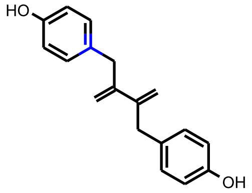 [*]:[c](:[*]):[*]	-0.247

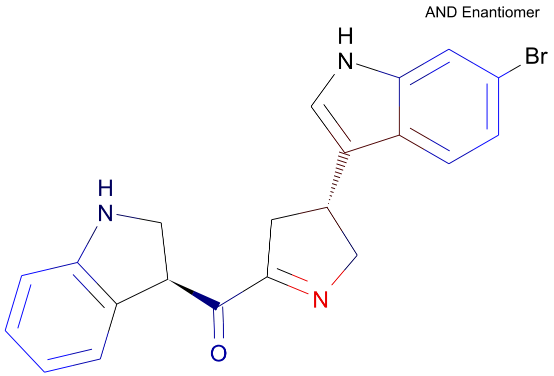

$C_{21}H_{18}BrN_3O$
Molecular Weight: 408.29112
ALogP: 3.919
Rotatable Bonds: 3
Acceptors: 3
Donors: 2

Model Prediction

Prediction: 11.2
Unit: mg/kg_body_weight/day
Mahalanobis Distance: 11.5
Mahalanobis Distance p-value: 0.000188

Mahalanobis Distance: The Mahalanobis distance (MD) is a generalization of the Euclidean distance that accounts for correlations among the X properties. It is calculated as the distance to the center of the training data. The larger the MD, the less trustworthy the prediction.
Mahalanobis Distance p-value: The p-value gives the fraction of training data with an MD greater than or equal to the one for the given sample, assuming normally distributed data. The smaller the p-value, the less trustworthy the prediciton. For highly non-normal X properties (e.g., fingerprints), the MD p-value is wildly inaccurate.

Structural Similar Compounds

Name	Phenolphthalein	Oxazepam	693
Structure	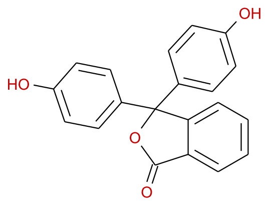	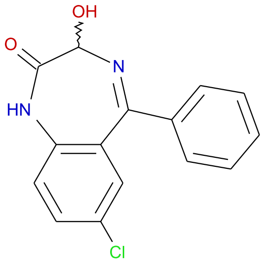	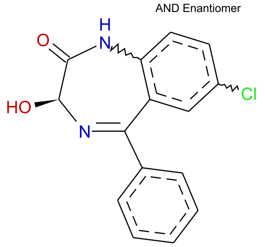
Actual Endpoint (-log C)	2.43468	3.90356	3.90356
Predicted Endpoint (-log C)	3.66084	3.39677	3.39677
Distance	0.664	0.788	0.788
Reference	CPDB	CPDB	CPDB

Model Applicability

Unknown features are fingerprint features in the query molecule, but not found or appearing too infrequently in the training set.

1. All properties and OPS components are within expected ranges.
2. Unknown ECFP_2 feature: -1020449580: [*][c]1:[*]:[*]:[nH]:c:1
3. Unknown ECFP_2 feature: -2097294478: [*]:[c](:[*])C1C[*]=[*]C1
4. Unknown ECFP_2 feature: -116689887: [*][C@H]1[*][*]=NC1
5. Unknown ECFP_2 feature: 103000222: [*]C(=[*])C1=N[*][*]C1
6. Unknown ECFP_2 feature: 1431365708: [*]C([*])C(=O)C(=[*])[*]
7. Unknown ECFP_2 feature: -2095227870: [*]C(=[*])[C@@H]1C[*][*]:[c]1:[*]
8. Unknown ECFP_2 feature: -1457159889: [*][C@H]1[*]:[*]NC1

Feature Contribution

Top features for positive contribution			
Fingerprint	Bit/Smiles	Feature Structure	Score

ECFP_6	655739385	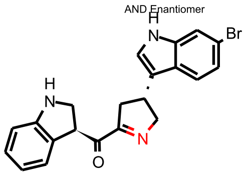 [*]:n:[*]	0.229
ECFP_6	1333660716	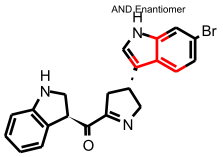 [*]:[cH]:[c]1:[c](:[*]):[*]:[*]:[c]:1:[*]	0.0746
ECFP_6	-167460056	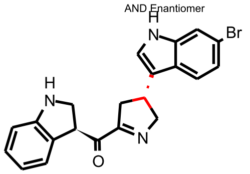 [*]C([*])[*]	0.0596

Top Features for negative contribution

Fingerprint	Bit/Smiles	Feature Structure	Score
ECFP_6	1996767644	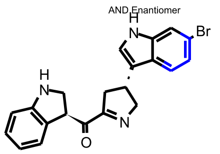 [*]:[cH]:[cH]:[c](:[*]):[*]	-0.251
ECFP_6	642810091	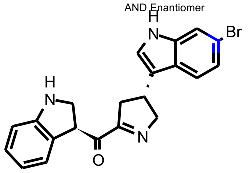 [*]:[c](:[*]):[*]	-0.247

ECFP_6	-182236392	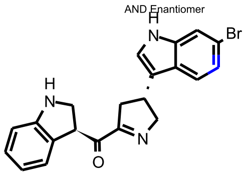<br>[*]:[cH]:[*]	-0.232

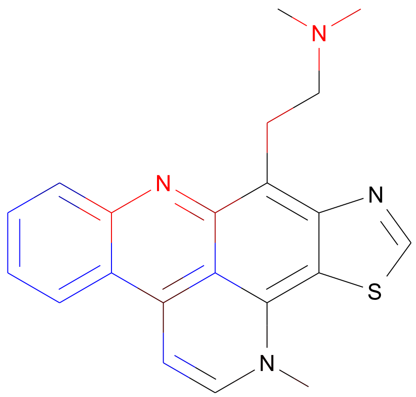

$C_{21}H_{20}N_4S$
Molecular Weight: 360.4753
ALogP: 3.682
Rotatable Bonds: 3
Acceptors: 4
Donors: 0

Structural Similar Compounds

Name	C.I. vat yellow 4	Phenolphthalein	3-(5-Nitro-2-furyl)-imidazo(1,2-a)py-ridine
Structure	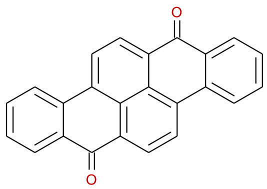	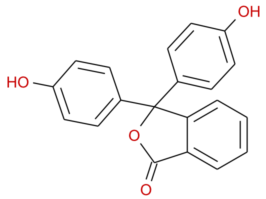	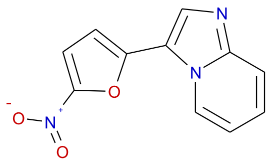
Actual Endpoint (-log C)	1.48417	2.43468	3.92883
Predicted Endpoint (-log C)	4.45029	3.66084	3.65427
Distance	0.727	0.809	0.835
Reference	CPDB	CPDB	CPDB

Model Prediction

Prediction: 0.668
Unit: mg/kg_body_weight/day
Mahalanobis Distance: 11.5
Mahalanobis Distance p-value: 0.000186

Mahalanobis Distance: The Mahalanobis distance (MD) is a generalization of the Euclidean distance that accounts for correlations among the X properties. It is calculated as the distance to the center of the training data. The larger the MD, the less trustworthy the prediction.
Mahalanobis Distance p-value: The p-value gives the fraction of training data with an MD greater than or equal to the one for the given sample, assuming normally distributed data. The smaller the p-value, the less trustworthy the prediciton. For highly non-normal X properties (e.g., fingerprints), the MD p-value is wildly inaccurate.

Model Applicability

Unknown features are fingerprint features in the query molecule, but not found or appearing too infrequently in the training set.

1. All properties and OPS components are within expected ranges.
2. Unknown ECFP_2 feature: -586331102: [*]N([*])[c](:[c](:[*]):[*]):[c](:[*]):[*]
3. Unknown ECFP_2 feature: -1658647648: [*]=C[c](:[c](:[*]):[*]):[c](:[*]):[*]
4. Unknown ECFP_2 feature: -1673960248: [*][c](:[*]):[c]1:s:[*]:[*]:[c]:1:[*]
5. Unknown ECFP_2 feature: 935510419: [*]\C=C/N([*])[*]
6. Unknown ECFP_2 feature: -225243421: [*]1:[*]:s:c:n:1

Feature Contribution

Top features for positive contribution

Fingerprint	Bit/Smiles	Feature Structure	Score
ECFP_6	-1072294614	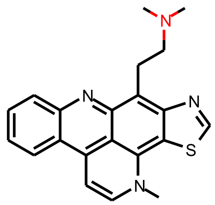 [*]N([*])[*]	0.428

ECFP_6	655739385	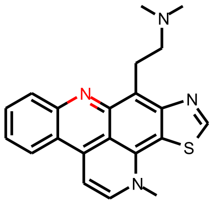 [*]:n:[*]	0.229
ECFP_6	865379614	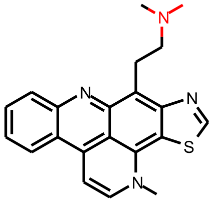 [*]N([*])C	0.219
**Top Features for negative contribution**			
**Fingerprint**	**Bit/Smiles**	**Feature Structure**	**Score**
ECFP_6	1996767644	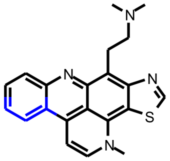 [*]:[cH]:[cH]:[c](:[*<br>]):[*]	-0.251
ECFP_6	642810091	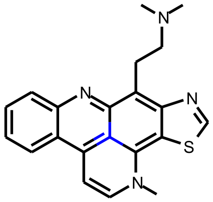 [*]:[c](:[*]):[*]	-0.247
ECFP_6	-182236392	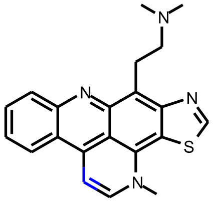 [*]:[cH]:[*]	-0.232

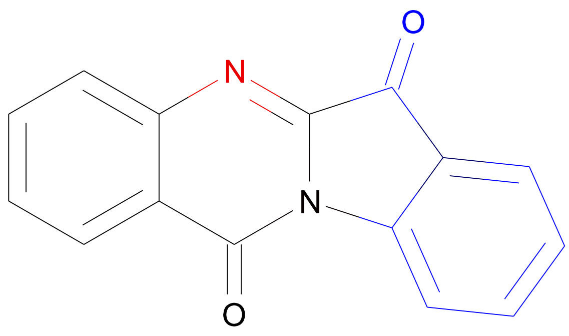

$C_{15}H_8N_2O_2$

Molecular Weight: 248.23621

ALogP: 2.331

Rotatable Bonds: 0

Acceptors: 3

Donors: 0

Model Prediction

Prediction: 53.5

Unit: mg/kg_body_weight/day

Mahalanobis Distance: 8.85

Mahalanobis Distance p-value: 0.356

Mahalanobis Distance: The Mahalanobis distance (MD) is a generalization of the Euclidean distance that accounts for correlations among the X properties. It is calculated as the distance to the center of the training data. The larger the MD, the less trustworthy the prediction.
Mahalanobis Distance p-value: The p-value gives the fraction of training data with an MD greater than or equal to the one for the given sample, assuming normally distributed data. The smaller the p-value, the less trustworthy the prediciton. For highly non-normal X properties (e.g., fingerprints), the MD p-value is wildly inaccurate.

Structural Similar Compounds

Name	Diftalone	2-Aminoanthraquinone	1-Amino-2-methylanthraquinone
Structure	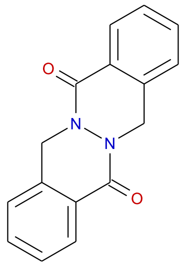	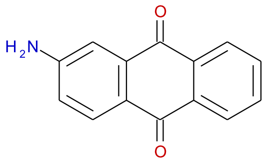	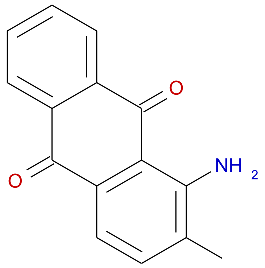
Actual Endpoint (-log C)	2.48505	2.2732	3.13466
Predicted Endpoint (-log C)	3.32164	3.00026	3.25812
Distance	0.494	0.618	0.622
Reference	CPDB	CPDB	CPDB

Model Applicability

Unknown features are fingerprint features in the query molecule, but not found or appearing too infrequently in the training set.

1. All properties and OPS components are within expected ranges.
2. Unknown ECFP_2 feature: -962771238: [*]C(=[*])N1C(=[*])[*][*]:[c]1:[*]
3. Unknown ECFP_2 feature: -1236953626: [*]N1[*][*][c](:[*]):[c]1:c:[*]
4. Unknown ECFP_2 feature: 671679640: [*]\N=C\1/N([*])[*]:[*]C1=[*]
5. Unknown ECFP_2 feature: -597295171: [*][c](:[*]):[c](N=[*]):c:[*]

Feature Contribution

Top features for positive contribution			
Fingerprint	Bit/Smiles	Feature Structure	Score
ECFP_6	655739385	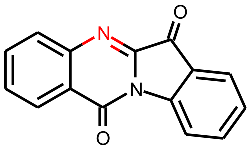 [*]:n:[*]	0.229

ECFP_6	670515721	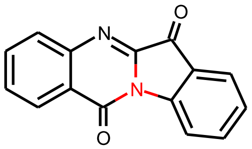 [*]N([*])[*]	0.00735
**Top Features for negative contribution**			
**Fingerprint**	**Bit/Smiles**	**Feature Structure**	**Score**
ECFP_6	2106656448	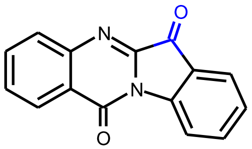 [*]C(=O)[*]	-0.275
ECFP_6	1996767644	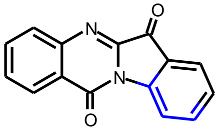 [*]:[cH]:[cH]:[c](:[*])):[*]	-0.251
ECFP_6	642810091	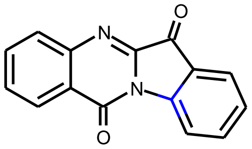 [*]:[c](:[*]):[*]	-0.247

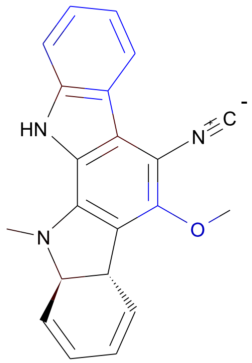

$C_{21}H_{17}N_3O$
Molecular Weight: 327.37918
ALogP: 4.078
Rotatable Bonds: 1
Acceptors: 2
Donors: 1

Model Prediction

Prediction: 9.33
Unit: mg/kg_body_weight/day
Mahalanobis Distance: 13.2
Mahalanobis Distance p-value: 3.55e-008

Mahalanobis Distance: The Mahalanobis distance (MD) is a generalization of the Euclidean distance that accounts for correlations among the X properties. It is calculated as the distance to the center of the training data. The larger the MD, the less trustworthy the prediction.
Mahalanobis Distance p-value: The p-value gives the fraction of training data with an MD greater than or equal to the one for the given sample, assuming normally distributed data. The smaller the p-value, the less trustworthy the prediciton. For highly non-normal X properties (e.g., fingerprints), the MD p-value is wildly inaccurate.

Structural Similar Compounds

Name	429	Phenolphthalein	C.I. vat yellow 4
Structure	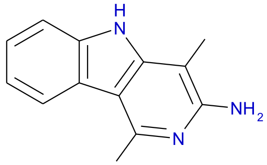	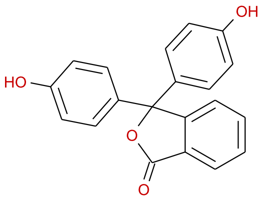	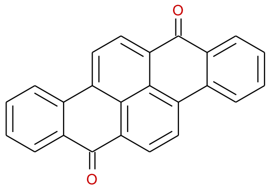
Actual Endpoint (-log C)	3.71523	2.43468	1.48417
Predicted Endpoint (-log C)	4.22904	3.66084	4.45029
Distance	0.693	0.714	0.719
Reference	CPDB	CPDB	CPDB

Model Applicability

Unknown features are fingerprint features in the query molecule, but not found or appearing too infrequently in the training set.

1. All properties and OPS components are within expected ranges.
2. Unknown ECFP_2 feature: 726108635: [*]#[C-]
3. Unknown ECFP_2 feature: 1464683384: [*][c](:[*]):[c]([N+]#[*]):[c](:[*]):[*]
4. Unknown ECFP_2 feature: -586331102: [*]N([*])[c](:[c](:[*]):[*]):[c](:[*]):[*]
5. Unknown ECFP_2 feature: 1617733200: [*][C@@H]1[*][*]:[c](:[*])N1C
6. Unknown ECFP_2 feature: -1337975340: [*][C@H]1[*]:[*]N([*])[C@@H]1C=[*]
7. Unknown ECFP_2 feature: -11961319: [*]:[c](:[*])[N+]#[C-]
8. Unknown ECFP_2 feature: -1334780583: [*][N+]#[C-]

Feature Contribution

Top features for positive contribution			
Fingerprint	Bit/Smiles	Feature Structure	Score

ECFP_6	1333660716	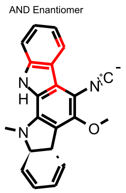 [*]:[cH]:[c]1:[c](:[*]):[*]:[*]:[c]:1:[*]	0.0746
ECFP_6	-167460056	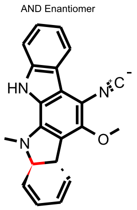 [*]C([*])[*]	0.0596
ECFP_6	734603939	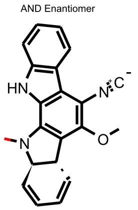 [*]C	0.0424

Top Features for negative contribution

Fingerprint	Bit/Smiles	Feature Structure	Score
ECFP_6	1996767644	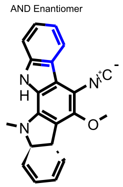 [*]:[cH]:[cH]:[c](:[*]):[*]	-0.251
ECFP_6	642810091	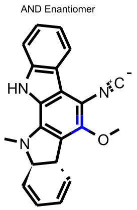 [*]:[c](:[*]):[*]	-0.247

ECFP_6	-182236392	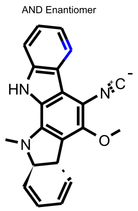 [*]:[cH]:[*]	-0.232

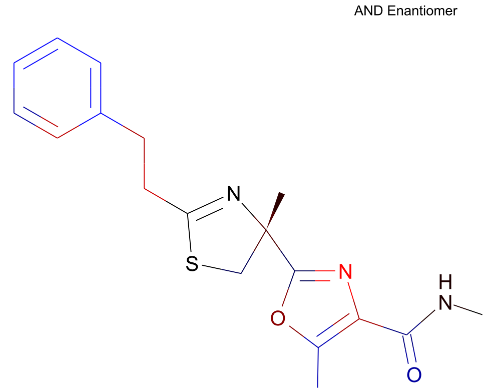

$C_{18}H_{21}N_3O_2S$

Molecular Weight: 343.44324

ALogP: 2.716

Rotatable Bonds: 5

Acceptors: 4

Donors: 1

Model Prediction

Prediction: 21.2

Unit: mg/kg_body_weight/day

Mahalanobis Distance: 12.7

Mahalanobis Distance p-value: 7.63e-007

Mahalanobis Distance: The Mahalanobis distance (MD) is a generalization of the Euclidean distance that accounts for correlations among the X properties. It is calculated as the distance to the center of the training data. The larger the MD, the less trustworthy the prediction.
Mahalanobis Distance p-value: The p-value gives the fraction of training data with an MD greater than or equal to the one for the given sample, assuming normally distributed data. The smaller the p-value, the less trustworthy the prediciton. For highly non-normal X properties (e.g., fingerprints), the MD p-value is wildly inaccurate.

Structural Similar Compounds

Name	C.I. pigment red 3	455	171
Structure	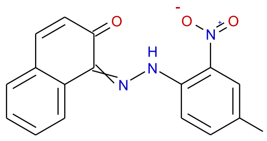	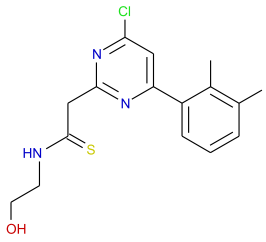	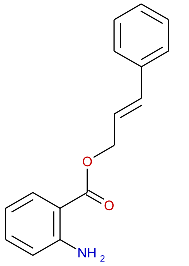
Actual Endpoint (-log C)	0.937339	3.87681	1.99201
Predicted Endpoint (-log C)	3.17837	3.77582	3.01089
Distance	0.618	0.651	0.667
Reference	CPDB	CPDB	CPDB

Model Applicability

Unknown features are fingerprint features in the query molecule, but not found or appearing too infrequently in the training set.

1. All properties and OPS components are within expected ranges.
2. Unknown ECFP_2 feature: 309047694: [*]C([*])([*])[c]1:o:[*]:[*]:n:1
3. Unknown ECFP_2 feature: 1576608821: [*][c]1:[*]:[*]:o:[c]:1C
4. Unknown ECFP_2 feature: 2127097785: [*]C1=[*][*]CS1
5. Unknown ECFP_2 feature: -1073216586: [*]CC1=N[*][*]S1
6. Unknown ECFP_2 feature: 1920241679: [*]C1([*])[*]=[*]SC1
7. Unknown ECFP_2 feature: 618128563: [*]:[c](:[*])[C@]1(C)C[*][*]=N1
8. Unknown ECFP_2 feature: 2085336332: [*]C1=NC([*])([*])[*][*]1
9. Unknown ECFP_2 feature: 1338334141: [*]C(=[*])NC

Feature Contribution

Top features for positive contribution			
Fingerprint	Bit/Smiles	Feature Structure	Score

ECFP_6	655739385	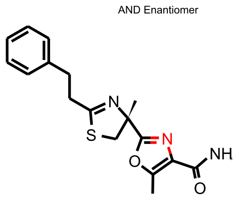 [*]:n:[*]	0.229
ECFP_6	1559650422	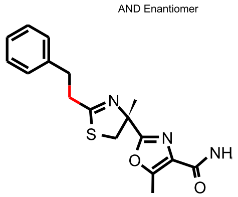 [*]C[*]	0.203
ECFP_6	-2024255407	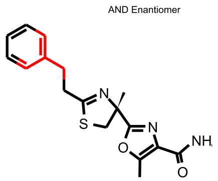 [*]C[c](:[cH]:[*]):[cH]:[*]	0.172
**Top Features for negative contribution**			
**Fingerprint**	**Bit/Smiles**	**Feature Structure**	**Score**
ECFP_6	1996767644	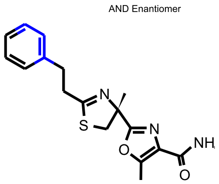 [*]:[cH]:[cH]:[c](:[*]):[*]	-0.251
ECFP_6	642810091	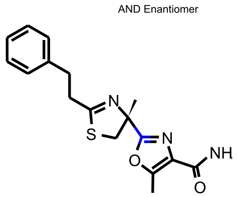 [*]:[c](:[*]):[*]	-0.247

ECFP_6	-182236392	AND Enantiomer 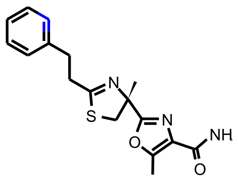 [*]:[cH]:[*]	-0.232

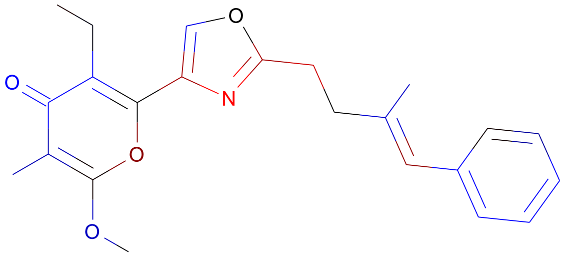

$C_{23}H_{25}NO_4$
Molecular Weight: 379.4489
ALogP: 5.22
Rotatable Bonds: 7
Acceptors: 4
Donors: 0

Model Prediction

Prediction: 78.2
Unit: mg/kg_body_weight/day
Mahalanobis Distance: 16.6
Mahalanobis Distance p-value: 4.01e-018

Mahalanobis Distance: The Mahalanobis distance (MD) is a generalization of the Euclidean distance that accounts for correlations among the X properties. It is calculated as the distance to the center of the training data. The larger the MD, the less trustworthy the prediction.
Mahalanobis Distance p-value: The p-value gives the fraction of training data with an MD greater than or equal to the one for the given sample, assuming normally distributed data. The smaller the p-value, the less trustworthy the prediciton. For highly non-normal X properties (e.g., fingerprints), the MD p-value is wildly inaccurate.

Structural Similar Compounds

Name	Phenylbutazone	Methyl clofenapate	Chlorobenzilate
Structure	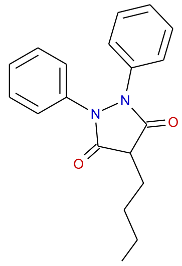	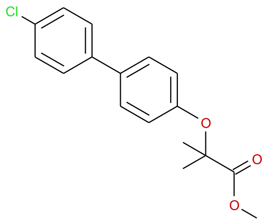	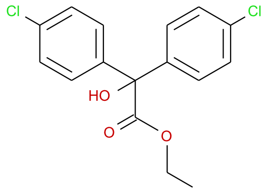
Actual Endpoint (-log C)	2.9413	5.0843	3.53947
Predicted Endpoint (-log C)	3.29616	3.36581	3.34564
Distance	0.662	0.681	0.682
Reference	CPDB	CPDB	CPDB

Model Applicability

Unknown features are fingerprint features in the query molecule, but not found or appearing too infrequently in the training set.

1. All properties and OPS components are within expected ranges.
2. Unknown ECFP_2 feature: -770854792: [*]CC(=C([*])[*])C(=[*])[*]
3. Unknown ECFP_2 feature: 1796421070: [*]OC(=C([*])[*])[c](:[*]):[*]
4. Unknown ECFP_2 feature: -435589429: [*]C[c]1:o:[*]:[*]:n:1
5. Unknown ECFP_2 feature: 431707261: [*]C\C(=C\[*])\C
6. Unknown ECFP_2 feature: 1792159373: [*]C(=C(C)C(=[*])[*])[*]
7. Unknown ECFP_2 feature: 1651701028: [*]OC(=C([*])[*])O[*]
8. Unknown ECFP_2 feature: -785659985: [*][c]1:[*]:[*]:o:c:1

Feature Contribution

Top features for positive contribution

Fingerprint	Bit/Smiles	Feature Structure	Score

ECFP_6	655739385	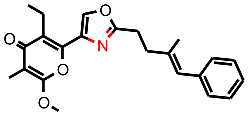 [*]:n:[*]	0.229
ECFP_6	1559650422	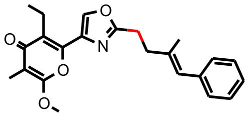 [*]C[*]	0.203
ECFP_6	834876373	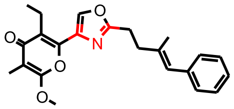 [*]:[c](:[*]):n:[c](:[*]):[*]	0.163

Top Features for negative contribution

Fingerprint	Bit/Smiles	Feature Structure	Score
ECFP_6	2106656448	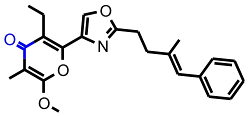 [*]C(=O)[*]	-0.275
ECFP_6	1996767644	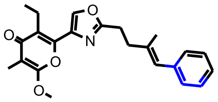 [*]:[cH]:[cH]:[c](:[*]):[*]	-0.251

ECFP_6	642810091	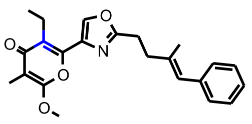<br>[*]:[c](:[*]):[*]	-0.247

Remdesivir

TOPKAT_Carcinogenic_Potency_TD50_Mouse

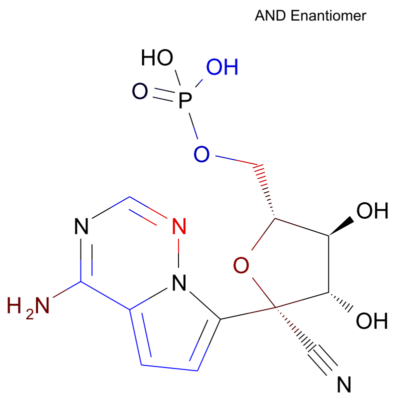

$C_{12}H_{14}N_5O_7P$
Molecular Weight: 371.24262
ALogP: -1.502
Rotatable Bonds: 4
Acceptors: 11
Donors: 5

Model Prediction

Prediction: 9.25
Unit: mg/kg_body_weight/day
Mahalanobis Distance: 14
Mahalanobis Distance p-value: 2.59e-010

Mahalanobis Distance: The Mahalanobis distance (MD) is a generalization of the Euclidean distance that accounts for correlations among the X properties. It is calculated as the distance to the center of the training data. The larger the MD, the less trustworthy the prediction.
Mahalanobis Distance p-value: The p-value gives the fraction of training data with an MD greater than or equal to the one for the given sample, assuming normally distributed data. The smaller the p-value, the less trustworthy the prediciton. For highly non-normal X properties (e.g., fingerprints), the MD p-value is wildly inaccurate.

Structural Similar Compounds

Name	377	(N-6)-(Methylnitroso)adenosine	338
Structure	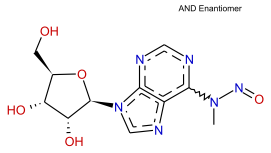	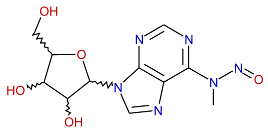	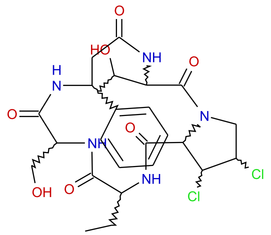
Actual Endpoint (-log C)	4.22928	4.22928	4.39533
Predicted Endpoint (-log C)	5.36013	5.36013	4.31268
Distance	0.852	0.852	0.919
Reference	CPDB	CPDB	CPDB

Model Applicability

Unknown features are fingerprint features in the query molecule, but not found or appearing too infrequently in the training set.

1. All properties and OPS components are within expected ranges.
2. Unknown ECFP_2 feature: 1126642748: [*]OP(=O)(O)O
3. Unknown ECFP_2 feature: 2024329577: [*]P(=[*])([*])O
4. Unknown ECFP_2 feature: -194719409: [*][C@@H]1[*][*]C([*])([*])O1
5. Unknown ECFP_2 feature: 1258791451: [*][C@H]1[*][*]O[C@]1(C#[*])[c](:[*]):[*]
6. Unknown ECFP_2 feature: -264833661: [*]C([*])([*])C#N
7. Unknown ECFP_2 feature: -1507082173: [*][c]1:[*]:[*]:[c](:[*]):n:1:n:[*]
8. Unknown ECFP_2 feature: -676555381: [*]:n(:[*]):n:c:[*]
9. Unknown ECFP_2 feature: -66263742: [*]C([*])([*])[c]1:n(:[*]):[*]:[*]:c:1

Feature Contribution

Top features for positive contribution			
Fingerprint	Bit/Smiles	Feature Structure	Score

ECFP_6	655739385	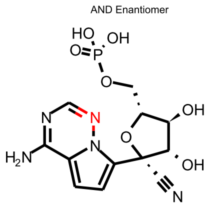 [*]:n:[*]	0.229
ECFP_6	1572579716	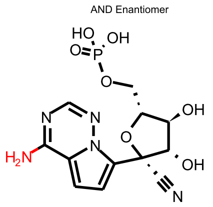 [*]N	0.225
ECFP_6	1559650422	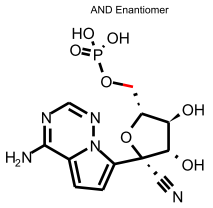 [*]C[*]	0.203

Top Features for negative contribution

**Fingerprint**	**Bit/Smiles**	**Feature Structure**	**Score**
ECFP_6	1996767644	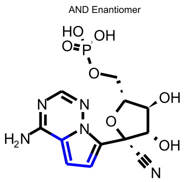 [*]:[cH]:[cH]:[c](:[*]):[*]	-0.251
ECFP_6	642810091	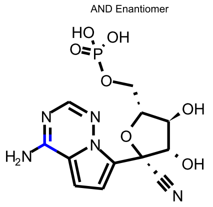 [*]:[c](:[*]):[*]	-0.247

ECFP_6	-182236392	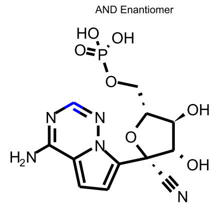[*]:[cH]:[*]	-0.232

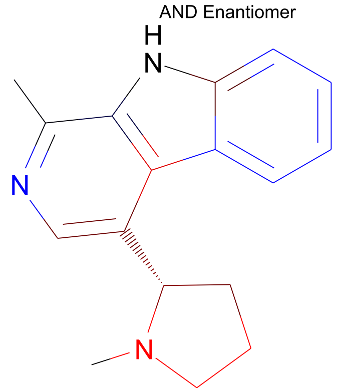

$C_{17}H_{19}N_3$
Molecular Weight: 265.35286
ALogP: 3.018
Rotatable Bonds: 1
Acceptors: 2
Donors: 1

Model Prediction

Prediction: 1.7
Unit: mg/kg_body_weight/day
Mahalanobis Distance: 13.6
Mahalanobis Distance p-value: 2.57e-007

Mahalanobis Distance: The Mahalanobis distance (MD) is a generalization of the Euclidean distance that accounts for correlations among the X properties. It is calculated as the distance to the center of the training data. The larger the MD, the less trustworthy the prediction.
Mahalanobis Distance p-value: The p-value gives the fraction of training data with an MD greater than or equal to the one for the given sample, assuming normally distributed data. The smaller the p-value, the less trustworthy the prediciton. For highly non-normal X properties (e.g., fingerprints), the MD p-value is wildly inaccurate.

Structural Similar Compounds

Name	429	1140	964
Structure	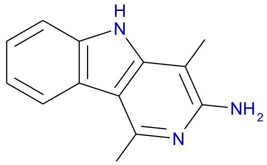	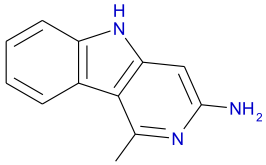	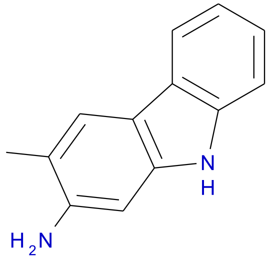
Actual Endpoint (-log C)	5.56515	4.47151	4.46673
Predicted Endpoint (-log C)	4.07595	3.89064	3.95814
Distance	0.548	0.567	0.569
Reference	CPDB	CPDB	CPDB

Model Applicability

Unknown features are fingerprint features in the query molecule, but not found or appearing too infrequently in the training set.

1. All properties and OPS components are within expected ranges.

Feature Contribution

Top features for positive contribution			
**Fingerprint**	**Bit/Smiles**	**Feature Structure**	**Score**
FCFP_6	9	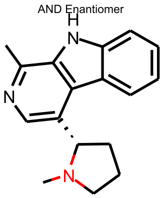 AND Enantiomer [*]N([*])[*]	0.385

FCFP_6	-587569116	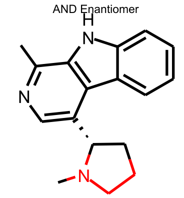 [*]N1[*][*]CC1	0.319
FCFP_6	203677720	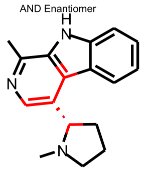 [*]C([*])[c](:[cH]:[*]):[c](:[*]):[*]	0.137
**Top Features for negative contribution**			
**Fingerprint**	**Bit/Smiles**	**Feature Structure**	**Score**
FCFP_6	991735244	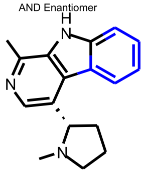 [*]:[c]1:[*]:[cH]:[cH]:[cH]:[cH]:1	-0.422
FCFP_6	16	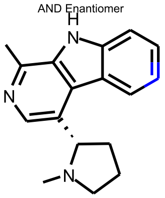 [*]:[cH]:[*]	-0.354
FCFP_6	17	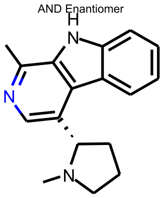 [*]:n:[*]	-0.149

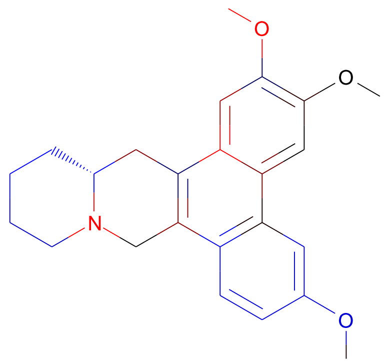

$C_{24}H_{27}NO_3$
Molecular Weight: 377.47608
ALogP: 4.691
Rotatable Bonds: 3
Acceptors: 4
Donors: 0

Model Prediction

Prediction: 0.112
Unit: mg/kg_body_weight/day
Mahalanobis Distance: 14.8
Mahalanobis Distance p-value: 1.61e-010

Mahalanobis Distance: The Mahalanobis distance (MD) is a generalization of the Euclidean distance that accounts for correlations among the X properties. It is calculated as the distance to the center of the training data. The larger the MD, the less trustworthy the prediction.
Mahalanobis Distance p-value: The p-value gives the fraction of training data with an MD greater than or equal to the one for the given sample, assuming normally distributed data. The smaller the p-value, the less trustworthy the prediciton. For highly non-normal X properties (e.g., fingerprints), the MD p-value is wildly inaccurate.

Structural Similar Compounds

Name	Acronycine	357	4-(4-N-Methyl-N-nitrosamino-styryl)quinoline
Structure	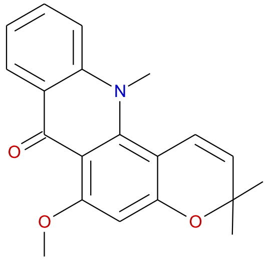	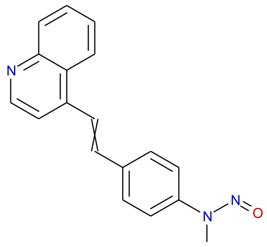	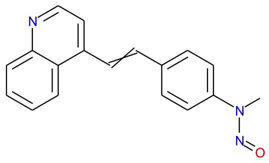
Actual Endpoint (-log C)	5.80371	5.61692	5.61692
Predicted Endpoint (-log C)	4.88757	5.49128	5.49128
Distance	0.658	0.708	0.708
Reference	CPDB	CPDB	CPDB

Model Applicability

Unknown features are fingerprint features in the query molecule, but not found or appearing too infrequently in the training set.

1. All properties and OPS components are within expected ranges.

Feature Contribution

Top features for positive contribution			
Fingerprint	Bit/Smiles	Feature Structure	Score
FCFP_6	136627117	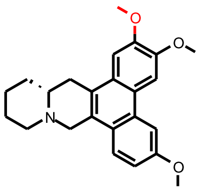 AND Enantiomer [*]OC	0.69

FCFP_6	9	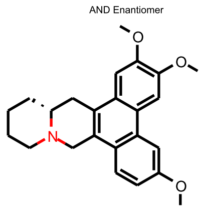 [*]N([*])[*]	0.385
FCFP_6	-587569116	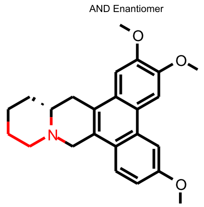 [*]N1[*][*]CC1	0.319

Top Features for negative contribution

Fingerprint	Bit/Smiles	Feature Structure	Score
FCFP_6	1175638033	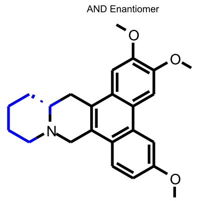 [*][C@@H]1[*]CCCC1	-0.512
FCFP_6	16	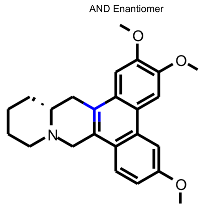 [*]:[cH]:[*]	-0.354
FCFP_6	1674451008	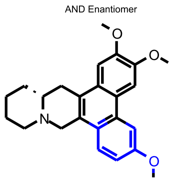 [*]O[c]1:[cH]:[*]:[c]<br>(:[*]):[cH]:[cH]:1	-0.233

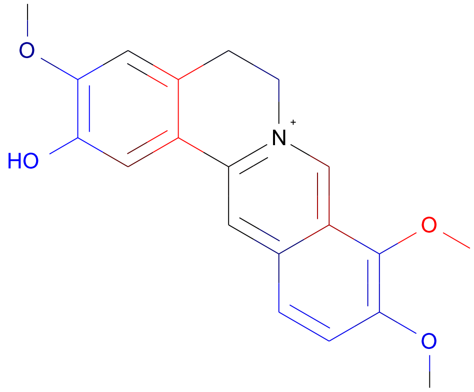

$C_{20}H_{20}NO_4$
Molecular Weight: 338.3771
ALogP: 3.936
Rotatable Bonds: 3
Acceptors: 4
Donors: 1

Model Prediction

Prediction: 1.85
Unit: mg/kg_body_weight/day
Mahalanobis Distance: 15.3
Mahalanobis Distance p-value: 4.19e-012

Mahalanobis Distance: The Mahalanobis distance (MD) is a generalization of the Euclidean distance that accounts for correlations among the X properties. It is calculated as the distance to the center of the training data. The larger the MD, the less trustworthy the prediction.
Mahalanobis Distance p-value: The p-value gives the fraction of training data with an MD greater than or equal to the one for the given sample, assuming normally distributed data. The smaller the p-value, the less trustworthy the prediciton. For highly non-normal X properties (e.g., fingerprints), the MD p-value is wildly inaccurate.

Structural Similar Compounds

Name	Indomethacin	Phenolphthalein	3-(Cyclopentyloxy)-N-(3,5-di-chloro-4-pyridyl)-4-methoxy-benzamide
Structure	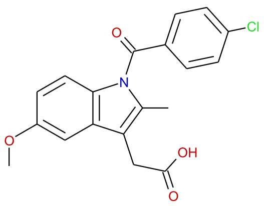	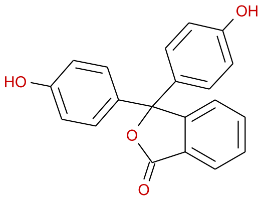	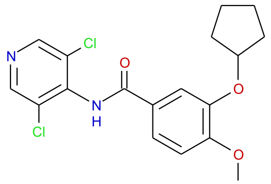
Actual Endpoint (-log C)	5.49293	2.54766	5.39369
Predicted Endpoint (-log C)	4.9569	3.7508	4.27874
Distance	0.573	0.605	0.628
Reference	CPDB	CPDB	CPDB

Model Applicability

Unknown features are fingerprint features in the query molecule, but not found or appearing too infrequently in the training set.

1. All properties and OPS components are within expected ranges.
2. Unknown FCFP_2 feature: 414371600: [*]C[n+](:[c]([*]):[*]):c:[*]
3. Unknown FCFP_2 feature: -150573739: [*]CC[n+](:[*]):[*]
4. Unknown FCFP_2 feature: -1861407456: [*][n+](:[*]):[c]([c](:[*]):[*]):c:[*]

Feature Contribution

Top features for positive contribution			
**Fingerprint**	**Bit/Smiles**	**Feature Structure**	**Score**
FCFP_6	136627117	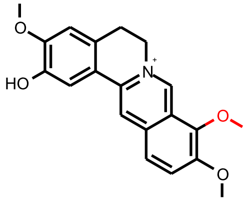 [*]OC	0.69

FCFP_6	-1861645784	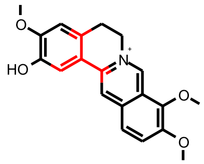 [*][c](:[*]):[c](:[cH]:[*])[c](:[*]):[*]	0.359
FCFP_6	1	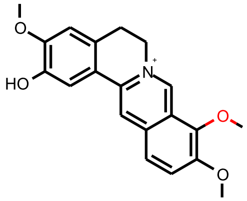 [*]O[*]	0.234

Top Features for negative contribution

Fingerprint	Bit/Smiles	Feature Structure	Score
FCFP_6	7	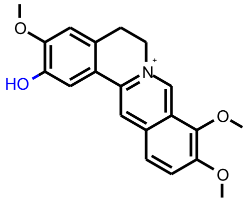 [*]O	-0.372
FCFP_6	16	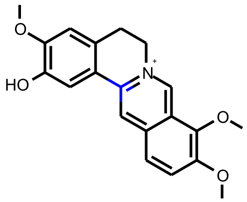 [*]:[cH]:[*]	-0.354
FCFP_6	74595001	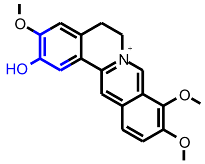 [*][c](:[*]):[c](O):[cH]:[*]	-0.267

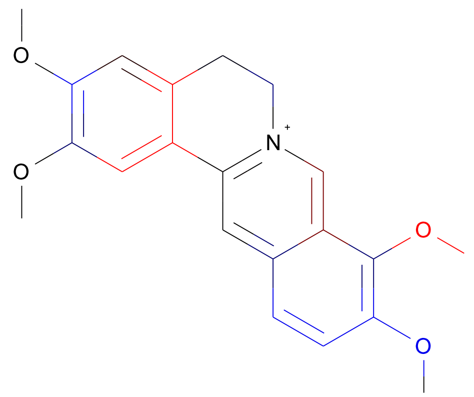

$C_{21}H_{22}NO_4$
Molecular Weight: 352.40368
ALogP: 4.161
Rotatable Bonds: 4
Acceptors: 4
Donors: 0

Model Prediction

Prediction: 0.474
Unit: mg/kg_body_weight/day
Mahalanobis Distance: 13.9
Mahalanobis Distance p-value: 4.58e-008

Mahalanobis Distance: The Mahalanobis distance (MD) is a generalization of the Euclidean distance that accounts for correlations among the X properties. It is calculated as the distance to the center of the training data. The larger the MD, the less trustworthy the prediction.
Mahalanobis Distance p-value: The p-value gives the fraction of training data with an MD greater than or equal to the one for the given sample, assuming normally distributed data. The smaller the p-value, the less trustworthy the prediciton. For highly non-normal X properties (e.g., fingerprints), the MD p-value is wildly inaccurate.

Structural Similar Compounds

Name	357	4-(4-N-Methyl-N-nitrosamino-styryl)quinoline	Acronycine
Structure	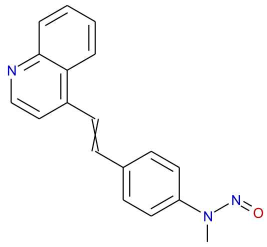	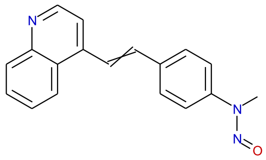	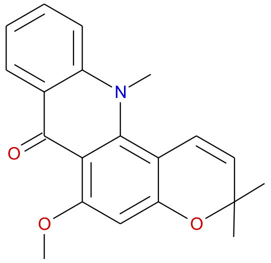
Actual Endpoint (-log C)	5.61692	5.61692	5.80371
Predicted Endpoint (-log C)	5.49128	5.49128	4.88757
Distance	0.597	0.597	0.637
Reference	CPDB	CPDB	CPDB

Model Applicability

Unknown features are fingerprint features in the query molecule, but not found or appearing too infrequently in the training set.

1. All properties and OPS components are within expected ranges.
2. Unknown FCFP_2 feature: 414371600: [*]C[n+](:[c](:[c]([*]):[*]):c:[*]
3. Unknown FCFP_2 feature: -150573739: [*]CC[n+](:[*]):[*]
4. Unknown FCFP_2 feature: -1861407456: [*][n+](:[*]):[c]([c](:[*]):[*]):c:[*]

Feature Contribution

Top features for positive contribution

Fingerprint	Bit/Smiles	Feature Structure	Score
FCFP_6	136627117	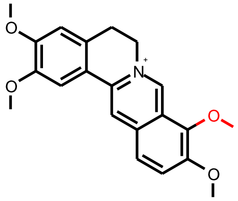 [*]OC	0.69

FCFP_6	-1861645784	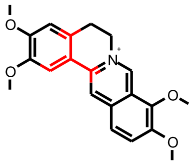<br>[*][c](:[*]):[c](:[cH<br>]:[*])[c](:[*]):[*]	0.359
FCFP_6	1	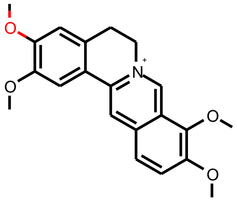<br>[*]O[*]	0.234
**Top Features for negative contribution**			
**Fingerprint**	**Bit/Smiles**	**Feature Structure**	**Score**
FCFP_6	16	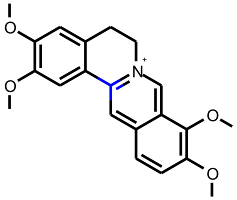<br>[*]:[cH]:[*]	-0.354
FCFP_6	1674451008	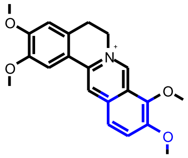<br>[*]O[c]1:[cH]:[*]:[c]<br>(:[*]):[cH]:[cH]:1	-0.233
FCFP_6	1676877079	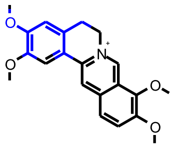<br>[*]C[c]1:[cH]:[c](O[*<br>]):[c]([*]):[*]:[c]:<br>1[*]	-0.146

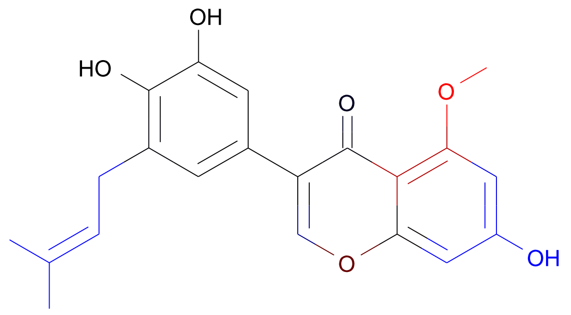

$C_{21}H_{20}O_6$
Molecular Weight: 368.3799
ALogP: 3.98
Rotatable Bonds: 4
Acceptors: 6
Donors: 3

Model Prediction

Prediction: 28
Unit: mg/kg_body_weight/day
Mahalanobis Distance: 14
Mahalanobis Distance p-value: 2.77e-008

Mahalanobis Distance: The Mahalanobis distance (MD) is a generalization of the Euclidean distance that accounts for correlations among the X properties. It is calculated as the distance to the center of the training data. The larger the MD, the less trustworthy the prediction.
Mahalanobis Distance p-value: The p-value gives the fraction of training data with an MD greater than or equal to the one for the given sample, assuming normally distributed data. The smaller the p-value, the less trustworthy the prediciton. For highly non-normal X properties (e.g., fingerprints), the MD p-value is wildly inaccurate.

Structural Similar Compounds

Name	542	Ochratoxin A	4-Chloro-6-(2,3-xylidino)-2-pyri-mi-dinylthio(N-b-hydroxy-ethyl) acetamide
Structure	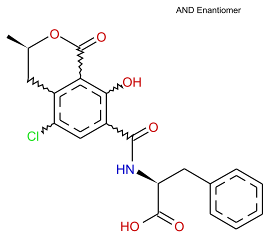	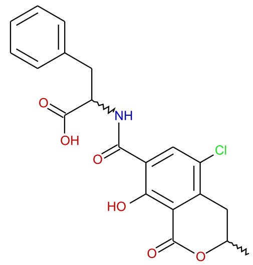	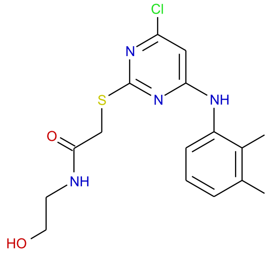
Actual Endpoint (-log C)	6.59334	6.47264	4.75226
Predicted Endpoint (-log C)	5.06501	5.06501	3.29421
Distance	0.553	0.553	0.642
Reference	CPDB	CPDB	CPDB

Model Applicability

Unknown features are fingerprint features in the query molecule, but not found or appearing too infrequently in the training set.

1. All properties and OPS components are within expected ranges.

Feature Contribution

Top features for positive contribution

Fingerprint	Bit/Smiles	Feature Structure	Score
FCFP_6	136627117	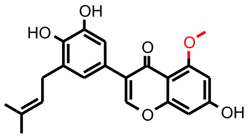 [*]OC	0.69

FCFP_6	1	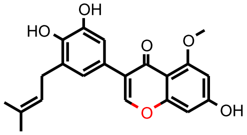<br>[*]O[*]	0.234
FCFP_6	203677720	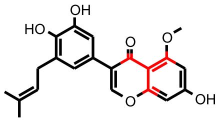<br>[*]C([*])[c](:[cH]:[*]):[c](:[*]):[*]	0.137

Top Features for negative contribution

**Fingerprint**	**Bit/Smiles**	**Feature Structure**	**Score**
FCFP_6	451847724	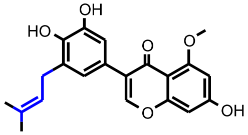<br>[*]CC=C([*])[*]	-0.436
FCFP_6	436886043	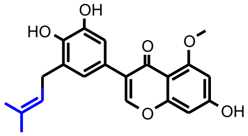<br>[*]C=C(C)C	-0.383
FCFP_6	7	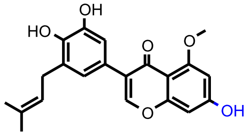<br>[*]O	-0.372

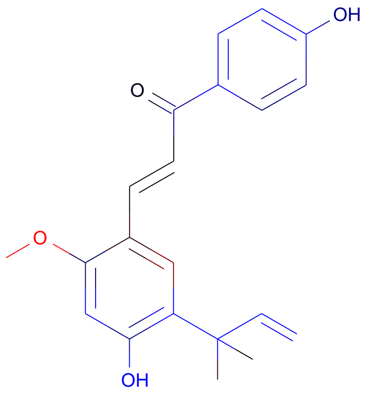

$C_{21}H_{22}O_4$
Molecular Weight: 338.39698
ALogP: 4.667
Rotatable Bonds: 6
Acceptors: 4
Donors: 2

Model Prediction

Prediction: 46.1
Unit: mg/kg_body_weight/day
Mahalanobis Distance: 11.5
Mahalanobis Distance p-value: 0.00382

Mahalanobis Distance: The Mahalanobis distance (MD) is a generalization of the Euclidean distance that accounts for correlations among the X properties. It is calculated as the distance to the center of the training data. The larger the MD, the less trustworthy the prediction.
Mahalanobis Distance p-value: The p-value gives the fraction of training data with an MD greater than or equal to the one for the given sample, assuming normally distributed data. The smaller the p-value, the less trustworthy the prediciton. For highly non-normal X properties (e.g., fingerprints), the MD p-value is wildly inaccurate.

Structural Similar Compounds

Name	455	5	Diethylstilbestrol
Structure	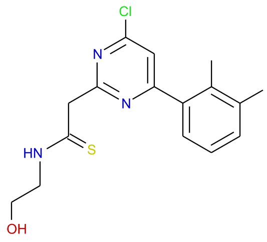	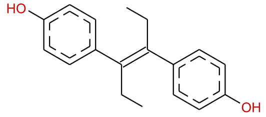	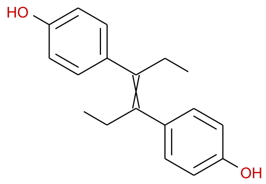
Actual Endpoint (-log C)	4.7139	6.0804	6.0804
Predicted Endpoint (-log C)	3.94556	3.53032	3.53032
Distance	0.579	0.597	0.597
Reference	CPDB	CPDB	CPDB

Model Applicability

Unknown features are fingerprint features in the query molecule, but not found or appearing too infrequently in the training set.

1. All properties and OPS components are within expected ranges.

Feature Contribution

Top features for positive contribution			
**Fingerprint**	**Bit/Smiles**	**Feature Structure**	**Score**
FCFP_6	136627117	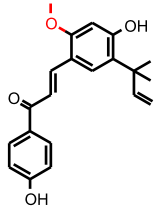 [*]OC	0.69

FCFP_6	1	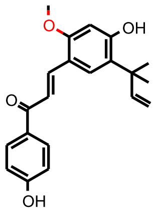 [*]O[*]	0.234
FCFP_6	203677720	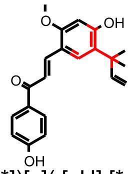 [*]C([*])[c](:[cH]:[*]):[c](:[*]):[*]	0.137
**Top Features for negative contribution**			
**Fingerprint**	**Bit/Smiles**	**Feature Structure**	**Score**
FCFP_6	451847724	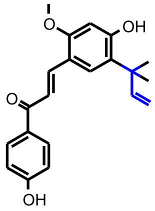 [*]CC=C([*])[*]	-0.436
FCFP_6	7	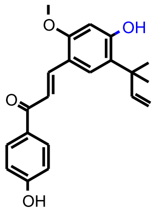 [*]O	-0.372
FCFP_6	16	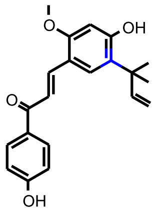 [*]:[cH]:[*]	-0.354

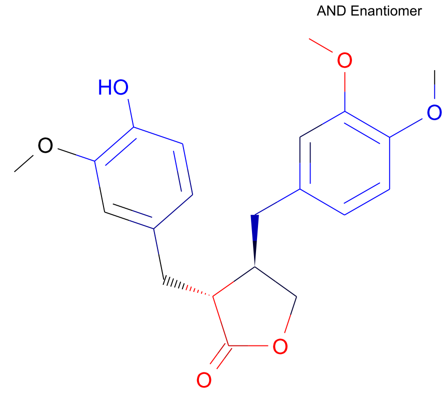

$C_{21}H_{24}O_6$
Molecular Weight: 372.41166
ALogP: 3.743
Rotatable Bonds: 7
Acceptors: 6
Donors: 1

Model Prediction

Prediction: 15.2
Unit: mg/kg_body_weight/day
Mahalanobis Distance: 13.5
Mahalanobis Distance p-value: 3.27e-007

Mahalanobis Distance: The Mahalanobis distance (MD) is a generalization of the Euclidean distance that accounts for correlations among the X properties. It is calculated as the distance to the center of the training data. The larger the MD, the less trustworthy the prediction.
Mahalanobis Distance p-value: The p-value gives the fraction of training data with an MD greater than or equal to the one for the given sample, assuming normally distributed data. The smaller the p-value, the less trustworthy the prediciton. For highly non-normal X properties (e.g., fingerprints), the MD p-value is wildly inaccurate.

Structural Similar Compounds

Name	3-(Cyclopentyloxy)-N-(3,5-di-chloro-4-pyridyl)-4-methoxy-benzamide	FD & C violet no. 1	3,3´-Dimethoxybenzidine-4,4´-diisocyanate
Structure	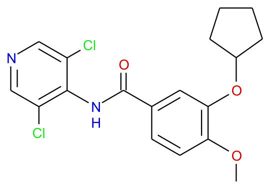	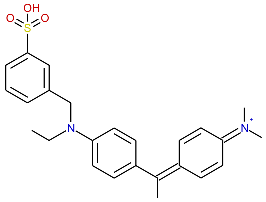	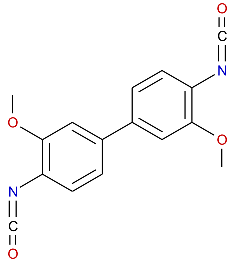
Actual Endpoint (-log C)	5.39369	2.8543	2.25951
Predicted Endpoint (-log C)	4.27874	3.40838	4.84384
Distance	0.562	0.627	0.629
Reference	CPDB	CPDB	CPDB

Model Applicability

Unknown features are fingerprint features in the query molecule, but not found or appearing too infrequently in the training set.

1. All properties and OPS components are within expected ranges.

Feature Contribution

Top features for positive contribution			
**Fingerprint**	**Bit/Smiles**	**Feature Structure**	**Score**
FCFP_6	136627117	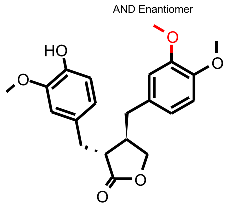 [*]OC	0.69

FCFP_6	565998553	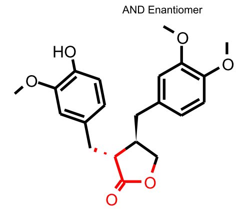<br>[*][C@@H]1[*][*]OC1=O	0.357
FCFP_6	1	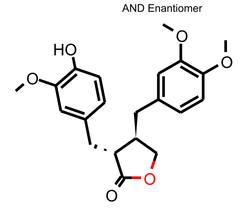<br>[*]O[*]	0.234

Top Features for negative contribution

Fingerprint	Bit/Smiles	Feature Structure	Score
FCFP_6	7	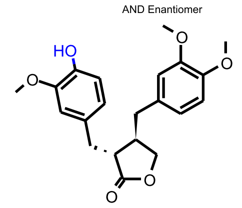<br>[*]O	-0.372
FCFP_6	16	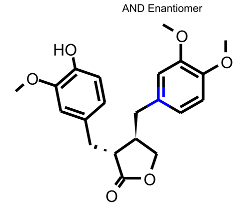<br>[*]:[cH]:[*]	-0.354
FCFP_6	74595001	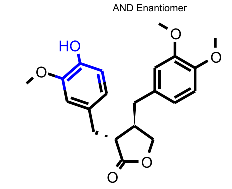<br>[*][c](:[*]):[c](O):[cH]:[*]	-0.267

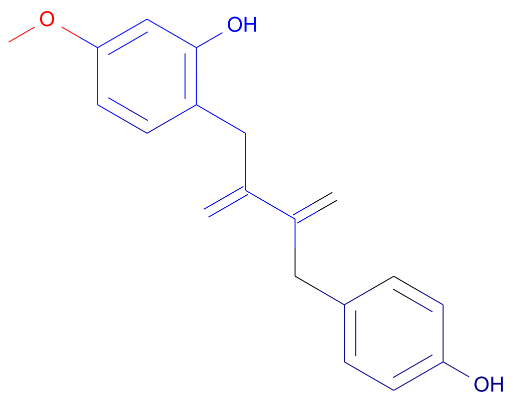

$C_{19}H_{20}O_3$
Molecular Weight: 296.3603
ALogP: 4.784
Rotatable Bonds: 6
Acceptors: 3
Donors: 2

Structural Similar Compounds

Name	Diethylstilbestrol	5	Nafenopin s
Structure	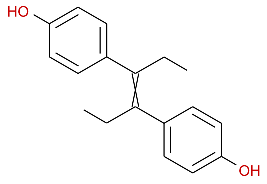	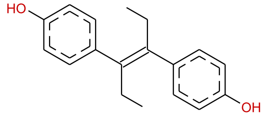	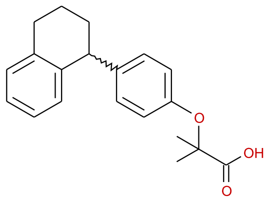
Actual Endpoint (-log C)	6.0804	6.0804	4.45051
Predicted Endpoint (-log C)	3.53032	3.53032	3.8403
Distance	0.494	0.494	0.586
Reference	CPDB	CPDB	CPDB

Model Prediction

Prediction: 62.1
Unit: mg/kg_body_weight/day
Mahalanobis Distance: 13.9
Mahalanobis Distance p-value: 4.77e-008

Mahalanobis Distance: The Mahalanobis distance (MD) is a generalization of the Euclidean distance that accounts for correlations among the X properties. It is calculated as the distance to the center of the training data. The larger the MD, the less trustworthy the prediction.
Mahalanobis Distance p-value: The p-value gives the fraction of training data with an MD greater than or equal to the one for the given sample, assuming normally distributed data. The smaller the p-value, the less trustworthy the prediciton. For highly non-normal X properties (e.g., fingerprints), the MD p-value is wildly inaccurate.

Model Applicability

Unknown features are fingerprint features in the query molecule, but not found or appearing too infrequently in the training set.

1. All properties and OPS components are within expected ranges.

Feature Contribution

Top features for positive contribution

Fingerprint	Bit/Smiles	Feature Structure	Score
FCFP_6	136627117	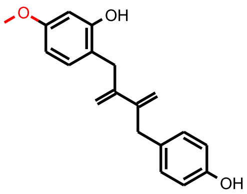 [*]OC	0.69

FCFP_6	1	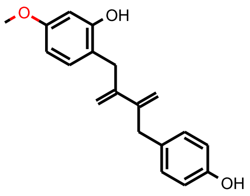 [*]O[*]	0.234
FCFP_6	203677720	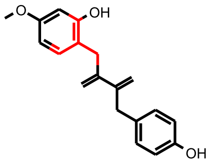 [*]C([*])[c](:[cH]:[*]):[c](:[*]):[*]	0.137
**Top Features for negative contribution**			
**Fingerprint**	**Bit/Smiles**	**Feature Structure**	**Score**
FCFP_6	436886043	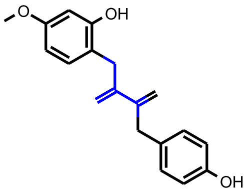 [*]C=C(C)C	-0.383
FCFP_6	7	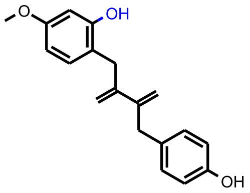 [*]O	-0.372
FCFP_6	16	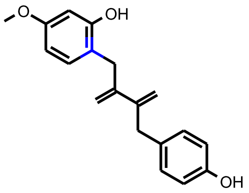 [*]:[cH]:[*]	-0.354

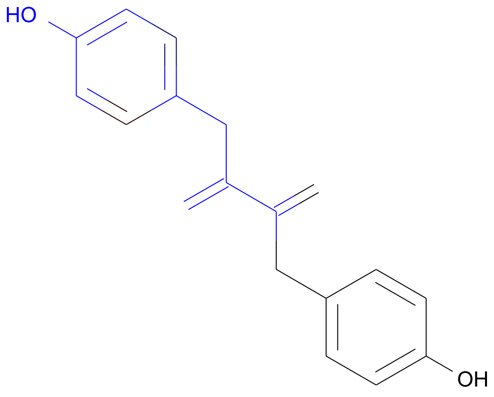

$C_{18}H_{18}O_2$
Molecular Weight: 266.33432
ALogP: 4.8
Rotatable Bonds: 5
Acceptors: 2
Donors: 2

Model Prediction

Prediction: 260
Unit: mg/kg_body_weight/day
Mahalanobis Distance: 12
Mahalanobis Distance p-value: 0.000551

Mahalanobis Distance: The Mahalanobis distance (MD) is a generalization of the Euclidean distance that accounts for correlations among the X properties. It is calculated as the distance to the center of the training data. The larger the MD, the less trustworthy the prediction.
Mahalanobis Distance p-value: The p-value gives the fraction of training data with an MD greater than or equal to the one for the given sample, assuming normally distributed data. The smaller the p-value, the less trustworthy the prediciton. For highly non-normal X properties (e.g., fingerprints), the MD p-value is wildly inaccurate.

Structural Similar Compounds

Name	5	Diethylstilbestrol	1-(4-Chlorophenyl)-1-phenyl-2-propynyl carbamate
Structure	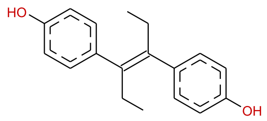	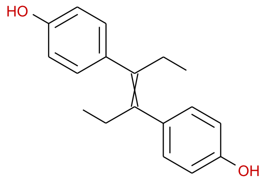	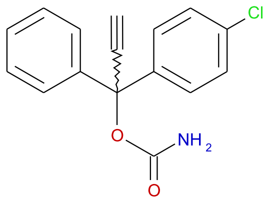
Actual Endpoint (-log C)	6.0804	6.0804	4.51245
Predicted Endpoint (-log C)	3.53032	3.53032	3.49372
Distance	0.354	0.354	0.591
Reference	CPDB	CPDB	CPDB

Model Applicability

Unknown features are fingerprint features in the query molecule, but not found or appearing too infrequently in the training set.

1. All properties and OPS components are within expected ranges.

Feature Contribution

Top features for positive contribution			
**Fingerprint**	**Bit/Smiles**	**Feature Structure**	**Score**
FCFP_6	203677720	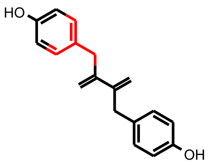 [*]C([*])[c](:[cH]:[*]):[c](:[*]):[*]	0.137

FCFP_6	129344189	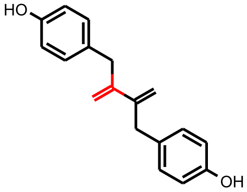 [*]C=C	0.112
**Top Features for negative contribution**			
**Fingerprint**	**Bit/Smiles**	**Feature Structure**	**Score**
FCFP_6	436886043	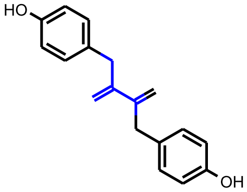 [*]C=C(C)C	-0.383
FCFP_6	7	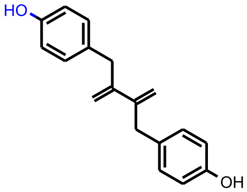 [*]O	-0.372
FCFP_6	16	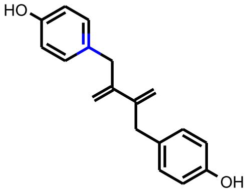 [*]:[cH]:[*]	-0.354

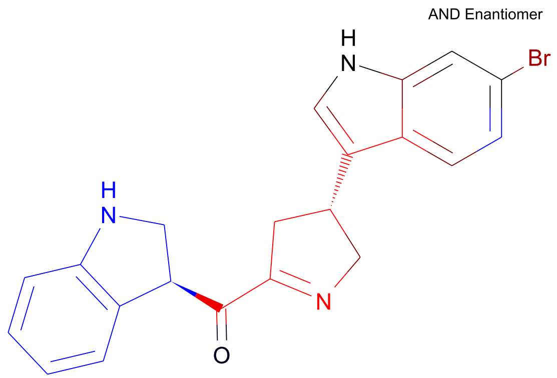

$C_{21}H_{18}BrN_3O$
Molecular Weight: 408.29112
ALogP: 3.919
Rotatable Bonds: 3
Acceptors: 3
Donors: 2

Model Prediction

Prediction: 3.2
Unit: mg/kg_body_weight/day
Mahalanobis Distance: 15.2
Mahalanobis Distance p-value: 8.19e-012

Mahalanobis Distance: The Mahalanobis distance (MD) is a generalization of the Euclidean distance that accounts for correlations among the X properties. It is calculated as the distance to the center of the training data. The larger the MD, the less trustworthy the prediction.
Mahalanobis Distance p-value: The p-value gives the fraction of training data with an MD greater than or equal to the one for the given sample, assuming normally distributed data. The smaller the p-value, the less trustworthy the prediciton. For highly non-normal X properties (e.g., fingerprints), the MD p-value is wildly inaccurate.

Structural Similar Compounds

Name	Phenolphthalein	Indomethacin	3-(Cyclopentyloxy)-N-(3,5-di-chloro-4-pyridyl)-4-methoxy-benzamide
Structure	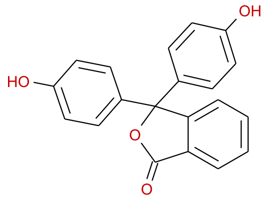	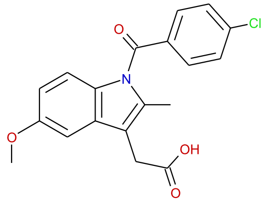	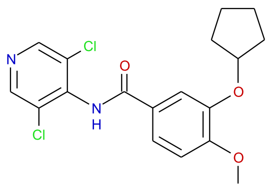
Actual Endpoint (-log C)	2.54766	5.49293	5.39369
Predicted Endpoint (-log C)	3.7508	4.9569	4.27874
Distance	0.618	0.703	0.724
Reference	CPDB	CPDB	CPDB

Model Applicability

Unknown features are fingerprint features in the query molecule, but not found or appearing too infrequently in the training set.

1. All properties and OPS components are within expected ranges.

Feature Contribution

Top features for positive contribution

Fingerprint	Bit/Smiles	Feature Structure	Score
FCFP_6	565968762	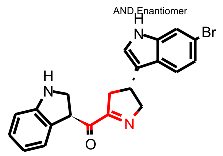 [*]C(=[*])C1=N[*][*]C 1	0.266

FCFP_6	1	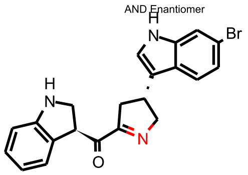 [*]O[*]	0.234
FCFP_6	32	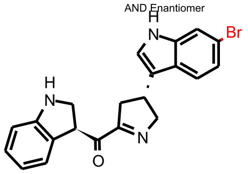 [*]Br	0.154
**Top Features for negative contribution**			
**Fingerprint**	**Bit/Smiles**	**Feature Structure**	**Score**
FCFP_6	-1272709286	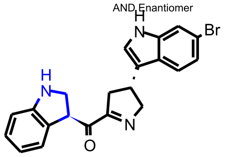 [*][C@H]1[*]:[*]NC1	-0.526
FCFP_6	991735244	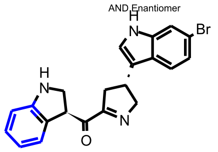 [*]:[c]1:[*]:[cH]:[cH]:[cH]:[cH]:1	-0.422
FCFP_6	16	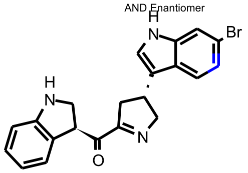 [*]:[cH]:[*]	-0.354

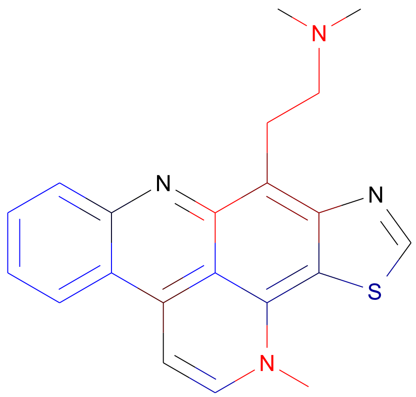

$C_{21}H_{20}N_4S$
Molecular Weight: 360.4753
ALogP: 3.682
Rotatable Bonds: 3
Acceptors: 4
Donors: 0

Structural Similar Compounds

Name	357	4-(4-N-Methyl-N-nitrosamino-styryl)quinoline	1-Nitropyrene
Structure	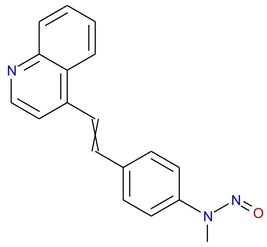	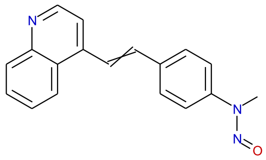	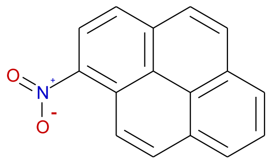
Actual Endpoint (-log C)	5.61692	5.61692	4.87069
Predicted Endpoint (-log C)	5.49128	5.49128	5.08823
Distance	0.653	0.653	0.703
Reference	CPDB	CPDB	CPDB

Model Prediction

Prediction: 0.282
Unit: mg/kg_body_weight/day
Mahalanobis Distance: 15.5
Mahalanobis Distance p-value: 8.06e-013

Mahalanobis Distance: The Mahalanobis distance (MD) is a generalization of the Euclidean distance that accounts for correlations among the X properties. It is calculated as the distance to the center of the training data. The larger the MD, the less trustworthy the prediction.
Mahalanobis Distance p-value: The p-value gives the fraction of training data with an MD greater than or equal to the one for the given sample, assuming normally distributed data. The smaller the p-value, the less trustworthy the prediciton. For highly non-normal X properties (e.g., fingerprints), the MD p-value is wildly inaccurate.

Model Applicability

Unknown features are fingerprint features in the query molecule, but not found or appearing too infrequently in the training set.

1. OPS PC25 out of range. Value: 4.6913. Training min, max, SD, explained variance: -3.5991, 4.3957, 1.055, 0.0107.

Feature Contribution

Top features for positive contribution

Fingerprint	Bit/Smiles	Feature Structure	Score
FCFP_6	136627117	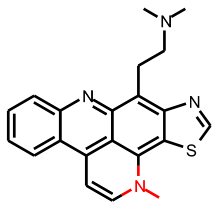 [*]OC	0.69

FCFP_6	9	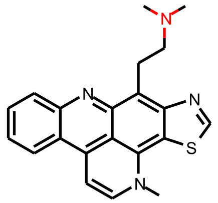 [*]N([*])[*]	0.385
FCFP_6	-587569116	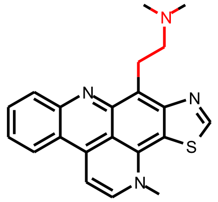 [*]N1[*][*]CC1	0.319

Top Features for negative contribution

Fingerprint	Bit/Smiles	Feature Structure	Score
FCFP_6	991735244	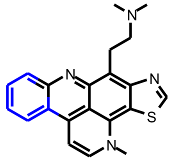 [*]:[c]1:[*]:[cH]:[cH]:[cH]:[cH]:1	-0.422
FCFP_6	16	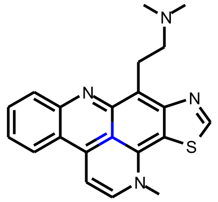 [*]:[cH]:[*]	-0.354
FCFP_6	675769755	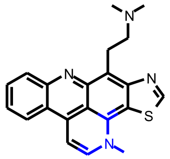 [*]=CN(C)[c](:[*]):[*]	-0.172

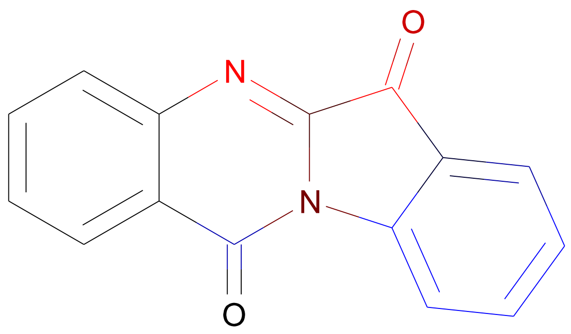

$C_{15}H_8N_2O_2$
Molecular Weight: 248.23621
ALogP: 2.331
Rotatable Bonds: 0
Acceptors: 3
Donors: 0

Structural Similar Compounds

Name	N-1-Diacetamidofluorene	8-Methoxypsoralen	N-Hydroxy-2-acetylamino-fluorene s
Structure	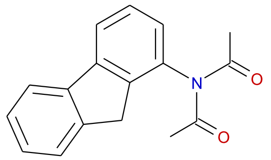	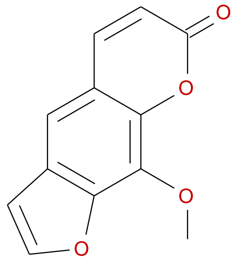	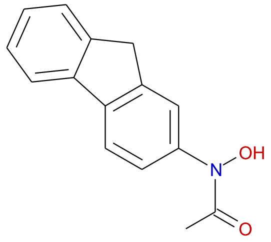
Actual Endpoint (-log C)	4.14499	3.82429	5.38413
Predicted Endpoint (-log C)	4.21549	4.90875	3.97008
Distance	0.511	0.569	0.585
Reference	CPDB	CPDB	CPDB

Model Prediction

Prediction: 9.36
Unit: mg/kg_body_weight/day
Mahalanobis Distance: 11.1
Mahalanobis Distance p-value: 0.0157

Mahalanobis Distance: The Mahalanobis distance (MD) is a generalization of the Euclidean distance that accounts for correlations among the X properties. It is calculated as the distance to the center of the training data. The larger the MD, the less trustworthy the prediction.
Mahalanobis Distance p-value: The p-value gives the fraction of training data with an MD greater than or equal to the one for the given sample, assuming normally distributed data. The smaller the p-value, the less trustworthy the prediciton. For highly non-normal X properties (e.g., fingerprints), the MD p-value is wildly inaccurate.

Model Applicability

Unknown features are fingerprint features in the query molecule, but not found or appearing too infrequently in the training set.

1. All properties and OPS components are within expected ranges.

Feature Contribution

Top features for positive contribution			
Fingerprint	Bit/Smiles	Feature Structure	Score
FCFP_6	565998553	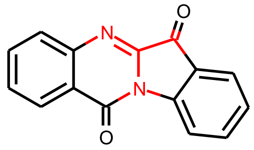 [*][C@@H]1[*][*]OC1=O	0.357

FCFP_6	-2090462286	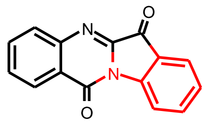<br>[*]N1[*][*][c]2:[cH]:<br>[cH]:[cH]:[cH]:[c]1:<br>2	0.245
FCFP_6	1	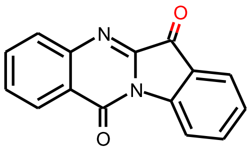<br>[*]O[*]	0.234

Top Features for negative contribution

Fingerprint	Bit/Smiles	Feature Structure	Score
FCFP_6	991735244	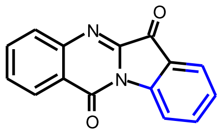<br>[*]:[c]1:[*]:[cH]:[cH<br>]:[cH]:[cH]:1	-0.422
FCFP_6	16	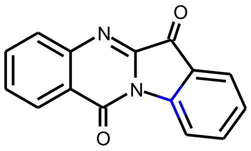<br>[*]:[cH]:[*]	-0.354
FCFP_6	1674451008	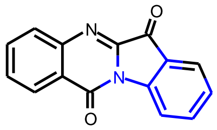<br>[*]O[c]1:[cH]:[*]:[c]<br>(:[*]):[cH]:[cH]:1	-0.233

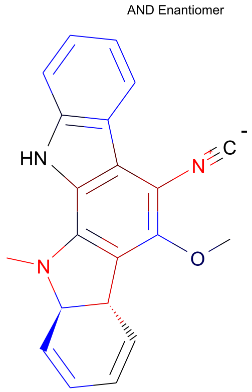

$C_{21}H_{17}N_3O$
Molecular Weight: 327.37918
ALogP: 4.078
Rotatable Bonds: 1
Acceptors: 2
Donors: 1

Structural Similar Compounds

Name	Phenolphthalein	Acronycine	1-Nitropyrene
Structure	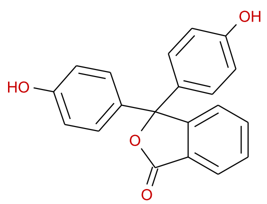	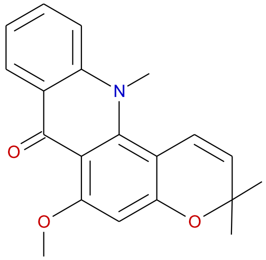	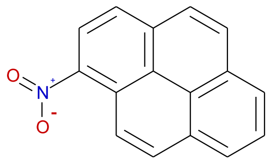
Actual Endpoint (-log C)	2.54766	5.80371	4.87069
Predicted Endpoint (-log C)	3.7508	4.88757	5.08823
Distance	0.672	0.680	0.693
Reference	CPDB	CPDB	CPDB

Model Prediction

Prediction: 0.947
Unit: mg/kg_body_weight/day
Mahalanobis Distance: 16.4
Mahalanobis Distance p-value: 1.91e-015

Mahalanobis Distance: The Mahalanobis distance (MD) is a generalization of the Euclidean distance that accounts for correlations among the X properties. It is calculated as the distance to the center of the training data. The larger the MD, the less trustworthy the prediction.
Mahalanobis Distance p-value: The p-value gives the fraction of training data with an MD greater than or equal to the one for the given sample, assuming normally distributed data. The smaller the p-value, the less trustworthy the prediciton. For highly non-normal X properties (e.g., fingerprints), the MD p-value is wildly inaccurate.

Model Applicability

Unknown features are fingerprint features in the query molecule, but not found or appearing too infrequently in the training set.

1. All properties and OPS components are within expected ranges.
2. Unknown FCFP_2 feature: 1934974835: [*]:[c](:[*])[N+]#[C-]
3. Unknown FCFP_2 feature: -1487147388: [*][N+]#[C-]

Feature Contribution

Top features for positive contribution

Fingerprint	Bit/Smiles	Feature Structure	Score
FCFP_6	136627117	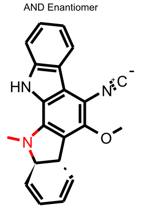 [*]OC	0.69

FCFP_6	8	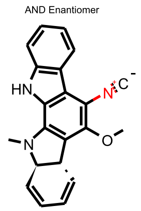<br>[*][N+]#[*]	0.336
FCFP_6	1	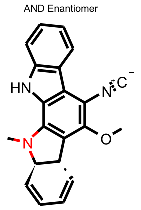<br>[*]O[*]	0.234
**Top Features for negative contribution**			
**Fingerprint**	**Bit/Smiles**	**Feature Structure**	**Score**
FCFP_6	451847724	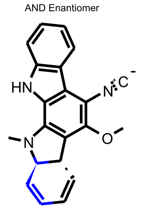<br>[*]CC=C([*])[*]	-0.436
FCFP_6	991735244	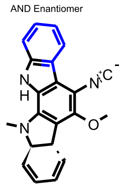<br>[*]:[c]1:[*]:[cH]:[cH<br>]:[cH]:[cH]:1	-0.422
FCFP_6	16	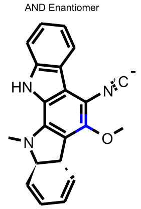<br>[*]:[cH]:[*]	-0.354

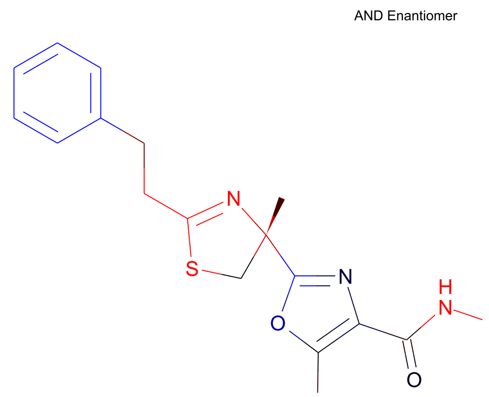

$C_{18}H_{21}N_3O_2S$
Molecular Weight: 343.44324
ALogP: 2.716
Rotatable Bonds: 5
Acceptors: 4
Donors: 1

Model Prediction

Prediction: 22.7
Unit: mg/kg_body_weight/day
Mahalanobis Distance: 13.8
Mahalanobis Distance p-value: 8.85e-008

Mahalanobis Distance: The Mahalanobis distance (MD) is a generalization of the Euclidean distance that accounts for correlations among the X properties. It is calculated as the distance to the center of the training data. The larger the MD, the less trustworthy the prediction.
Mahalanobis Distance p-value: The p-value gives the fraction of training data with an MD greater than or equal to the one for the given sample, assuming normally distributed data. The smaller the p-value, the less trustworthy the prediciton. For highly non-normal X properties (e.g., fingerprints), the MD p-value is wildly inaccurate.

Structural Similar Compounds

Name	3-(Cyclopentyloxy)-N-(3,5-di-chloro-4-pyridyl)-4-methoxy-benzamide	C.I. pigment red 3	Omeprazole
Structure	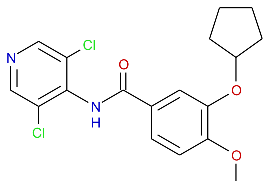	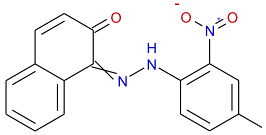	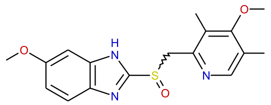
Actual Endpoint (-log C)	5.39369	2.41938	3.4628
Predicted Endpoint (-log C)	4.27874	4.26375	4.7324
Distance	0.588	0.589	0.592
Reference	CPDB	CPDB	CPDB

Model Applicability

Unknown features are fingerprint features in the query molecule, but not found or appearing too infrequently in the training set.

1. OPS PC20 out of range. Value: -4.1427. Training min, max, SD, explained variance: -3.9266, 5.5565, 1.236, 0.0147.
2. Unknown FCFP_2 feature: -836603894: [*]:[c](:[*])[C@]1(C)C[*][*]=N1

Feature Contribution

Top features for positive contribution			
Fingerprint	Bit/Smiles	Feature Structure	Score
FCFP_6	565998553	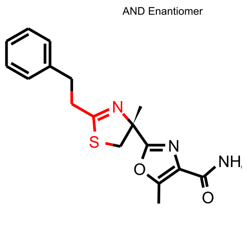 [*][C@@H]1[*][*]OC1=O	0.357

FCFP_6	1	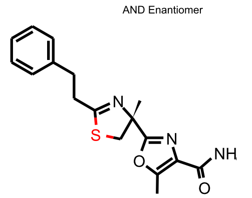 [*]O[*]	0.234
FCFP_6	-885550502	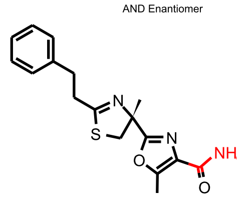 [*]C(=[*])NC	0.229

Top Features for negative contribution

Fingerprint	Bit/Smiles	Feature Structure	Score
FCFP_6	991735244	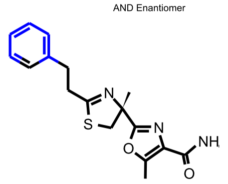 [*]:[c]1:[*]:[cH]:[cH]:[cH]:[cH]:1	-0.422
FCFP_6	-2093839777	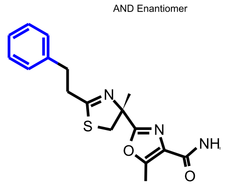 [*][c]1:[cH]:[cH]:[cH]:[cH]:[cH]:1	-0.378
FCFP_6	16	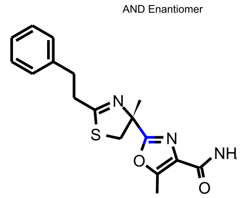 [*]:[cH]:[*]	-0.354

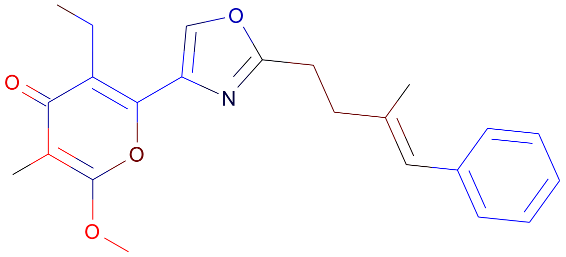

$C_{23}H_{25}NO_4$
Molecular Weight: 379.4489
ALogP: 5.22
Rotatable Bonds: 7
Acceptors: 4
Donors: 0

Model Prediction

Prediction: 31.3
Unit: mg/kg_body_weight/day
Mahalanobis Distance: 15
Mahalanobis Distance p-value: 2.54e-011

Mahalanobis Distance: The Mahalanobis distance (MD) is a generalization of the Euclidean distance that accounts for correlations among the X properties. It is calculated as the distance to the center of the training data. The larger the MD, the less trustworthy the prediction.
Mahalanobis Distance p-value: The p-value gives the fraction of training data with an MD greater than or equal to the one for the given sample, assuming normally distributed data. The smaller the p-value, the less trustworthy the prediciton. For highly non-normal X properties (e.g., fingerprints), the MD p-value is wildly inaccurate.

Structural Similar Compounds

Name	Butyl benzyl phthalate	3-(Cyclopentyloxy)-N-(3,5-di-chloro-4-pyridyl)-4-methoxy-benzamide	FD & C violet no. 1
Structure	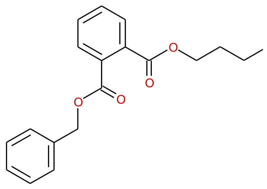	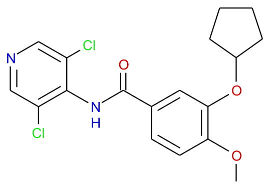	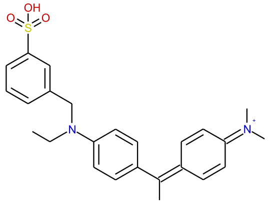
Actual Endpoint (-log C)	2.47762	5.39369	2.8543
Predicted Endpoint (-log C)	3.20177	4.27874	3.40838
Distance	0.603	0.608	0.621
Reference	CPDB	CPDB	CPDB

Model Applicability

Unknown features are fingerprint features in the query molecule, but not found or appearing too infrequently in the training set.

1. All properties and OPS components are within expected ranges.
2. Unknown FCFP_2 feature: -2115241127: [*]OC(=C([*])[*])O[*]

Feature Contribution

Top features for positive contribution			
Fingerprint	Bit/Smiles	Feature Structure	Score
FCFP_6	136627117	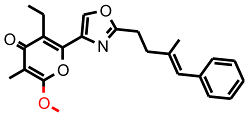 [*]OC	0.69

FCFP_6	565968762	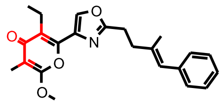 [*]C(=[*])C1=N[*][*]C1	0.266
FCFP_6	1	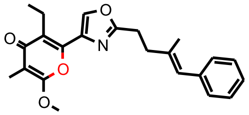 [*]O[*]	0.234

Top Features for negative contribution

Fingerprint	Bit/Smiles	Feature Structure	Score
FCFP_6	991735244	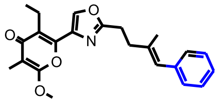 [*]:[c]1:[*]:[cH]:[cH]:[cH]:[cH]:1	-0.422
FCFP_6	436886043	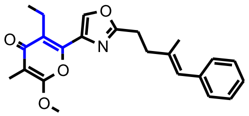 [*]C=C(C)C	-0.383
FCFP_6	-2093839777	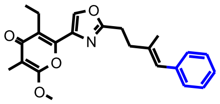 [*][c]1:[cH]:[cH]:[cH]:[cH]:[cH]:1	-0.378

Remdesivir

TOPKAT_Carcinogenic_Potency_TD50_Rat

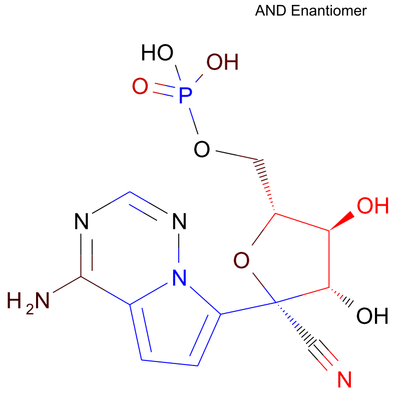

$C_{12}H_{14}N_5O_7P$

Molecular Weight: 371.24262

ALogP: -1.502

Rotatable Bonds: 4

Acceptors: 11

Donors: 5

Model Prediction

Prediction: 1.01

Unit: mg/kg_body_weight/day

Mahalanobis Distance: 16.2

Mahalanobis Distance p-value: 4.38e-015

Mahalanobis Distance: The Mahalanobis distance (MD) is a generalization of the Euclidean distance that accounts for correlations among the X properties. It is calculated as the distance to the center of the training data. The larger the MD, the less trustworthy the prediction.
Mahalanobis Distance p-value: The p-value gives the fraction of training data with an MD greater than or equal to the one for the given sample, assuming normally distributed data. The smaller the p-value, the less trustworthy the prediciton. For highly non-normal X properties (e.g., fingerprints), the MD p-value is wildly inaccurate.

Structural Similar Compounds

Name	b-Thioguanine deoxyriboside	Hexamethylmelamine	604
Structure	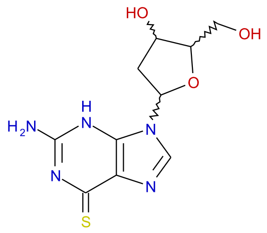	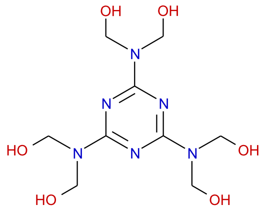	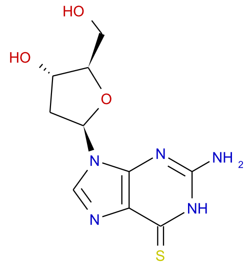
Actual Endpoint (-log C)	5.13004	4.47751	5.13004
Predicted Endpoint (-log C)	4.82552	3.76275	4.96687
Distance	0.805	0.832	0.835
Reference	CPDB	CPDB	CPDB

Model Applicability

Unknown features are fingerprint features in the query molecule, but not found or appearing too infrequently in the training set.

1. All properties and OPS components are within expected ranges.
2. Unknown FCFP_2 feature: 472180098: [*]OP(=O)(O)O
3. Unknown FCFP_2 feature: -836603894: [*]:[c](:[*])[C@]1(C)C[*][*]=N1

Feature Contribution

Top features for positive contribution

Fingerprint	Bit/Smiles	Feature Structure	Score
FCFP_6	-1043250487	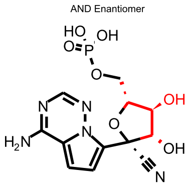 [*][C@@H]1[*][*][C@H]([*])C1O	1.15

FCFP_6	9	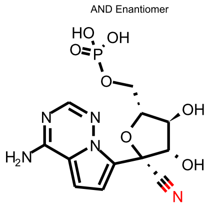 [*]N([*])[*]	0.385
FCFP_6	1	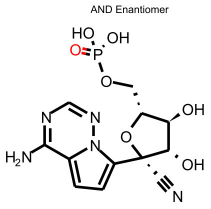 [*]O[*]	0.234

Top Features for negative contribution

Fingerprint	Bit/Smiles	Feature Structure	Score
FCFP_6	-1280036918	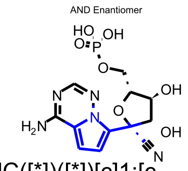 [*]C([*])([*])[c]1:[cH]:[cH]:[c](:[*]):n:1:[*]	-0.363
FCFP_6	16	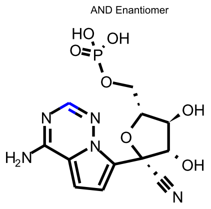 [*]:[cH]:[*]	-0.354
FCFP_6	17	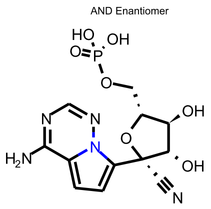 [*]:n:[*]	-0.149

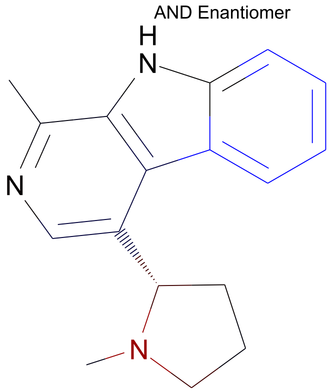

$C_{17}H_{19}N_3$
Molecular Weight: 265.35286
ALogP: 3.018
Rotatable Bonds: 1
Acceptors: 2
Donors: 1

Model Prediction

Prediction: 0.0475
Unit: g/kg_body_weight
Mahalanobis Distance: 33
Mahalanobis Distance p-value: 4.56e-030

Mahalanobis Distance: The Mahalanobis distance (MD) is a generalization of the Euclidean distance that accounts for correlations among the X properties. It is calculated as the distance to the center of the training data. The larger the MD, the less trustworthy the prediction.
Mahalanobis Distance p-value: The p-value gives the fraction of training data with an MD greater than or equal to the one for the given sample, assuming normally distributed data. The smaller the p-value, the less trustworthy the prediciton. For highly non-normal X properties (e.g., fingerprints), the MD p-value is wildly inaccurate.

Structural Similar Compounds

Name	3-AMINO-9-ETHYLCARBAZOLE.HCL	ESTAZOLAM	MIDAZOLAM.HCL
Structure	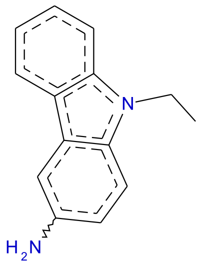	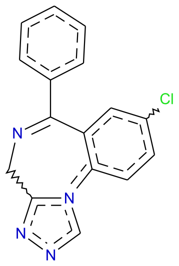	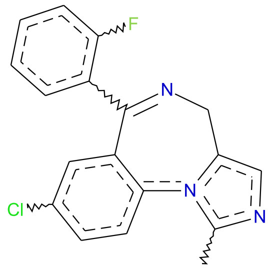
Actual Endpoint (-log C)	3.72074	3.99232	4.55867
Predicted Endpoint (-log C)	4.28782	3.88059	3.94765
Distance	0.528	0.559	0.560
Reference	NTP 93 VII	NDA-19080	NDA-18654

Model Applicability

Unknown features are fingerprint features in the query molecule, but not found or appearing too infrequently in the training set.

1. OPS PC8 out of range. Value: -6.8506. Training min, max, SD, explained variance: -5.7428, 7.3359, 2.68, 0.0314.
2. Unknown ECFP_6 feature: -152683720: [*]:[nH]:[*]
3. Unknown ECFP_6 feature: 1997021792: [*]:[cH]:[cH]:[cH]:[*]
4. Unknown ECFP_6 feature: 1099224616: [*]:[cH]:[c]1:[nH]:[*]:[*]:[c]:1:[*]
5. Unknown ECFP_6 feature: 1333660716: [*]:[cH]:[c]1:[c](:[*]):[*]:[*]:[c]:1:[*]
6. Unknown ECFP_6 feature: -1661653144: [*][c](:[*]):[c]1:[c](:[*]):[*]:[*]:[c]:1:[*]
7. Unknown ECFP_6 feature: 978230116: [*][c](:[*]):[c]1:[nH]:[*]:[*]:[c]:1:[*]
8. Unknown ECFP_6 feature: 558201926: [*]:[c]1:[*]:[*]:[c](:[*]):[nH]:1
9. Unknown ECFP_6 feature: 1652635785: [*]:n:[c](C):[c](:[*]):[*]
10. Unknown ECFP_6 feature: -677309799: [*][c](:[*]):n:[cH]:[*]
11. Unknown ECFP_6 feature: -709633021: [*][c](:[*]):[cH]:n:[*]
12. Unknown ECFP_6 feature: 1336304100: [*]C([*])[c](:[cH]:[*]):[c](:[*]):[*]
13. Unknown ECFP_6 feature: -484970154: [*]N1[*][*]C[C@H]1[c](:[*]):[*]
14. Unknown ECFP_6 feature: -1332781180: [*]1[*]CCC1
15. Unknown ECFP_6 feature: -757679000: [*]N1[*][*]CC1
16. Unknown ECFP_6 feature: 48510090: [*][C@@H]1[*][*]CN1C
17. Unknown ECFP_6 feature: 866343404: [*]N([*])C

Feature Contribution

Top features for positive contribution			
**Fingerprint**	**Bit/Smiles**	**Feature Structure**	**Score**
ECFP_6	-167460056	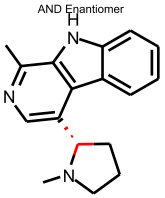<br>[*]C([*])[*]	0.136
FCFP_6	9	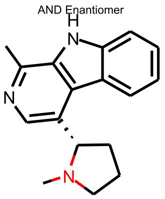<br>[*]N([*])[*]	0.0797
ECFP_6	-992506539	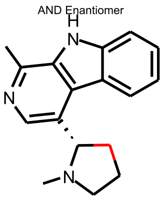<br>[*]C[*]	0.0554

Top Features for negative contribution			
**Fingerprint**	**Bit/Smiles**	**Feature Structure**	**Score**
FCFP_6	991735244	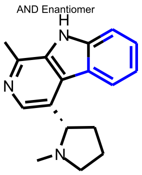<br>[*]:[c]1:[*]:[cH]:[cH<br>]:[cH]:[cH]:1	-0.134

ECFP_6	1564392544	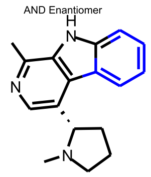 [*]:[c]1:[*]:[cH]:[cH]:[cH]:[cH]:1	-0.133
FCFP_6	203677720	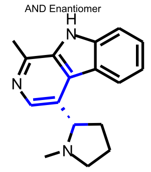 [*]C([*])[c](:[cH]:[*]):[c](:[*]):[*]	-0.0713

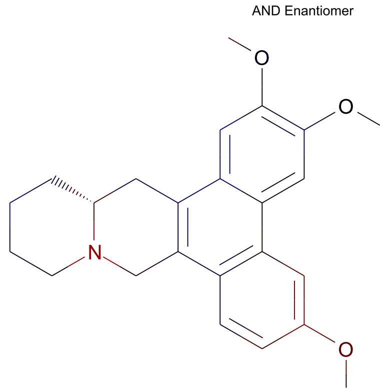

$C_{24}H_{27}NO_3$
Molecular Weight: 377.47608
ALogP: 4.691
Rotatable Bonds: 3
Acceptors: 4
Donors: 0

Model Prediction

Prediction: 0.0103
Unit: g/kg_body_weight
Mahalanobis Distance: 29
Mahalanobis Distance p-value: 7.37e-023

Mahalanobis Distance: The Mahalanobis distance (MD) is a generalization of the Euclidean distance that accounts for correlations among the X properties. It is calculated as the distance to the center of the training data. The larger the MD, the less trustworthy the prediction.
Mahalanobis Distance p-value: The p-value gives the fraction of training data with an MD greater than or equal to the one for the given sample, assuming normally distributed data. The smaller the p-value, the less trustworthy the prediciton. For highly non-normal X properties (e.g., fingerprints), the MD p-value is wildly inaccurate.

Structural Similar Compounds

Name	TRIAZOLAM	QUININE SULFATE	MIDAZOLAM.HCL
Structure	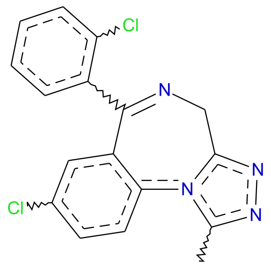	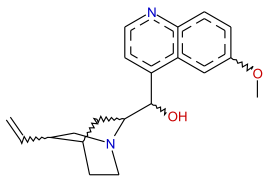	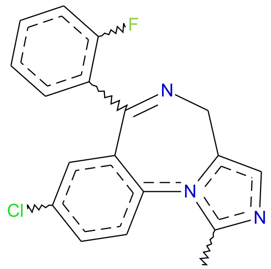
Actual Endpoint (-log C)	3.83659	3.66601	4.55867
Predicted Endpoint (-log C)	3.85527	5.04022	3.94765
Distance	0.617	0.626	0.643
Reference	UPJ-33030	PATHOL.RES.PRAC.163. 373.1978	NDA-18654

Model Applicability

Unknown features are fingerprint features in the query molecule, but not found or appearing too infrequently in the training set.

1. OPS PC18 out of range. Value: -4.8659. Training min, max, SD, explained variance: -4.7991, 6.1674, 1.831, 0.0147.
2. Unknown ECFP_6 feature: 1307307440: [*]:[c](:[*])OC
3. Unknown ECFP_6 feature: -1103661462: [*]CN(C[*])C([*])[*]
4. Unknown ECFP_6 feature: -1869628272: [*]CC(C[*])N([*])[*]
5. Unknown ECFP_6 feature: 53207596: [*]C([*])C[c](:[*]):[*]
6. Unknown ECFP_6 feature: -757679000: [*]N1[*][*]CC1
7. Unknown ECFP_6 feature: -762035154: [*]N([*])C[c](:[*]):[*]
8. Unknown ECFP_6 feature: -1332781180: [*]1[*]CCC1
9. Unknown ECFP_6 feature: -1660340418: [*]C[c](:[c]([*]):[*]):[c](:[*]):[*]
10. Unknown ECFP_6 feature: 1333660716: [*]:[cH]:[c]1:[c](:[*]):[*]:[*]:[c]:1:[*]

Feature Contribution

Top features for positive contribution			
**Fingerprint**	**Bit/Smiles**	**Feature Structure**	**Score**

ECFP_6	-167460056	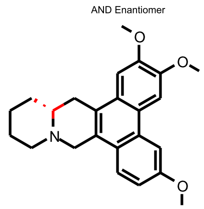 [*]C([*])[*]	0.136
ECFP_6	-176455838	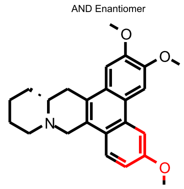 [*]O[c](:[cH]:[*]):[c H]:[*]	0.106
FCFP_6	9	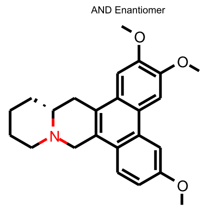 [*]N([*])[*]	0.0797

Top Features for negative contribution

**Fingerprint**	**Bit/Smiles**	**Feature Structure**	**Score**
FCFP_6	1	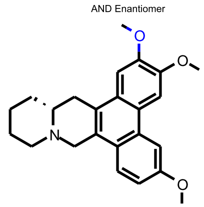 [*]O[*]	-0.102
FCFP_6	203677720	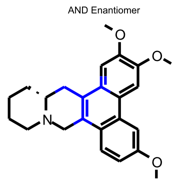 [*]C([*])[c](:[cH]:[*]):[c](:[*]):[*]	-0.0713

FCFP_6	1175638033	AND Enantiomer 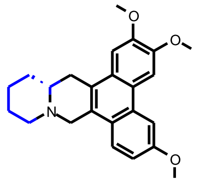 [*][C@@H]1[*]CCCC1	-0.0664

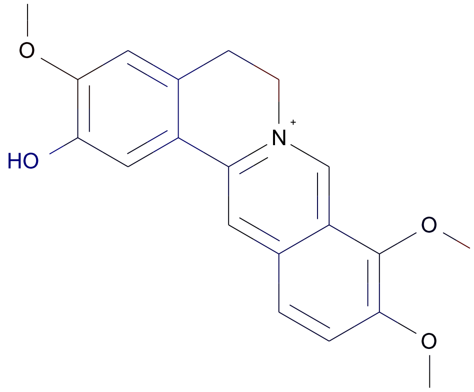

$C_{20}H_{20}NO_4$
Molecular Weight: 338.3771
ALogP: 3.936
Rotatable Bonds: 3
Acceptors: 4
Donors: 1

Model Prediction

Prediction: 0.016
Unit: g/kg_body_weight
Mahalanobis Distance: 27.6
Mahalanobis Distance p-value: 2.57e-020

Mahalanobis Distance: The Mahalanobis distance (MD) is a generalization of the Euclidean distance that accounts for correlations among the X properties. It is calculated as the distance to the center of the training data. The larger the MD, the less trustworthy the prediction.
Mahalanobis Distance p-value: The p-value gives the fraction of training data with an MD greater than or equal to the one for the given sample, assuming normally distributed data. The smaller the p-value, the less trustworthy the prediciton. For highly non-normal X properties (e.g., fingerprints), the MD p-value is wildly inaccurate.

Structural Similar Compounds

Name	QUININE SULFATE	C.I. SOLVENT YELLOW 14	TRIAZOLAM
Structure	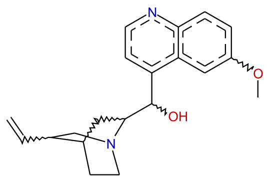	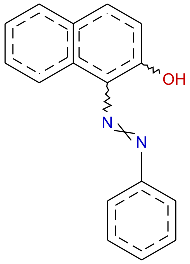	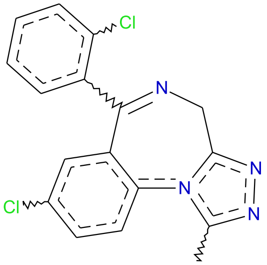
Actual Endpoint (-log C)	3.66601	4.298	3.83659
Predicted Endpoint (-log C)	5.04022	3.36361	3.85527
Distance	0.567	0.600	0.604
Reference	PATHOL.RES.PRAC.163. 373.1978	NTP REPORT # 226	UPJ-33030

Model Applicability

Unknown features are fingerprint features in the query molecule, but not found or appearing too infrequently in the training set.

1. All properties and OPS components are within expected ranges.
2. Unknown FCFP_2 feature: 24: [*][n+](:[*]):[*]
3. Unknown FCFP_2 feature: 414371600: [*]C[n+](:[c]([*]):[*]):c:[*]
4. Unknown FCFP_2 feature: -150573739: [*]CC[n+](:[*]):[*]
5. Unknown FCFP_2 feature: -1861407456: [*][n+](:[*]):[c]([c](:[*]):[*]):c:[*]
6. Unknown FCFP_2 feature: 1618392993: [*][n+](:[*]):c:[c](:[*]):[*]
7. Unknown ECFP_6 feature: -1508366470: [*][n+](:[*]):[*]
8. Unknown ECFP_6 feature: 1307307440: [*]:[c](:[*])OC
9. Unknown ECFP_6 feature: 2019062761: [*]:[c](:[*])O
10. Unknown ECFP_6 feature: 1508268466: [*]C[n+](:[cH]:[*]):[c]([*]):[*]
11. Unknown ECFP_6 feature: -1333923932: [*]CC[n+](:[*]):[*]
12. Unknown ECFP_6 feature: 51876938: [*]CC[c](:[*]):[*]
13. Unknown ECFP_6 feature: 2078070048: [*][n+](:[*]):[c](:[cH]:[*])[c](:[*]):[*]
14. Unknown ECFP_6 feature: 1334973442: [*]C[c](:[cH]:[*]):[c]([*]):[*]
15. Unknown ECFP_6 feature: 688916016: [*][n+](:[*]):[cH]:[c](:[*]):[*]
16. Unknown ECFP_6 feature: 1333660716: [*]:[cH]:[c]1:[c](:[*]):[*]:[*]:[c]:1:[*]
17. Unknown ECFP_6 feature: -178525456: [*]:[cH]:[c](:[cH]:[*]):[c](:[*]):[*]
18. Unknown ECFP_6 feature: -1531301414: [*]O[c](:[c]([*]):[*]):[c](:[*]):[*]

Feature Contribution

Top features for positive contribution			
**Fingerprint**	**Bit/Smiles**	**Feature Structure**	**Score**
FCFP_6	1036089772	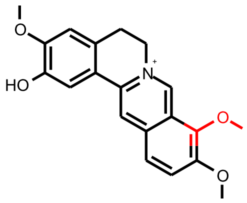 [*]:[c](:[*])OC	0.073
ECFP_6	-992506539	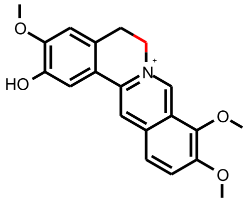 [*]C[*]	0.0554
FCFP_6	136627117	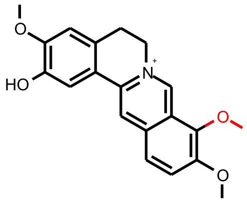 [*]OC	0.0538

Top Features for negative contribution			
**Fingerprint**	**Bit/Smiles**	**Feature Structure**	**Score**
FCFP_6	1	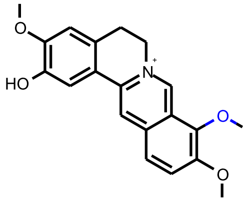 [*]O[*]	-0.102

FCFP_6	203677720	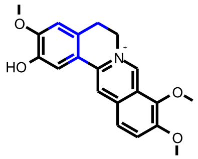 [*]C([*])[c](:[cH]:[*]):[c](:[*]):[*]	-0.0713
FCFP_6	7	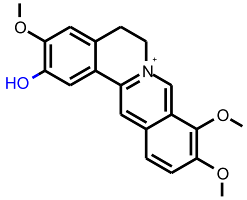 [*]O	-0.0664

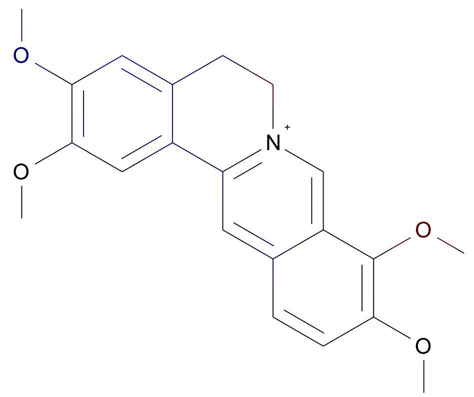

$C_{21}H_{22}NO_4$
Molecular Weight: 352.40368
ALogP: 4.161
Rotatable Bonds: 4
Acceptors: 4
Donors: 0

Structural Similar Compounds

Name	TRIAZOLAM	QUININE SULFATE	ROTENONE
Structure	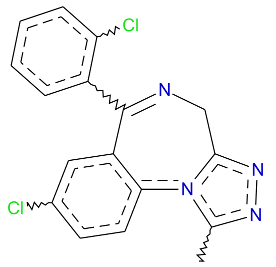	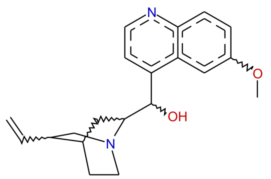	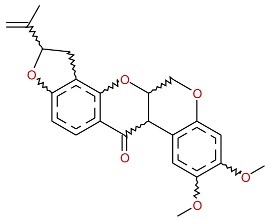
Actual Endpoint (-log C)	3.83659	3.66601	5.0219
Predicted Endpoint (-log C)	3.85527	5.04022	4.24871
Distance	0.595	0.611	0.630
Reference	UPJ-33030	PATHOL.RES.PRAC.163. 373.1978	NTP REPORT # 320

Model Prediction

Prediction: 0.00937
Unit: g/kg_body_weight
Mahalanobis Distance: 24.1
Mahalanobis Distance p-value: 5.67e-014

Mahalanobis Distance: The Mahalanobis distance (MD) is a generalization of the Euclidean distance that accounts for correlations among the X properties. It is calculated as the distance to the center of the training data. The larger the MD, the less trustworthy the prediction.
Mahalanobis Distance p-value: The p-value gives the fraction of training data with an MD greater than or equal to the one for the given sample, assuming normally distributed data. The smaller the p-value, the less trustworthy the prediciton. For highly non-normal X properties (e.g., fingerprints), the MD p-value is wildly inaccurate.

Model Applicability

Unknown features are fingerprint features in the query molecule, but not found or appearing too infrequently in the training set.

1. All properties and OPS components are within expected ranges.
2. Unknown FCFP_2 feature: 24: [*][n+](:[*]):[*]
3. Unknown FCFP_2 feature: 414371600: [*]C[n+](:[c]([*]):[*]):c:[*]
4. Unknown FCFP_2 feature: -150573739: [*]CC[n+](:[*]):[*]
5. Unknown FCFP_2 feature: -1861407456: [*][n+](:[*]):[c]([c](:[*]):[*]):c:[*]
6. Unknown FCFP_2 feature: 1618392993: [*][n+](:[*]):c:[c](:[*]):[*]
7. Unknown ECFP_6 feature: -1508366470: [*][n+](:[*]):[*]
8. Unknown ECFP_6 feature: 1307307440: [*]:[c](:[*])OC
9. Unknown ECFP_6 feature: 1508268466: [*]C[n+](:[cH]:[*]):[c]([*]):[*]
10. Unknown ECFP_6 feature: -1333923932: [*]CC[n+](:[*]):[*]
11. Unknown ECFP_6 feature: 51876938: [*]CC[c](:[*]):[*]
12. Unknown ECFP_6 feature: 2078070048: [*][n+](:[*]):[c](:[cH]:[*])[c](:[*]):[*]
13. Unknown ECFP_6 feature: 1334973442: [*]C[c](:[cH]:[*]):[c]([*]):[*]
14. Unknown ECFP_6 feature: 688916016: [*][n+](:[*]):[cH]:[c](:[*]):[*]
15. Unknown ECFP_6 feature: 1333660716: [*]:[cH]:[c]1:[c](:[*]):[*]:[*]:[c]:1:[*]
16. Unknown ECFP_6 feature: -178525456: [*]:[cH]:[c](:[cH]:[*]):[c](:[*]):[*]
17. Unknown ECFP_6 feature: -1531301414: [*]O[c](:[c]([*]):[*]):[c](:[*]):[*]

Feature Contribution

Top features for positive contribution			
**Fingerprint**	**Bit/Smiles**	**Feature Structure**	**Score**
FCFP_6	1036089772	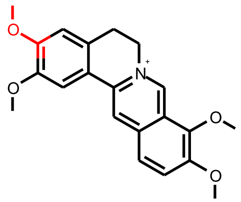 [*]:[c](:[*])OC	0.073
ECFP_6	-992506539	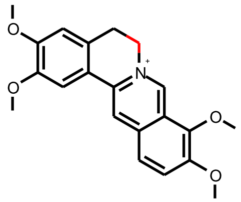 [*]C[*]	0.0554
FCFP_6	136627117	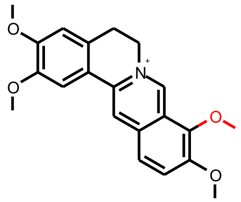 [*]OC	0.0538

Top Features for negative contribution			
**Fingerprint**	**Bit/Smiles**	**Feature Structure**	**Score**
FCFP_6	1	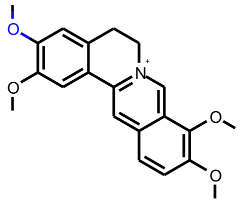 [*]O[*]	-0.102

FCFP_6	203677720	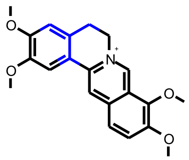<br>[*]C([*])[c](:[cH]:[*<br>]):[c](:[*]):[*]	-0.0713
ECFP_6	1996767644	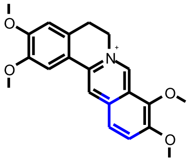<br>[*]:[cH]:[cH]:[c](:[*<br>]):[*]	-0.0497

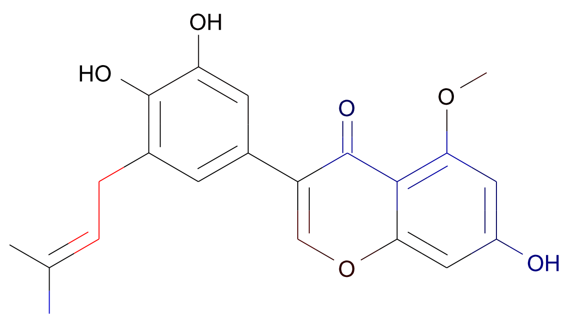

$C_{21}H_{20}O_6$
Molecular Weight: 368.3799
ALogP: 3.98
Rotatable Bonds: 4
Acceptors: 6
Donors: 3

Model Prediction

Prediction: 0.0367
Unit: g/kg_body_weight
Mahalanobis Distance: 28.8
Mahalanobis Distance p-value: 1.56e-022

Mahalanobis Distance: The Mahalanobis distance (MD) is a generalization of the Euclidean distance that accounts for correlations among the X properties. It is calculated as the distance to the center of the training data. The larger the MD, the less trustworthy the prediction.
Mahalanobis Distance p-value: The p-value gives the fraction of training data with an MD greater than or equal to the one for the given sample, assuming normally distributed data. The smaller the p-value, the less trustworthy the prediciton. For highly non-normal X properties (e.g., fingerprints), the MD p-value is wildly inaccurate.

Structural Similar Compounds

Name	ZERANOL	D & C RED 9	SODIUM ACIFLUORFEN
Structure	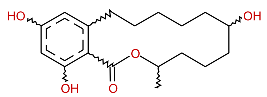	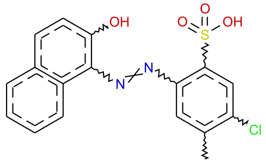	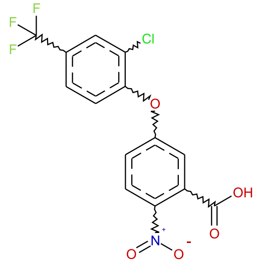
Actual Endpoint (-log C)	4.20737	3.87715	4.16036
Predicted Endpoint (-log C)	3.4948	3.6546	4.65915
Distance	0.669	0.680	0.702
Reference	REGULAT. TOXICOL. PHARMACOL. 1983; 3: 9-	NTP REPORT # 225	EPA COVER SHEET 0192;891101;(1)

Model Applicability

Unknown features are fingerprint features in the query molecule, but not found or appearing too infrequently in the training set.

1. All properties and OPS components are within expected ranges.
2. Unknown ECFP_6 feature: -177786161: [*]:[cH]:[c](O):[cH]:[*]
3. Unknown ECFP_6 feature: -813997308: [*]C(=[*])[c](:[c]([*]):[*]):[c]([*]):[*]
4. Unknown ECFP_6 feature: -570915357: [*]O[c](:[cH]:[*]):[c]([*]):[*]
5. Unknown ECFP_6 feature: 1717462980: [*]C(=[*])C(=O)[c](:[*]):[*]
6. Unknown ECFP_6 feature: 1717082529: [*]\C=C(/C(=[*])[*])\[c](:[*]):[*]
7. Unknown ECFP_6 feature: 471124258: [*]OC=C([*])[*]
8. Unknown ECFP_6 feature: -554736825: [*]=CO[c](:[*]):[*]
9. Unknown ECFP_6 feature: -181568884: [*]C(=[*])[c](:[cH]:[*]):[cH]:[*]
10. Unknown ECFP_6 feature: -2024509555: [*]C[c](:[cH]:[*]):[c]([*]):[*]
11. Unknown ECFP_6 feature: -1660913849: [*][c](:[*]):[c](O):[c]([*]):[*]
12. Unknown ECFP_6 feature: 2019062761: [*]:[c](:[*])O
13. Unknown ECFP_6 feature: 770519970: [*]=CC[c](:[*]):[*]
14. Unknown ECFP_6 feature: -98561723: [*]CC=C([*])[*]
15. Unknown ECFP_6 feature: -1774681326: [*]C=C(C)C
16. Unknown ECFP_6 feature: 1307307440: [*]:[c](:[*])OC

Feature Contribution

Top features for positive contribution

Fingerprint	Bit/Smiles	Feature Structure	Score
FCFP_6	451847724	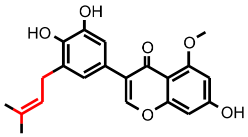 [*]CC=C([*])[*]	0.16
ECFP_6	1559650422	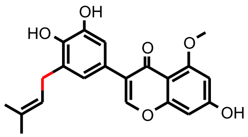 [*]C[*]	0.129
ECFP_6	-1925046727	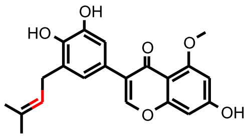 [*]C=[*]	0.0915
**Top Features for negative contribution**			
**Fingerprint**	**Bit/Smiles**	**Feature Structure**	**Score**
ECFP_6	2106656448	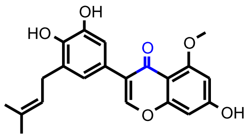 [*]C(=O)[*]	-0.11

FCFP_6	1	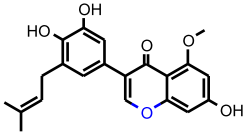<br>[*]O[*]	-0.102
FCFP_6	136597326	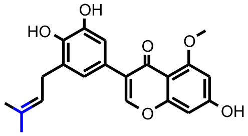<br>[*]C(=[*])C	-0.0815

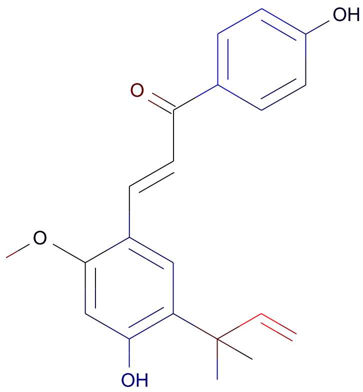

$C_{21}H_{22}O_{4}$
Molecular Weight: 338.39698
ALogP: 4.667
Rotatable Bonds: 6
Acceptors: 4
Donors: 2

Model Prediction

Prediction: 0.026
Unit: g/kg_body_weight
Mahalanobis Distance: 18.5
Mahalanobis Distance p-value: 4.97e-005

Mahalanobis Distance: The Mahalanobis distance (MD) is a generalization of the Euclidean distance that accounts for correlations among the X properties. It is calculated as the distance to the center of the training data. The larger the MD, the less trustworthy the prediction.
Mahalanobis Distance p-value: The p-value gives the fraction of training data with an MD greater than or equal to the one for the given sample, assuming normally distributed data. The smaller the p-value, the less trustworthy the prediciton. For highly non-normal X properties (e.g., fingerprints), the MD p-value is wildly inaccurate.

Structural Similar Compounds

Name	ISOXABEN	ETODOLAC	CHLORBENZILATE
Structure	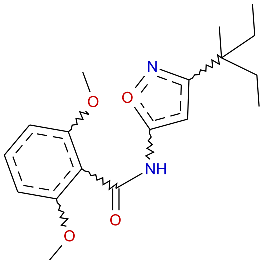	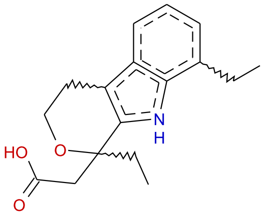	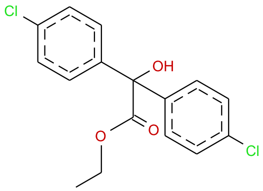
Actual Endpoint (-log C)	3.81665	4.9813	3.609
Predicted Endpoint (-log C)	4.42315	4.39289	4.43906
Distance	0.538	0.587	0.592
Reference	EPA COVER SHEET 0339;881201;(1)	NDA-18922	NTP 75 C-6

Model Applicability

Unknown features are fingerprint features in the query molecule, but not found or appearing too infrequently in the training set.

1. All properties and OPS components are within expected ranges.
2. Unknown ECFP_6 feature: 1544874086: [*]=C
3. Unknown ECFP_6 feature: 2019062761: [*]:[c](:[*])O
4. Unknown ECFP_6 feature: 1307307440: [*]:[c](:[*])OC
5. Unknown ECFP_6 feature: -144557007: [*]=CC(C)(C)[c](:[*]):[*]
6. Unknown ECFP_6 feature: 1336678434: [*][c](:[*]):[c](:[cH]:[*])C([*])([*])[*]
7. Unknown ECFP_6 feature: 865857320: [*]C([*])([*])C
8. Unknown ECFP_6 feature: -1193716553: [*]C([*])([*])C=C
9. Unknown ECFP_6 feature: 1335702447: [*][c](:[*]):[c](C=[*]):[cH]:[*]
10. Unknown ECFP_6 feature: -1831055759: [*]\C=C\[c](:[*]):[*]
11. Unknown ECFP_6 feature: -2090955291: [*]C=C
12. Unknown ECFP_6 feature: -470416293: [*]\C=C\C(=[*])[*]
13. Unknown ECFP_6 feature: 1430764055: [*]=CC(=O)[c](:[*]):[*]
14. Unknown ECFP_6 feature: -175146122: [*]C(=[*])[c](:[cH]:[*]):[cH]:[*]
15. Unknown ECFP_6 feature: -177786161: [*]:[cH]:[c](O):[cH]:[*]

Feature Contribution

Top features for positive contribution

Fingerprint	Bit/Smiles	Feature Structure	Score
FCFP_6	451847724	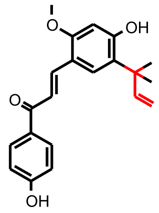 [*]CC=C([*])[*]	0.16
ECFP_6	-1925046727	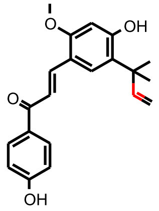 [*]C=[*]	0.0915
ECFP_6	2099970318	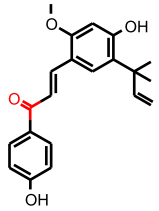 [*]C(=O)[*]	0.0766

Top Features for negative contribution

Fingerprint	Bit/Smiles	Feature Structure	Score
FCFP_6	1	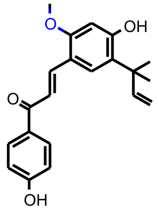 [*]O[*]	-0.102

FCFP_6	-453677277	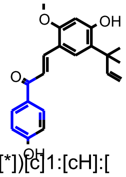<br>[*]C(=[*])[c]1:[cH]:[*]:[c]([*]):[cH]:[cH]:1	-0.0906
FCFP_6	136597326	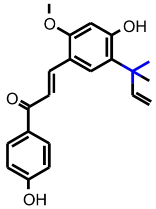<br>[*]C(=[*])C	-0.0815

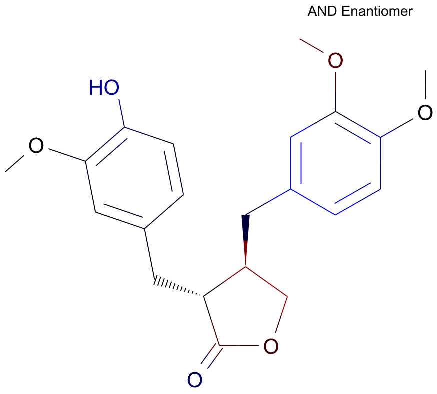

$C_{21}H_{24}O_6$
Molecular Weight: 372.41166
ALogP: 3.743
Rotatable Bonds: 7
Acceptors: 6
Donors: 1

Model Prediction

Prediction: 0.0588
Unit: g/kg_body_weight
Mahalanobis Distance: 28.8
Mahalanobis Distance p-value: 2.18e-022

Mahalanobis Distance: The Mahalanobis distance (MD) is a generalization of the Euclidean distance that accounts for correlations among the X properties. It is calculated as the distance to the center of the training data. The larger the MD, the less trustworthy the prediction.
Mahalanobis Distance p-value: The p-value gives the fraction of training data with an MD greater than or equal to the one for the given sample, assuming normally distributed data. The smaller the p-value, the less trustworthy the prediciton. For highly non-normal X properties (e.g., fingerprints), the MD p-value is wildly inaccurate.

Structural Similar Compounds

Name	ISOXABEN	DILTIAZEM	ROTENONE
Structure	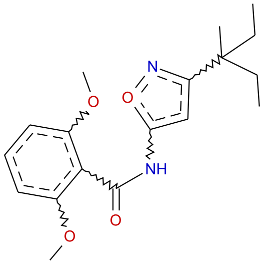	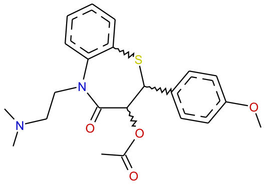	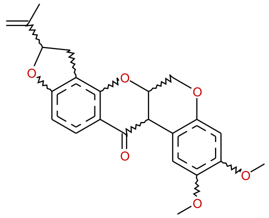
Actual Endpoint (-log C)	3.81665	4.21961	5.0219
Predicted Endpoint (-log C)	4.42315	4.005	4.24871
Distance	0.543	0.544	0.587
Reference	EPA COVER SHEET 0339;881201;(1)	NDA-18602	NTP REPORT # 320

Model Applicability

Unknown features are fingerprint features in the query molecule, but not found or appearing too infrequently in the training set.

1. All properties and OPS components are within expected ranges.
2. Unknown ECFP_6 feature: -556429595: [*]=C1[*][*]CO1
3. Unknown ECFP_6 feature: 1307307440: [*]:[c](:[*])OC
4. Unknown ECFP_6 feature: 2019062761: [*]:[c](:[*])O
5. Unknown ECFP_6 feature: -1794289895: [*]C[C@H]1C[*][*][C@@H]1[*]
6. Unknown ECFP_6 feature: -409631777: [*]C[C@@H]1[C@@H]([*])[*][*]C1=[*]
7. Unknown ECFP_6 feature: -90310073: [*][C@@H]1[*][*]OC1
8. Unknown ECFP_6 feature: 771121623: [*]C([*])C[c](:[*]):[*]
9. Unknown ECFP_6 feature: -1886208901: [*][C@@H]1[*][*]OC1=O

Feature Contribution

Top features for positive contribution			
**Fingerprint**	**Bit/Smiles**	**Feature Structure**	**Score**

ECFP_6	-167460056	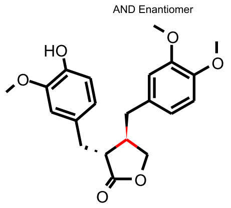 [*]C([*])[*]	0.136
FCFP_6	-1143715940	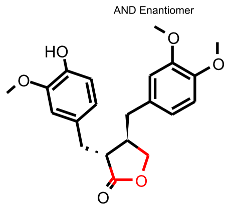 [*]=C1[*][*]CO1	0.13
ECFP_6	1559650422	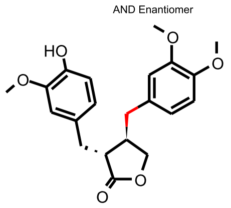 [*]C[*]	0.129
**Top Features for negative contribution**			
**Fingerprint**	**Bit/Smiles**	**Feature Structure**	**Score**
ECFP_6	2106656448	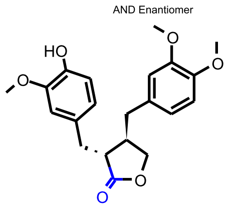 [*]C(=O)[*]	-0.11
FCFP_6	1	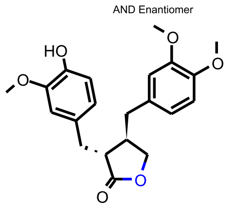 [*]O[*]	-0.102

FCFP_6	-453677277	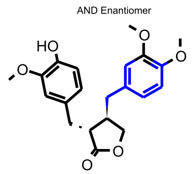 [*]C(=[*])[c]1:[cH]:[*]:[c]([*]):[cH]:[cH]:1	-0.0906

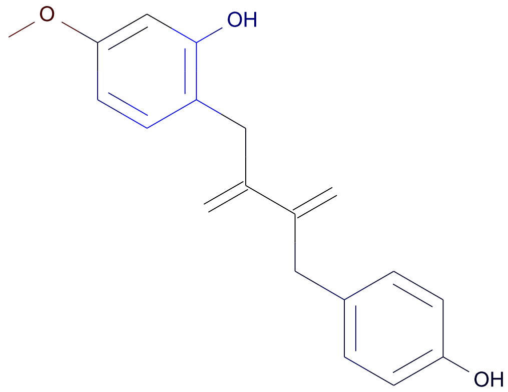

$C_{19}H_{20}O_3$
Molecular Weight: 296.3603
ALogP: 4.784
Rotatable Bonds: 6
Acceptors: 3
Donors: 2

Model Prediction

Prediction: 0.0626
Unit: g/kg_body_weight
Mahalanobis Distance: 22.2
Mahalanobis Distance p-value: 1.35e-010

Mahalanobis Distance: The Mahalanobis distance (MD) is a generalization of the Euclidean distance that accounts for correlations among the X properties. It is calculated as the distance to the center of the training data. The larger the MD, the less trustworthy the prediction.
Mahalanobis Distance p-value: The p-value gives the fraction of training data with an MD greater than or equal to the one for the given sample, assuming normally distributed data. The smaller the p-value, the less trustworthy the prediciton. For highly non-normal X properties (e.g., fingerprints), the MD p-value is wildly inaccurate.

Structural Similar Compounds

Name	DICHLOFENAC.NA	CHLORBENZILATE	ETODOLAC
Structure	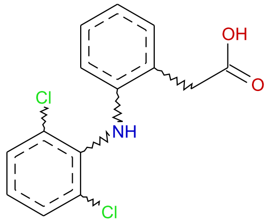	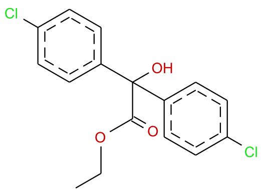	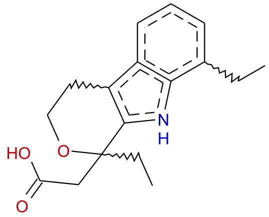
Actual Endpoint (-log C)	5.47151	3.609	4.9813
Predicted Endpoint (-log C)	3.9421	4.43906	4.39289
Distance	0.527	0.551	0.556
Reference	NDA-19201	NTP 75 C-6	NDA-18922

Model Applicability

Unknown features are fingerprint features in the query molecule, but not found or appearing too infrequently in the training set.

1. All properties and OPS components are within expected ranges.
2. Unknown ECFP_6 feature: 1544874086: [*]=C
3. Unknown ECFP_6 feature: 2019062761: [*]:[c](:[*])O
4. Unknown ECFP_6 feature: 1307307440: [*]:[c](:[*])OC
5. Unknown ECFP_6 feature: 771857573: [*]C(=[*])C[c](:[*]):[*]
6. Unknown ECFP_6 feature: -1505409543: [*]CC(=C)C(=[*])[*]
7. Unknown ECFP_6 feature: -2024509555: [*]C[c](:[cH]:[*]):[c]([*]):[*]
8. Unknown ECFP_6 feature: -2092468108: [*]C(=C)[*]
9. Unknown ECFP_6 feature: -177786161: [*]:[cH]:[c](O):[cH]:[*]

Feature Contribution

Top features for positive contribution			
**Fingerprint**	**Bit/Smiles**	**Feature Structure**	**Score**

ECFP_6	1559650422	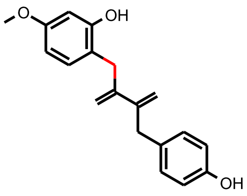<br>[*]C[*]	0.129
ECFP_6	-176455838	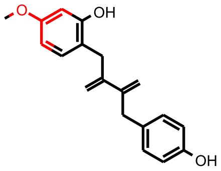<br>[*]O[c](:[cH]:[*]):[c<br>H]:[*]	0.106
FCFP_6	1036089772	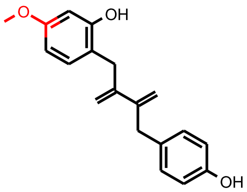<br>[*]:[c](:[*])OC	0.073
**Top Features for negative contribution**			
**Fingerprint**	**Bit/Smiles**	**Feature Structure**	**Score**
FCFP_6	1	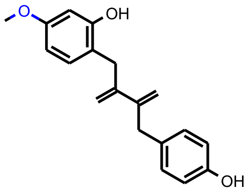<br>[*]O[*]	-0.102
FCFP_6	-453677277	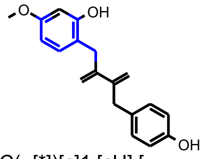<br>[*]C(=[*])[c]1:[cH]:[<br>*]:[c]([*]):[cH]:[cH<br>]:1	-0.0906

FCFP_6	203677720	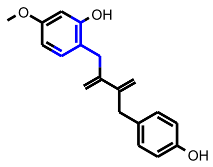 [*]C([*])[c](:[cH]:[*]):[c](:[*]):[*]	-0.0713

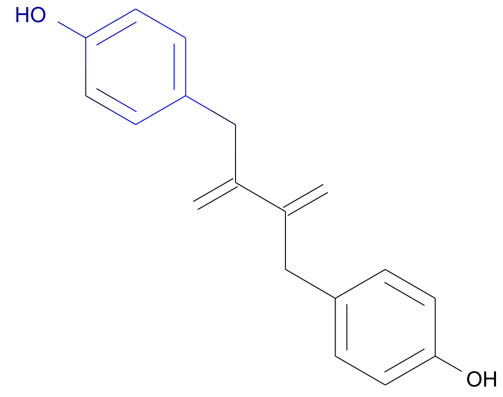

$C_{18}H_{18}O_2$

Molecular Weight: 266.33432

ALogP: 4.8

Rotatable Bonds: 5

Acceptors: 2

Donors: 2

Model Prediction

Prediction: 0.0919

Unit: g/kg_body_weight

Mahalanobis Distance: 17.6

Mahalanobis Distance p-value: 0.000687

Mahalanobis Distance: The Mahalanobis distance (MD) is a generalization of the Euclidean distance that accounts for correlations among the X properties. It is calculated as the distance to the center of the training data. The larger the MD, the less trustworthy the prediction.
Mahalanobis Distance p-value: The p-value gives the fraction of training data with an MD greater than or equal to the one for the given sample, assuming normally distributed data. The smaller the p-value, the less trustworthy the prediciton. For highly non-normal X properties (e.g., fingerprints), the MD p-value is wildly inaccurate.

Structural Similar Compounds

Name	BISPHENOL A	DICHLOFENAC.NA	CHLORBENZILATE
Structure	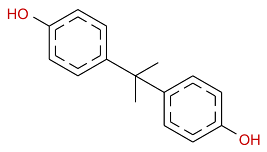	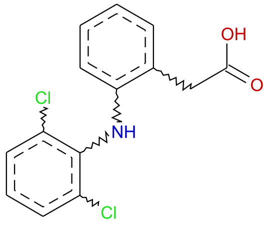	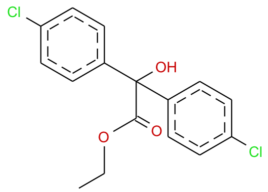
Actual Endpoint (-log C)	3.6595	5.47151	3.609
Predicted Endpoint (-log C)	3.33364	3.9421	4.43906
Distance	0.489	0.511	0.575
Reference	EPA COVER SHEET 0356;890901;(1)	NDA-19201	NTP 75 C-6

Model Applicability

Unknown features are fingerprint features in the query molecule, but not found or appearing too infrequently in the training set.

1. All properties and OPS components are within expected ranges.
2. Unknown ECFP_6 feature: 1544874086: [*]=C
3. Unknown ECFP_6 feature: 2019062761: [*]:[c](:[*])O
4. Unknown ECFP_6 feature: 771857573: [*]C(=[*])C[c](:[*]):[*]
5. Unknown ECFP_6 feature: -1505409543: [*]CC(=C)C(=[*])[*]
6. Unknown ECFP_6 feature: -2092468108: [*]C(=C)[*]
7. Unknown ECFP_6 feature: -177786161: [*]:[cH]:[c](O):[cH]:[*]

Feature Contribution

Top features for positive contribution

Fingerprint	Bit/Smiles	Feature Structure	Score

ECFP_6	1559650422	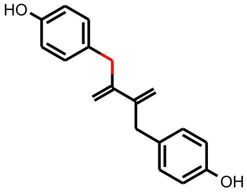<br>[*]C[*]	0.129
ECFP_6	642810091	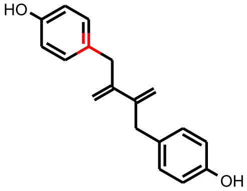<br>[*]:[c](:[*]):[*]	0.0424
FCFP_6	0	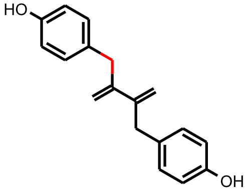<br>[*]C([*])[*]	0.0177
**Top Features for negative contribution**			
**Fingerprint**	**Bit/Smiles**	**Feature Structure**	**Score**
FCFP_6	-453677277	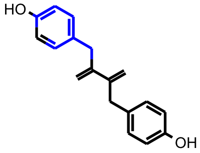<br>[*]C(=[*])[c]1:[cH]:[<br>*]:[c]([*]):[cH]:[cH<br>]:1	-0.0906
FCFP_6	203677720	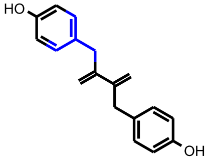<br>[*]C([*])[c](:[cH]:[*<br>]):[c](:[*]):[*]	-0.0713

FCFP_6	7	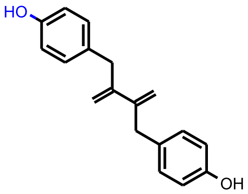<br>[*]O	-0.0664

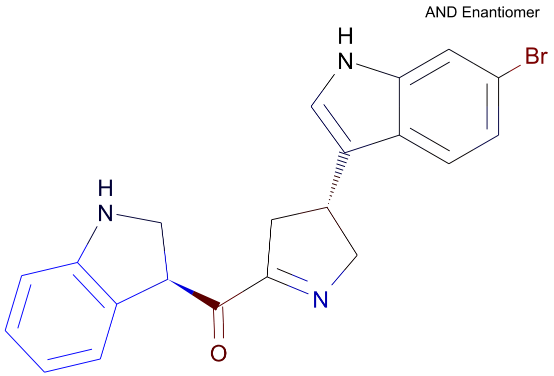

$C_{21}H_{18}BrN_3O$
Molecular Weight: 408.29112
ALogP: 3.919
Rotatable Bonds: 3
Acceptors: 3
Donors: 2

Model Prediction

Prediction: 0.0445
Unit: g/kg_body_weight
Mahalanobis Distance: 36.3
Mahalanobis Distance p-value: 8.02e-036

Mahalanobis Distance: The Mahalanobis distance (MD) is a generalization of the Euclidean distance that accounts for correlations among the X properties. It is calculated as the distance to the center of the training data. The larger the MD, the less trustworthy the prediction.
Mahalanobis Distance p-value: The p-value gives the fraction of training data with an MD greater than or equal to the one for the given sample, assuming normally distributed data. The smaller the p-value, the less trustworthy the prediciton. For highly non-normal X properties (e.g., fingerprints), the MD p-value is wildly inaccurate.

Structural Similar Compounds

Name	LORAZEPAM	ETODOLAC	HALOPERIDOL
Structure	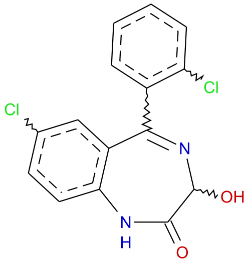	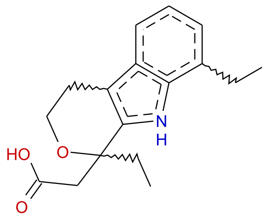	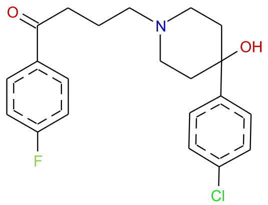
Actual Endpoint (-log C)	3.7286	4.9813	5.3709
Predicted Endpoint (-log C)	2.8638	4.39289	5.15563
Distance	0.619	0.653	0.675
Reference	NDA-17794	NDA-18922	NDA-17473

Model Applicability

Unknown features are fingerprint features in the query molecule, but not found or appearing too infrequently in the training set.

1. All properties and OPS components are within expected ranges.
2. Unknown ECFP_6 feature: -152683720: [*]:[nH]:[*]
3. Unknown ECFP_6 feature: -302078100: [*]Br
4. Unknown ECFP_6 feature: -154530762: [*]N[*]
5. Unknown ECFP_6 feature: -177935549: [*]:[cH]:[c](Br):[cH]:[*]
6. Unknown ECFP_6 feature: 1099224616: [*]:[cH]:[c]1:[nH]:[*]:[*]:[c]:1:[*]
7. Unknown ECFP_6 feature: 1333660716: [*]:[cH]:[c]1:[c](:[*]):[*]:[*]:[c]:1:[*]
8. Unknown ECFP_6 feature: 1336304100: [*]C([*])[c](:[cH]:[*]):[c](:[*]):[*]
9. Unknown ECFP_6 feature: -1020449580: [*][c]1:[*]:[*]:[nH]:[cH]:1
10. Unknown ECFP_6 feature: -953984246: [*]:[c]1:[*]:[*]:[cH]:[nH]:1
11. Unknown ECFP_6 feature: 459826767: [*]:[c](:[*])Br
12. Unknown ECFP_6 feature: -2097294478: [*]:[c](:[*])C1C[*]=[*]C1
13. Unknown ECFP_6 feature: -116689887: [*][C@H]1[*][*]=NC1
14. Unknown ECFP_6 feature: 2090054846: [*]C1=NC[*][*]1
15. Unknown ECFP_6 feature: 103000222: [*]C(=[*])C1=N[*][*]C1
16. Unknown ECFP_6 feature: 53207596: [*]C([*])C[c](:[*]):[*]
17. Unknown ECFP_6 feature: 1431365708: [*]C([*])C(=O)C(=[*])[*]
18. Unknown ECFP_6 feature: -2095227870: [*]C(=[*])[C@@H]1C[*][*]:[c]1:[*]
19. Unknown ECFP_6 feature: -1457159889: [*][C@H]1[*]:[*]NC1
20. Unknown ECFP_6 feature: 1335833675: [*]:[cH]:[c]1N[*][*][c]:1:[*]

21. Unknown ECFP_6 feature: -1694930393: [*]:[c]1:[*][*]CN1
22. Unknown ECFP_6 feature: 1997021792: [*]:[cH]:[cH]:[cH]:[*]

Feature Contribution

Top features for positive contribution

Fingerprint	Bit/Smiles	Feature Structure	Score
ECFP_6	-167460056	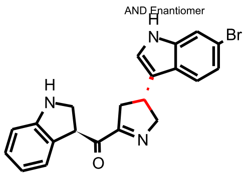 [*]C([*])[*]	0.136
FCFP_6	32	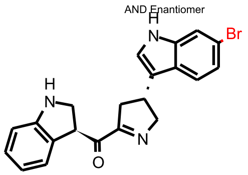 [*]Br	0.101
FCFP_6	3	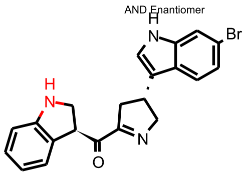 [*]N[*]	0.0924

Top Features for negative contribution

Fingerprint	Bit/Smiles	Feature Structure	Score
FCFP_6	991735244	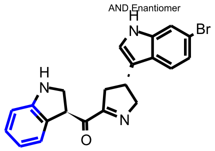 [*]:[c]1:[*]:[cH]:[cH<br>]:[cH]:[cH]:1	-0.134

ECFP_6	1564392544	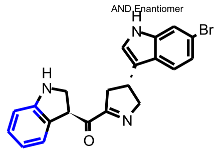<br><br>[*]:[c]1:[*]:[cH]:[cH<br>]:[cH]:[cH]:1	-0.133
FCFP_6	1	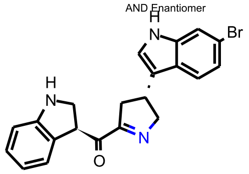<br><br>[*]O[*]	-0.102

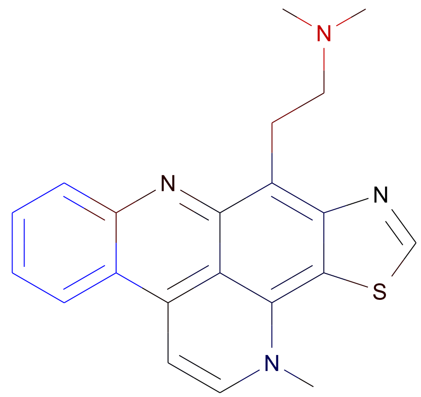

$C_{21}H_{20}N_4S$
Molecular Weight: 360.4753
ALogP: 3.682
Rotatable Bonds: 3
Acceptors: 4
Donors: 0

Structural Similar Compounds

Name	TRIAZOLAM	BROTIZOLAM	ESTAZOLAM
Structure	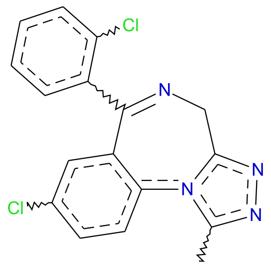	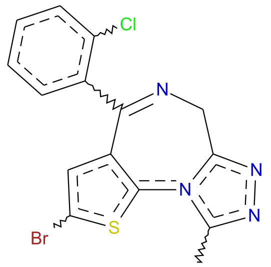	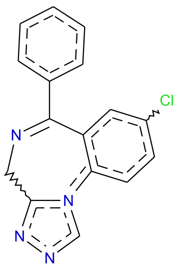
Actual Endpoint (-log C)	3.83659	2.99309	3.99232
Predicted Endpoint (-log C)	3.85527	3.70649	3.88059
Distance	0.634	0.636	0.661
Reference	UPJ-33030	ARZNEI.FORSCH.36.592.1986	NDA-19080

Model Prediction

Prediction: 0.0127
Unit: g/kg_body_weight
Mahalanobis Distance: 37.7
Mahalanobis Distance p-value: 2.32e-038

Mahalanobis Distance: The Mahalanobis distance (MD) is a generalization of the Euclidean distance that accounts for correlations among the X properties. It is calculated as the distance to the center of the training data. The larger the MD, the less trustworthy the prediction.
Mahalanobis Distance p-value: The p-value gives the fraction of training data with an MD greater than or equal to the one for the given sample, assuming normally distributed data. The smaller the p-value, the less trustworthy the prediciton. For highly non-normal X properties (e.g., fingerprints), the MD p-value is wildly inaccurate.

Model Applicability

Unknown features are fingerprint features in the query molecule, but not found or appearing too infrequently in the training set.

1. OPS PC8 out of range. Value: -6.8557. Training min, max, SD, explained variance: -5.7428, 7.3359, 2.68, 0.0314.
2. Unknown ECFP_6 feature: 914325265: [*]:s:[*]
3. Unknown ECFP_6 feature: -1426923364: [*]:[c]1:[*]:[*]:[cH]:s:1
4. Unknown ECFP_6 feature: 1618095312: [*]=CN(C)[c](:[*]):[*]
5. Unknown ECFP_6 feature: -1026752769: [*]CN(C)C
6. Unknown ECFP_6 feature: -677309799: [*][c](:[*]):n:[cH]:[*]
7. Unknown ECFP_6 feature: -1661653144: [*][c](:[*]):[c]1:[c](:[*]):[*]:[*]:[c]:1:[*]
8. Unknown ECFP_6 feature: -512323383: [*]C[c](:[c](:[*]):[*]):[c](:[*]):[*]
9. Unknown ECFP_6 feature: -586331102: [*]N([*])[c](:[c](:[*]):[*]):[c](:[*]):[*]
10. Unknown ECFP_6 feature: 1048320787: [*][c](:[*]):[c](:n:[*]):[c](:[*]):[*]
11. Unknown ECFP_6 feature: -1658647648: [*]=C[c](:[c](:[*]):[*]):[c](:[*]):[*]
12. Unknown ECFP_6 feature: -1795525632: [*]CC[c](:[*]):[*]
13. Unknown ECFP_6 feature: -1673960248: [*][c](:[*]):[c]1:s:[*]:[*]:[c]:1:[*]
14. Unknown ECFP_6 feature: 1333660716: [*]:[cH]:[c]1:[c](:[*]):[*]:[*]:[c]:1:[*]
15. Unknown ECFP_6 feature: -1789942192: [*]CCN([*])[*]
16. Unknown ECFP_6 feature: 1410041175: [*]:[cH]:[c](:n:[*]):[c](:[*]):[*]
17. Unknown ECFP_6 feature: 1745066357: [*]\C=C/[c](:[*]):[*]
18. Unknown ECFP_6 feature: 935510419: [*]\C=C/N([*])[*]

19. Unknown ECFP_6 feature: 866343404: [*]N([*])C
20. Unknown ECFP_6 feature: -225243421: [*]1:[*]:s:[cH]:n:1
21. Unknown ECFP_6 feature: 1997021792: [*]:[cH]:[cH]:[cH]:[*]

Feature Contribution

Top features for positive contribution

Fingerprint	Bit/Smiles	Feature Structure	Score
ECFP_6	1559650422	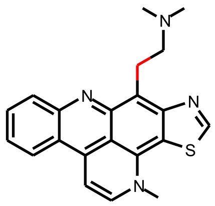 [*]C[*]	0.129
FCFP_6	9	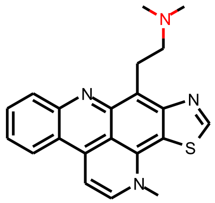 [*]N([*])[*]	0.0797
ECFP_6	834876373	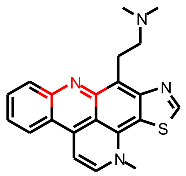 [*]:[c](:[*]):n:[c](:[*]):[*]	0.0634

Top Features for negative contribution

Fingerprint	Bit/Smiles	Feature Structure	Score

FCFP_6	991735244	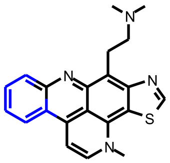<br>[*]:[c]1:[*]:[cH]:[cH<br>]:[cH]:[cH]:1	-0.134
ECFP_6	1564392544	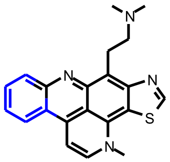<br>[*]:[c]1:[*]:[cH]:[cH<br>]:[cH]:[cH]:1	-0.133
FCFP_6	1	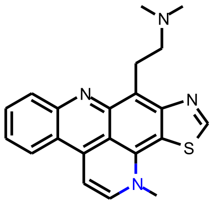<br>[*]O[*]	-0.102

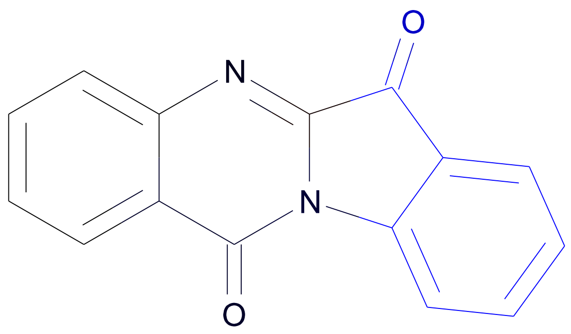

$C_{15}H_8N_2O_2$
Molecular Weight: 248.23621
ALogP: 2.331
Rotatable Bonds: 0
Acceptors: 3
Donors: 0

Structural Similar Compounds

Name	8-METHOXYPSORALEN	TEMAZEPAM	1-AMINO-2-METHYLANTHRAQUINONE
Structure	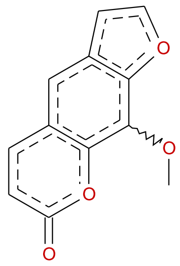	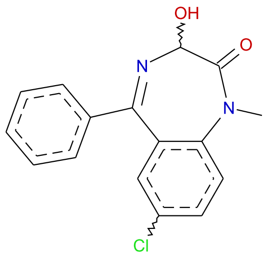	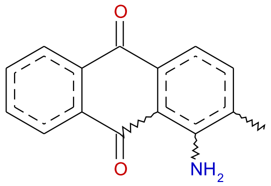
Actual Endpoint (-log C)	3.9067	4.4782	3.6763
Predicted Endpoint (-log C)	4.12187	3.40322	3.47653
Distance	0.501	0.520	0.546
Reference	NTP REPORT # 359	FUND. APPL. TOXICOL. 1984; 4: 394-405	NTP 111 A-4

Model Prediction

Prediction: 0.206
Unit: g/kg_body_weight
Mahalanobis Distance: 31.5
Mahalanobis Distance p-value: 1.91e-027

Mahalanobis Distance: The Mahalanobis distance (MD) is a generalization of the Euclidean distance that accounts for correlations among the X properties. It is calculated as the distance to the center of the training data. The larger the MD, the less trustworthy the prediction.
Mahalanobis Distance p-value: The p-value gives the fraction of training data with an MD greater than or equal to the one for the given sample, assuming normally distributed data. The smaller the p-value, the less trustworthy the prediciton. For highly non-normal X properties (e.g., fingerprints), the MD p-value is wildly inaccurate.

Model Applicability

Unknown features are fingerprint features in the query molecule, but not found or appearing too infrequently in the training set.

1. All properties and OPS components are within expected ranges.
2. Unknown ECFP_6 feature: -962771238: [*]C(=[*])N1C(=[*])[*][*]:[c]1:[*]
3. Unknown ECFP_6 feature: 2085698692: [*]C(=N[c](:[*]):[*])[*]
4. Unknown ECFP_6 feature: -1236953626: [*]N1[*][*][c](:[*]):[c]1:[cH]:[*]
5. Unknown ECFP_6 feature: 671679640: [*]\N=C\1/N([*])[*]:[*]C1=[*]
6. Unknown ECFP_6 feature: 1717462980: [*]C(=[*])C(=O)[c](:[*]):[*]
7. Unknown ECFP_6 feature: 1945129186: [*]N([*])C(=O)[c](:[*]):[*]
8. Unknown ECFP_6 feature: -597295171: [*][c](:[*]):[c](:[cH]:[*])N=[*]
9. Unknown ECFP_6 feature: 1997021792: [*]:[cH]:[cH]:[cH]:[*]

Feature Contribution

Top features for positive contribution

Fingerprint	Bit/Smiles	Feature Structure	Score

ECFP_6	642810091	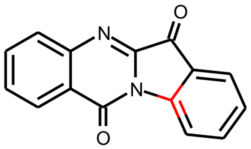 [*]:[c](:[*]):[*]	0.0424
FCFP_6	-2090462286	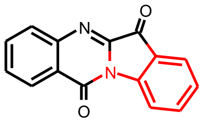 [*]N1[*][*][c]2:[cH]:[cH]:[cH]:[cH]:[c]1:2	0.0369
FCFP_6	0	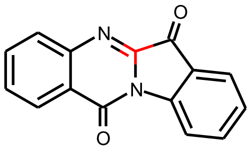 [*]C([*])[*]	0.0177
**Top Features for negative contribution**			
**Fingerprint**	**Bit/Smiles**	**Feature Structure**	**Score**
FCFP_6	991735244	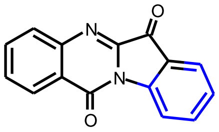 [*]:[c]1:[*]:[cH]:[cH]:[cH]:[cH]:1	-0.134
ECFP_6	1564392544	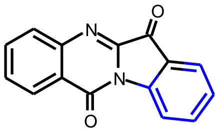 [*]:[c]1:[*]:[cH]:[cH]:[cH]:[cH]:1	-0.133

ECFP_6	2106656448	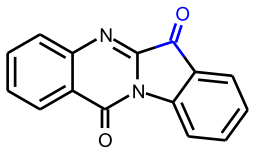 [*]C(=O)[*]	-0.11

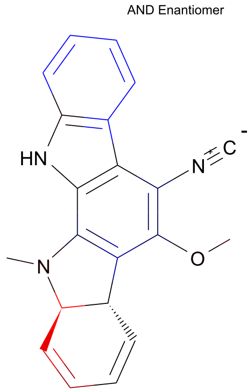

$C_{21}H_{17}N_3O$
Molecular Weight: 327.37918
ALogP: 4.078
Rotatable Bonds: 1
Acceptors: 2
Donors: 1

Model Prediction

Prediction: 0.00458
Unit: g/kg_body_weight
Mahalanobis Distance: 37.4
Mahalanobis Distance p-value: 9.37e-038

Mahalanobis Distance: The Mahalanobis distance (MD) is a generalization of the Euclidean distance that accounts for correlations among the X properties. It is calculated as the distance to the center of the training data. The larger the MD, the less trustworthy the prediction.
Mahalanobis Distance p-value: The p-value gives the fraction of training data with an MD greater than or equal to the one for the given sample, assuming normally distributed data. The smaller the p-value, the less trustworthy the prediciton. For highly non-normal X properties (e.g., fingerprints), the MD p-value is wildly inaccurate.

Structural Similar Compounds

Name	TRIAZOLAM	MIDAZOLAM.HCL	C.I. SOLVENT YELLOW 14
Structure	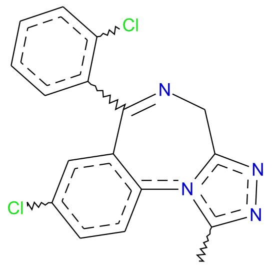	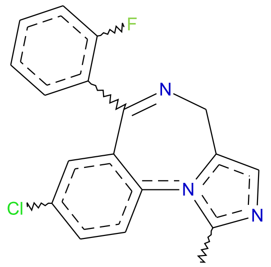	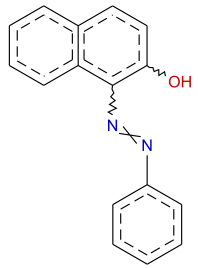
Actual Endpoint (-log C)	3.83659	4.55867	4.298
Predicted Endpoint (-log C)	3.85527	3.94765	3.36361
Distance	0.561	0.571	0.591
Reference	UPJ-33030	NDA-18654	NTP REPORT # 226

Model Applicability

Unknown features are fingerprint features in the query molecule, but not found or appearing too infrequently in the training set.

1. All properties and OPS components are within expected ranges.
2. Unknown FCFP_2 feature: 4: [*]#[C-]
3. Unknown FCFP_2 feature: -828984032: [*][c](:[*]):[c]([N+]#[*]):[c](:[*]):[*]
4. Unknown FCFP_2 feature: 1934974835: [*]:[c](:[*])[N+]#[C-]
5. Unknown FCFP_2 feature: -1487147388: [*][N+]#[C-]
6. Unknown ECFP_6 feature: -152683720: [*]:[nH]:[*]
7. Unknown ECFP_6 feature: 1029014155: [*][N+]#[*]
8. Unknown ECFP_6 feature: 726108635: [*]#[C-]
9. Unknown ECFP_6 feature: -1531301414: [*]O[c](:[c]([*]):[*]):[c](:[*]):[*]
10. Unknown ECFP_6 feature: 1464683384: [*][c](:[*]):[c]([N+]#[*]):[c](:[*]):[*]
11. Unknown ECFP_6 feature: -1661653144: [*][c](:[*]):[c]1:[c](:[*]):[*]:[*]:[c]:1:[*]
12. Unknown ECFP_6 feature: 978230116: [*][c](:[*]):[c]1:[nH]:[*]:[*]:[c]:1:[*]
13. Unknown ECFP_6 feature: -586331102: [*]N([*])[c](:[c](:[*]):[*]):[c](:[*]):[*]
14. Unknown ECFP_6 feature: -1659009760: [*][C@@H]1[*][*][c](:[*]):[c]1:[c]([*]):[*]
15. Unknown ECFP_6 feature: 1333660716: [*]:[cH]:[c]1:[c](:[*]):[*]:[*]:[c]:1:[*]
16. Unknown ECFP_6 feature: 1099224616: [*]:[cH]:[c]1:[nH]:[*]:[*]:[c]:1:[*]
17. Unknown ECFP_6 feature: 558201926: [*]:[c]1:[*]:[*]:[c](:[*]):[nH]:1
18. Unknown ECFP_6 feature: 1617733200: [*][C@@H]1[*][*]:[c](:[*])N1C
19. Unknown ECFP_6 feature: -1337975340: [*][C@H]1[*]:[*]N([*])[C@@H]1C=[*]
20. Unknown ECFP_6 feature: -1565641546: [*][C@H]1[*][*]:[c](:[*])[C@@H]1C=[*]

21. Unknown ECFP_6 feature: 1997021792: [*]:[cH]:[cH]:[cH]:[*]
22. Unknown ECFP_6 feature: 890368401: [*]\C=C/C([*])[*]
23. Unknown ECFP_6 feature: 300955665: [*]\C=C/C=[*]
24. Unknown ECFP_6 feature: 866343404: [*]N([*])C
25. Unknown ECFP_6 feature: 1307307440: [*]:[c](:[*])OC
26. Unknown ECFP_6 feature: -11961319: [*]:[c](:[*])[N+]#[C-]
27. Unknown ECFP_6 feature: -1334780583: [*][N+]#[C-]

Feature Contribution

Top features for positive contribution

Fingerprint	Bit/Smiles	Feature Structure	Score
FCFP_6	451847724	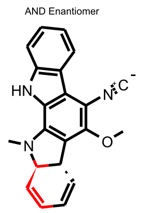 [*]CC=C([*])[*]	0.16
ECFP_6	-167460056	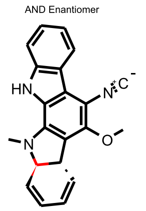 [*]C([*])[*]	0.136
FCFP_6	1036089772	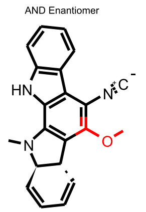 [*]:[c](:[*])OC	0.073

Top Features for negative contribution

Fingerprint	Bit/Smiles	Feature Structure	Score

FCFP_6	991735244	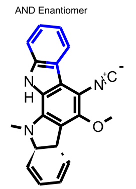<br>[*]:[c]1:[*]:[cH]:[cH]:[cH]:[cH]:1	-0.134
ECFP_6	1564392544	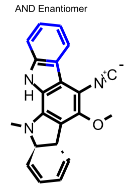<br>[*]:[c]1:[*]:[cH]:[cH]:[cH]:[cH]:1	-0.133
FCFP_6	1	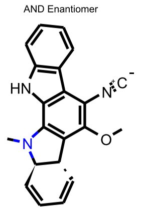<br>[*]O[*]	-0.102

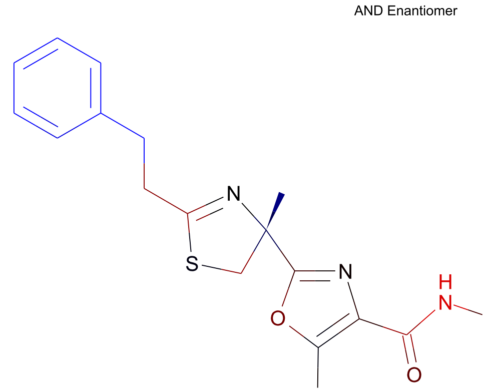

$C_{18}H_{21}N_3O_2S$
Molecular Weight: 343.44324
ALogP: 2.716
Rotatable Bonds: 5
Acceptors: 4
Donors: 1

Model Prediction

Prediction: 0.0462
Unit: g/kg_body_weight
Mahalanobis Distance: 42.2
Mahalanobis Distance p-value: 7.95e-046

Mahalanobis Distance: The Mahalanobis distance (MD) is a generalization of the Euclidean distance that accounts for correlations among the X properties. It is calculated as the distance to the center of the training data. The larger the MD, the less trustworthy the prediction.
Mahalanobis Distance p-value: The p-value gives the fraction of training data with an MD greater than or equal to the one for the given sample, assuming normally distributed data. The smaller the p-value, the less trustworthy the prediciton. For highly non-normal X properties (e.g., fingerprints), the MD p-value is wildly inaccurate.

Structural Similar Compounds

Name	ISOXABEN	DANTROLENE.NA	ETODOLAC
Structure	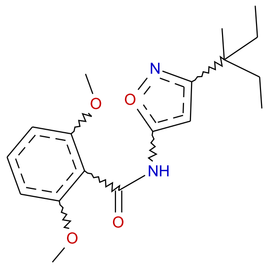	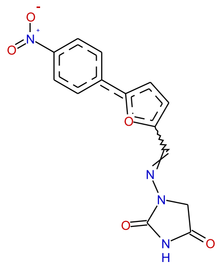	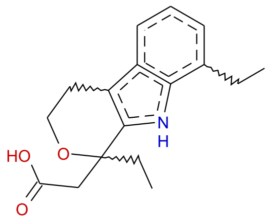
Actual Endpoint (-log C)	3.81665	4.19625	4.9813
Predicted Endpoint (-log C)	4.42315	4.62637	4.39289
Distance	0.524	0.582	0.606
Reference	EPA COVER SHEET 0339;881201;(1)	NDA-17443	NDA-18922

Model Applicability

Unknown features are fingerprint features in the query molecule, but not found or appearing too infrequently in the training set.

1. All properties and OPS components are within expected ranges.
2. Unknown ECFP_6 feature: 912478223: [*]S[*]
3. Unknown ECFP_6 feature: 1203316083: [*][c]1:[*]:[*]:[c]([*]):o:1
4. Unknown ECFP_6 feature: 309047694: [*]C([*])([*])[c]1:o:[*]:[*]:n:1
5. Unknown ECFP_6 feature: 1051700121: [*]C(=[*])[c]1:n:[*]:[*]:[c]:1[*]
6. Unknown ECFP_6 feature: 1576608821: [*][c]1:[*]:[*]:o:[c]:1C
7. Unknown ECFP_6 feature: 1430169877: [*]NC(=O)[c](:[*]):[*]
8. Unknown ECFP_6 feature: 2127097785: [*]C1=[*][*]CS1
9. Unknown ECFP_6 feature: -1073216586: [*]CC1=N[*][*]S1
10. Unknown ECFP_6 feature: 1920241679: [*]C1([*])[*]=[*]SC1
11. Unknown ECFP_6 feature: 618128563: [*]:[c](:[*])[C@]1(C)C[*][*]=N1
12. Unknown ECFP_6 feature: 2085336332: [*]C1=NC([*])([*])[*][*]1
13. Unknown ECFP_6 feature: 859433814: [*]C([*])([*])C
14. Unknown ECFP_6 feature: -1795525632: [*]CC[c](:[*]):[*]
15. Unknown ECFP_6 feature: 1997021792: [*]:[cH]:[cH]:[cH]:[*]
16. Unknown ECFP_6 feature: 1338334141: [*]C(=[*])NC
17. Unknown ECFP_6 feature: 864287155: [*]NC

Feature Contribution

Top features for positive contribution

Fingerprint	Bit/Smiles	Feature Structure	Score
FCFP_6	-1143715940	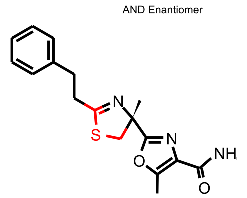 [*]=C1[*][*]CO1	0.13
ECFP_6	1559650422	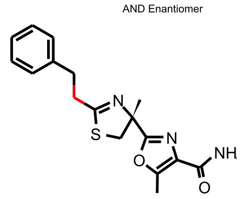 [*]C[*]	0.129
FCFP_6	3	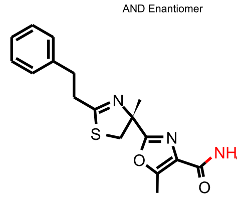 [*]N[*]	0.0924

Top Features for negative contribution

Fingerprint	Bit/Smiles	Feature Structure	Score
FCFP_6	991735244	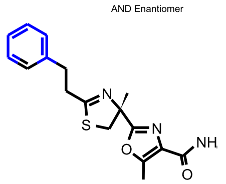 [*]:[c]1:[*]:[cH]:[cH]:[cH]:[cH]:1	-0.134

ECFP_6	1564392544	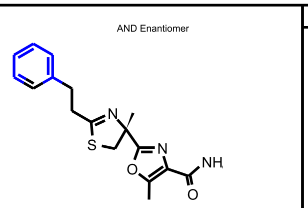 [*]:[c]1:[*]:[cH]:[cH]:[cH]:[cH]:1	-0.133
FCFP_6	1	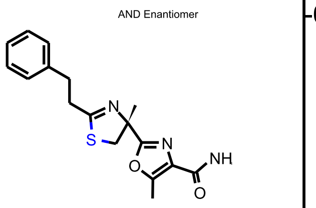 [*]O[*]	-0.102

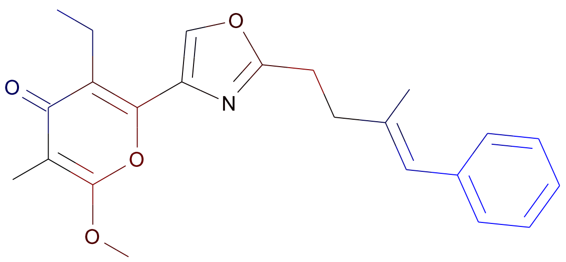

$C_{23}H_{25}NO_4$
Molecular Weight: 379.4489
ALogP: 5.22
Rotatable Bonds: 7
Acceptors: 4
Donors: 0

Model Prediction

Prediction: 0.0234
Unit: g/kg_body_weight
Mahalanobis Distance: 31.5
Mahalanobis Distance p-value: 1.82e-027

Mahalanobis Distance: The Mahalanobis distance (MD) is a generalization of the Euclidean distance that accounts for correlations among the X properties. It is calculated as the distance to the center of the training data. The larger the MD, the less trustworthy the prediction.
Mahalanobis Distance p-value: The p-value gives the fraction of training data with an MD greater than or equal to the one for the given sample, assuming normally distributed data. The smaller the p-value, the less trustworthy the prediciton. For highly non-normal X properties (e.g., fingerprints), the MD p-value is wildly inaccurate.

Structural Similar Compounds

Name	BAYTHROID	CYHALOTHRIN	PERMETHRIN
Structure	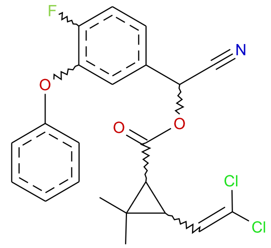	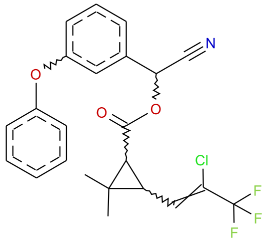	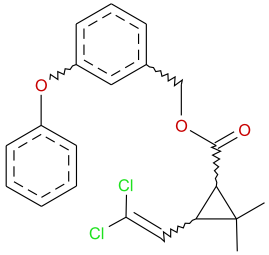
Actual Endpoint (-log C)	4.76272	5.47699	4.19456
Predicted Endpoint (-log C)	5.1129	5.48165	5.1846
Distance	0.484	0.536	0.537
Reference	EPA COVER SHEET 0132;891101;(1)	EPA COVER SHEET 0279;880822;(1)	FUND.APPL.TOXICOL.11. 308.1988

Model Applicability

Unknown features are fingerprint features in the query molecule, but not found or appearing too infrequently in the training set.

1. OPS PC28 out of range. Value: -4.0006. Training min, max, SD, explained variance: -3.911, 3.7469, 1.37, 0.0082.
2. Unknown FCFP_2 feature: -2115241127: [*]OC(=C([*])[*])O[*]
3. Unknown ECFP_6 feature: -560785749: [*]C(=[*])OC(=[*])[*]
4. Unknown ECFP_6 feature: -308870089: [*][c]1:[*]:[*]:[cH]:o:1
5. Unknown ECFP_6 feature: 1307307440: [*]:[c](:[*])OC
6. Unknown ECFP_6 feature: -770854792: [*]CC(=C([*])[*])C(=[*])[*]
7. Unknown ECFP_6 feature: -1795525632: [*]CC[c](:[*]):[*]
8. Unknown ECFP_6 feature: -1789102870: [*]CCC(=[*])[*]
9. Unknown ECFP_6 feature: 1796421070: [*]OC(=C([*])[*])[c](:[*]):[*]
10. Unknown ECFP_6 feature: 767488533: [*]C(=[*])CC
11. Unknown ECFP_6 feature: -428002189: [*]C(=[*])[c]1:[cH]:[*]:[*]:n:1
12. Unknown ECFP_6 feature: -435589429: [*]C[c]1:o:[*]:[*]:n:1
13. Unknown ECFP_6 feature: 431707261: [*]C\C(=C\[*])\C
14. Unknown ECFP_6 feature: 1717462980: [*]C(=[*])C(=O)[c](:[*]):[*]
15. Unknown ECFP_6 feature: 1792159373: [*]C(=C(C)C(=[*])[*])[*]
16. Unknown ECFP_6 feature: 1651701028: [*]OC(=C([*])[*])O[*]
17. Unknown ECFP_6 feature: -785659985: [*][c]1:[*]:[*]:o:[cH]:1
18. Unknown ECFP_6 feature: -1832568576: [*]C(=C[c](:[*]):[*])[*]

19. Unknown ECFP_6 feature: -176483725: [*]=C[c](:[cH]:[*]):[cH]:[*]
20. Unknown ECFP_6 feature: 1997021792: [*]:[cH]:[cH]:[cH]:[*]

Feature Contribution

Top features for positive contribution			
**Fingerprint**	**Bit/Smiles**	**Feature Structure**	**Score**
FCFP_6	-1143715940	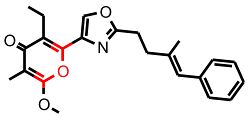 [*]=C1[*][*]CO1	0.13
ECFP_6	1559650422	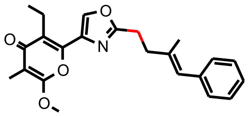 [*]C[*]	0.129
ECFP_6	-1925046727	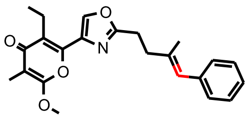 [*]C=[*]	0.0915

Top Features for negative contribution			
**Fingerprint**	**Bit/Smiles**	**Feature Structure**	**Score**
FCFP_6	991735244	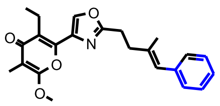 [*]:[c]1:[*]:[cH]:[cH]:[cH]:[cH]:1	-0.134

ECFP_6	1564392544	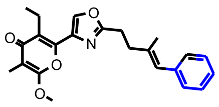 [*]:[c]1:[*]:[cH]:[cH]:[cH]:[cH]:1	-0.133
ECFP_6	2106656448	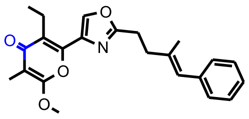 [*]C(=O)[*]	-0.11

Remdesivir

TOPKAT_Chronic_LOAEL

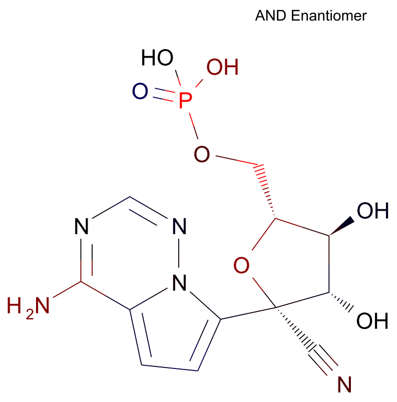

$C_{12}H_{14}N_5O_7P$
Molecular Weight: 371.24262
ALogP: -1.502
Rotatable Bonds: 4
Acceptors: 11
Donors: 5

Model Prediction

Prediction: 0.00379
Unit: g/kg_body_weight
Mahalanobis Distance: 47.7
Mahalanobis Distance p-value: 2.93e-054

Mahalanobis Distance: The Mahalanobis distance (MD) is a generalization of the Euclidean distance that accounts for correlations among the X properties. It is calculated as the distance to the center of the training data. The larger the MD, the less trustworthy the prediction.
Mahalanobis Distance p-value: The p-value gives the fraction of training data with an MD greater than or equal to the one for the given sample, assuming normally distributed data. The smaller the p-value, the less trustworthy the prediciton. For highly non-normal X properties (e.g., fingerprints), the MD p-value is wildly inaccurate.

Structural Similar Compounds

Name	TETRACYCLINE .HCL	4;4'-DIAMINO-2;2'-STILBENEDIS	OXYTETRACYCLINE .HCL
Structure	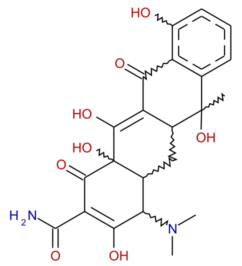	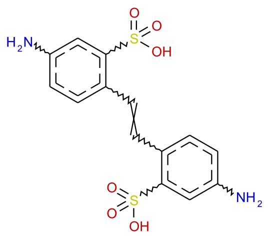	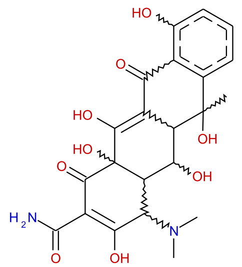
Actual Endpoint (-log C)	2.85193	2.47175	2.56626
Predicted Endpoint (-log C)	3.94748	3.53715	3.75581
Distance	0.746	0.746	0.802
Reference	NTP REPORT # 344	NTP 412 82	NTP REPORT # 315

Model Applicability

Unknown features are fingerprint features in the query molecule, but not found or appearing too infrequently in the training set.

1. All properties and OPS components are within expected ranges.
2. Unknown FCFP_2 feature: 472180098: [*]OP(=O)(O)O
3. Unknown FCFP_2 feature: -332197802: [*][c]1:[*]:[*]:[c](:[*]):n:1:n:[*]
4. Unknown ECFP_6 feature: -1114776580: [*]C#[*]
5. Unknown ECFP_6 feature: -1101847286: [*]#N
6. Unknown ECFP_6 feature: 672362763: [*]:n(:[*]):[*]
7. Unknown ECFP_6 feature: 1126642748: [*]OP(=O)(O)O
8. Unknown ECFP_6 feature: 2100964382: [*]P(=O)([*])[*]
9. Unknown ECFP_6 feature: 2024329577: [*]P(=[*])([*])O
10. Unknown ECFP_6 feature: -1250439909: [*]COP(=[*])([*])[*]
11. Unknown ECFP_6 feature: -1687549011: [*]OCC([*])[*]
12. Unknown ECFP_6 feature: -194719409: [*][C@@H]1[*][*]C([*])([*])O1
13. Unknown ECFP_6 feature: -553149446: [*]C[C@H]1O[*][*][C@@H]1[*]
14. Unknown ECFP_6 feature: 305695353: [*][C@@H]1[*][*][C@H]([*])C1O
15. Unknown ECFP_6 feature: -521596699: [*][C@@H]1[*][*]C([*])([*])[C@H]1O
16. Unknown ECFP_6 feature: 1258791451: [*][C@H]1[*][*]O[C@]1(C#[*])[c](:[*]):[*]
17. Unknown ECFP_6 feature: 2024749573: [*]C([*])O
18. Unknown ECFP_6 feature: -264833661: [*]C([*])([*])C#N
19. Unknown ECFP_6 feature: 1412053881: [*]C#N

20. Unknown ECFP_6 feature: -1507082173: [*][c]1:[*]:[*]:[c](:[*]):n:1:n:[*]
21. Unknown ECFP_6 feature: -676555381: [*]:[cH]:n:n(:[*]):[*]
22. Unknown ECFP_6 feature: -710237522: [*]:n:[cH]:n:[*]
23. Unknown ECFP_6 feature: -677309799: [*][c](:[*]):n:[cH]:[*]
24. Unknown ECFP_6 feature: -1734834311: [*]:n:[c](N):[c](:[*]):[*]
25. Unknown ECFP_6 feature: 1334415134: [*][c](:[*]):[c]1:[cH]:[*]:[*]:n:1:[*]
26. Unknown ECFP_6 feature: -66263742: [*]C([*])([*])[c]1:[cH]:[*]:[*]:n:1:[*]
27. Unknown ECFP_6 feature: -938530932: [*]:[c](:[*])N

Feature Contribution

Top features for positive contribution			
**Fingerprint**	**Bit/Smiles**	**Feature Structure**	**Score**
ECFP_6	-167460056	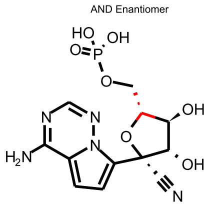 [*]C([*])[*]	0.136
FCFP_6	-1143715940	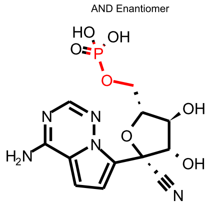 [*]=C1[*][*]CO1	0.13
ECFP_6	1559650422	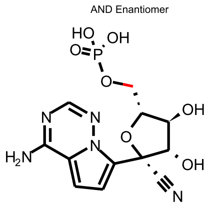 [*]C[*]	0.129

Top Features for negative contribution			
**Fingerprint**	**Bit/Smiles**	**Feature Structure**	**Score**

FCFP_6	1	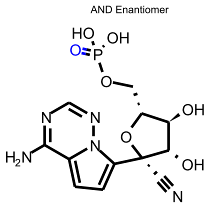 [*]O[*]	-0.102
ECFP_6	1996767644	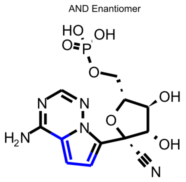 [*]:[cH]:[cH]:[c](:[*]):[*]	-0.0497
FCFP_6	16	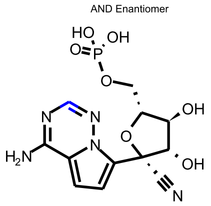 [*]:[cH]:[*]	-0.0462

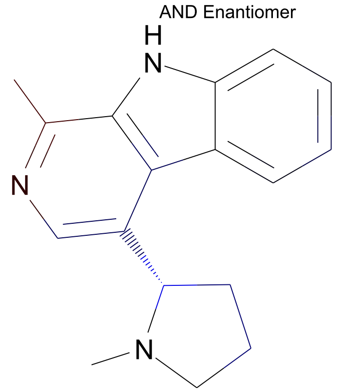

$C_{17}H_{19}N_3$

Molecular Weight: 265.35286

ALogP: 3.018

Rotatable Bonds: 1

Acceptors: 2

Donors: 1

Structural Similar Compounds

Name	D&C YELLOW NO. 11	N-PHENYL-2-NAPTHTHYLAMINE	DOXYLAMINE
Structure	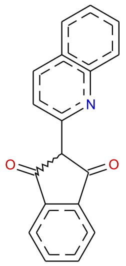	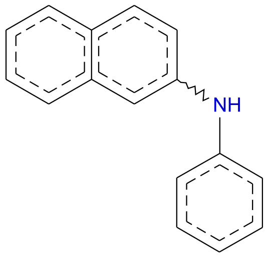	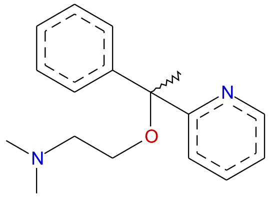
Actual Endpoint (-log C)	4.03869	2.98883	3.47773
Predicted Endpoint (-log C)	3.54593	3.29932	3.48742
Distance	0.543	0.626	0.638
Reference	NCI/NTP TR-463	NCI/NTP TR-333	NCI/NTP Report 10, Nov. 1987

Model Prediction

Prediction: 0.0904

Unit: g/kg_body_weight

Mahalanobis Distance: 7.8

Mahalanobis Distance p-value: 0.0392

Mahalanobis Distance: The Mahalanobis distance (MD) is a generalization of the Euclidean distance that accounts for correlations among the X properties. It is calculated as the distance to the center of the training data. The larger the MD, the less trustworthy the prediction.
Mahalanobis Distance p-value: The p-value gives the fraction of training data with an MD greater than or equal to the one for the given sample, assuming normally distributed data. The smaller the p-value, the less trustworthy the prediciton. For highly non-normal X properties (e.g., fingerprints), the MD p-value is wildly inaccurate.

Model Applicability

Unknown features are fingerprint features in the query molecule, but not found or appearing too infrequently in the training set.

1. All properties and OPS components are within expected ranges.

Feature Contribution

Top features for positive contribution

Fingerprint	Bit/Smiles	Feature Structure	Score
FCFP_2	136120670	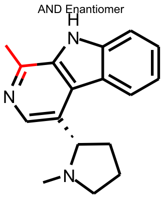 [*]:[c](:[*])C	0.064

FCFP_2	17	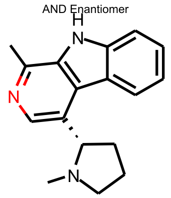 [*]:n:[*]	0.0441
**Top Features for negative contribution**			
**Fingerprint**	**Bit/Smiles**	**Feature Structure**	**Score**
FCFP_2	-1272798659	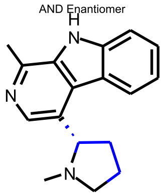 [*][C@H]1[*][*]CC1	-0.111
FCFP_2	203677720	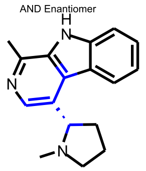 [*]C([*])[c](:[cH]:[*]):[c](:[*]):[*]	-0.0829
FCFP_2	16	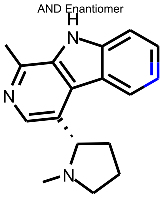 [*]:[cH]:[*]	-0.0512

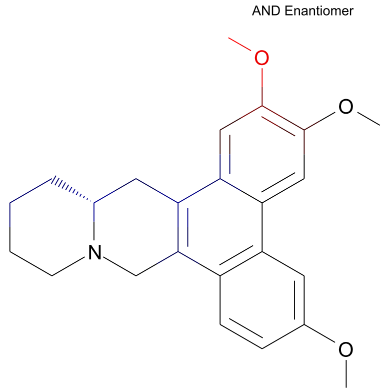

$C_{24}H_{27}NO_3$
Molecular Weight: 377.47608
ALogP: 4.691
Rotatable Bonds: 3
Acceptors: 4
Donors: 0

Structural Similar Compounds

Name	METHOXYCHLOR	MICHLER'S KETONE	PYRILAMINE
Structure	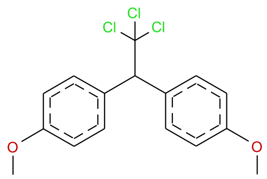	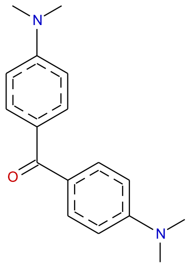	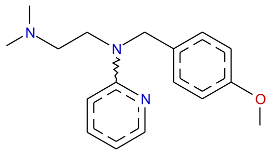
Actual Endpoint (-log C)	3.95856	4.07654	3.32511
Predicted Endpoint (-log C)	4.08958	3.57549	3.65163
Distance	0.597	0.629	0.641
Reference	NCI/NTP TR-35	NCI/NTP TR-181	NCI/NTP Report 10, Nov. 1987

Model Applicability

Unknown features are fingerprint features in the query molecule, but not found or appearing too infrequently in the training set.

1. All properties and OPS components are within expected ranges.
2. Unknown FCFP_2 feature: 906798516: [*]N([*])C[c](:[*]):[*]

Model Prediction

Prediction: 0.0385
Unit: g/kg_body_weight
Mahalanobis Distance: 6.66
Mahalanobis Distance p-value: 0.323

Mahalanobis Distance: The Mahalanobis distance (MD) is a generalization of the Euclidean distance that accounts for correlations among the X properties. It is calculated as the distance to the center of the training data. The larger the MD, the less trustworthy the prediction.
Mahalanobis Distance p-value: The p-value gives the fraction of training data with an MD greater than or equal to the one for the given sample, assuming normally distributed data. The smaller the p-value, the less trustworthy the prediciton. For highly non-normal X properties (e.g., fingerprints), the MD p-value is wildly inaccurate.

Feature Contribution

Top features for positive contribution

Fingerprint	Bit/Smiles	Feature Structure	Score
FCFP_2	136627117	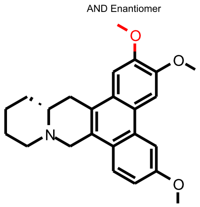 [*]OC	0.173

FCFP_2	1036089772	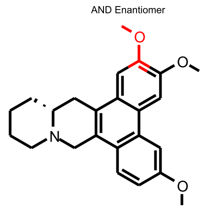 [*]:[c](:[*])OC	0.0749
FCFP_2	332760439	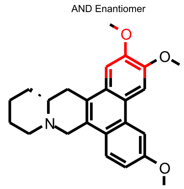 [*]O[c](:[cH]:[*]):[c]([*]):[*]	0.0611

Top Features for negative contribution

Fingerprint	Bit/Smiles	Feature Structure	Score
FCFP_2	-1272798659	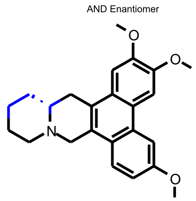 [*][C@H]1[*][*]CC1	-0.111
FCFP_2	203677720	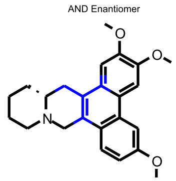 [*]C([*])[c](:[cH]:[*]):[c](:[*]):[*]	-0.0829
FCFP_2	1	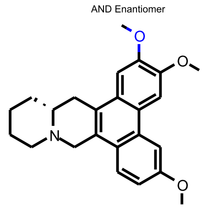 [*]O[*]	-0.0796

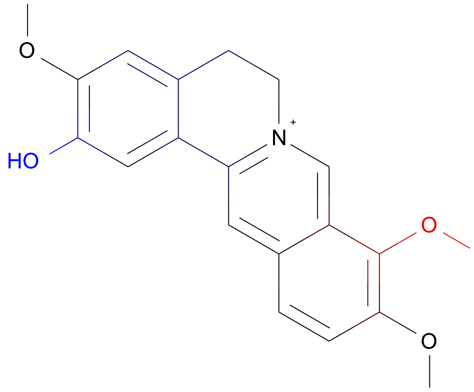

$C_{20}H_{20}NO_4$
Molecular Weight: 338.3771
ALogP: 3.936
Rotatable Bonds: 3
Acceptors: 4
Donors: 1

Model Prediction

Prediction: 0.119
Unit: g/kg_body_weight
Mahalanobis Distance: 5.99
Mahalanobis Distance p-value: 0.644

Mahalanobis Distance: The Mahalanobis distance (MD) is a generalization of the Euclidean distance that accounts for correlations among the X properties. It is calculated as the distance to the center of the training data. The larger the MD, the less trustworthy the prediction.
Mahalanobis Distance p-value: The p-value gives the fraction of training data with an MD greater than or equal to the one for the given sample, assuming normally distributed data. The smaller the p-value, the less trustworthy the prediciton. For highly non-normal X properties (e.g., fingerprints), the MD p-value is wildly inaccurate.

Structural Similar Compounds

Name	C.I. SOLVENT YELLOW 14	PHENOLPHTHALEIN	C.I.PIGMENT RED 3
Structure	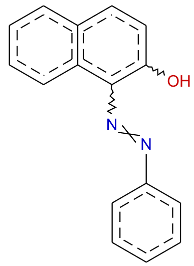	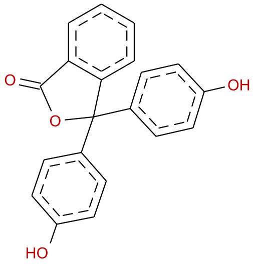	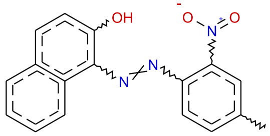
Actual Endpoint (-log C)	4.04277	2.20184	2.65635
Predicted Endpoint (-log C)	2.8989	2.8857	2.97957
Distance	0.503	0.524	0.561
Reference	NCI/NTP TR-226	NCI/NTP TR-465	NCI/NTP TR-407

Model Applicability

Unknown features are fingerprint features in the query molecule, but not found or appearing too infrequently in the training set.

1. All properties and OPS components are within expected ranges.
2. Unknown FCFP_2 feature: 24: [*][n+](:[*]):[*]
3. Unknown FCFP_2 feature: 414371600: [*]C[n+](:[c]([*]):[*]):c:[*]
4. Unknown FCFP_2 feature: -150573739: [*]CC[n+](:[*]):[*]
5. Unknown FCFP_2 feature: -1861407456: [*][n+](:[*]):[c]([c](:[*]):[*]):c:[*]
6. Unknown FCFP_2 feature: 1618392993: [*][n+](:[*]):c:[c](:[*]):[*]

Feature Contribution

Top features for positive contribution

Fingerprint	Bit/Smiles	Feature Structure	Score
FCFP_2	136627117	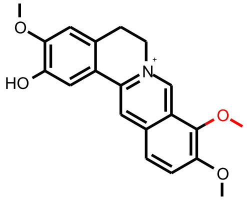 [*]OC	0.173

FCFP_2	1036089772	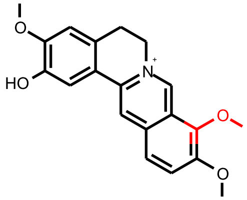 [*]:[c](:[*])OC	0.0749
FCFP_2	332760439	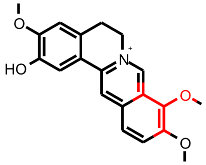 [*]O[c](:[cH]:[*]):[c]([*]):[*]	0.0611

Top Features for negative contribution

Fingerprint	Bit/Smiles	Feature Structure	Score
FCFP_2	7	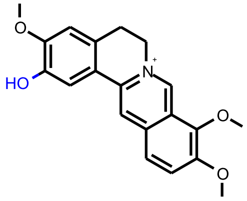 [*]O	-0.214
FCFP_2	-549108873	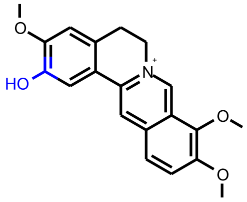 [*]:[c](:[*])O	-0.127
FCFP_2	203677720	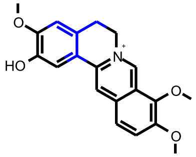 [*]C([*])[c](:[cH]:[*]):[c](:[*]):[*]	-0.0829

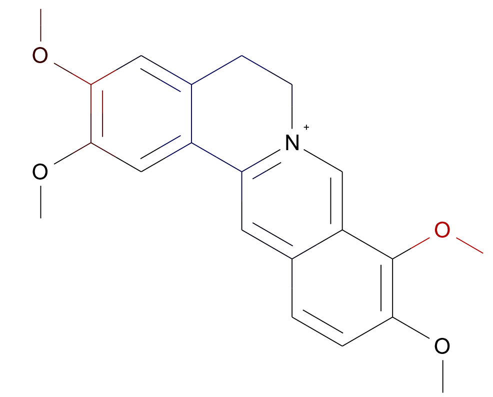

$C_{21}H_{22}NO_4$
Molecular Weight: 352.40368
ALogP: 4.161
Rotatable Bonds: 4
Acceptors: 4
Donors: 0

Model Prediction

Prediction: 0.0351
Unit: g/kg_body_weight
Mahalanobis Distance: 5.47
Mahalanobis Distance p-value: 0.858

Mahalanobis Distance: The Mahalanobis distance (MD) is a generalization of the Euclidean distance that accounts for correlations among the X properties. It is calculated as the distance to the center of the training data. The larger the MD, the less trustworthy the prediction.
Mahalanobis Distance p-value: The p-value gives the fraction of training data with an MD greater than or equal to the one for the given sample, assuming normally distributed data. The smaller the p-value, the less trustworthy the prediciton. For highly non-normal X properties (e.g., fingerprints), the MD p-value is wildly inaccurate.

Structural Similar Compounds

Name	MICHLER'S KETONE	METHOXYCHLOR	ROTENONE
Structure	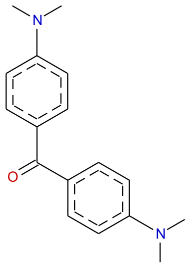	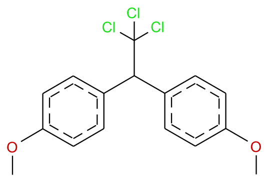	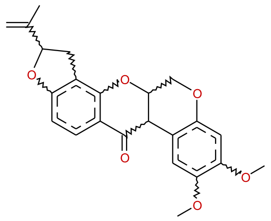
Actual Endpoint (-log C)	4.07654	3.95856	5.06769
Predicted Endpoint (-log C)	3.57549	4.08958	4.11907
Distance	0.602	0.605	0.613
Reference	NCI/NTP TR-181	NCI/NTP TR-35	NCI/NTP TR-320

Model Applicability

Unknown features are fingerprint features in the query molecule, but not found or appearing too infrequently in the training set.

1. All properties and OPS components are within expected ranges.
2. Unknown FCFP_2 feature: 24: [*][n+](:[*]):[*]
3. Unknown FCFP_2 feature: 414371600: [*]C[n+](:[c]([*]):[*]):c:[*]
4. Unknown FCFP_2 feature: -150573739: [*]CC[n+](:[*]):[*]
5. Unknown FCFP_2 feature: -1861407456: [*][n+](:[*]):[c]([c](:[*]):[*]):c:[*]
6. Unknown FCFP_2 feature: 1618392993: [*][n+](:[*]):c:[c](:[*]):[*]

Feature Contribution

Top features for positive contribution

Fingerprint	Bit/Smiles	Feature Structure	Score
FCFP_2	136627117	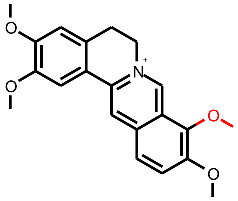 [*]OC	0.173

FCFP_2	1036089772	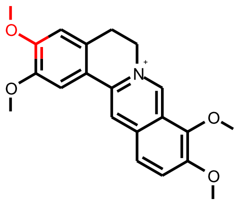 [*]:[c](:[*])OC	0.0749
FCFP_2	332760439	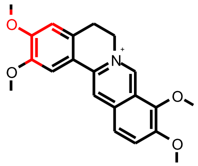 [*]O[c](:[cH]:[*]):[c](([*]):[*]	0.0611

Top Features for negative contribution

Fingerprint	Bit/Smiles	Feature Structure	Score
FCFP_2	203677720	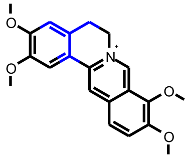 [*]C([*])[c](:[cH]:[*]):[c](:[*]):[*]	-0.0829
FCFP_2	1	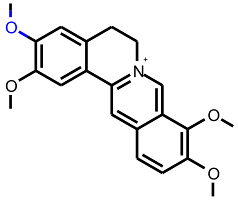 [*]O[*]	-0.0796
FCFP_2	16	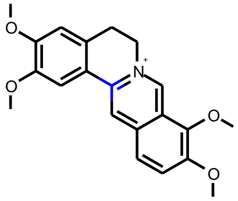 [*]:[cH]:[*]	-0.0512

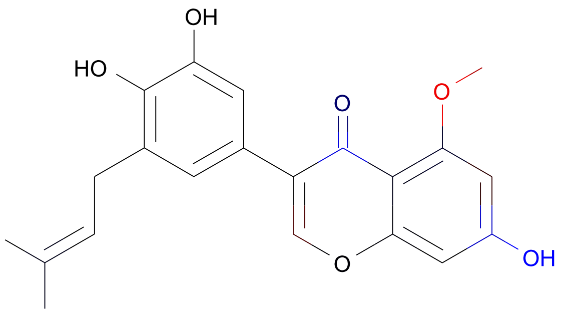

$C_{21}H_{20}O_6$
Molecular Weight: 368.3799
ALogP: 3.98
Rotatable Bonds: 4
Acceptors: 6
Donors: 3

Model Prediction

Prediction: 0.344
Unit: g/kg_body_weight
Mahalanobis Distance: 6.23
Mahalanobis Distance p-value: 0.529

Mahalanobis Distance: The Mahalanobis distance (MD) is a generalization of the Euclidean distance that accounts for correlations among the X properties. It is calculated as the distance to the center of the training data. The larger the MD, the less trustworthy the prediction.
Mahalanobis Distance p-value: The p-value gives the fraction of training data with an MD greater than or equal to the one for the given sample, assuming normally distributed data. The smaller the p-value, the less trustworthy the prediciton. For highly non-normal X properties (e.g., fingerprints), the MD p-value is wildly inaccurate.

Structural Similar Compounds

Name	DISPERSE YELLOW 3	PHENOLPHTHALEIN	PROPYL GALLATE
Structure	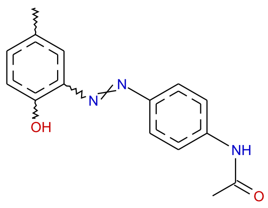	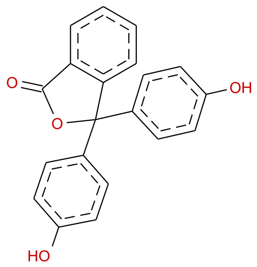	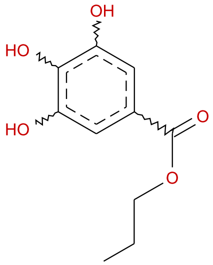
Actual Endpoint (-log C)	2.77703	2.20184	2.59435
Predicted Endpoint (-log C)	2.80195	2.8857	2.18569
Distance	0.606	0.669	0.679
Reference	NCI/NTP TR-222	NCI/NTP TR-465	NCI/NTP TR-240

Model Applicability

Unknown features are fingerprint features in the query molecule, but not found or appearing too infrequently in the training set.

1. All properties and OPS components are within expected ranges.

Feature Contribution

Top features for positive contribution			
**Fingerprint**	**Bit/Smiles**	**Feature Structure**	**Score**
FCFP_2	136627117	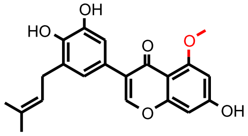 [*]OC	0.173

FCFP_2	1036089772	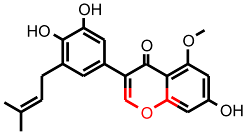 [*]:[c](:[*])OC	0.0749
FCFP_2	332760439	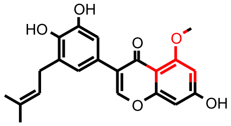 [*]O[c](:[cH]:[*]):[c]([*]):[*]	0.0611
**Top Features for negative contribution**			
**Fingerprint**	**Bit/Smiles**	**Feature Structure**	**Score**
FCFP_2	7	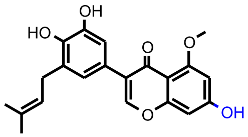 [*]O	-0.214
FCFP_2	-549108873	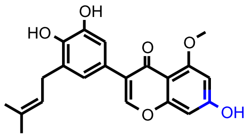 [*]:[c](:[*])O	-0.127
FCFP_2	1872154524	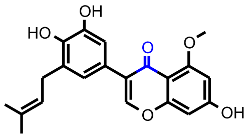 [*]C(=O)[*]	-0.105

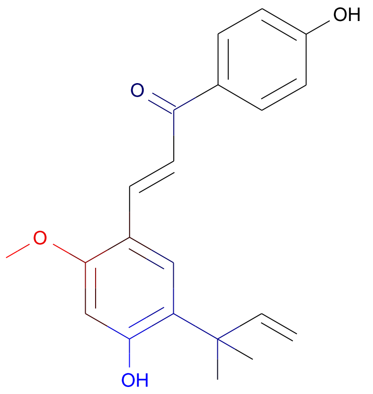

$C_{21}H_{22}O_4$
Molecular Weight: 338.39698
ALogP: 4.667
Rotatable Bonds: 6
Acceptors: 4
Donors: 2

Model Prediction

Prediction: 0.303
Unit: g/kg_body_weight
Mahalanobis Distance: 6.12
Mahalanobis Distance p-value: 0.583

Mahalanobis Distance: The Mahalanobis distance (MD) is a generalization of the Euclidean distance that accounts for correlations among the X properties. It is calculated as the distance to the center of the training data. The larger the MD, the less trustworthy the prediction.
Mahalanobis Distance p-value: The p-value gives the fraction of training data with an MD greater than or equal to the one for the given sample, assuming normally distributed data. The smaller the p-value, the less trustworthy the prediciton. For highly non-normal X properties (e.g., fingerprints), the MD p-value is wildly inaccurate.

Structural Similar Compounds

Name	4,4'-THIOBIS(6-t-BUTYL-m-CRESOL)	DISPERSE YELLOW 3	CHLORBENZILATE
Structure	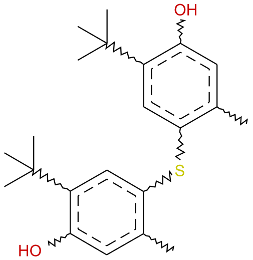	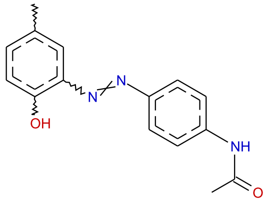	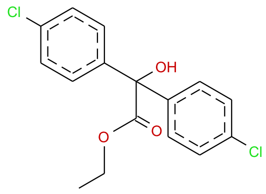
Actual Endpoint (-log C)	3.55454	2.77703	3.38252
Predicted Endpoint (-log C)	3.06707	2.80195	3.27894
Distance	0.495	0.499	0.593
Reference	NCI/NTP TR-435	NCI/NTP TR-222	NCI/NTP TR-75

Model Applicability

Unknown features are fingerprint features in the query molecule, but not found or appearing too infrequently in the training set.

1. All properties and OPS components are within expected ranges.

Feature Contribution

Top features for positive contribution			
**Fingerprint**	**Bit/Smiles**	**Feature Structure**	**Score**
FCFP_2	136627117	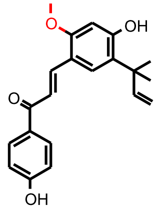 [*]OC	0.173

FCFP_2	1036089772	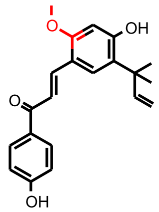 [*]:[c](:[*])OC	0.0749
FCFP_2	332760439	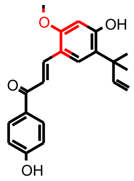 [*]O[c](:[cH]:[*]):[c]([*]):[*]	0.0611

Top Features for negative contribution

**Fingerprint**	**Bit/Smiles**	**Feature Structure**	**Score**
FCFP_2	7	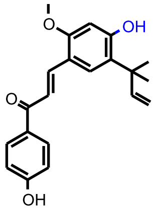 [*]O	-0.214
FCFP_2	-549108873	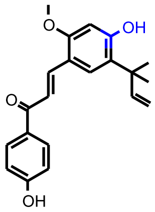 [*]:[c](:[*])O	-0.127
FCFP_2	1872154524	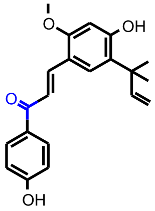 [*]C(=O)[*]	-0.105

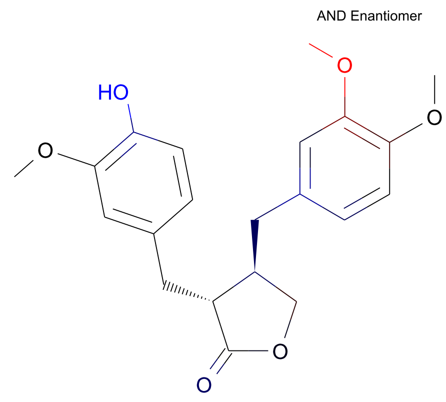

$C_{21}H_{24}O_6$
Molecular Weight: 372.41166
ALogP: 3.743
Rotatable Bonds: 7
Acceptors: 6
Donors: 1

Structural Similar Compounds

Name	3,3'-DIMETHOXYBENZIDINE-4,4'-DIISOCYANATE	ROTENONE	COUMAPHOS
Structure	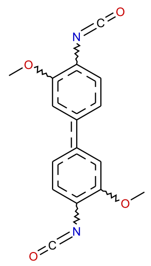	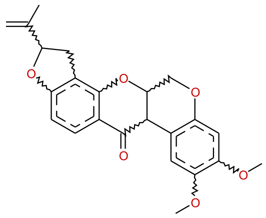	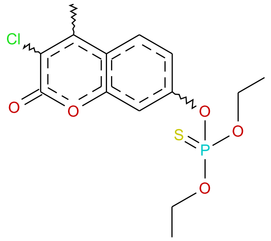
Actual Endpoint (-log C)	2.17504	5.06769	5.60537
Predicted Endpoint (-log C)	3.78717	4.11907	4.15004
Distance	0.532	0.545	0.548
Reference	NCI/NTP TR-128	NCI/NTP TR-320	NCI/NTP TR-96

Model Prediction

Prediction: 0.149
Unit: g/kg_body_weight
Mahalanobis Distance: 9.42
Mahalanobis Distance p-value: 0.000348

Mahalanobis Distance: The Mahalanobis distance (MD) is a generalization of the Euclidean distance that accounts for correlations among the X properties. It is calculated as the distance to the center of the training data. The larger the MD, the less trustworthy the prediction.
Mahalanobis Distance p-value: The p-value gives the fraction of training data with an MD greater than or equal to the one for the given sample, assuming normally distributed data. The smaller the p-value, the less trustworthy the prediciton. For highly non-normal X properties (e.g., fingerprints), the MD p-value is wildly inaccurate.

Model Applicability

Unknown features are fingerprint features in the query molecule, but not found or appearing too infrequently in the training set.

1. All properties and OPS components are within expected ranges.

Feature Contribution

Top features for positive contribution

Fingerprint	Bit/Smiles	Feature Structure	Score
FCFP_2	136627117	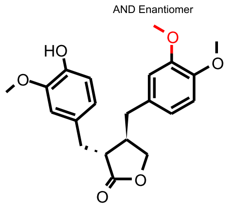 [*]OC	0.173

FCFP_2	-1143715940	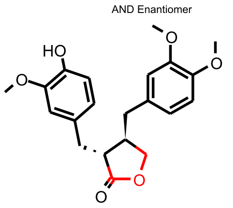 [*]=C1[*][*]CO1	0.095
FCFP_2	1036089772	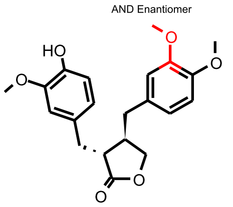 [*]:[c](:[*])OC	0.0749
**Top Features for negative contribution**			
**Fingerprint**	**Bit/Smiles**	**Feature Structure**	**Score**
FCFP_2	7	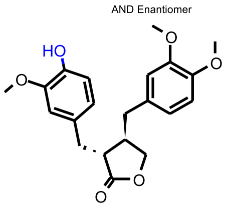 [*]O	-0.214
FCFP_2	-549108873	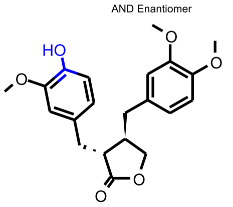 [*]:[c](:[*])O	-0.127
FCFP_2	1872154524	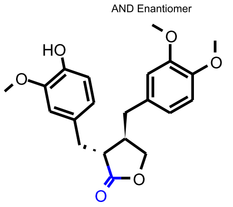 [*]C(=O)[*]	-0.105

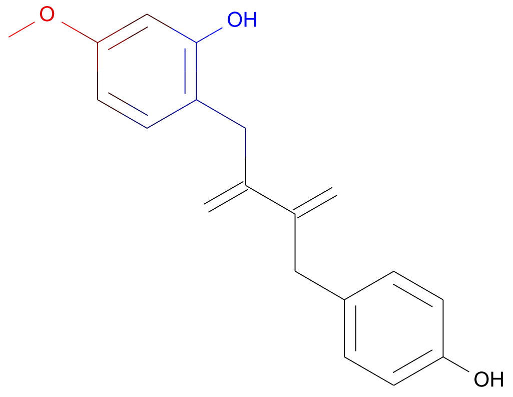

$C_{19}H_{20}O_3$
Molecular Weight: 296.3603
ALogP: 4.784
Rotatable Bonds: 6
Acceptors: 3
Donors: 2

Model Prediction

Prediction: 0.312
Unit: g/kg_body_weight
Mahalanobis Distance: 5.88
Mahalanobis Distance p-value: 0.696

Mahalanobis Distance: The Mahalanobis distance (MD) is a generalization of the Euclidean distance that accounts for correlations among the X properties. It is calculated as the distance to the center of the training data. The larger the MD, the less trustworthy the prediction.
Mahalanobis Distance p-value: The p-value gives the fraction of training data with an MD greater than or equal to the one for the given sample, assuming normally distributed data. The smaller the p-value, the less trustworthy the prediciton. For highly non-normal X properties (e.g., fingerprints), the MD p-value is wildly inaccurate.

Structural Similar Compounds

Name	EUGENOL	4,4'-THIOBIS(6-t-BUTYL-m-CRESOL)	DISPERSE YELLOW 3
Structure	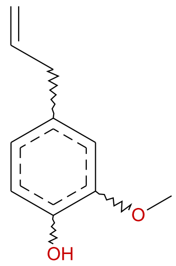	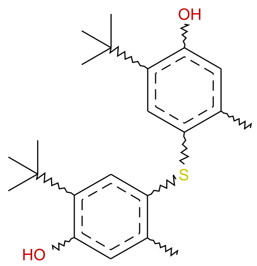	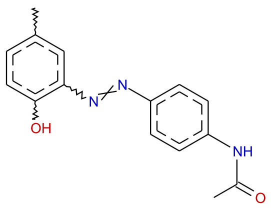
Actual Endpoint (-log C)	2.78402	3.55454	2.77703
Predicted Endpoint (-log C)	2.98617	3.06707	2.80195
Distance	0.518	0.532	0.544
Reference	NCI/NTP TR-223	NCI/NTP TR-435	NCI/NTP TR-222

Model Applicability

Unknown features are fingerprint features in the query molecule, but not found or appearing too infrequently in the training set.

1. All properties and OPS components are within expected ranges.

Feature Contribution

Top features for positive contribution			
**Fingerprint**	**Bit/Smiles**	**Feature Structure**	**Score**
FCFP_2	136627117	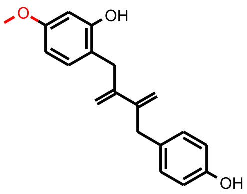 [*]OC	0.173

FCFP_2	1036089772	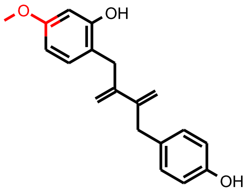 [*]:[c](:[*])OC	0.0749
FCFP_2	332760439	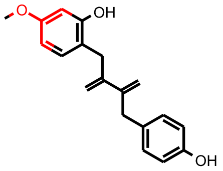 [*]O[c](:[cH]:[*]):[c]([*]):[*]	0.0611
**Top Features for negative contribution**			
**Fingerprint**	**Bit/Smiles**	**Feature Structure**	**Score**
FCFP_2	7	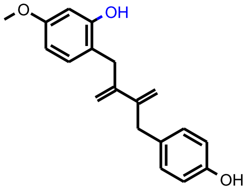 [*]O	-0.214
FCFP_2	-549108873	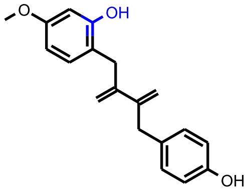 [*]:[c](:[*])O	-0.127
FCFP_2	203677720	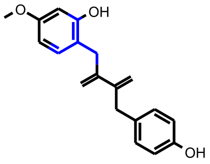 [*]C([*])[c](:[cH]:[*]):[c](:[*]):[*]	-0.0829

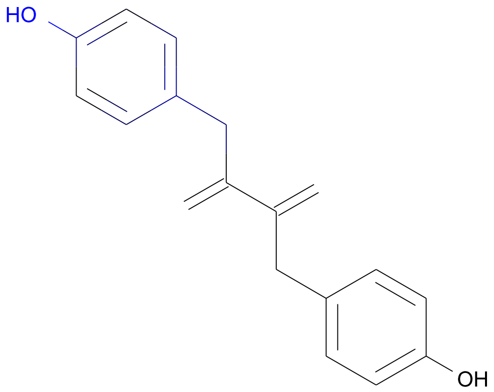

$C_{18}H_{18}O_2$
Molecular Weight: 266.33432
ALogP: 4.8
Rotatable Bonds: 5
Acceptors: 2
Donors: 2

Model Prediction

Prediction: 0.529
Unit: g/kg_body_weight
Mahalanobis Distance: 4.94
Mahalanobis Distance p-value: 0.964

Mahalanobis Distance: The Mahalanobis distance (MD) is a generalization of the Euclidean distance that accounts for correlations among the X properties. It is calculated as the distance to the center of the training data. The larger the MD, the less trustworthy the prediction.
Mahalanobis Distance p-value: The p-value gives the fraction of training data with an MD greater than or equal to the one for the given sample, assuming normally distributed data. The smaller the p-value, the less trustworthy the prediciton. For highly non-normal X properties (e.g., fingerprints), the MD p-value is wildly inaccurate.

Structural Similar Compounds

Name	BISPHENOL	BUTYLATED HYDROXYTOLUENE	EUGENOL
Structure	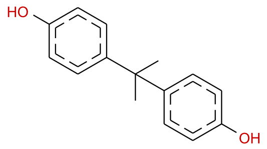	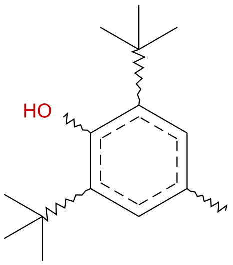	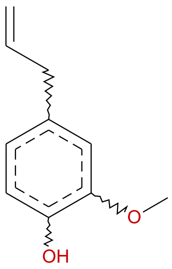
Actual Endpoint (-log C)	3.40425	2.91176	2.78402
Predicted Endpoint (-log C)	2.7079	3.0237	2.98617
Distance	0.408	0.580	0.593
Reference	NCI/NTP TR-215	NCI/NTP TR-150	NCI/NTP TR-223

Model Applicability

Unknown features are fingerprint features in the query molecule, but not found or appearing too infrequently in the training set.

1. All properties and OPS components are within expected ranges.

Feature Contribution

Top Features for negative contribution			
**Fingerprint**	**Bit/Smiles**	**Feature Structure**	**Score**
FCFP_2	7	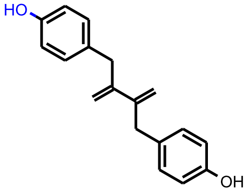 [*]O	-0.214

FCFP_2	-549108873	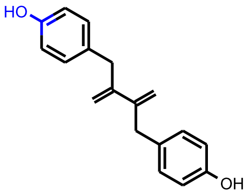 [*]:[c](:[*])O	-0.127
FCFP_2	203677720	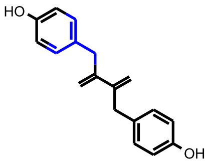 [*]C([*])[c](:[cH]:[*]):[c](:[*]):[*]	-0.0829

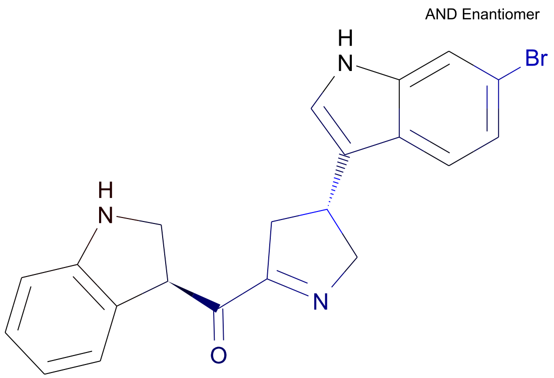

$C_{21}H_{18}BrN_3O$

Molecular Weight: 408.29112

ALogP: 3.919

Rotatable Bonds: 3

Acceptors: 3

Donors: 2

Model Prediction

Prediction: 0.198

Unit: g/kg_body_weight

Mahalanobis Distance: 10.4

Mahalanobis Distance p-value: 8.73e-006

Mahalanobis Distance: The Mahalanobis distance (MD) is a generalization of the Euclidean distance that accounts for correlations among the X properties. It is calculated as the distance to the center of the training data. The larger the MD, the less trustworthy the prediction.

Mahalanobis Distance p-value: The p-value gives the fraction of training data with an MD greater than or equal to the one for the given sample, assuming normally distributed data. The smaller the p-value, the less trustworthy the prediciton. For highly non-normal X properties (e.g., fingerprints), the MD p-value is wildly inaccurate.

Structural Similar Compounds

Name	OXAZEPAM	PHENOLPHTHALEIN	1-AMINO-2,4-DIBROMOANTHRAQUINONE
Structure	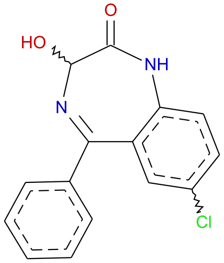	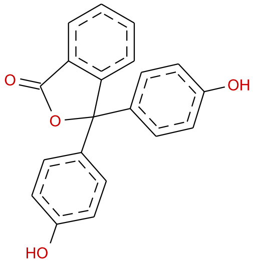	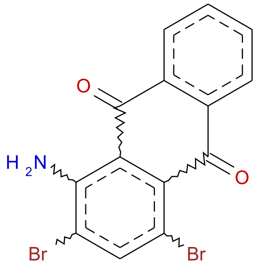
Actual Endpoint (-log C)	3.05262	2.20184	2.82966
Predicted Endpoint (-log C)	3.13073	2.8857	3.92444
Distance	0.588	0.599	0.603
Reference	NCI/NTP TR-468	NCI/NTP TR-465	NCI/NTP TR-383

Model Applicability

Unknown features are fingerprint features in the query molecule, but not found or appearing too infrequently in the training set.

1. All properties and OPS components are within expected ranges.

Feature Contribution

Top features for positive contribution			
Fingerprint	Bit/Smiles	Feature Structure	Score
FCFP_2	3	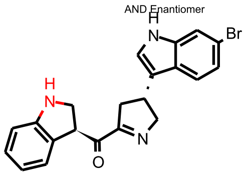 [*]N[*]	0.0737

FCFP_2	590925877	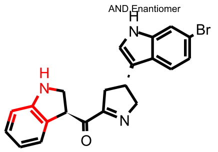<br>[*]:[cH]:[c]1N[*][*][c]:1:[*]	0.00762
FCFP_2	1294255210	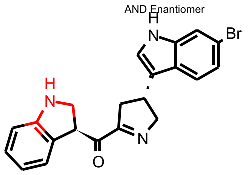<br>[*]:[c]1:[*][*]CN1	0.00319

Top Features for negative contribution

Fingerprint	Bit/Smiles	Feature Structure	Score
FCFP_2	71476542	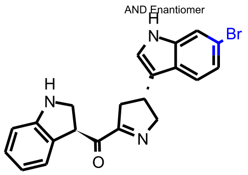<br>[*]:[c](:[*])Br	-0.134
FCFP_2	-1272798659	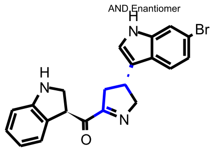<br>[*][C@H]1[*][*]CC1	-0.111
FCFP_2	1872154524	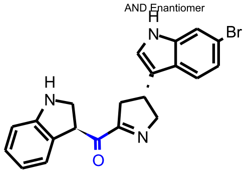<br>[*]C(=O)[*]	-0.105

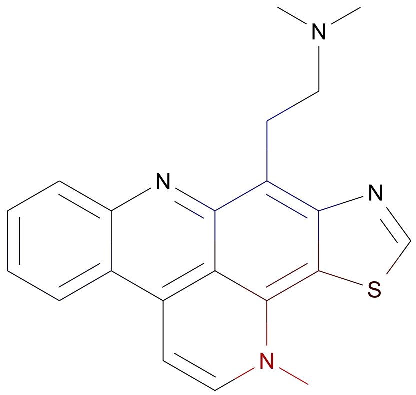

$C_{21}H_{20}N_4S$

Molecular Weight: 360.4753

ALogP: 3.682

Rotatable Bonds: 3

Acceptors: 4

Donors: 0

Model Prediction

Prediction: 0.0312

Unit: g/kg_body_weight

Mahalanobis Distance: 8.07

Mahalanobis Distance p-value: 0.0204

Mahalanobis Distance: The Mahalanobis distance (MD) is a generalization of the Euclidean distance that accounts for correlations among the X properties. It is calculated as the distance to the center of the training data. The larger the MD, the less trustworthy the prediction.

Mahalanobis Distance p-value: The p-value gives the fraction of training data with an MD greater than or equal to the one for the given sample, assuming normally distributed data. The smaller the p-value, the less trustworthy the prediciton. For highly non-normal X properties (e.g., fingerprints), the MD p-value is wildly inaccurate.

Structural Similar Compounds

Name	D&C YELLOW NO. 11	PYRILAMINE	C.I. SOLVENT YELLOW 14
Structure	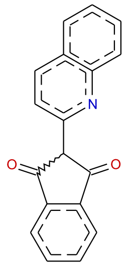	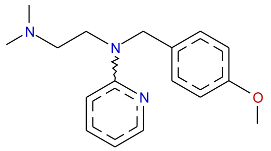	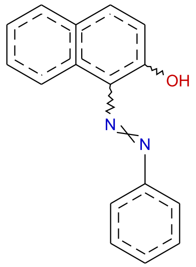
Actual Endpoint (-log C)	4.03869	3.32511	4.04277
Predicted Endpoint (-log C)	3.54593	3.65163	2.8989
Distance	0.609	0.750	0.751
Reference	NCI/NTP TR-463	NCI/NTP Report 10, Nov. 1987	NCI/NTP TR-226

Model Applicability

Unknown features are fingerprint features in the query molecule, but not found or appearing too infrequently in the training set.

1. All properties and OPS components are within expected ranges.
2. Unknown FCFP_2 feature: -124685461: [*]1:[*]:s:c:n:1

Feature Contribution

Top features for positive contribution

Fingerprint	Bit/Smiles	Feature Structure	Score
FCFP_2	136627117	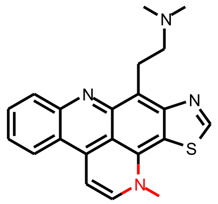 [*]OC	0.173

FCFP_2	332760439	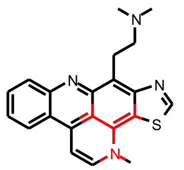 [*]O[c](:[cH]:[*]):[c]([*]):[*]	0.0611
FCFP_2	17	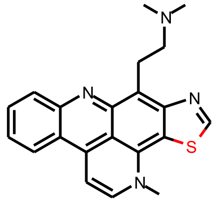 [*]:n:[*]	0.0441
**Top Features for negative contribution**			
**Fingerprint**	**Bit/Smiles**	**Feature Structure**	**Score**
FCFP_2	203677720	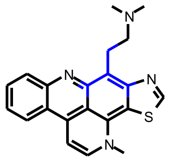 [*]C([*])[c](:[cH]:[*]):[c](:[*]):[*]	-0.0829
FCFP_2	1	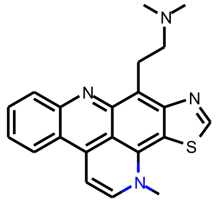 [*]O[*]	-0.0796
FCFP_2	16	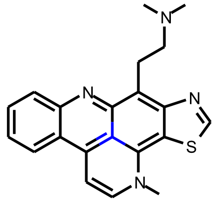 [*]:[cH]:[*]	-0.0512

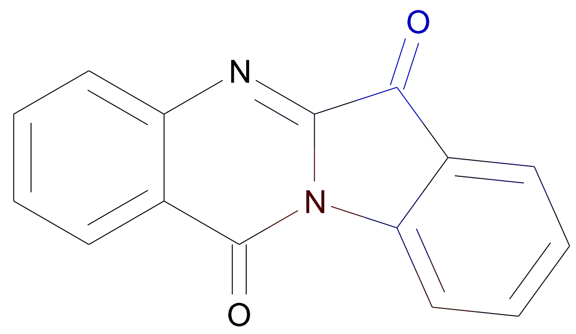

$C_{15}H_8N_2O_2$
Molecular Weight: 248.23621
ALogP: 2.331
Rotatable Bonds: 0
Acceptors: 3
Donors: 0

Model Prediction

Prediction: 0.0496
Unit: g/kg_body_weight
Mahalanobis Distance: 7.23
Mahalanobis Distance p-value: 0.13

Mahalanobis Distance: The Mahalanobis distance (MD) is a generalization of the Euclidean distance that accounts for correlations among the X properties. It is calculated as the distance to the center of the training data. The larger the MD, the less trustworthy the prediction.
Mahalanobis Distance p-value: The p-value gives the fraction of training data with an MD greater than or equal to the one for the given sample, assuming normally distributed data. The smaller the p-value, the less trustworthy the prediciton. For highly non-normal X properties (e.g., fingerprints), the MD p-value is wildly inaccurate.

Structural Similar Compounds

Name	1-NITRONAPHTHALENE	MICHLER'S KETONE	PHTHALIC ANHYDRIDE
Structure	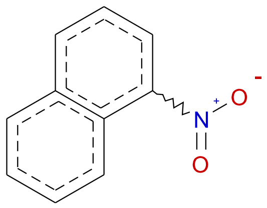	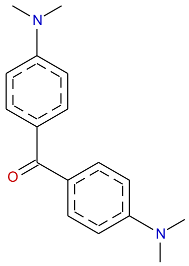	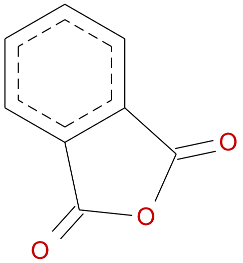
Actual Endpoint (-log C)	3.32999	4.07654	2.3413
Predicted Endpoint (-log C)	3.27306	3.57549	3.37248
Distance	0.453	0.510	0.515
Reference	NCI/NTP TR-64	NCI/NTP TR-181	NCI/NTP TR-159

Model Applicability

Unknown features are fingerprint features in the query molecule, but not found or appearing too infrequently in the training set.

1. All properties and OPS components are within expected ranges.

Feature Contribution

Top features for positive contribution

Fingerprint	Bit/Smiles	Feature Structure	Score
FCFP_2	332760439	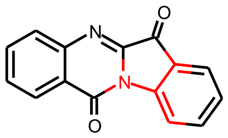 [*]O[c](:[cH]:[*]):[c]([*]):[*]	0.0611

FCFP_2	565998553	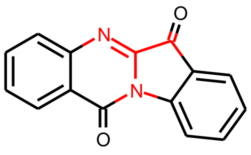 [*][C@@H]1[*][*]OC1=O	0.00813
**Top Features for negative contribution**			
**Fingerprint**	**Bit/Smiles**	**Feature Structure**	**Score**
FCFP_2	1872154524	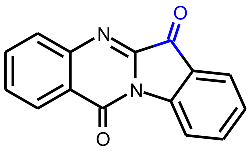 [*]C(=O)[*]	-0.105
FCFP_2	203677720	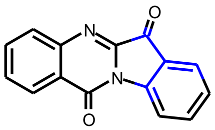 [*]C([*])[c](:[cH]:[*<br>]):[c](:[*]):[*]	-0.0829
FCFP_2	1	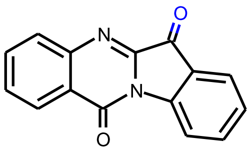 [*]O[*]	-0.0796

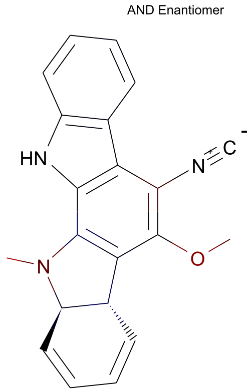

$C_{21}H_{17}N_3O$

Molecular Weight: 327.37918

ALogP: 4.078

Rotatable Bonds: 1

Acceptors: 2

Donors: 1

Model Prediction

Prediction: 0.0305

Unit: g/kg_body_weight

Mahalanobis Distance: 7.4

Mahalanobis Distance p-value: 0.095

Mahalanobis Distance: The Mahalanobis distance (MD) is a generalization of the Euclidean distance that accounts for correlations among the X properties. It is calculated as the distance to the center of the training data. The larger the MD, the less trustworthy the prediction.
Mahalanobis Distance p-value: The p-value gives the fraction of training data with an MD greater than or equal to the one for the given sample, assuming normally distributed data. The smaller the p-value, the less trustworthy the prediciton. For highly non-normal X properties (e.g., fingerprints), the MD p-value is wildly inaccurate.

Structural Similar Compounds

Name	D&C YELLOW NO. 11	C.I. SOLVENT YELLOW 14	PHENOLPHTHALEIN
Structure	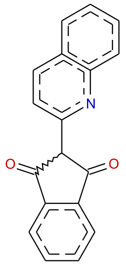	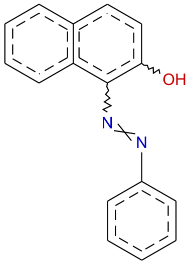	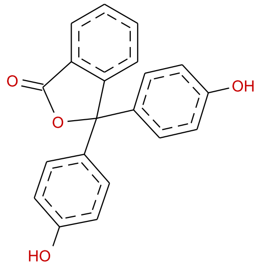
Actual Endpoint (-log C)	4.03869	4.04277	2.20184
Predicted Endpoint (-log C)	3.54593	2.8989	2.8857
Distance	0.572	0.586	0.666
Reference	NCI/NTP TR-463	NCI/NTP TR-226	NCI/NTP TR-465

Model Applicability

Unknown features are fingerprint features in the query molecule, but not found or appearing too infrequently in the training set.

1. All properties and OPS components are within expected ranges.
2. Unknown FCFP_2 feature: 8: [*][N+]#[*]
3. Unknown FCFP_2 feature: 4: [*]#[C-]
4. Unknown FCFP_2 feature: -828984032: [*][c](:[*]):[c]([N+]#[*]):[c](:[*]):[*]
5. Unknown FCFP_2 feature: 1934974835: [*]:[c](:[*])[N+]#[C-]
6. Unknown FCFP_2 feature: -1487147388: [*][N+]#[C-]

Feature Contribution

Top features for positive contribution			
**Fingerprint**	**Bit/Smiles**	**Feature Structure**	**Score**
FCFP_2	136627117	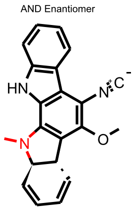 [*]OC	0.173

FCFP_2	1036089772	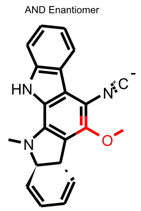 [*]:[c](:[*])OC	0.0749
FCFP_2	332760439	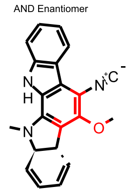 [*]O[c](:[cH]:[*]):[c]([*]):[*]	0.0611
**Top Features for negative contribution**			
**Fingerprint**	**Bit/Smiles**	**Feature Structure**	**Score**
FCFP_2	203677720	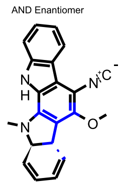 [*]C([*])[c](:[cH]:[*]):[c](:[*]):[*]	-0.0829
FCFP_2	1	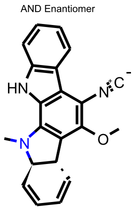 [*]O[*]	-0.0796
FCFP_2	16	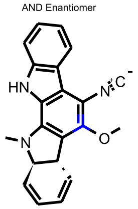 [*]:[cH]:[*]	-0.0512

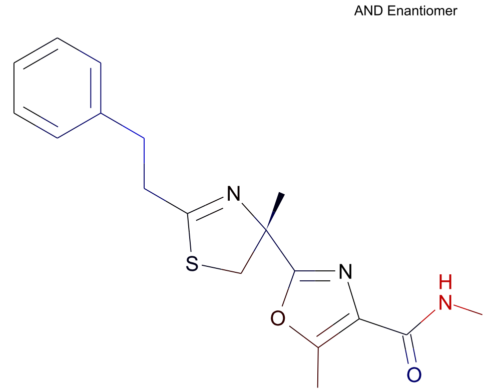

$C_{18}H_{21}N_3O_2S$
Molecular Weight: 343.44324
ALogP: 2.716
Rotatable Bonds: 5
Acceptors: 4
Donors: 1

Model Prediction

Prediction: 0.044
Unit: g/kg_body_weight
Mahalanobis Distance: 9.2
Mahalanobis Distance p-value: 0.000736

Mahalanobis Distance: The Mahalanobis distance (MD) is a generalization of the Euclidean distance that accounts for correlations among the X properties. It is calculated as the distance to the center of the training data. The larger the MD, the less trustworthy the prediction.
Mahalanobis Distance p-value: The p-value gives the fraction of training data with an MD greater than or equal to the one for the given sample, assuming normally distributed data. The smaller the p-value, the less trustworthy the prediciton. For highly non-normal X properties (e.g., fingerprints), the MD p-value is wildly inaccurate.

Structural Similar Compounds

Name	TOLAZAMIDE	CHLORBENZILATE	TOLBUTAMIDE
Structure	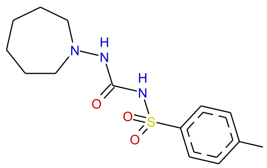	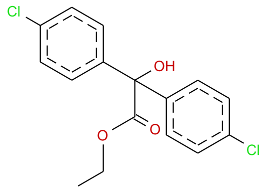	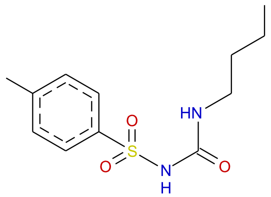
Actual Endpoint (-log C)	2.84011	3.38252	2.3985
Predicted Endpoint (-log C)	3.59315	3.27894	3.32272
Distance	0.587	0.588	0.589
Reference	NCI/NTP TR-051	NCI/NTP TR-75	NCI/NTP TR-031

Model Applicability

Unknown features are fingerprint features in the query molecule, but not found or appearing too infrequently in the training set.

1. OPS PC8 out of range. Value: 4.691. Training min, max, SD, explained variance: -3.8548, 3.9137, 1.331, 0.0400.
2. Unknown FCFP_2 feature: -836603894: [*]:[c](:[*])[C@]1(C)C[*][*]=N1

Feature Contribution

Top features for positive contribution			
**Fingerprint**	**Bit/Smiles**	**Feature Structure**	**Score**
FCFP_2	-885550502	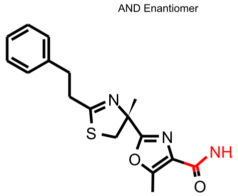 [*]C(=[*])NC	0.115

FCFP_2	-1143715940	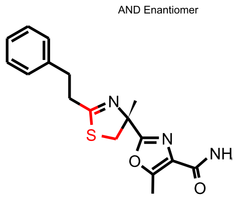 [*]=C1[*][*]CO1	0.095
FCFP_2	3	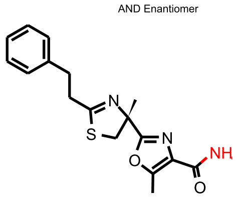 [*]N[*]	0.0737
**Top Features for negative contribution**			
**Fingerprint**	**Bit/Smiles**	**Feature Structure**	**Score**
FCFP_2	-1272798659	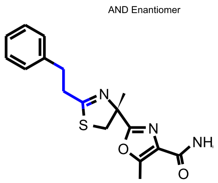 [*][C@H]1[*][*]CC1	-0.111
FCFP_2	1872154524	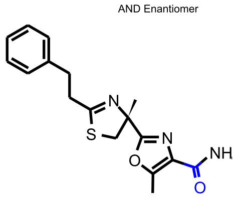 [*]C(=O)[*]	-0.105
FCFP_2	203677720	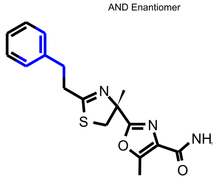 [*]C([*])[c](:[cH]:[*]):[c](:[*]):[*]	-0.0829

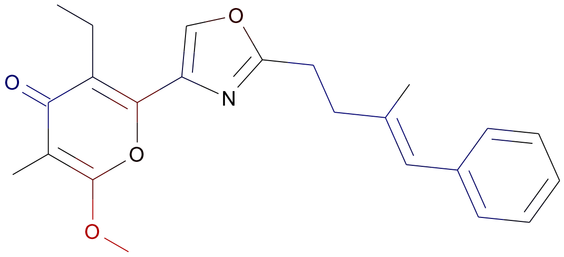

$C_{23}H_{25}NO_4$
Molecular Weight: 379.4489
ALogP: 5.22
Rotatable Bonds: 7
Acceptors: 4
Donors: 0

Model Prediction

Prediction: 0.0509
Unit: g/kg_body_weight
Mahalanobis Distance: 8.22
Mahalanobis Distance p-value: 0.0138

Mahalanobis Distance: The Mahalanobis distance (MD) is a generalization of the Euclidean distance that accounts for correlations among the X properties. It is calculated as the distance to the center of the training data. The larger the MD, the less trustworthy the prediction.
Mahalanobis Distance p-value: The p-value gives the fraction of training data with an MD greater than or equal to the one for the given sample, assuming normally distributed data. The smaller the p-value, the less trustworthy the prediciton. For highly non-normal X properties (e.g., fingerprints), the MD p-value is wildly inaccurate.

Structural Similar Compounds

Name	BUTYL BENZYL PHTHALATE	PIPERONYL SULFOXIDE	CHLORBENZILATE
Structure	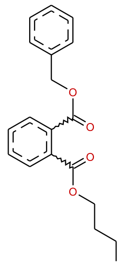	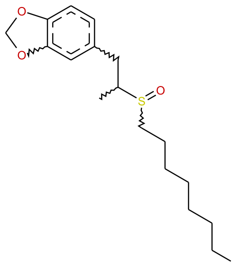	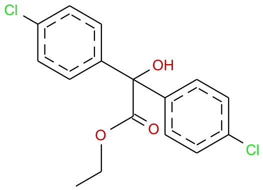
Actual Endpoint (-log C)	2.79569	3.38085	3.38252
Predicted Endpoint (-log C)	3.18498	3.46483	3.27894
Distance	0.489	0.597	0.606
Reference	NCI/NTP TR-458	NCI/NTP TR-124	NCI/NTP TR-75

Model Applicability

Unknown features are fingerprint features in the query molecule, but not found or appearing too infrequently in the training set.

1. All properties and OPS components are within expected ranges.
2. Unknown FCFP_2 feature: -2115241127: [*]OC(=C([*])[*])O[*]

Feature Contribution

Top features for positive contribution			
Fingerprint	Bit/Smiles	Feature Structure	Score
FCFP_2	136627117	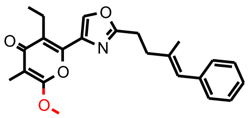 [*]OC	0.173

FCFP_2	-1143715940	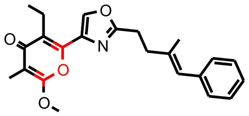 [*]=C1[*][*]CO1	0.095
FCFP_2	17	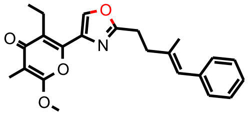 [*]:n:[*]	0.0441
**Top Features for negative contribution**			
**Fingerprint**	**Bit/Smiles**	**Feature Structure**	**Score**
FCFP_2	-1272798659	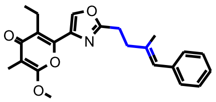 [*][C@H]1[*][*]CC1	-0.111
FCFP_2	1872154524	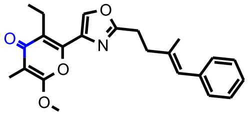 [*]C(=O)[*]	-0.105
FCFP_2	203677720	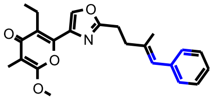 [*]C([*])[c](:[cH]:[*]):[c](:[*]):[*]	-0.0829

Remdesivir

TOPKAT_Rat_Maximum_Tolerated_Dose_Feed

AND Enantiomer

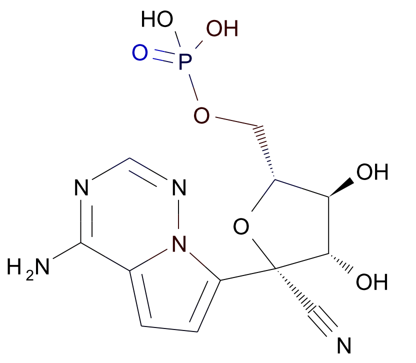

$C_{12}H_{14}N_5O_7P$

Molecular Weight: 371.24262

ALogP: -1.502

Rotatable Bonds: 4

Acceptors: 11

Donors: 5

Structural Similar Compounds

Name	4,4'-DIAMINO-2,2'-STILBENEDISULFONIC ACID.2NaSALT	OXYTETRACYCLINE	50%1,4,5,8-TETRAAMINOANTHRAQUIONONE + DERIVATIVES
Structure	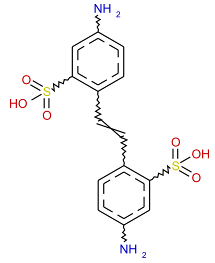	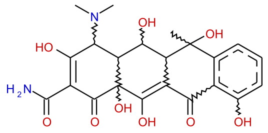	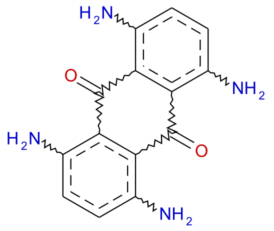
Actual Endpoint (-log C)	2.50759	2.36214	3.0764
Predicted Endpoint (-log C)	3.26068	2.77834	3.08142
Distance	0.743	0.818	0.989
Reference	NCI/NTP TR-412	NCI/NTP TR-315	NCI/NTP TR-299

Model Prediction

Prediction: 0.235

Unit: g/kg_body_weight

Mahalanobis Distance: 9.52

Mahalanobis Distance p-value: 0.000247

Mahalanobis Distance: The Mahalanobis distance (MD) is a generalization of the Euclidean distance that accounts for correlations among the X properties. It is calculated as the distance to the center of the training data. The larger the MD, the less trustworthy the prediction.
Mahalanobis Distance p-value: The p-value gives the fraction of training data with an MD greater than or equal to the one for the given sample, assuming normally distributed data. The smaller the p-value, the less trustworthy the prediciton. For highly non-normal X properties (e.g., fingerprints), the MD p-value is wildly inaccurate.

Model Applicability

Unknown features are fingerprint features in the query molecule, but not found or appearing too infrequently in the training set.

1. Molecular_PolarSurfaceArea out of range. Value: 206.26. Training min, max, mean, SD: 0, 201.84, 63.052, 40.7.
2. Unknown FCFP_2 feature: 472180098: [*]OP(=O)(O)O
3. Unknown FCFP_2 feature: -836603894: [*]:[c](:[*])[C@]1(C)C[*][*]=N1
4. Unknown FCFP_2 feature: -1277879912: [*]C([*])([*])C#N
5. Unknown FCFP_2 feature: -332197802: [*][c]1:[*]:[*]:[c](:[*]):n:1:n:[*]
6. Unknown FCFP_2 feature: -124685461: [*]1:[*]:s:c:n:1

Feature Contribution

Top features for positive contribution

Fingerprint	Bit/Smiles	Feature Structure	Score

FCFP_2	-1143715940	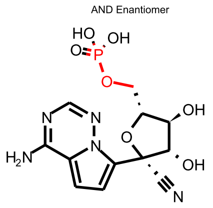 [*]=C1[*][*]CO1	0.095
FCFP_2	3	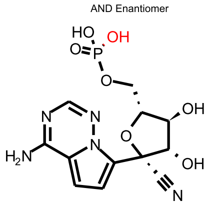 [*]N[*]	0.0737
FCFP_2	17	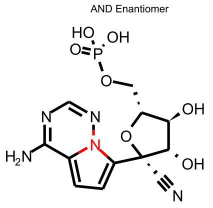 [*]:n:[*]	0.0441

Top Features for negative contribution

**Fingerprint**	**Bit/Smiles**	**Feature Structure**	**Score**
FCFP_2	1872154524	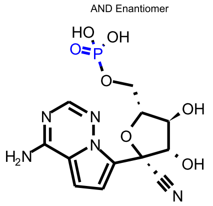 [*]C(=O)[*]	-0.105
FCFP_2	1	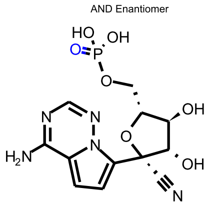 [*]O[*]	-0.0796

FCFP_2	16	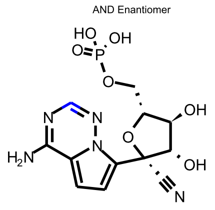<br>[*]:[cH]:[*]	-0.0512

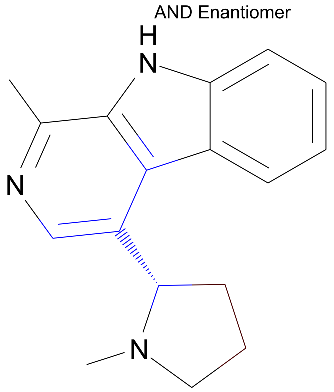

$C_{17}H_{19}N_3$

Molecular Weight: 265.35286

ALogP: 3.018

Rotatable Bonds: 1

Acceptors: 2

Donors: 1

Model Prediction

Prediction: 0.116

Unit: g/kg_body_weight

Mahalanobis Distance: 9.97

Mahalanobis Distance p-value: 6.16e-006

Mahalanobis Distance: The Mahalanobis distance (MD) is a generalization of the Euclidean distance that accounts for correlations among the X properties. It is calculated as the distance to the center of the training data. The larger the MD, the less trustworthy the prediction.

Mahalanobis Distance p-value: The p-value gives the fraction of training data with an MD greater than or equal to the one for the given sample, assuming normally distributed data. The smaller the p-value, the less trustworthy the prediciton. For highly non-normal X properties (e.g., fingerprints), the MD p-value is wildly inaccurate.

Structural Similar Compounds

Name	CHLORPHENIRAMINE MALEATE	8-METHOXYPSORALEN	o-BENZYL-p-CHLOROPHENOL
Structure	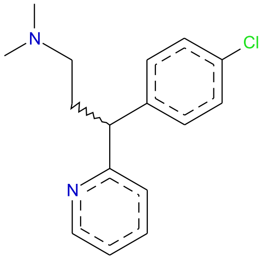	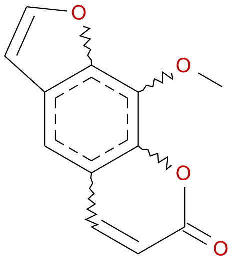	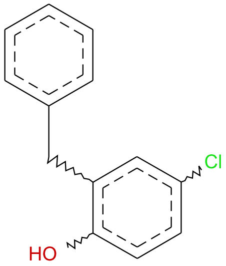
Actual Endpoint (-log C)	3.96188	3.45978	3.26063
Predicted Endpoint (-log C)	3.83117	4.14745	3.64448
Distance	0.658	0.753	0.759
Reference	NCI/NTP TR-317	NCI/NTP TR-359	NCI/NTP TR-424

Model Applicability

Unknown features are fingerprint features in the query molecule, but not found or appearing too infrequently in the training set.

1. Num_AromaticRings out of range. Value: 3. Training min, max, mean, SD: 0, 2, 0.5625, 0.693.
2. Unknown FCFP_2 feature: 19: [*]:[nH]:[*]
3. Unknown FCFP_2 feature: 307448885: [*]:[c]1:[*]:[*]:[nH]:[c]:1:c:[*]
4. Unknown FCFP_2 feature: 2005402822: [*]:[c]1:[*]:[*]:[c](:[*]):[nH]:1
5. Unknown FCFP_2 feature: 1871533475: [*]N1[*][*]C[C@H]1[c](:[*]):[*]

Feature Contribution

Top features for positive contribution			
Fingerprint	Bit/Smiles	Feature Structure	Score
FCFP_2	-1272798659	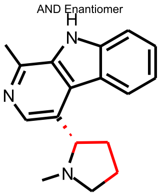 [*][C@H]1[*][*]CC1	0.0703

Top Features for negative contribution			
Fingerprint	Bit/Smiles	Feature Structure	Score
FCFP_2	203677720	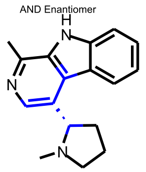 [*]C([*])[c](:[cH]:[*]):[c](:[*]):[*]	-0.406
FCFP_2	0	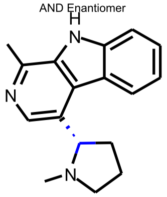 [*]C([*])[*]	-0.29

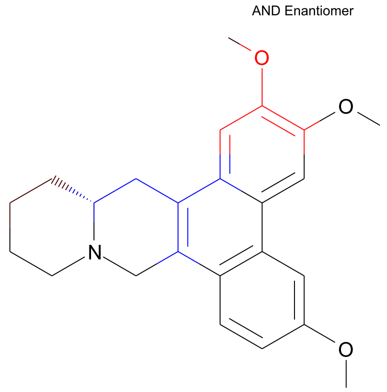

$C_{24}H_{27}NO_3$
Molecular Weight: 377.47608
ALogP: 4.691
Rotatable Bonds: 3
Acceptors: 4
Donors: 0

Model Prediction

Prediction: 0.000356
Unit: g/kg_body_weight
Mahalanobis Distance: 10.5
Mahalanobis Distance p-value: 1.11e-006

Mahalanobis Distance: The Mahalanobis distance (MD) is a generalization of the Euclidean distance that accounts for correlations among the X properties. It is calculated as the distance to the center of the training data. The larger the MD, the less trustworthy the prediction.
Mahalanobis Distance p-value: The p-value gives the fraction of training data with an MD greater than or equal to the one for the given sample, assuming normally distributed data. The smaller the p-value, the less trustworthy the prediciton. For highly non-normal X properties (e.g., fingerprints), the MD p-value is wildly inaccurate.

Structural Similar Compounds

Name	PROMETHAZINE.HCL	PHENYLBUTAZONE	CHLORPHENIRAMINE MALEATE
Structure	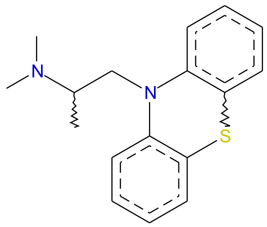	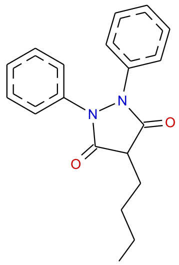	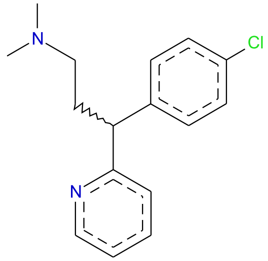
Actual Endpoint (-log C)	3.93152	3.48909	3.96188
Predicted Endpoint (-log C)	4.72433	3.17333	3.83117
Distance	0.651	0.768	0.780
Reference	NCI/NTP TR-425	NCI/NTP TR-367	NCI/NTP TR-317

Model Applicability

Unknown features are fingerprint features in the query molecule, but not found or appearing too infrequently in the training set.

1. Num_AromaticRings out of range. Value: 3. Training min, max, mean, SD: 0, 2, 0.5625, 0.693.
2. OPS PC6 out of range. Value: -3.389. Training min, max, SD, explained variance: -2.4321, 2.9885, 1.256, 0.0488.
3. Unknown FCFP_2 feature: 906798516: [*]N([*])C[c](:[*]):[*]

Feature Contribution

Top features for positive contribution

Fingerprint	Bit/Smiles	Feature Structure	Score
FCFP_2	332760439	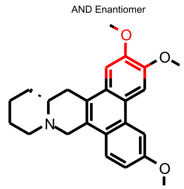 [*]O[c](:[cH]:[*]):[c]([*]):[*]	0.672

FCFP_2	1	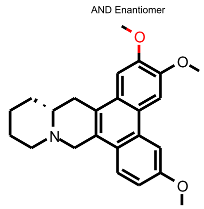 [*]O[*]	0.511
FCFP_2	-1272798659	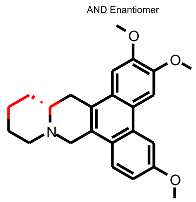 [*][C@H]1[*][*]CC1	0.0703
**Top Features for negative contribution**			
**Fingerprint**	**Bit/Smiles**	**Feature Structure**	**Score**
FCFP_2	203677720	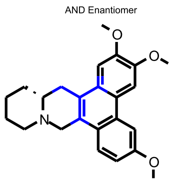 [*]C([*])[c](:[cH]:[*]):[c](:[*]):[*]	-0.406
FCFP_2	0	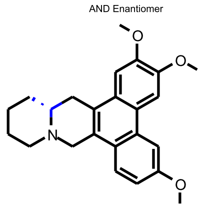 [*]C([*])[*]	-0.29

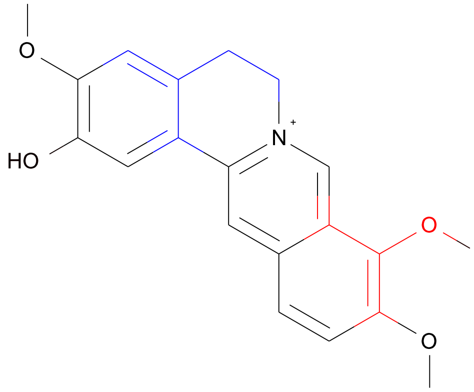

$C_{20}H_{20}NO_4$
Molecular Weight: 338.3771
ALogP: 3.936
Rotatable Bonds: 3
Acceptors: 4
Donors: 1

Structural Similar Compounds

Name	8-METHOXYPSORALEN	PROMETHAZINE.HCL	PHENYLBUTAZONE
Structure	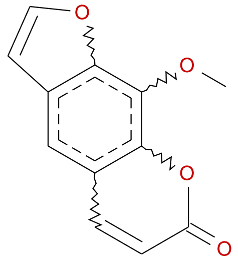	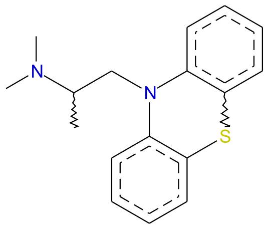	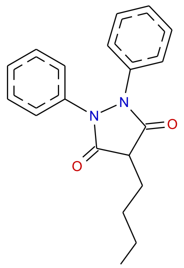
Actual Endpoint (-log C)	3.45978	3.93152	3.48909
Predicted Endpoint (-log C)	4.14745	4.72433	3.17333
Distance	0.826	0.826	0.834
Reference	NCI/NTP TR-359	NCI/NTP TR-425	NCI/NTP TR-367

Model Prediction

Prediction: 0.00121
Unit: g/kg_body_weight
Mahalanobis Distance: 8.72
Mahalanobis Distance p-value: 0.000277

Mahalanobis Distance: The Mahalanobis distance (MD) is a generalization of the Euclidean distance that accounts for correlations among the X properties. It is calculated as the distance to the center of the training data. The larger the MD, the less trustworthy the prediction.
Mahalanobis Distance p-value: The p-value gives the fraction of training data with an MD greater than or equal to the one for the given sample, assuming normally distributed data. The smaller the p-value, the less trustworthy the prediciton. For highly non-normal X properties (e.g., fingerprints), the MD p-value is wildly inaccurate.

Model Applicability

Unknown features are fingerprint features in the query molecule, but not found or appearing too infrequently in the training set.

1. Num_AromaticRings out of range. Value: 3. Training min, max, mean, SD: 0, 2, 0.5625, 0.693.
2. OPS PC6 out of range. Value: -2.5473. Training min, max, SD, explained variance: -2.4321, 2.9885, 1.256, 0.0488.
3. OPS PC9 out of range. Value: 4.7528. Training min, max, SD, explained variance: -2.7086, 2.9267, 1.019, 0.0321.
4. Unknown FCFP_2 feature: 24: [*][n+](:[*]):[*]
5. Unknown FCFP_2 feature: 414371600: [*]C[n+](:[c]([*]):[*]):c:[*]
6. Unknown FCFP_2 feature: -150573739: [*]CC[n+](:[*]):[*]
7. Unknown FCFP_2 feature: -1861407456: [*][n+](:[*]):[c]([c](:[*]):[*]):c:[*]
8. Unknown FCFP_2 feature: -1861645784: [*][c](:[*]):[c]([c](:[*]):[*]):c:[*]
9. Unknown FCFP_2 feature: 1618392993: [*][n+](:[*]):c:[c](:[*]):[*]

Feature Contribution

Top features for positive contribution

Fingerprint	Bit/Smiles	Feature Structure	Score

FCFP_2	332760439	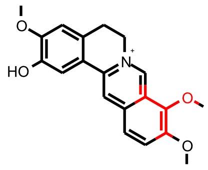 [*]O[c](:[cH]:[*]):[c]([*]):[*]	0.672
FCFP_2	1	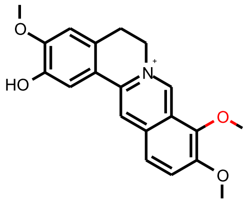 [*]O[*]	0.511
FCFP_2	136627117	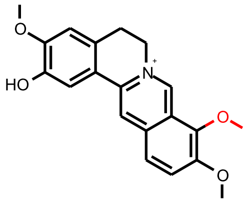 [*]OC	0.0304
**Top Features for negative contribution**			
**Fingerprint**	**Bit/Smiles**	**Feature Structure**	**Score**
FCFP_2	203677720	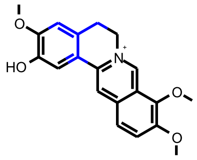 [*]C([*])[c](:[cH]:[*]):[c](:[*]):[*]	-0.406
FCFP_2	0	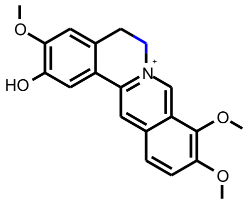 [*]C([*])[*]	-0.29

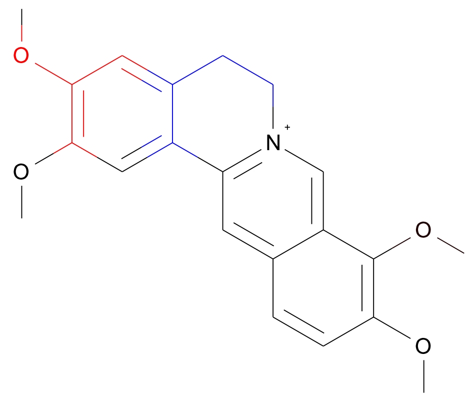

$C_{21}H_{22}NO_4$
Molecular Weight: 352.40368
ALogP: 4.161
Rotatable Bonds: 4
Acceptors: 4
Donors: 0

Model Prediction

Prediction: 0.00123
Unit: g/kg_body_weight
Mahalanobis Distance: 8.22
Mahalanobis Distance p-value: 0.00114

Mahalanobis Distance: The Mahalanobis distance (MD) is a generalization of the Euclidean distance that accounts for correlations among the X properties. It is calculated as the distance to the center of the training data. The larger the MD, the less trustworthy the prediction.
Mahalanobis Distance p-value: The p-value gives the fraction of training data with an MD greater than or equal to the one for the given sample, assuming normally distributed data. The smaller the p-value, the less trustworthy the prediciton. For highly non-normal X properties (e.g., fingerprints), the MD p-value is wildly inaccurate.

Structural Similar Compounds

Name	PROMETHAZINE.HCL	PHENYLBUTAZONE	8-METHOXYPSORALEN
Structure	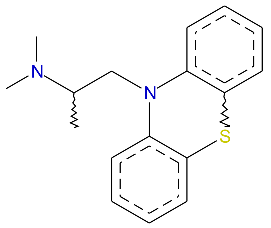	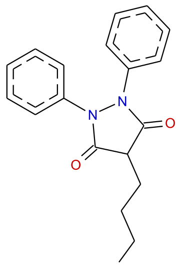	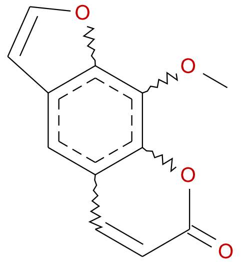
Actual Endpoint (-log C)	3.93152	3.48909	3.45978
Predicted Endpoint (-log C)	4.72433	3.17333	4.14745
Distance	0.726	0.741	0.808
Reference	NCI/NTP TR-425	NCI/NTP TR-367	NCI/NTP TR-359

Model Applicability

Unknown features are fingerprint features in the query molecule, but not found or appearing too infrequently in the training set.

1. Num_AromaticRings out of range. Value: 3. Training min, max, mean, SD: 0, 2, 0.5625, 0.693.
2. OPS PC6 out of range. Value: -3.4928. Training min, max, SD, explained variance: -2.4321, 2.9885, 1.256, 0.0488.
3. Unknown FCFP_2 feature: 24: [*][n+](:[*]):[*]
4. Unknown FCFP_2 feature: 414371600: [*]C[n+](:[c]([*]):[*]):c:[*]
5. Unknown FCFP_2 feature: -150573739: [*]CC[n+](:[*]):[*]
6. Unknown FCFP_2 feature: -1861407456: [*][n+](:[*]):[c]([c](:[*]):[*]):c:[*]
7. Unknown FCFP_2 feature: -1861645784: [*][c](:[*]):[c]([c](:[*]):[*]):c:[*]
8. Unknown FCFP_2 feature: 1618392993: [*][n+](:[*]):c:[c](:[*]):[*]

Feature Contribution

Top features for positive contribution

Fingerprint	Bit/Smiles	Feature Structure	Score

FCFP_2	332760439	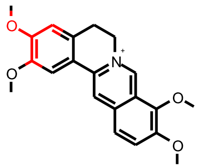 [*]O[c](:[cH]:[*]):[c]([*]):[*]	0.672
FCFP_2	1	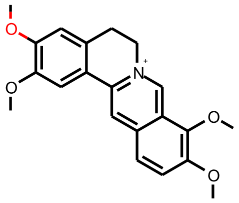 [*]O[*]	0.511
FCFP_2	136627117	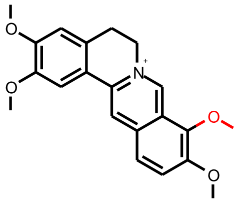 [*]OC	0.0304

Top Features for negative contribution

Fingerprint	Bit/Smiles	Feature Structure	Score
FCFP_2	203677720	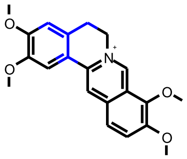 [*]C([*])[c](:[cH]:[*]):[c](:[*]):[*]	-0.406
FCFP_2	0	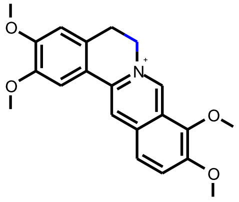 [*]C([*])[*]	-0.29

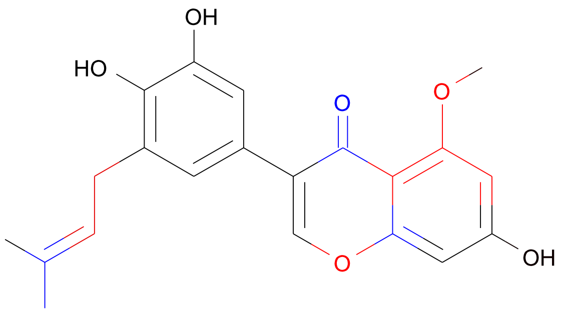

$C_{21}H_{20}O_6$
Molecular Weight: 368.3799
ALogP: 3.98
Rotatable Bonds: 4
Acceptors: 6
Donors: 3

Model Prediction

Prediction: 0.00098
Unit: g/kg_body_weight
Mahalanobis Distance: 10.8
Mahalanobis Distance p-value: 4.14e-007

Mahalanobis Distance: The Mahalanobis distance (MD) is a generalization of the Euclidean distance that accounts for correlations among the X properties. It is calculated as the distance to the center of the training data. The larger the MD, the less trustworthy the prediction.
Mahalanobis Distance p-value: The p-value gives the fraction of training data with an MD greater than or equal to the one for the given sample, assuming normally distributed data. The smaller the p-value, the less trustworthy the prediciton. For highly non-normal X properties (e.g., fingerprints), the MD p-value is wildly inaccurate.

Structural Similar Compounds

Name	OCHRATOXIN	PENICILLIN VK	SULFISOOXAZOLE
Structure	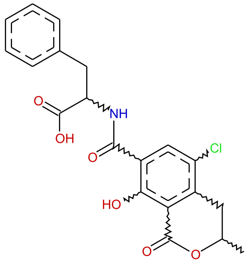	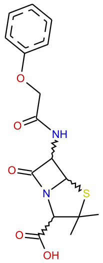	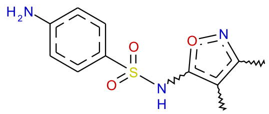
Actual Endpoint (-log C)	6.28396	2.54455	2.82494
Predicted Endpoint (-log C)	5.12358	3.9702	3.0705
Distance	0.500	0.858	0.905
Reference	NCI/NTP TR-358	NCI/NTP TR-336	NCI/NTP TR-138

Model Applicability

Unknown features are fingerprint features in the query molecule, but not found or appearing too infrequently in the training set.

1. OPS PC9 out of range. Value: 4.3596. Training min, max, SD, explained variance: -2.7086, 2.9267, 1.019, 0.0321.
2. Unknown FCFP_2 feature: -1549192822: [*]C(=[*])C(=O)[c](:[*]):[*]
3. Unknown FCFP_2 feature: -1678275541: [*]\C=C(/C(=[*])[*])\[c](:[*]):[*]

Feature Contribution

Top features for positive contribution

Fingerprint	Bit/Smiles	Feature Structure	Score
FCFP_2	332760439	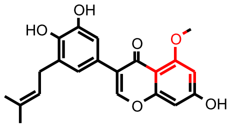 [*]O[c](:[cH]:[*]):[c]([*]):[*]	0.672

FCFP_2	1	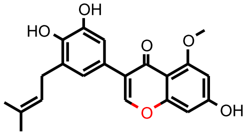 [*]O[*]	0.511
FCFP_2	451847724	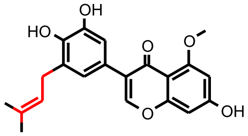 [*]CC=C([*])[*]	0.225
**Top Features for negative contribution**			
**Fingerprint**	**Bit/Smiles**	**Feature Structure**	**Score**
FCFP_2	136597326	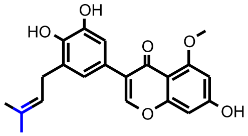 [*]C(=[*])C	-0.489
FCFP_2	203677720	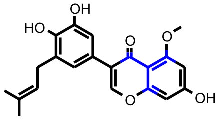 [*]C([*])[c](:[cH]:[*]):[c](:[*]):[*]	-0.406
FCFP_2	1872154524	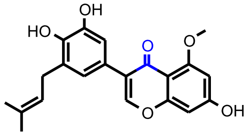 [*]C(=O)[*]	-0.307

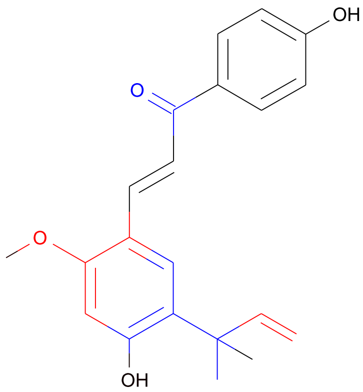

$C_{21}H_{22}O_4$
Molecular Weight: 338.39698
ALogP: 4.667
Rotatable Bonds: 6
Acceptors: 4
Donors: 2

Structural Similar Compounds

Name	1-TRANS-DELTA(9)-TETRAHYDROCANNABINOL	PROBENECID	4-HEXYLRESORCINOL
Structure	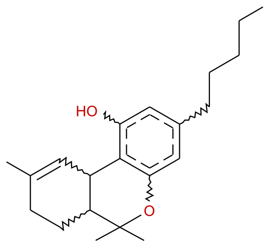	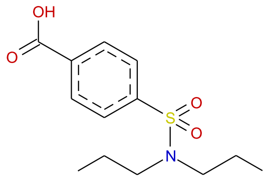	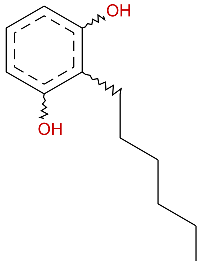
Actual Endpoint (-log C)	3.79861	2.85333	3.1915
Predicted Endpoint (-log C)	4.44032	2.4258	2.16134
Distance	0.783	0.793	0.793
Reference	NCI/NTP TR-446	NCI/NTP TR-395	NCI/NTP TR-330

Model Prediction

Prediction: 0.00459
Unit: g/kg_body_weight
Mahalanobis Distance: 8.96
Mahalanobis Distance p-value: 0.000136

Mahalanobis Distance: The Mahalanobis distance (MD) is a generalization of the Euclidean distance that accounts for correlations among the X properties. It is calculated as the distance to the center of the training data. The larger the MD, the less trustworthy the prediction.
Mahalanobis Distance p-value: The p-value gives the fraction of training data with an MD greater than or equal to the one for the given sample, assuming normally distributed data. The smaller the p-value, the less trustworthy the prediciton. For highly non-normal X properties (e.g., fingerprints), the MD p-value is wildly inaccurate.

Model Applicability

Unknown features are fingerprint features in the query molecule, but not found or appearing too infrequently in the training set.

1. OPS PC9 out of range. Value: 4.9865. Training min, max, SD, explained variance: -2.7086, 2.9267, 1.019, 0.0321.
2. Unknown FCFP_2 feature: -836633685: [*]=CC(C)(C)[c](:[*]):[*]
3. Unknown FCFP_2 feature: -1549192822: [*]C(=[*])C(=O)[c](:[*]):[*]

Feature Contribution

Top features for positive contribution

Fingerprint	Bit/Smiles	Feature Structure	Score
FCFP_2	332760439	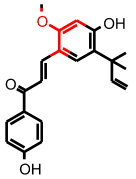 [*]O[c](:[cH]:[*]):[c]([*]):[*]	0.672

FCFP_2	129344189	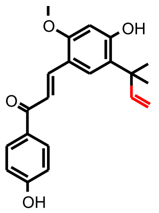 [*]C=C	0.519
FCFP_2	1	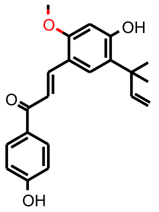 [*]O[*]	0.511
**Top Features for negative contribution**			
**Fingerprint**	**Bit/Smiles**	**Feature Structure**	**Score**
FCFP_2	136597326	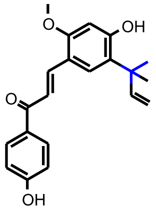 [*]C(=[*])C	-0.489
FCFP_2	203677720	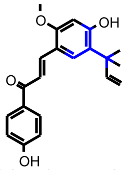 [*]C([*])[c](:[cH]:[*]):[c](:[*]):[*]	-0.406
FCFP_2	1872154524	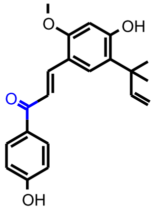 [*]C(=O)[*]	-0.307

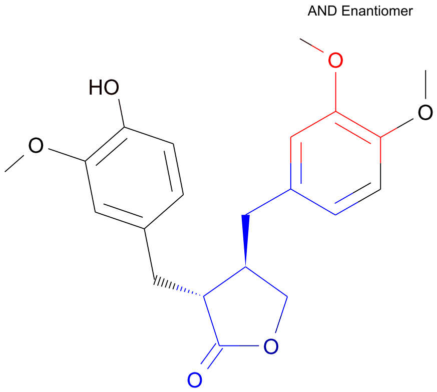

$C_{21}H_{24}O_6$
Molecular Weight: 372.41166
ALogP: 3.743
Rotatable Bonds: 7
Acceptors: 6
Donors: 1

Structural Similar Compounds

Name	PROBENECID	DIALLYL PHTHALATE	PHENYLBUTAZONE
Structure	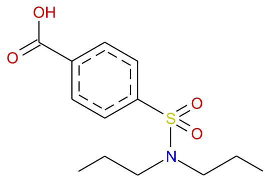	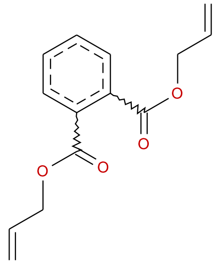	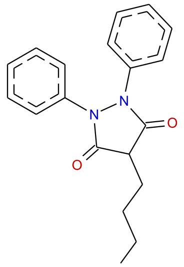
Actual Endpoint (-log C)	2.85333	3.3914	3.48909
Predicted Endpoint (-log C)	2.4258	3.50093	3.17333
Distance	0.792	0.811	0.866
Reference	NCI/NTP TR-395	NCI/NTP TR-284	NCI/NTP TR-367

Model Prediction

Prediction: 0.00518
Unit: g/kg_body_weight
Mahalanobis Distance: 8.32
Mahalanobis Distance p-value: 0.000858

Mahalanobis Distance: The Mahalanobis distance (MD) is a generalization of the Euclidean distance that accounts for correlations among the X properties. It is calculated as the distance to the center of the training data. The larger the MD, the less trustworthy the prediction.
Mahalanobis Distance p-value: The p-value gives the fraction of training data with an MD greater than or equal to the one for the given sample, assuming normally distributed data. The smaller the p-value, the less trustworthy the prediciton. For highly non-normal X properties (e.g., fingerprints), the MD p-value is wildly inaccurate.

Model Applicability

Unknown features are fingerprint features in the query molecule, but not found or appearing too infrequently in the training set.

1. OPS PC9 out of range. Value: 4.7288. Training min, max, SD, explained variance: -2.7086, 2.9267, 1.019, 0.0321.

Feature Contribution

Top features for positive contribution

Fingerprint	Bit/Smiles	Feature Structure	Score
FCFP_2	332760439	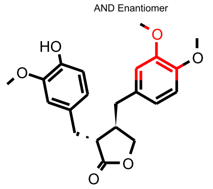 [*]O[c](:[cH]:[*]):[c]([*]):[*]	0.672

FCFP_2	1	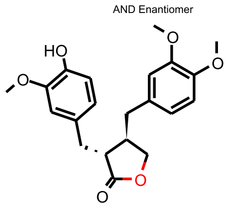 [*]O[*]	0.511
FCFP_2	136627117	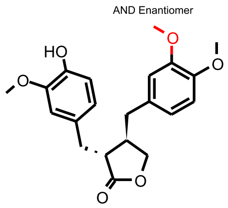 [*]OC	0.0304

Top Features for negative contribution

Fingerprint	Bit/Smiles	Feature Structure	Score
FCFP_2	203677720	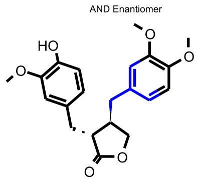 [*]C([*])[c](:[cH]:[*<br>]):[c](:[*]):[*]	-0.406
FCFP_2	565998553	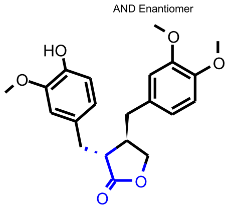 [*][C@@H]1[*][*]OC1=O	-0.348
FCFP_2	1872154524	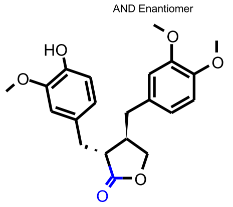 [*]C(=O)[*]	-0.307

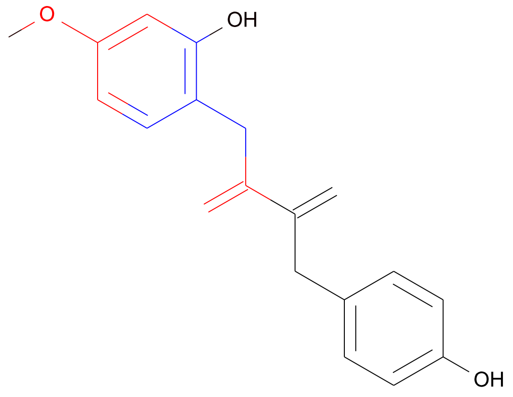

$C_{19}H_{20}O_3$
Molecular Weight: 296.3603
ALogP: 4.784
Rotatable Bonds: 6
Acceptors: 3
Donors: 2

Structural Similar Compounds

Name	4-HEXYLRESORCINOL	1-TRANS-DELTA(9)-TETRAHYDROCANNABINOL	o-BENZYL-p-CHLOROPHENOL
Structure	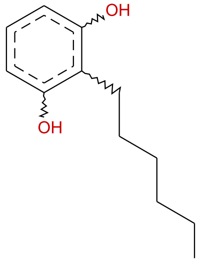	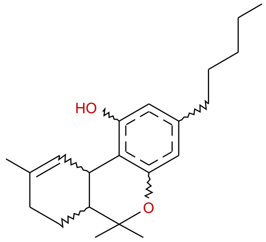	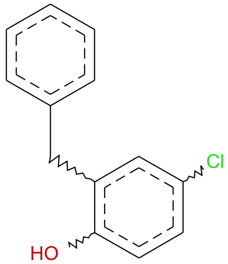
Actual Endpoint (-log C)	3.1915	3.79861	3.26063
Predicted Endpoint (-log C)	2.16134	4.44032	3.64448
Distance	0.618	0.633	0.787
Reference	NCI/NTP TR-330	NCI/NTP TR-446	NCI/NTP TR-424

Model Prediction

Prediction: 0.00292
Unit: g/kg_body_weight
Mahalanobis Distance: 9.32
Mahalanobis Distance p-value: 4.62e-005

Mahalanobis Distance: The Mahalanobis distance (MD) is a generalization of the Euclidean distance that accounts for correlations among the X properties. It is calculated as the distance to the center of the training data. The larger the MD, the less trustworthy the prediction.
Mahalanobis Distance p-value: The p-value gives the fraction of training data with an MD greater than or equal to the one for the given sample, assuming normally distributed data. The smaller the p-value, the less trustworthy the prediciton. For highly non-normal X properties (e.g., fingerprints), the MD p-value is wildly inaccurate.

Model Applicability

Unknown features are fingerprint features in the query molecule, but not found or appearing too infrequently in the training set.

1. OPS PC9 out of range. Value: 4.9452. Training min, max, SD, explained variance: -2.7086, 2.9267, 1.019, 0.0321.

Feature Contribution

Top features for positive contribution

Fingerprint	Bit/Smiles	Feature Structure	Score
FCFP_2	332760439	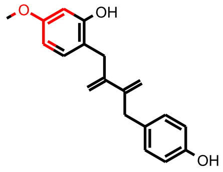 [*]O[c](:[cH]:[*]):[c]([*]):[*]	0.672

FCFP_2	129344189	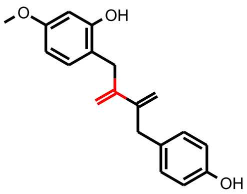 [*]C=C	0.519
FCFP_2	1	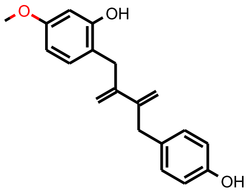 [*]O[*]	0.511

Top Features for negative contribution

Fingerprint	Bit/Smiles	Feature Structure	Score
FCFP_2	203677720	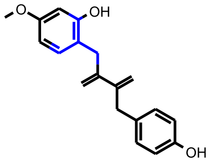 [*]C([*])[c](:[cH]:[*]):[c](:[*]):[*]	-0.406
FCFP_2	0	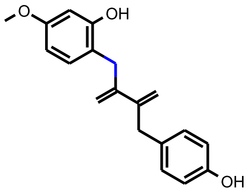 [*]C([*])[*]	-0.29

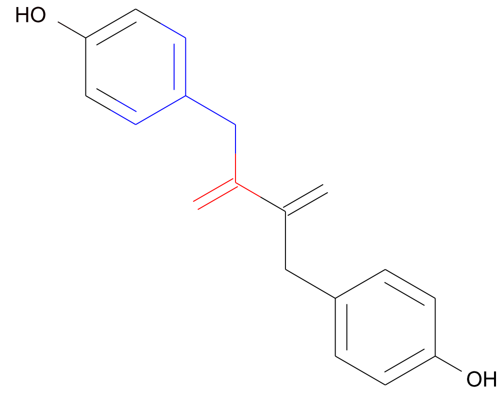

$C_{18}H_{18}O_2$
Molecular Weight: 266.33432
ALogP: 4.8
Rotatable Bonds: 5
Acceptors: 2
Donors: 2

Model Prediction

Prediction: 0.116
Unit: g/kg_body_weight
Mahalanobis Distance: 7.7
Mahalanobis Distance p-value: 0.00454

Mahalanobis Distance: The Mahalanobis distance (MD) is a generalization of the Euclidean distance that accounts for correlations among the X properties. It is calculated as the distance to the center of the training data. The larger the MD, the less trustworthy the prediction.
Mahalanobis Distance p-value: The p-value gives the fraction of training data with an MD greater than or equal to the one for the given sample, assuming normally distributed data. The smaller the p-value, the less trustworthy the prediciton. For highly non-normal X properties (e.g., fingerprints), the MD p-value is wildly inaccurate.

Structural Similar Compounds

Name	4-HEXYLRESORCINOL	o-BENZYL-p-CHLOROPHENOL	1-TRANS-DELTA(9)-TETRAHYDROCANNABINOL
Structure	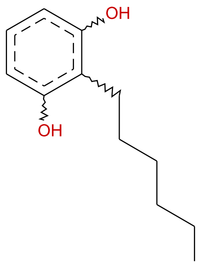	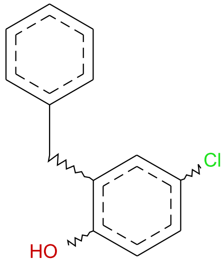	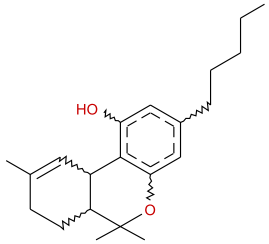
Actual Endpoint (-log C)	3.1915	3.26063	3.79861
Predicted Endpoint (-log C)	2.16134	3.64448	4.44032
Distance	0.473	0.615	0.661
Reference	NCI/NTP TR-330	NCI/NTP TR-424	NCI/NTP TR-446

Model Applicability

Unknown features are fingerprint features in the query molecule, but not found or appearing too infrequently in the training set.

1. All properties and OPS components are within expected ranges.

Feature Contribution

Top features for positive contribution

Fingerprint	Bit/Smiles	Feature Structure	Score
FCFP_2	129344189	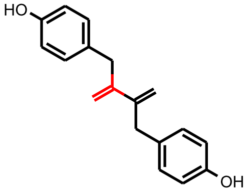 [*]C=C	0.519

FCFP_2	7	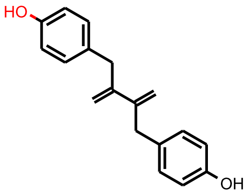 [*]O	0.0144
FCFP_2	74595001	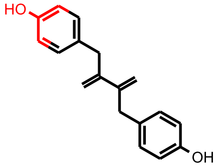 [*][c](:[*]):[c](O):[cH]:[*]	0.000246
**Top Features for negative contribution**			
**Fingerprint**	**Bit/Smiles**	**Feature Structure**	**Score**
FCFP_2	203677720	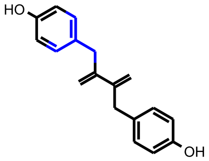 [*]C([*])[c](:[cH]:[*]):[c](:[*]):[*]	-0.406
FCFP_2	0	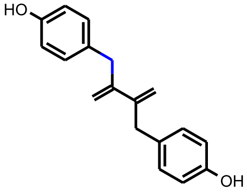 [*]C([*])[*]	-0.29

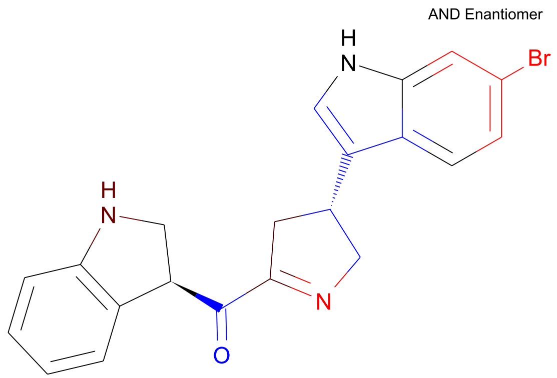

$C_{21}H_{18}BrN_3O$
Molecular Weight: 408.29112
ALogP: 3.919
Rotatable Bonds: 3
Acceptors: 3
Donors: 2

Model Prediction

Prediction: 0.00743
Unit: g/kg_body_weight
Mahalanobis Distance: 12
Mahalanobis Distance p-value: 8.63e-009

Mahalanobis Distance: The Mahalanobis distance (MD) is a generalization of the Euclidean distance that accounts for correlations among the X properties. It is calculated as the distance to the center of the training data. The larger the MD, the less trustworthy the prediction.
Mahalanobis Distance p-value: The p-value gives the fraction of training data with an MD greater than or equal to the one for the given sample, assuming normally distributed data. The smaller the p-value, the less trustworthy the prediciton. For highly non-normal X properties (e.g., fingerprints), the MD p-value is wildly inaccurate.

Structural Similar Compounds

Name	PHENYLBUTAZONE	o-BENZYL-p-CHLOROPHENOL	SULFISOOXAZOLE
Structure	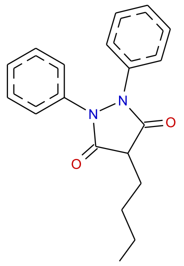	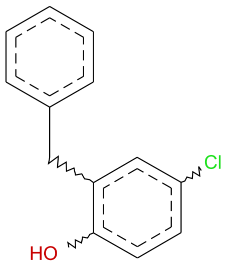	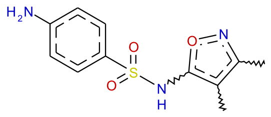
Actual Endpoint (-log C)	3.48909	3.26063	2.82494
Predicted Endpoint (-log C)	3.17333	3.64448	3.0705
Distance	0.973	0.984	1.009
Reference	NCI/NTP TR-367	NCI/NTP TR-424	NCI/NTP TR-138

Model Applicability

Unknown features are fingerprint features in the query molecule, but not found or appearing too infrequently in the training set.

1. Num_AromaticRings out of range. Value: 3. Training min, max, mean, SD: 0, 2, 0.5625, 0.693.
2. Unknown FCFP_2 feature: 19: [*]:[nH]:[*]
3. Unknown FCFP_2 feature: 307448885: [*]:[c]1:[*]:[*]:[nH]:[c]:1:c:[*]
4. Unknown FCFP_2 feature: 1618184456: [*][c]1:[*]:[*]:[nH]:c:1
5. Unknown FCFP_2 feature: 2005402822: [*]:[c]1:[*]:[*]:[c](:[*]):[nH]:1
6. Unknown FCFP_2 feature: 580930443: [*]C1=NC[*][*]1

Feature Contribution

Top features for positive contribution			
**Fingerprint**	**Bit/Smiles**	**Feature Structure**	**Score**
FCFP_2	32	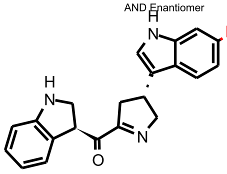 [*]Br	0.526

FCFP_2	1	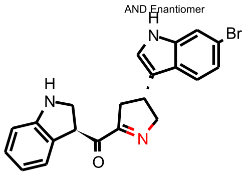 [*]O[*]	0.511
FCFP_2	367998008	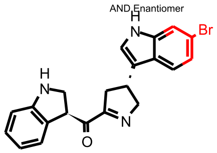 [*]:[cH]:[c](Br):[cH]:[*]	0.413

Top Features for negative contribution

**Fingerprint**	**Bit/Smiles**	**Feature Structure**	**Score**
FCFP_2	203677720	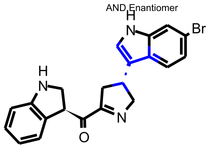 [*]C([*])[c](:[cH]:[*]):[c](:[*]):[*]	-0.406
FCFP_2	1872154524	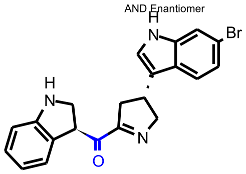 [*]C(=O)[*]	-0.307
FCFP_2	0	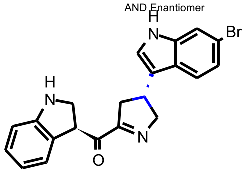 [*]C([*])[*]	-0.29

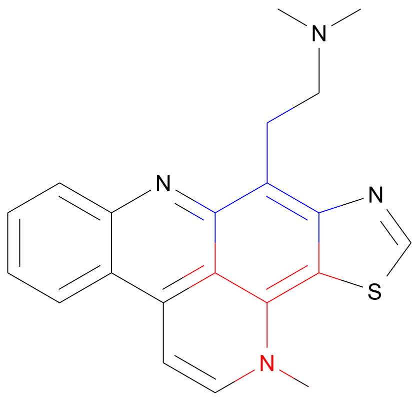

$C_{21}H_{20}N_4S$

Molecular Weight: 360.4753

ALogP: 3.682

Rotatable Bonds: 3

Acceptors: 4

Donors: 0

Model Prediction

Prediction: 0.0015

Unit: g/kg_body_weight

Mahalanobis Distance: 9.65

Mahalanobis Distance p-value: 1.71e-005

Mahalanobis Distance: The Mahalanobis distance (MD) is a generalization of the Euclidean distance that accounts for correlations among the X properties. It is calculated as the distance to the center of the training data. The larger the MD, the less trustworthy the prediction.
Mahalanobis Distance p-value: The p-value gives the fraction of training data with an MD greater than or equal to the one for the given sample, assuming normally distributed data. The smaller the p-value, the less trustworthy the prediciton. For highly non-normal X properties (e.g., fingerprints), the MD p-value is wildly inaccurate.

Structural Similar Compounds

Name	8-METHOXYPSORALEN	PROMETHAZINE.HCL	CHLORPHENIRAMINE MALEATE
Structure	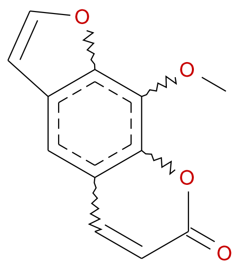	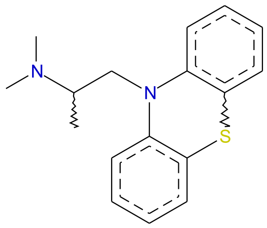	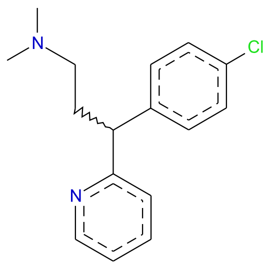
Actual Endpoint (-log C)	3.45978	3.93152	3.96188
Predicted Endpoint (-log C)	4.14745	4.72433	3.83117
Distance	0.902	0.932	0.983
Reference	NCI/NTP TR-359	NCI/NTP TR-425	NCI/NTP TR-317

Model Applicability

Unknown features are fingerprint features in the query molecule, but not found or appearing too infrequently in the training set.

1. Num_AromaticRings out of range. Value: 4. Training min, max, mean, SD: 0, 2, 0.5625, 0.693.
2. OPS PC6 out of range. Value: -3.6163. Training min, max, SD, explained variance: -2.4321, 2.9885, 1.256, 0.0488.
3. Unknown FCFP_2 feature: -124685461: [*]1:[*]:s:c:n:1

Feature Contribution

Top features for positive contribution

Fingerprint	Bit/Smiles	Feature Structure	Score
FCFP_2	332760439	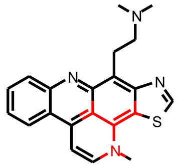 [*]O[c](:[cH]:[*]):[c]([*]):[*]	0.672

FCFP_2	1	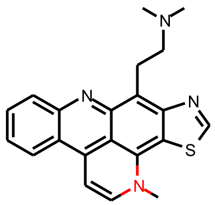 [*]O[*]	0.511
FCFP_2	136627117	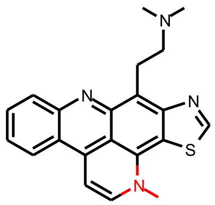 [*]OC	0.0304
**Top Features for negative contribution**			
**Fingerprint**	**Bit/Smiles**	**Feature Structure**	**Score**
FCFP_2	203677720	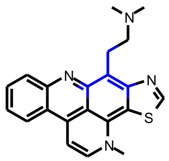 [*]C([*])[c](:[cH]:[*]):[c](:[*]):[*]	-0.406
FCFP_2	0	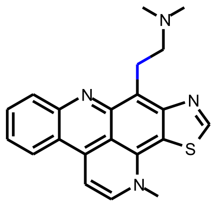 [*]C([*])[*]	-0.29

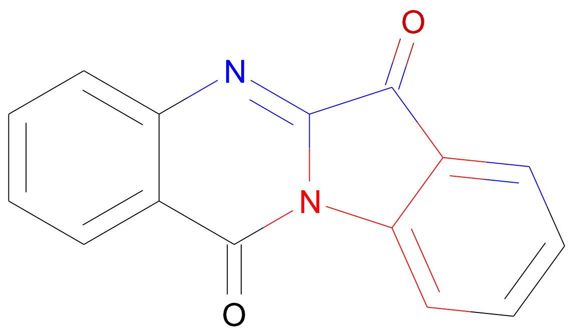

$C_{15}H_8N_2O_2$
Molecular Weight: 248.23621
ALogP: 2.331
Rotatable Bonds: 0
Acceptors: 3
Donors: 0

Model Prediction

Prediction: 0.0197
Unit: g/kg_body_weight
Mahalanobis Distance: 5.13
Mahalanobis Distance p-value: 0.512

Mahalanobis Distance: The Mahalanobis distance (MD) is a generalization of the Euclidean distance that accounts for correlations among the X properties. It is calculated as the distance to the center of the training data. The larger the MD, the less trustworthy the prediction.
Mahalanobis Distance p-value: The p-value gives the fraction of training data with an MD greater than or equal to the one for the given sample, assuming normally distributed data. The smaller the p-value, the less trustworthy the prediciton. For highly non-normal X properties (e.g., fingerprints), the MD p-value is wildly inaccurate.

Structural Similar Compounds

Name	8-METHOXYPSORALEN	3,4-DIHYDROCOUMARIN	COUMARIN
Structure	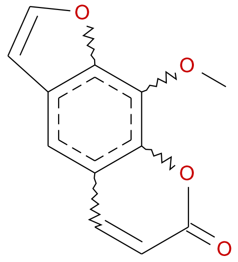	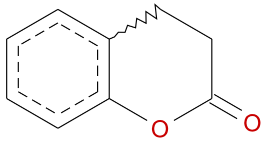	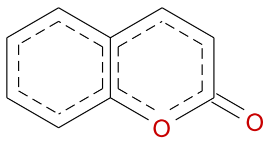
Actual Endpoint (-log C)	3.45978	2.69361	3.76684
Predicted Endpoint (-log C)	4.14745	3.51534	3.6624
Distance	0.404	0.616	0.619
Reference	NCI/NTP TR-359	NCI/NTP TR-423	NCI/NTP TR-422

Model Applicability

Unknown features are fingerprint features in the query molecule, but not found or appearing too infrequently in the training set.

1. OPS PC6 out of range. Value: -2.63. Training min, max, SD, explained variance: -2.4321, 2.9885, 1.256, 0.0488.
2. Unknown FCFP_2 feature: 580453787: [*]C(=N[c](:[*]):[*])[*]
3. Unknown FCFP_2 feature: -1549192822: [*]C(=[*])C(=O)[c](:[*]):[*]

Feature Contribution

Top features for positive contribution

Fingerprint	Bit/Smiles	Feature Structure	Score
FCFP_2	332760439	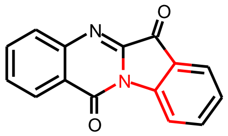 [*]O[c](:[cH]:[*]):[c]([*]):[*]	0.672

FCFP_2	1	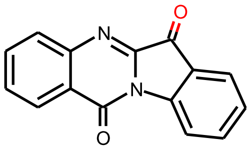<br>[*]O[*]	0.511

Top Features for negative contribution

Fingerprint	Bit/Smiles	Feature Structure	Score
FCFP_2	203677720	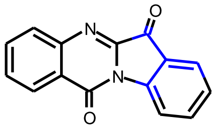<br>[*]C([*])[c](:[cH]:[*]):[c](:[*]):[*]	-0.406
FCFP_2	565998553	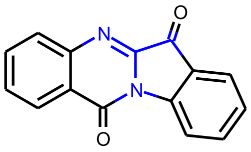<br>[*][C@@H]1[*][*]OC1=O	-0.348
FCFP_2	1872154524	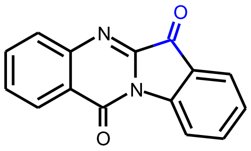<br>[*]C(=O)[*]	-0.307

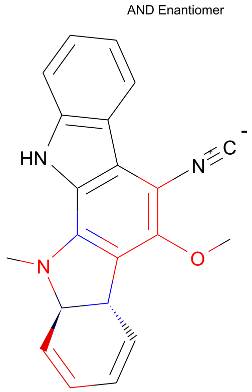

$C_{21}H_{17}N_3O$

Molecular Weight: 327.37918

ALogP: 4.078

Rotatable Bonds: 1

Acceptors: 2

Donors: 1

Model Prediction

Prediction: 0.00518

Unit: g/kg_body_weight

Mahalanobis Distance: 9.31

Mahalanobis Distance p-value: 4.84e-005

Mahalanobis Distance: The Mahalanobis distance (MD) is a generalization of the Euclidean distance that accounts for correlations among the X properties. It is calculated as the distance to the center of the training data. The larger the MD, the less trustworthy the prediction.
Mahalanobis Distance p-value: The p-value gives the fraction of training data with an MD greater than or equal to the one for the given sample, assuming normally distributed data. The smaller the p-value, the less trustworthy the prediciton. For highly non-normal X properties (e.g., fingerprints), the MD p-value is wildly inaccurate.

Structural Similar Compounds

Name	8-METHOXYPSORALEN	PHENYLBUTAZONE	PROMETHAZINE.HCL
Structure	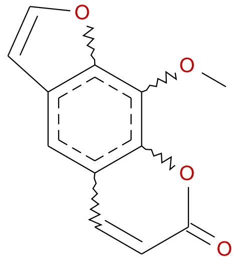	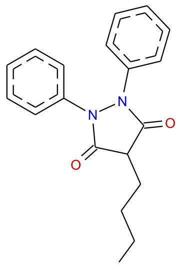	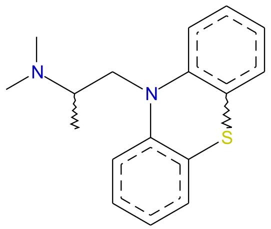
Actual Endpoint (-log C)	3.45978	3.48909	3.93152
Predicted Endpoint (-log C)	4.14745	3.17333	4.72433
Distance	0.792	0.841	0.845
Reference	NCI/NTP TR-359	NCI/NTP TR-367	NCI/NTP TR-425

Model Applicability

Unknown features are fingerprint features in the query molecule, but not found or appearing too infrequently in the training set.

1. Num_AromaticRings out of range. Value: 3. Training min, max, mean, SD: 0, 2, 0.5625, 0.693.
2. OPS PC6 out of range. Value: -2.8793. Training min, max, SD, explained variance: -2.4321, 2.9885, 1.256, 0.0488.
3. Unknown FCFP_2 feature: 19: [*]:[nH]:[*]
4. Unknown FCFP_2 feature: 8: [*][N+]#[*]
5. Unknown FCFP_2 feature: 4: [*]#[C-]
6. Unknown FCFP_2 feature: -828984032: [*][c](:[*]):[c]([N+]#[*]):[c](:[*]):[*]
7. Unknown FCFP_2 feature: 307448885: [*]:[c]1:[*]:[*]:[nH]:[c]:1:c:[*]
8. Unknown FCFP_2 feature: 2005402822: [*]:[c]1:[*]:[*]:[c](:[*]):[nH]:1
9. Unknown FCFP_2 feature: 1934974835: [*]:[c](:[*])[N+]#[C-]
10. Unknown FCFP_2 feature: -1487147388: [*][N+]#[C-]

Feature Contribution

Top features for positive contribution			
Fingerprint	Bit/Smiles	Feature Structure	Score

FCFP_2	332760439	AND Enantiomer 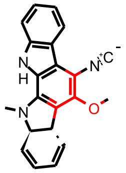 [*]O[c](:[cH]:[*]):[c]([*]):[*]	0.672
FCFP_2	1	AND Enantiomer 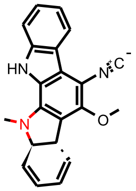 [*]O[*]	0.511
FCFP_2	451847724	AND Enantiomer 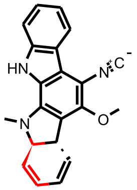 [*]CC=C([*])[*]	0.225
**Top Features for negative contribution**			
**Fingerprint**	**Bit/Smiles**	**Feature Structure**	**Score**
FCFP_2	203677720	AND Enantiomer 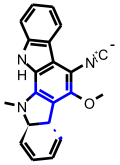 [*]C([*])[c](:[cH]:[*]):[c](:[*]):[*]	-0.406
FCFP_2	0	AND Enantiomer 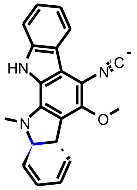 [*]C([*])[*]	-0.29

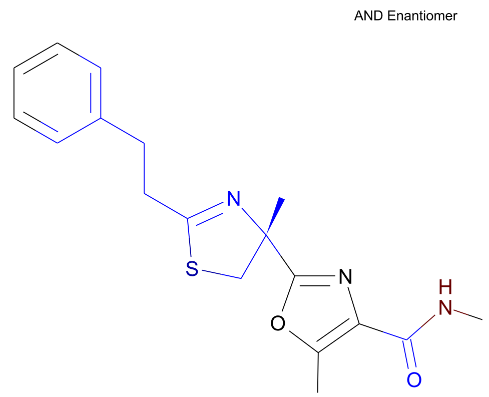

$C_{18}H_{21}N_3O_2S$
Molecular Weight: 343.44324
ALogP: 2.716
Rotatable Bonds: 5
Acceptors: 4
Donors: 1

Model Prediction

Prediction: 1.22
Unit: g/kg_body_weight
Mahalanobis Distance: 8.24
Mahalanobis Distance p-value: 0.00107

Mahalanobis Distance: The Mahalanobis distance (MD) is a generalization of the Euclidean distance that accounts for correlations among the X properties. It is calculated as the distance to the center of the training data. The larger the MD, the less trustworthy the prediction.
Mahalanobis Distance p-value: The p-value gives the fraction of training data with an MD greater than or equal to the one for the given sample, assuming normally distributed data. The smaller the p-value, the less trustworthy the prediciton. For highly non-normal X properties (e.g., fingerprints), the MD p-value is wildly inaccurate.

Structural Similar Compounds

Name	PROBENECID	SULFISOOXAZOLE	PENICILLIN VK
Structure	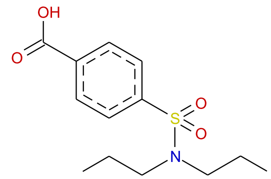	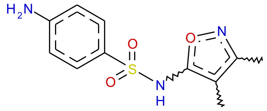	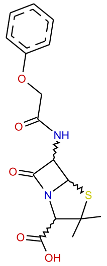
Actual Endpoint (-log C)	2.85333	2.82494	2.54455
Predicted Endpoint (-log C)	2.4258	3.0705	3.9702
Distance	0.688	0.730	0.835
Reference	NCI/NTP TR-395	NCI/NTP TR-138	NCI/NTP TR-336

Model Applicability

Unknown features are fingerprint features in the query molecule, but not found or appearing too infrequently in the training set.

1. All properties and OPS components are within expected ranges.
2. Unknown FCFP_2 feature: -1539162406: [*]C([*])([*])[c]1:o:[*]:[*]:n:1
3. Unknown FCFP_2 feature: -836603894: [*]:[c](:[*])[C@]1(C)C[*][*]=N1
4. Unknown FCFP_2 feature: 580930443: [*]C1=NC[*][*]1
5. Unknown FCFP_2 feature: 136686699: [*]NC

Feature Contribution

Top features for positive contribution

Fingerprint	Bit/Smiles	Feature Structure	Score
FCFP_2	1	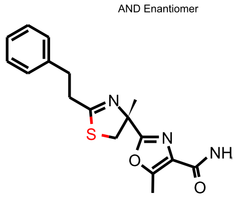 [*]O[*]	0.511

FCFP_2	3	AND Enantiomer<br>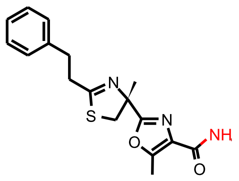<br>[*]N[*]	0.104
FCFP_2	-1272798659	AND Enantiomer<br>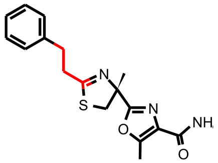<br>[*][C@H]1[*][*]CC1	0.0703

Top Features for negative contribution

Fingerprint	Bit/Smiles	Feature Structure	Score
FCFP_2	136597326	AND Enantiomer<br>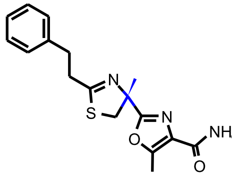<br>[*]C(=[*])C	-0.489
FCFP_2	203677720	AND Enantiomer<br>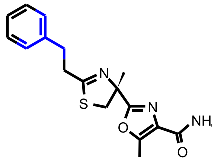<br>[*]C([*])[c](:[cH]:[*]):[c](:[*]):[*]	-0.406
FCFP_2	565998553	AND Enantiomer<br>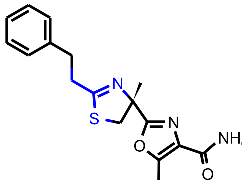<br>[*][C@@H]1[*][*]OC1=O	-0.348

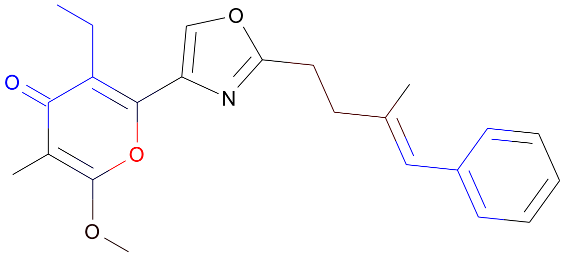

$C_{23}H_{25}NO_4$
Molecular Weight: 379.4489
ALogP: 5.22
Rotatable Bonds: 7
Acceptors: 4
Donors: 0

Model Prediction

Prediction: 0.115
Unit: g/kg_body_weight
Mahalanobis Distance: 8.9
Mahalanobis Distance p-value: 0.000163

Mahalanobis Distance: The Mahalanobis distance (MD) is a generalization of the Euclidean distance that accounts for correlations among the X properties. It is calculated as the distance to the center of the training data. The larger the MD, the less trustworthy the prediction.
Mahalanobis Distance p-value: The p-value gives the fraction of training data with an MD greater than or equal to the one for the given sample, assuming normally distributed data. The smaller the p-value, the less trustworthy the prediciton. For highly non-normal X properties (e.g., fingerprints), the MD p-value is wildly inaccurate.

Structural Similar Compounds

Name	PHENYLBUTAZONE	DIALLYL PHTHALATE	CHLORPHENIRAMINE MALEATE
Structure	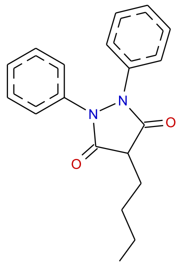	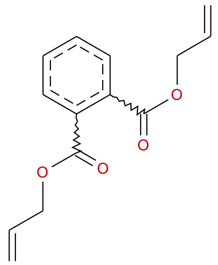	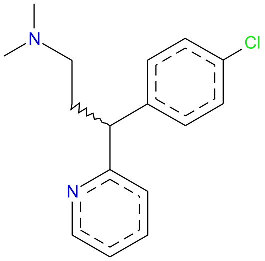
Actual Endpoint (-log C)	3.48909	3.3914	3.96188
Predicted Endpoint (-log C)	3.17333	3.50093	3.83117
Distance	0.713	0.829	0.834
Reference	NCI/NTP TR-367	NCI/NTP TR-284	NCI/NTP TR-317

Model Applicability

Unknown features are fingerprint features in the query molecule, but not found or appearing too infrequently in the training set.

1. All properties and OPS components are within expected ranges.
2. Unknown FCFP_2 feature: -1678245750: [*]OC(=C([*])[*])[c](:[*]):[*]
3. Unknown FCFP_2 feature: -1539162406: [*]C([*])([*])[c]1:o:[*]:[*]:n:1
4. Unknown FCFP_2 feature: -2115241127: [*]OC(=C([*])[*])O[*]

Feature Contribution

Top features for positive contribution			
**Fingerprint**	**Bit/Smiles**	**Feature Structure**	**Score**
FCFP_2	1	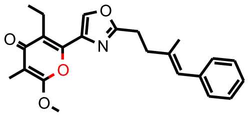 [*]O[*]	0.511

FCFP_2	-1272798659	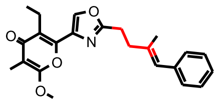 [*][C@H]1[*][*]CC1	0.0703
FCFP_2	136627117	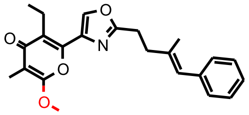 [*]OC	0.0304
**Top Features for negative contribution**			
**Fingerprint**	**Bit/Smiles**	**Feature Structure**	**Score**
FCFP_2	136597326	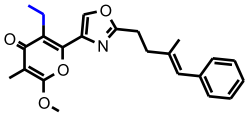 [*]C(=[*])C	-0.489
FCFP_2	203677720	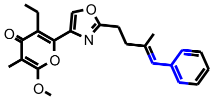 [*]C([*])[c](:[cH]:[*]):[c](:[*]):[*]	-0.406
FCFP_2	1872154524	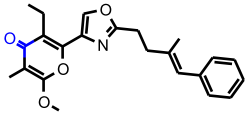 [*]C(=O)[*]	-0.307

Remdesivir

TOPKAT_Rat_Maximum_Tolerated_Dose_Gavage

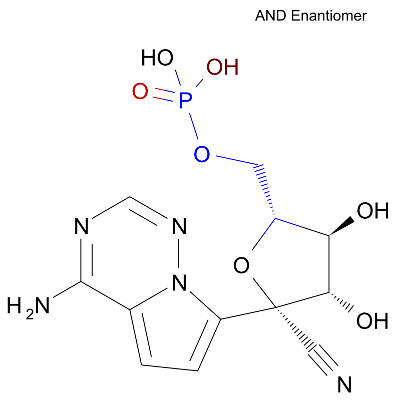

$C_{12}H_{14}N_5O_7P$

Molecular Weight: 371.24262

ALogP: -1.502

Rotatable Bonds: 4

Acceptors: 11

Donors: 5

Model Prediction

Prediction: 0.000298

Unit: g/kg_body_weight

Mahalanobis Distance: 17.2

Mahalanobis Distance p-value: 5.05e-016

Mahalanobis Distance: The Mahalanobis distance (MD) is a generalization of the Euclidean distance that accounts for correlations among the X properties. It is calculated as the distance to the center of the training data. The larger the MD, the less trustworthy the prediction.
Mahalanobis Distance p-value: The p-value gives the fraction of training data with an MD greater than or equal to the one for the given sample, assuming normally distributed data. The smaller the p-value, the less trustworthy the prediciton. For highly non-normal X properties (e.g., fingerprints), the MD p-value is wildly inaccurate.

Structural Similar Compounds

Name	AMPICILLIN TRIHYDRATE	OCHRATOXIN	PENICILLIN VK
Structure	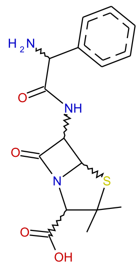	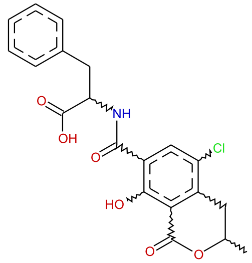	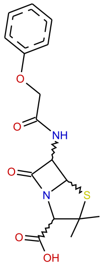
Actual Endpoint (-log C)	2.36724	6.28396	2.54455
Predicted Endpoint (-log C)	2.27651	5.12358	3.9702
Distance	1.255	1.482	1.498
Reference	NCI/NTP TR-318	NCI/NTP TR-358	NCI/NTP TR-336

Model Applicability

Unknown features are fingerprint features in the query molecule, but not found or appearing too infrequently in the training set.

1. Num_H_Donors out of range. Value: 5. Training min, max, mean, SD: 0, 3, 0.4375, 0.8311.
2. Num_H_Acceptors out of range. Value: 11. Training min, max, mean, SD: 0, 6, 1.6146, 1.644.
3. Molecular_PolarSASA out of range. Value: 321.97. Training min, max, mean, SD: 0, 223.97, 50.816, 55.15.
4. Molecular_PolarSurfaceArea out of range. Value: 206.26. Training min, max, mean, SD: 0, 138.03, 28.978, 32.1.
5. OPS PC1 out of range. Value: 9.0116. Training min, max, SD, explained variance: -4.0008, 7.9165, 2.861, 0.2531.
6. OPS PC5 out of range. Value: -4.1876. Training min, max, SD, explained variance: -3.4, 4.1587, 1.489, 0.0686.
7. OPS PC9 out of range. Value: -2.7276. Training min, max, SD, explained variance: -2.7086, 2.9267, 1.019, 0.0321.
8. Unknown FCFP_2 feature: 472180098: [*]OP(=O)(O)O
9. Unknown FCFP_2 feature: -836603894: [*]:[c](:[*])[C@]1(C)C[*][*]=N1
10. Unknown FCFP_2 feature: -1277879912: [*]C([*])([*])C#N
11. Unknown FCFP_2 feature: -1362791977: [*]C#N
12. Unknown FCFP_2 feature: -332197802: [*][c]1:[*]:[*]:[c](:[*]):n:1:n:[*]
13. Unknown FCFP_2 feature: -124685461: [*]1:[*]:s:c:n:1

Feature Contribution

Top features for positive contribution

Fingerprint	Bit/Smiles	Feature Structure	Score
FCFP_2	1	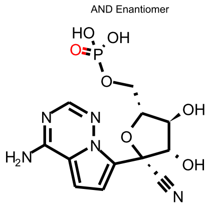 [*]O[*]	0.511
FCFP_2	3	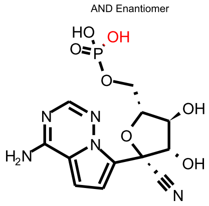 [*]N[*]	0.104

Top Features for negative contribution

Fingerprint	Bit/Smiles	Feature Structure	Score
FCFP_2	1872154524	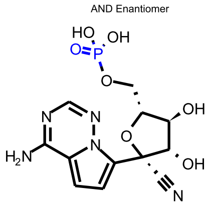 [*]C(=O)[*]	-0.307
FCFP_2	0	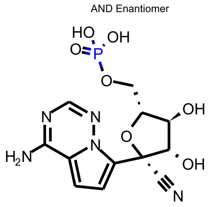 [*]C([*])[*]	-0.29

FCFP_2	-1272768868	AND Enantiomer 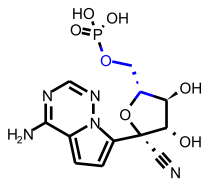 [*][C@@H]1[*][*]OC1	-0.271

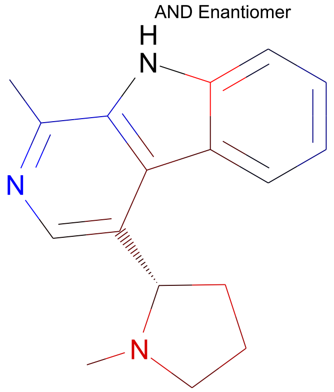

$C_{17}H_{19}N_3$

Molecular Weight: 265.35286

ALogP: 3.018

Rotatable Bonds: 1

Acceptors: 2

Donors: 1

Structural Similar Compounds

Name	3-AMINO-9-ETHYLCARBAZOLE .HCl (HCl STRIPPED)	TRP-P-1	BENZOPHENONE; 4'-CHLORO-2-(2-IMIDAZOL-2-YL)-
Structure	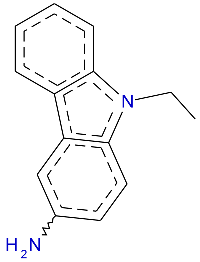	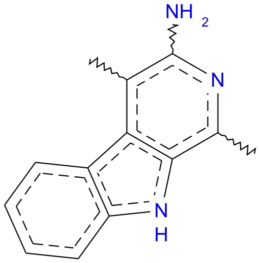	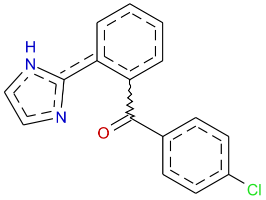
Actual Endpoint (-log C)	2.954	3.325	2.673
Predicted Endpoint (-log C)	2.71735	2.34981	3.18028
Distance	0.553	0.581	0.582
Reference	JPETAB 99;450;50	PPTCBY 9;159;79	JMCMAR 18;182;75

Model Prediction

Prediction: 0.375

Unit: g/kg_body_weight

Mahalanobis Distance: 18.4

Mahalanobis Distance p-value: 8.86e-005

Mahalanobis Distance: The Mahalanobis distance (MD) is a generalization of the Euclidean distance that accounts for correlations among the X properties. It is calculated as the distance to the center of the training data. The larger the MD, the less trustworthy the prediction.
Mahalanobis Distance p-value: The p-value gives the fraction of training data with an MD greater than or equal to the one for the given sample, assuming normally distributed data. The smaller the p-value, the less trustworthy the prediciton. For highly non-normal X properties (e.g., fingerprints), the MD p-value is wildly inaccurate.

Model Applicability

Unknown features are fingerprint features in the query molecule, but not found or appearing too infrequently in the training set.

1. All properties and OPS components are within expected ranges.
2. Unknown FCFP_6 feature: 16: [*]:[cH]:[*]
3. Unknown FCFP_6 feature: 19: [*]:[nH]:[*]
4. Unknown FCFP_6 feature: 1618154665: [*]:[cH]:[cH]:[cH]:[*]
5. Unknown FCFP_6 feature: 307448885: [*]:[cH]:[c]1:[nH]:[*]:[*]:[c]:1:[*]
6. Unknown FCFP_6 feature: 2005402822: [*]:[c]1:[*]:[*]:[c](:[*]):[nH]:1
7. Unknown FCFP_6 feature: 1747237384: [*][c](:[*]):n:[cH]:[*]
8. Unknown FCFP_6 feature: 1871533475: [*]N1[*][*]C[C@H]1[c](:[*]):[*]

Feature Contribution

Top features for positive contribution

Fingerprint	Bit/Smiles	Feature Structure	Score

ECFP_6	642810091	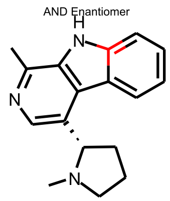 [*]:[c](:[*]):[*]	0.281
FCFP_6	309602933	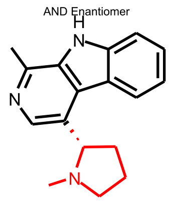 [*][C@@H]1CCCN1C	0.125
FCFP_6	136388789	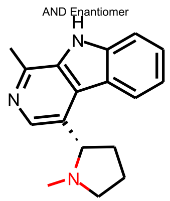 [*]N([*])C	0.119

Top Features for negative contribution

**Fingerprint**	**Bit/Smiles**	**Feature Structure**	**Score**
ECFP_6	655739385	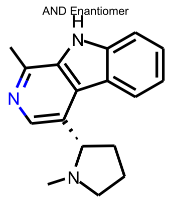 [*]:n:[*]	-0.239
ECFP_6	734603939	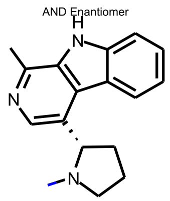 [*]C	-0.201

FCFP_6	-1539132615	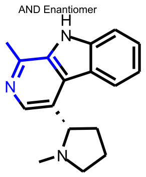<br><br>[*]:n:[c](C):[c](:[*]):[*]	-0.2

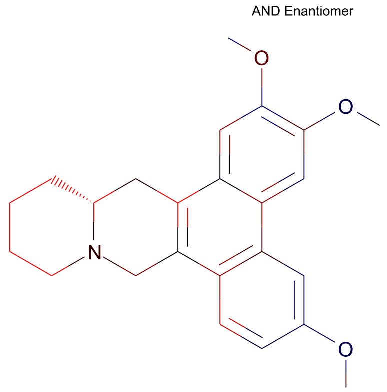

$C_{24}H_{27}NO_3$
Molecular Weight: 377.47608
ALogP: 4.691
Rotatable Bonds: 3
Acceptors: 4
Donors: 0

Structural Similar Compounds

Name	TYLOCREBRINE	OXYRIDAZINE	METHOPHOLINE
Structure	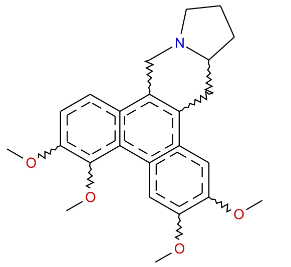	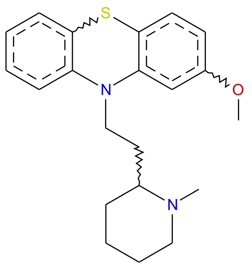	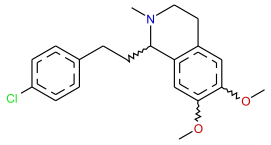
Actual Endpoint (-log C)	3.782	2.497	2.937
Predicted Endpoint (-log C)	3.12068	3.05313	2.8769
Distance	0.279	0.553	0.584
Reference	NCICP* -;77;64	27ZQAG -;27;72	MDCHAG 5;318;65

Model Prediction

Prediction: 0.174
Unit: g/kg_body_weight
Mahalanobis Distance: 18.4
Mahalanobis Distance p-value: 0.000124

Mahalanobis Distance: The Mahalanobis distance (MD) is a generalization of the Euclidean distance that accounts for correlations among the X properties. It is calculated as the distance to the center of the training data. The larger the MD, the less trustworthy the prediction.
Mahalanobis Distance p-value: The p-value gives the fraction of training data with an MD greater than or equal to the one for the given sample, assuming normally distributed data. The smaller the p-value, the less trustworthy the prediciton. For highly non-normal X properties (e.g., fingerprints), the MD p-value is wildly inaccurate.

Model Applicability

Unknown features are fingerprint features in the query molecule, but not found or appearing too infrequently in the training set.

1. All properties and OPS components are within expected ranges.
2. Unknown FCFP_6 feature: 16: [*]:[cH]:[*]
3. Unknown FCFP_6 feature: -1946918893: [*]CC(C[*])N([*])[*]
4. Unknown FCFP_6 feature: 906798516: [*]N([*])C[c](:[*]):[*]
5. Unknown FCFP_6 feature: 1618154665: [*]:[cH]:[cH]:[cH]:[*]

Feature Contribution

Top features for positive contribution			
Fingerprint	Bit/Smiles	Feature Structure	Score
ECFP_6	642810091	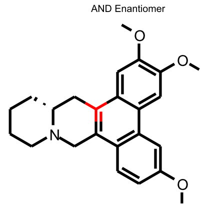 [*]:[c](:[*]):[*]	0.281

FCFP_6	136627117	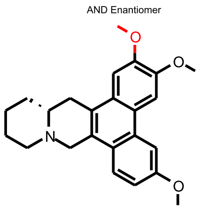 [*]OC	0.17
FCFP_6	309602933	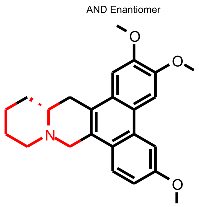 [*][C@@H]1CCCN1C	0.125
**Top Features for negative contribution**			
**Fingerprint**	**Bit/Smiles**	**Feature Structure**	**Score**
ECFP_6	-176455838	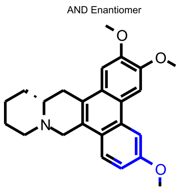 [*]O[c](:[cH]:[*]):[c H]:[*]	-0.257
ECFP_6	734603939	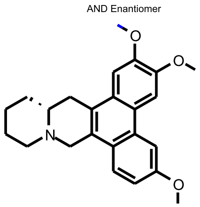 [*]C	-0.201
FCFP_6	1036089772	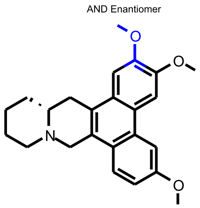 [*]:[c](:[*])OC	-0.136

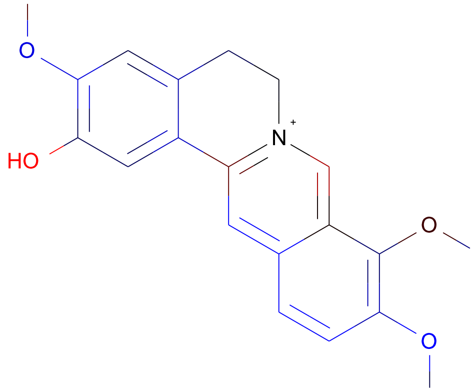

$C_{20}H_{20}NO_4$
Molecular Weight: 338.3771
ALogP: 3.936
Rotatable Bonds: 3
Acceptors: 4
Donors: 1

Model Prediction

Prediction: 0.816
Unit: g/kg_body_weight
Mahalanobis Distance: 17.6
Mahalanobis Distance p-value: 0.00414

Mahalanobis Distance: The Mahalanobis distance (MD) is a generalization of the Euclidean distance that accounts for correlations among the X properties. It is calculated as the distance to the center of the training data. The larger the MD, the less trustworthy the prediction.
Mahalanobis Distance p-value: The p-value gives the fraction of training data with an MD greater than or equal to the one for the given sample, assuming normally distributed data. The smaller the p-value, the less trustworthy the prediciton. For highly non-normal X properties (e.g., fingerprints), the MD p-value is wildly inaccurate.

Structural Similar Compounds

Name	TRIARIMOL	.alpha.-(2-CHLOROPHENYL)-.alpha.-(4-CHLOROPHENYL)-5-PYRIMIDINE METHANOL	INDOMETHAZINE
Structure	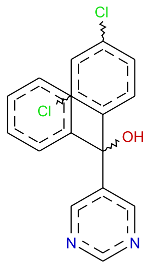	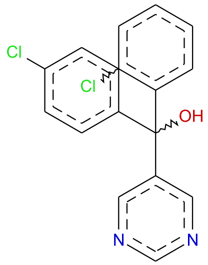	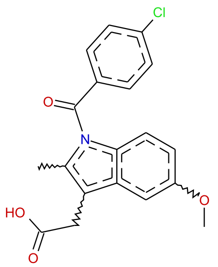
Actual Endpoint (-log C)	2.742	2.122	5.17
Predicted Endpoint (-log C)	2.87257	2.72534	3.33605
Distance	0.516	0.518	0.541
Reference	FMCHA2 -;C293;89	FMCHA2 -;C254;89	ARZNAD 25;1526;75

Model Applicability

Unknown features are fingerprint features in the query molecule, but not found or appearing too infrequently in the training set.

1. All properties and OPS components are within expected ranges.
2. Unknown ECFP_2 feature: -1508366470: [*][n+](:[*]):[*]
3. Unknown ECFP_2 feature: 1508268466: [*]C[n+](:[c]([*]):[*]):c:[*]
4. Unknown ECFP_2 feature: -1333923932: [*]CC[n+](:[*]):[*]
5. Unknown ECFP_2 feature: 2078070048: [*][n+](:[*]):[c]([c](:[*]):[*]):c:[*]
6. Unknown ECFP_2 feature: 688916016: [*][n+](:[*]):c:[c](:[*]):[*]
7. Unknown FCFP_6 feature: 24: [*][n+](:[*]):[*]
8. Unknown FCFP_6 feature: 16: [*]:[cH]:[*]
9. Unknown FCFP_6 feature: -549108873: [*]:[c](:[*])O
10. Unknown FCFP_6 feature: 414371600: [*]C[n+](:[cH]:[*]):[c]([*]):[*]
11. Unknown FCFP_6 feature: -150573739: [*]CC[n+](:[*]):[*]
12. Unknown FCFP_6 feature: -1861407456: [*][n+](:[*]):[c](:[cH]:[*])[c](:[*]):[*]
13. Unknown FCFP_6 feature: -1861645784: [*][c](:[*]):[c](:[cH]:[*])[c](:[*]):[*]
14. Unknown FCFP_6 feature: 1618392993: [*][n+](:[*]):[cH]:[c](:[*]):[*]
15. Unknown FCFP_6 feature: 1618154665: [*]:[cH]:[cH]:[cH]:[*]
16. Unknown FCFP_6 feature: 74595001: [*][c](:[*]):[c](O):[cH]:[*]

Feature Contribution

Top features for positive contribution			
**Fingerprint**	**Bit/Smiles**	**Feature Structure**	**Score**
ECFP_6	642810091	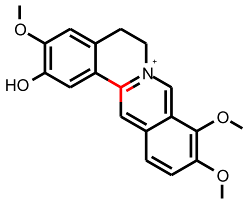 [*]:[c](:[*]):[*]	0.281
FCFP_6	136627117	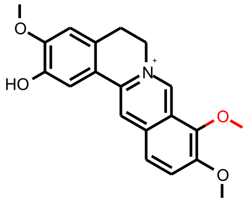 [*]OC	0.17
ECFP_6	1334973442	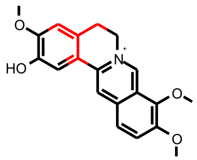 [*]C[c](:[cH]:[*]):[c]([*]):[*]	0.15

Top Features for negative contribution			
**Fingerprint**	**Bit/Smiles**	**Feature Structure**	**Score**
FCFP_6	1676877079	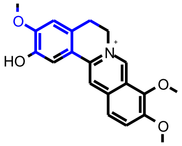 [*]C[c]1:[cH]:[c](O[*]):[c]([*]):[*]:[c]: 1[*]	-0.254

ECFP_6	2077607946	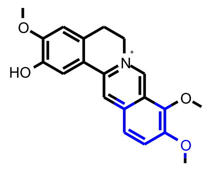 [*]O[c]1:[cH]:[cH]:[c](:[*]):[*]:[c]:1[*]	-0.252
ECFP_6	734603939	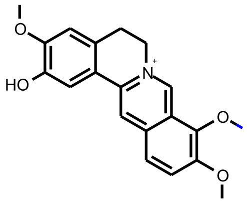 [*]C	-0.201

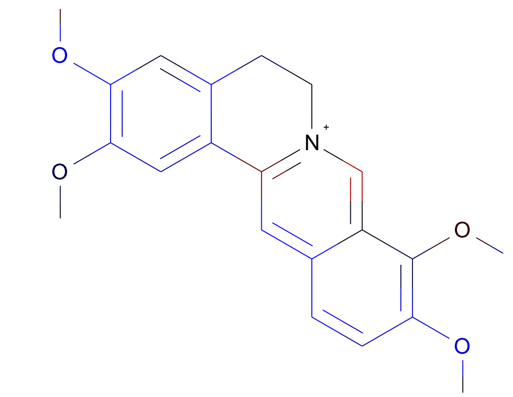

$C_{21}H_{22}NO_4$
Molecular Weight: 352.40368
ALogP: 4.161
Rotatable Bonds: 4
Acceptors: 4
Donors: 0

Model Prediction

Prediction: 1.28
Unit: g/kg_body_weight
Mahalanobis Distance: 17.1
Mahalanobis Distance p-value: 0.0222

Mahalanobis Distance: The Mahalanobis distance (MD) is a generalization of the Euclidean distance that accounts for correlations among the X properties. It is calculated as the distance to the center of the training data. The larger the MD, the less trustworthy the prediction.
Mahalanobis Distance p-value: The p-value gives the fraction of training data with an MD greater than or equal to the one for the given sample, assuming normally distributed data. The smaller the p-value, the less trustworthy the prediciton. For highly non-normal X properties (e.g., fingerprints), the MD p-value is wildly inaccurate.

Structural Similar Compounds

Name	TYLOCREBRINE	PAPAVERINE	TRIPHENYLPHOSPHATE
Structure	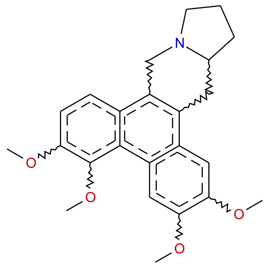	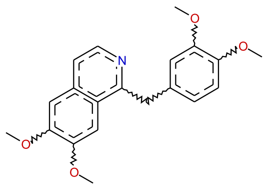	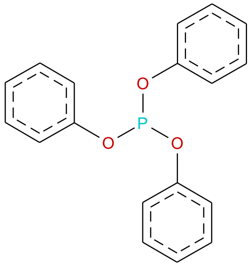
Actual Endpoint (-log C)	3.782	3.019	2.288
Predicted Endpoint (-log C)	3.12068	1.94961	2.22473
Distance	0.441	0.476	0.554
Reference	NCICP* -;77;64	ARZNAD 20;1338;70	14CYAT 2;1918;63

Model Applicability

Unknown features are fingerprint features in the query molecule, but not found or appearing too infrequently in the training set.

1. All properties and OPS components are within expected ranges.
2. Unknown ECFP_2 feature: -1508366470: [*][n+](:[*]):[*]
3. Unknown ECFP_2 feature: 1508268466: [*]C[n+](:[c]([*]):[*]):c:[*]
4. Unknown ECFP_2 feature: -1333923932: [*]CC[n+](:[*]):[*]
5. Unknown ECFP_2 feature: 2078070048: [*][n+](:[*]):[c]([c](:[*]):[*]):c:[*]
6. Unknown ECFP_2 feature: 688916016: [*][n+](:[*]):c:[c](:[*]):[*]
7. Unknown FCFP_6 feature: 24: [*][n+](:[*]):[*]
8. Unknown FCFP_6 feature: 16: [*]:[cH]:[*]
9. Unknown FCFP_6 feature: 414371600: [*]C[n+](:[cH]:[*]):[c]([*]):[*]
10. Unknown FCFP_6 feature: -150573739: [*]CC[n+](:[*]):[*]
11. Unknown FCFP_6 feature: -1861407456: [*][n+](:[*]):[c](:[cH]:[*])[c](:[*]):[*]
12. Unknown FCFP_6 feature: -1861645784: [*][c](:[*]):[c](:[cH]:[*])[c](:[*]):[*]
13. Unknown FCFP_6 feature: 1618392993: [*][n+](:[*]):[cH]:[c](:[*]):[*]
14. Unknown FCFP_6 feature: 1618154665: [*]:[cH]:[cH]:[cH]:[*]

Feature Contribution

Top features for positive contribution			
Fingerprint	Bit/Smiles	Feature Structure	Score

ECFP_6	642810091	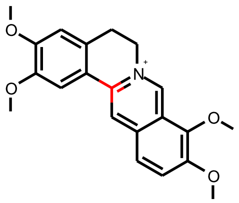 [*]:[c](:[*]):[*]	0.281
FCFP_6	136627117	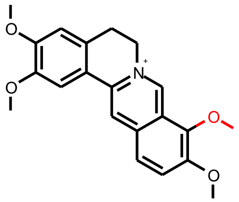 [*]OC	0.17
ECFP_6	1334973442	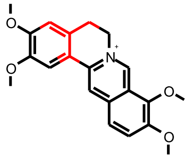 [*]C[c](:[cH]:[*]):[c]([*]):[*]	0.15

Top Features for negative contribution

**Fingerprint**	**Bit/Smiles**	**Feature Structure**	**Score**
FCFP_6	1676877079	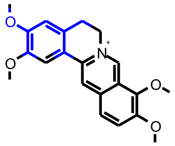 [*]C[c]1:[cH]:[c](O[*]):[c]([*]):[*]:[c]:1[*]	-0.254
ECFP_6	2077607946	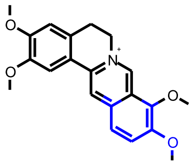 [*]O[c]1:[cH]:[cH]:[c](:[*]):[*]:[c]:1[*]	-0.252

ECFP_6	734603939	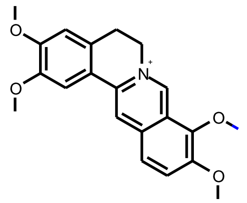 [*]C	-0.201

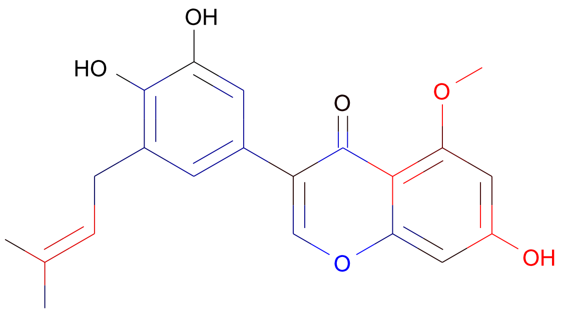

$C_{21}H_{20}O_6$
Molecular Weight: 368.3799
ALogP: 3.98
Rotatable Bonds: 4
Acceptors: 6
Donors: 3

Model Prediction

Prediction: 0.337
Unit: g/kg_body_weight
Mahalanobis Distance: 20
Mahalanobis Distance p-value: 8.53e-009

Mahalanobis Distance: The Mahalanobis distance (MD) is a generalization of the Euclidean distance that accounts for correlations among the X properties. It is calculated as the distance to the center of the training data. The larger the MD, the less trustworthy the prediction.
Mahalanobis Distance p-value: The p-value gives the fraction of training data with an MD greater than or equal to the one for the given sample, assuming normally distributed data. The smaller the p-value, the less trustworthy the prediciton. For highly non-normal X properties (e.g., fingerprints), the MD p-value is wildly inaccurate.

Structural Similar Compounds

Name	OCHRATOXIN A	DICOUMAROL	FLUORESCEINE; SODIUM SALT (Na STRIPPED)
Structure	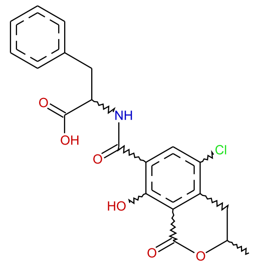	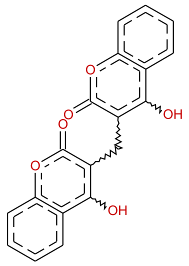	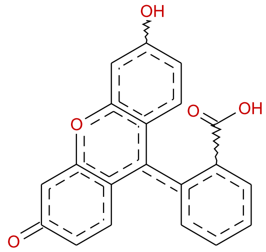
Actual Endpoint (-log C)	4.305	3.129	1.694
Predicted Endpoint (-log C)	3.03558	2.86156	2.71831
Distance	0.542	0.593	0.601
Reference	FCTXAV 6;479;68	SMWOAS 83;471;53	JOPRAJ 48;228;77

Model Applicability

Unknown features are fingerprint features in the query molecule, but not found or appearing too infrequently in the training set.

1. All properties and OPS components are within expected ranges.
2. Unknown ECFP_2 feature: 1717082529: [*]\C=C(/C(=[*])[*])\[c](:[*]):[*]
3. Unknown FCFP_6 feature: 16: [*]:[cH]:[*]
4. Unknown FCFP_6 feature: 74595001: [*][c](:[*]):[c](O):[cH]:[*]
5. Unknown FCFP_6 feature: 1618154665: [*]:[cH]:[cH]:[cH]:[*]
6. Unknown FCFP_6 feature: -1678275541: [*]\C=C(/C(=[*])[*])\[c](:[*]):[*]
7. Unknown FCFP_6 feature: -549108873: [*]:[c](:[*])O

Feature Contribution

Top features for positive contribution			
**Fingerprint**	**Bit/Smiles**	**Feature Structure**	**Score**

ECFP_6	642810091	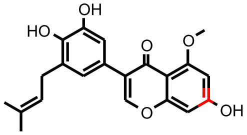 [*]:[c](:[*]):[*]	0.281
FCFP_6	136627117	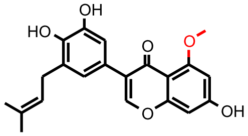 [*]OC	0.17
FCFP_6	-1549192822	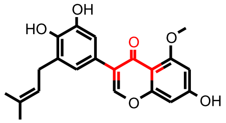 [*]C(=[*])C(=O)[c](:[*]):[*]	0.168
**Top Features for negative contribution**			
**Fingerprint**	**Bit/Smiles**	**Feature Structure**	**Score**
ECFP_6	2106656448	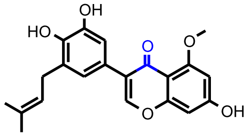 [*]C(=O)[*]	-0.352
ECFP_6	683445015	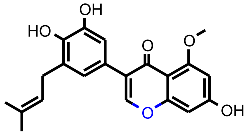 [*]O[*]	-0.266

ECFP_6	734603939	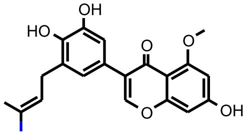 [*]C	-0.201

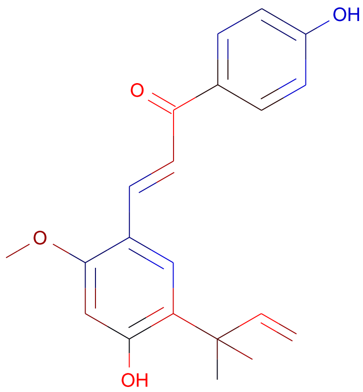

$C_{21}H_{22}O_4$
Molecular Weight: 338.39698
ALogP: 4.667
Rotatable Bonds: 6
Acceptors: 4
Donors: 2

Structural Similar Compounds

Name	BEZAFIBRATE	TERBUFIBROL	CARBANILIC ACID; m-HYDROXY-; METHYL ESTER; m-METHYLCARBANILATE
Structure	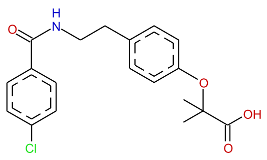	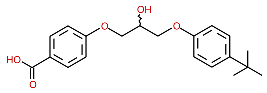	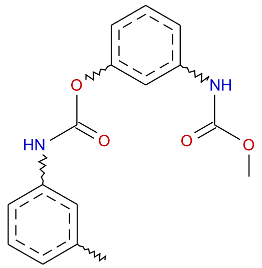
Actual Endpoint (-log C)	1.946	2.157	1.876
Predicted Endpoint (-log C)	2.54395	2.06158	1.81916
Distance	0.495	0.503	0.549
Reference	ARZNAD 30;2023;80	DRFUD4 4;140;79	GISAAA 49(4);16;84

Model Prediction

Prediction: 0.406
Unit: g/kg_body_weight
Mahalanobis Distance: 15.8
Mahalanobis Distance p-value: 0.397

Mahalanobis Distance: The Mahalanobis distance (MD) is a generalization of the Euclidean distance that accounts for correlations among the X properties. It is calculated as the distance to the center of the training data. The larger the MD, the less trustworthy the prediction.
Mahalanobis Distance p-value: The p-value gives the fraction of training data with an MD greater than or equal to the one for the given sample, assuming normally distributed data. The smaller the p-value, the less trustworthy the prediciton. For highly non-normal X properties (e.g., fingerprints), the MD p-value is wildly inaccurate.

Model Applicability

Unknown features are fingerprint features in the query molecule, but not found or appearing too infrequently in the training set.

1. All properties and OPS components are within expected ranges.
2. Unknown ECFP_2 feature: -144557007: [*]=CC(C)(C)[c](:[*]):[*]
3. Unknown FCFP_6 feature: 16: [*]:[cH]:[*]
4. Unknown FCFP_6 feature: -549108873: [*]:[c](:[*])O
5. Unknown FCFP_6 feature: 1618154665: [*]:[cH]:[cH]:[cH]:[*]
6. Unknown FCFP_6 feature: 74595001: [*][c](:[*]):[c](O):[cH]:[*]
7. Unknown FCFP_6 feature: 451371068: [*]\C=C\[c](:[*]):[*]

Feature Contribution

Top features for positive contribution			
Fingerprint	Bit/Smiles	Feature Structure	Score

ECFP_6	642810091	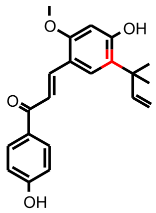 [*]:[c](:[*]):[*]	0.281
FCFP_6	136627117	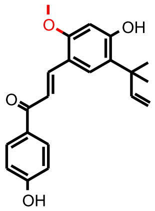 [*]OC	0.17
FCFP_6	-1549192822	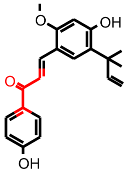 [*]C(=[*])C(=O)[c](:[*]):[*]	0.168

Top Features for negative contribution

Fingerprint	Bit/Smiles	Feature Structure	Score
FCFP_6	946589555	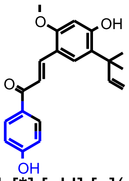 [*][c]1:[*]:[cH]:[c](O):[cH]:[cH]:1	-0.204
ECFP_6	734603939	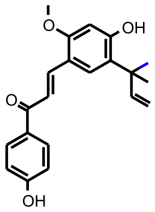 [*]C	-0.201

FCFP_6	-451251206	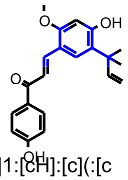<br>[*]C[c]1:[cH]:[c](:[cH]:[*]:[c]:1[*])C(=[*])[*]	-0.143

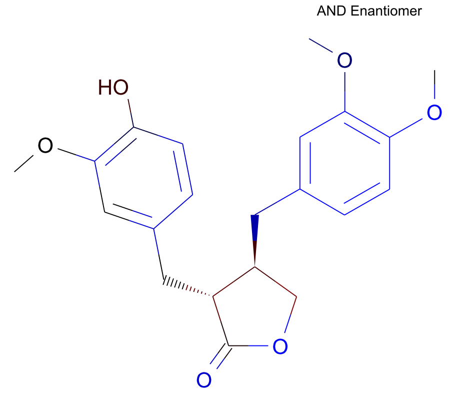

$C_{21}H_{24}O_6$
Molecular Weight: 372.41166
ALogP: 3.743
Rotatable Bonds: 7
Acceptors: 6
Donors: 1

Model Prediction

Prediction: 12.4
Unit: g/kg_body_weight
Mahalanobis Distance: 21.9
Mahalanobis Distance p-value: 8.3e-016

Mahalanobis Distance: The Mahalanobis distance (MD) is a generalization of the Euclidean distance that accounts for correlations among the X properties. It is calculated as the distance to the center of the training data. The larger the MD, the less trustworthy the prediction.
Mahalanobis Distance p-value: The p-value gives the fraction of training data with an MD greater than or equal to the one for the given sample, assuming normally distributed data. The smaller the p-value, the less trustworthy the prediciton. For highly non-normal X properties (e.g., fingerprints), the MD p-value is wildly inaccurate.

Structural Similar Compounds

Name	3-QUINOLINECARBOXYLIC ACID; 6;7-bis-(CYCLOPROPYLMETHOXY)-4-HYDROXY-; ETHYL ESTER	KETONE; 10-[3-[4-(2-HYDROXYETHYL)PIPERIDINO]PROPYL]PHENOTHIAZIN-2-YL METHYL	CLOXYPENDYL
Structure	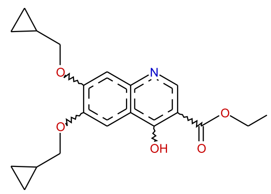	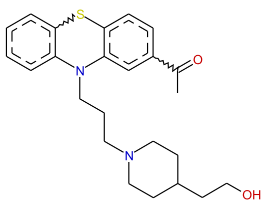	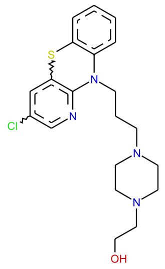
Actual Endpoint (-log C)	2.076	3.022	2.79
Predicted Endpoint (-log C)	2.50101	2.45974	2.59858
Distance	0.465	0.532	0.547
Reference	TXAPA9 18;185;71	TXAPA9 5;49;63	ARZNAD 18;435;68

Model Applicability

Unknown features are fingerprint features in the query molecule, but not found or appearing too infrequently in the training set.

1. All properties and OPS components are within expected ranges.
2. Unknown FCFP_6 feature: 16: [*]:[cH]:[*]
3. Unknown FCFP_6 feature: -549108873: [*]:[c](:[*])O
4. Unknown FCFP_6 feature: 1618154665: [*]:[cH]:[cH]:[cH]:[*]
5. Unknown FCFP_6 feature: 74595001: [*][c](:[*]):[c](O):[cH]:[*]

Feature Contribution

Top features for positive contribution

Fingerprint	Bit/Smiles	Feature Structure	Score

ECFP_6	642810091	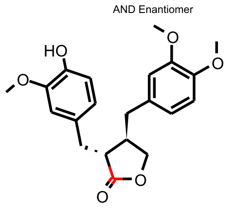 [*]:[c](:[*]):[*]	0.281
FCFP_6	136627117	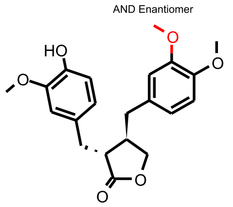 [*]OC	0.17
ECFP_6	-1074141656	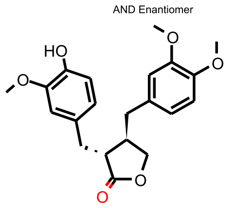 [*]=O	0.142
**Top Features for negative contribution**			
**Fingerprint**	**Bit/Smiles**	**Feature Structure**	**Score**
ECFP_6	2106656448	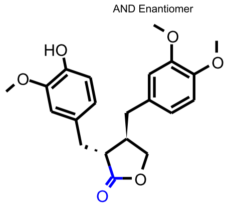 [*]C(=O)[*]	-0.352
ECFP_6	683445015	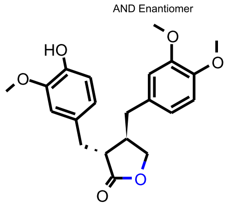 [*]O[*]	-0.266

FCFP_6	1676877079	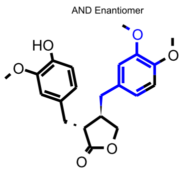<br>[*]C[c]1:[cH]:[c](O[*]):[c]([*]):[*]:[c]:1[*]	-0.254

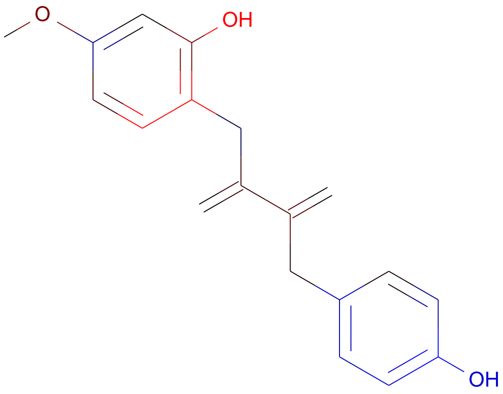

$C_{19}H_{20}O_3$
Molecular Weight: 296.3603
ALogP: 4.784
Rotatable Bonds: 6
Acceptors: 3
Donors: 2

Model Prediction

Prediction: 2.65
Unit: g/kg_body_weight
Mahalanobis Distance: 16.3
Mahalanobis Distance p-value: 0.169

Mahalanobis Distance: The Mahalanobis distance (MD) is a generalization of the Euclidean distance that accounts for correlations among the X properties. It is calculated as the distance to the center of the training data. The larger the MD, the less trustworthy the prediction.
Mahalanobis Distance p-value: The p-value gives the fraction of training data with an MD greater than or equal to the one for the given sample, assuming normally distributed data. The smaller the p-value, the less trustworthy the prediciton. For highly non-normal X properties (e.g., fingerprints), the MD p-value is wildly inaccurate.

Structural Similar Compounds

Name	DICLOFENAC	FLUFENAMIC ACID	ITANOXONE
Structure	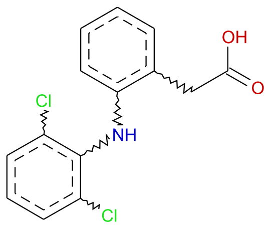	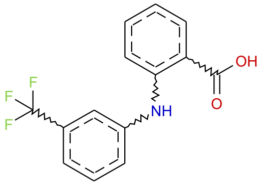	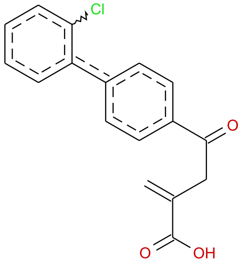
Actual Endpoint (-log C)	3.676	3.053	2.098
Predicted Endpoint (-log C)	2.72031	2.26897	2.22753
Distance	0.515	0.556	0.573
Reference	ARZNAD 30;1398;80	AIPTAK 221;132;76	EJMCA5 22;45;87

Model Applicability

Unknown features are fingerprint features in the query molecule, but not found or appearing too infrequently in the training set.

1. All properties and OPS components are within expected ranges.
2. Unknown FCFP_6 feature: 16: [*]:[cH]:[*]
3. Unknown FCFP_6 feature: -549108873: [*]:[c](:[*])O
4. Unknown FCFP_6 feature: 74595001: [*][c](:[*]):[c](O):[cH]:[*]
5. Unknown FCFP_6 feature: 1618154665: [*]:[cH]:[cH]:[cH]:[*]

Feature Contribution

Top features for positive contribution

Fingerprint	Bit/Smiles	Feature Structure	Score
ECFP_6	642810091	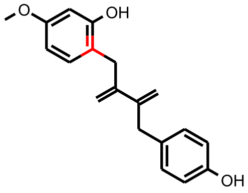 [*]:[c](:[*]):[*]	0.281

FCFP_6	136627117	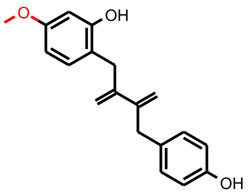 [*]OC	0.17
ECFP_6	2019062761	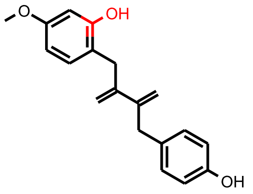 [*]:[c](:[*])O	0.138
**Top Features for negative contribution**			
**Fingerprint**	**Bit/Smiles**	**Feature Structure**	**Score**
ECFP_6	-176455838	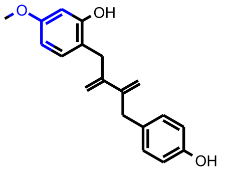 [*]O[c](:[cH]:[*]):[cH]:[*]	-0.257
FCFP_6	946589555	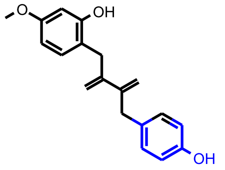 [*][c]1:[*]:[cH]:[c](O):[cH]:[cH]:1	-0.204
ECFP_6	734603939	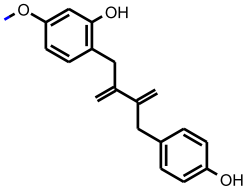 [*]C	-0.201

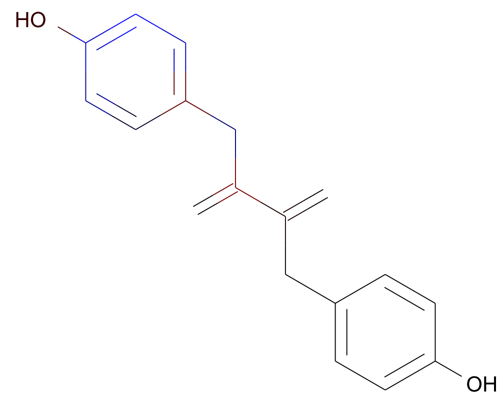

$C_{18}H_{18}O_2$

Molecular Weight: 266.33432

ALogP: 4.8

Rotatable Bonds: 5

Acceptors: 2

Donors: 2

Structural Similar Compounds

Name	4;4'-DIHYDROXYDIPHENYLMETHANE	BISPHENOL A	DICHLOROPHENE
Structure	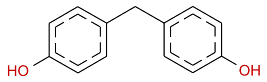	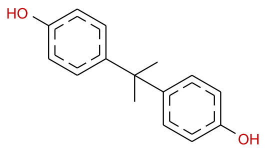	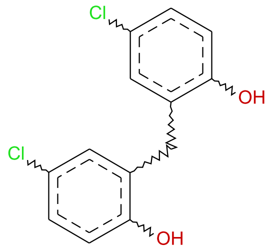
Actual Endpoint (-log C)	1.607	1.847	2.252
Predicted Endpoint (-log C)	1.8824	1.65879	2.30751
Distance	0.483	0.496	0.507
Reference	AIHAAP 23;95;62	AIHAAP 28;301;67	FAATDF 7;299;86

Model Prediction

Prediction: 2.87

Unit: g/kg_body_weight

Mahalanobis Distance: 15

Mahalanobis Distance p-value: 0.787

Mahalanobis Distance: The Mahalanobis distance (MD) is a generalization of the Euclidean distance that accounts for correlations among the X properties. It is calculated as the distance to the center of the training data. The larger the MD, the less trustworthy the prediction.

Mahalanobis Distance p-value: The p-value gives the fraction of training data with an MD greater than or equal to the one for the given sample, assuming normally distributed data. The smaller the p-value, the less trustworthy the prediciton. For highly non-normal X properties (e.g., fingerprints), the MD p-value is wildly inaccurate.

Model Applicability

Unknown features are fingerprint features in the query molecule, but not found or appearing too infrequently in the training set.

1. All properties and OPS components are within expected ranges.
2. Unknown FCFP_6 feature: 16: [*]:[cH]:[*]
3. Unknown FCFP_6 feature: -549108873: [*]:[c](:[*])O
4. Unknown FCFP_6 feature: 1618154665: [*]:[cH]:[cH]:[cH]:[*]
5. Unknown FCFP_6 feature: 74595001: [*][c](:[*]):[c](O):[cH]:[*]

Feature Contribution

Top features for positive contribution

Fingerprint	Bit/Smiles	Feature Structure	Score
ECFP_6	642810091	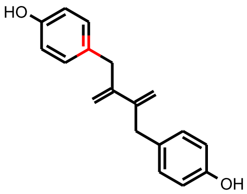 [*]:[c](:[*]):[*]	0.281

ECFP_6	2019062761	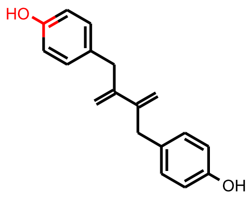 [*]:[c](:[*])O	0.138
FCFP_6	7	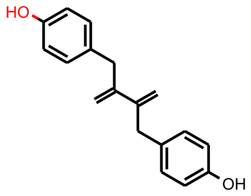 [*]O	0.0882
**Top Features for negative contribution**			
**Fingerprint**	**Bit/Smiles**	**Feature Structure**	**Score**
FCFP_6	946589555	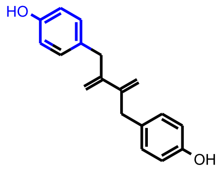 [*][c]1:[*]:[cH]:[c](O):[cH]:[cH]:1	-0.204
FCFP_6	0	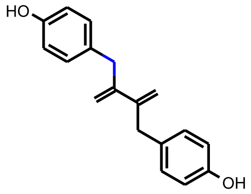 [*]C([*])[*]	-0.0791
ECFP_6	1088861418	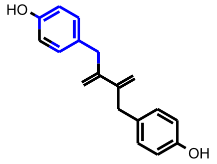 [*]C[c]1:[cH]:[*]:[c] ([*]):[cH]:[cH]:1	-0.0677

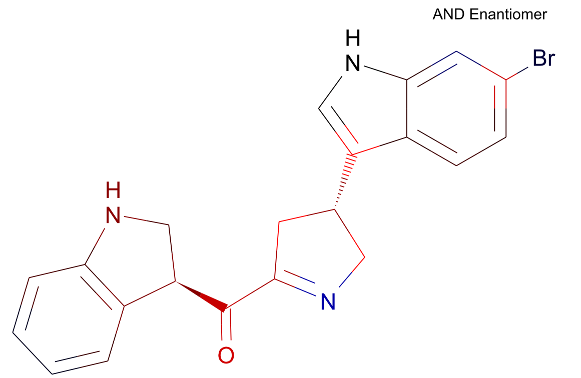

$C_{21}H_{18}BrN_3O$
Molecular Weight: 408.29112
ALogP: 3.919
Rotatable Bonds: 3
Acceptors: 3
Donors: 2

Model Prediction

Prediction: 0.264
Unit: g/kg_body_weight
Mahalanobis Distance: 24.4
Mahalanobis Distance p-value: 1.34e-027

Mahalanobis Distance: The Mahalanobis distance (MD) is a generalization of the Euclidean distance that accounts for correlations among the X properties. It is calculated as the distance to the center of the training data. The larger the MD, the less trustworthy the prediction.
Mahalanobis Distance p-value: The p-value gives the fraction of training data with an MD greater than or equal to the one for the given sample, assuming normally distributed data. The smaller the p-value, the less trustworthy the prediciton. For highly non-normal X properties (e.g., fingerprints), the MD p-value is wildly inaccurate.

Structural Similar Compounds

Name	TRIARIMOL	.alpha.-(2-CHLOROPHENYL)-.alpha.-(4-CHLOROPHENYL)-5-PYRIMIDINE METHANOL	CARPROFEN
Structure	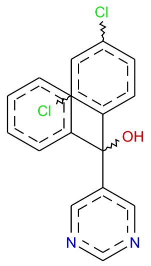	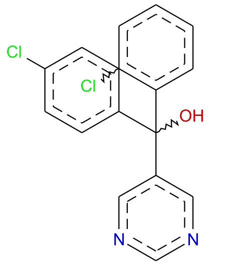	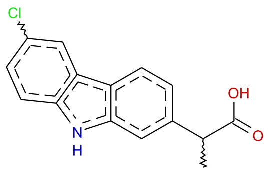
Actual Endpoint (-log C)	2.742	2.122	3.568
Predicted Endpoint (-log C)	2.87257	2.72534	3.46422
Distance	0.633	0.634	0.642
Reference	FMCHA2 -;C293;89	FMCHA2 -;C254;89	OYYAA2 14;251;77

Model Applicability

Unknown features are fingerprint features in the query molecule, but not found or appearing too infrequently in the training set.

1. All properties and OPS components are within expected ranges.
2. Unknown ECFP_2 feature: 103000222: [*]C(=[*])C1=N[*][*]C1
3. Unknown ECFP_2 feature: 1431365708: [*]C([*])C(=O)C(=[*])[*]
4. Unknown FCFP_6 feature: 16: [*]:[cH]:[*]
5. Unknown FCFP_6 feature: 19: [*]:[nH]:[*]
6. Unknown FCFP_6 feature: 1618154665: [*]:[cH]:[cH]:[cH]:[*]
7. Unknown FCFP_6 feature: 307448885: [*]:[cH]:[c]1:[nH]:[*]:[*]:[c]:1:[*]
8. Unknown FCFP_6 feature: 1618184456: [*][c]1:[*]:[*]:[nH]:[cH]:1
9. Unknown FCFP_6 feature: 2005402822: [*]:[c]1:[*]:[*]:[c](:[*]):[nH]:1
10. Unknown FCFP_6 feature: 71476542: [*]:[c](:[*])Br

Feature Contribution

Top features for positive contribution

Fingerprint	Bit/Smiles	Feature Structure	Score

FCFP_6	1186303932	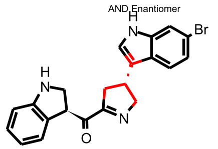 [*]:[c](:[*])C1C[*]=[*]C1	0.375
ECFP_6	642810091	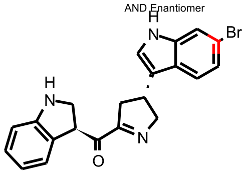 [*]:[c](:[*]):[*]	0.281
ECFP_6	-1074141656	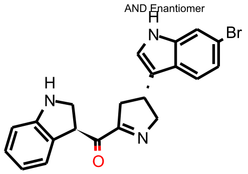 [*]=O	0.142

Top Features for negative contribution

Fingerprint	Bit/Smiles	Feature Structure	Score
ECFP_6	655739385	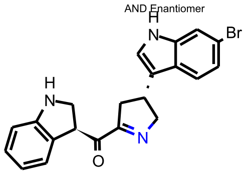 [*]:n:[*]	-0.239
FCFP_6	3	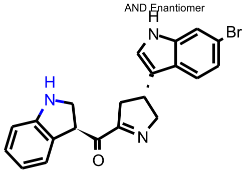 [*]N[*]	-0.107

ECFP_6	-302078100	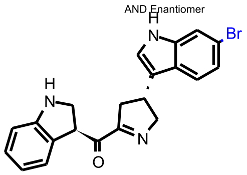 [*]Br	-0.103

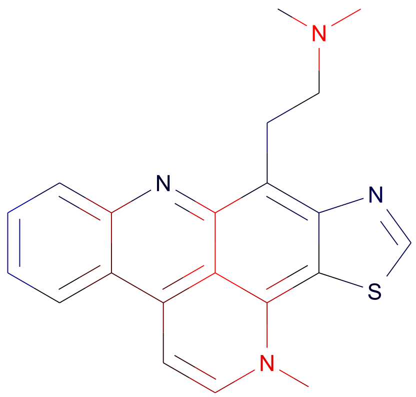

$C_{21}H_{20}N_4S$
Molecular Weight: 360.4753
ALogP: 3.682
Rotatable Bonds: 3
Acceptors: 4
Donors: 0

Model Prediction

Prediction: 0.461
Unit: g/kg_body_weight
Mahalanobis Distance: 24.5
Mahalanobis Distance p-value: 2.36e-028

Mahalanobis Distance: The Mahalanobis distance (MD) is a generalization of the Euclidean distance that accounts for correlations among the X properties. It is calculated as the distance to the center of the training data. The larger the MD, the less trustworthy the prediction.
Mahalanobis Distance p-value: The p-value gives the fraction of training data with an MD greater than or equal to the one for the given sample, assuming normally distributed data. The smaller the p-value, the less trustworthy the prediciton. For highly non-normal X properties (e.g., fingerprints), the MD p-value is wildly inaccurate.

Structural Similar Compounds

Name	ETIZOLAM	ALPRAZOLAM	TYLOCREBRINE
Structure	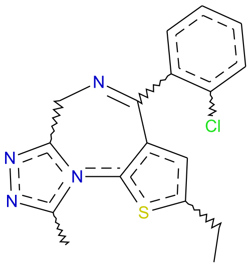	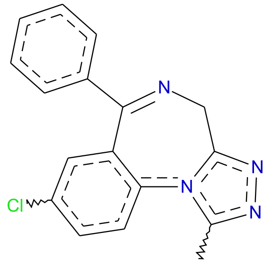	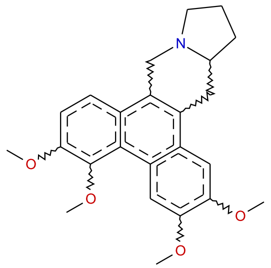
Actual Endpoint (-log C)	1.99	2.403	3.782
Predicted Endpoint (-log C)	2.85244	3.03888	3.12068
Distance	0.658	0.688	0.694
Reference	ARZNAD 28;1158;78	YACHDS 8;4695;80	NCICP* -;77;64

Model Applicability

Unknown features are fingerprint features in the query molecule, but not found or appearing too infrequently in the training set.

1. All properties and OPS components are within expected ranges.
2. Unknown ECFP_2 feature: 1618095312: [*]=CN(C)[c](:[*]):[*]
3. Unknown ECFP_2 feature: -1673960248: [*][c](:[*]):[c]1:s:[*]:[*]:[c]:1:[*]
4. Unknown FCFP_6 feature: 16: [*]:[cH]:[*]
5. Unknown FCFP_6 feature: 1747237384: [*][c](:[*]):n:[cH]:[*]
6. Unknown FCFP_6 feature: 451371068: [*]\C=C\[c](:[*]):[*]
7. Unknown FCFP_6 feature: 1618154665: [*]:[cH]:[cH]:[cH]:[*]
8. Unknown FCFP_6 feature: -124685461: [*]1:[*]:s:[cH]:n:1

Feature Contribution

Top features for positive contribution

Fingerprint	Bit/Smiles	Feature Structure	Score

ECFP_6	865379614	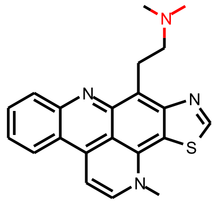 [*]N([*])C	0.322
ECFP_6	642810091	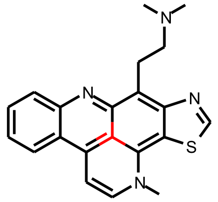 [*]:[c](:[*]):[*]	0.281
ECFP_6	-1789942192	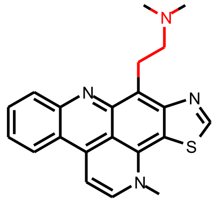 [*]CCN([*])[*]	0.196
**Top Features for negative contribution**			
**Fingerprint**	**Bit/Smiles**	**Feature Structure**	**Score**
ECFP_6	655739385	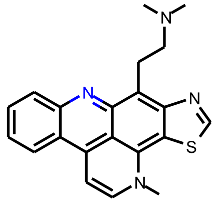 [*]:n:[*]	-0.239
ECFP_6	734603939	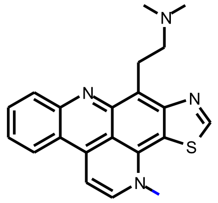 [*]C	-0.201

ECFP_6	-1795525632	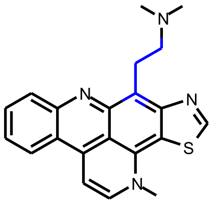 [*]CC[c](:[*]):[*]	-0.176

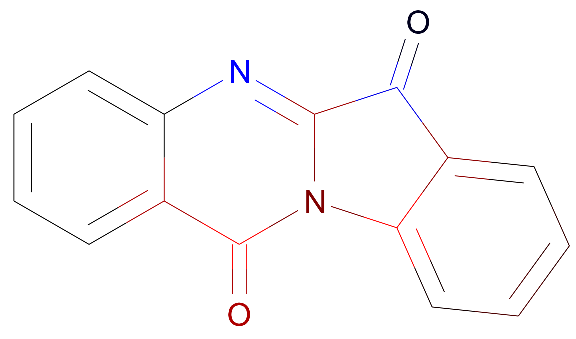

$C_{15}H_8N_2O_2$
Molecular Weight: 248.23621
ALogP: 2.331
Rotatable Bonds: 0
Acceptors: 3
Donors: 0

Model Prediction

Prediction: 0.604
Unit: g/kg_body_weight
Mahalanobis Distance: 20.6
Mahalanobis Distance p-value: 8.25e-011

Mahalanobis Distance: The Mahalanobis distance (MD) is a generalization of the Euclidean distance that accounts for correlations among the X properties. It is calculated as the distance to the center of the training data. The larger the MD, the less trustworthy the prediction.
Mahalanobis Distance p-value: The p-value gives the fraction of training data with an MD greater than or equal to the one for the given sample, assuming normally distributed data. The smaller the p-value, the less trustworthy the prediciton. For highly non-normal X properties (e.g., fingerprints), the MD p-value is wildly inaccurate.

Structural Similar Compounds

Name	METHAQUALONE	4H-3;1-BENZOXAZIN-4-ONE; 2-PHENYL-	7H-FURO[3;2-g][1]BENZOPYRAN-7-ONE; 9-METHOXY-
Structure	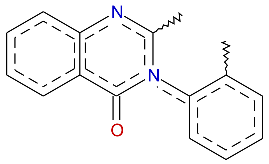	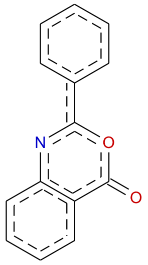	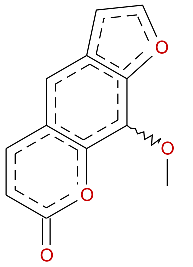
Actual Endpoint (-log C)	3.037	2.145	2.437
Predicted Endpoint (-log C)	2.51053	2.09073	2.99348
Distance	0.391	0.434	0.489
Reference	ARZNAD 17;229;67	FMCHA2 -;C41;89	DCTODJ 2;309;79

Model Applicability

Unknown features are fingerprint features in the query molecule, but not found or appearing too infrequently in the training set.

1. All properties and OPS components are within expected ranges.
2. Unknown FCFP_6 feature: 16: [*]:[cH]:[*]
3. Unknown FCFP_6 feature: 580453787: [*]C(=N[c](:[*]):[*])[*]
4. Unknown FCFP_6 feature: 1618154665: [*]:[cH]:[cH]:[cH]:[*]

Feature Contribution

Top features for positive contribution

Fingerprint	Bit/Smiles	Feature Structure	Score
ECFP_6	642810091	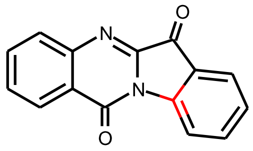 [*]:[c](:[*]):[*]	0.281

FCFP_6	-1549163031	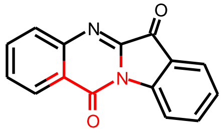 [*]N([*])C(=O)[c](:[*]):[*]	0.171
FCFP_6	-1549192822	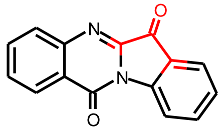 [*]C(=[*])C(=O)[c](:[*]):[*]	0.168

Top Features for negative contribution

Fingerprint	Bit/Smiles	Feature Structure	Score
ECFP_6	2106656448	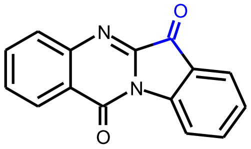 [*]C(=O)[*]	-0.352
ECFP_6	655739385	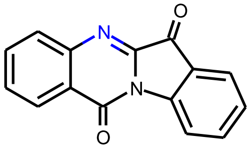 [*]:n:[*]	-0.239
ECFP_6	-1236953626	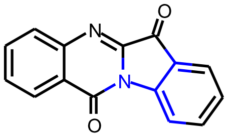 [*]N1[*][*][c](:[*]):[c]1:[cH]:[*]	-0.11

AND Enantiomer

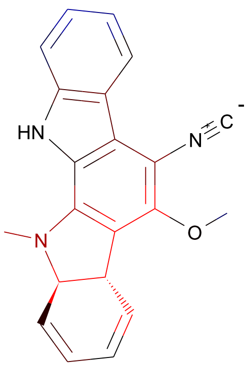

$C_{21}H_{17}N_3O$

Molecular Weight: 327.37918

ALogP: 4.078

Rotatable Bonds: 1

Acceptors: 2

Donors: 1

Model Prediction

Prediction: 0.146

Unit: g/kg_body_weight

Mahalanobis Distance: 23.9

Mahalanobis Distance p-value: 2.18e-025

Mahalanobis Distance: The Mahalanobis distance (MD) is a generalization of the Euclidean distance that accounts for correlations among the X properties. It is calculated as the distance to the center of the training data. The larger the MD, the less trustworthy the prediction.
Mahalanobis Distance p-value: The p-value gives the fraction of training data with an MD greater than or equal to the one for the given sample, assuming normally distributed data. The smaller the p-value, the less trustworthy the prediciton. For highly non-normal X properties (e.g., fingerprints), the MD p-value is wildly inaccurate.

Structural Similar Compounds

Name	BENZOPHENONE; 4'-CHLORO-2-(2-IMIDAZOL-2-YL)-	.alpha.-(2-CHLOROPHENYL)-.alpha.-(4-CHLOROPHENYL)-5-PYRIMIDINE METHANOL	TRIARIMOL
Structure	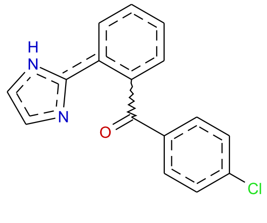	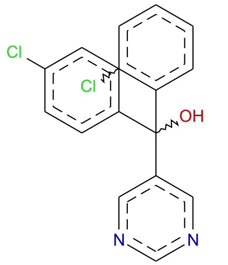	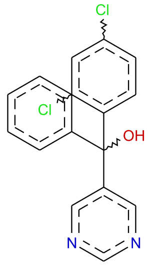
Actual Endpoint (-log C)	2.673	2.122	2.742
Predicted Endpoint (-log C)	3.18028	2.72534	2.87257
Distance	0.554	0.567	0.568
Reference	JMCMAR 18;182;75	FMCHA2 -;C254;89	FMCHA2 -;C293;89

Model Applicability

Unknown features are fingerprint features in the query molecule, but not found or appearing too infrequently in the training set.

1. All properties and OPS components are within expected ranges.
2. Unknown ECFP_2 feature: 1029014155: [*][N+]#[*]
3. Unknown ECFP_2 feature: 726108635: [*]#[C-]
4. Unknown ECFP_2 feature: 1464683384: [*][c](:[*]):[c]([N+]#[*]):[c](:[*]):[*]
5. Unknown ECFP_2 feature: -1337975340: [*][C@H]1[*]:[*]N([*])[C@@H]1C=[*]
6. Unknown ECFP_2 feature: -11961319: [*]:[c](:[*])[N+]#[C-]
7. Unknown ECFP_2 feature: -1334780583: [*][N+]#[C-]
8. Unknown FCFP_6 feature: 16: [*]:[cH]:[*]
9. Unknown FCFP_6 feature: 19: [*]:[nH]:[*]
10. Unknown FCFP_6 feature: 8: [*][N+]#[*]
11. Unknown FCFP_6 feature: 4: [*]#[C-]
12. Unknown FCFP_6 feature: -828984032: [*][c](:[*]):[c]([N+]#[*]):[c](:[*]):[*]
13. Unknown FCFP_6 feature: 307448885: [*]:[cH]:[c]1:[nH]:[*]:[*]:[c]:1:[*]
14. Unknown FCFP_6 feature: 2005402822: [*]:[c]1:[*]:[*]:[c](:[*]):[nH]:1
15. Unknown FCFP_6 feature: 1618154665: [*]:[cH]:[cH]:[cH]:[*]
16. Unknown FCFP_6 feature: 1934974835: [*]:[c](:[*])[N+]#[C-]
17. Unknown FCFP_6 feature: -1487147388: [*][N+]#[C-]

Feature Contribution

Top features for positive contribution			
**Fingerprint**	**Bit/Smiles**	**Feature Structure**	**Score**
FCFP_6	1186303932	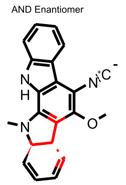 [*]:[c](:[*])C1C[*]=[*]C1	0.375
ECFP_6	642810091	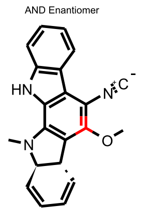 [*]:[c](:[*]):[*]	0.281
FCFP_6	136627117	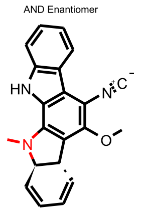 [*]OC	0.17

Top Features for negative contribution			
**Fingerprint**	**Bit/Smiles**	**Feature Structure**	**Score**
ECFP_6	734603939	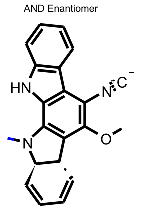 [*]C	-0.201

FCFP_6	1036089772	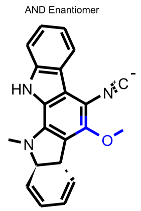<br><br>[*]:[c](:[*])OC	-0.136
ECFP_6	864909220	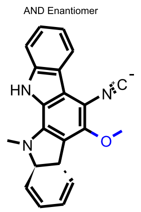<br><br>[*]OC	-0.119

AND Enantiomer

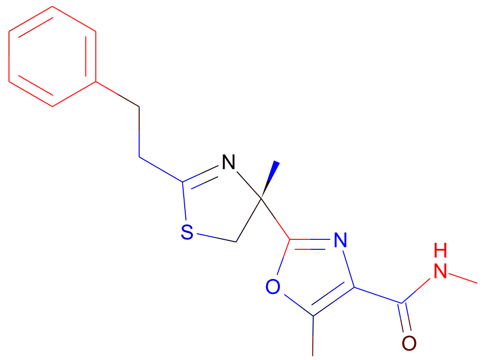

$C_{18}H_{21}N_3O_2S$

Molecular Weight: 343.44324

ALogP: 2.716

Rotatable Bonds: 5

Acceptors: 4

Donors: 1

Model Prediction

Prediction: 1.1

Unit: g/kg_body_weight

Mahalanobis Distance: 28

Mahalanobis Distance p-value: 1.56e-049

Mahalanobis Distance: The Mahalanobis distance (MD) is a generalization of the Euclidean distance that accounts for correlations among the X properties. It is calculated as the distance to the center of the training data. The larger the MD, the less trustworthy the prediction.
Mahalanobis Distance p-value: The p-value gives the fraction of training data with an MD greater than or equal to the one for the given sample, assuming normally distributed data. The smaller the p-value, the less trustworthy the prediciton. For highly non-normal X properties (e.g., fingerprints), the MD p-value is wildly inaccurate.

Structural Similar Compounds

Name	GLYBUZOLE	DIFLUMIDONE	PROPERICIAZINE
Structure	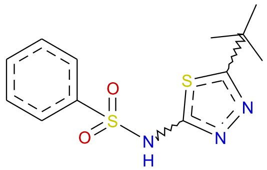	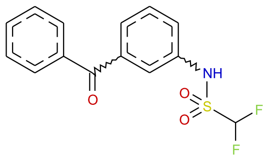	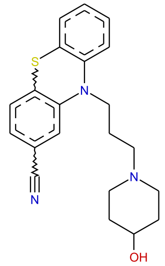
Actual Endpoint (-log C)	2.774	2.618	2.966
Predicted Endpoint (-log C)	2.33197	2.88827	2.66806
Distance	0.560	0.563	0.566
Reference	YIKUAO 18;21;69	AIPTAK 221;132;76	TXAPA9 21;315;72

Model Applicability

Unknown features are fingerprint features in the query molecule, but not found or appearing too infrequently in the training set.

1. All properties and OPS components are within expected ranges.
2. Unknown ECFP_2 feature: 309047694: [*]C([*])([*])[c]1:o:[*]:[*]:n:1
3. Unknown ECFP_2 feature: -1073216586: [*]CC1=N[*][*]S1
4. Unknown ECFP_2 feature: 1920241679: [*]C1([*])[*]=[*]SC1
5. Unknown ECFP_2 feature: 618128563: [*]:[c](:[*])[C@]1(C)C[*][*]=N1
6. Unknown FCFP_6 feature: 16: [*]:[cH]:[*]
7. Unknown FCFP_6 feature: 1747237384: [*][c](:[*]):n:[cH]:[*]
8. Unknown FCFP_6 feature: -1539162406: [*]C([*])([*])[c]1:o:[*]:[*]:n:1
9. Unknown FCFP_6 feature: -836603894: [*]:[c](:[*])[C@]1(C)C[*][*]=N1
10. Unknown FCFP_6 feature: 1618154665: [*]:[cH]:[cH]:[cH]:[*]
11. Unknown FCFP_6 feature: 136686699: [*]NC

Feature Contribution

Top features for positive contribution			
Fingerprint	Bit/Smiles	Feature Structure	Score

ECFP_6	642810091	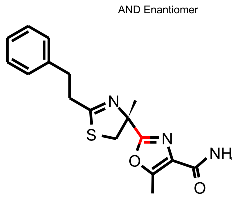 [*]:[c](:[*]):[*]	0.281
ECFP_6	-1897341097	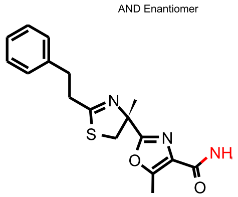 [*]N[*]	0.216
ECFP_6	1571214559	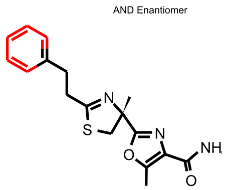 [*]1:[cH]:[cH]:[cH]:[cH]:[cH]:1	0.19
**Top Features for negative contribution**			
**Fingerprint**	**Bit/Smiles**	**Feature Structure**	**Score**
ECFP_6	683445015	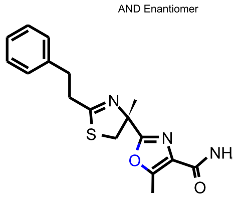 [*]O[*]	-0.266
ECFP_6	655739385	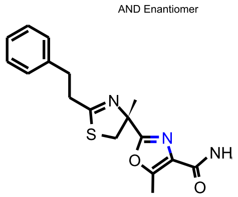 [*]:n:[*]	-0.239

ECFP_6	734603939	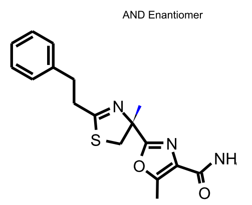<br>[*]C	-0.201

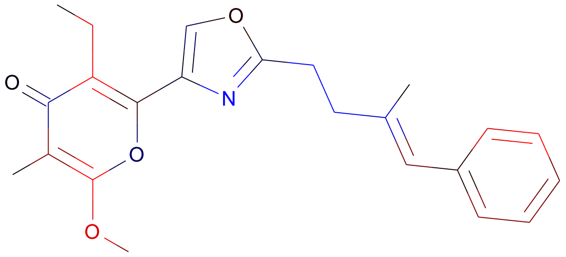

$C_{23}H_{25}NO_4$
Molecular Weight: 379.4489
ALogP: 5.22
Rotatable Bonds: 7
Acceptors: 4
Donors: 0

Model Prediction

Prediction: 1.02
Unit: g/kg_body_weight
Mahalanobis Distance: 21.6
Mahalanobis Distance p-value: 8.51e-015

Mahalanobis Distance: The Mahalanobis distance (MD) is a generalization of the Euclidean distance that accounts for correlations among the X properties. It is calculated as the distance to the center of the training data. The larger the MD, the less trustworthy the prediction.
Mahalanobis Distance p-value: The p-value gives the fraction of training data with an MD greater than or equal to the one for the given sample, assuming normally distributed data. The smaller the p-value, the less trustworthy the prediciton. For highly non-normal X properties (e.g., fingerprints), the MD p-value is wildly inaccurate.

Structural Similar Compounds

Name	CYPERMETHRIN;TRANS	2;2;3;3-TETRAMETHYLCYCLOPROPANECARBOXYLIC ACID; CYANO(3-PHENOXYPHENYL)METHYL ESTER	2-CHLORO-1-[(3-ETHOXY-4-NITROPHENOXY)-4-TRIFLUOROMETHYL]BENZENE
Structure	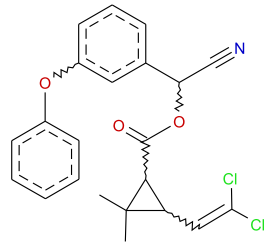	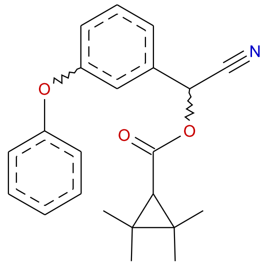	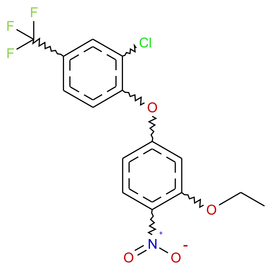
Actual Endpoint (-log C)	3.774	4.288	1.859
Predicted Endpoint (-log C)	3.49395	3.16879	2.53827
Distance	0.471	0.496	0.503
Reference	85JFAN A649;86	PSSCBG 8;579;77	85ARAE 2;201;77

Model Applicability

Unknown features are fingerprint features in the query molecule, but not found or appearing too infrequently in the training set.

1. All properties and OPS components are within expected ranges.
2. Unknown ECFP_2 feature: -435589429: [*]C[c]1:o:[*]:[*]:n:1
3. Unknown ECFP_2 feature: 1651701028: [*]OC(=C([*])[*])O[*]
4. Unknown ECFP_2 feature: -785659985: [*][c]1:[*]:[*]:o:c:1
5. Unknown FCFP_6 feature: 16: [*]:[cH]:[*]
6. Unknown FCFP_6 feature: 1747237384: [*][c](:[*]):n:[cH]:[*]
7. Unknown FCFP_6 feature: -1678245750: [*]OC(=C([*])[*])[c](:[*]):[*]
8. Unknown FCFP_6 feature: -1539162406: [*]C([*])([*])[c]1:o:[*]:[*]:n:1
9. Unknown FCFP_6 feature: -2115241127: [*]OC(=C([*])[*])O[*]
10. Unknown FCFP_6 feature: 451371068: [*]\C=C\[c](:[*]):[*]
11. Unknown FCFP_6 feature: 1618154665: [*]:[cH]:[cH]:[cH]:[*]

Feature Contribution

Top features for positive contribution			
Fingerprint	Bit/Smiles	Feature Structure	Score

ECFP_6	642810091	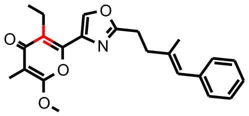 [*]:[c](:[*]):[*]	0.281
ECFP_6	-560785749	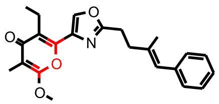 [*]C(=[*])OC(=[*])[*]	0.259
ECFP_6	1571214559	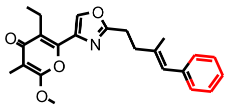 [*]1:[cH]:[cH]:[cH]:[cH]:[cH]:1	0.19
**Top Features for negative contribution**			
**Fingerprint**	**Bit/Smiles**	**Feature Structure**	**Score**
ECFP_6	2106656448	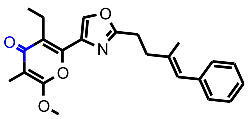 [*]C(=O)[*]	-0.352
ECFP_6	683445015	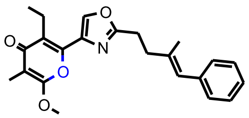 [*]O[*]	-0.266

ECFP_6	655739385	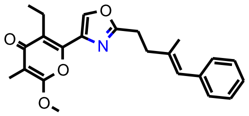<br>[*]:n:[*]	-0.239

Remdesivir

TOPKAT_Rat_Oral_LD50

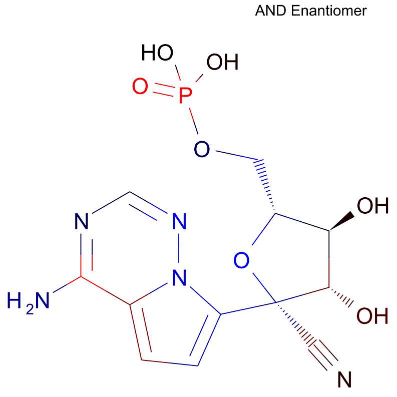

$C_{12}H_{14}N_5O_7P$
Molecular Weight: 371.24262
ALogP: -1.502
Rotatable Bonds: 4
Acceptors: 11
Donors: 5

Model Prediction

Prediction: 0.309
Unit: g/kg_body_weight
Mahalanobis Distance: 29.4
Mahalanobis Distance p-value: 1.72e-059

Mahalanobis Distance: The Mahalanobis distance (MD) is a generalization of the Euclidean distance that accounts for correlations among the X properties. It is calculated as the distance to the center of the training data. The larger the MD, the less trustworthy the prediction.
Mahalanobis Distance p-value: The p-value gives the fraction of training data with an MD greater than or equal to the one for the given sample, assuming normally distributed data. The smaller the p-value, the less trustworthy the prediciton. For highly non-normal X properties (e.g., fingerprints), the MD p-value is wildly inaccurate.

Structural Similar Compounds

Name	5'-ADENYLIC ACID; POTASSIUM SALT (K STRIPPED)	INOSINATE; DISODIUM SALT (Na STRIPPED)	INOSINE-5'-PHOSPHORIC ACID
Structure	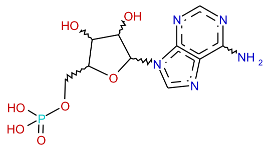	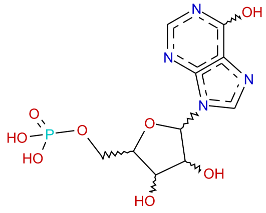	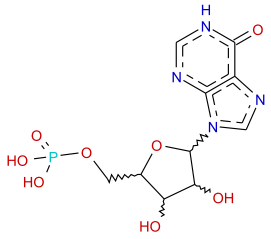
Actual Endpoint (-log C)	1.49	1.34	1.338
Predicted Endpoint (-log C)	2.45569	2.92201	1.35922
Distance	0.361	0.428	0.592
Reference	OYYAA2 4;689;70	AJINO* -;-;73	ARTODN 47;77;81

Model Applicability

Unknown features are fingerprint features in the query molecule, but not found or appearing too infrequently in the training set.

1. OPS PC10 out of range. Value: 15.526. Training min, max, SD, explained variance: -6.0395, 14.892, 2.468, 0.0220.
2. Unknown ECFP_2 feature: 1258791451: [*][C@H]1[*][*]O[C@]1(C#[*])[c](:[*]):[*]
3. Unknown ECFP_2 feature: -264833661: [*]C([*])([*])C#N
4. Unknown ECFP_2 feature: -66263742: [*]C([*])([*])[c]1:n(:[*]):[*]:[*]:c:1
5. Unknown FCFP_6 feature: 16: [*]:[cH]:[*]
6. Unknown FCFP_6 feature: 472180098: [*]OP(=O)(O)O
7. Unknown FCFP_6 feature: -836603894: [*]:[c](:[*])[C@]1(C)C[*][*]=N1
8. Unknown FCFP_6 feature: -332197802: [*][c]1:[*]:[*]:[c](:[*]):n:1:n:[*]
9. Unknown FCFP_6 feature: 4427049: [*]:[cH]:n:n(:[*]):[*]
10. Unknown FCFP_6 feature: -124685461: [*]1:[*]:s:[cH]:n:1
11. Unknown FCFP_6 feature: 1747237384: [*][c](:[*]):n:[cH]:[*]
12. Unknown FCFP_6 feature: -1151884458: [*]:n:[c](N):[c](:[*]):[*]
13. Unknown FCFP_6 feature: 1618154665: [*]:[cH]:[cH]:[cH]:[*]
14. Unknown FCFP_6 feature: 1069584379: [*]:[c](:[*])N

Feature Contribution

Top features for positive contribution

Fingerprint	Bit/Smiles	Feature Structure	Score
ECFP_6	642810091	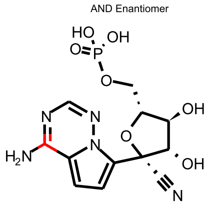 [*]:[c](:[*]):[*]	0.281
ECFP_6	-826638028	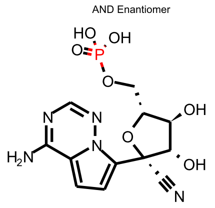 [*]P(=[*])([*])[*]	0.225
ECFP_6	2100964382	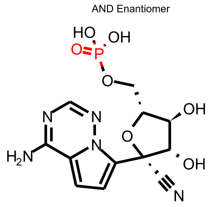 [*]P(=O)([*])[*]	0.166

Top Features for negative contribution

Fingerprint	Bit/Smiles	Feature Structure	Score
ECFP_6	683445015	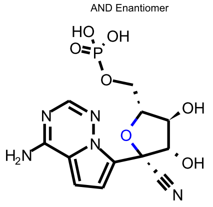 [*]O[*]	-0.266

ECFP_6	655739385	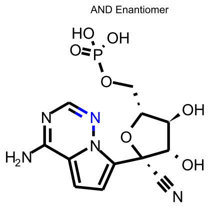 [*]:n:[*]	-0.239
FCFP_6	-1539132615	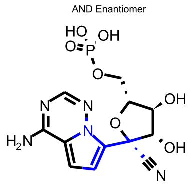 [*]:n:[c](C):[c](:[*]):[*]	-0.2
